# Electrochemical Deoxygenative Silylation of Alcohols

**DOI:** 10.1002/anie.202508697

**Published:** 2025-08-11

**Authors:** Piret Villo, Malin Lill, Ziwei Fan, Kevin Breitwieser, Jai White, Sergio Pérez Morente, Mårten Ahlquist, Helena Lundberg

**Affiliations:** ^1^ Department of Chemistry KTH Royal Institute of Technology Stockholm SE‐10044 Sweden

**Keywords:** Alcohol, Carbanion, Carboxylation, Organic electrosynthesis, Reaction mechanism, Silylation

## Abstract

Alcohols are highly common organic compounds but remain scarce as alkyl donors in synthetic procedures. Here, we describe an electrochemical procedure for their deoxygenative cross‐electrophile coupling with hydrosilanes, furnishing organosilane products in good to excellent yields. Mechanistic studies provide insights into the operating pathways of this semi‐paired electrolytic transformation, suggesting that silyl ethers are likely reaction intermediates. Furthermore, a unified mechanistic proposal is presented that accounts for observed reactivity differences with analogous deoxygenative electrocarboxylation.

## Graphical abstract

Herein, electroreductive deoxysilylation of benzylic and allylic alcohols is demonstrated. The cathodic transformation proceeds via carbanionic intermediates and can be extended to aldehydes and ketones. Mechanistic studies reveal that the reaction outcome is dependent on the thermodynamics and kinetics for the coupling step in combination with the reductive stability of the coupling partner, thereby supporting a unified mechanism to that of deoxygenative cross‐coupling with CO_2_ as electrophile.

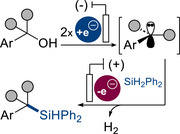

## Introduction

Alcohols represent a highly abundant compound class among both natural compounds as synthetic products, including pharmaceuticals,^[^ ^1^, ^2^, ^3^ ^]^ and their hydroxyl group represents a ubiquitous handle for further synthetic manipulations. Due to the strength of the C─OH bond, protocols to cleave these for subsequent use of the carbon backbone in cross‐coupling reactions are scarce.^[^ ^4^, ^5^, ^6^ ^]^ Instead, stoichiometric derivatization of the alcohol in or ex situ into, e.g., sulfonate/xanthate/oxalate/acetate/toluate esters, alkoxyphosphonium species or NHC‐adducts is a common go‐to strategy to enable C─O cleavage in both radical and polar manifolds.^[^ ^7^, ^8^, ^9^, ^10^, ^11^, ^12^, ^13^, ^14^, ^15^, ^16^, ^17^, ^18^ ^]^ Though effective, these stoichiometric strategies result in poor atom economy. Thus, methods that enable the use of non‐derivatized alcohols as alkyl donors remain a great synthetic quest, highlighted by the ACS Pharmaceutical Roundtable as a Key Green Research Area.^[^ ^19^ ^]^ Recently, elegant catalytic strategies were developed that circumvent the need for such stoichiometric derivatization of alcohols. In a two‐electron manifold, Denton and co‐workers developed a phosphonium‐based catalyst that enables Mitsunobu‐type substitutions of the hydroxyl group in primary and secondary alcohols with carboxylic acid and phenol derivatives, sulfonamides and thiobenzoic acid as nucleophiles.^[^ ^20^ ^]^ In a radical setting, Shu and co‐workers demonstrated that dehydroxylative C─C bond formation could be achieved for benzylic, allylic, and tertiary alcohols, including naturally occurring saccharides,^[^ ^21^ ^]^ using Ti(III)‐catalysis and powdered metals as a terminal reductant.^[^ ^22^, ^23^, ^24^, ^25^, ^26^, ^27^, ^28^ ^]^ In recent years, synthetic organic electrochemistry has re‐emerged as a promising strategy for atom‐efficient transformations that utilize electricity as a terminal redox reagent. This strategy has enabled a variety of synthetic protocols for C─O bond cleavage in alcohol derivatives,^[^ ^29^, ^30^, ^31^, ^32^, ^33^, ^34^ ^]^ whereas the number of methods available for non‐derivatized alcohols remain scarce. Currently, these electrochemical transformations are limited to dehydroxylative C─H bond formation,^[^ ^35^, ^36^, ^37^, ^38^, ^39^ ^]^ carboxylation with CO_2_,^[^ ^35^, ^40^, ^41^ ^]^ and borylation with pinacolborane.^[^ ^42^ ^]^

**Figure 1:**
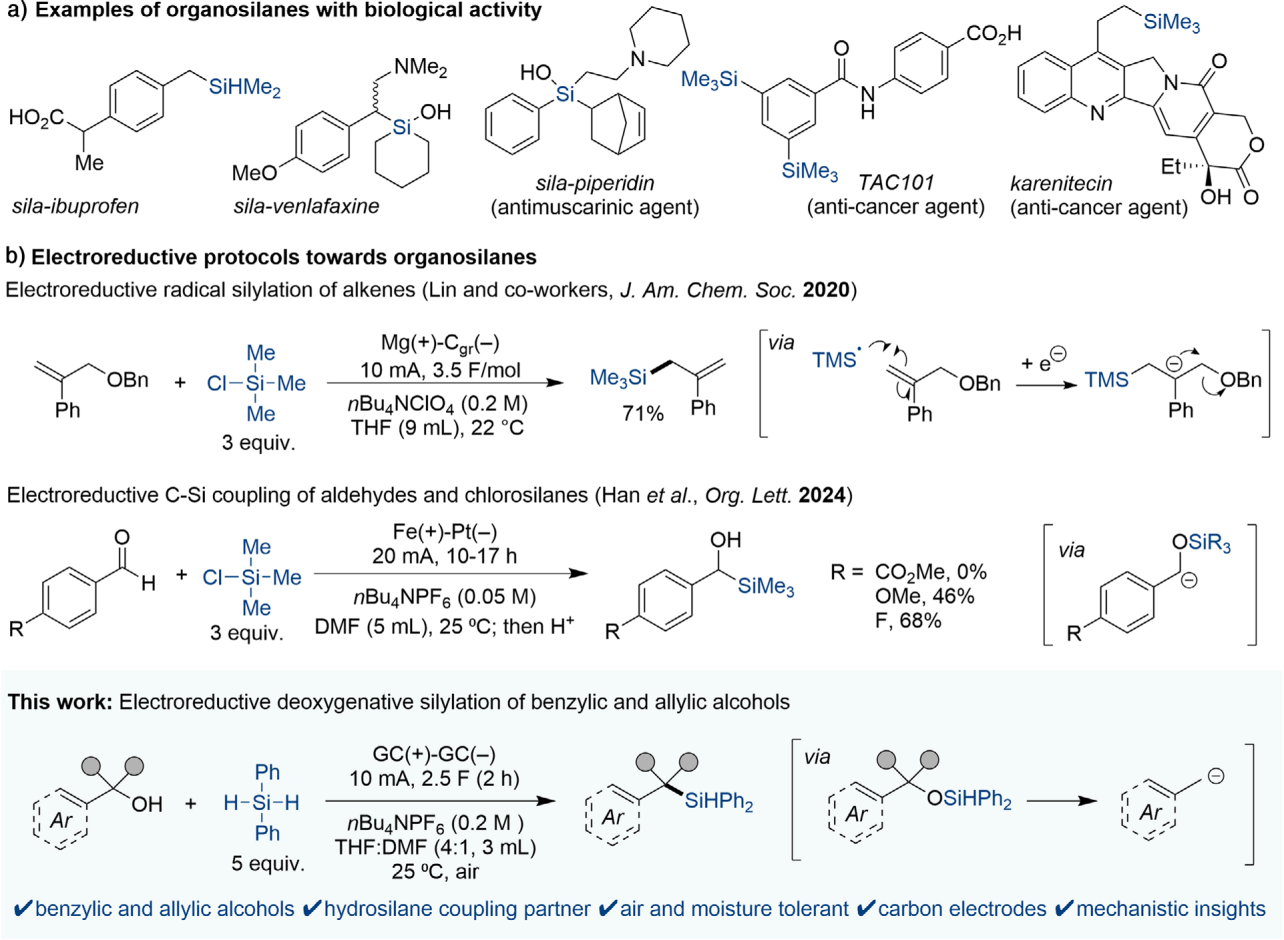
a) Examples of bioactive organosilanes. b) Electroreductive protocols forming organosilanes: silylation of alkenes by Lin and co‐workers (top), formation of *α*‐silyl alcohols by Han et al. (middle), **This work**: deoxygenative coupling of benzylic and allylic alcohols with diphenylsilane (bottom).

Organosilane compounds are of great interest for a variety of fields, including organic synthesis, material science and medicinal chemistry.^[^ ^43^, ^44^, ^45^ ^]^ In the context of the former, functionalization of Si─H bonds is an expanding area,^[^ ^46^, ^47^, ^48^, ^49^, ^50^, ^51^ ^]^ and hydrosilanes can act as directing groups for, e.g., remote *para* C─H functionalization,^[^ ^52^ ^]^ *meta* C─H olefination,^[^ ^53^ ^]^ and enantioselective borylation.^[^ ^54^ ^]^ Furthermore, allylsilanes^[^ ^55^ ^]^ are established nucleophiles for C─C bond formation with carbonyl compounds^[^ ^56^, ^57^ ^]^ whereas aryl‐ and alkylsilanes can be used as coupling partners in transition metal catalyzed transformations in both polar and radical manifolds.^[^ ^58^, ^59^, ^60^, ^61^, ^62^ ^]^ In the context of medicinal chemistry, biologically active compounds containing at least one C─Si bond have been demonstrated to possess new or elevated pharmacological potency compared to their C─C analogues,^[^ ^63^ ^]^ including sila‐ibuprofen,^[^ ^64^ ^]^ sila‐venlafaxine,^[^ ^65^, ^66^ ^]^ and sila‐biperiden^[^ ^67^, ^68^ ^]^ as well as potent anti‐cancer agents, the anti‐cancer agent TAC101^[^ ^69^ ^]^ and karenitecin^[^ ^70^ ^]^ (Figure 1a). Silicon‐containing moieties are also investigated for ^18^F‐labelling of amino acids^[^ ^71^, ^72^ ^]^ and peptides.^[^ ^73^ ^]^ There are several synthetic routes to organosilane compounds, encompassing polar metal‐catalyzed silylation of alkenes with hydro‐ or chlorosilanes,^[^ ^74^, ^75^ ^]^ nucleophilic substitution of chlorosilanes^[^ ^59^ ^]^ or benzylic ether^[^ ^76^ ^]^ with organometallic reagents, requiring excess of Grignard reagents or a secondary reductant, as well as radical additions of open shell silyl‐species to heteroarenes^[^ ^77^, ^78^ ^]^ and alkenes.^[^ ^79^, ^80^, ^81^, ^82^, ^83^, ^84^, ^85^ ^]^ Allyl sulfones, acetates and carbamates were recently demonstrated to be feasible for silylation under organophotocatalytic conditions using silanecarboxylic acids as the silyl source to furnish allylsilane products.^[^ ^85^ ^]^ In an electrochemical setting, Lin and co‐workers disclosed a silylation protocol for alkenes proceeding via electroreductive formation of a silyl radical from chlorosilanes (Figure 1b, top).^[^ ^86^, ^87^ ^]^ In addition, electrochemically driven reductive cross‐electrophile couplings of alkyl bromides and chlorosilanes was demonstrated by the same group.^[^ ^88^ ^]^ Chlorosilanes were also used as electrophiles in the electroreductive formation of *α*‐silyl alcohols from aromatic aldehydes by Han et al. (Figure 1b, middle).^[^ ^89^ ^]^ Waldvogel and colleagues demonstrated a boron‐catalyzed electrochemical coupling of hydrosilanes with benzyl‐ or allyl chlorides.^[^ ^90^ ^]^ In this work, we present an electrochemical protocol for the first dehydroxylative silylation of non‐derivatized benzylic and allylic alcohols (Figure 1b, bottom). This air‐tolerant process utilizes glassy carbon (GC) electrodes and diphenylsilane as electrophile, effectively expanding the yet limited scope of deoxygenative cross‐coupling reactions for non‐derivatized alcohols. Experimental and computational studies provide mechanistic insights that explain reactivity and selectivity differences compared to that observed for analogous dehydroxylative carboxylation. This work was carried out in parallel with, but separate from, the related work from the groups of Lin and Xu.^[^ ^91^, ^92^ ^]^

## Results and Discussion

Motivated by the limited number of available methods for electrochemically driven dehydroxylative transformations of non‐derivatized alcohols,^[^ ^29^, ^37^, ^42^ ^]^ we set out to explore the scope of this transformation class. While our previous studies had demonstrated that oxidative dissolution of a Zn anode or borohydride oxidation at a carbon‐based electrode were effective anodic counter electrode reactions for such dehydroxylative transformations,^[^ ^35^, ^93^ ^]^ we had also observed that anodic oxidation of tetrahydrofuran (THF) appeared as a feasible alternative. These findings aligned with previous literature on electrosynthetic transformations in this ethereal solvent,^[^ ^94^, ^95^, ^96^, ^97^ ^]^ and, thus, we set out to explore dehydroxylative cross‐couplings using THF oxidation as tentative counter reaction. Our initial attempts using chlorosilanes as coupling partners to benzylic alcohols failed to furnish any C─Si cross‐coupling products. For this reason, we decided to probe hydrosilanes as alternative electrophiles due to their expected higher cathodic stability compared to their chloride congeners.^[^ ^98^ ^]^ While hydrosilanes are commonly used as reducing agents due to the hydridic nature of the Si─H bond,^[^ ^99^, ^100^, ^102^ ^]^ a handful of early protocols report on their use as electrophiles with Grignard reagents.^[^ ^103^, ^104^ ^]^ In addition, the hydrosilanes’ lower propensity for hydrolysis suggested that the need for strictly anhydrous conditions could be circumvented.^[^ ^105^ ^]^ To our delight, the use of diphenylsilane did result in traces of the silylated product **2a** from *p‐*methoxybenzyl alcohol (**1a**) (Table 1, entry 1). By increasing the silane equivalence from 1 to 3 and then to 5 resulted in increased yield of product **2a** (Table 1, entries 1–3). Notably, similar reaction outcomes were achieved when either glassy carbon (GC) or less costly graphite (C_gr_) were used as electrode materials (Table 1, entries 3 versus 4). Alas, the product yield was still moderate and associated with the build‐up of a jelly‐like layer on the anode and concomitant electrode passivation. This issue was circumvented by the addition of *N,N*‐dimethylformamide (DMF) as co‐solvent, resulting in a dramatically increased yield of product **2a** and clean electrodes by visual inspection at the end of the reaction (Table 1, entry 5). Pleasingly, the reaction proved to be relatively robust, tolerating air and non‐dry conditions, omitting the use of molecular sieves (MS) and inert atmosphere (Table 1, entry 6, for further details see Supporting Information). While a more environmentally benign solvent mixture of 2‐methyltetrahydrofuran (2‐Me THF) and *N,N′*‐dimethylpropylene urea (DMPU) could be used equally well to THF:DMF system (Table 1, entry 7), we continued with the latter for ease of experimental setup (see Supporting Information). By extending the reaction time to 3 h, nearly full conversion of the starting material **1a** and 90% yield of product **2a** was observed (Table 1, entry 8), with similar results obtained using 10 equivalents of silane (Table 1, entry 9). No reaction took place in the absence of electric current, clearly demonstrating that the dehydroxylative silylation is electrochemically driven (Table 1, entry 10).

**Table 1:** Evaluation of reaction conditions for deoxygenative silylation.

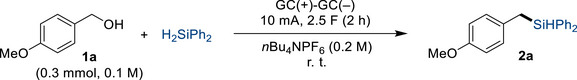					
	*Reaction conditions*				
Entry	Ph_2_SiH_2_ (equiv.)	Time (h)	Solvent (ratio)	Other conditions	2a yield (%)^a)^
1^b)^	1	2	THF	4 Å MS, N_2_ atm.	3
2^b)^	3	2	THF	4 Å MS, N_2_ atm.	30^c)^
3^b)^	5	2	THF	4 Å MS, N_2_ atm.	38
4^b)^, ^d)^	5	2	THF	4 Å MS, N_2_ atm., C_gr_‐C_gr_	41
5^b)^	5	2	THF:DMF (5:1)	4 Å MS, N_2_ atm.	73
6	5	2	THF:DMF (5:1)	air atm.	76
7	5	2	2‐Me THF:DMPU (5:1)	air atm.	71
8^e)^	5	3	THF:DMF (5:1)	air atm.	90
9^e)^	10	3	THF:DMF (4:1)	air atm.	91
10	10	3	THF:DMF (4:1)	no current	–

^a)^ Yields assessed by ^1^H‐NMR analysis with 1,3,5‐trimethoxybenzene as internal standard.

^b)^ Molecular sieves (powder, 4 Å, 50 mg), under nitrogen atmosphere.

^c)^ Isolated yield.

^d)^ Graphite was used as anode and cathode material.

^e)^ 3.7 F (3 h).

**Figure 2:**
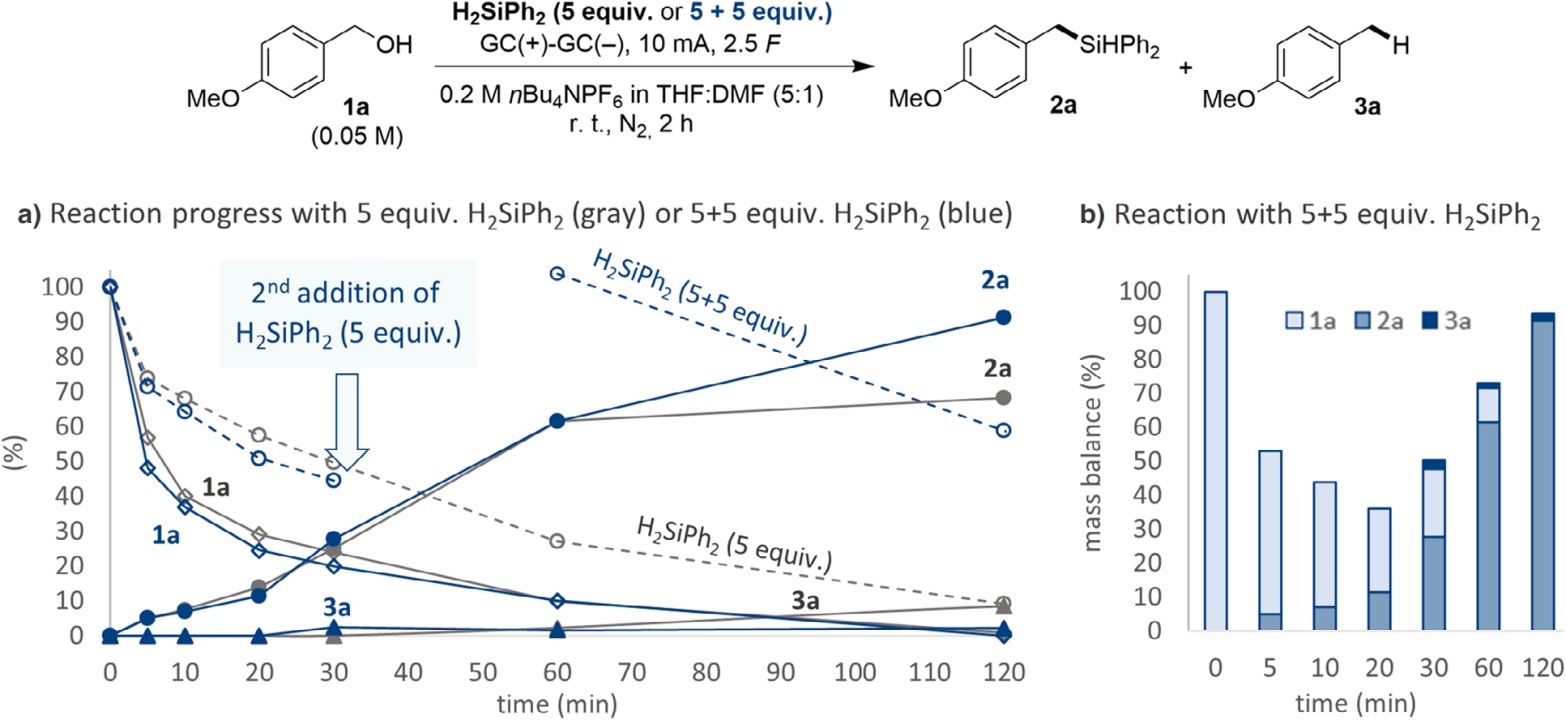
Time‐resolved study for the deoxygenative silylation of **1a**. a) Comparison of a reaction using 5 equiv. H_2_SiPh_2_ (gray) with a second reaction, to which additional 5 equiv. of H_2_SiPh_2_ was added after 30 min (blue). Diphenylsilane (5 equiv.) normalized to 100%. b) Mass balance for deoxygenative silylation of **1a** using 5 + 5 equiv. H_2_SiPh_2_ (blue), showing consumption of **1a** and formation of **2a** and **3a** in (%). Conditions: a mixture of **1a** (0.15 mmol, 1 equiv.), diphenylsilane (5 equiv.) and *n*Bu_4_NPF_6_ (2 equiv.) is stirred in THF:DMF (5:1, 3 mL) at room temperature for 2 h, with glassy carbon electrodes at 10 mA constant current electrolysis under nitrogen atmosphere. Reaction progression was assessed by HPLC analysis.

To probe the origin of the need for excess diphenylsilane, the reaction was followed over time by sampling and off‐line HPLC‐analysis (see Supporting Information for details). With 5 equivalents of diphenylsilane, the reaction proceeded with a steady consumption of both **1a** and diphenylsilane, along with the formation of the silylated product **2a** (Figure 2a). Approximately 20 min into the reaction, formation of side‐product **3a** was observed along with a slower formation of **2a**. This change in selectivity correlated with a significant reduction in silane concentration, indicating that protons had become competitive coupling partners at this time. Based on the findings in our previous work on deoxygenative electroreduction of benzyl alcohols,^[^ ^35^ ^]^ such protons may originate from the alcohol itself, from the solvent, or from the tetrabutylammonium cation of the supporting electrolyte via Hofmann elimination. To probe whether the build‐up of side‐product **3a** could be suppressed, a second reaction was carried out under identical conditions but to which an additional 5 equivalents of diphenylsilane were added after 30 min (Figure 2a). At this point in time, traces of **3a** were observed, similar to the original reaction. However, subsequent to the second addition of diphenylsilane, further formation of **3a** was suppressed while the silylated product concentration **2a** increased. This behavior is consistent with the two products forming from one common intermediate that is able to react with various electrophiles. Furthermore, the time‐study revealed a rapid decrease of the mass balance, measured as the combined concentrations of **1a**, **2a**, and **3a**, in the early stages of the reaction but with a steady recovery over time (Figure 2b) towards in situ formed intermediate species in the transformation (vide infra).

**Figure 3:**
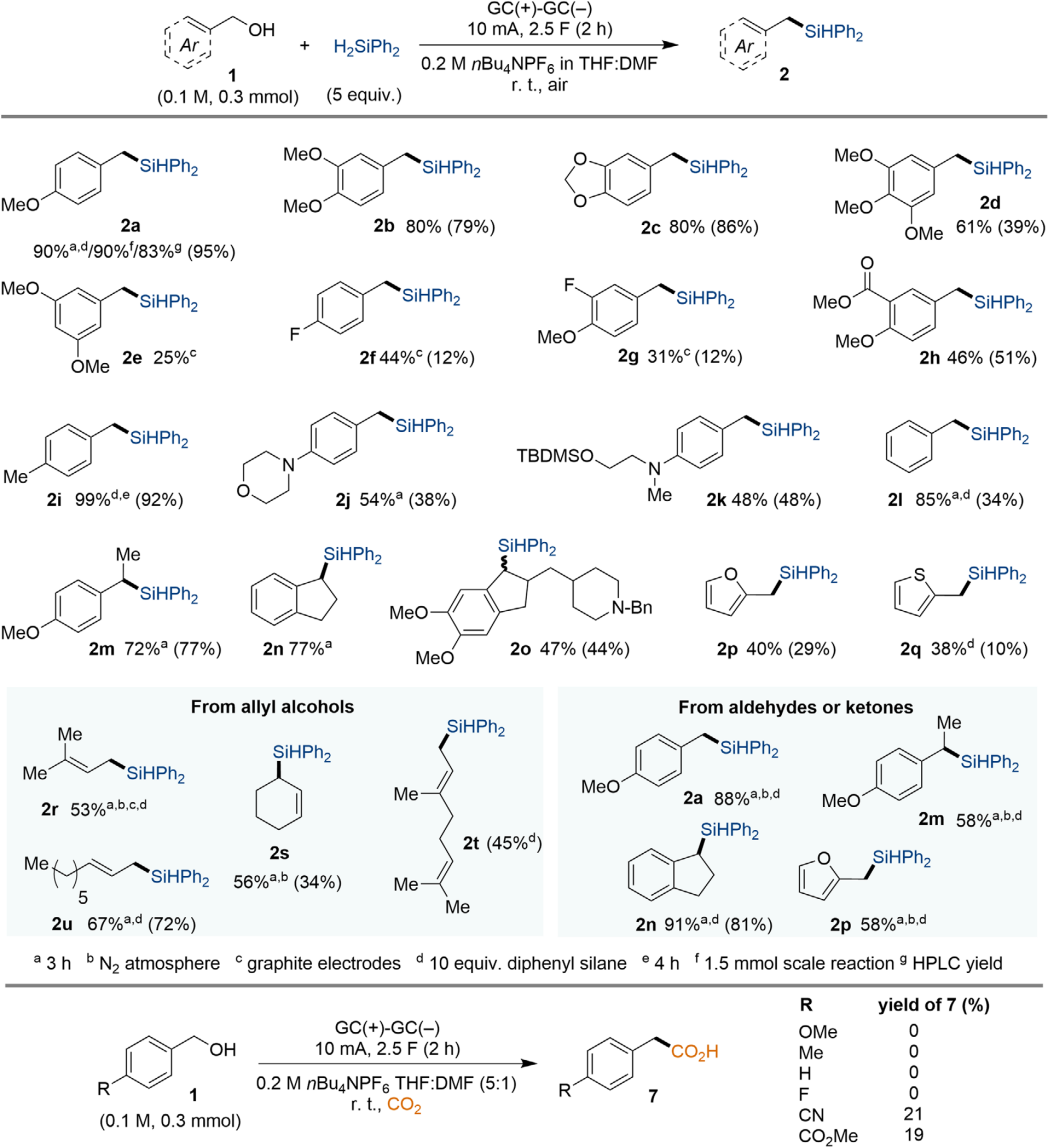
Scope for electroreductive deoxygenative cross‐couplings. Top: Deoxygenative silylation. Conditions: Alcohol (0.3 mmol, 1 equiv.), diphenylsilane (5 equiv.) and *n*Bu_4_NPF_6_ (2 equiv.) is stirred in THF:DMF (5:1, 3 mL) at room temperature at 10 mA for 2 h, sealed in air. Yields were determined using ^1^H‐NMR analysis with 1,3,5‐trimethoxybenzene as internal standard or via isolation (in parenthesis). Bottom: Deoxygenative carboxylation. Conditions: A mixture of alcohol (0.3 mmol, 1 equiv.), and *n*Bu_4_NPF_6_ (2 equiv.) is stirred in THF:DMF (5:1, 3 mL) at room temperature at 10 mA for 2 h, sealed under CO_2_. Yields were determined via isolation.

Using the optimized reaction conditions, we turned to assess the generality of the transformation (Figure 3). When the benchmark benzyl alcohol (**1a**) was equipped with an additional methoxy group in *meta*‐position, the product yields were considerably lower for the corresponding silylated products **2b‐e** compared to benchmark product **2a**. This trend is consistent with the π‐donating and σ‐withdrawing capacity of methoxy groups,^[^ ^106^ ^]^ pointing to the need for electron‐rich arene backbones. As such, the moderate yield of product **2f** may be explained by the π‐donating ability of the *para*‐positioned fluoride that balances its strong inductively withdrawing effect, whereas a somewhat lower yield was achieved for **2g** that bears the fluoride in *meta*‐position and a methoxy group in *para*‐position. For the analogous *meta*‐fluoride substituted benzyl alcohol with a less donating phenyl group in *para*‐position, the yield of the corresponding product resulted in mere 5% (see Supporting Information). Along similar lines, a π‐accepting methyl ester group was tolerated when situated in *meta*‐position to furnish **2h** in good yield, whereas its *para*‐regioisomer failed to furnish the corresponding silylated product (see Supporting Information). Consistent with these results, the *para*‐methyl substituted product **2i**, as well as the amine‐containing products **2j** and **2k**, resulted in good to excellent yields. Benzyl alcohol furnished product **2l** in a satisfactory yield, and the reaction tolerated secondary alcohols as starting materials well (**2m** and **2n**), although sterically hindered backbones resulted in moderate yields, e.g., in **2o** from bioactive dihydro‐donepezil. Deoxysilylation was also possible for heteroaromatic alcohols, furnishing products **2p** and **2q** in moderate yields. Satisfyingly, allyl silanes **2r** to **2u** could be synthesized in good yields from their respective allyl alcohols. Furthermore, aldehydes and ketones could be used to furnish deoxysilylation products **2a**, **2m**, **2n** and **2p**. The conversion of benchmark substrate **1a** into product **2a** could successfully be scaled from 0.3 mmol to 1.5 mmol without loss in yield, whereas further scale‐up required modifiation of reaction conditions (see Supporting Information). The use of differently substituted silanes, as well as alcohols including electron‐poor benzylic substrates, *N*‐heterocyclic substrates and aliphatic alcohols resulted in low or no yield of the targeted silylated products (see Supporting Information). In some of these cases, traces of butyldiphenylsilane were instead observed, likely originating from reaction of the silane reagent with the supporting electrolyte (see Supporting Information). Interestingly, isochroman did not react under the silylation conditions and 99% of the starting material was retained after 3 h. This stands in contrast to our previous findings, where benzylic ethers would readily undergo C─O bond cleavage.^[^ ^35^ ^]^ The silylated products can be transformed into the corresponding silanols and carboxylic acids (see Supporting Information).^[^ ^107^, ^108^, ^109^ ^]^ Furthermore, the transformation of benzylic silanes into the corresponding homologated carboxylic acids by reaction with CO_2_ as well as their cross‐coupling with aryl nitriles, aryl chlorides and aryl sulfonates has been reported.^[^ ^110^, ^111^ ^]^ Interestingly, whereas the deoxysilylation required electron‐rich alcohols to furnish the targeted products while electron‐poor analogues failed, the opposite trend was observed for deoxycarboxylation with CO_2_ as alternative electrophile under otherwise identical conditions (Figure 3, bottom).

### Mechanistic Considerations

**Figure 4:**
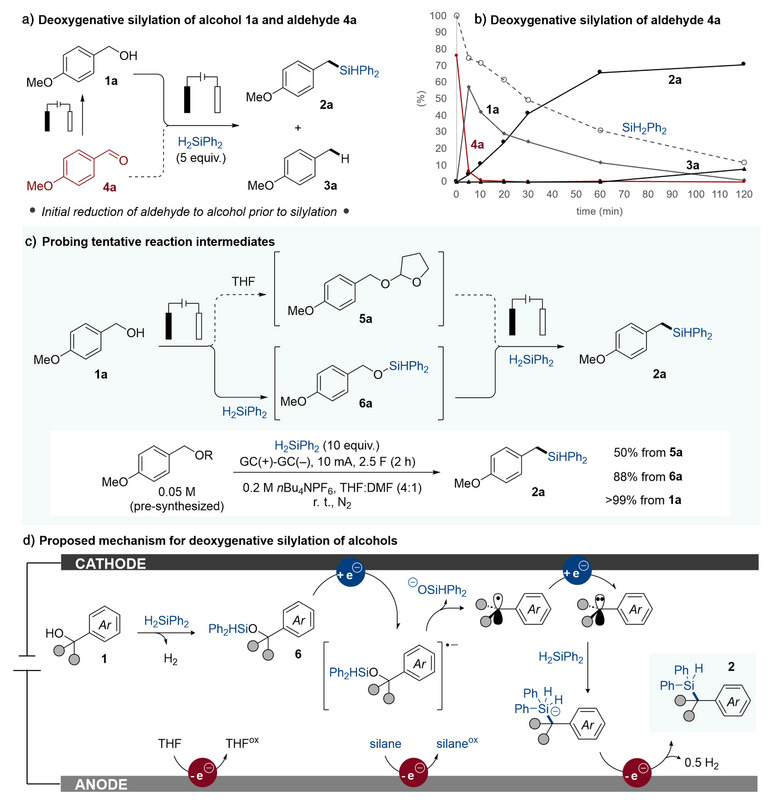
Mechanistic considerations. a) Electroreductive silylation of **1a** (0.05 M) or **4a** (0.05 M) under standard conditions, analysed by ^1^H‐NMR (1,3,5‐trimethoxybenzene as internal standard). b) Electroreductive silylation of **4a** (0.05 M), traced over time by sampling and HPLC analysis. Diphenylsilane (5 equiv.) was normalized to 100%. c) Assessment of tentative reaction intermediates **5a** and **6a** for the formation of product **2a**. d) Proposed reaction mechanism. See Supporting Information for details on synthetic procedures.

Since aldehydes and ketones were found able to furnish the corresponding deoxysilylated products (Figure 3), the reaction mechanism may be envisioned to proceed via initial anodic oxidation of the alcohol substrates. However, when monitoring the deoxysilylation over time using either **1a** or **4a** as starting material, it was found that the latter rapidly reduced to the former (Figure 4a,b). This finding strongly suggests that the deoxygenative silylation does not proceed via alcohol oxidation. Instead, we turned our attention to acetal **5a** as a potential reaction intermediate (Figure 4c), since this species had been isolated from a reaction in the early screenings for optimal reaction conditions (see Supporting Information). In addition, electrosynthesis of a scope of THF‐acetals from a variety of alcohols had previously been reported by others under similar conditions to ours.^[^ ^112^ ^]^ To probe the hypothetical role of acetal **5a** as a reaction intermediate in our deoxygenative silylation, we synthesized and assessed the THF‐acetal as well as a related THP‐acetal under the conditions used for the substrate scope in Figure 3. Both these acetals were able to form the corresponding silylated products, however, in consistently lower yields compared to their parent alcohol (see Supporting Information). In addition, the reduction potential of acetal **5a** was determined by means of DFT calculations to be more cathodic compared to the parent alcohol **1a** (see Supporting Information). These synthetic and computational findings combined suggested that THF‐acetals are not key intermediates in the deoxygenative silylation of alcohols. Nevertheless, the observed formation of **5a** indicates that THF oxidation takes place at the anode.

Next, we assessed another possible reaction intermediate: the silyl ether **6a** (Figure 4c). Such silyl ethers have previously been reported to form under similar electrochemical conditions to ours,^[^ ^113^ ^]^ and their hypothetical formation was thought to partially explain the need for excess silane reagent (Table 1) as well as the poor mass balance observed in the intermediate stages of the reaction (Figure 2). In support of this hypothesis, the reduction potential of the tentative intermediate **6a** was found to be more anodic compared to alcohol **1a** as determined by DFT and cyclic voltammetry (CV), with **6a** formed via a telescoped synthesis (see Supporting Information for details). Furthermore, the use of **6a** as starting material for deoxygenative silylation resulted in **2a** in good yields (Figure 4c), as did its more hydrolytically stable analogue (see Supporting Information, Section 8.3). These combined findings suggest that silyl ethers are plausible reaction intermediates in the deoxygenative silylation. A control experiment in the absence of current did not result in conversion of alcohol **1a** into detectable intermediates or products (Table 1, entry 10 and Supporting Information, Section 3.10), indicating that such formation of tentative silyl ether intermediates is electrochemically driven. Considering the conditions required for non‐electrochemical synthesis of **6a**,^[^ ^114^ ^]^ it appears likely that this silyl ether can form under our conditions upon reaction of the hydrosilane with an electrogenerated alkoxide base, formed from **1a** via cathodic proton reduction.

Based on the combined findings, we propose that the deoxygenative silylation of benzylic and allylic alcohols proceeds via cathodic reduction of an intermediate in situ formed silyl ether to generate a radical anion that decomposes into a silanolate and a carbon centered radical (Figure 4d).^[^ ^35^, ^115^ ^]^ At the applied potentials, this open‐shell species undergoes a rapid reductive radical‐polar crossover to form a carbanion that reacts with the diphenylsilane electrophile.^[^ ^35^, ^116^ ^]^ The resulting hydridosilicate intermediate is proposed to oxidize at the anode to generate product **2** and hydrogen in a semi‐paired electrolytic sequence, similar to the proposed mode of action for analogous borohydride species.^[^ ^93^, ^117^, ^118^ ^]^ However, with the known ability of hydridosilicates to undergo hydride transfer to an available hydride acceptor,^[^ ^119^, ^120^ ^]^ the formation of product **2** along with H_2_ via a chemical route cannot be ruled out. The proposed rapid radical‐polar crossover is supported by control experiments (see Supporting Information). Interestingly, the silylation only exhibited yields below 30% when using benzyl bromide or chloride as alternative carbanion precursors, highlighting the benefit of the present transformation (see Supporting Information). For the anodic counter reaction, oxidation of the hydridosilicate intermediate, THF and diphenylsilane is proposed to provide the required electrons for the net reductive product formation (see Supporting Information). Oxidative degradation of the silane may be expected to contribute to the rapid silane consumption seen in Figure 2, in conjunction with the formation of intermediate **6a** and, potentially, with formation of silanols with residual water and/or fluorosilanes with fluorides from the supporting electrolyte.^[^ ^121^, ^122^ ^]^ In addition, contributions from electroreductive decomposition of diphenylsilane cannot be ruled out due to its electroactivity at potentials close to that of the proposed reaction intermediate **6a** (vide infra). An in‐depth mechanistic study may in due time elucidate the multiple reactivities of the silane under the applied conditions.

**Figure 5:**
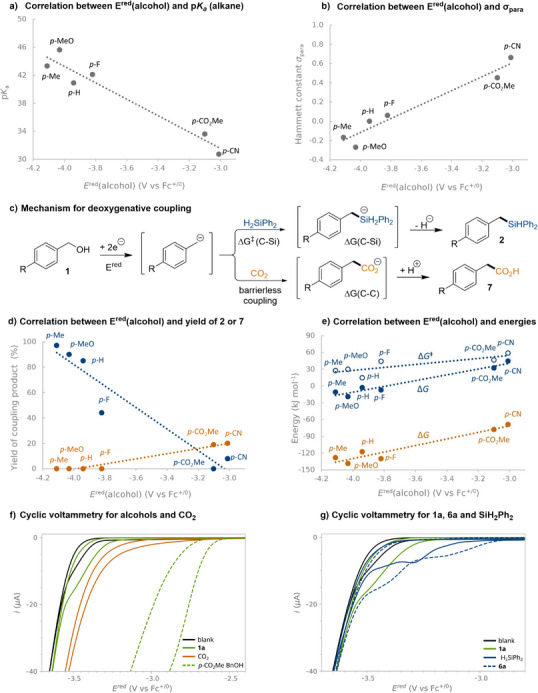
Comparison of electroreductive silylation and carboxylation of alcohols. a) Reduction potential *E* ^red^ (V versus Fc^+/0^) versus p*K*_a_ of respective benzyl alcohols; b) reduction potential *E* ^red^ (V versus Fc^+/0^) for benzylic alcohols versus Hammett constant *σ*_para_; c) Mechanism for reductive silylation and carboxylation of benzylic alcohols via carbanionic intermediate; d) reduction potential *E* ^red^ (V versus Fc^+/0^) of benzylic alcohols versus yield (%) of silylation products **2** (blue) and carboxylation products **7** (orange); e) reduction potential *E* ^red^ (V versus Fc^+/0^) for benzylic alcohols versus Gibbs free energy for the formation of silylation products (Δ*G*, blue, filled circles), reaction barrier for the C─Si coupling step (Δ*G* ^‡^, blue, hollow circles), and Gibbs free energy for the formation of carboxylation products (Δ*G*, orange filled circles). f) CVs in the cathodic region for the blank solution (0.1 M *n*Bu_4_NPF_6_ in THF:DMF [5:1]) and for solutions of 10 mM **1a**, saturated CO_2_ and 10 mM methyl 4‐(hydroxymethyl)benzoate, voltage window from −2.4 to −3.7 V versus Fc^+/0^. g) CVs in the cathodic region for the blank solution (0.1 M *n*Bu_4_NPF_6_ in THF:DMF [5:1]) and for solutions of 10 mM **1a**, 10 mM H_2_SiPh_2_ and 10 mM **6a**. Voltage window from −2.8 to −3.7 V versus Fc^+/0^. See Supporting Information for computational and experimental details.

It is well‐known that the electronic nature of benzylic alcohols is closely correlated to their p*K_a_*. In the same way, the p*K_a_* for the corresponding acid of the proposed carbanionic reaction intermediate, i.e., the alkane, may be expected to display a similar correlation. Indeed, a linear relationship can be observed when plotting the theoretically determined p*K_a_* of these alkanes versus the reduction potential of the corresponding benzylic alcohol (Figure 5a, see Supporting Information for details), suggesting a strong correlation between the reduction potential of the alcohol starting material and the reactivity of the corresponding carbanions that form upon their reductive deoxygenation. Similarly, a linear relationship is observed when the reduction potential of the benzylic alcohols is plotted against the *σ*_para_‐values of their substituents (Figure 5b).^[^ ^106^ ^]^ These linear relationships are interesting in light of the synthetic trends observed in Figure 3, demonstrating that electron‐rich alcohols are best suited for the deoxysilylation. Notably, this trend stands in stark contrast to that observed for electrocarboxylation with carbon dioxide (CO_2_), in which only electron‐poor alcohols would undergo the targeted C─C coupling (Figure 3, bottom). The need for electron deficient alcohols to achieve deoxygenative carboxylation was in line with that previously observed by our group as well as by Senboku and co‐workers.^[^ ^35^, ^40^ ^]^ To probe whether this contrasting reactivity was due to differences in reaction conditions or to the intrinsic nature of the transformations, a comparison was made for a set of alcohols using either diphenylsilane or CO_2_ as coupling partners under otherwise identical conditions (Figure 5c). As evident from Figure 5d, the opposing trends remained under these conditions: the formation of C─Si coupling products **2** was favored for electron‐rich alcohols with highly negative reduction potential and formation of C─C coupling products **7** occurred only for electron‐poor alcohols with moderate reduction potentials (Figure 5d, blue and orange series, respectively). These contrasting trends could indicate different reaction mechanisms for the two deoxygenative transformations. However, when the Gibb's free energy for the coupling of the hypothesized carbanionic intermediate with either electrophile is plotted versus the reduction potential for the alcohol starting material (Figure 5e), the trends converge: reactions with carbanions formed from electron‐rich alcohols are more energetically favoured compared to those involving less electron‐rich analogues for both silane and CO_2_ as coupling partner. For the deoxysilylation, the C─Si coupling step was determined to be an energetically downhill process only for electron‐rich benzylic anions (Figure 5e, filled blue series). Similarly, the energy barriers for the C─Si coupling step were found to decrease as the benzylic carbanionic intermediates became more electron‐rich, i.e., as the reduction potential of the parent alcohol became more cathodic (Figure 5e, hollow blue series). As such, these results suggest that the synthetically observed trend with respect to product formation could be explained by the thermodynamics and kinetics for the C─Si coupling: only in the case of highly nucleopilic, electron‐rich carbanionic intermediates would the C─Si coupling be energetically favored. Interestingly, the C─C coupling step was determined to be an energetically favored and essentially barrierless process for all benzylic carbanions (Figure 5e, orange filled series and Supporting Information), suggesting that carboxylation products should be obtained from all alcohols and not just for the electron‐poor substrates as experimentally observed. To understand this discrepancy between predicted and synthetically verified results, we turned our attention to the reduction potential of the electrophile. In the case of C─C coupling, the CO_2_ electrophile undergoes electrochemical reduction at less cathodic potentials compared to most alcohols.^[^ ^123^ ^]^ Only in the case of easily reduced electron‐poor alcohols is this electrophile expected to remain intact at the potentials required to convert the alcohol starting material into the key carbanionic intermediate and, thus, stay available for subsequent C─C coupling. Indeed, this explanation model is consistent with experimental data from cyclic voltammetry (CV) (Figure 5f), by which CO_2_ (orange line) was found to undergo reductive electron transfer at a more anodic potential compared to the methoxy‐substituted alcohol **1a** (solid green line) but at a more cathodic potential compared to the methyl ester‐decorated benzylic alcohol (dashed green line). In contrast, diphenylsilane demonstrated a slightly more cathodic onset potential compared to the electron‐rich silyl ether **6a** (Figure 5g) that was identified as the tentative carbanion precursor for the formation of benchmark product **2a** (Figure 4d). This behavior suggests that the diphenylsilane can withstand the potentials required to convert **6a** to the reactive carbanionic intermediate and thereby remain available for nucleophilic attack. Hence, we propose that the synthetically observed diverging trends for deoxygenative silylation and carboxylation with respect to product formation, respectively, result from a combination of thermodynamics and kinetics for the C─C and C─Si coupling steps (Figure 5d) and the reductive stability of the CO_2_ and diphenylsilane coupling partners (Figure 5e), rather than fundamental mechanistic differences for the transformations.

## Conclusions

Herein, a direct deoxygenative silylation of non‐derivatized alcohols is presented. The electrochemically driven transformation tolerates a variety of functional groups in the alcohol starting materials and can be extended to aldehydes and ketones. Mechanistic studies suggest that the deoxygenative process proceeds via initial formation of a silyl ether intermediate, formed in situ under electrochemical conditions. This silyl ether undergoes cathodically induced mesolytic C−O bond cleavage to form a carbon centered radical, followed by a subsequent radical‐polar crossover to the corresponding carbanion that reacts with the silane electrophile. Comparisons between the deoxygenative silylation and analogous carboxylation using CO_2_ as coupling partner revealed that a unified mechanism is likely operating, despite the orthogonal trends observed with respect to product outcome. While the coupling step between the carbanion and the diphenylsilane or CO_2_ electrophile is positively correlated with the nucleophilicity of the former with respect to both kinetics and thermodynamics, the electrophile must be able to withstand the reductive potentials required to convert the pro‐nucleophile to the carbanion intermediate. Here, we demonstrate that diphenylsilane is only able to react with electron‐rich carbanions whereas it withstands the cathodic potentials required to convert the corresponding silyl ether pro‐nucleophiles to the reactive intermediate. In contrast, CO_2_ can react with any benzylic carbanion but only withstands the reductive potentials that convert electron‐poor benzylic alcohols to the corresponding carbanions. The present work expands the limited literature on cross‐electrophile couplings of non‐derivatized alcohols, provides a new synthetic route to the formation of organosilane compounds and delivers mechanistic understanding that paves the way for further developments in the field.

## Conflict of Interests

The authors declare no conflict of interest.

## Supporting information



Supporting Information

## Data Availability

The data that support the findings of this study is found in the Supporting Information file that accompanies the article.
