# Supplementary material for: Electrochemical Deoxygenative Silylation of Alcohols
Source: Angew Chem Int Ed Engl. 2025 Aug 11;64(39):e202508697. doi: 10.1002/anie.202508697 (PMC12455427; doi:10.1002/anie.202508697)
Supplement: Supplementary file 1 — Supporting Information [file ANIE-64-e202508697-s001.pdf]

Supporting Information

Electrochemical Deoxygenative Silylation of Alcohols

Piret Villo, Malin Lill,‡ Ziwei Fan,‡ Kevin Breitwieser, Jai White, Sergio Pérez Morente, Mårten Ahlquist*, Helena Lundberg*

Department of Chemistry, KTH Royal Institute of Technology, SE-100 44, Stockholm, Sweden. E-mail: hellundb@kth.se; ahlqui@kth.se

‡ These authors contributed equally to this work

Table of contents

1. General Information 3
2. Analysis of the reactions 4
2.1. HPLC analysis 4
2.2. NMR analysis 4
2.3. Average yield and standard deviation 5
3. Electrochemical deoxygenative silylation of alcohols 6
3.1. Screening of general reaction conditions 6
3.2 Screening of co-solvents 7
3.3 Assessment of supporting electrolytes 7
3.4 Reproducibility 8
3.5 Sampling over time, assessed by HPLC analysis 9
3.6 Reactivity of alcohols with electron-withdrawing groups 10
3.7 Diphenylsilane concentration 10
3.8 Alcohol concentration 11
3.9 Zinc as sacrificial anode 12
3.10 Comparison of reactions under constant current 5 mA and 10 mA or no current 13
3.11 Other hydrosilanes 14
3.12 Scale-up silylation of 1a 14
4. General reaction procedures 15
4.1. General Procedure for electrochemical deoxygenative silylation 15
4.2. General Procedure for electrochemical deoxygenative carboxylation 15
5. Synthetic details and analytical data for products 2a-2s 16
6. Electroreductive carboxylation of alcohols 1 and 6a 24
7. Derivatization of hydrosilanes 2 25
8. Mechanistic studies 27
8.1 Formation of THF-acetals under electrochemical conditions 27
8.2 Formation of *O*-silyl ethers under electrochemical conditions 27
8.3 Electrochemical silylation of *O*-silyl ethers 28
8.4 Electrochemical silylation of THF- and THP-acetals 30
8.5. Formation of butyldiphenylsilane under electrochemical conditions 31
8.6. Mechanistic control reactions 32
9. Synthesis of 6a, 8a, 1k, 1o and THF- and THP-acetals 33
10. Unsuccessful and low-yielding alcohol substrates 36
11. Cyclic voltammetry 37

12. DFT calculations 41
13. References 126
14. Quantitative ^{1}H-NMR analysis of reaction mixtures for 2a-2s 128
15. ^{1}H-NMR and ^{13}C-NMR spectra for compounds 2a-2s and silanols 146
16. HPLC chromatograms 171

1. General Information

All reactions were carried out in oven-dried glassware. Unless stated otherwise, all solvents and chemicals were purchased from commercial suppliers and used without further purification. Supporting electrolyte, Bu_4NPF_6, was recrystallized once from EtOH/H_2O (3:1) mixture, three times from EtOH, and dried at 120 °C for 48 h under high vacuum. Anhydrous solvents like dimethylformamide (DMF), tetrahydrofuran (THF) and acetonitrile (AcCN) were dried using a solvent dispensing system under argon, where the solvent is passed through activated alumina columns, stored under N_2, and over activated 4Å molecular sieves (beads) when needed. Water used for reactions and HPLC analysis was obtained from a Milli-Q® system. All electrosynthetic reactions, including screening for parameters, were either run on IKA ElectraSyn 2.0 platforms with a GoGo module or on 4-channeled Aim-TTi MX Series multi-range power supplies, and in IKA 5 mL glass vials. The graphite electrodes (52 x 7 x 1.5) mm were cleaned first by rinsing with ethyl acetate, acetone and water, then soaked in an aqueous 1M HCl solution, followed by sonication in Milli-Q® water, acetone, and subsequent drying at room temperature under high vacuum overnight. Polishing with ultra-fine sandpaper (Bosch C355) was only used when cleaning with solvents and tissue paper did not remove inconsistencies on the electrode surface. Glassy carbon electrodes were polished with a commercially available polishing pad and alumina (Al_2O_3), followed by washing with Milli-Q® water and acetone. Molecular sieves (4Å, powder or beads) were heat gun-dried under a high vacuum for 15 minutes and cooled under N_2 prior to use. TLC analyses were performed on pre-coated silica gel 60 F254 plates, and visualized using UV light, $KMnO_4$ (3 g $KMnO_4$, 20 g K_2CO_3, 5 mL 5% NaOH (aq), 300 mL H_2O), phosphomolybdic acid stain (10% solution in EtOH) or ceric ammonium molybdate stain (12 g ammonium molybdate, 0.5 g ceric ammonium molybdate, 15 mL concentrated sulfuric acid, 235 mL water). Flash column chromatography was conducted using 40-60 µm, 230-400 mesh, 60Å silica gel or C18-RP silica gel (23%C, *ca* 1 mmol/g, particle size 40-63 µm) or neutral silica gel (treated with Et_3N) as stationary phase. Preparatory TLC purifications were conducted on pre-coated silica gel 60 F254 plates (layer thickness 250 µm) on glass and visualized using UV light. NMR spectra were recorded using either a Bruker Avance II 400 MHz or a Bruker Avance 500 MHz spectrometer at 298 K using $CDCl_3$ as solvent. Chemical shifts are given in ppm relative to the residual solvent peak (^{1}H-NMR: $CDCl_3$ δ 7.26, THF-d8 δ 1.72 and 3.58; ^{13}C-NMR: $CDCl_3$ δ 77.16, THF-d8 δ 25.31 and 67.21) with multiplicity (br = broad, s = singlet, d = doublet, t = triplet, q = quartet, quin = quintuplet, sext = sextet, m = multiplet), coupling constants (in Hz) and integration. Agilent 1260 Infinity Quaternary LC (Eclipse Plus 18C column 3.5 µm, 4.6 × 100 mm^2 ; UV detector, 265 nm) with a gradient of acetonitrile and 0.1% formic acid in Milli-Q® water at a flow rate of 1.0 mL/min, using 4,4'-di-*tert*-butylbiphenyl (DTBB) as internal standard. Either syringeless filter vials (0.5 mL) or standard glass vials (1.5 mL) were used for HPLC analysis. High-resolution mass spectrometry analyses were performed using a Thermo

Scientific Q Exactive HF Hybrid Quadrupole-Orbitrap HESI. Full analytical data is given if the compound is novel.

2. Analysis of the reactions

2.1. HPLC analysis

HPLC analysis was conducted by Agilent 1260 Infinity Quaternary LC (Eclipse Plus 18C column 3.5 μm, 4.6 × 100 mm^2; UV detector, 265 nm) with a gradient of acetonitrile and 0.1% formic acid in Milli-Q® water at a flow rate of 1.0 mL/min.

HPLC gradient:

Time [min]	Acetonitrile [%]	Water [%]
0	40	60
8	60	40
12	95	5
15	95	5
30	40	60
35	40	60

Reactions monitored over time by HPLC analysis were sampled by removal of 20 μL aliquots of the reaction mixture at the beginning of the reaction (zero-time aliquot, no electrical current), then at every specified time point (*e.g.*, at 5 min, 10 min, 20 min, 30 min, 1 h, 2 h and 3 h) and at the end of the reaction. The HPLC sample was assembled by adding to the 20 μL reaction aliquot in a filter vial with 500 μL acetonitrile and 20 μL standard solution [0.1 M 4,4'-di-*tert*-butylbiphenyl (DTBB) in DMF], after which the sample was submitted for HPLC analysis. The concentrations of the starting material and formed products were calculated based on the precise volumes of the aliquot and dilution solvent and quantified towards the DTBB standard added. The molarity of the standard solution (0.1 M DTBB in DMF) was checked daily by HPLC. Negligible differences in analyte concentrations were observed when calculations were based on volumes or towards the DTBB standard. The retention times and calibrations were determined for all compounds investigated, either by using commercially available reference compounds or products isolated from the reaction mixture. The purity of the compounds was determined by NMR analysis, and then calibrated by HPLC under the same conditions as the reactions were analyzed. Three-fold serial dilutions of five with a dilution factor of 1/3 were used and plotted against the areas obtained for **1a** (R_t = 1.6 min), **2a** (R_t = 15.0 min), **3a** (R_t = 7.7 min), **4a** (R_t = 3.2 min), DTBB (R_t = 17.7 min) and H_2SiPh_2 (R_t = 14.3 min).

2.2. NMR analysis

The reported NMR yields were determined by ^{1}H-NMR (relaxation delay D1 = 5, number of scans 80 or 128; Bruker Avance 500 MHz spectrometer at 298 K) with internal standard 1,3,5-trimethoxybenzene (99.91% ± 0.28%). The standard was either added to the reaction mixture directly at end of the reaction after removing the volatiles or to the crude after extraction of the reaction mixture.

2.3. Average yield and standard deviation

The average yield and conversion were calculated as the arithmetic mean, $\bar{X}$, by using equation (1) for 2-3 parallel reactions. The n is the number of values and X_i is the annotation of different values.

$$\bar{X} = \frac{1}{n}\sum_{i=1}^{n} X_i \qquad \text{(Eqn. 1)}$$

The standard deviation, σ, was determined based on the calculated arithmetic mean by applying equation (2) for 2-3 parallel reactions.

$$\sigma = \sqrt{\frac{\sum_{i=1}^{n}(X_i-\bar{X})^2}{n-1}} \qquad \text{(Eqn. 2)}$$

The sample standard deviation where n is the number of values in the sample and X_i is the annotation of different values within the sample.

3. Electrochemical deoxygenative silylation of alcohols

3.1. Screening of general reaction conditions

Table S-1. Screening of reaction conditions

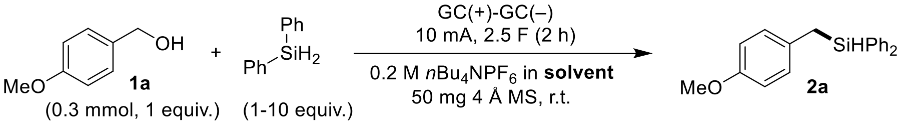

Entry	H_2SiPh_2 [equiv.]	Solvent (ratio)	Electrodes	Time [h]	Atm	4Å MS	Yield 2a [%][a]	Conv. 1a [%][a]
1	1	THF	GC(+)-GC(–)	2	N_2	yes	3	nd
2	3	THF	GC(+)-GC(–)	2	N_2	yes	30[b]	nd
3	5	THF	GC(+)-GC(–)	2	N_2	yes	38	nd
4	5	THF	C_{gr}(+)-C_{gr}(–)	2	N_2	yes	41	nd
5	5	THF	SS(+)-SS(–)	2	N_2	yes	-	-
6	5	DMF	GC(+)-GC(–)	2	N_2	yes	33	80
7	5	DMF	GC(+)-GC(–)	2	N_2	yes	33	80
8[c]	5	THF:DMF (1:1)	GC(+)-GC(–)	2	N_2	yes	74	85
9[c]	5	THF:DMF (1:1)	GC(+)-GC(–)	2	N_2	yes	70	91
10[c]	5	THF:DMF (1:1)	GC(+)-GC(–)	2	N_2	yes	70	84
11[c]	5	THF:DMF (1:1)	GC(+)-GC(–)	2	N_2	yes	74	nd
12	5	THF:DMF (1:1)	GC(+)-GC(–)	1	N_2	yes	51	84
13	5	THF:DMF (1:1)	GC(+)-GC(–)	4	N_2	yes	9	75
14[d]	5	THF:DMF (1:1)	GC(+)-GC(–)	2	air	-	67	81
15	8	THF:DMF (1:1)	GC(+)-GC(–)	2	N_2	yes	62	84
16[d]	8	THF:DMF (1:1)	GC(+)-GC(–)	2	air	-	61	99
17	5	THF:DMF (5:1)	GC(+)-GC(–)	2	air		76	87
18[d,e]	5	THF:DMF (5:1)	GC(+)-GC(–)	3	air	-	88	95
19[d,e]	5	THF:DMF (5:1)	GC(+)-GC(–)	3	air	-	92	95
20[d,e]	5	THF:DMF (5:1)	GC(+)-GC(–)	3	air	-	91	nd
21	8	THF:DMF (5:1)	GC(+)-GC(–)	2	N_2	yes	67	84
22	8	THF:DMF (5:1)	GC(+)-GC(–)	2	N_2	-	66	90
23[d,f]	10	THF:DMF (4:1)	GC(+)-GC(–)	2	air	-	65	nd
24[d,f]	10	THF:DMF (4:1)	GC(+)-GC(–)	2	air	-	68	nd
25[d]	10	THF:DMF (4:1)	GC(+)-GC(–)	3	air	-	91	>99
26[d,j]	5	THF:DMF (5:1)	GC(+)-GC(–)	2	air	-	60	nd

[a] ^{1}H-NMR analysis with 1,3,5-trimethoxybenzene as internal standard; [b] Isolated yield; [c] Parallel reactions; [d] in air, no molecular sieves; [e] Parallel reactions; [f] Parallel reactions; [j] H_2O (1 equiv.) added with the reactants. MS – molecular sieves; SS – stainless steel; C_{gr} – graphite; nd – not determined.

3.2 Screening of co-solvents

Table S-2. Screening of co-solvents

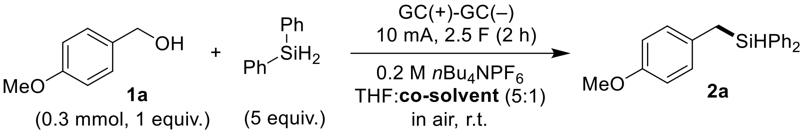

Entry	Co-solvent	**Yield 2a** [%][a]	**Conv. 1a** [%][a]
1	DMF	76	87
2	DMC	18	nd
3	sulfolane	65	nd
4	DMA	26	nd
5[b]	EtOAc	37	nd
6[b]	anisole	12	nd
7	DMPU	73	nd
8[c]	2-Me THF : DMPU	71	nd
9[b]	AcCN	28	nd

[a] ^{1}H-NMR analysis with 1,3,5-trimethoxybenzene as internal standard; [b] Visible layer formed on anode; [c] Solvent mixture 2Me THF:DMPU (5:1) was used instead of THF:DMF (5:1). DMC – dimethyl carbonate, DMA – dimethylacetamide, EtOAc – ethyl acetate, DMPU – *N,N'*-dimethylpropyleneurea, 2-Me THF – 2-methyltetrahydrofuran, AcCN – acetonitrile, nd – not determined.

3.3 Assessment of supporting electrolytes

Table S-3. Screening of supporting electrolyte salts

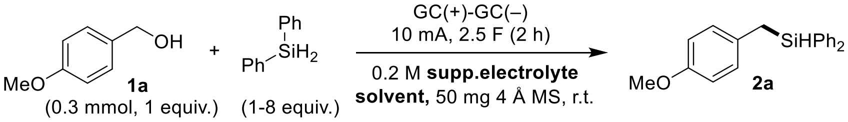

Entry	**H_2SiPh_2** [equiv.]	Solvent (ratio)	**Supp. Electrolyte** [M]	atm	4Å MS	**Yield 2a** [%][a]	**Conversion 1a** [%][a]
1	1	THF:DMF (1:1)	0.2 M KPF_6	N_2	yes	<1	nd
2	3	THF:DMF (1:1)	0.2 M KPF_6	N_2	yes	48	80
3	5	THF:DMF (1:1)	0.2 M KPF_6	N_2	yes	52	87
4[b]	8	THF:DMF (2:1)	0.2 M KPF_6	air	-	55	79
5	8	THF:DMF (2:1)	0.2 M KPF_6	N_2	yes	61	90
6[b]	5	THF:DMF (5:1)	0.1 M KPF_6	air	-	13	nd
7[b]	5	THF:DMF (5:1)	0.2 M $n$$Bu_4NPF_6$	air	-	76	87
8[b,c]	5	THF:DMF (5:1)	0.1 M $n$$Bu_4NPF_6$	air	-	66	nd
9[b,c]	5	THF:DMF (5:1)	0.1 M $n$$Bu_4NPF_6$	air	-	70	nd
10[b,c]	5	THF:DMF (5:1)	0.1 M $n$$Bu_4NPF_6$	air	-	66	nd
11[b]	5	THF:DMF (5:1)	0.05 M $n$$Bu_4NPF_6$	air	-	60	nd

[a] ^{1}H-NMR analysis with 1,3,5-trimethoxybenzene as internal standard; [b] in air, no molecular sieves; [c] Parallel reactions. MS – molecular sieves, nd – not detected.

3.4 Reproducibility

Reproducibility between reactions was ensured by using oven-dried glassware and stir bars, as well as by a rigorous cleaning procedure of the electrodes (see general information, section 1). All reagents and solvents were measured out or dispensed prior to the reaction (for cleaning methods, see general information). Parallel reactions were run using the same brand of power supply. Keeping to these guidelines, we observed reproducible results throughout optimization of the reaction conditions (Table S-1).

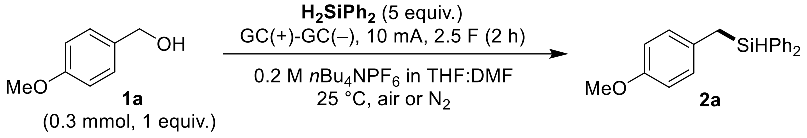

Table S-4. Parallel electrochemical silylation reactions of *p*-phenylbenzyl alcohol (**1a**).[a]

N°	**Solvent** (ratio)	**Conditions**	**Time** [h]	**Yield 2a [%]**[b]	**Average 2a yield ± σ [%]**[c]	**Conv. 1a [%]**[b]	**Average 1a conv. ± σ [%]**[c]
1				74		85	
2	THF:DMF (1:1)	50 mg 4Å MS, N_2	2	70	73 ± 2	90	88 ± 3
3				74		90	
4	THF:DMF (5:1)	air, no MS	3	88	90 ± 3	95	95
5				92		95	

[a]A mixture of *p*-methoxy benzyl alcohol **(1a)** (0.3 mmol, 1 equiv.), diphenylsilane (5 equiv.) and supporting electrolyte nBu$_4$NPF$_6$ (2 equiv.) was stirred for 2 h (2.5 F) or 3 h (3.7 F) at 25 °C in solvent mixture THF:DMF (4:1, 3 mL) sealed under air or nitrogen, using a glassy carbon cathode and glassy carbon anode at a constant current of 10 mA. [b]Yield for **2a** and conversion for **1a** from one reaction measured by ^{1}H-NMR analysis. [c]Average yield and conversion with standard deviation calculated from four parallel reactions. MS – molecular sieves.

3.5 Sampling over time, assessed by HPLC analysis

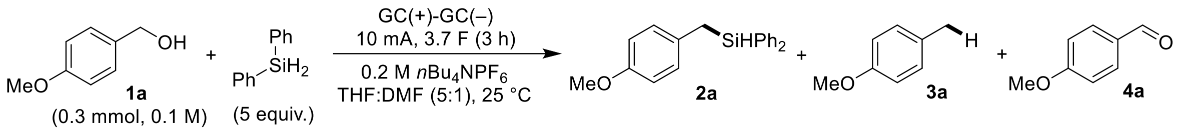

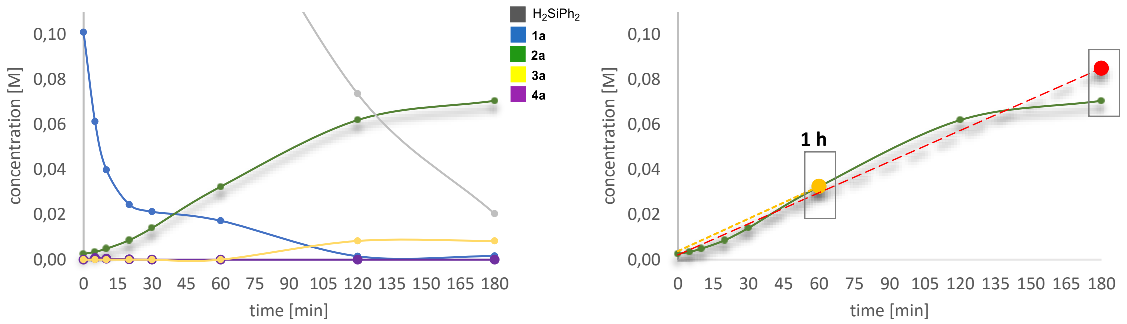

Figure S-1. *To left:* Electroreductive silylation of benzyl alcohols assessed by HPLC analysis, aliquots removed at specified time points. Consumption of benzyl alcohol **1a** (blue), diphenyl silane (gray) and formation of silylation product **2a** (green), **3a** (yellow) and **4a** (purple) are shown. *To right:* The yield of **2a** in the sampled reaction (see to left; green) compared to reactions run without sampling (no aliquots removed) for 1 h (orange) and 3 h (red). Analyzed by HPLC.

To assess to which extent the electroreductive silylation of benzyl alcohols is affected by sampling over time, a set of three parallel reactions was run. Firstly, a 3 h reaction that was sampled and analysed by HPLC by removing aliquots of 20 µL at specific time points (0-time, 5, 10, 20, 30 min and 1, 2, 3 h) and submitted to HPLC analysis (Figure S-1, left). The second and third reactions were run 1 h and 3 h, respectively, with only 0-time and end-time samples removed. All three parallel reactions were additionally assessed by quantitative ^{1}H-NMR analysis at the end of the reaction. Shown on left in Figure S-1, after 1 h, the sampled reaction (green) had 32% yield for **2a**, whereas the non-sampled 1 h reaction (orange) gave **2a** in 31% (37% NMR yield). After 3 h, the sampled reaction (green) gave **2a** in 70% yield (66% NMR yield) and the non-sampled reaction (red) gave **2a** in 79% (82% NMR yield). In summary, although the electroreductive silylation was increasingly affected by sampling with accumulating time and sampling, the effect was negligible enough not to disturb the general trend of the reaction. Additionally, the yields assessed by HPLC and ^{1}H-NMR analysis corroborated each other well.

3.6 Reactivity of alcohols with electron-withdrawing groups

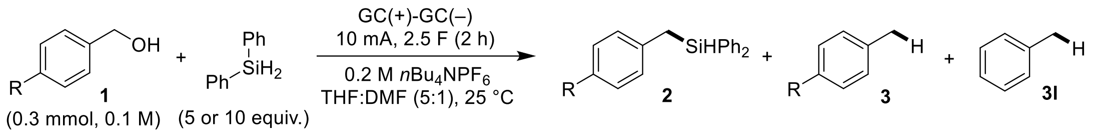

Table S-5. Silylation of benzylic alcohols with electron-withdrawing groups in *para*-position

Entry	R	H_2SiPh_2 (equiv.)	Yield 2 [%][a]	Yield 3 [%][a]	Yield toluene (3l) [%][a]	Conversion 1 [%][a]
1 [c,e]	Br	5	traces	-	80	>99
2 [c,e]	Cl	5	traces	-	39	>99
3 [b,c,d,e]	F	5	44	-	54	>99
4	CO_2Me	10	-	20	-	>99
5	CN	10	-	37	-	>99

[a] Analyzed by HPLC. [b] ^{1}H-NMR analysis with 1,3,5-trimethoxybenzene as internal standard; [c] graphite electrodes were used for both anode and cathode; [d] No sampling over time; [e] 0.1 M nBu$_4$NPF$_6$; nd – not determined.

3.7 Diphenylsilane concentration

Electroreductive silylation of **1a** in the presence of 2 equiv. diphenylsilane gave 23% silylated product **2a** and 42% alkane **3a**. Increasing diphenylsilane to 5 equiv. increased the **2a** yield to 70% and lowered the yield of **3a** to 8% at the end of the reaction. Using the excess of 10 equiv. of diphenyl silane resulted in a high yield of 83% for **2a** and no observable **3a**.

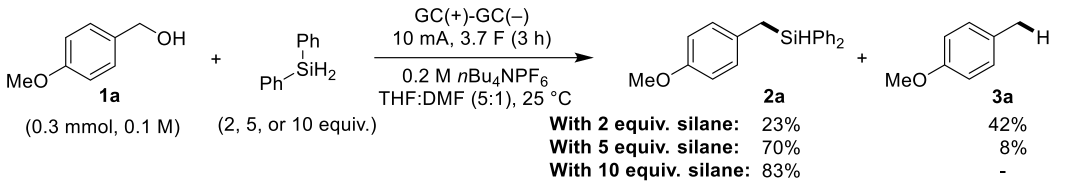

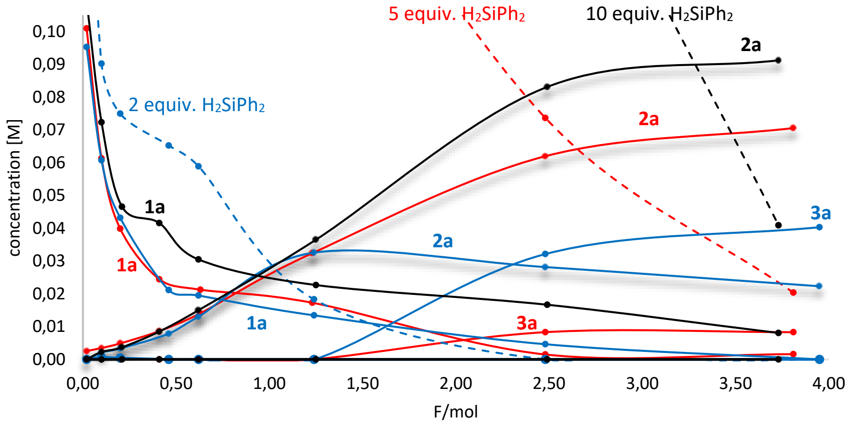

Figure S-2. Comparison of three reactions run until 3.7 F/mol (3 h) with 2 equiv. H_2SiPh_2 (blue), 5 equiv. H_2SiPh_2 (red) and 10 equiv. H_2SiPh_2 (black). Consumption of benzyl alcohol **1a**, diphenyl silane and formation of silylation product **2a**, as well as **3a** are shown. All reactions were sampled over time and analyzed by HPLC.

3.8 Alcohol concentration

Comparison of three reactions, where either 0.1 M (Figure S-3, green), 0.05 M (black) or 0.02 M (blue) benzyl alcohol **1a** were run with 5 equiv. diphenyl silane, respectively, showed that after *ca* 4 F the yields for **2a** are comparable for **1a** starting concentrations 0.1 M and 0.05 M and do not seem to depend on the concentrations of 1a and diphenyl silane. Diminished yields were achieved when **1a** concentration was as low as 0.02 M.

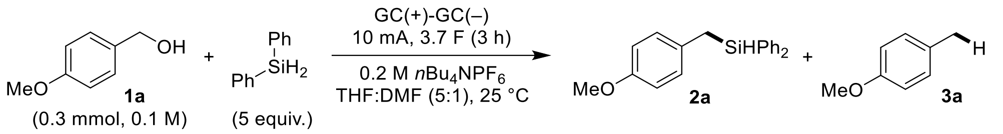

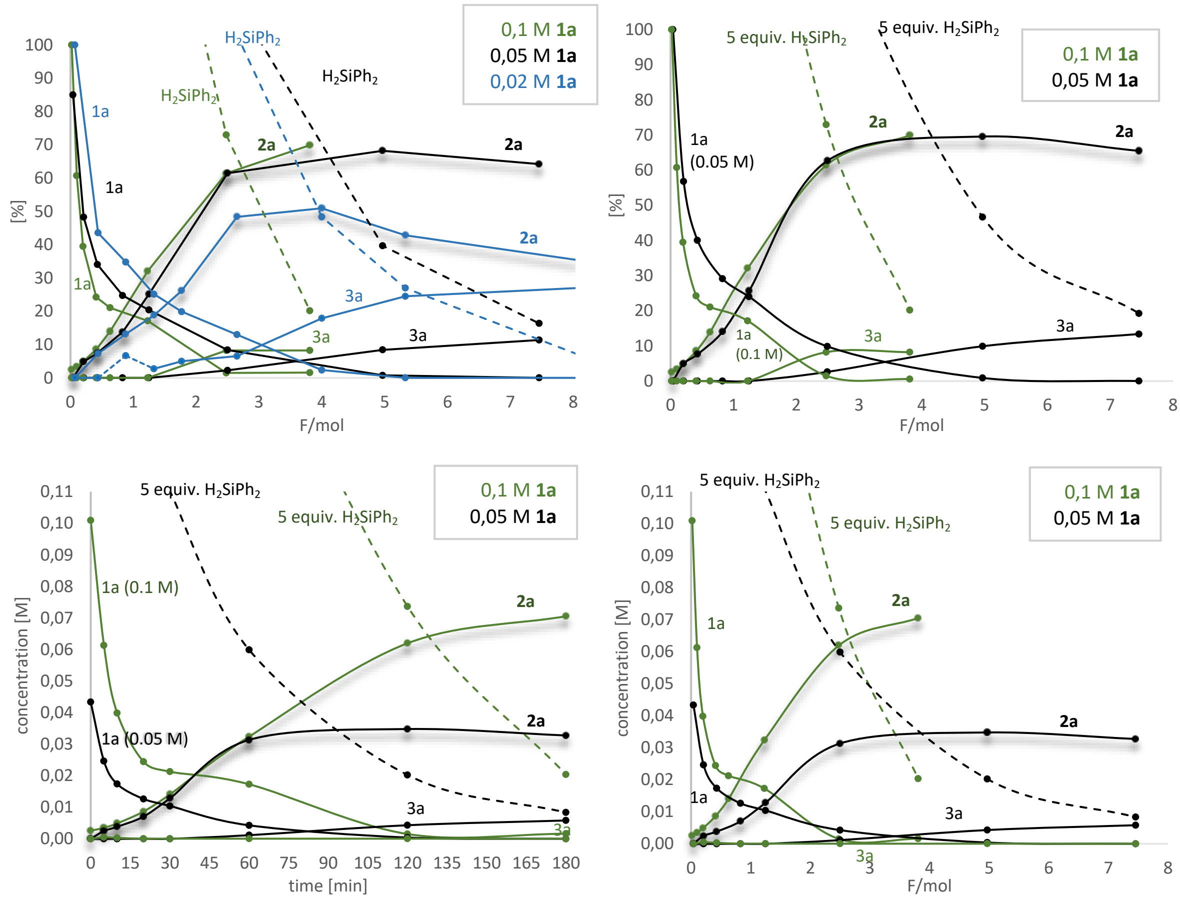

Figure S-3. Comparison of three parallel reactions with different alcohol **1a** concentrations. **Green:** benzyl alcohol **1a** (0.30 mmol, 0.1 M, 1 equiv.) and H_2SiPh_2 (1.5 mmol, 0.5 M, 5 equiv.) with 0.2 M Bu_4NPF_6 in THF:DMF (5:1, 3 mL). Sampled at 0-time, 5 min (0.10 F/mol), 10 min (0.20 F/mol), 20 min (0.41 F/mol), 30 min (0.63 F/mol), 1 h (1.23 F/mol), 2 h (2.48 F/mol), 3 h (3.81 F/mol). **Black:** benzyl alcohol **1a** (0.15 mmol, 0.05 M, 1 equiv.) and H_2SiPh_2 (0.75 mmol, 0.25 M, 5 equiv.) with 0.2 M Bu_4NPF_6 in THF:DMF (5:1, 3 mL). Sampled at 0-time, 5 min (0.21 F/mol), 10 min (0.43 F/mol), 20 min (0.84 F/mol), 30 min (1.25 F/mol), 1 h (2.50 F/mol), 2 h (4.97 F/mol), 3 h (7.46 F/mol). **Blue:** benzyl alcohol **1a** (0.07 mmol, 0.02 M, 1 equiv.) and H_2SiPh_2 (0.37 mmol, 0.12 M, 5 equiv.) with 0.2 M Bu_4NPF_6 in THF:DMF (5:1, 3 mL). Sampled at 0-time, 5 min (0.21 F/mol), 10 min (0.43 F/mol), 20 min (0.84 F/mol), 30 min (1.25 F/mol), 1 h (2.50 F/mol), 2 h (4.97 F/mol), 3 h (7.46 F/mol).

3.9 Zinc as sacrificial anode

The electroreductive silylation was tested with zinc as sacrificial anode (Table S-6). Two parallel reactions sampled over time (entries 2 and 3) gave inconsistent results, although following the trend of rapid consumption of **1a** and diphenylsilane and low **2a** yields (see also Figure S-4, left). HPLC yield for **2a** at 3 h (18%) was also confirmed by ^{1}H-NMR analysis (17%, see entry 2). Reactions not sampled and analyzed only by ^{1}H-NMR at the end of the reaction corroborated low **2a** yields (entries 1 and 4). Increasing H_2SiPh_2 from 5 equiv. to 10 equiv. resulted in a higher yield for **2a** up till 30 min into the reaction, followed by degradation of the formed silylation product to only trace amount at 3 h (Table S-6, entry 5 and Figure S-4, right).

Table S-6. Zinc as sacrificial anode under silylation conditions.

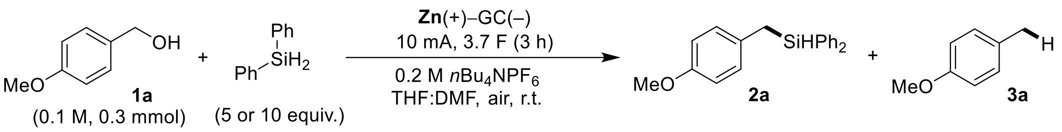

entry	H_2SiPh_2 [equiv.]	Solvent (ratio)	Yield 2a [%][a]	Yield 3a [%][a]	Conversion 1a [%][a]
1 [b,c,d,e]	5	DMF	23 [b]	nd	nd
2 [f]	5	THF:DMF (5:1)	18 (17) [b]	13	92
3 [f]	5	THF:DMF (5:1)	1	43	83
4 [b,c,d]	5	THF:DMF (5:1)	24 [b]	nd	nd
5	10	THF:DMF (4:1)	traces	traces	43

[a] Analyzed by HPLC. [b] ^{1}H-NMR analysis with 1,3,5-trimethoxybenzene as internal standard; [c] under N_2, 50 mg 4Å molecular sieves; [d] No sampling over time; [e] Reaction time 2 h (2.5 F); [f] Parallel reactions; nd – not determined.

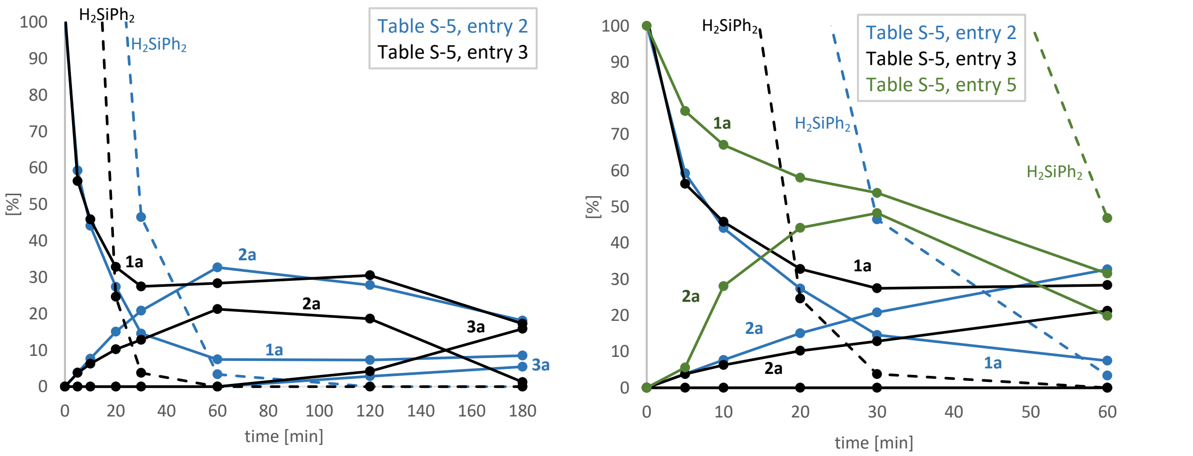

Figure S-4. *Left:* Comparison of two parallel reactions with 5 equiv. H_2SiPh_2 over 3 h (Table S-5, entry 2 – blue; Table S-5, entry 3 – black). *Right:* Comparison of the reaction with 10 equiv. H_2SiPh_2 (Table S-5, entry 5 – green) to the two parallel reactions with 5 equiv. H_2SiPh_2 shown over 1 h (Table S-5, entry 2 – blue; Table S-5, entry 3 – green). Yields determined by HPLC analysis.

3.10 Comparison of reactions under constant current 5 mA and 10 mA or no current

The electrochemical silylation of **1a** was assessed at constant current 5 mA and 10 mA (Figure S-5). The reactions were sampled and monitored by HPLC analysis, after termination of the reaction (6 h at 5 mA and 3 h at 10 mA) also by ^{1}H-NMR analysis (1,3,5-trimethoxybenzene as internal standard). After *ca* 4 F the yields for **2a** are comparable and do not seem to depend on the current density used. The reaction mixture was also assessed in the absence of electrical current (Table S-7). No reaction occurred when current was omitted, the starting materials remained unreacted.

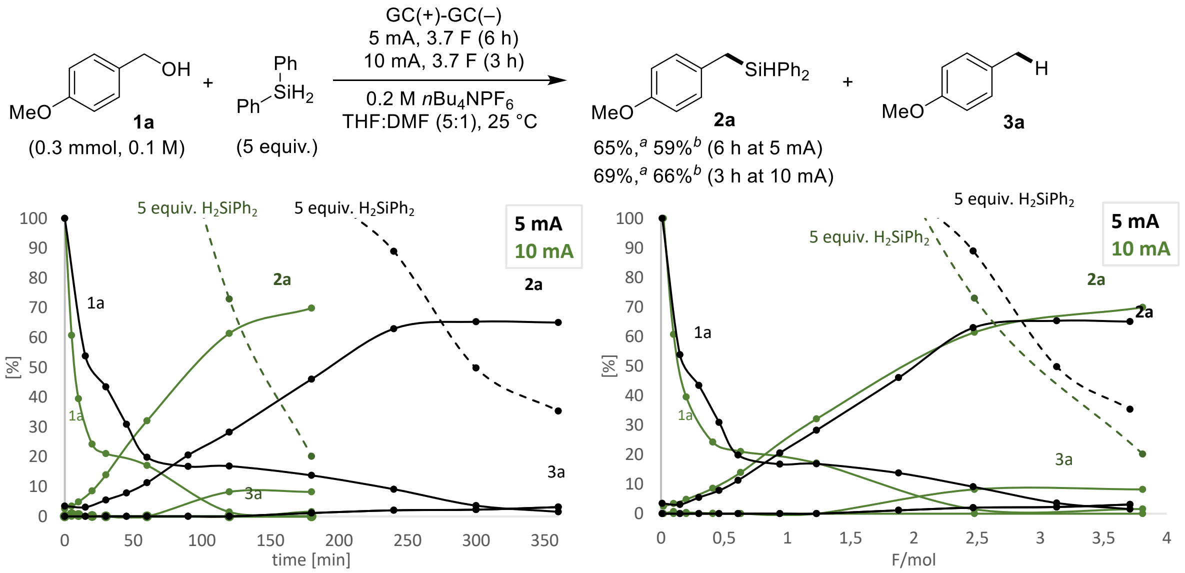

Figure S-5. Electroreductive silylation of **1a** using constant current 5 mA (black) or 10 mA (green). Consumption of benzyl alcohols **1a**, diphenyl silane and formation of silylation product **2a** and alkane **3a** are shown**.** All reactions were sampled over time and analyzed by HPLC.

Table S-7. Benzyl alcohol **1a** under standard silylation conditions without electrical current.

**Entry**	**H_2SiPh_2** [equiv.]	**Solvent** (ratio)	**Time** [h]	**Atm**	**4Å MS**	**Yield 2a** [%]	**Conv. 1a** [%]
1[a]	5	THF:DMF (1:1)	2	N_2	yes	0	0
2[b,c]	10	THF:DMF (4:1)	3	air	-	0	0

[a] NMR analysis; [b] HPLC analysis; [c] No change after 24 h, HPLC analysis.

When 4-methoxybenzaldehyde was stirred under silylation conditions without electrical current, no reduction of aldehyde **3a** to corresponding alcohol **1a** was observed (Figure S-6).

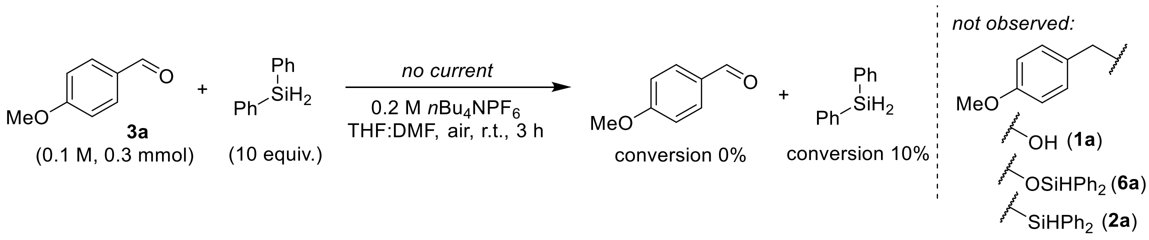

Figure S-6. 4-Methoxybenzaldehyde under silylation conditions without electrical current. Yields determined by HPLC.

3.11 Other hydrosilanes

Other hydrosilanes, like methylphenylsilane and phenylsilane, were tested under electrochemical silylation conditions with *p*-MeO benzyl alcohol (**1a**) but did not result in the desired products in significant amounts (Figure S-7).

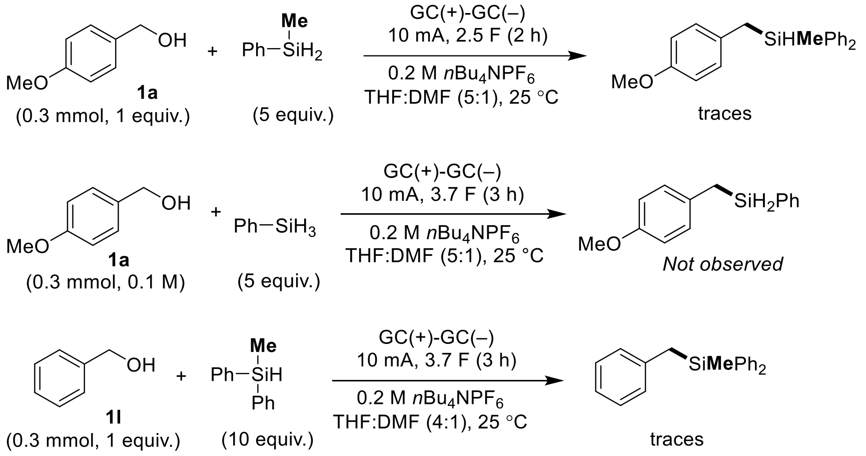

Figure S-7. Alcohol **1a** under electrochemical silylation conditions with methylphenylsilane (top), phenylsilane (middle) and methyldiphenylsilane (bottom).

3.12 Scale-up silylation of 1a

Silylation of the benchmark substrate **1a** was shown to preform equally well both at 0.3 mmol and 1.5 mmol scale giving **2a** in excellent yield and good faradaic efficiency (FE) (Table S-8, entries 1 and 2). For further scale-up some modifications were necessary, *e.g.*, increasing the amount of DMF used and decreasing the charges passed, giving **2a** in lower yield but in comparable FE (entries 3 and 4).

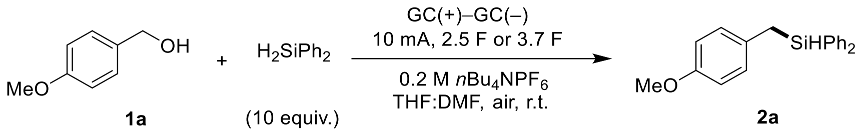

Table S-8. *p*-MeO Benzyl alcohol (**1a**) silylation at 0.3, 1.5 and 3.5 mmol scale.

**entry**	**1a** (amount used)	**Solvent** (ratio)	***F*** (time)	**2a yield** [%][a]	**FE** [%]
1	0.3 mmol (42 mg), 0.1 M	THF:DMF (4:1)	3.7 F (3 h)	91	48.6
2	1.5 mmol (211 mg), 0.1 M	THF:DMF (4:1)	3.7 F (15.5 h)	89	47.9
3 [b]	3.5 mmol (500 mg), 0.2 M	THF:DMF (4:1)	3.7 F (36 h)	nd	nd
4 [c]	3.5 mmol (500 mg), 0.2 M	THF:DMF (1:1)	2.5 F (24 h)	59	46.9

[a] ^{1}H-NMR analysis with 1,3,5-trimethoxybenzene as internal standard; [b] reaction was shut down at undetermined time due to passivation of electrodes; [c] 0.05 M of nBu$_4$NPF$_6$ in THF:DMF (1:1) was used; FE – faradaic efficiency; nd – not determined.

The faradaic efficiency was calculated using equation 1 where *N* is the number of moles of product formed, *n* is the number of electrons required for 1 mol of product formation, and *F* is the Faraday constant (96485 C/mol).[49]

$$FE\ [\%] = \frac{N \times n \times F}{Q_{total}} \times 100 \qquad \text{(Eqn. 3)}$$

4. General reaction procedures

4.1. General Procedure for electrochemical deoxygenative silylation

Oven-dried 5 mL IKA vial equipped with a magnetic stir bar was charged with alcohol (0.3 mmol, 1 equiv.) and Bu_4NPF_6 (0.6 mmol, 2 equiv.). Diphenylsilane (1.5 mmol, 5 equiv.) was added with a 500 µL Hamilton syringe. The vial was sealed with an IKA cap equipped with glassy carbon (or graphite) as both anode and cathode and flushed with nitrogen flow when indicated. Anhydrous THF (2.5 mL) and anhydrous DMF (0.5 mL) were added and stirring (800 rpm) was turned on. Constant current electrolysis was performed at 10 mA. After electrolysis, the reaction mixture was concentrated *in vacuo*, the residue was diluted with ethyl acetate and washed with water and brine. After drying the collected organic phases on $MgSO_4$, the solution was filtered and concentrated *in vacuo*. If the yield was assessed by quantitative ^{1}H-NMR analysis, the NMR standard 1,3,5-trimethoxybenzene was added to the crude. Isolation was performed by flash column chromatography on silica gel, further purification by preparative TLC.

4.2. General Procedure for electrochemical deoxygenative carboxylation

Oven-dried 5 mL IKA vial equipped with a magnetic stir bar was charged with alcohol (0.3 mmol, 1 equiv.) and Bu_4NPF_6 (0.6 mmol, 2 equiv.). The vial was sealed with an IKA cap equipped with glassy carbon as both anode and cathode, and flushed with carbon dioxide for 10-20 min. Anhydrous THF (2.5 mL) and anhydrous DMF (0.5 mL) were added and stirring (800 rpm) was turned on. Constant current electrolysis was performed at 10 mA under carbon dioxide flow to the overhead space. After electrolysis, the reaction mixture was concentrated

in vacuo, and following an acid-base workup, the obtained product was assessed by NMR analysis.

5. Synthetic details and analytical data for products 2a-2s

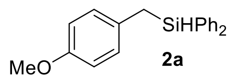

2a

(4-Methoxybenzyl)diphenylsilane (2a) was synthesized from 4-methoxy benzylalcohol (**1a**) according to general procedure for electrochemical deoxygenative silylation and isolated by flash column chromatography on neutral silica gel (100% pentane) in 95% yield (white solid). **^{1}H NMR** (500 MHz, $CDCl_3$) δ 7.54 – 74.9 (m, 4H), 7.42–7.34 (m, 6H), 6.94 (m, 2H), 6.74 (m, 2H), 4.82 (t, J = 3.8 Hz, 1H), 3.77 (s, 3H), 2.64 (d, J = 3.8 Hz, 2H). **^{13}C NMR** (126 MHz, $CDCl_3$) δ 157.09, 135.41, 134.49, 133.87, 130.46, 129.82, 129.69, 128.08, 113.94, 55.35, 21.10. HRMS(HESI) for $C_{20}H_{24}NOSi$ $(M+NH_4)^+$ calculated 322.1627 measured 322.1622.
Yields of **2a** were assessed by quantitative ^{1}H-NMR analysis from reaction mixtures. After termination of the silylation reaction (see general procedure for electrochemical deoxygenative silylation) and extraction workup, the volatiles were removed *in vacuo*, and 1,3,5-trimethoxybenzene (TMB) was weighed to the crude. NMR analysis was performed (see section 2.2 NMR analysis), methylene signal 2.64 (d, J = 3.8 Hz, 2H) was used for quantification.
2a was also assessed by HPLC analysis in reactions monitored over time via sampling. HPLC analysis was done on Agilent 1260 Infinity Quaternary LC (Eclipse Plus 18C column [3.5 µm, 4.6×100 mm^2], UV detector 265 nm, with a gradient of acetonitrile and 0.1% formic acid in Milli-Q® water at a flow rate of 1.0 mL/min, using 4,4'-di-*tert*-butylbiphenyl [DTBB] as internal standard, 5 µL injection volume). Retention times for **2a** are 15.0 min and for DTBB 17.8 min, respectively. All the HPLC yields reported are obtained by comparing against HPLC calibrations on commercially available reference compounds or products isolated from the reaction mixture.

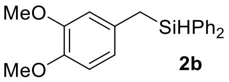

2b

(3,4-Dimethoxybenzyl)diphenylsilane (2b) was synthesized from 3,4-dimethoxybenzyl alcohol (**1b**) according to general procedure for electrochemical deoxygenative silylation and isolated by flash column chromatography on neutral silica gel (20% CH_2Cl_2 in pentane) in 79% (white solid). **^{1}H NMR** (500 MHz, $CDCl_3$) δ 7.51 (dt, J = 6.6, 1.5 Hz, 4H), 7.43 – 7.38 (m, 2H), 7.35 (dd, J = 7.8, 6.5 Hz, 4H), 6.70 (d, J = 8.1 Hz, 1H), 6.58 (dd, J = 8.2, 2.0 Hz, 1H), 6.38 (d, J = 2.0 Hz, 1H), 4.93 (t, J = 3.6 Hz, 1H), 3.83 (s, 3H), 3.60 (s, 3H), 2.63 (d, J = 3.6 Hz, 2H). **^{13}C NMR** (126 MHz, $CDCl_3$) δ 148.64, 146.41, 135.46, 133.82, 129.85, 128.08, 120.50, 112.32, 111.44, 55.99, 55.57, 21.73. HRMS(HESI) for $C_{21}H_{23}O_2Si$ $(M+H)^+$ calculated 335.1467, measured 335.1464.
Yields of **2b** were assessed by quantitative ^{1}H-NMR analysis from reaction mixtures. After termination of the silylation reaction (see general procedure for electrochemical deoxygenative silylation) and extraction workup, the volatiles were removed *in vacuo*, and 1,3,5-trimethoxybenzene (TMB) was weighed to the crude. NMR analysis was performed (see section 2.2 NMR analysis), methylene signal 2.63 (d, J = 3.6 Hz, 2H) was used for quantification.

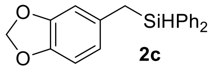

2c

(Benzo[d][1,3]dioxol-5-ylmethyl)diphenylsilane (2c) was synthesized from (**1c**) according to general procedure for electrochemical deoxygenative silylation and isolated by flash column chromatography on neutral silica gel (2% CH_2Cl_2 in pentane) in 86% (white solid), further isolation by preparative TLC (colourless oil). **^{1}H NMR** (500 MHz, $CDCl_3$) δ 7.52 (dd, J = 7.6, 3.5 Hz, 4H), 7.48 – 7.33 (m, 6H), 6.72 – 6.41 (m, 4H), 5.90 (d, J = 3.4 Hz, 2H), 4.94 (d, J = 3.9 Hz, 1H), 2.62 (d, J = 3.7 Hz, 2H). **^{13}C NMR** (126 MHz, CDCl3) δ 147.46, 144.77, 135.24, 134.36, 133.55, 132.14, 129.76, 127.98, 127.76, 127.59, 121.26, 109.27, 108.17, 100.62, 29.71, 21.84.

Yields of **2c** were assessed by quantitative ^{1}H-NMR analysis from reaction mixtures. After termination of the silylation reaction (see general procedure for electrochemical deoxygenative silylation) and extraction workup, the volatiles were removed *in vacuo*, and 5.2 mg 1,3,5-trimethoxybenzene (TMB) was weighed to the crude. NMR analysis was performed (see section 2.2 NMR analysis), methylene signal 2.57 (d, J = 3.7 Hz, 2H) was used for quantification.

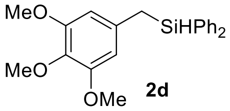

2d

Diphenyl(3,4,5-trimethoxybenzyl)silane (2d) was synthesized from 3,4,5-trimethoxybenzene (**1d**) according to general procedure for electrochemical deoxygenative silylation and isolated by flash column chromatography on silica gel (5% EtOAc in petroleum ether). **^{1}H NMR** (400 MHz, $CDCl_3$) δ 7.51 (d, J = 6.9 Hz, 4H), 7.37 (dt, J = 14.4, 7.3 Hz, 6H), 6.11 (d, J = 4.0 Hz, 2H), 4.92 (q, J = 3.8 Hz, 1H), 3.79 (s, 3H), 3.63 (s, 6H), 2.61 (d, J = 3.6 Hz, 2H). **^{13}C NMR** (101 MHz, $CDCl_3$) δ 152.91, 135.45, 135.15, 134.21, 133.60, 129.91, 128.06, 105.64, 61.00, 55.84, 22.84. HRMS(HESI) for $C_{22}H_{25}O_3Si$ $(M+H)^+$ calculated 365.1573 measured 365.1575.

Yields of **2d** were assessed by quantitative ^{1}H-NMR analysis from reaction mixtures. After termination of the silylation reaction (see general procedure for electrochemical deoxygenative silylation) and extraction workup, the volatiles were removed *in vacuo*, and 1,3,5-trimethoxybenzene (TMB) was weighed to the crude. NMR analysis was performed (see section 2.2 NMR analysis), methylene signal 2.63 (d, J = 3.6 Hz, 2H) was used for quantification.

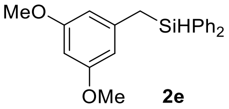

2e

(3,5-Dimethoxybenzyl)diphenylsilane (2e) was synthesized from 3,5-dimethoxybenzyl alcohol (**1e**) according to general procedure for electrochemical deoxygenative silylation and quantified by ^{1}H qNMR, isolation by preparative TLC. **^{1}H NMR** (400 MHz, CDCl3) δ 7.55 – 7.31 (m, 10H), 6.19 (d, J = 2.3 Hz, 1H), 6.13 (d, J = 2.2 Hz, 2H), 4.94 (t, J = 3.7 Hz, 1H), 3.62 (s, 6H), 2.63 (d, J = 3.7 Hz, 2H). **^{13}C NMR** (101 MHz, CDCl3) δ 160.66, 141.02, 135.44, 133.71, 129.90, 128.09, 106.72, 97.37, 77.36, 55.21, 23.01. HRMS(HESI) for $C_{21}H_{23}O_2Si$ $(M+H)^+$ calculated 335.1467, measured 335.1469.

Yield (25%) of **2e** was assessed by quantitative ^{1}H-NMR analysis from the reaction mixture. After termination of the silylation reaction (see general procedure for electrochemical deoxygenative silylation) and extraction workup, the volatiles were removed *in vacuo*, and 18.4 mg 1,3,5-trimethoxybenzene (TMB) was weighed to the crude. NMR analysis was performed

(see section 2.2 NMR analysis), methylene signal 2.63 (d, J = 3.7 Hz, 2H) was used for quantification.

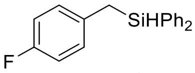

2f

(4-Fluorobenzyl)diphenylsilane (2f) was synthesized from 4-fluorobenzyl alcohol (**1f**) according to general procedure for electrochemical deoxygenative silylation and purified by flash column chromatography on neutral silica gel (5% CH_2Cl_2 in pentane) as a clear oil. **^{1}H NMR** (500 MHz, $CDCl_3$) δ 7.50–7.46 (m, 4H), 7.43–7.38 (m, 2H), 7.37–7.32 (m, 4H), 6.92 (dd, J = 8.5, 5.5 Hz, 2H), 6.88–6.80 (m, 2H), 2.64 (d, J = 3.8 Hz, 2H). **^{13}C NMR** (101 MHz, $CDCl_3$) δ 161.68, 159.75, 135.37, 134.21, 134.18, 133.42, 130.01, 129.98, 129.94, 128.15, 115.24, 115.07, 21.54. **^{19}F NMR** (377 MHz, $CDCl_3$) δ -119.17 (tt, J = 9.3, 5.4 Hz).
Yields of **2f** were assessed by quantitative ^{1}H-NMR analysis from reaction mixtures. After termination of the silylation reaction (see general procedure for electrochemical deoxygenative silylation) and extraction workup, the volatiles were removed *in vacuo*, and 1,3,5-trimethoxybenzene (TMB) was weighed to the crude. NMR analysis was performed (see section 2.2 NMR analysis), methylene signal 2.64 (d, J = 3.8 Hz, 2H) was used for quantification. For characterization purposes **2f** was oxidized to a corresponding silanol (see section 7, derivatization of hydrosilanes 2).

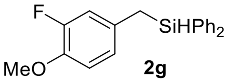

2g

(3-Fluoro-4-methoxybenzyl)diphenylsilane (2g) was synthesized from 3-fluoro-4-methoxybenzyl alcohol (**1g**) according to general procedure for electrochemical deoxygenative silylation and purified by flash column chromatography on neutral silica gel (100% pentane) as a colourless oil. **^{1}H NMR** (400 MHz, $CDCl_3$) δ 7.54 (d, J = 7.1 Hz, 4H), 7.43 (dd, J = 8.5, 6.1 Hz, 2H), 7.37 (t, J = 7.3 Hz, 5H), 6.80–6.66 (m, 3H), 3.82 (s, 3H), 2.62 (s, 2H). **^{13}C NMR** (101 MHz, $CDCl_3$) δ 153.3, 151.4, 135.2, 134.5, 134.4, 130.3, 128.1, 124.4, 124.3, 116.8, 116.6, 113.7, 113.7, 56.5, 24.2.
Yields of **2g** were assessed by quantitative ^{1}H-NMR analysis from reaction mixtures. After termination of the silylation reaction (see general procedure for electrochemical deoxygenative silylation) and extraction workup, the volatiles were removed *in vacuo*, and 1,3,5-trimethoxybenzene (TMB) was weighed to the crude. NMR analysis was performed (see section 2.2 NMR analysis), methylene signal 2.62 (d, J = 3.8 Hz, 2H) was used for quantification. For characterization purposes **2g** was oxidized to a corresponding silanol (see Section S7, derivatization of hydrosilanes).

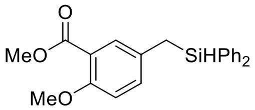

2h

Methyl 5-[(diphenylsilyl)methyl]-2-methoxybenzoate (2h) was synthesized from methyl 5-(hydroxymethyl)-2-methoxybenzoate according to general procedure for electrochemical deoxygenative silylation after 2 h with 10 equiv. of diphenylsilane in 46% (NMR yield) and isolated by flash column chromatography on silica gel (8% EtOAc in petroleum ether) in 51% as colourless oil with minor impurities. **^{1}H NMR** (500 MHz, $CDCl_3$) δ 7.53–7.47 (m, 4H), 7.45 (d, J = 2.6 Hz, 1H), 7.43 – 7.39 (m, 2H), 7.35 (m, 4H), 7.05 (dd, J = 8.5, 2.6 Hz, 1H), 6.77 (d, J = 8.5 Hz, 1H), 4.94 (t, J = 3.8 Hz, 1H), 3.84 (s, 6H), 2.63 (d, J = 3.8 Hz, 2H). **^{13}C NMR** (126 MHz, $CDCl_3$) δ 166.8, 156.7, 135.38, 134.43, 133.7, 133.4, 131.8, 130.5, 130.2, 129.9, 128.3,

128.2, 128.1, 127.99, 127.96, 127.8, 119.8, 112.3, 56.2, 51.99, 21.0. HRMS(HESI) for $C_{22}H_{23}O_3Si$ $(M+H)^+$ calculated 363.1416, measured 363.1402.
Yield of **2h** was assessed by quantitative ^{1}H-NMR analysis from reaction mixture. After termination of the silylation reaction (see general procedure for electrochemical deoxygenative silylation) and extraction, 1,3,5-trimethoxybenzene (6.9 mg) was weighed to the crude. NMR analysis was performed (see section 2.2 NMR analysis), where the aromatic signal at 6.82 ppm was used for quantification.

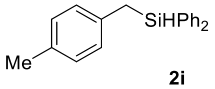

2i

(4-Methylbenzyl)diphenylsilane (2i) was synthesized from (**1i**) according to general procedure for electrochemical deoxygenative silylation and isolated by flash column chromatography on neutral silica gel (100% pentane) as a colourless oil. **^{1}H NMR** (500 MHz, $CDCl_3$) 7.53–7.47 (m, 4H), 7.43–7.31 (m, 6H), 6.97 (m, 2H), 6.90 (m, 2H), 4.93 (t, *J* = 3.8 Hz, 1H), 2.65 (d, *J* = 3.8 Hz, 2H), 2.27 (s, 3H). **^{13}C NMR** (126 MHz, $CDCl_3$) δ 135.3, 134.0, 133.8, 129.69, 129.0, 128.6, 127.9, 21.7, 20.9.
Yields of **2i** were assessed by quantitative ^{1}H-NMR analysis from reaction mixtures. After termination of the silylation reaction (see general procedure for electrochemical deoxygenative silylation) and extraction workup, the volatiles were removed *in vacuo*, and 7.2 mg 1,3,5-trimethoxybenzene (TMB) was weighed to the crude. NMR analysis was performed (see section 2.2 NMR analysis), methylene signal 2.65 (d, *J* = 3.8 Hz, 2H) was used for quantification.

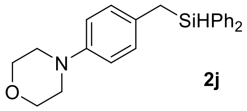

2j

4-(4-((Diphenylsilyl)methyl)phenyl)morpholine (2j) was synthesized from (**1j**) according to General procedure for electrochemical deoxygenative silylation and isolated by flash column chromatography on neutral silica gel (2% Et_2O in pentane) as a white solid. **^{1}H NMR** (500 MHz, $CDCl_3$) 7.44–7.40 (m, 4H), 7.33–7.23 (m, 6H), 6.88–6.81 (m, 2H), 6.73–6.62 (m, 2H), 4.85 (t, *J* = 3.7 Hz, 1H), 3.83–3.70 (m, 4H), 3.04–2.92 (m, 4H), 2.53 (d, *J* = 3.8 Hz, 2H). **^{13}C NMR** (126 MHz, $CDCl_3$) δ 135.4, 134.5, 134.0, 130.2, 129.8, 129.6, 128.1, 116.2, 67.1, 50.0, 21.1. HRMS(HESI) for $C_{23}H_{26}NOSi$ $(M+H)^+$ calculated 360.1784, measured 360.1794.
Yields of **2j** were assessed by quantitative ^{1}H-NMR analysis from reaction mixtures. After termination of the silylation reaction (see general procedure for electrochemical deoxygenative silylation) and extraction workup, the volatiles were removed *in vacuo*, and 10.0 mg 1,3,5-trimethoxybenzene (TMB) was weighed to the crude. After this, NMR analysis was performed (see section 2.2 NMR analysis), signal 4.85 (t, *J* = 3.7 Hz, 1H) was used for quantification.

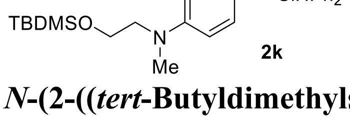

2k

***N*-(2-((*tert*-Butyldimethylsilyl)oxy)ethyl)-4-((diphenylsilyl)methyl)-*N*-methylaniline (2k)** was synthesized from (**1k**, see section 9, synthesis of substrates) according to general procedure for electrochemical deoxygenative silylation and isolated by flash column chromatography on silica gel (30% CH_2Cl_2 in petroleum ether) in 48% yield (colourless oil). **^{1}H NMR** (500 MHz, $CDCl_3$) δ 7.57–7.50 (m, 4H), 7.43–7.32 (m, 4H), 7.19 (m, 2H), 6.90 (m, 2H), 6.58 (m, 2H), 4.96 (t, *J* = 3.7 Hz, 1H), 3.76 (t, *J* = 6.3 Hz, 2H), 3.42 (t, *J* = 6.3 Hz, 2H), 2.94 (s, 3H), 2.61 (d,

J = 3.8 Hz, 2H), 0.91 (s, 10H), 0.05 (s, 6H). **^{13}C NMR** (126 MHz, $CDCl_3$) δ 146.9, 135.5, 134.5, 129.7, 129.6, 128.0, 112.5, 60.7, 55.3, 39.3, 26.1, 20.6, 18.4, -5.2. HRMS(HESI) for $C_{28}H_{40}NOSi_2$ $(M+H)^+$ calculated 462.2648, measured 462.2662.
Yield of **2k** was assessed by quantitative ^{1}H-NMR analysis from reaction mixture. After termination of the silylation reaction (see general procedure for electrochemical deoxygenative silylation) and evaporation of the volatiles, 1,3,5-trimethoxybenzene (7.3 mg) was weighed to the crude. NMR analysis was performed (see section 2.2 NMR analysis), the methylene signal 2.55 ppm was used for quantification.

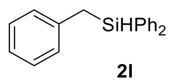

2l

Benzyldiphenylsilane (2l) was synthesized from benzyl alcohol according to general procedure for electrochemical deoxygenative silylation in 85% (NMR yield) after 3 h with 10 equiv. diphenylsilane and isolated by flash column chromatography on silica gel (100% c-hexane) in 34% yield as a colourless oil (28.0 mg, 0.102 mmol). The experimental data matches the literature:[1] **^{1}H NMR** (500 MHz, $CDCl_3$) δ 7.55–7.47 (m, 4H), 7.46–7.30 (m, 6H), 7.18 (m, 2H), 7.09 (m, 1H), 7.02 (m, 2H), 4.97 (t, J = 3.8 Hz, 0H), 2.71 (d, J = 3.8 Hz, 1H). **^{13}C NMR** (126 MHz, $CDCl_3$) δ 138.7, 135.4, 133.7, 129.9, 128.8, 128.4, 128.1, 124.7, 22.4.
Yield of **2l** was assessed by quantitative ^{1}H-NMR analysis from reaction mixtures. After termination of the silylation reaction (see general procedure for electrochemical deoxygenative silylation) and extraction, 1,3,5-trimethoxybenzene (3.5 mg) was weighed to the crude. NMR analysis was performed (see section 2.2 NMR analysis), where the methylene signal 2.71 ppm was used for quantification.

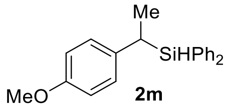

2m

(1-[4-Methoxyphenyl]ethyl)diphenylsilane (2m) was synthesized from 1-(4-methoxyphenyl)ethan-1-ol (**1m**) according to general procedure for electrochemical deoxygenative silylation and isolated by flash column chromatography on neutral silica gel (100% pentane) as a colorless oil. The experimental data matches the literature:[2] **^{1}H NMR** (500 MHz, $CDCl_3$) δ 7.43–7.38 (m, 2H), 7.34–7.30 (m, 2H), 7.19–7.13 (m, 6H), 6.75–6.70 (m, 2H), 6.57–6.52 (m, 2H), 4.63 (d, J = 3.4 Hz, 1H), 3.55 (s, 3H), 2.57 (qd, J = 7.6, 3.4 Hz, 1H), 1.23 (d, J = 7.6 Hz, 3H). **^{13}C NMR** (126 MHz, $CDCl_3$) δ 157.3, 136.4, 135.8, 135.7, 133.4, 133.3, 129.8, 129.7, 128.7, 128.0, 127.9, 113.8, 55.4, 26.0, 17.0.
Yields of **2m** were assessed by quantitative ^{1}H-NMR analysis from reaction mixtures. After termination of the silylation reaction (see general procedure for electrochemical deoxygenative silylation) and extraction workup, the volatiles were removed *in vacuo*, and 5.9 mg 1,3,5-trimethoxybenzene (TMB) was weighed to the crude. NMR analysis was performed (see section 2.2 NMR analysis), signal 4.63 (d, J = 3.4 Hz, 1H) was used for quantification.

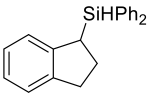

2n

(2,3-Dihydro-1*H*-inden-1-yl)diphenylsilane (2n) was synthesized from 2,3-dihydro-1H-inden-1-ol (**1n**) according to general procedure for electrochemical deoxygenative silylation and isolated by flash column chromatography on neutral silica gel (100% pentane) in 81% yield as a colourless oil. The experimental data matches the literature:[3] **^{1}H NMR** (400 MHz, $CDCl_3$)

δ 7.57–7.40 (m, 4H), 7.39–7.23 (m, 6H), 7.14–7.06 (m, 1H), 7.02 (t, J = 7.3 Hz, 1H), 6.97 (t, J = 7.3 Hz, 1H), 6.90–6.77 (m, 1H), 4.88 (d, J = 3.1 Hz, 1H), 3.16 (dt, J = 8.6, 3.9 Hz, 1H), 2.79 (ddd, J = 14.3, 9.1, 4.5 Hz, 1H), 2.56 (dt, J = 16.2, 8.6 Hz, 1H), 2.36 (dq, J = 13.0, 8.9 Hz, 1H), 2.15 (ddt, J = 13.1, 9.2, 4.4 Hz, 1H). **^{13}C NMR** (101 MHz, $CDCl_3$) δ 145.0, 144.0, 135.8, 135.5, 133.6, 133.3, 129.8, 127.99, 127.95, 126.1, 125.5, 124.5, 124.3, 32.7, 31.3, 28.2.
Yields of **2n** were assessed by quantitative ^{1}H-NMR analysis from reaction mixtures. After termination of the silylation reaction (see general procedure for electrochemical deoxygenative silylation) and extraction workup, the volatiles were removed *in vacuo*, and 8.9 mg 1,3,5-trimethoxybenzene (TMB) was weighed to the crude. NMR analysis was performed (see section 2.2 NMR analysis), signal 6.90–6.77 (m, 1H) was used for quantification.

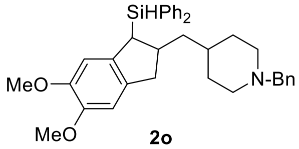

2o

1-Benzyl-4-((1-(diphenylsilyl)-5,6-dimethoxy-2,3-dihydro-1*H*-inden-2-yl)methyl)piperidine (2o) was synthesized from alcohol **1o** according to general procedure for electrochemical deoxygenative silylation and isolated by flash column chromatography on silica gel (3% MeOH in CH_2Cl_2) in 44% as a colourless oil. *Major diastereomer:* **^{1}H NMR** (500 MHz, $CDCl_3$) δ 7.60–7.52 (m, 2H), 7.49– 7.26 (m, 13H), 6.67 (s, 1H), 6.17 (s, 1H), 4.83 (d, J = 3.7 Hz, 1H), 3.84 (s, 3H), 3.52 (s, 2H), 3.50 (s, 3H), 2.92–2.73 (m, 5H), 2.63–2.56 (m, 1H), 2.46 (dd, J = 15.6, 2.7 Hz, 1H), 2.01–1.85 (m, 2H), 1.58 (m, 1H), 1.46 (m, 1H), 1.42–1.28 (m, 2H), 1.25–1.14 (m, 2H). **^{13}C NMR** (126 MHz, $CDCl_3$) δ 147.5, 147.3, 135.9, 135.87, 135.5, 135.2, 133.8, 133.7, 133.2, 129.9, 129.8, 129.5, 128.3, 128.1, 127.97, 127.2, 108.1, 108.0, 63.5, 56.0, 55.6, 53.9, 53.8, 44.4, 38.96, 38.4, 38.34, 33.6, 32.4, 32.2. *Minor diastereomer:* **^{1}H NMR** (500 MHz, $CDCl_3$) δ 5.07 (d, J = 2.1 Hz, 1H). Other signals for the minor diastereomer are overlapping with those for the major diastereomer. Diastereomeric ratio dr 8:1 for **2o** was determined by ^{1}H-NMR analysis from signals 5.07 ppm and 4.83 ppm. HRMS(HESI) for $C_{36}H_{42}NO_2Si$ $(M+H)^+$ calculated 548.2985, measured 548.3007.
Yields of **2o** were assessed by quantitative ^{1}H-NMR analysis from reaction mixtures. After termination of the silylation reaction (see general procedure for electrochemical deoxygenative silylation) and extraction workup, the volatiles were removed *in vacuo*, and 1,3,5-trimethoxybenzene (TMB) was weighed to the crude. NMR analysis was performed (see section 2.2 NMR analysis), signal 2.61 (d, J = 3.8 Hz, 2H) was used for quantification.

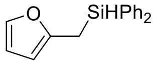

2p

(Furan-2-ylmethyl)diphenylsilane (2p) was synthesized from (**1p**) according to general procedure for electrochemical deoxygenative silylation and isolated by flash column chromatography on silica gel (2% EtOAc in pentane) as a colourless oil. **^{1}H NMR** (500 MHz, $CDCl_3$) δ 7.63–7.49 (m, 4H), 7.47–7.29 (m, 6H), 7.28–7.20 (m, 1H), 6.23 (t, J = 2.5 Hz, 1H), (m, 1H), 5.84 (d, J = 3.1 Hz, 1H), 5.07 (t, J = 3.6 Hz, 1H), 2.71 (d, J = 3.6 Hz, 1H). **^{13}C NMR** (126 MHz, $CDCl_3$) δ 152.4, 140.6, 136.0, 135.3, 135.1, 135.0, 134.7, 134.5, 133.4, 131.2, 130.6, 130.4, 130.0, 128.5, 128.2, 128.2, 128.1, 110.7, 105.4, 99.2, 13.9. HRMS(HESI) for $C_{17}H_{17}OSi$ $(M+H)^+$ calculated 265.1049 measured 265.1046.
Yields of **2p** were assessed by quantitative ^{1}H-NMR analysis from reaction mixtures. After termination of the silylation reaction (see general procedure for electrochemical deoxygenative

silylation) and extraction workup, the volatiles were removed *in vacuo*, and 8.0 mg 1,3,5-trimethoxybenzene (TMB) was weighed to the crude. NMR analysis was performed (see section 2.2 NMR analysis), signal 5.07 (t, J = 3.6 Hz, 1H) was used for quantification.

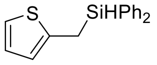

2q

Diphenyl(thiophen-2-ylmethyl)silane (2q) was synthesized from 2-thiophenemethanol (**1q**) according to general procedure for electrochemical deoxygenative silylation in 15% NMR-yield after 3 h with 5 equiv. diphenylsilane, and in 38% NMR-yield after 2 h with 10 equiv. diphenylsilane. Product **2q** was isolated by flash column chromatography on silica gel (10% CH_2Cl_2 in pentane) followed by flash column chromatography on reversed phase silica gel C18-RP (30% H_2O in acetonitrile) in 10% as a colourless oil (9.1 mg, 0.03 mmol). **^{1}H NMR** (500 MHz, $CDCl_3$) 7.56–7.50 (m, 4H), 7.45–7.32 (m, 6H), 6.98 (dd, J = 5.2, 1.2 Hz, 1H), 6.82 (dd, J = 5.2, 3.4 Hz, 1H), 6.60 (d, J = 3.4 Hz, 1H), 5.03 (t, J = 3.6 Hz, 1H), 2.89 (d, J = 3.7 Hz, 2H). **^{13}C NMR** (126 MHz, $CDCl_3$) δ 140.6, 135.4, 133.3, 130.1, 128.2, 127.1, 124.6, 122.4, 15.9. HRMS(HESI) for $C_{17}H_{17}SSi$ $(M+H)^+$ calculated 281.0820 measured 281.0822.
Yields of **2q** were assessed by quantitative ^{1}H-NMR analysis from reaction mixtures. After termination of the reaction (see general procedure for electrochemical deoxygenative silylation) and extraction workup, 8.2 mg 1,3,5-trimethoxybenzene was added to the crude. NMR analysis was performed (see section 2.2 NMR analysis), aromatic signal 6.6 ppm (d, J = 3.4 Hz, 1H) of **2q** was used for quantifications.

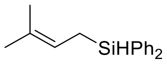

2r

(3-Methylbut-2-en-1-yl)diphenylsilane (2r) was synthesized from 1,1-dimethylallyl alcohol (**1r**) according to general procedure for electrochemical deoxygenative silylation in 49% (NMR yield) and isolated by flash column chromatography on silica gel (100% petroleum ether) as a colourless oil. The experimental data matches the literature:[4] **^{1}H NMR** (500 MHz, $CDCl_3$) δ 7.59 – 7.54 (m, 4H), 7.42 – 7.33 (m, 6H), 5.23 (ddt, J = 9.7, 6.8, 1.5 Hz, 1H), 4.83 (t, J = 3.6 Hz, 1H), 2.02 (dd, J = 8.2, 3.5 Hz, 2H), 1.66 (s, 3H), 1.47 (s, 3H). **^{13}C NMR** (126 MHz, $CDCl_3$) δ 135.33, 134.47, 131.14, 129.69, 128.03, 118.39, 25.86, 17.75, 14.23.
Yields of **2r** were assessed by quantitative ^{1}H-NMR analysis from reaction mixtures. After termination of the silylation reaction (see general procedure for electrochemical deoxygenative silylation) and extraction workup, the volatiles were removed *in vacuo*, and 4.2 mg 1,3,5-trimethoxybenzene (TMB) was weighed to the crude. NMR analysis was performed (see section 2.2 NMR analysis), methylene signal 5.23 (ddt, J = 9.7, 6.8, 1.5 Hz, 1H), was used for quantification.

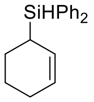

2s

Cyclohex-2-en-1-yldiphenylsilane (2s) was synthesized from (**1s**) according to general procedure for electrochemical deoxygenative silylation in 56% (NMR yield) and isolated by flash column chromatography on neutral silica gel (100% pentane) as a colourless oil. The experimental data matches the literature:[5] **^{1}H NMR** (500 MHz, $CDCl_3$) δ 7.68–7.64 (m, 2H), 7.62–7.58 (m, 2H), 7.44–7.36 (m, 6H), 5.78–5.68 (m, 2H), 4.82 (d, J = 3.8 Hz, 1H), 2.33–2.24

(m, 1H), 2.12–1.99 (m, 2H), 2.98–1.90 (m, 1H), 1.80–1.67 (m, 2H), 1.62–1.54 (m, 1H). **^{13}C NMR** (101 MHz, $CDCl_3$) δ 135.7, 135.4, 133.8, 133.5, 129.7, 129.6, 128.0, 127.2, 126.7, 25.0, 24.5, 23.3, 22.1.

Yields of **2s** were assessed by quantitative ^{1}H-NMR analysis from reaction mixtures. After termination of the silylation reaction (see general procedure for electrochemical deoxygenative silylation) and extraction workup, the volatiles were removed *in vacuo*, and 7.0 mg 1,3,5-trimethoxybenzene (TMB) was weighed to the crude. NMR analysis was performed (see section 2.2 NMR analysis), signal 4.82 (d, J = 3.8Hz, 1H) was used for quantification.

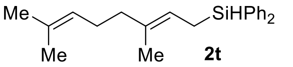

2t **(*E*)-(3,7-Dimethylocta-2,6-dien-1-yl)diphenylsilane (2t)** was synthesized from geraniol according to general procedure for electrochemical deoxygenative silylation and isolated by flash column chromatography on neutral silica gel (100% pentane) in 44% as a colourless oil. **^{1}H NMR** (500 MHz, $CDCl_3$) δ ^{1}H NMR (500 MHz, $CDCl_3$) δ 7.60–7.56 (m, 4H), 7.43–7.34 (m, 6H), 5.26 (m, 1H), 5.06 (m, 1H), 4.84 (t, J = 3.6 Hz, 1H), 2.07–1.94 (m, 6H), 1.67 (s, 3H), 1.59 (, 3H), 1.47 (d, J = 1.3 Hz, 3H). ^{13}C NMR (126 MHz, $CDCl_3$) δ 135.4, 134.8, 134.5, 131.4, 129.7, 128.0, 124.5, 118.5, 40.0, 26.9, 25.8, 17.8, 16.0, 14.2.

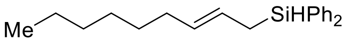

2u **(E)-Non-2-en-1-yldiphenylsilane (2u)** was synthesized from *(E)*-2-nonen-1-ol according to general procedure for electrochemical deoxygenative silylation and isolated by flash column chromatography on neutral silica gel (100% pentane) in 72% as a colourless oil. ^{1}H NMR (500 MHz, $CDCl_3$) δ 7.63–7.55 (m, 4H), 7.45–7.35 (m, 6H), 5.53–5.41 (m, 1H), 5.41–5.29 (m, 1H), 4.87 (t, J = 3.4 Hz, 1H), 2.10–2.05 (m, 2H), 1.95 (m, 2H), 1.52 – 1.37 (m, 1H), 1.26 (m, 8H), 0.90 (m, 3H). ^{13}C NMR (126 MHz, $CDCl_3$) δ 135.41, 134.20, 131.45, 129.72, 128.04, 124.47, 77.41, 77.16, 76.91, 32.88, 31.91, 29.82, 28.87, 22.79, 18.03, 14.27.

6. Electroreductive carboxylation of alcohols 1 and 6a

Electroreductive carboxylation of benzyl alcohols **1** was assessed by omitting diphenyl silane under CO_2 atmosphere (Figure S-8). Only *p*-CO_2Me and *p*-CN benzyl alcohols gave the respective carboxylic acids as the C–C coupling products. No carboxylation was observed with silyl ether **6a** (Figure S-9). For synthesis of **6a** see Section 9.

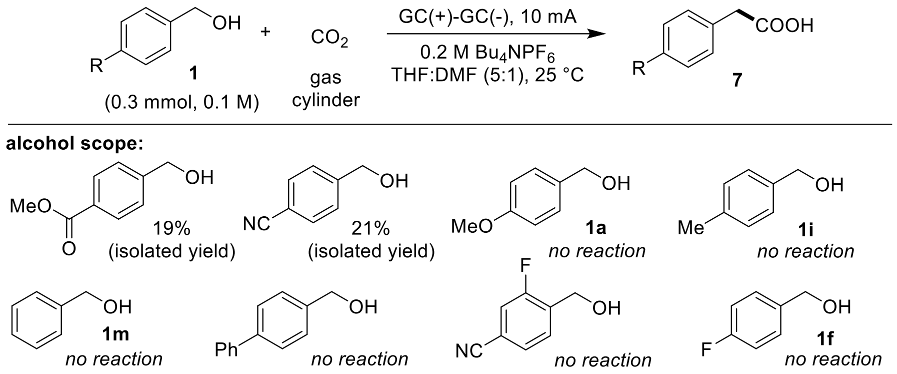

Figure S-8. Electroreductive deoxygenative carboxylation of benzyl alcohols **1**. Isolated yields.

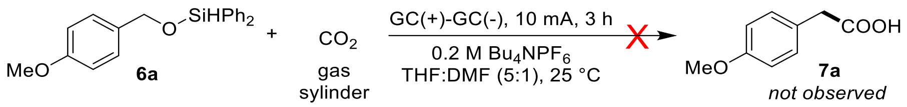

Figure S-9. Electroreductive deoxygenative carboxylation of **6a**. Assessed by NMR analysis.

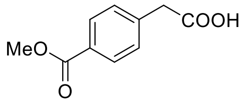

2-[4-(Methoxycarbonyl)phenyl]acetic acid was synthesized from methyl 4-(hydroxymethyl)benzoate according to general procedure for electrochemical deoxygenative carboxylation (Section 4.2), isolated by acid-base extraction using basic Milli-Q® water (K_2CO_3, pH 11) and EtOAc, then acidifying with aqueous 1 M HCl until pH 3. The product was obtained in 19% as a white solid (11.3 mg, 0.06 mmol) after concentration *in vacuo*. The experimental data matches the literature:[6] **^{1}H NMR** (500 MHz, $CDCl_3$), δ 7.99 (m, 2H), δ 7.36 (m, 2H), δ 3.91 (s, 3H), δ 3.70 (s, 2H). **^{13}C NMR** (126 MHz, $CDCl_3$), δ 175.8, δ 167.0, δ 138.7, δ 130.0, δ 129.6, δ 129.3, δ 52.3, δ 41.1.

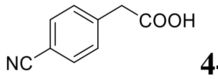

4-Cyanophenylacetic acid was synthesized according to general procedure for electrochemical deoxygenative carboxylation (Section 4.2), isolated by acid-base extraction using basic Milli-Q® water (K_2CO_3, pH 11) and EtOAc, then acidifying with aqueous 1 M HCl until pH 3. The product was obtained in 21% as a white solid (10.3 mg, 0.06 mmol) after concentration *in vacuo*. The experimental data matches the literature:[7] **^{1}H NMR** (500 MHz, $CDCl_3$), δ 7.64 (m, 2H), 7.41 (m, 2H), 3.73 (s, 2H). **^{13}C NMR** (126 MHz, $CDCl_3$), δ 175.9, 138.6, 132.6, 130.4, 118.7, 111.7, 40.9.

7. Derivatization of hydrosilanes 2

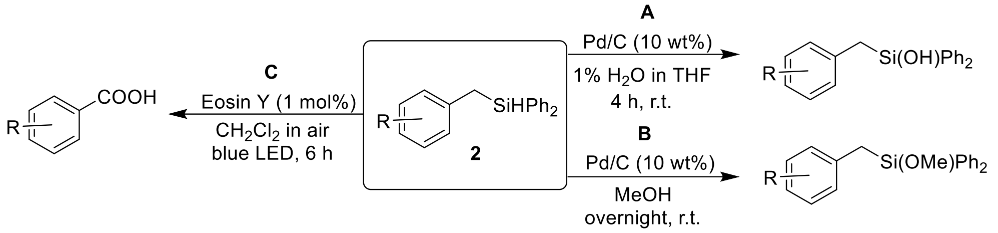

Figure S-10. Derivatization of hydrosilanes according to literature procedures **A)** to silanol,[8] **B)** methoxysilane[9] and **C)** carboxylic acid via Tamao-oxidation.[10]

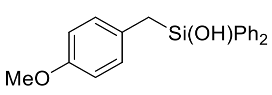

(4-Methoxybenzyl)diphenylsilanol was synthesized from **2a** following a literature procedure (Figure S-10, A)[8] and isolated by flash chromatography (5% EtOAc in pentane). **^{1}H NMR** (500 MHz, $CDCl_3$) *δ* 7.59–7.51 (m, 4H), 7.44–7.40 (m, 2H), 7.36 (m, 4H), 6.96–6.88 (m, 2H), 6.75–6.70 (m, 2H), 3.75 (s, 3H), 2.64 (s, 2H). **^{13}C NMR** (126 MHz, $CDCl_3$) *δ* 157.2, 135.6, 134.5, 130.15, 129.8, 129.4, 128.0, 114.1, 55.4, 23.9. HRMS(HESI$^-$) for $C_{20}H_{19}O_2Si$ $[M-H]^-$ calculated 319,1154, found 319.1155.

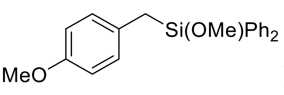

Methoxy(4-methoxybenzyl)diphenylsilanol was synthesized from **2a** following a literature procedure (Figure S-10, B)[9] and isolated by flash chromatography (5% EtOAc in pentane). **^{1}H NMR** (500 MHz, $CDCl_3$) *δ* 7.39–7.32 (m, 4H), 7.29 (m, 2H), 7.17 (m, 4H), 6.89 (m, 2H), 6.69 (m, 2H), 3.75 (s, 3H), 3.52 (s, 3H), 2.65 (s, 2H). **^{13}C NMR** (126 MHz, $CDCl_3$) *δ* 157.1, 135.0, 134.5, 130.1, 130.1, 130.0, 127.9, 127.7, 113.8, 55.4, 51.8, 22.5.

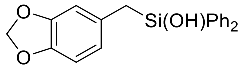

(Benzo[d][1,3]dioxol-5-ylmethyl)diphenylsilanol was synthesized from **2c** following a literature procedure (Figure S-10, A)[1] and isolated by flash chromatography (10% Et_2O in pentane). **^{1}H NMR** (500 MHz, $CDCl_3$) *δ* 7.58–7.54 (m, 4H), 7.46–7.40 (m, 2H), 7.40–7.34 (m, 4H), 6.63 (d, *J* = 8.0 Hz, 1H), 6.50 (d, *J* = 1.7 Hz, 1H), 6.45 (dd, *J* = 8.0, 1.7 Hz, 1H), 5.87 (s, 2H), 2.62 (s, 2H). **^{13}C NMR** (126 MHz, $CDCl_3$) *δ* 147.7, 145.0, 135.5, 134.5, 131.2, 130.2, 128.1, 121.5, 109.5, 108.4, 100.8, 24.8. HRMS(HESI) for $C_{22}H_{19}O_3Si$ $(M+H)^+$ calculated 335.1103, measured 335.1106.

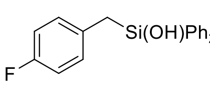

(4-Fluorobenzyl)diphenylsilanol was synthesized from **2f** following a literature procedure (Figure S-10, A)[1] and isolated by flash chromatography (10% Et_2O in pentane). **^{1}H NMR** (400 MHz, $CDCl_3$) *δ* 7.57 – 7.50 (m, 4H), 7.47 – 7.33 (m, 6H), 6.93 (dd, *J* = 8.4, 5.5 Hz, 2H), 6.85 (t, *J* = 8.6 Hz, 2H), 2.66 (s, 2H). **^{13}C NMR** (126 MHz, $CDCl_3$) *δ* 161.97, 159.56, 135.15, 134.45, 130.30, 130.14, 130.07, 128.08, 115.31, 115.10, 77.36, 24.37. HRMS(HESI) for $C_{19}H_{16}FOSi$ $(M-H)^-$ calculated 307.0954, measured 307.0958.

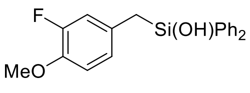

(3-Fluoro-4-methoxybenzyl)diphenylsilanol was synthesized from **2g** following a literature procedure (Figure S-10, A)[1] and isolated by flash chromatography (10% Et_2O in pentane). **^{1}H NMR** (400 MHz, CDCl3) *δ* 7.54 (d, *J* = 7.1 Hz, 4H), 7.43 (dd, *J* = 8.5, 6.1 Hz, 2H), 7.37 (t, *J* = 7.3 Hz, 5H), 6.80 – 6.66 (m, 3H), 3.82 (s, 3H), 2.62 (s, 2H). **^{13}C NMR**

(126 MHz, $CDCl_3$) δ 153.32, 151.38, 135.19, 134.49, 134.44, 130.31, 128.11, 124.36, 124.34, 116.79, 116.64, 113.76, 113.74, 56.51, 24.21. HRMS(HESI) for $C_{20}H_{18}FO_2Si$ $(M-H)^-$ calculated 337.1060, measured 337.1065.

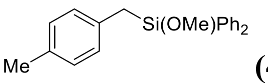

(4-Methylbenzyl)diphenylsilanol was synthesized from **2i** following a literature procedure (Figure S-10, A).[1] HRMS(HESI) for $C_{20}H_{19}OSi$ $(M-H)^-$ calculated 303.1205, measured 303.1201.

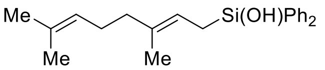

(*E*)-3,7-Dimethyl-2,6-octadien-1-yl)diphenylsilanol was synthesized from **2t** following a literature procedure (Figure S-10, A)[1] and isolated by filtration via celite as a beige oil in >99% yield (19.9 mg, 0.06 mmol). **^{1}H NMR** (500 MHz, $CDCl_3$) δ ^{1}H NMR (500 MHz, $CDCl_3$) δ 7.66–7.56 (m, 4H), 7.49–7.31 (m, 6H), 5.25 (m, 1H), 5.07 – 4.97 (m, 1H), 2.13–1.95 (m, 6H), 1.68 (s, 3H), 1.59 (s, 3H), 1.50 (s, 3H). **^{13}C NMR** (126 MHz, $CDCl_3$) δ 136.2, 135.3, 134.4, 131.8, 130.0, 127.98, 124.6, 117.8, 40.0, 26.7, 25.9, 17.8, 17.4, 16.0. HRMS(HESI) for $C_{22}H_{29}OSi$ $(M+H)^+$ calculated 337.1988, measured 337.1982.

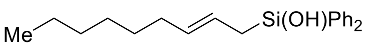

(E)-Non-2-en-1-yldiphenylsilanol was synthesized from **2u** following a literature procedure (Figure S-10, A)[1] and isolated by filtration via celite as a beige oil. HRMS(HESI) for $C_{21}H_{29}OSi$ $(M+H)^+$ calculated 325.1988, measured 325.2008.

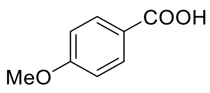

4-Methoxybenzoic acid was synthesized from **2a** following a literature procedure (Figure S-10, C)[10] isolated by flash chromatography (50% EtOAc in pentane). Data in good accordance with literature:[11] **^{1}H NMR** (500 MHz, $CDCl_3$) δ 8.07 (m, 2H), 6.95 (m, 2H), 3.88 (s, 3H). **^{13}C NMR** (126 MHz, $CDCl_3$) δ 171.1, 164.2, 132.5, 121.7, 113.9, 55.6.

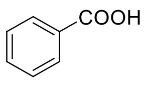

Benzoic acid was synthesized from **2l** following a literature procedure (Figure S-10, C)[10] and isolated by flash chromatography (5% EtOAc in pentane) in 25% yield. Data in good accordance with literature:[11] **^{1}H NMR** (500 MHz, $CDCl_3$) δ 8.1 –8.10 (m, 2H), 7.63 (m, 1H), 7.49 (m, 2H). **^{13}C NMR** (126 MHz, $CDCl_3$) δ 171.9, 133.9, 130.4, 129.4, 128.7.

8. Mechanistic studies

8.1 Formation of THF-acetals under electrochemical conditions

During investigations into electrochemical silylation of alcohol **1a**, traces of THF-acetals **5a** could be observed by NMR analysis (Figure S-11). For synthesis of **5a** see section 9 in supporting information.

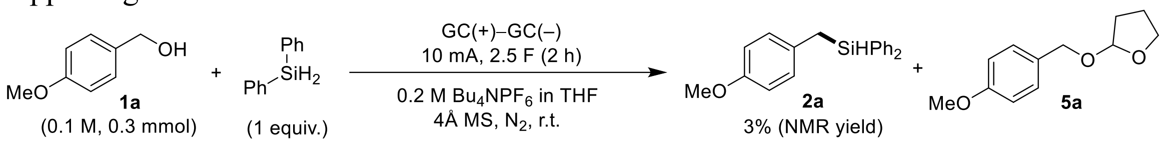

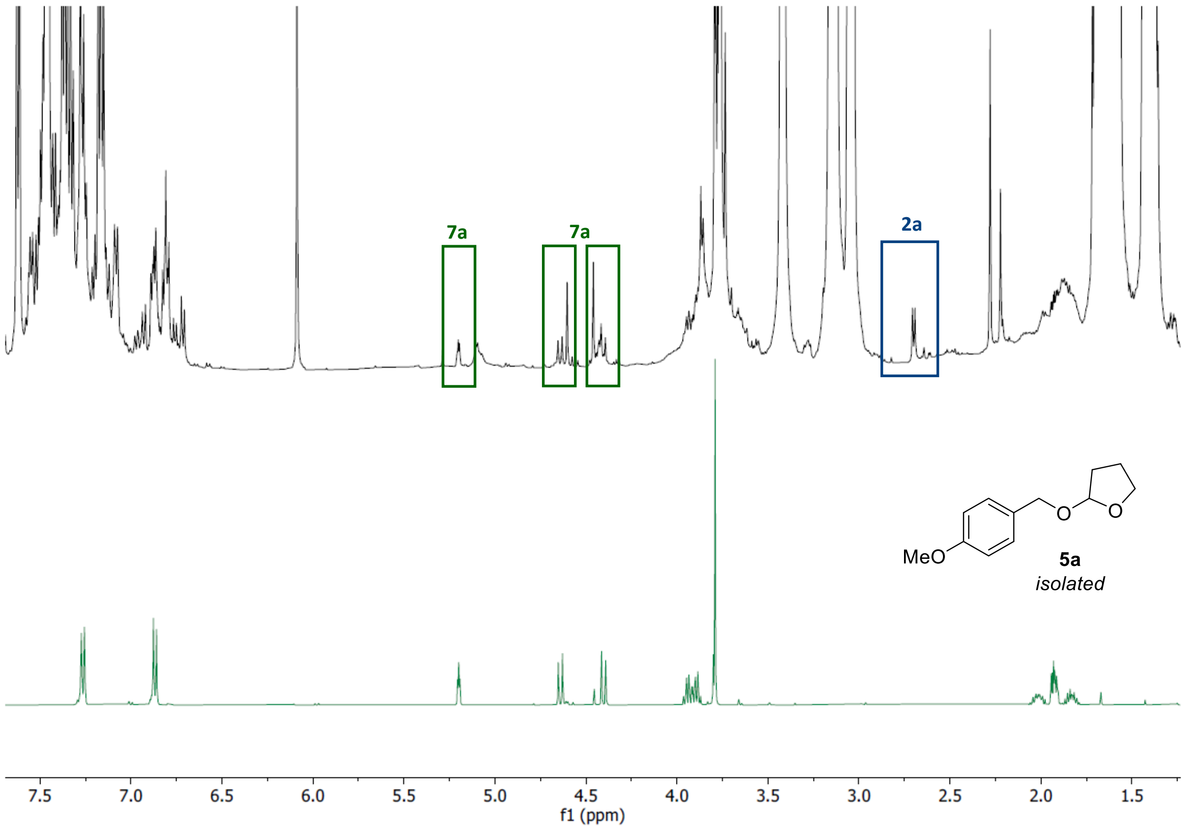

Figure S-11. Comparison of ^{1}H-NMR (500 MHz, $CDCl_3$) spectra of electrochemical silylation of **1a** with 1 equiv. diphenyl silane, crude reaction mixture (*top*) and isolated THF-acetal **5a** (*bottom*).

8.2 Formation of *O*-silyl ethers under electrochemical conditions

The formation of silyl-ether **6a** under silylation reaction conditions was assessed by NMR analysis. A silylation reaction using THF-d8 as one of the solvents was analyzed by ^{1}H-NMR analaysis directly without any prior workup (Figure S-12). That was compared to ^{1}H-NMR of a separately synthesized and isolated silyl-ether **6a** (see section 9).

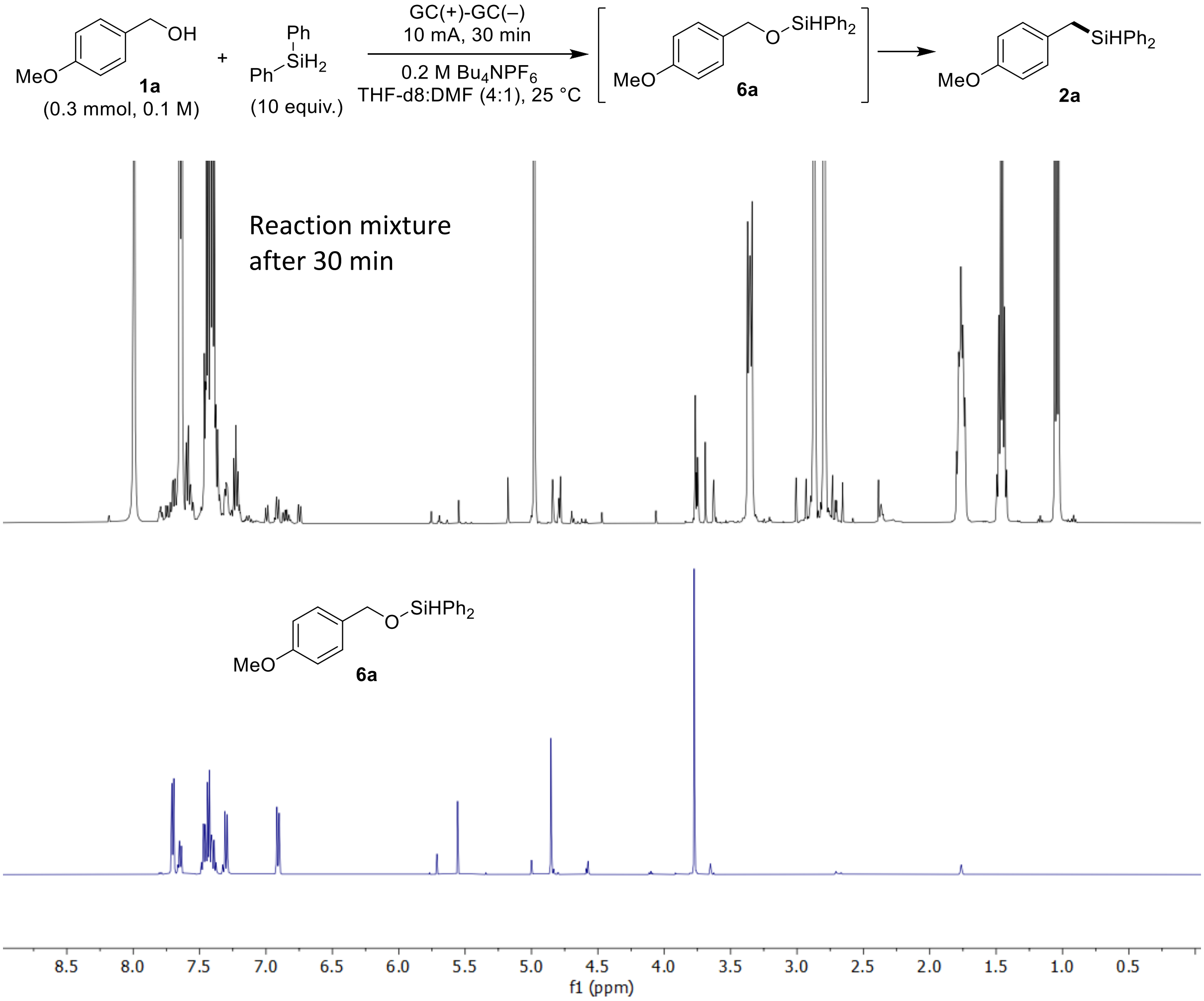

Figure S-12. Comparison of ^{1}H-NMR (500 MHz, THF-d8) spectra of electrochemical silylation of **1a** in (THF-d8):DMF after 30 min (*top*) and synthesized and isolated **6a** (*bottom*).

8.3 Electrochemical silylation of *O*-silyl ethers

O-Silyl ether **6a** was used as substrate under electrochemical silylation conditions and compared to results from **1a** silylation (Figure S-13). Both reactions were sampled over time and assessed by HPLC analysis. For synthesis of **6a** see section 9.

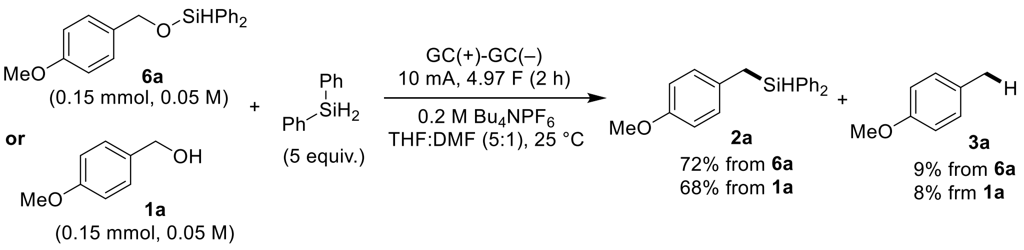

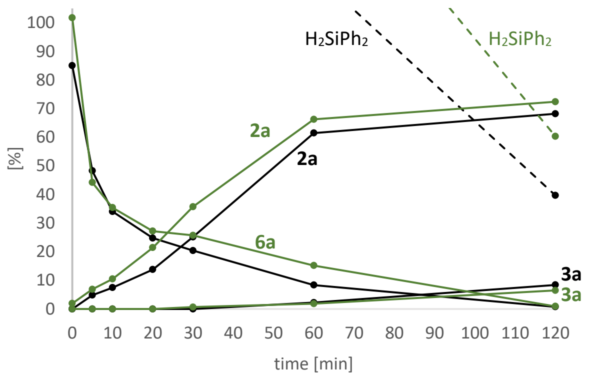

Figure S-13. Comparison of electrochemical silylation of **6a** (green) to electrochemical silylation of **1a** (black) using 5 equiv. H_2SiPh_2. Consumption of **1a**, **6a**, H_2SiPh_2 and formation of **2a** and **3a** are shown. Both reactions were sampled over time and analyzed by HPLC.

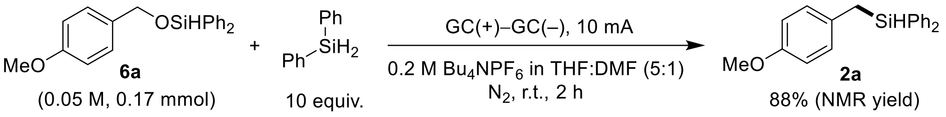

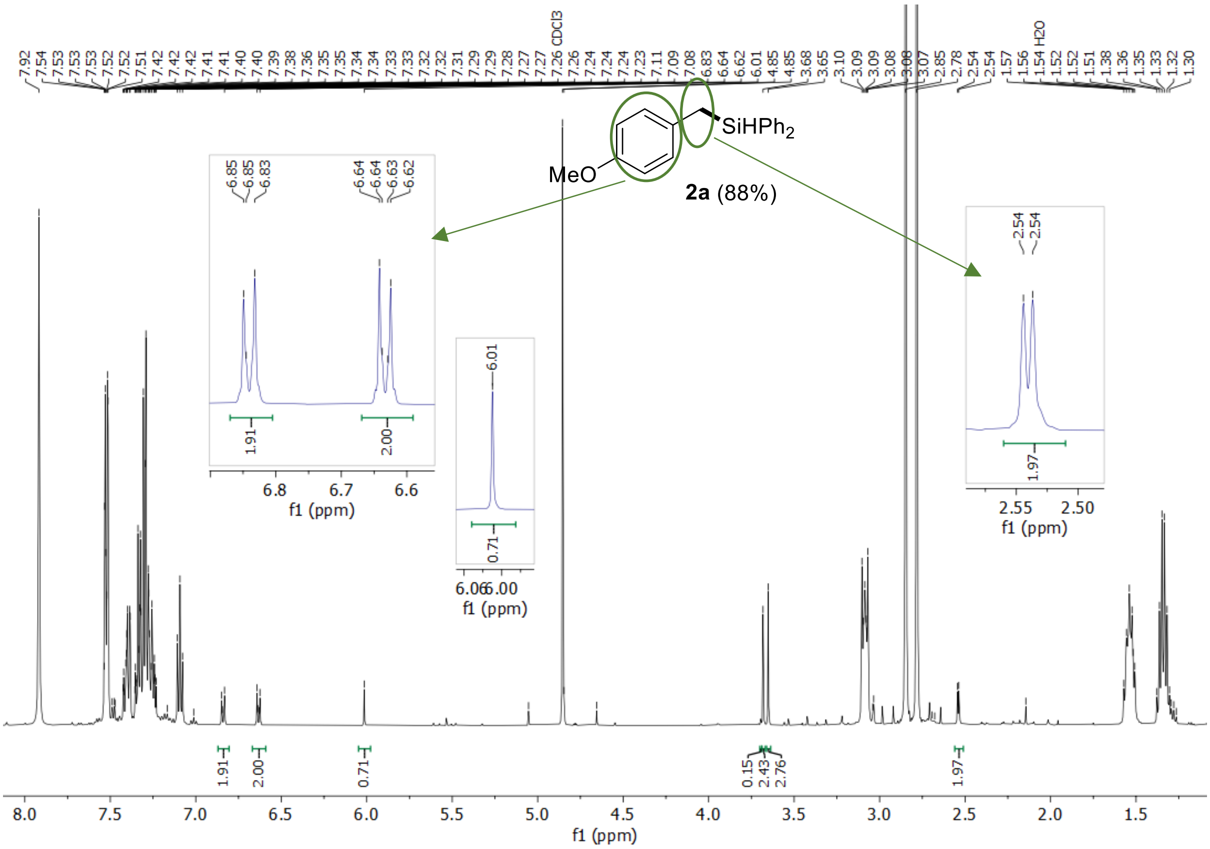

Figure S-14. Electrochemical silylation of **6a** with 10 equiv. H_2SiPh_2 giving **2a** after 2 h. Sealed under nitrogen, not sampled. ^{1}H-NMR (500 MHz, $CDCl_3$) analysis of the reaction mixture with 1,3,5-trimethoxybenzene (6.01 ppm) as internal standard after evaporation of volatiles.

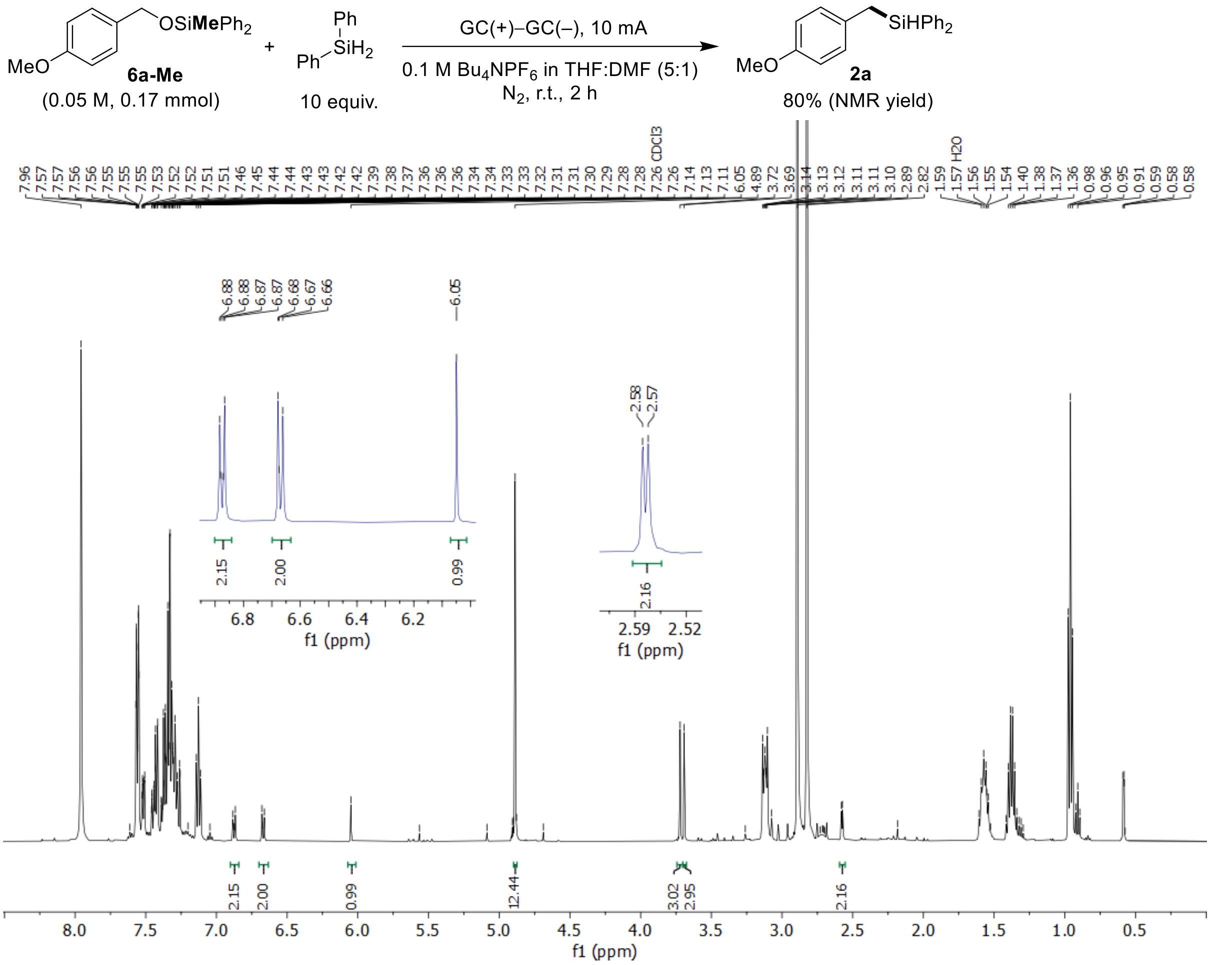

Figure S-15. Electrochemical silylation of **6a-Me** with 10 equiv. H_2SiPh_2 giving **2a** after 2 h. Sealed under nitrogen, not sampled. ^{1}H-NMR (500 MHz, $CDCl_3$) analysis of the reaction mixture with 1,3,5-trimethoxybenzene (6.05 ppm) as internal standard after evaporation of volatiles.

8.4 Electrochemical silylation of THF- and THP-acetals

The synthesized THF- and THP-acetals and the corresponding alcohol **1a** were assessed under electrochemical silylation conditions (Table S-9). None of the acetals tested outperformed the parent alcohol for the formation of the silylated product.

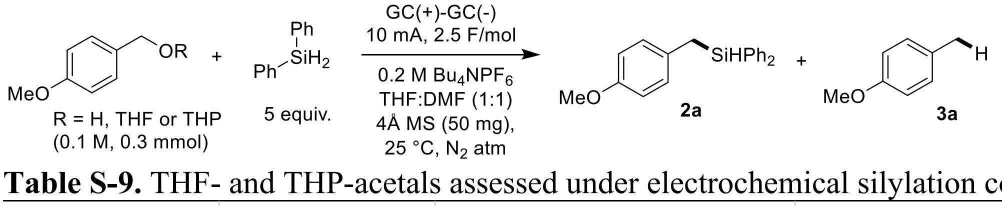

Table S-9. THF- and THP-acetals assessed under electrochemical silylation conditions.

Entry	R	Yield 2a [%][a]	Yield 3a [%][a]
1	H	74	0
2[b]	THF	50	0
3	THP	41	0

[a] Yields determined by ^{1}H-NMR analysis with 1,3,5-trimethoxybenzene as internal standard; [b]10 equiv. diphenyl silane in THF:DMF (4:1)

8.5. Formation of butyldiphenylsilane under electrochemical conditions

3-Phenyl-1-propanol and the corresponding THP ether as starting materials did not give the desired coupling product with diphenylsilane. The starting materials remained mostly unreactive throughout the reaction. However, traces of butyldiphenylsilane were observed when supporting electrolyte Bu_4NPF_6 was used. The isolated compound from the reaction with THP-acetal and diphenylsilane corresponded to the data reported in the literature for butyldiphenylsilane.[12] In case when KPF_6 was used, no such silylated compound was observed (Figure S-16).

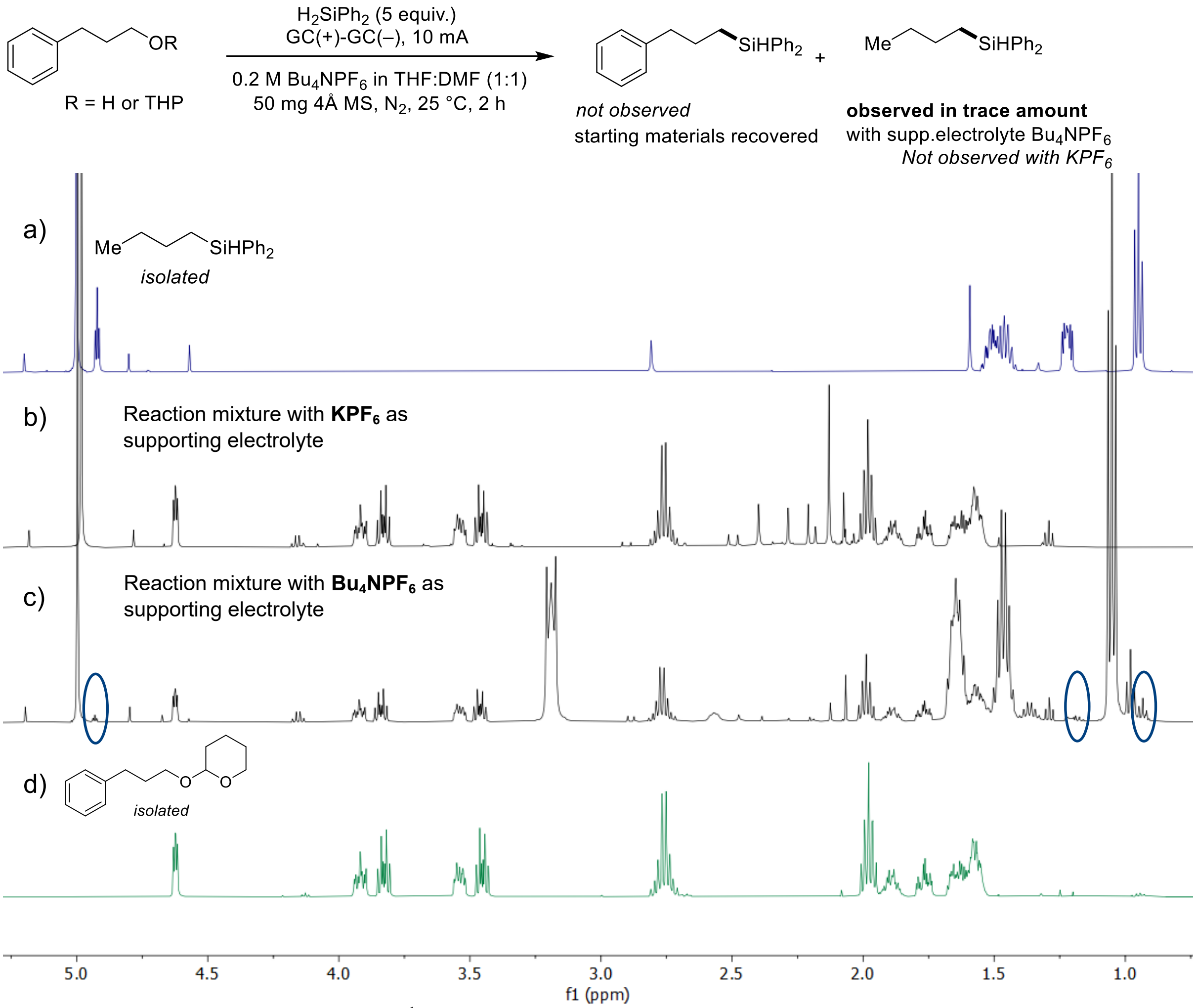

Figure S-16. Comparison of ^{1}H-NMR (500 MHz, $CDCl_3$) spectra of a) isolated butyl-diphenylsilane, b) silylation reaction of THP-acetal with KPF_6 as supporting electrolyte, c) silylation reaction of THP-acetal with Bu_4NPF_6 as supporting electrolyte. Traces of butyldiphenylsilane marked with blue rings, d) isolated THP-acetal.

8.6. Mechanistic control reactions

Common radical traps like TEMPO and 1,1-diphenylethylene (DPE) are not suitable under the herein described electroreductive conditions, as these compounds undergo reduction at more anodic potentials compared to the target compounds (see voltammograms in section 11), thereby losing the ability to act as a radical traps under these conditions. Instead, cyclization - a classic probe for detecting the existence of radical intermediates with sufficient lifetime - was used to probe the existence of such radical intermediates under the silylation conditions, using a benzylic alcohol with a pendant alkene side-chain - (2-allylphenyl)methanol (Figure S-17). However, the reaction using this compound did not result in the formation of cyclized product but only the open-chain silylated product, thereby supporting the proposed carbanionic mechanism.

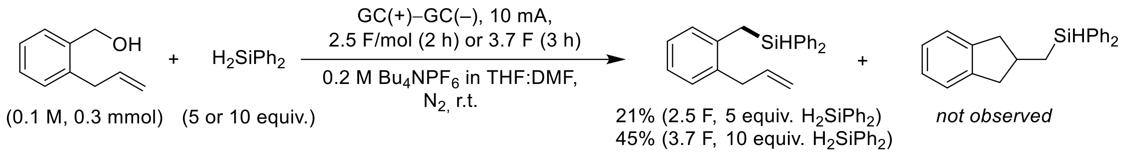

Figure S-17. Behavior of (2-Allylphenyl)methanol under silylation conditions. Yields were determined using ^{1}H-NMR analysis with 1,3,5-trimethoxybenzene as internal standard.

Similar to the proposed mechanism for the electroreductive deoxygenative transformation of benzylic alcohols, benzyl halides are known to undergo reductive mesolytic bond scission to carbon-centered radicals that can undergo a second electron transfer in a radical-polar crossover to furnish carbanions. The use of benzyl chloride and bromide under the silylation conditions afforded the silylated product **2l** along with alkane **3l** (Figure S-18), thereby supporting the proposed mechanism. However, the yields of **2l** was in these cases considerably lower (~20%) compared to that observed when benzyl alcohol was used (85%, Figure 3 in main text) as analyzed by HPLC. While an in depth study of the mechanistic features behind these differences is the topic of a separate study, it may be assumed that the silane concentration is reduced via reaction with the released halide as demonstrated by Lennox and co-workers.[13] Such reduced silane concentration affects the yield of the silylated product negatively (see section 3.7).

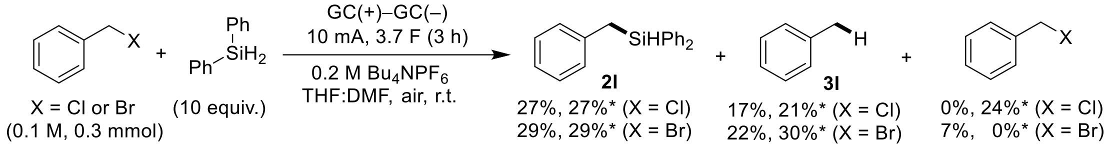

Figure S-18. Benzyl bromide and benzyl chloride under silylation conditions. Sampled over time, analyzed by HPLC. The yields with an asterisk (*) correspond to reactions not sampled over time.

9. Synthesis of 6a, 8a, 1k, 1o and THF- and THP-acetals

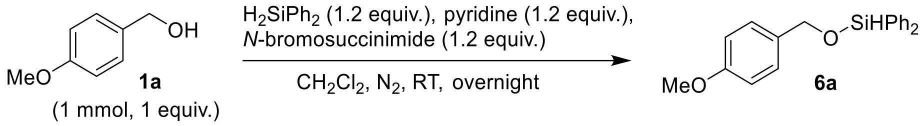

Compound **6a** was synthesized from **1a** according to a literature procedure.[14] Due to **6a** readily decomposing to **1a** by hydrolysis on silica gel, the mixture was used as is after filtration through celite and concentation in vacuo. Data is in good correspondence to literature:[14] **^{1}H NMR** (500 MHz, $CDCl_3$) δ 7.66 (m, 4H), 7.42 (m, 6H), 7.22 (m, 2H), 5.53 (bs, 1H), 4.74 (bs, 2H), 3.79 (bs, 3H). **^{13}C NMR** (126 MHz, $CDCl_3$) δ 159.0, 135.2, 135.6, 134.9, 134.6, 134.5, 134.4, 132.7, 130.6, 130.5, 128.4, 128.2, 128.0, 113.8, 64.9, 55.4. The ^{1}H- and ^{13}C-NMR (500 MHz, THF-d8) are seen in Figure S-19.

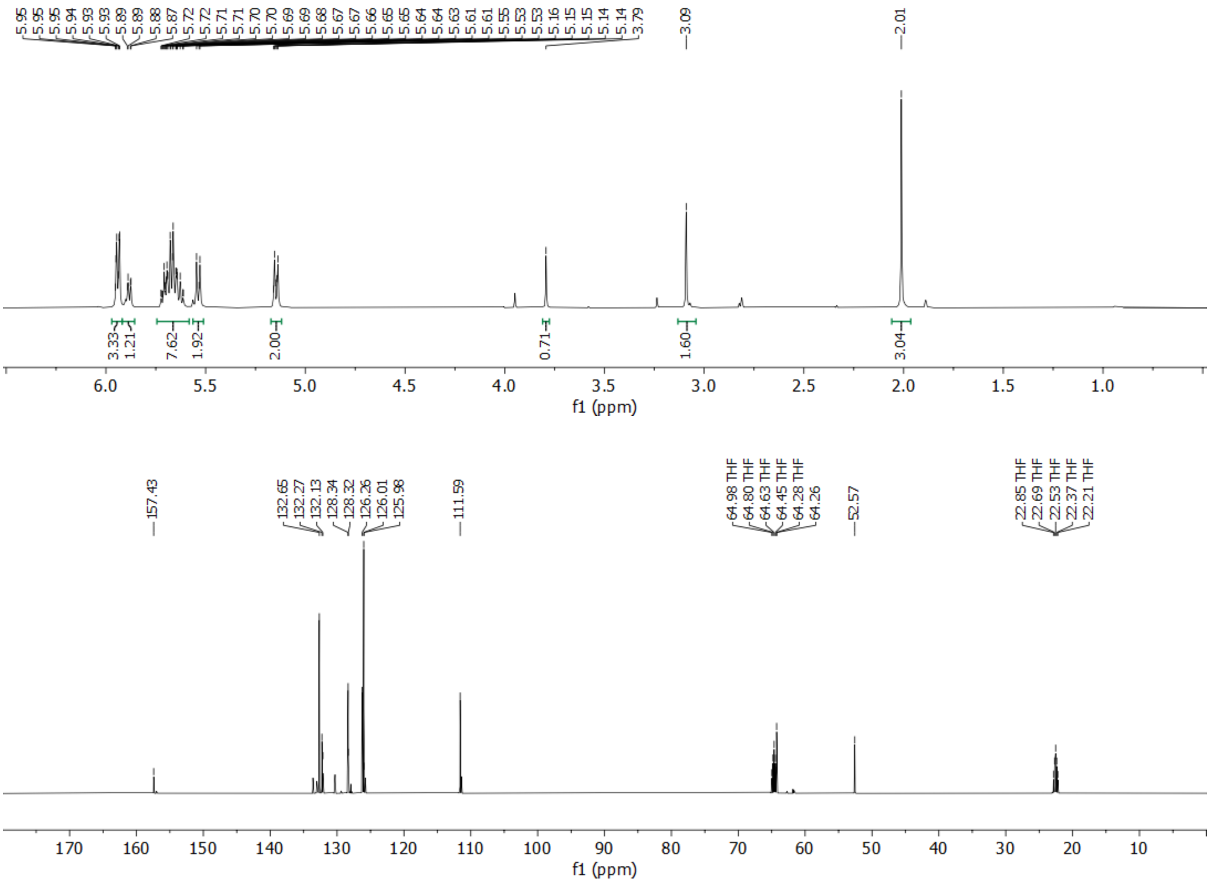

Figure S-19. ^{1}H-NMR (500 MHz, THF-d8) and ^{13}C-NMR (126 MHz, THF-d8) spectra of **6a**. after filtration through celite with pentane and evaporation of volatiles.

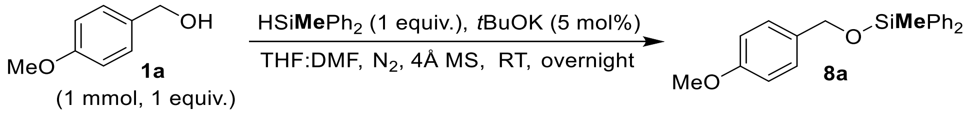

Compound **8a** was synthesized from **1a** according to a literature procedure[15] and isolated by flash column chromatography on silica gel (50% CH_2Cl_2/petroleum ether) in 14% yield (colourless oil). Experimental data matches literature:[16] **^{1}H NMR** (500 MHz, $CDCl_3$) δ 7.49 (m, 4H), 7.32–7.23 (m, 6H), 7.12 (m, 2H), 6.74 (m, 2H), 4.61 (s, 2H), 3.68 (s, 3H), 0.53 (s, 3H). **^{13}C NMR** (126 MHz, $CDCl_3$) δ 158.9, 136.1, 134.6, 132.9, 130.0, 128.2, 128.0, 113.8, 65.2, 55.4, -2.7.

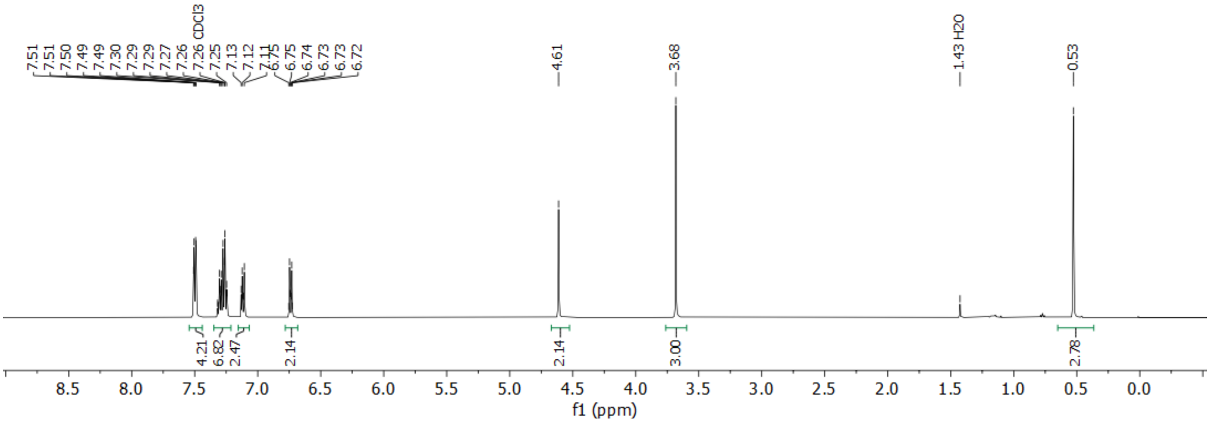

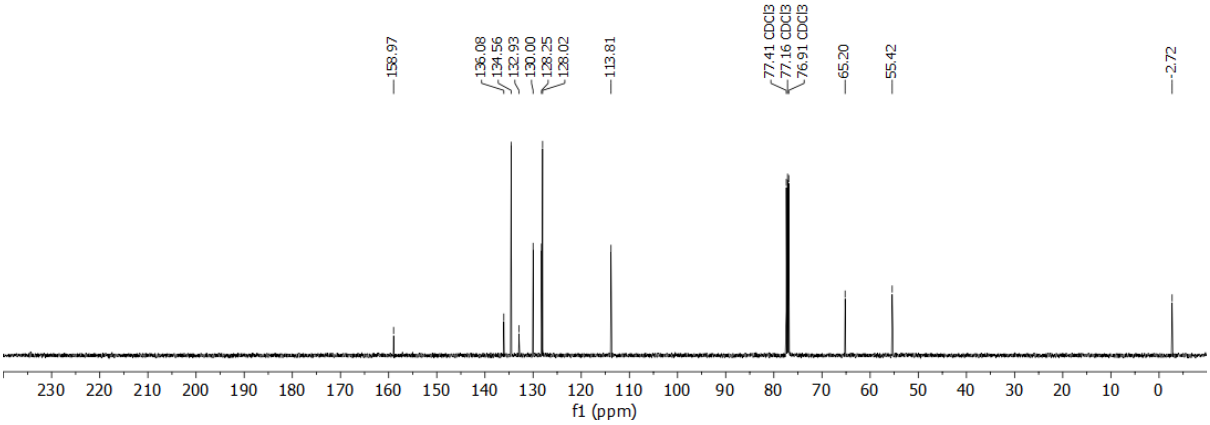

Figure S-20. ^{1}H-NMR (500 MHz, $CDCl_3$) and ^{13}C-NMR (126 MHz, $CDCl_3$) spectra of **8a**. after isolation by flash chromatography.

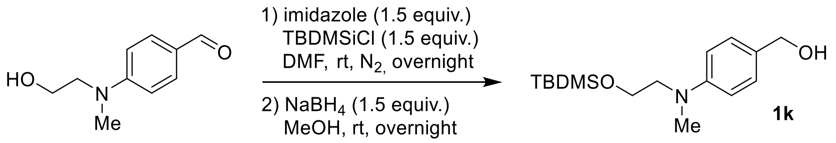

Compound **1k** was synthesized according to literature procedures[17],[18] starting from *N*-methyl-*N*-(2-hydroxyethyl)-4-aminobenzaldehyde and isolated by flash column chromatography on silica gel (20% ethyl acetate/petroleum ether) in 98% yield over two steps (beige oil). **^{1}H NMR** (500 MHz, $CDCl_3$) δ 7.22 (m, 2H), 6.69 (m, 2H), 4.55 (s, 2H), 3.77 (t, J = 6.2 Hz, 2H), 3.47 (t, J = 6.2 Hz, 2H), 2.99 (s, 3H), 0.89 (s, 9H), 0.03 (s, 6H). **^{13}C NMR** (126 MHz, $CDCl_3$) δ 149.1, 128.9, 128.4, 111.9, 77.4, 77.4, 77.2, 76.9, 65.5, 60.6, 55.0, 39.3, 26.0, 18.4, -5.3.

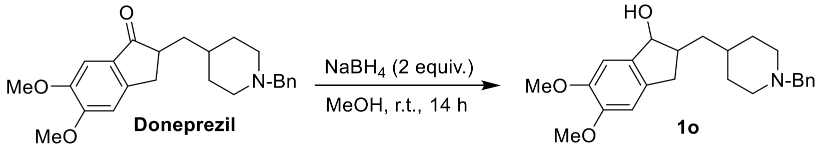

Compound **1o** was synthesized from commercial doneprezil (392 mg, 1 mmol) by stirring with $NaBH_4$ (74 mg, 2 mmol) in MeOH (10 mL) at room temperature for 14 h. After quenching with water and extraction with EtOAc, the collected organic phases were dried over Na_2SO_4, filtrated and concentrated in vacuo. The alcohol **1o** was obtained in 36% after flash chromatography on silica gel (4% MeOH in CH_2Cl_2) as yellow oil. Major diastereomer of **1o**:

^{1}**H NMR** (500 MHz, $CDCl_3$) δ 7.31–7.16 (m, 5H), 6.82 (s, 1H), 6.61 (s, 1H), 4.65 (d, J = 6.3 Hz, 1H), 3.77 (s, 6H), 3.51 (s, 2H), 3.00–2.84 (m, 3H), 2.29 (m, 1H), 2.23–2.12 (m, 1H), 2.06–2.90 (m, 2H), 1.73 – 1.51 (m, 3H), 1.45 – 1.21 (m, 6H). ^{13}**C NMR** (126 MHz, $CDCl_3$) δ 149.4, 148.5, 136.7, 133.6, 129.7, 128.3, 127.5, 107.6, 107.0, 82.2, 63.0, 56.1, 53.6, 48.3, 40.6, 36.3, 34.0, 32.4, 31.5. D.r. 1:3 was assessed by ^{1}H-NMR analysis from signals 4.82 ppm and 4.65 ppm.

All THF- and THP-acetals studied were synthesized following literature procedures.[19],[20] The isolated and characterized products matched data reported in literature.

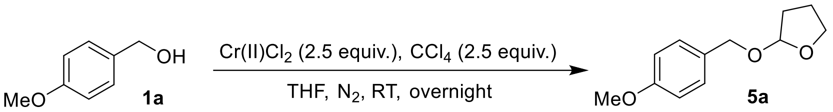

THF acetate **5a** was synthesized from **1a** according to a literature procedure[19] and isolated by flash column chromatography on silica gel (5% Et_2O/pentane) in 23% yield (colourless oil). Experimental data matches literature:[21] ^{1}**H NMR** (500 MHz, $CDCl_3$) δ 7.32 – 7.26 (m, 2H), 6.92 – 6.86 (m, 2H), 5.23 (t, J = 3.2 Hz, 1H), 4.67 (d, J = 11.4 Hz, 1H), 4.43 (d, J = 11.4 Hz, 1H), 4.01–3.88 (m, 2H), 3.82(s, 3H), 2.10–1.99 (m, 1H), 1.98–1.93 (m, 2H), 1.91–1.81(m, 1H). ^{13}**C NMR** (126 MHz, $CDCl_3$) δ 159.2, 130.5, 129.6, 113.9, 102.9, 68.6, 67.1, 55.4, 32.4, 23.6.

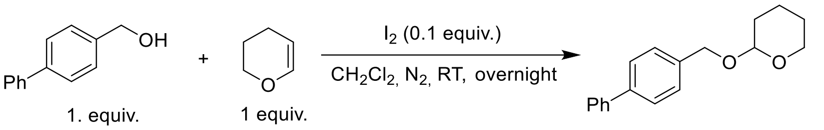

2-([1,1'-Biphenyl]-4-ylmethoxy)tetrahydro-2*H*-pyran was synthesized from 4-phenylbenzyl alcohol and 3,4-dihydro-2*H*-pyran according to a literature procedure[20] and isolated by flash column chromatography on silica gel (5% Et_2O/pentane) in 43% yield (colourless oil). Experimental data matches literature:[22] ^{1}**H NMR** (500 MHz, $CDCl_3$) δ 7.67–7.56 (m, 4H), 7.53–7.41 (m, 4H), 7.37 (m, 1H), 4.87 (d, J = 12.0 Hz, 1H), 4.78 (m, 1H), 4.58 (d, J = 12.0 Hz, 1H), 3.98 (m, 1H), 3.60 (m, 1H), 1.97–1.87 (m, 1H), 1.84–1.75 (m, 1H), 1.75–1.51 (m, 4H). ^{13}**C NMR** (126 MHz, $CDCl_3$) δ 141.1, 140.6, 137.5, 128.8, 128.4, 127.3, 127.3, 127.2, 97.9, 68.7, 62.2, 30.7, 25.6, 19.5

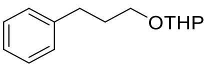

2-(3-Phenylpropoxy)tetrahydro-2*H*-pyran was synthesized from 4-phenylbenzyl alcohol and 3,4-dihydro-2*H*-pyran according to a literature procedure[20] and isolated by flash column chromatography on silica gel (5% Et_2O/pentane) in 43% yield (colourless oil). Experimental data matches literature:[23] ^{1}H-NMR (500 MHz, $CDCl_3$) δ 7.30–7.23 (m, 2H), 7.23–7.15 (m, 3H), 4.58 (m, 1H), 3.87 (m, 1H), 3.78 (m, 1H), 3.50 (m, 1H), 3.41 (m, 1H), 2.77–2.63 (m, 2H), 1.93 (m, 2H), 1.85 (m, 1H), 1.71 (m, 1H), 1.65–1.46 (m, 4H).

10. Unsuccessful and low-yielding alcohol substrates

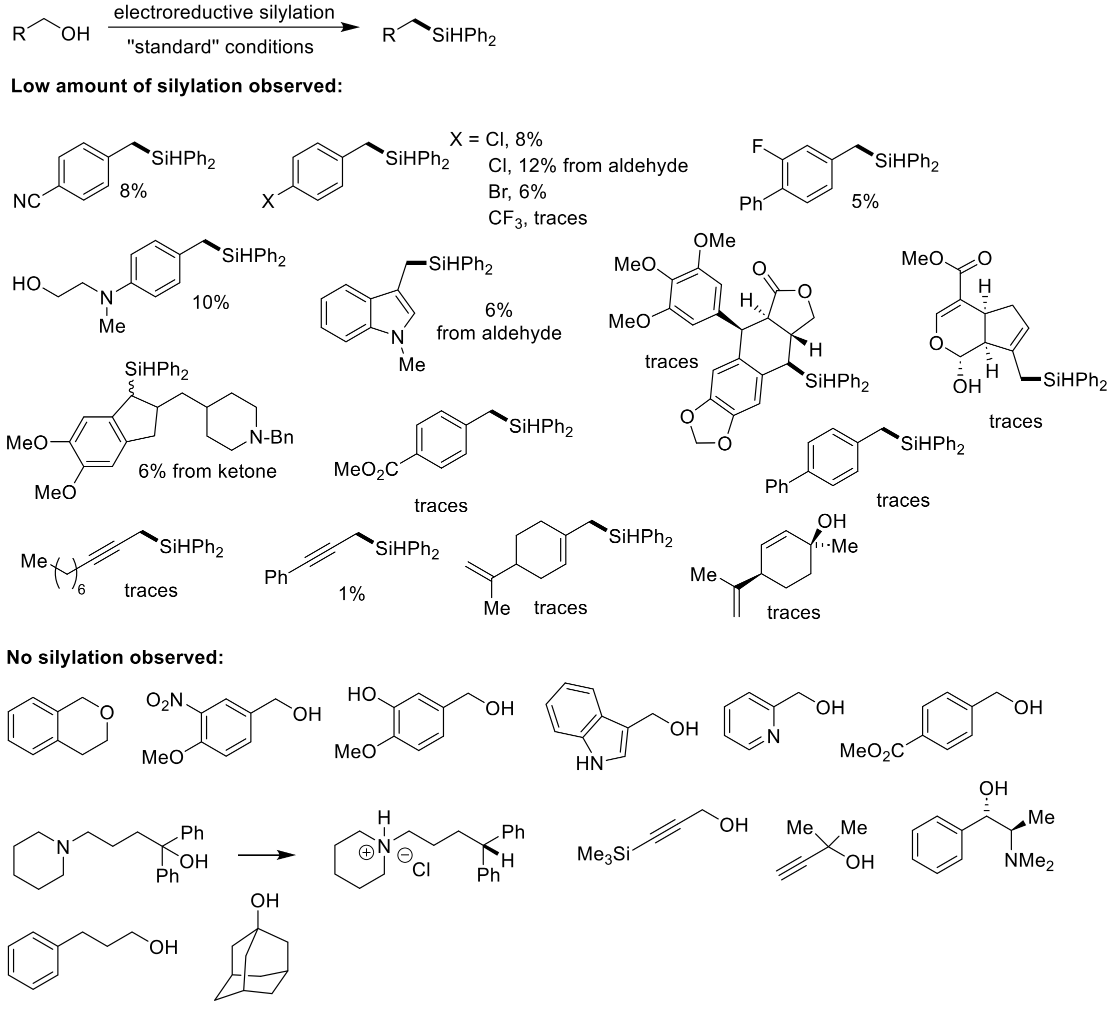

Figure S-21. Unsuccessful and low yielding substrates. Assessed by ^{1}H-NMR with 1,3,5-trimethoxybenzene as internal standard.

11. Cyclic voltammetry

Experimental details

Cyclic voltammetry was performed using a CH Instruments Inc. 650E potentiostat/galvanostat (Austin, TX, USA) at ambient temperature in a 3-electrode cell setup with a glassy carbon (Ø: 1 mm) working electrode (WE), platinum wire counter electrode (CE) and a Ag/$AgNO_3$ reference electrode (RE). The WE, prior to the cyclic voltammogram (CV) measurements, was polished using a damp polishing cloth coated with 0.3 μm alumina polish from Buehler (Lake Bluff, IL, USA) and then thoroughly washed before being rinsed with Milli-Q water and acetone, and then placed in an ultrasonication bath of acetone to remove any remaining alumina or other possible contaminants from the surface of the WE and its PEEK plastic housing. A similar procedure was done for the CE, however, instead of using a polishing cloth, paper towel was coated with the alumina polish and the CE was sandwiched in between two sides of the coated paper towel and pulled through under some pressure several times. The CE was then rinsed with Milli-Q water and acetone before being ultrasonicated in acetone for 10 minutes. After ultrasonication, both the WE and CE were dried using compressed air.

The volume of analyte in the electrochemical cell was 10 ml. The solvent was a mixture of THF and DMF in a ratio of 5:1 and the electrolyte was 0.1 M tetrabutylammonium hexafluorophosphate (Bu_4NPF_6). The solution with only the solvent and electrolyte present is termed the "blank" solution. For the other analytes, 0.1 mmol of the substrate of interest was placed in the blank solution for a concentration of 10 mM. Prior to any CVs being recorded, the analyte solution was purged with Ar for 15 minutes with the Ar gas being pushed through a flask of dry THF before reaching the analyte as to compensate for any evaporation of THF from the analyte. Additionally, the volume level of the analyte in the CV vial was consistently monitored. The exceptions to the above procedures were the CVs done for the reduction of CO_2, for which the blank solution was purged for 15 minutes with CO_2 gas, which was previously pushed through dry THF, instead of Ar gas. The CV measurements of the blank solution and the other analytes were normalized to the potential of the ferrocene redox couple (Fc^+/Fc) by measuring CVs using 0.5 mM of ferrocene added to the blank solution with the 3-electrode cell setup described. The ferrocene redox couple potential was determined by finding the average of the peak potentials from the CVs of both the anodic and cathodic processes. All CV measurements were measured at a sweep rate of 100 mV/s.

The CV measurements were followed by differential pulse voltammetry (DPV) and the settings are shown in Figure S-22.

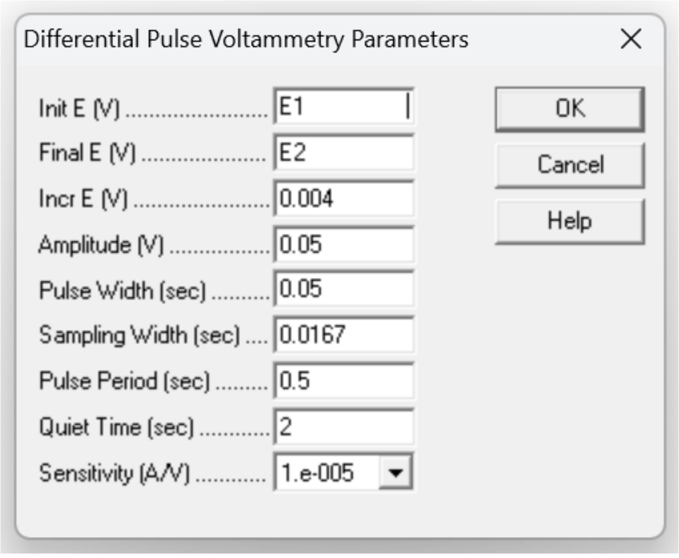

Figure S-22. DPV settings used with a CH Instruments Inc. 650E potentiostat/galvanostat.

Figure S-23 (a) and (b) shows the CVs for the blank solution and for solutions of 10 mM *p*-MeO BnOH (**1a**), 10 mM H_2SiPh_2 (diphenylsilane) and 10 mM *p*-MeO $BnOSiHPh_2$ (**6a**). The overlay of the four CVs shows that the silyl ether (*p*-MeO $BnOSiHPh_2$) requires the least cathodic potential to be reduced, whereas the blank solution requires the most cathodic potential for reduction and therefore a cathodic current to be initiated. Figure S-23 (c) and (d) shows the reduction of **1a** and H_2SiPh_2, and **6a** and H_2SiPh_2, respectively, overlaid with the reduction of common radical traps; 1,1 diphenylethylene (DPE) and 2,2,6,6-tetramethylpiperidine 1-oxyl (TEMPO). Figure S-23 (e) shows the oxidation of **1a** and H_2SiPh_2 overlaid with the oxidation of DPE and TEMPO. Figure S-23 (f) shows the reduction of the blank solution, **1a** and H_2SiPh_2 overlaid with the reduction of the benzyl halides, benzyl chloride (BnCl) and benzyl bromide (BnBr).

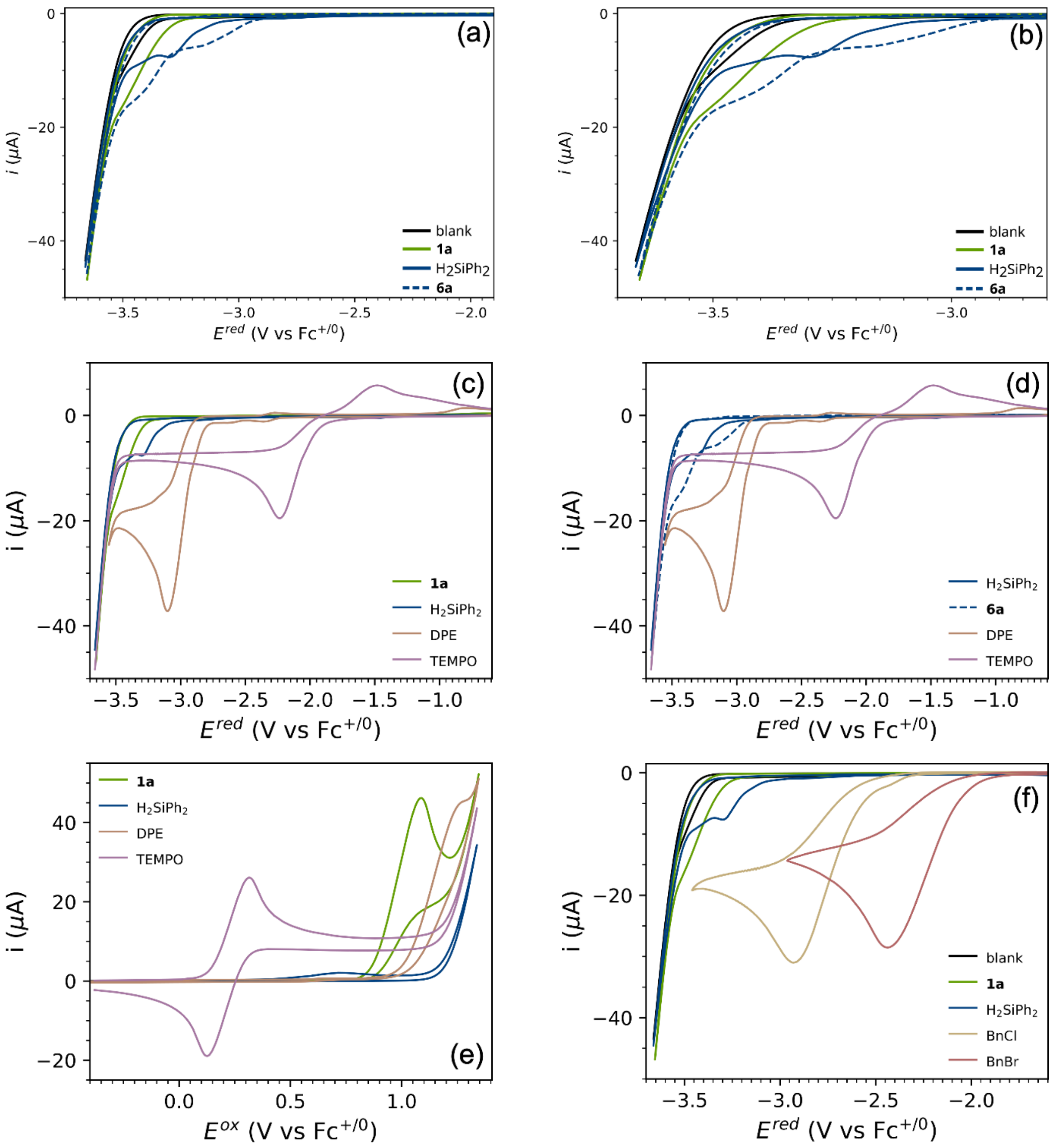

Figure S-23. CVs in the cathodic region (a – d, f) and anodic region (e) for the blank solution and for respective solutions of 10 mM **1a**, H_2SiPh_2, **6a**, DPE, TEMPO, BnCl and BnBr. (a) voltage window of –1.9 to –3.75 V vs. Fc^+/Fc for blank, **1a**, H_2SiPh_2 and **6a**. (b) voltage window of –2.8 to –3.7 V vs. Fc^+/Fc for blank, **1a**, H_2SiPh_2 and **6a**. (c) voltage window of –0.6 to –3.7 V vs. Fc^+/Fc for **1a**, H_2SiPh_2, DPE and TEMPO. (d) voltage window of –0.6 to –3.7 V vs. Fc^+/Fc for H_2SiPh_2, **6a**, DPE and TEMPO. (e) voltage window of –0.4 to +1.4 V vs. Fc^+/Fc for **1a**, H_2SiPh_2, DPE and TEMPO. (f) voltage window of –1.6 to –3.7 V vs. Fc^+/Fc for blank, **1a**, H_2SiPh_2, BnCl and BnBr.

Figure S-24 shows a comparison of the shoulders and peaks of the forward sweep for the CVs for different substrates and the corresponding DPVs of those substrates. The arrows in Figure S-24 (a), (b) and (c) show the correlation of the shoulders and peaks between the DPVs and CVs.

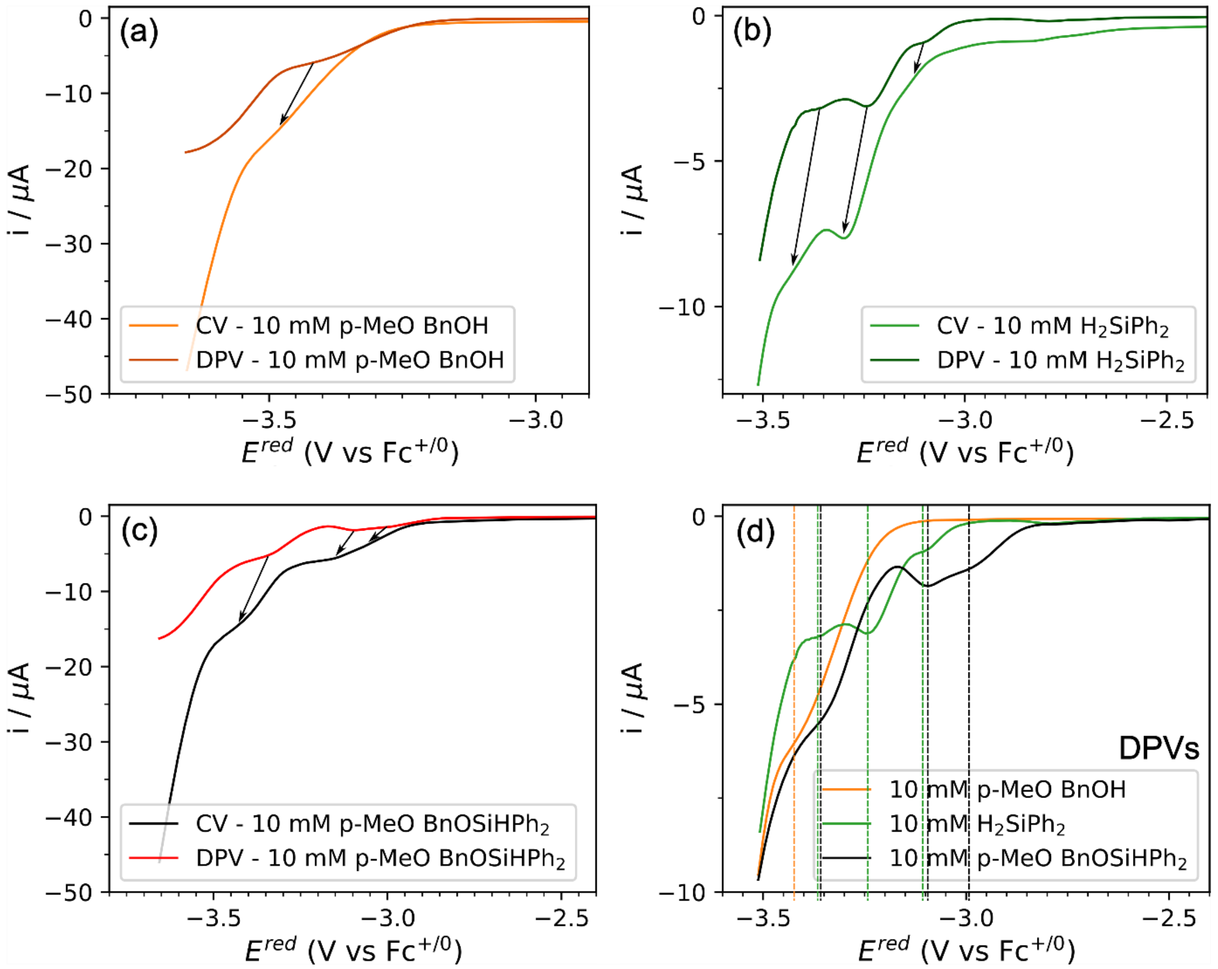

Figure S-24. Comparisons of CVs and DPVs for (a) 10 mM *p*-MeO BnOH (b) 10 mM H_2SiPh_2 and (c) 10 mM *p*-MeO $BnOSiHPh_2$. (d) Overlay of DPVs for 10 mM *p*-MeO BnOH, 10 mM H_2SiPh_2, and 10 mM *p*-MeO $BnOSiHPh_2$.

Figure S-24 (a) displays the CVs and DPVs for 10 mM *p*-MeO BnOH (**1a**). The correlation between the CV and DPV shown by the arrow indicates that the slight shoulder of the CV between –3.5 and –3.3 V vs Fc^+/Fc is represented as a shallow peak in the corresponding CV. (b) displays the CVs and DPVs for 10 mM H_2SiPh_2, from which two slight shoulders and one definitive peak in the CV between –3.5 and –3.0 V vs Fc^+/Fc are shown to be correlated with three definitive peaks in the DPV. (c) displays the CVs and DPVs for 10 mM *p*-MeO $BnOSiHPh_2$ (**6a**), from which three slight shoulders in the CV between –3.5 and –2.8 V vs Fc^+/Fc are shown to be correlated with three shallow peaks in the DPV. (d) displays the overlay of DPVs of 10 mM *p*-MeO BnOH (**1a**), 10 mM H_2SiPh_2 and 10 mM *p*-MeO $BnOSiHPh_2$ (**6a**) with the peak positions signified with vertical dashed lines. It is shown that *p*-MeO $BnOSiHPh_2$ (**6a**) is reduced prior to H_2SiPh_2 and that H_2SiPh_2 is reduced prior to *p*-MeO BnOH (**1a**). These findings from the DPVs correlate with the order of reduction shown by the overlay of CVs in Figure S-24.

Figure S-25 shows overlays of CVs for the reduction of CO_2 with the reduction of a benzylic alcohol with an electron donating substituent in the *para* position (*p*-MeO BnOH), a benzylic alcohol with an electron withdrawing substituent in the *para* position (*p*-CO_2Me BnOH), and the blank solvent and supporting electrolyte mixture.

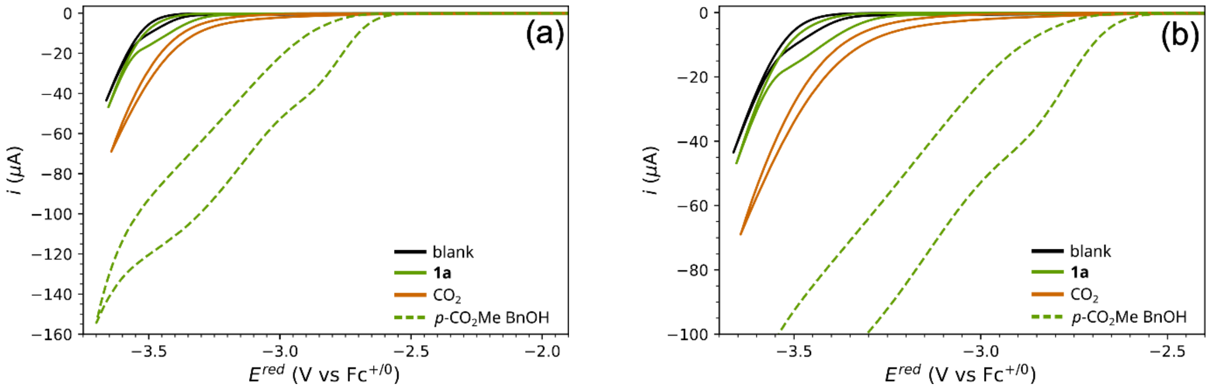

Figure S-25. CVs in the cathodic region for the blank solution and for solutions of 10 mM *p*-MeO BnOH (**1a**), saturated CO_2 and 10 mM *p*-CO_2Me BnOH. (a) voltage window from -1.9 to -3.75 V vs. Fc^+/Fc. (b) voltage window from -2.4 to -3.7 V vs. Fc^+/Fc.

The CVs in Figure S-25 show that the reduction of CO_2 is preferential to the reduction of the blank solution and the 10 mM *p*-MeO BnOH (**1a**) solution, having a less cathodic WE potential at equivalent cathodic currents, i.e. more than 100 mV less for current levels between 10 and 45 µA. Figure S-25 also demonstrates that the benzylic alcohol with the electron withdrawing subsituent (*p*-CO_2Me BnOH) is reduced at a much less negative potential than that of *p*-MeO BnOH and CO_2. For example, the WE potential for the CV of *p*-CO_2Me BnOH is more than 600 mV less cathodic for current levels between 10 and 45 mA. This indicates that *p*-CO_2Me BnOH in an electrosynthetic setting would most likely be reduced in solution prior to *p*-MeO BnOH, CO_2 and H_2SiPh_2.

12. DFT calculations

Computational Details

Computations were performed with ORCA 5.0.4.[24–26] All optimized ground state structures were verified as true minima by the absence of negative eigenvalues in the harmonic vibrational frequency analysis. Tighter than normal conversion criteria were used for geometry optimization (*tightopt*) and SCF (*tightscf*). The geometry optimizations were performed using the r²SCAN-3c composite method.[27–31] Single point calculations were performed using the ωB97X hybrid functional.[32] The ZORA-def2-TZVPP basis set was used.[33] Scalar relativistic effects were described using the zeroth order regular approximation (ZORA)[34] and corrections for dispersion interactions were carried out using the D4 correction.[25,26] Computations were sped up using the RI*J*COSX approximation and the auxiliary basis set SARC/*J*.[35–39] Solvent effects were described using the SMD solvation model.[40] Gibbs free energy values are corrected by 7.91 k*J* mol^{-1} (0.003013 Eh) to convert from the gas standard state to the solution standard state.[41] Orbitals were visualized using IboView.[42]

Reduction potential of benzyl alcohols and acetals

Redox potentials were computed relative to ferrocene from the Gibbs energy of reaction for the oxidation reaction according to convention. As reference, a redox potential for the ferrocenium/ferrocene couple $E_{Fc^{+}/0}$ of +5 V[43] was assumed.

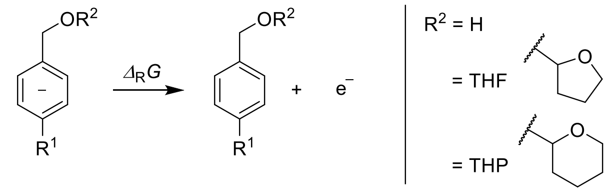

$$E = \frac{\Delta_R G}{F} - E_{Fc^{+}/Fc}$$

Table S-10. Gibbs energy of reaction $\Delta_R G$ and redox potentials for the reduction of benzyl alcohols and corresponding THF and THP acetals (ZORA-ωB97X(SMD=THF)-D4/def2-TVZPP//r²SCAN-3c).

R^1, R^2	$\Delta_R G$/ kJ mol^{-1}	E/ V
R^1 = OMe, R^2 = H	+94.0	−4.03
R^1 = OMe, R^2 = THF	+84.9	−4.12
R^1 = OMe, R^2 = THP	+135.1	−3.60
R^1 = Me, R^2 = H	+85.7	−4.11
R^1 = H, R^2 = H	+102.1	−3.94
R^1 = H, R^2 = THF	+130.4	−3.65
R^1 = H, R^2 = THP	+134.6	−4.61
R^1 = Ph, R^2 = H	+156.2	−3.38
R^1 = COOMe, R^2 = H	+182.9	−3.10
R^1 = COOMe, R^2 = THF	+210.8	−2.81
R^1 = COOMe, R^2 = THP	+205.7	−2.87
R^1 = F, R^2 = H	+113.7	−3.82
R^1 = F, R^2 = THF	+110.6	−3.85
R^1 = F, R^2 = THP	+110.8	−3.85
R^1 = CN, R^2 = H	+191.9	−3.01
R^1 = CN, R^2 = THF	+181.5	−3.12
R^1 = CN, R^2 = THP	+184.6	−3.09

The LUMO of *p*-methoxybenzylalcohol is not notably delocalized on the methoxy group, unlike the LUMO for *p*-hydroxymethybenzoate (Figure S-26), meaning that the electron-donating mesomeric effect of the methoxy group does not significantly overcompensate the electron-withdrawing inductive effect of the electronegative oxygen atom.

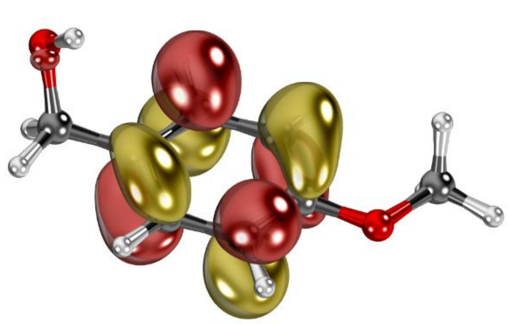

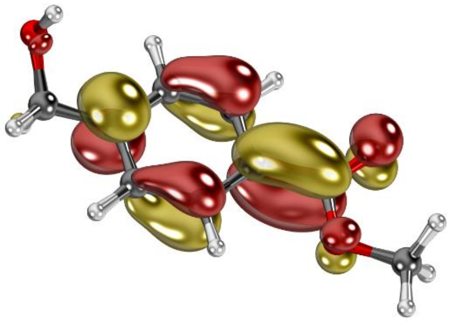

Figure S-26. LUMO for *p*-methoxybenzylalcohol (left) and methyl *p*-hydroxymethylbenzoate (right) (60% isovalue, ZORA-ωB97X(SMD=THF)-D4/def2-TVZPP//r²SCAN-3c).

Reduction potential of other compounds

Redox potentials were computed relative to ferrocene from the Gibbs energy of reaction for the oxidation reaction according to convention. As reference, a redox potential for the ferrocenium/ferrocene couple $E_{Fc^{+}/0}$ of +5 V[43] was assumed.

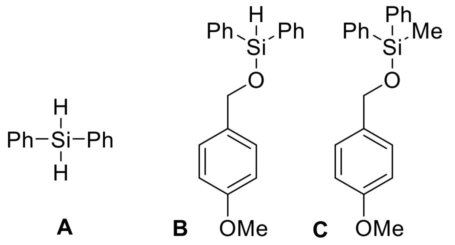

$$E = \frac{\Delta_R G}{F} - E_{Fc^+/Fc}$$

Table S-11. Gibbs energy of reaction $\Delta_R G$ and redox potentials for the reduction of other compounds (ZORA-ωB97X(SMD=THF)-D4/def2-TVZPP//r²SCAN-3c).

Compound	$\Delta_R G$/ kJ mol^{-1}	E/ V
**A**	+112.0	−3.84
**B**	+115.8	−3.80
**C**	+108.9	−3.87

Basicity of benzyl anions

The basicity of benzyl anions was determined as the acidity of the corresponding toluene derivative. All values were obtained from the equilibrium reaction with toluene, for which a literature pK_a value of 40.9 was used.[44] As temperature, 273.15 K was used.

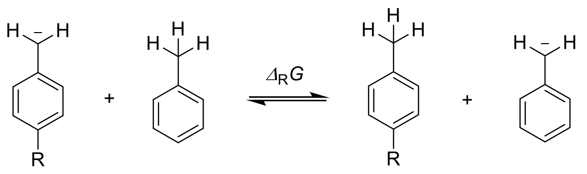

$$pK_a = log_{10}\left(e^{-\frac{\Delta_R G}{RT}}\right) + pK_{a,toluene}$$

Table S-12. Gibbs energy of reaction $\Delta_R G$ and the resulting pK_a value for the equilibrium acid base reaction between benzylic anions and toluene (ZORA-ωB97X(SMD=THF)-D4/def2-TVZPP//r²SCAN-3c).

	**R = OMe**	**R = Me**	**R = F**	**R = H**	**R = Ph**	**R = COOMe**	**R = CN**
**$\Delta_R G$/ k*J* mol^{-1}**	−24.6	−12.4	−6.4	0	+7.9	+38.3	+53.3
**pK_a**	45.6	43.3	42.1	40.9[11]	39.4	33.6	30.7

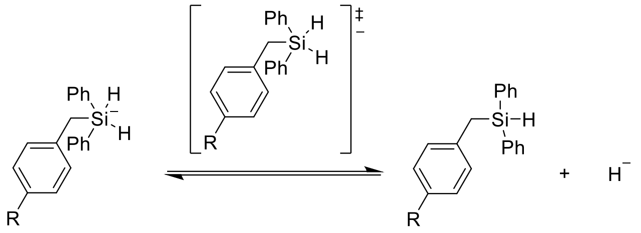

Table S-13. Gibbs energy of reaction $\Delta_R G$ and relative Gibbs energy for the transition state $\Delta_R G^{\ddagger}$ for the dissociation of hydride ions from pentavalent silicon species (ZORA-ωB97X(SMD=THF)-D4/def2-TVZPP//r²SCAN-3c).

**R**	**$\Delta_R G$/ k*J* mol^{-1}**	**$\Delta_R G^{\ddagger}$/ k*J* mol^{-1}**
H	−11.9	+14.0
OMe	−16.4	+4.8

Reaction with CO_2

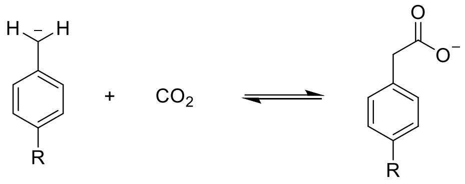

Table S-14. Gibbs energy of reaction $\Delta_R G$ for the reaction of benzylic anions with carbon dioxide (ZORA-ωB97X(SMD=THF)-D4/def2-TVZPP//r²SCAN-3c).

**R**	**OMe**	**Me**	**F**	**H**	**Ph**	**CF_3**	**COOMe**	**NO_2**	**CN**
**$\Delta_R G$/ k*J* mol^{-1}**	−138.9	−128.1	−130.2	−118.0	−109.0	−90.5	−77.8	−47.6	−68.9

Reaction barriers are very small and the reactions are all notably exergonic. As such, it may be feasible to generally assume a diffusion-controlled reaction barrier of 14.4 k*J* mol^{-1} (see below) in all cases. The geometry scans for the formed C–C bond are shown below and only for the case of *p*-NC-Bn^{-} there is a transition state visible if the geometry scan was conducted using

the SMD solvation model. Even then, it is only a few kJ mol^{-1}, indicating that diffusion is probably the more important factor.

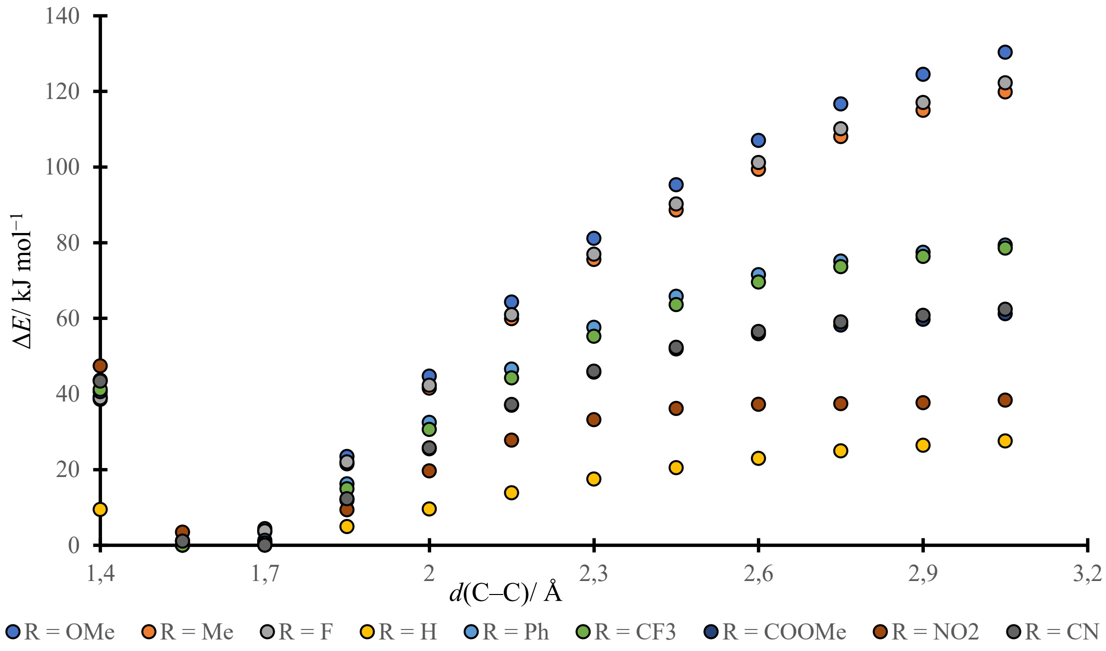

Figure S-27. Geometry scan along the C–C bond for the reaction of CO_2 with benzyl anions. (r²SCAN-3c). Note the lack of visible transition state.

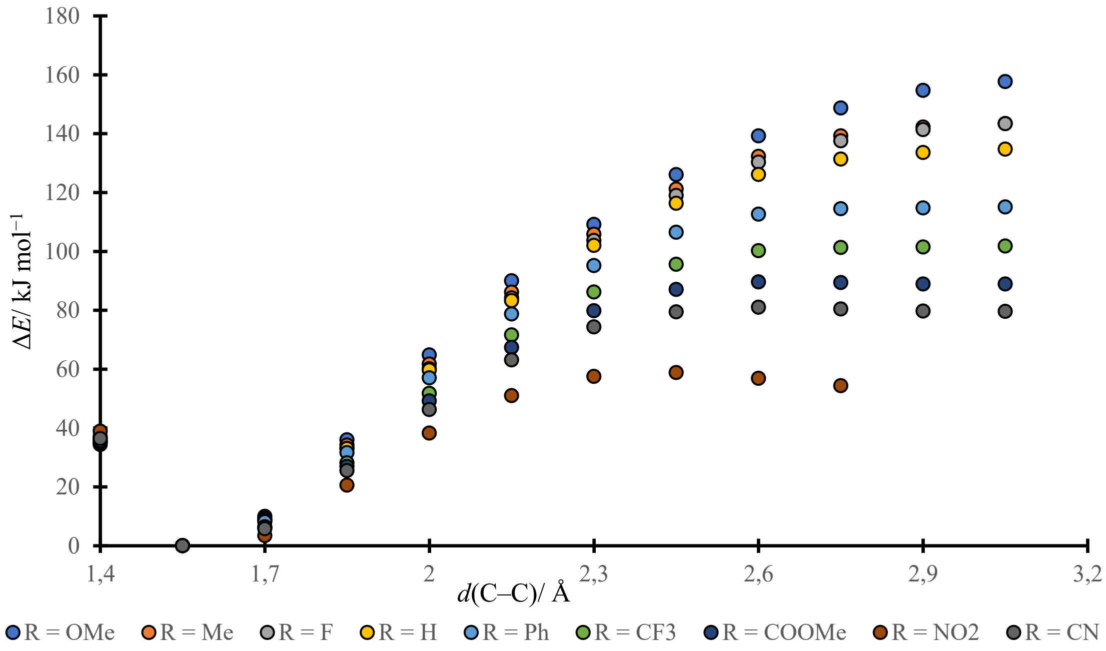

Figure S-28. Geometry scan along the C–C bond for the reaction of CO_2 with benzyl anions. (r²SCAN-3c(SMD = THF)). Note (with the exception of the NO_2 compound) the lack of visible transition state.

Thermodynamic stability of anionic cross-coupled C–Si intermediates and reaction barriers for their formation

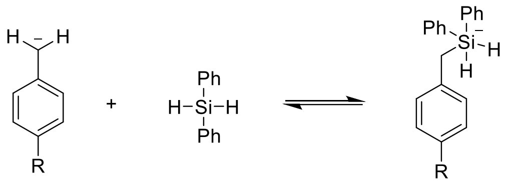

Reaction barriers are very low for the formation of the anionic C–Si cross-coupling intermediate species. Most transition states could only be localized using the climbing image nudged elastic band method (CI-NEB)[45] between the ground state of the pentavalent species and the endpoint of a geometry scan along the reaction coordinate. The energy of those transition states may be very close to the higher ground state of the reaction step, if not even below. In those cases, the reaction barrier for the reaction of two molecules can be assumed to be diffusion controlled and the method of McMullin *et al.*[46] can be used to estimate the reaction barrier from the Eyring equation.[47,48]

$$k = \frac{\kappa k_B T}{h} e^{-\frac{\Delta G^{\ddagger}}{RT}}$$

$$\Delta G^{\ddagger} = RTln\left(\frac{\kappa k_B T}{hk}\right)$$

With the gas constant R = 8.3145 J mol^{-1} K^{-1}, Boltzmann constant k_B = 1.3806×10^{-23} J K^{-1}, Planck constant h = 6.6261×10^{-34} J s,[49] estimated transmission constant $\kappa \approx 1$ and rate constant $k \approx 10^{10}$ M^{-1} s^{-1},[46] a minimum reaction barrier of $\Delta G^{\ddagger} \approx$ 14.4 kJ mol^{-1} above the higher endpoint of the geometry scan can be estimated at T = 273.15 K.

Table S-15. Relative thermodynamic stability of pentavalent silicon compounds (ZORA-ωB97X(SMD=THF)-D4/def2-TVZPP//r²SCAN-3c). For the relative Gibbs energies of the transition states, if the transition state was low enough to assume diffusion control, the computed barrier is given in parenthesis and the energy barrier from the Eyring equation is given instead.

**R**	$\Delta_\mathrm{R}G$/ kJ mol^{-1}	$\Delta_\mathrm{R}G^{\ddagger}$/ kJ mol^{-1}
MeO	−19.3	+29.7
Me	−11.1	+27.3
F	−7.4	+43.9
H	−2.9	+14.4 (+0.0)*
Ph	+9.3	+32.3
CF_3	+25.8	+40.2 (+38.8)*
COOMe	+32.1	+46.5 (+38.7)*
NO_2	+36.6	not found
CN	+44.3	+58.7 (+39.8)

* Corrected to a minimum using the Eyring equation as described above.

For the nitro complex, no transition state could be localized. This can be seen in the geometry scans along the Si–C bond.

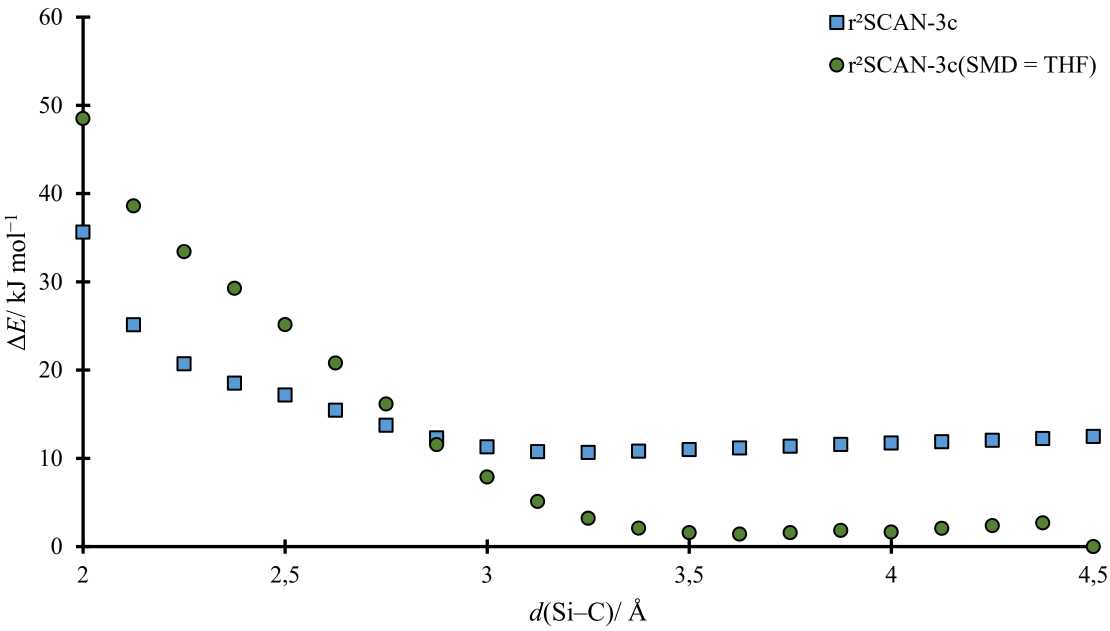

Figure S-29. Geometry scan along the Si–C bond for the reaction of Ph_2H_2Si with a *p*-nitrobenzyl anion. Blue: r²SCAN-3c; green: r²SCAN-3c(SMD = THF). Note the lack of visible transition state.

Energies

Table S-16. Energies and Gibbs energies of the computed compounds (r^{2}SCAN-3c or ZORA-ωB97X(SMD=THF)-D4/def2-TVZPP//r^{2}SCAN-3c)

Compound	E(r^{2}SCAN-3c)/ Eh	G(r^{2}SCAN-3c)/ Eh	E(ωB97X)/ Eh	G(ωB97X)/ Eh	G(ωB97X)/ kJ mol^{-1}
H^-	−0.439597	−0.446584	−0.643036	−0.650023	−1706.64
F^-	−99.829167	−99.840313	−100.151706	−100.162851	−262977.77
BnF	−370.724159	−370.632133	−371.414934	−371.322908	−974909.06
CO_2	−188.570060	−188.571809	−188.901090	−188.902839	−495964.79
$CO_2^{\bullet-}$	−188.458106	−188.463376	−188.866154	−188.871424	−495882.31
MeOBnOH	−461.202532	−461.069038	−462.055729	−461.922235	−1212777.78
MeOBnOTHF	−692.402903	−692.178607	−693.694343	−693.470047	−1820707.04
MeOBnOTHP	−731.708850	−731.456172	−733.078378	−732.825701	−1924035.39
MeOBnOSiPh$_2$H	−1213.923964	−1213.624605	−1216.638709	−1216.339349	−3193501.46
MeOBnOSiPh$_2$Me	−1253.244479	−1252.917852	−1256.038453	−1255.711826	−3296873.98
MeBnOH	−385.998862	−385.870434	−386.718276	−386.589848	−1014992.44
FBnOH	−445.940399	−445.845870	−446.764527	−446.669998	−1172733.00
FBnOTHF	−677.145520	−676.959659	−678.405782	−678.219922	−1780667.80
FBnOTHP	−716.442804	−716.228916	−717.781371	−717.567483	−1883974.90
BnOH	−346.697104	−346.592652	−347.337166	−347.232714	−911660.21
BnOTHF	−577.897489	−577.702876	−578.975712	−578.781099	−1519590.97
BnOTHP	−617.203414	−616.980280	−618.359661	−618.136526	−1622918.72
PhBnOH	−577.700053	−577.521154	−578.749828	−578.570928	−1519039.16
MeOOCBnOH	−574.549907	−574.408843	−575.601625	−575.460561	−1510872.89
MeOOCBnOTHF	−805.733727	−805.501131	−807.227499	−806.994904	−2118766.78
MeOOCBnOTHP	−845.039764	−844.780851	−846.611460	−846.352547	−2222100.35
NCCBnOH	−438.924135	−438.823674	−439.719067	−439.618606	−1154219.56
NCBnOTHF	−670.131883	−669.939041	−671.361680	−671.168839	−1762155.17
NCBnOTHP	−709.426397	−709.207668	−710.736433	−710.517704	−1865465.69
MeOBnOH$^{\bullet-}$	−461.163142	−461.038421	−462.082754	−461.958033	−1212871.77
MeOBnOTHF$^{\bullet-}$	−692.365659	−692.149936	−693.718110	−693.502387	−1820791.94
MeOBnOTHP$^{\bullet-}$	−731.696229	−731.456883	−733.116498	−732.877151	−1924170.47
MeOBnOSiPh$_2$H$^{\bullet-}$	−1213.922109	−1213.629081	−1216.676467	−1216.383438	−3193617.22
MeOBnOSiPh$_2$Me$^{\bullet-}$	−1253.240182	−1252.920035	−1256.073467	−1255.753320	−3296982.92
MeBnOH$^{\bullet-}$	−385.956604	−385.836805	−386.742303	−386.622503	−1015078.18
FBnOH$^{\bullet-}$	−445.913215	−445.826001	−446.800499	−446.713285	−1172846.65
FBnOTHF$^{\bullet-}$	−677.115272	−676.938516	−678.438801	−678.262045	−1780778.39
FBnOTHP$^{\bullet-}$	−716.412417	−716.207276	−717.814841	−717.609701	−1884085.75
BnOH$^{\bullet-}$	−346.661692	−346.566849	−347.366450	−347.271607	−911762.32
BnOTHF$^{\bullet-}$	−577.886170	−577.704201	−579.012749	−578.830780	−1519721.40
BnOTHP$^{\bullet-}$	−617.192007	−616.981730	−618.398051	−618.187775	−1623053.27
PhBnOH$^{\bullet-}$	−577.700053	−577.521154	−578.749828	−578.570928	−1519039.16
MeOOCBnOH$^{\bullet-}$	−574.549907	−574.408843	−575.601625	−575.460561	−1510872.89
MeOOCBnOTHF$^{\bullet-}$	−805.757162	−805.530687	−807.301679	−807.075204	−2118977.61
MeOOCBnOTHP$^{\bullet-}$	−845.061569	−844.807420	−846.685056	−846.430907	−2222306.09
NCBnOH$^{\bullet-}$	−438.938049	−438.843881	−439.785867	−439.691699	−1154411.46
NCBnOTHF$^{\bullet-}$	−670.141349	−669.955048	−671.424250	−671.237949	−1762336.62
NCBnOTHP$^{\bullet-}$	−709.442822	−709.228143	−710.802692	−710.588014	−1865650.29
MeOBn$^-$	−385.361752	−385.248432	−386.141134	−386.027813	−1013516.82
MeBn$^-$	−310.166176	−310.056306	−310.810854	−310.700984	−815746.07
FBn$^-$	−370.111456	−370.035881	−370.858422	−370.782847	−973491.13
Bn$^-$	−270.865232	−270.779891	−271.433002	−271.347661	−712423.84
PhBn$^-$	−501.889134	−501.729021	−502.849044	−502.688931	−1319810.82
F$_3$CBn$^-$	−607.950258	−607.865218	−609.134920	−609.049881	−1599061.72
MeOOCBn$^-$	−498.751784	−498.628799	−499.713620	−499.590635	−1311676.24
O$_2$NBn$^-$	−475.419171	−475.332986	−476.310985	−476.224800	−1250329.19
NCBn$^-$	−363.133056	−363.050215	−363.837370	−363.754530	−955038.27
MeOBnH	−385.991884	−385.863332	−386.708060	−386.579509	−1014965.30
MeBnH	−310.791044	−310.666987	−311.372115	−311.248058	−817182.42
FBnH	−370.732454	−370.641731	−371.418352	−371.327630	−974921.46
BnH	−271.487227	−271.387115	−271.990105	−271.889993	−713847.74
PhBnH	−502.490093	−502.315647	−503.402709	−503.228263	−1321226.84

MeOOCBnH	−499.340754	−499.204121	−500.255008	−500.118376	−1313061.82
NCBnH	−363.715315	−363.619218	−364.372642	−364.276546	−956408.82
MeOBnBn	−656.289197	−656.057703	−657.495135	−657.263641	−1725647.04
MeBnBn	−581.089451	−580.862277	−582.159973	−581.932798	−1527865.76
FBnBn	−641.027540	−640.835318	−642.203597	−642.011375	−1685602.19
BnBn	−541.784309	−541.582228	−542.777085	−542.575004	−1424531.79
PhBnBn	−772.788612	−772.510698	−774.190712	−773.912798	−2031909.64
MeOOCBnBn	−769.637679	−769.398488	−771.041863	−770.802672	−2023744.00
NCBnBn	−634.012096	−633.813832	−635.159296	−634.961032	−1667091.49
Ph$_2$H$_2$Si	−753.858567	−753.695693	−755.731828	−755.568954	−1983747.84
Ph$_2$H$_2$Si$^{\bullet-}$	−753.846005	−753.688997	−755.768616	−755.611608	−1983859.83
Ph$_2$H$_2$(MeOBn)Si$^-$	−1139.265222	−1138.966504	−1141.902838	−1141.604120	−2997283.97
Ph$_2$H$_2$(MeBn)Si$^-$	−1064.065641	−1063.771678	−1066.568122	−1066.274159	−2799505.00
Ph$_2$H$_2$(FBn)Si$^-$	−1124.010250	−1123.750371	−1126.614484	−1126.354605	−2957246.33
Ph$_2$H$_2$BnSi$^-$	−1024.763174	−1024.493830	−1027.187046	−1026.917703	−2696174.54
Ph$_2$H$_2$(PhBn)Si$^-$	−1255.771670	−1255.427093	−1258.598931	−1258.254354	−3303549.39
Ph$_2$H$_2$(F3CBn)Si$^-$	−1361.833712	−1361.563197	−1364.879513	−1364.608999	−3582783.73
Ph$_2$H$_2$(MeOOCBn)Si$^-$	−1252.629202	−1252.323395	−1255.453174	−1255.147367	−3295391.99
Ph$_2$H$_2$(O2NBn)Si$^-$	−1229.292220	−1229.023250	−1232.048784	−1231.779814	−3234040.44
Ph$_2$H$_2$(NCBn)Si$^-$	−1117.009613	−1116.742751	−1119.573457	−1119.306595	−2938741.77
Ph$_2$H(MeOBn)Si	−1138.677975	−1138.382382	−1141.255945	−1140.960352	−2995593.75
Ph$_2$HBnSi	−1024.173382	−1023.907430	−1026.538154	−1026.272202	−2694479.78
MeOBnCO$_2^-$	−573.997647	−573.869080	−575.112122	−574.983555	−1509620.51
MeBnCO$_2^-$	−498.797385	−498.673398	−499.776598	−499.652612	−1311838.96
FBnCO$_2^-$	−558.743618	−558.655043	−559.823838	−559.735263	−1469586.08
BnCO$_2^-$	−459.494583	−459.395223	−460.394813	−460.295453	−1208506.66
PhBnCO$_2^-$	−690.501701	−690.327697	−691.807298	−691.633294	−1815884.64
F3CBnCO$_2^-$	−796.563201	−796.464767	−798.085642	−797.987208	−2095117.06
MeOOCBnCO$_2^-$	−687.356992	−687.220808	−688.659271	−688.523087	−1807718.78
O2NBnCO$_2^-$	−664.013975	−663.915498	−665.244258	−665.145782	−1746341.62
NCBnCO$_2^-$	−551.738830	−551.643247	−552.779197	−552.683614	−1451071.97

Table S-17. Energies, Gibbs energies and imaginary frequencies of the computed transition states (r²SCAN-3c or ZORA-ωB97X(SMD=THF)-D4/def2-TVZPP//r²SCAN-3c)

Compound	ν_{img}/ cm^{-1}	*E*(r²SCAN-3c)/ Eh	*G*(r²SCAN-3c)/ Eh	*E*(ωB97X)/ Eh	*G*(ωB97X)/ Eh	*G*(ωB97X)/ kJ mol^{-1}
TS(MeOBn--Bn--F$^-$)	441i	−756.099765	−755.874005	−757.545355	−757.319595	−1988344.16
TS(MeBn--Bn--F$^-$)	449i	−680.901451	−680.680124	−682.212806	−681.991479	−1790570.03
TS(FBn--Bn--F$^-$)	448i	−740.846580	−740.659155	−742.260226	−742.072801	−1948313.67
TS(Bn--Bn--F$^-$)	454i	−641.599379	−641.402606	−642.833100	−642.636326	−1687243.00
TS(PhBn--Bn--F$^-$)	490i	−872.609928	−872.337954	−874.249576	−873.977602	−2294629.99
TS(MeOOCBn--Bn--F$^-$)	502i	−869.468048	−869.233538	−871.108033	−870.873522	−2286480.22
TS(NCBn--Bn--F$^-$)	500i	−733.849259	−733.655333	−735.229541	−735.035614	−1929837.52
TS(MeOBn--SiH$_2$Ph$_2^-$)	35i	−1139.238485	−1138.942519	−1141.881414	−1141.585448	−2997234.94
TS(MeBn--SiH$_2$Ph$_2^-$)	28i	−1064.041664	−1063.750420	−1066.550783	−1066.259540	−2799466.62
TS(FBn--SiH$_2$Ph$_2^-$)	19i	−1123.989000	−1123.729010	−1126.595054	−1126.335065	−2957195.03
TS(Bn--SiH$_2$Ph$_2^-$)	44i	−1024.745818	−1024.478380	−1027.173172	−1026.905734	−2696143.12
TS(PhBn--SiH$_2$Ph$_2^-$)	68i	−1255.765850	−1255.421730	−1258.589687	−1258.245567	−3303526.33
TS(F$_3$CBn--SiH$_2$Ph$_2^-$)	61i	−1361.828844	−1361.558549	−1364.874368	−1364.604073	−3582770.80
TS(MeOOCBn--SiH$_2$Ph$_2^-$)	18i	−1252.627628	−1252.320542	−1255.451920	−1255.144835	−3295385.35
TS(NCBn--SiH$_2$Ph$_2^-$)	22i	−1117.004774	−1116.739213	−1119.573857	−1119.308296	−2938746.23
TS(Ph$_2$H(MeOBn)Si--H$^-$)	317i	−1139.211620	−1138.916409	−1141.897505	−1141.602294	−2997279.17
TS(Ph$_2$HBnSi--H$^-$)	301i	−1024.704926	−1024.439151	−1027.178141	−1026.912366	−2696160.53

XYZ Coordinates

1
H−
H 0.000000 0.000000 0.000000

1
F−
F 0.000000 0.000000 0.000000

15
BnF

C	-4.257024	1.761967	0.461967
C	-3.798943	2.662321	1.420232
C	-3.377937	0.863681	-0.140416
C	-2.031941	0.869390	0.234616
C	-1.575437	1.759335	1.197877
C	-2.458666	2.660354	1.790267
H	-5.307409	1.756090	0.180998
H	-4.490918	3.360509	1.882122
H	-0.528150	1.755885	1.485811
H	-2.100070	3.359358	2.540268
H	-1.345348	0.165082	-0.228596
C	-3.863263	-0.115534	-1.165177
F	-3.757414	-1.428325	-0.668419
H	-3.258190	-0.075220	-2.079979
H	-4.915745	0.059548	-1.416858

3
CO2

O	-1.442279	1.444026	0.000000
C	-0.285231	1.322448	0.000000
O	0.871842	1.201121	0.000000

3
CO2•−

O	-1.540587	1.454354	0.000001
C	-0.285233	1.322430	-0.000002
O	0.970152	1.190810	0.000001

20
MeOBnOH

C	-4.927377	3.283022	0.017260
C	-3.717858	3.897271	-0.306557
C	-3.610399	5.280006	-0.412173
C	-4.738456	6.075579	-0.187655
C	-5.958181	5.475854	0.135014
C	-6.044125	4.095529	0.230917
H	-6.823622	6.109297	0.302160
H	-2.841436	3.279674	-0.487014
H	-2.652467	5.720863	-0.662691
H	-6.999384	3.638585	0.480125
C	-5.024139	1.783247	0.096290
O	-4.679165	1.140985	-1.138891
H	-6.036216	1.493833	0.421138
H	-4.314653	1.384009	0.830425
H	-5.174373	1.585311	-1.837229
O	-4.750828	7.437141	-0.260317
C	-3.530532	8.092759	-0.588843
H	-3.758076	9.159989	-0.594544
H	-3.169002	7.792904	-1.581743
H	-2.752908	7.890955	0.160123

31
MeOBnOTHF

C	-2.322799	-2.971633	-0.733751
C	-1.107228	-1.657949	0.869738
C	-0.925275	-1.084679	-0.533341
O	-1.921818	-1.732118	-1.338176
H	0.082074	-1.307312	-0.923249
H	-1.091133	-0.004132	-0.594557
O	-2.227547	-4.036566	-1.643381
C	-1.487976	-3.102871	0.551180
H	-3.399199	-2.914549	-0.522384
C	-0.917898	-4.296456	-2.176862
C	-1.060892	-4.781444	-3.589449
H	-0.325875	-3.370383	-2.162623
H	-0.400608	-5.052562	-1.567438
C	-0.575807	-6.020150	-3.986782
C	-0.690074	-6.458142	-5.307569
C	-1.314343	-5.636651	-6.244796
C	-1.815373	-4.387896	-5.854979
C	-1.686850	-3.970511	-4.544194
H	-0.097464	-6.669469	-3.256996
H	-0.300413	-7.431941	-5.580033
H	-2.300380	-3.766032	-6.601018
H	-2.082788	-3.005519	-4.238258
O	-1.490342	-5.954556	-7.560585
H	-1.929400	-1.144605	1.381784
H	-0.207228	-1.568460	1.485615
H	-0.592456	-3.701441	0.350456
H	-2.053718	-3.599244	1.343711
C	-1.000284	-7.214271	-8.003591
H	0.087270	-7.291520	-7.868089
H	-1.236473	-7.269656	-9.067669
H	-1.493285	-8.043638	-7.478252

34
MeOBnOTHP

C	-2.082700	-3.237407	-0.817931
C	-1.708032	-2.505503	1.545071
C	-1.400604	-1.108803	1.002816
C	-2.096596	-0.913710	-0.338034
H	-1.730745	-0.332384	1.704483
H	-0.317601	-0.991101	0.864957
O	-1.729384	-1.928006	-1.274785
H	-1.821533	0.038217	-0.803053
H	-3.193104	-0.922313	-0.196806
O	-1.845824	-4.137420	-1.857621
C	-1.361317	-3.558768	0.488058
H	-1.158437	-2.696985	2.473617
H	-2.778386	-2.572846	1.789348
H	-1.650552	-4.564751	0.815657
H	-0.278500	-3.557849	0.311009
H	-3.176266	-3.274996	-0.658276
C	-0.465237	-4.304646	-2.240487
C	-0.421159	-4.682784	-3.690483
H	0.074531	-3.361763	-2.080965
H	0.004555	-5.088486	-1.627882
C	0.112200	-5.893347	-4.111902
C	0.168633	-6.233704	-5.464712
C	-0.331217	-5.341368	-6.411650
C	-0.880354	-4.120393	-5.999348
C	-0.921747	-3.800568	-4.655953
H	0.494538	-6.596832	-3.375839
H	0.592091	-7.187919	-5.755456
H	-1.266188	-3.442296	-6.754229
H	-1.355932	-2.857557	-4.333525
O	-0.334963	-5.561123	-7.758818
C	0.206039	-6.791019	-8.225637
H	1.266889	-6.889908	-7.957569
H	0.108913	-6.767064	-9.312410
H	-0.352209	-7.650352	-7.829580

43
MeOBnOSiPh2H

Si	-2.867949	0.227516	-1.558213
C	-3.569678	1.823519	-0.870674
C	-1.077358	0.015143	-1.068671
C	-0.702841	0.154429	0.274966
C	-0.083212	-0.281847	-2.009384
C	1.243425	-0.440423	-1.617565
C	0.621418	-0.003670	0.669224
C	1.597086	-0.302738	-0.278577
C	-2.902636	3.039828	-1.077103
C	-4.778286	1.842118	-0.163167

C	-5.306491	3.035062	0.322661
C	-3.428791	4.234976	-0.598411
C	-4.632622	4.233069	0.103005
H	-5.042882	5.165600	0.480998
H	-6.243578	3.031220	0.872983
H	-1.954814	3.049380	-1.612836
H	-2.899346	5.168762	-0.766714
H	-5.317605	0.911130	0.000261
H	-1.454625	0.399012	1.024042
H	0.894620	0.109545	1.714727
H	2.632665	-0.425443	0.026826
H	-0.353499	-0.384853	-3.056819
H	2.003720	-0.670098	-2.359206
O	-2.837576	0.242309	-3.219133
H	-3.710101	-0.878303	-1.024367
C	-3.948901	0.690621	-4.010974
C	-5.235301	0.002229	-3.646840
H	-3.676654	0.481648	-5.052923
H	-4.065797	1.778534	-3.901013
C	-5.368667	-1.382454	-3.813871
C	-6.300073	0.706616	-3.096648
C	-7.480868	0.066509	-2.719683
C	-7.593247	-1.312748	-2.893115
C	-6.527408	-2.036938	-3.443179
H	-8.288873	0.650573	-2.294724
H	-6.210007	1.779342	-2.942677
H	-6.637337	-3.109444	-3.569227
H	-4.540734	-1.950542	-4.231477
O	-8.691906	-2.048540	-2.561834
C	-9.801488	-1.358617	-1.997280
H	-10.561245	-2.119028	-1.809219
H	-10.201986	-0.607909	-2.691910
H	-9.531092	-0.872947	-1.049793

46

MeOBnOSiPh2Me

Si	-2.838517	0.193592	-1.438933
C	-3.538368	1.820901	-0.810142
C	-1.027408	0.062240	-0.978139
C	-0.628129	0.277615	0.348244
C	-0.044305	-0.266991	-1.919857
C	1.292274	-0.383469	-1.546850
C	0.705885	0.162918	0.724987
C	1.669048	-0.170101	-0.224223
C	-2.944288	3.024837	-1.217031
C	-4.654594	1.890861	0.032599
C	-5.162575	3.116967	0.454377
C	-3.451219	4.252942	-0.805670
C	-4.562849	4.300023	0.032609
H	-4.958680	5.258052	0.358594
H	-6.027395	3.150270	1.111701
H	-2.068130	2.997465	-1.863297
H	-2.978019	5.174601	-1.133375
H	-5.142317	0.974740	0.358314
H	-1.368735	0.553369	1.098019
H	0.996716	0.337858	1.757233
H	2.712279	-0.258760	0.066729
H	-0.331296	-0.426859	-2.955878
H	2.042400	-0.638564	-2.290676
O	-2.818778	0.200906	-3.104500
C	-3.801320	-1.244588	-0.725315
C	-3.897524	0.688027	-3.915303
C	-5.202952	0.011030	-3.602010
H	-3.601890	0.499805	-4.954769
H	-4.001635	1.774575	-3.781002
C	-5.389990	-1.342822	-3.908407
C	-6.221447	0.684316	-2.936670
C	-7.402583	0.039634	-2.568761
C	-7.566528	-1.311199	-2.877005
C	-6.552201	-2.001162	-3.553742
H	-8.172170	0.598419	-2.049054
H	-6.089871	1.732203	-2.677604
H	-6.703476	-3.050266	-3.788034
H	-4.599939	-1.886780	-4.420968
O	-8.671259	-2.047824	-2.568609
C	-9.732730	-1.389473	-1.885688
H	-10.507806	-2.143919	-1.741372
H	-10.137931	-0.560287	-2.481131
H	-9.406156	-1.011999	-0.907193
H	-3.654423	-1.309256	0.359350
H	-3.440207	-2.180545	-1.165056
H	-4.874557	-1.170093	-0.930906

19

MeBnOH

C	-4.798965	3.017529	-0.039808
C	-3.711342	3.878576	0.088655
C	-3.905495	5.256254	0.113510
C	-5.181027	5.812455	0.008064
C	-6.265474	4.941843	-0.123587
C	-6.078225	3.565483	-0.140314
C	-5.385310	7.302105	0.069283
H	-6.275197	7.606639	-0.489648
H	-4.523534	7.838681	-0.338557
H	-5.517821	7.636014	1.105913
H	-7.271440	5.347327	-0.205699
H	-2.712208	3.465203	0.172933
H	-3.044562	5.913298	0.216610
H	-6.940879	2.908424	-0.235295
C	-4.622959	1.523859	-0.113170
O	-3.348801	1.160585	0.414831
H	-4.710341	1.202192	-1.165641
H	-5.437018	1.037224	0.449118
H	-3.217835	0.220706	0.255961

16

FBnOH

C	-2.978139	1.285170	-0.098775
C	-3.038396	-0.096356	-0.025979
C	-1.728585	1.893437	-0.050366
C	-0.563767	1.136532	0.083105
C	-0.663334	-0.253005	0.152769
C	-1.904011	-0.880236	0.095163
H	-3.890794	1.861988	-0.204954
H	-1.663863	2.976937	-0.120849
H	0.239880	-0.846850	0.241291
H	-1.997233	-1.960361	0.140417
C	0.769640	1.827382	0.188950
O	1.795959	0.954261	-0.280925
H	0.952603	2.103640	1.241932
H	0.742360	2.759900	-0.397732
F	-4.247959	-0.699879	-0.084947
H	2.646379	1.358460	-0.081628

27

FBnOTHF

C	-2.287061	1.876334	0.015494
C	-3.119711	0.783918	0.195742
C	-0.914647	1.662528	-0.045181
C	-0.386727	0.375199	0.067217
C	-1.257230	-0.701562	0.234531
C	-2.633357	-0.507263	0.307918
H	-2.713012	2.870021	-0.077078
H	-0.238753	2.498910	-0.198373

H	-0.859305	-1.711324	0.308721
H	-3.322208	-1.335570	0.436624
C	1.110855	0.153992	0.042101
F	-4.456204	0.984174	0.255040
C	1.602422	0.946103	-2.155467
C	2.666680	0.028043	-2.753125
O	1.790520	2.192078	-2.761987
C	3.147450	2.304937	-3.253666
C	3.848039	0.994709	-2.883158
H	3.615615	3.184905	-2.799159
H	3.097440	2.451627	-4.340961
H	4.358829	1.090163	-1.920378
H	4.577295	0.685465	-3.636675
H	2.332985	-0.325139	-3.736423
H	2.875488	-0.839691	-2.121386
O	1.809094	1.108770	-0.743825
H	1.326512	-0.877337	-0.286886
H	1.521910	0.259774	1.053900
H	0.565303	0.626940	-2.335341

30

FBnOTHP

C	-1.884264	2.074001	0.477618
C	-2.994896	1.261791	0.632981
C	-0.677465	1.488154	0.109780
C	-0.589119	0.112174	-0.103590
C	-1.732826	-0.672531	0.048195
C	-2.946281	-0.106557	0.425522
H	-1.970799	3.143366	0.640109
H	0.206875	2.102385	-0.031353
H	-1.679936	-1.744707	-0.129309
H	-3.843527	-0.704274	0.547378
C	0.734046	-0.533403	-0.458736
F	-4.171884	1.824690	0.991833
C	1.301700	0.720864	-2.417160
C	2.029341	-0.183224	-3.400550
O	1.624585	2.073694	-2.600080
C	2.919825	2.336523	-3.171332
C	3.955925	1.318505	-2.727478
H	3.180710	3.347912	-2.844438
H	2.837781	2.347768	-4.271226
O	1.654744	0.357286	-1.068095
H	0.555259	-1.423578	-1.086758
H	1.234054	-0.884193	0.453166
H	0.208445	0.648532	-2.524152
C	3.553862	-0.091149	-3.205240
H	1.675456	-1.213440	-3.282247
H	1.745156	0.131752	-4.412796
H	4.049828	-0.331749	-4.152508
H	3.882474	-0.834894	-2.473557
H	4.017184	1.344424	-1.634211
H	4.938388	1.603836	-3.121197

16

BnOH

C	-6.654696	2.806998	0.217286
C	-7.654994	3.658438	-0.248596
C	-6.998611	1.581653	0.777626
C	-8.337658	1.210326	0.873204
C	-9.343876	2.052899	0.401471
C	-8.990061	3.284190	-0.158031
H	-7.391334	4.617119	-0.686456
H	-6.224813	0.916157	1.149671
H	-8.603799	0.254981	1.320406
H	-9.773465	3.946890	-0.516020
C	-10.795533	1.651257	0.492724
O	-11.634970	2.696820	0.987070
H	-10.890058	0.740303	1.104994
H	-11.188530	1.417335	-0.504349
H	-11.258198	2.997743	1.822445
H	-5.611458	3.100925	0.146451

27

BnOTHF

C	-2.340162	-2.919377	-0.773036
C	-1.152924	-1.648664	0.883915
C	-0.887720	-1.083333	-0.508823
O	-1.869422	-1.697006	-1.358306
H	0.127503	-1.340781	-0.854118
H	-1.013722	0.002042	-0.577639
O	-2.240684	-3.989032	-1.678909
C	-1.568545	-3.079731	0.547285
H	-3.422586	-2.826158	-0.611535
C	-0.921362	-4.297031	-2.147504
C	-1.011974	-4.800564	-3.561414
H	-0.299377	-3.390147	-2.126133
H	-0.452164	-5.057802	-1.505730
C	-0.391478	-5.989308	-3.939038
C	-0.446363	-6.426615	-5.260626
C	-1.131989	-5.679847	-6.212013
C	-1.760858	-4.493250	-5.837552
C	-1.700665	-4.055013	-4.521030
H	0.135308	-6.581075	-3.193803
H	0.038955	-7.356463	-5.543327
H	-2.300096	-3.908089	-6.577267
H	-2.193425	-3.135140	-4.218453
H	-1.979383	-1.106643	1.358116
H	-0.279679	-1.590356	1.540632
H	-0.685518	-3.707481	0.383777
H	-2.184379	-3.557193	1.313594
H	-1.181079	-6.021599	-7.241919

30

BnOTHP

C	-2.122865	-3.180573	-0.691577
C	-1.664399	-2.344936	1.621552
C	-1.313547	-0.992123	1.000356
C	-2.032582	-0.836527	-0.333400
H	-1.595092	-0.167565	1.667377
H	-0.230221	-0.927020	0.833193
O	-1.730455	-1.912720	-1.223832
H	-1.729078	0.077211	-0.853864
H	-3.124852	-0.790751	-0.168702
O	-1.941595	-4.141238	-1.688688
C	-1.385894	-3.464709	0.613956
H	-1.100069	-2.511218	2.546019
H	-2.730140	-2.355006	1.893422
H	-1.708834	-4.439595	0.998962
H	-0.308225	-3.518715	0.414993
H	-3.213307	-3.168700	-0.509299
C	-0.581802	-4.378047	-2.090235
C	-0.575047	-4.810046	-3.529588
H	0.001915	-3.453535	-1.981317
H	-0.127801	-5.156038	-1.458414
C	0.118936	-5.949134	-3.931565
C	0.155457	-6.317904	-5.274413
C	-0.512797	-5.552987	-6.223825
C	-1.215692	-4.416804	-5.825605
C	-1.246071	-4.046270	-4.487477
H	0.632211	-6.555817	-3.188893
H	0.698053	-7.209390	-5.576015
H	-0.490815	-5.841762	-7.270763
H	-1.741611	-3.817918	-6.563977
H	-1.796556	-3.166339	-4.166239

26
PhBnOH

C	-4.793587	4.529307	0.093080
C	-5.167002	3.179433	0.104081
C	-3.457167	4.896354	-0.009328
C	-2.467258	3.922408	-0.103794
C	-2.825413	2.577401	-0.095013
C	-4.161383	2.209142	0.008110
H	-5.557438	5.295342	0.194938
H	-3.186817	5.948494	-0.005927
H	-1.423095	4.209924	-0.184475
H	-2.061052	1.810072	-0.178986
H	-4.435189	1.157869	-0.015094
C	-6.587292	2.788229	0.214406
C	-7.586436	3.505060	-0.459738
C	-6.974094	1.695958	0.997906
C	-8.312386	1.334996	1.104556
C	-9.301239	2.045649	0.425741
C	-8.919955	3.139348	-0.356598
H	-7.305972	4.339690	-1.096629
H	-6.222679	1.146903	1.558794
H	-8.592533	0.493430	1.734390
H	-9.682954	3.701134	-0.889003
C	-10.753478	1.654804	0.531555
O	-11.611989	2.766617	0.798398
H	-10.871149	0.867952	1.293608
H	-11.108400	1.243154	-0.421281
H	-11.265627	3.216168	1.578360

22
MeOOCBnOH

C	-5.018828	3.165234	-0.025172
C	-3.818705	3.756987	-0.429485
C	-3.715890	5.135274	-0.533610
C	-4.812236	5.949473	-0.233661
C	-6.015119	5.363257	0.169147
C	-6.113432	3.981551	0.265051
C	-4.643610	7.418607	-0.363992
H	-6.871030	5.989217	0.395058
H	-2.968071	3.123129	-0.662166
H	-2.788837	5.606103	-0.845280
H	-7.055548	3.529601	0.567231
C	-5.126770	1.665176	0.109497
O	-3.620307	7.964366	-0.718461
O	-5.770007	8.095315	-0.038538
C	-5.664575	9.528651	-0.142865
H	-6.641262	9.914513	0.150587
H	-5.425531	9.820730	-1.169362
H	-4.883635	9.903706	0.524608
O	-4.360809	0.950322	-0.855848
H	-6.187206	1.367411	0.082547
H	-4.728495	1.351917	1.083126
H	-4.662381	1.224444	-1.730142

33
MeOOCBnOTHF

C	-2.323204	-3.356848	-0.920338
C	-1.550646	-1.759695	0.693998
C	-1.376791	-1.207926	-0.718891
O	-2.129837	-2.092195	-1.565975
H	-0.315999	-1.210503	-1.018617
H	-1.772636	-0.195822	-0.850145
O	-1.941028	-4.417048	-1.762035
C	-1.584389	-3.263260	0.423455
H	-3.401904	-3.517435	-0.789292
C	-0.577135	-4.416065	-2.198453
C	-0.526857	-4.781504	-3.656617
H	-0.138093	-3.416393	-2.069938
H	0.010119	-5.131942	-1.604941
C	0.325078	-5.782646	-4.117385
C	0.378697	-6.099060	-5.470627
C	-0.411210	-5.399054	-6.382817
C	-1.247827	-4.375821	-5.924854
C	-1.317742	-4.082759	-4.572830
H	0.951642	-6.324644	-3.413056
H	1.054775	-6.875741	-5.816203
H	-1.834106	-3.814407	-6.646111
H	-1.982205	-3.304298	-4.208704
C	-0.314179	-5.625334	-7.855618
O	-0.213774	-4.714331	-8.640827
O	-0.304998	-6.901261	-8.327383
C	-0.830633	-7.992869	-7.549287
H	-0.015471	-8.558861	-7.087425
H	-1.361435	-8.636476	-8.254825
H	-1.520603	-7.645071	-6.775827
H	-2.503937	-1.421154	1.116018
H	-0.746633	-1.455289	1.370586
H	-0.567269	-3.656889	0.313962
H	-2.089828	-3.845170	1.198147

36
MeOOCBnOTHP

C	-2.119417	-3.352191	-0.890419
C	-1.756025	-2.423206	1.404085
C	-1.343482	-1.107765	0.742066
C	-1.997331	-0.995093	-0.628363
H	-1.634891	-0.248409	1.358806
H	-0.252477	-1.074269	0.622816
O	-1.679204	-2.117175	-1.456726
H	-1.648915	-0.113060	-1.174353
H	-3.094462	-0.919671	-0.518105
O	-1.899454	-4.358244	-1.836769
C	-1.456592	-3.593676	0.462027
H	-1.241450	-2.560413	2.361627
H	-2.833611	-2.397882	1.622838
H	-1.824396	-4.541931	0.872259
H	-0.371976	-3.684155	0.321582
H	-3.217584	-3.320507	-0.768619
C	-0.528262	-4.604824	-2.181988
C	-0.450641	-4.930751	-3.647350
H	0.076654	-3.709823	-1.981942
H	-0.130583	-5.437216	-1.582768
C	0.266828	-6.035899	-4.099860
C	0.355629	-6.313536	-5.459526
C	-0.262548	-5.472864	-6.385691
C	-0.961919	-4.348811	-5.934351
C	-1.069368	-4.091006	-4.577535
H	0.759980	-6.688979	-3.383846
H	0.926750	-7.172805	-5.798747
H	-1.411675	-3.682445	-6.664274
H	-1.629927	-3.232595	-4.218639
C	-0.111216	-5.666476	-7.858272
O	0.171312	-4.758765	-8.601801
O	-0.263716	-6.915146	-8.377374
C	-0.997292	-7.934690	-7.673321
H	-0.309665	-8.624881	-7.174227
H	-1.563118	-8.476977	-8.434551
H	-1.683657	-7.505545	-6.938470

17
NCBnOH

C	-3.021146	0.930836	0.105802
C	-3.220145	-0.451257	-0.007723

C	-1.742300	1.451499	-0.016724
C	-0.649741	0.616904	-0.262818
C	-0.856024	-0.760178	-0.373752
C	-2.128047	-1.296169	-0.247553
H	-3.868668	1.579982	0.299804
H	-1.590269	2.523576	0.083683
H	-0.005544	-1.408857	-0.560030
H	-2.289034	-2.365813	-0.334038
C	0.733844	1.201969	-0.424607
C	-4.529066	-0.996369	0.129223
N	-5.593207	-1.439624	0.241855
O	1.775817	0.325746	-0.012854
H	0.787886	2.169490	0.099264
H	0.924731	1.401206	-1.487064
H	1.661687	0.150956	0.928906

28
NCBnOTHF

C	-2.701675	0.308576	-0.656169
C	-2.838146	-0.499357	0.483029
C	-1.444662	0.533727	-1.190345
C	-0.308067	-0.035490	-0.607522
C	-0.454068	-0.844646	0.518840
C	-1.706673	-1.077064	1.070200
H	-3.584484	0.751734	-1.105395
H	-1.332830	1.150244	-2.077978
H	0.423464	-1.306014	0.964177
H	-1.820304	-1.708427	1.945312
C	1.053594	0.222809	-1.206309
C	-4.128787	-0.733700	1.038780
N	-5.178849	-0.921640	1.490083
O	1.093115	-0.041658	-2.611521
H	1.817472	-0.366658	-0.682263
H	1.310743	1.284163	-1.110093
C	0.947941	-1.425524	-2.925203
C	1.056841	-1.553880	-4.451677
O	1.994820	-2.200169	-2.374609
H	0.015701	-1.810870	-2.489294
C	3.088152	-2.132977	-3.308625
C	2.414581	-2.253771	-4.675071
H	3.772850	-2.950896	-3.070148
H	3.617218	-1.174203	-3.202340
H	2.268463	-3.308827	-4.925902
H	3.002164	-1.790425	-5.472489
H	1.023376	-0.557075	-4.898821
H	0.224638	-2.136468	-4.854273

31
NCBnOTHP

C	-2.244315	1.069789	0.397807
C	-3.076515	-0.058949	0.380983
C	-1.028264	1.032477	-0.261567
C	-0.613688	-0.118816	-0.938409
C	-1.445678	-1.237682	-0.953808
C	-2.670187	-1.211612	-0.300845
H	-2.563771	1.963855	0.922923
H	-0.388784	1.912231	-0.252631
H	-1.129545	-2.128909	-1.483170
H	-3.319682	-2.080845	-0.314121
C	0.728699	-0.115095	-1.614635
C	-4.331574	-0.029338	1.053407
N	-5.352335	-0.003479	1.600458
O	0.879446	-1.287273	-2.404622
H	1.524609	-0.076029	-0.848483
H	0.832830	0.783657	-2.239235
C	2.178250	-1.481679	-2.879486
C	2.238356	-2.832169	-3.596097
O	2.493844	-0.428468	-3.782737
H	2.894714	-1.436745	-2.029667
C	3.242577	-2.767900	-4.758826
H	1.233786	-3.072256	-3.959693
H	2.523897	-3.606720	-2.874189
C	3.888767	-0.435453	-4.120791
C	4.412277	-1.861735	-4.374457
H	4.468193	0.060995	-3.324656
H	3.959713	0.174145	-5.026925
H	4.897282	-2.259681	-3.473450
H	5.174626	-1.839215	-5.160625
H	2.749498	-2.361703	-5.649438
H	3.594948	-3.772576	-5.013778

20
MeOBnOH•−

C	-4.795771	3.245829	-0.040877
C	-3.577710	3.893023	-0.154951
C	-3.516197	5.336013	-0.276237
C	-4.730155	6.032407	-0.297302
C	-5.965005	5.404801	-0.206745
C	-6.011374	3.960150	-0.081697
H	-6.868356	6.003696	-0.265176
H	-2.659930	3.310328	-0.209601
H	-2.556492	5.836668	-0.294417
H	-6.941972	3.467336	0.196345
C	-4.857209	1.748687	0.067237
O	-5.693000	1.171287	-0.965338
H	-5.274970	1.445486	1.044292
H	-3.848748	1.319258	-0.023391
H	-6.110141	1.954253	-1.363728
O	-4.790724	7.425635	-0.417261
C	-3.566311	8.114213	-0.505021
H	-3.815480	9.175560	-0.615929
H	-2.972044	7.790089	-1.374476
H	-2.951604	7.982095	0.400069

31
MeOBnOTHF•−

C	-2.227058	-2.197806	-1.077743
C	-1.325550	-4.311651	-0.259836
C	-0.242187	-3.249448	-0.414570
O	-0.948177	-1.999835	-0.453793
H	0.309492	-3.409545	-1.353462
H	0.458326	-3.202811	0.428346
O	-2.344757	-1.533145	-2.310526
C	-2.464484	-3.726574	-1.102116
H	-2.962157	-1.675512	-0.447558
C	-1.316794	-1.774532	-3.315923
C	-1.329768	-3.122409	-3.983621
H	-1.529085	-0.984643	-4.048918
H	-0.332269	-1.575659	-2.869960
C	-0.149526	-3.853216	-4.035225
C	-0.100842	-5.137989	-4.702415
C	-1.302081	-5.632323	-5.229864
C	-2.497971	-4.932815	-5.166255
C	-2.511915	-3.618335	-4.554025
H	0.761854	-3.439578	-3.607531
H	0.835024	-5.676622	-4.774307
H	-3.394323	-5.377670	-5.586150
H	-3.435217	-3.051746	-4.478076
O	-1.381234	-6.875093	-5.866162
H	-1.613030	-4.404103	0.796226
H	-0.997446	-5.289909	-0.626004
H	-2.431156	-4.078615	-2.137011
H	-3.453344	-3.963287	-0.695412
C	-0.217393	-7.667646	-5.872468

H	0.604430	-7.193558	-6.432992
H	-0.487125	-8.608306	-6.365428
H	0.140182	-7.881976	-4.852758

34

MeOBnOTHP•−

C	-2.320438	-3.634111	-0.069229
C	-0.983310	-1.445190	0.197905
C	-1.707776	-0.849822	-1.010032
C	-3.065538	-1.539373	-1.179307
H	-1.840933	0.237857	-0.895164
H	-1.114652	-1.022117	-1.919902
O	-2.936614	-2.921416	-1.347785
H	-3.594875	-1.131928	-2.057620
H	-3.688287	-1.300637	-0.286328
O	-2.320695	-4.909220	-0.224987
C	-0.927478	-2.971883	0.090783
H	0.024914	-1.015471	0.300132
H	-1.536997	-1.169837	1.110126
H	-0.458441	-3.406962	0.984662
H	-0.329739	-3.280797	-0.778304
H	-3.002611	-3.218068	0.737116
C	-0.136713	-6.682770	-1.766306
C	-0.222666	-6.105160	-3.044782
H	-0.791306	-6.310089	-0.976285
H	0.597279	-7.460912	-1.569265
C	0.601996	-6.497425	-4.130110
C	0.497080	-5.901437	-5.381221
C	-0.440800	-4.884206	-5.580923
C	-1.269911	-4.473794	-4.524740
C	-1.173624	-5.067784	-3.288065
H	1.336873	-7.285649	-3.977749
H	1.148413	-6.233604	-6.183036
H	-1.988890	-3.677251	-4.695810
H	-1.809495	-4.749065	-2.460084
O	-0.625105	-4.222297	-6.777993
C	0.201569	-4.603209	-7.859769
H	1.266810	-4.438818	-7.636765
H	-0.089564	-3.971513	-8.703088
H	0.055433	-5.659390	-8.133239

43

MeOBnOSiPh2H•−

Si	-2.755819	0.148243	-1.607555
C	-3.673100	1.501942	-0.748403
C	-0.965950	-0.052224	-1.168361
C	0.008641	0.914437	-1.532499
C	-0.524342	-1.116874	-0.348795
C	0.789168	-1.216339	0.080727
C	1.319465	0.824898	-1.091001
C	1.732161	-0.238855	-0.279058
C	-3.313063	2.873233	-0.904286
C	-4.763471	1.233719	0.119625
C	-5.450277	2.243382	0.768678
C	-3.997248	3.883291	-0.249038
C	-5.077733	3.591958	0.597509
H	-5.613587	4.386736	1.108479
H	-6.290168	1.992361	1.414399
H	-2.481412	3.135665	-1.557309
H	-3.693142	4.918511	-0.397018
H	-5.083298	0.201597	0.257205
H	-0.277240	1.736429	-2.185777
H	2.039380	1.585025	-1.390162
H	2.761391	-0.308949	0.062182
H	-1.239789	-1.881919	-0.048992
H	1.091795	-2.058474	0.700340
O	-2.722343	0.477402	-3.263940
H	-3.452713	-1.136198	-1.283681
C	-3.843516	1.118435	-3.890339
C	-5.141971	0.389679	-3.685851
H	-3.598095	1.163697	-4.961669
H	-3.950437	2.148784	-3.519068
C	-5.343399	-0.889370	-4.236718
C	-6.164540	0.946661	-2.924328
C	-7.365662	0.273539	-2.701943
C	-7.545152	-0.998123	-3.261353
C	-6.532533	-1.575447	-4.025497
H	-8.124229	0.738130	-2.082767
H	-6.009443	1.915696	-2.456261
H	-6.693109	-2.566894	-4.439911
H	-4.544360	-1.354434	-4.808617
O	-8.687618	-1.758767	-3.107639
C	-9.704517	-1.226860	-2.278478
H	-10.501624	-1.974602	-2.258672
H	-10.105453	-0.283177	-2.677734
H	-9.344189	-1.053795	-1.253962

46

MeOBnOSiPh2Me•−

Si	-2.773159	0.125894	-1.541617
C	-3.647106	1.485562	-0.635864
C	-0.966177	-0.023977	-1.120178
C	-0.031069	0.947533	-1.562931
C	-0.469685	-1.020405	-0.247869
C	0.856586	-1.051410	0.154506
C	1.292894	0.926238	-1.153300
C	1.759162	-0.072229	-0.289021
C	-3.258575	2.847193	-0.801780
C	-4.694584	1.244094	0.292841
C	-5.318417	2.270410	0.979385
C	-3.882294	3.874037	-0.114197
C	-4.925633	3.608814	0.787289
H	-5.415103	4.415749	1.325010
H	-6.126315	2.038600	1.671596
H	-2.450355	3.089290	-1.491140
H	-3.558486	4.900982	-0.276638
H	-5.039142	0.223666	0.451295
H	-0.359465	1.716564	-2.259143
H	1.980254	1.687632	-1.518305
H	2.798268	-0.091065	0.028220
H	-1.144763	-1.792088	0.118516
H	1.199515	-1.842972	0.818313
O	-2.733447	0.504905	-3.185800
C	-3.642043	-1.515979	-1.218779
C	-3.831500	1.178045	-3.818118
C	-5.143783	0.468125	-3.638318
H	-3.571735	1.236376	-4.885224
H	-3.922467	2.203358	-3.429365
C	-5.385768	-0.766877	-4.265637
C	-6.114842	0.982850	-2.788275
C	-7.311011	0.301904	-2.540558
C	-7.530215	-0.930720	-3.172831
C	-6.573206	-1.460636	-4.032625
H	-8.030726	0.729930	-1.853034
H	-5.923756	1.918513	-2.269111
H	-6.768266	-2.420395	-4.502709
H	-4.623794	-1.198184	-4.909869
O	-8.669336	-1.692574	-3.001722
C	-9.636093	-1.205852	-2.088697
H	-10.438342	-1.948256	-2.072752
H	-10.047671	-0.236843	-2.407917
H	-9.221059	-1.098619	-1.076079
H	-3.574038	-1.814259	-0.166038
H	-3.187071	-2.306361	-1.826823

H	-4.701779	-1.453361	-1.489125

19
MeBnOH•–

C	-4.641954	3.013935	-0.121894
C	-3.619962	3.982264	-0.246287
C	-3.861225	5.339151	-0.181603
C	-5.201953	5.836278	0.025401
C	-6.218629	4.880384	0.165968
C	-5.984452	3.515891	0.104553
C	-5.456601	7.308570	0.113723
H	-6.524291	7.515075	0.263929
H	-5.145235	7.852281	-0.797485
H	-4.912432	7.790787	0.947823
H	-7.239973	5.231039	0.336435
H	-2.597148	3.632646	-0.404629
H	-3.038458	6.044920	-0.294125
H	-6.800860	2.811341	0.248742
C	-4.368091	1.585174	-0.239855
O	-4.347629	0.878933	1.095089
H	-3.389006	1.414583	-0.717131
H	-5.147605	1.065180	-0.831553
H	-4.440899	-0.068388	0.900289

16
FBnOH•–

C	-2.919914	1.274537	-0.260537
C	-2.900160	-0.115996	-0.122301
C	-1.654472	1.943823	-0.086125
C	-0.511972	1.233381	0.220457
C	-0.519036	-0.186231	0.319471
C	-1.791618	-0.871513	0.157512
H	-3.847803	1.804051	-0.438811
H	-1.590538	3.020451	-0.236675
H	0.317759	-0.699189	0.791869
H	-1.874416	-1.952279	0.215511
C	0.806864	1.930656	0.374807
O	1.796417	1.408331	-0.546752
H	1.199602	1.797237	1.399791
H	0.696946	3.008058	0.185854
F	-4.106820	-0.775788	-0.294233
H	1.369898	0.601490	-0.882366

27
FBnOTHF•–

C	-2.448073	1.697324	0.031502
C	-2.953787	0.435361	-0.107442
C	-1.011623	1.850736	0.173250
C	-0.208191	0.681869	0.132886
C	-0.769824	-0.572254	-0.031442
C	-2.192925	-0.744965	-0.140190
H	-3.119141	2.550446	0.036440
H	-0.557411	2.831734	0.263790
H	-0.119862	-1.446257	-0.063600
H	-2.666097	-1.713174	-0.244127
C	1.277430	0.800582	0.263882
F	-4.325590	0.291212	-0.232275
C	1.481133	1.386591	-2.069398
C	2.006785	0.060728	-2.620961
O	2.088529	2.359277	-2.899891
C	3.423686	1.924758	-3.199557
C	3.474017	0.405968	-2.918453
H	4.145114	2.473846	-2.577783
H	3.615327	2.166602	-4.253478
H	4.094515	0.201430	-2.040895
H	3.878829	-0.159091	-3.764298
H	1.456046	-0.182579	-3.536801
H	1.878510	-0.764044	-1.916247
O	1.926712	1.628986	-0.747182
H	1.734749	-0.203033	0.263660
H	1.556809	1.305164	1.199824
H	0.392555	1.513484	-2.132997

30
FBnOTHP•–

C	-2.277162	1.878317	0.427881
C	-3.069759	0.822219	0.074384
C	-0.838166	1.684300	0.438831
C	-0.332432	0.420991	0.040167
C	-1.178934	-0.610167	-0.329948
C	-2.608142	-0.448773	-0.303277
H	-2.730065	2.827159	0.697416
H	-0.163067	2.500770	0.672229
H	-0.753159	-1.569066	-0.624534
H	-3.299052	-1.241927	-0.559597
C	1.146330	0.184788	0.025417
F	-4.442611	1.005801	0.079763
C	1.373787	1.295956	-2.107039
C	1.496815	0.044919	-2.970367
O	2.086935	2.382685	-2.685037
C	3.223976	2.023940	-3.469975
C	3.922967	0.769774	-2.964033
H	3.896651	2.890366	-3.439674
H	2.920123	1.878571	-4.524064
O	1.918602	1.096537	-0.810791
H	1.354803	-0.855871	-0.276628
H	1.583426	0.349084	1.020614
H	0.329095	1.623658	-2.012874
C	2.958031	-0.432995	-3.022572
H	0.826542	-0.724925	-2.573988
H	1.129059	0.296371	-3.974224
H	3.120905	-1.012646	-3.940389
H	3.159201	-1.103393	-2.179927
H	4.236679	0.947565	-1.930301
H	4.823262	0.589948	-3.566892

16
BnOH•–

C	-6.641972	2.839333	0.266740
C	-7.648818	3.617851	-0.377562
C	-7.063040	1.649039	0.934321
C	-8.382365	1.264304	0.952913
C	-9.408260	2.031461	0.291639
C	-8.970490	3.240336	-0.364072
H	-7.364700	4.533489	-0.897457
H	-6.321949	1.027218	1.437539
H	-8.670812	0.350326	1.474414
H	-9.715045	3.861261	-0.864237
C	-10.823034	1.679993	0.396691
O	-11.543268	2.292296	1.594250
H	-10.968258	0.595331	0.511973
H	-11.401156	2.027621	-0.472703
H	-10.826570	2.773484	2.026663
H	-5.602314	3.150691	0.268785

27
BnOTHF•–

C	-2.669733	-1.775326	-1.131147
C	-1.547587	-2.658295	0.879437
C	-0.435571	-2.004262	0.040812
O	-1.049989	-1.235061	-0.941759
H	0.196707	-2.815367	-0.396051
H	0.234719	-1.388874	0.673636
O	-2.986870	-1.957746	-2.347278

C	-2.586656	-2.993100	-0.179265
H	-3.147797	-0.907564	-0.604681
C	0.311838	-3.234464	-3.187517
C	-0.305143	-4.282459	-3.890912
H	-0.280110	-2.477391	-2.670078
H	1.397418	-3.177912	-3.147660
C	0.446119	-5.297864	-4.548414
C	-0.183965	-6.324426	-5.227951
C	-1.581299	-6.385001	-5.280174
C	-2.336472	-5.395458	-4.637551
C	-1.727894	-4.357249	-3.956050
H	1.533089	-5.255901	-4.507169
H	0.409820	-7.089824	-5.723581
H	-3.422896	-5.438794	-4.674844
H	-2.305314	-3.577117	-3.443675
H	-1.954616	-1.924843	1.590621
H	-1.193781	-3.527917	1.451148
H	-2.273619	-3.857020	-0.781679
H	-3.580742	-3.203607	0.236911
H	-2.073442	-7.192814	-5.815981

30

BnOTHP•−

C	-3.090413	-2.449425	-1.046379
C	-2.041898	-3.044731	1.232641
C	-0.948013	-1.999549	1.011452
C	-1.461551	-0.919875	0.053112
H	-0.632689	-1.548159	1.965724
H	-0.066869	-2.476827	0.557792
O	-1.852812	-1.452985	-1.179382
H	-0.676879	-0.165433	-0.129412
H	-2.308606	-0.391852	0.548367
O	-3.491004	-2.827667	-2.204696
C	-2.565209	-3.567010	-0.108513
H	-1.674764	-3.867491	1.864857
H	-2.872606	-2.572216	1.781451
H	-3.389543	-4.277044	0.047102
H	-1.773087	-4.097604	-0.655300
H	-3.800622	-1.794682	-0.451037
C	0.395003	-3.233708	-2.963205
C	-0.076947	-4.308502	-3.735152
H	-0.300432	-2.586774	-2.428864
H	1.464359	-3.051366	-2.883269
C	0.809265	-5.183863	-4.426279
C	0.326491	-6.239412	-5.177843
C	-1.051587	-6.469715	-5.268091
C	-1.936486	-5.622321	-4.590151
C	-1.478436	-4.553994	-3.839249
H	1.881604	-5.010166	-4.353620
H	1.020997	-6.894937	-5.699414
H	-1.428085	-7.300367	-5.860105
H	-3.008232	-5.797811	-4.651297
H	-2.181356	-3.900430	-3.299433

26

PhBnOH•−

C	-4.772005	4.479995	-0.341699
C	-5.203789	3.185455	0.095858
C	-3.438053	4.831148	-0.404551
C	-2.421275	3.931531	-0.041477
C	-2.814993	2.654005	0.391730
C	-4.145427	2.290664	0.460357
H	-5.511002	5.227148	-0.613783
H	-3.174658	5.834671	-0.736860
H	-1.373717	4.212509	-0.092878
H	-2.056736	1.924835	0.674977
H	-4.383766	1.281946	0.782862
C	-6.588941	2.814105	0.163229
C	-7.638059	3.637401	-0.367470
C	-7.034295	1.587026	0.759909
C	-8.364498	1.238813	0.825073
C	-9.386619	2.065065	0.299142
C	-8.964802	3.277434	-0.298743
H	-7.387242	4.566759	-0.869684
H	-6.307672	0.907345	1.194124
H	-8.643710	0.298079	1.301061
H	-9.717580	3.939863	-0.727742
C	-10.821708	1.728917	0.453930
O	-11.415588	2.266226	1.690696
H	-10.969634	0.638968	0.481420
H	-11.415499	2.135830	-0.378347
H	-10.680319	2.719100	2.120922

22

MeOOCBnOH•−

C	-4.926768	3.111727	0.070534
C	-3.690923	3.763225	-0.194955
C	-3.599273	5.128194	-0.311753
C	-4.754904	5.967672	-0.178216
C	-6.001413	5.309825	0.092665
C	-6.071987	3.941581	0.206082
C	-4.611486	7.376190	-0.309348
H	-6.897041	5.910547	0.213032
H	-2.792268	3.154728	-0.303534
H	-2.644399	5.608645	-0.504824
H	-7.034942	3.471843	0.410592
C	-5.032829	1.635027	0.100882
O	-3.572364	8.019316	-0.537481
O	-5.837600	8.066765	-0.147842
C	-5.722388	9.472782	-0.274717
H	-6.732505	9.873168	-0.133589
H	-5.341227	9.763860	-1.262847
H	-5.045436	9.897291	0.479043
O	-5.299796	1.040424	-1.223603
H	-5.854114	1.310191	0.757217
H	-4.099763	1.178681	0.464545
H	-5.328858	1.803300	-1.813612

33

MeOOCBnOTHF•−

C	-2.499955	-2.873109	-0.727372
C	-0.951327	-2.309940	1.084509
C	-1.018674	-1.215939	0.019590
O	-2.245113	-1.447784	-0.669171
H	-0.160868	-1.284778	-0.670596
H	-1.053592	-0.201267	0.432928
O	-2.374055	-3.413261	-2.001232
C	-1.575082	-3.501622	0.349832
H	-3.562123	-2.999233	-0.477162
C	-1.081152	-3.161033	-2.700444
C	-0.943175	-4.022878	-3.886029
H	-1.076467	-2.094883	-2.964036
H	-0.264943	-3.350857	-1.985756
C	-0.385428	-5.324586	-3.789171
C	-0.275737	-6.154707	-4.878516
C	-0.734426	-5.740338	-6.172927
C	-1.310268	-4.429706	-6.264257
C	-1.410200	-3.615893	-5.163163
H	-0.031818	-5.671550	-2.816990
H	0.166402	-7.139521	-4.768181
H	-1.663843	-4.098782	-7.236405
H	-1.858301	-2.627309	-5.265672
C	-0.642477	-6.544032	-7.342606
O	-1.011661	-6.259426	-8.494724

O	-0.045724	-7.804628	-7.094037
C	0.048574	-8.628054	-8.242572
H	0.524044	-9.557714	-7.910451
H	0.653937	-8.161609	-9.031565
H	-0.939164	-8.847783	-8.670106
H	-1.561413	-2.020699	1.948680
H	0.070000	-2.501229	1.432033
H	-0.815807	-4.118187	-0.138701
H	-2.150222	-4.155433	1.011410

36

MeOOCBnOTHP•−

C	-2.021656	-3.091946	-0.674501
C	-1.397365	-2.539811	1.692164
C	-1.376367	-1.063851	1.291445
C	-2.275868	-0.851270	0.076468
H	-1.711050	-0.423139	2.118919
H	-0.352147	-0.764449	1.031040
O	-1.904888	-1.685096	-1.008898
H	-2.214559	0.180193	-0.290261
H	-3.328744	-1.048205	0.361149
O	-1.873056	-3.848412	-1.812848
C	-1.062071	-3.409621	0.477077
H	-0.697604	-2.733175	2.515191
H	-2.401806	-2.799320	2.060160
H	-1.138537	-4.477796	0.715616
H	-0.034472	-3.207828	0.153671
H	-3.062626	-3.263395	-0.331484
C	-0.495543	-3.884594	-2.419242
C	-0.524284	-4.540319	-3.734120
H	-0.157757	-2.842199	-2.482016
H	0.146597	-4.440452	-1.721703
C	-0.304447	-5.935244	-3.869072
C	-0.350771	-6.567773	-5.088484
C	-0.637033	-5.836975	-6.289145
C	-0.875323	-4.430223	-6.146852
C	-0.824274	-3.817384	-4.920210
H	-0.087353	-6.519033	-2.973563
H	-0.165730	-7.634975	-5.153762
H	-1.096342	-3.861335	-7.045411
H	-1.017744	-2.747305	-4.844981
C	-0.687990	-6.424406	-7.583668
O	-0.922922	-5.863821	-8.667628
O	-0.426325	-7.816189	-7.562071
C	-0.470508	-8.425389	-8.840030
H	0.271507	-7.993023	-9.524843
H	-1.457135	-8.317321	-9.310678
H	-0.251511	-9.486595	-8.677209

17

NCBnOH•−

C	-2.944475	0.880569	-0.440314
C	-3.221727	-0.485501	-0.069733
C	-1.675748	1.391946	-0.355068
C	-0.571227	0.615831	0.107781
C	-0.847631	-0.734702	0.475990
C	-2.106816	-1.270780	0.400348
H	-3.765413	1.503493	-0.786096
H	-1.500330	2.428057	-0.645649
H	-0.026626	-1.356873	0.833293
H	-2.284289	-2.300488	0.699875
C	0.806509	1.148097	0.096561
C	-4.504269	-1.018748	-0.156127
N	-5.589179	-1.470625	-0.229298
O	1.516720	0.932010	-1.186090
H	1.423710	0.669929	0.872065
H	0.815067	2.234725	0.270281
H	0.866498	0.466957	-1.726445

28

NCBnOTHF•−

C	-2.511205	0.242052	-1.305490
C	-2.750225	-0.856848	-0.406135
C	-1.274667	0.822706	-1.402519
C	-0.163258	0.375898	-0.623953
C	-0.401001	-0.709611	0.264373
C	-1.629906	-1.307699	0.384049
H	-3.338893	0.611186	-1.906109
H	-1.126216	1.645745	-2.101608
H	0.425742	-1.077706	0.873651
H	-1.778580	-2.125763	1.083748
C	1.193576	0.911475	-0.854068
C	-4.004249	-1.452564	-0.301707
N	-5.064184	-1.955494	-0.208885
O	1.875362	0.446220	-2.088606
H	1.855124	0.663684	-0.006440
H	1.197834	1.999018	-0.999084
C	1.993899	-0.954459	-2.143834
C	0.784224	-1.642071	-2.821818
O	3.135009	-1.275803	-2.944626
H	2.194902	-1.346454	-1.131833
C	2.681683	-1.713125	-4.227367
C	1.408535	-2.497026	-3.930478
H	3.485013	-2.307772	-4.677169
H	2.470862	-0.848174	-4.879941
H	1.667443	-3.497511	-3.562954
H	0.757342	-2.606733	-4.804553
H	0.140504	-0.860250	-3.235302
H	0.183499	-2.205777	-2.103888

31

NCBnOTHP•−

C	-2.248272	-0.301995	-0.871125
C	-1.749191	-1.087369	0.227698
C	-1.419511	0.518405	-1.589629
C	-0.029570	0.648217	-1.285059
C	0.465007	-0.140575	-0.206032
C	-0.343117	-0.971130	0.526754
H	-3.304428	-0.368845	-1.120139
H	-1.827861	1.090491	-2.422547
H	1.526409	-0.079533	0.035502
H	0.065688	-1.550447	1.350497
C	0.870268	1.440133	-2.133945
C	-2.584984	-1.922608	0.964620
N	-3.289420	-2.627142	1.590669
O	1.248040	0.799960	-3.455478
H	1.814762	1.660299	-1.621232
H	0.416343	2.374725	-2.483627
C	1.589801	-0.526844	-3.342592
C	1.685826	-1.150244	-4.738318
O	2.881709	-0.646638	-2.696440
H	0.859615	-1.064959	-2.708247
C	2.711708	-2.298051	-4.738286
H	1.973989	-0.369351	-5.451075
H	0.691402	-1.513845	-5.026682
C	3.086543	-1.994663	-2.276784
C	2.697723	-3.004061	-3.381347
H	2.512772	-2.194985	-1.355588
H	4.155984	-2.068155	-2.044614
H	1.692620	-3.402234	-3.192236
H	3.387325	-3.857123	-3.369363
H	3.714859	-1.891737	-4.917925
H	2.499934	-3.006871	-5.548191

18
MeOBn−

C	-3.328954	2.353201	0.045960
C	-3.595703	0.999530	-0.013565
C	-1.990444	2.893239	0.103978
C	-0.969326	1.877371	0.093293
C	-1.245476	0.516230	0.033156
C	-2.563394	0.054623	-0.021238
H	-4.164184	3.052627	0.049447
H	-4.624891	0.645178	-0.055808
H	-0.408024	-0.177997	0.029756
H	0.071860	2.194315	0.134496
C	-1.724097	4.258280	0.163941
H	-2.530950	4.984703	0.169046
H	-0.704014	4.627960	0.204927
O	-2.953305	-1.301110	-0.083905
C	-1.906538	-2.232236	-0.091561
H	-1.239214	-2.103212	-0.962686
H	-2.366128	-3.225898	-0.141055
H	-1.285879	-2.170404	0.820428

17
MeBn−

C	-4.728843	2.928035	0.049608
C	-3.664476	3.904170	0.058844
C	-3.898891	5.265202	0.049843
C	-5.193665	5.815628	0.034676
C	-6.250564	4.886701	0.028111
C	-6.045813	3.521018	0.036798
C	-5.431541	7.298592	-0.046300
H	-5.500403	7.681397	-1.080415
H	-4.621909	7.860244	0.440228
H	-6.368398	7.582972	0.453467
H	-7.277666	5.260429	0.019512
H	-2.637228	3.541124	0.083559
H	-3.040913	5.942332	0.058484
H	-6.907399	2.853655	0.044095
C	-4.508467	1.556945	0.060784
H	-3.500612	1.152666	0.076164
H	-5.338700	0.856793	0.059263

14
FBn−

C	-3.023849	1.272967	0.051639
C	-3.079922	-0.115587	0.030561
C	-1.797664	1.916311	0.005610
C	-0.539029	1.209035	-0.064456
C	-0.682410	-0.229175	-0.082771
C	-1.912005	-0.865934	-0.036486
H	-3.948118	1.845600	0.104456
H	-1.774477	3.004772	0.022956
H	0.221021	-0.834711	-0.134984
H	-1.973761	-1.952643	-0.051895
C	0.693284	1.851170	-0.109680
F	-4.313544	-0.759170	0.079120
H	0.760046	2.934669	-0.093631
H	1.617396	1.283757	-0.161816

14
Bn−

C	-3.389138	2.485738	0.046598
C	-3.731737	1.150310	-0.016522
C	-2.017125	2.938215	0.106766
C	-1.052344	1.861376	0.093937
C	-1.418926	0.532352	0.030372
C	-2.764965	0.128928	-0.027029
H	-4.175620	3.240301	0.052092
H	-4.789458	0.884407	-0.059895
H	-0.633796	-0.225819	0.024658
H	-3.044104	-0.919320	-0.077375
H	0.004784	2.123322	0.137190
C	-1.660909	4.277568	0.170754
H	-2.416458	5.057808	0.178114
H	-0.618119	4.578366	0.213988

24
PhBn−

C	-4.740023	4.503200	-0.178172
C	-5.132401	3.162481	0.087998
C	-3.410059	4.877048	-0.279656
C	-2.379184	3.947446	-0.112890
C	-2.735436	2.622362	0.154923
C	-4.063150	2.238398	0.247265
H	-5.504299	5.268276	-0.275834
H	-3.169169	5.920497	-0.477251
H	-1.337162	4.244557	-0.189019
H	-1.958819	1.868955	0.277971
H	-4.290700	1.190425	0.417142
C	-6.518243	2.767361	0.189456
C	-7.581203	3.561402	-0.323920
C	-6.925874	1.554002	0.810471
C	-8.237947	1.173587	0.917107
C	-9.325668	1.966490	0.394271
C	-8.897932	3.191942	-0.238907
H	-7.346168	4.487262	-0.845642
H	-6.172349	0.910460	1.260595
H	-8.483873	0.244067	1.428954
H	-9.661385	3.833811	-0.676660
C	-10.648597	1.589338	0.490350
H	-10.931995	0.657086	0.969492
H	-11.439916	2.215372	0.089264

17
F3CBn−

C	-3.357448	2.324226	0.215000
C	-3.631575	0.983427	0.160746
C	-2.013252	2.854209	0.110195
C	-0.993669	1.840185	-0.063946
C	-1.280688	0.501389	-0.120183
C	-2.605687	0.019521	0.004335
H	-4.175476	3.030492	0.347751
H	-4.663193	0.646508	0.246558
H	-0.469531	-0.211399	-0.253873
H	0.041516	2.166907	-0.148866
C	-1.731063	4.201190	0.174221
H	-2.522048	4.933686	0.304139
H	-0.710495	4.563963	0.097794
C	-2.924041	-1.389061	-0.152149
F	-1.881664	-2.227264	0.147519
F	-3.299447	-1.786129	-1.447061
F	-3.990340	-1.805480	0.613014

20
MeOOCBn−

C	-4.749230	3.109172	0.029043
C	-3.510825	3.858455	-0.030641
C	-3.480352	5.221115	-0.107989
C	-4.668317	6.005812	-0.135615
C	-5.899968	5.292786	-0.078394
C	-5.942764	3.928991	-0.000814
C	-4.569948	7.427054	-0.216595
H	-6.829644	5.855038	-0.097225
H	-2.576402	3.299040	-0.012055
H	-2.528922	5.746498	-0.150972

H	-6.906926	3.423844	0.041064
C	-4.788465	1.736789	0.107612
H	-3.874771	1.150137	0.128091
H	-5.734071	1.204413	0.150921
O	-3.546686	8.113920	-0.269572
O	-5.824084	8.058956	-0.234153
C	-5.758127	9.475028	-0.313486
H	-6.795953	9.823749	-0.318675
H	-5.247510	9.806573	-1.226701
H	-5.223512	9.904649	0.543387

16

O2NBn−

C	-3.364506	2.361055	0.063825
C	-3.647715	1.026332	0.019330
C	-2.009789	2.869615	0.103335
C	-0.980000	1.852344	0.092413
C	-1.265348	0.518089	0.048126
C	-2.607238	0.064315	0.009975
H	-4.179823	3.082308	0.070230
H	-4.672343	0.671280	-0.009798
H	-0.474387	-0.224262	0.040817
H	0.058296	2.178115	0.120967
C	-1.723796	4.212419	0.147606
H	-2.516335	4.954402	0.154749
H	-0.697809	4.566247	0.176253
N	-2.897983	-1.302326	-0.036132
O	-1.951630	-2.133644	-0.042568
O	-4.100474	-1.675684	-0.070525

15

NCBn−

C	-3.192129	0.949849	-0.168817
C	-3.358989	-0.455770	-0.011530
C	-1.953954	1.525128	-0.237610
C	-0.727550	0.757948	-0.156257
C	-0.929070	-0.667998	0.002559
C	-2.170468	-1.236341	0.070695
H	-4.081960	1.573009	-0.234765
H	-1.873185	2.604204	-0.357783
H	-0.047320	-1.303100	0.069878
H	-2.265668	-2.313833	0.190757
C	0.520082	1.333517	-0.223610
C	-4.628710	-1.041412	0.060647
N	-5.690654	-1.531303	0.122583
H	0.636553	2.406455	-0.342248
H	1.419840	0.729293	-0.158613

19

MeOBnH

C	-4.788350	3.281291	-0.103095
C	-3.580294	3.955978	-0.242465
C	-3.518201	5.348252	-0.327690
C	-4.696358	6.090194	-0.272047
C	-5.921884	5.430420	-0.131803
C	-5.958703	4.049793	-0.049337
H	-6.830957	6.022340	-0.089334
H	-2.653306	3.388983	-0.287044
H	-2.552745	5.828808	-0.435849
H	-6.919955	3.551979	0.060059
C	-4.850431	1.780174	-0.013552
O	-4.763625	7.453089	-0.344561
C	-3.538569	8.161780	-0.483876
H	-3.806805	9.219098	-0.520390
H	-3.018787	7.886780	-1.412037
H	-2.873390	7.985028	0.372569
H	-3.850579	1.339012	-0.048672
H	-5.433037	1.356007	-0.839596
H	-5.327323	1.456907	0.918895

18

MeBnH

C	-4.889663	3.002765	-0.105045
C	-3.807122	3.885432	-0.070774
C	-4.003388	5.258286	0.012901
C	-5.289662	5.801747	0.060854
C	-6.370423	4.920498	0.026967
C	-6.173807	3.545344	-0.056541
C	-5.499458	7.291075	0.114077
H	-4.710682	7.783913	0.690608
H	-6.462521	7.541533	0.568350
H	-5.488112	7.725056	-0.893424
H	-7.383543	5.314824	0.064121
H	-2.794062	3.490223	-0.106860
H	-3.141720	5.921990	0.039096
H	-7.035785	2.882345	-0.081417
C	-4.670945	1.514811	-0.162130
H	-4.415096	1.117145	0.827831
H	-3.848567	1.259351	-0.837757
H	-5.569172	0.993281	-0.505255

15

FBnH

C	-2.996217	1.281943	0.002070
C	-3.056706	-0.100646	-0.020690
C	-1.745053	1.889063	0.047735
C	-0.569670	1.134393	0.068493
C	-0.678725	-0.258187	0.049046
C	-1.918620	-0.887877	0.003335
H	-3.912080	1.863597	-0.011396
H	-1.684922	2.974468	0.068568
H	0.222375	-0.866240	0.070788
H	-2.009032	-1.969075	-0.009264
C	0.778452	1.803636	0.081254
F	-4.268319	-0.702575	-0.063183
H	0.736615	2.774146	0.584092
H	1.139455	1.977961	-0.939792
H	1.524032	1.187239	0.591824

15

BnH

C	-6.638075	2.808879	0.206453
C	-7.619193	3.641740	-0.323073
C	-7.011702	1.621279	0.828378
C	-8.355184	1.270941	0.917703
C	-9.349284	2.096494	0.386959
C	-8.961541	3.287587	-0.231515
H	-7.339218	4.573799	-0.806091
H	-6.254401	0.966279	1.250164
H	-8.638731	0.342732	1.408841
H	-9.722097	3.946257	-0.644909
C	-10.799273	1.699494	0.455099
H	-5.589994	3.085459	0.139045
H	-11.002608	1.083435	1.335891
H	-11.084758	1.114699	-0.428048
H	-11.451433	2.577042	0.491811

25

PhBnH

C	-4.736360	4.526483	0.072598
C	-5.124144	3.180670	0.092301
C	-3.397432	4.879749	-0.044915
C	-2.417956	3.895748	-0.144689
C	-2.789961	2.554564	-0.126228

C	-4.128675	2.200390	-0.009213
H	-5.491482	5.300441	0.179837
H	-3.116296	5.929132	-0.048340
H	-1.371727	4.172315	-0.236415
H	-2.034064	1.779182	-0.213680
H	-4.413256	1.151805	-0.024931
C	-6.547183	2.804312	0.217304
C	-7.545959	3.524719	-0.448144
C	-6.940348	1.719807	1.010192
C	-8.279236	1.371544	1.130656
C	-9.275337	2.087720	0.463348
C	-8.883266	3.171262	-0.325730
H	-7.267790	4.356875	-1.089363
H	-6.189584	1.163881	1.565447
H	-8.557980	0.533390	1.765438
H	-9.638007	3.744022	-0.860063
C	-10.721461	1.687436	0.568031
H	-10.934838	1.213201	1.530484
H	-10.985194	0.968563	-0.217701
H	-11.383288	2.551352	0.456632

21
MeOOCBnH

C	-4.795877	3.159978	-0.069474
C	-3.715974	3.821280	-0.662870
C	-3.662204	5.205881	-0.702790
C	-4.692856	5.966394	-0.144420
C	-5.775729	5.316295	0.454814
C	-5.820106	3.929731	0.489756
C	-4.582640	7.443544	-0.211312
H	-6.577041	5.899167	0.895048
H	-2.906690	3.239677	-1.097676
H	-2.824056	5.721425	-1.160899
H	-6.664371	3.432309	0.961056
C	-4.868033	1.658948	-0.058419
H	-3.870125	1.211352	-0.049648
H	-5.384775	1.291755	-0.953872
H	-5.420153	1.292455	0.811721
O	-3.658612	8.043604	-0.719148
O	-5.640408	8.064440	0.363860
C	-5.587793	9.503503	0.327321
H	-6.497775	9.840952	0.824526
H	-5.557326	9.858852	-0.706596
H	-4.701240	9.867543	0.854352

16
NCBnH

C	-3.168848	0.938201	-0.181560
C	-3.321230	-0.444824	-0.017420
C	-1.899232	1.493467	-0.194451
C	-0.759157	0.697437	-0.043556
C	-0.925973	-0.681351	0.115650
C	-2.188089	-1.254615	0.131024
H	-4.047831	1.563081	-0.301334
H	-1.788695	2.566905	-0.325274
H	-0.051245	-1.316611	0.228324
H	-2.308230	-2.326127	0.252962
C	0.610327	1.314635	-0.025032
C	-4.621753	-1.024883	-0.007579
N	-5.679750	-1.496529	-0.001192
H	0.660033	2.194148	-0.673219
H	1.373990	0.601929	-0.347864
H	0.870698	1.640327	0.989645

32
MeOBnBn

C	-3.001813	4.102062	-0.244991
C	-4.234548	4.386311	-0.824100
C	-2.530651	2.789295	-0.170101
C	-3.325314	1.766082	-0.692810
C	-4.558450	2.045414	-1.272854
C	-5.017268	3.357849	-1.340617
H	-2.396008	4.909609	0.161084
H	-4.587214	5.412987	-0.868126
H	-5.165353	1.235975	-1.668943
H	-5.981447	3.577404	-1.789793
H	-2.973518	0.737800	-0.638494
C	-1.172053	2.490441	0.404384
C	-0.070280	2.513447	-0.679629
H	-1.171633	1.504853	0.886338
H	-0.917793	3.223902	1.179452
C	1.289373	2.207941	-0.111148
H	-0.069215	3.500273	-1.158976
H	-0.328477	1.784833	-1.458457
C	2.126334	3.222242	0.345625
C	1.730203	0.886806	0.028276
C	2.957326	0.590776	0.597233
C	3.783936	1.623658	1.049520
C	3.366030	2.948714	0.922590
H	1.811554	4.259210	0.247466
H	1.098841	0.073245	-0.323280
H	3.300875	-0.434092	0.697624
H	3.987942	3.770635	1.257813
O	4.975205	1.233224	1.591394
C	5.847472	2.253363	2.062530
H	6.150778	2.928448	1.250533
H	6.727533	1.738333	2.451491
H	5.382724	2.837825	2.868503

31
MeBnBn

C	-1.505847	0.773119	1.278706
C	-1.785548	-0.517804	1.715650
C	-1.807550	1.166848	-0.026694
C	-2.396360	0.234934	-0.885162
C	-2.677421	-1.056889	-0.452574
C	-2.371671	-1.437487	0.851364
H	-1.034022	1.484000	1.952974
H	-1.542620	-0.806496	2.734414
H	-3.140815	-1.766450	-1.132492
H	-2.592401	-2.444832	1.192508
H	-2.639421	0.527759	-1.904829
C	-1.444331	2.539403	-0.526142
C	-0.064586	2.562712	-1.231498
H	-1.424940	3.248664	0.310087
H	-2.204694	2.890343	-1.234911
C	1.062614	2.200360	-0.302192
H	0.099276	3.566894	-1.641899
H	-0.090260	1.865200	-2.077631
C	1.647831	3.165112	0.518396
C	1.506445	0.881310	-0.183391
C	2.502069	0.541707	0.723868
C	3.090740	1.507742	1.544069
C	2.645437	2.824938	1.426776
H	1.318665	4.200108	0.446275
H	1.053166	0.108593	-0.800174
H	2.827216	-0.493772	0.799255
H	3.088333	3.597175	2.051786
C	4.188453	1.135420	2.503837
H	3.940610	0.222964	3.056036
H	4.372557	1.933044	3.229081
H	5.128267	0.946736	1.970951

28

FBnBn

C	-3.051957	0.795528	-0.324407
C	-3.277806	-0.440180	0.257519
C	-1.739692	1.244263	-0.433947
C	-0.668625	0.476450	0.028948
C	-0.942606	-0.763580	0.610015
C	-2.246558	-1.233494	0.730429
H	-3.889829	1.388409	-0.676145
H	-1.546888	2.214561	-0.885975
H	-0.122489	-1.374402	0.980475
H	-2.468261	-2.193113	1.185684
C	0.751163	0.948993	-0.147689
F	-4.550148	-0.885859	0.372674
C	1.320590	0.502130	-1.499089
H	1.373489	0.552782	0.664312
H	0.787010	2.043337	-0.076841
C	2.741082	0.925896	-1.798030
H	0.663933	0.876920	-2.296934
H	1.256341	-0.593571	-1.560724
C	3.521313	1.660964	-0.905128
C	4.827264	2.025344	-1.230844
C	5.374601	1.660833	-2.454242
C	4.605283	0.926452	-3.354678
C	3.305351	0.565492	-3.027535
H	3.117764	1.958669	0.057714
H	5.416572	2.598280	-0.520330
H	6.391677	1.945582	-2.707041
H	5.020954	0.635278	-4.315295
H	2.710652	-0.007787	-3.736195

28
BnBn

C	-3.270514	4.060896	-0.315831
C	-4.497442	4.332726	-0.912643
C	-2.780572	2.754630	-0.248072
C	-3.550020	1.725218	-0.795384
C	-4.777660	1.992348	-1.392929
C	-5.255344	3.298265	-1.453811
H	-2.684473	4.872917	0.109877
H	-4.865178	5.354286	-0.951115
H	-5.365264	1.178185	-1.807881
H	-6.215067	3.508213	-1.916838
H	-3.183549	0.701864	-0.746467
C	-1.427947	2.470272	0.348152
C	-0.305118	2.554405	-0.710485
H	-1.413963	1.470197	0.798760
H	-1.209943	3.184659	1.151709
C	1.047989	2.268066	-0.116327
H	-0.318379	3.554958	-1.160014
H	-0.524366	1.841030	-1.514635
C	1.824688	3.297149	0.421056
C	1.531441	0.959679	-0.041056
C	2.759088	0.685655	0.553238
C	3.524282	1.719954	1.084414
C	3.053084	3.027844	1.016090
H	1.463120	4.321956	0.366244
H	0.940009	0.147494	-0.458864
H	3.121585	-0.337548	0.597457
H	4.484645	1.508308	1.545336
H	3.646406	3.841758	1.423317

38
PhBnBn

C	-1.091555	0.768860	1.461423
C	-1.292800	-0.538570	1.892626
C	-1.529744	1.181452	0.201401
C	-2.175236	0.251776	-0.617895
C	-2.378571	-1.056310	-0.190822
C	-1.936355	-1.455917	1.067563
H	-0.575949	1.478560	2.104228
H	-0.944178	-0.841261	2.876010
H	-2.887843	-1.764051	-0.839043
H	-2.096122	-2.476212	1.404152
H	-2.525358	0.559095	-1.601503
C	-1.252833	2.574065	-0.298193
C	0.043830	2.647443	-1.144755
H	-1.160082	3.263849	0.549205
H	-2.091265	2.922631	-0.913849
C	1.267973	2.296821	-0.343673
H	0.140280	3.663301	-1.547518
H	-0.053060	1.965050	-1.997753
C	1.909812	3.262583	0.435018
C	1.754231	0.988838	-0.300800
C	2.844284	0.656087	0.492239
C	3.488081	1.623120	1.273056
C	3.000682	2.935300	1.228808
H	1.543764	4.287456	0.424690
H	1.267654	0.219562	-0.895804
H	3.219621	-0.363601	0.487050
H	3.467997	3.700375	1.842985
C	4.649087	1.271849	2.116178
C	5.742994	2.139464	2.229656
C	4.683483	0.063150	2.823381
C	5.775289	-0.266192	3.617484
C	6.834199	1.809486	3.024312
C	6.855760	0.605276	3.722135
H	3.832246	-0.610278	2.770083
H	5.778284	-1.204363	4.165313
H	5.748092	3.068075	1.665304
H	7.676976	2.491779	3.090996
H	7.708838	0.347431	4.342813

34
MeOOCBnBn

C	-3.155657	1.675624	-0.748715
C	-4.369549	1.952742	-1.369194
C	-2.368672	2.703681	-0.224866
C	-2.825723	4.018444	-0.339086
C	-4.039012	4.300060	-0.958806
C	-4.814923	3.267033	-1.476509
H	-2.814624	0.645870	-0.663108
H	-4.971795	1.140111	-1.765630
H	-4.382022	5.328169	-1.033411
H	-5.764081	3.484735	-1.957349
H	-2.225535	4.829506	0.068392
C	-1.030659	2.406532	0.397538
C	0.105356	2.429158	-0.651528
H	-1.046292	1.421543	0.879956
H	-0.804179	3.141392	1.179804
C	1.440627	2.129434	-0.028997
H	0.122393	3.414732	-1.132272
H	-0.122038	1.694196	-1.433286
C	2.242768	3.157895	0.472835
C	1.882018	0.809889	0.110883
C	3.088458	0.524704	0.730742
C	3.884433	1.560189	1.228227
C	3.452786	2.883152	1.094728
H	1.914258	4.189548	0.368153
H	1.270968	-0.001684	-0.277948
H	3.437989	-0.497420	0.836914
H	4.068186	3.691084	1.474396
C	5.168179	1.198789	1.877624
O	5.848937	2.286287	2.311300
O	5.575742	0.064306	2.013204

C	7.105491	1.994924	2.952983
H	6.948330	1.365850	3.833736
H	7.517664	2.963117	3.238963
H	7.776907	1.477357	2.261947

29

NCBnBn

C	-2.312387	1.155874	-0.035166
C	-3.176724	0.091080	0.251816
C	-1.041258	0.896150	-0.522499
C	-0.601963	-0.414440	-0.733204
C	-1.475026	-1.467225	-0.443282
C	-2.749832	-1.227173	0.044889
H	-2.645258	2.175123	0.131598
H	-0.372570	1.725611	-0.739780
H	-1.146507	-2.492079	-0.598566
H	-3.420608	-2.048913	0.273487
C	0.756486	-0.682866	-1.316648
C	-4.482606	0.347552	0.758979
N	-5.545136	0.555743	1.170665
C	0.718940	-0.703755	-2.862939
H	1.139177	-1.646566	-0.959766
H	1.468505	0.085174	-0.991423
C	2.079671	-0.975220	-3.446526
C	2.514886	-2.285417	-3.657555
C	2.953444	0.074395	-3.737932
C	3.790912	-2.541498	-4.148644
C	4.230420	-0.177511	-4.229280
C	4.653288	-1.487306	-4.435480
H	0.337209	0.260317	-3.220988
H	0.005354	-1.470468	-3.189053
H	1.843071	-3.113461	-3.440500
H	5.648485	-1.685460	-4.822425
H	4.111139	-3.566476	-4.312901
H	2.626197	1.100692	-3.584004
H	4.895008	0.651202	-4.456537

25

Ph2H2Si

C	-2.804597	2.762610	0.728498
C	-3.090209	1.406448	0.596568
C	-1.705363	3.335273	0.075366
C	-0.894569	2.503588	-0.710253
C	-1.176431	1.148239	-0.843718
C	-2.276386	0.597788	-0.190578
H	-3.444815	3.382384	1.352670
H	-3.945930	0.979744	1.112423
H	-0.536419	0.519065	-1.456035
H	-2.496107	-0.461375	-0.292419
H	-0.025591	2.918767	-1.217236
Si	-1.333285	5.167564	0.227636
C	0.513434	5.451236	0.395158
C	1.292228	4.622309	1.215420
C	1.150269	6.499722	-0.281335
C	2.517761	6.718816	-0.139060
C	3.273341	5.888065	0.682379
C	2.658588	4.837750	1.359355
H	0.826429	3.791269	1.741672
H	0.572204	7.152474	-0.931985
H	2.994504	7.536015	-0.673216
H	4.341148	6.055834	0.792389
H	3.246185	4.184509	1.998450
H	-2.074058	5.683819	1.411472
H	-1.801008	5.920434	-0.968680

25

Ph2H2Si•−

Si	-1.103983	-0.016742	-0.046699
C	-2.211086	1.480909	-0.037319
C	0.693336	0.422421	-0.047033
H	-1.297193	-0.954831	-1.214825
H	-1.611120	-0.874758	1.096732
C	1.223966	1.503716	0.714879
C	1.647307	-0.388243	-0.729650
C	3.003603	-0.121167	-0.682686
C	2.578682	1.774352	0.755465
C	3.501532	0.966762	0.058655
C	-1.794716	2.754850	-0.480532
C	-3.592315	1.339382	0.292103
C	-4.473082	2.409059	0.191388
C	-2.667790	3.827466	-0.563811
C	-4.033762	3.665042	-0.230947
H	-4.727605	4.497529	-0.312397
H	-5.521451	2.265338	0.451778
H	-0.752622	2.893469	-0.764508
H	-2.300195	4.797806	-0.891156
H	-3.961570	0.374542	0.637154
H	0.540222	2.137288	1.278022
H	2.936882	2.619582	1.341331
H	4.567708	1.168948	0.106394
H	1.294177	-1.235360	-1.316602
H	3.693768	-0.760035	-1.232170

43

Ph2H2(MeOBn)Si−

C	-2.479933	4.452442	0.279922
C	-3.125718	3.613152	1.189154
C	-1.398639	4.021316	-0.497037
C	-0.999746	2.688499	-0.324852
C	-1.630604	1.833276	0.575616
C	-2.701829	2.295100	1.340979
H	-2.828746	5.480437	0.161928
H	-3.964043	3.983192	1.778152
H	-1.292768	0.803457	0.684454
H	-3.200986	1.633044	2.045558
H	-0.164229	2.310743	-0.918134
Si	-0.443681	5.248402	-1.763990
C	1.053114	5.480087	-0.592126
C	2.328778	5.008500	-0.930641
C	0.931931	6.177101	0.617220
C	2.032061	6.417355	1.436894
C	3.288971	5.933561	1.081617
C	3.431509	5.216798	-0.104514
H	2.465283	4.474221	-1.869811
H	-0.045810	6.546057	0.921453
H	1.908473	6.983153	2.358052
H	4.148997	6.111658	1.723292
H	4.406903	4.827258	-0.390167
C	0.376185	6.530574	-3.195555
H	-1.589026	6.248262	-1.630858
C	0.463868	7.910463	-2.699398
H	-0.319864	6.441907	-4.039945
H	1.352167	6.130235	-3.494801
C	1.614175	8.407639	-2.065964
C	-0.657820	8.765769	-2.705420
C	-0.625851	10.031009	-2.136773
C	0.536611	10.494166	-1.522886
C	1.663318	9.674463	-1.486927
H	2.494536	7.771889	-2.003765
H	-1.576564	8.417734	-3.171172
H	-1.501180	10.675161	-2.154486
H	2.582165	9.995352	-1.007296
H	-0.253194	4.105520	-2.752942
O	0.472567	11.772052	-0.985153

C	1.621231	12.226834	-0.300915
H	1.879898	11.570643	0.544350
H	1.377513	13.223009	0.079070
H	2.494667	12.300773	-0.967816

42

Ph2H2(MeBn)Si−

C	-5.085217	1.111425	-0.589867
C	-4.970905	0.024832	-1.467352
C	-4.897137	-1.287281	-1.004205
C	-4.963712	-1.549303	0.362382
C	-5.083301	-0.486024	1.253885
C	-5.126154	0.823021	0.780496
H	-4.948998	0.208308	-2.540647
H	-4.797036	-2.108930	-1.710971
H	-4.922014	-2.572521	0.728856
H	-5.142315	-0.678084	2.323190
H	-5.198474	1.642587	1.493015
C	-3.383281	4.245246	0.696454
C	-3.475126	3.467026	-0.463031
C	-2.156442	4.609723	1.253593
C	-0.969771	4.202170	0.649167
C	-1.028229	3.430746	-0.511918
C	-2.263226	3.077507	-1.050305
H	-4.306807	4.579583	1.173389
H	-2.122505	5.216174	2.157839
H	-0.009016	4.483669	1.075251
H	-0.106153	3.108901	-0.994231
H	-2.292192	2.475004	-1.960706
Si	-5.239297	2.907475	-1.231433
C	-7.121201	2.529806	-2.086686
H	-5.941134	3.911300	-0.324215
H	-4.701365	3.083416	-2.644180
C	-8.130485	2.251578	-1.063009
H	-7.006425	1.708907	-2.803922
H	-7.326887	3.460863	-2.629804
C	-8.768893	3.290212	-0.357314
C	-8.423754	0.940066	-0.636522
C	-9.301914	0.689152	0.407332
C	-9.936489	1.729528	1.095055
C	-9.649143	3.033332	0.688451
H	-8.566302	4.318615	-0.647170
H	-7.932031	0.106803	-1.133932
H	-9.496869	-0.341900	0.701655
H	-10.124702	3.868598	1.202296
C	-10.861266	1.449127	2.251218
H	-11.496130	0.576877	2.054737
H	-11.516835	2.303934	2.450876
H	-10.304689	1.241154	3.175568

39

Ph2H2(FBn)Si−

C	-3.174548	0.631800	-1.213493
C	-3.208137	-0.526124	-0.456355
C	-2.071365	1.471862	-1.101787
C	-0.994438	1.186425	-0.237082
C	-1.076811	-0.017185	0.493166
C	-2.172375	-0.869186	0.394316
H	-4.000648	0.866875	-1.878459
H	-2.033651	2.379065	-1.699737
H	-0.256699	-0.282573	1.155186
H	-2.227545	-1.790049	0.967806
C	0.205029	2.022992	-0.204988
F	-4.298981	-1.352980	-0.547839
Si	1.549441	1.319843	-1.662252
H	0.737190	1.957375	0.750823
H	-0.020294	3.071105	-0.428799
C	2.858226	2.634442	-1.143502
C	4.140634	2.238020	-0.737401
C	5.098610	3.160804	-0.324522
C	4.809955	4.523564	-0.343627
C	3.552317	4.946021	-0.766793
C	2.590389	4.009989	-1.143308
C	2.762771	0.528940	-3.046004
C	3.277977	-0.766651	-2.932847
C	3.135081	1.250171	-4.189777
C	3.979238	0.719357	-5.161279
C	4.482015	-0.574502	-5.016459
C	4.126362	-1.318511	-3.895097
H	2.753922	2.265342	-4.318841
H	4.249604	1.309775	-6.035589
H	5.141282	-0.996490	-5.772099
H	4.507793	-2.331538	-3.773823
H	2.999223	-1.364079	-2.062739
H	1.606866	4.360947	-1.449504
H	5.560018	5.249349	-0.037668
H	3.318198	6.008381	-0.797977
H	4.399039	1.180622	-0.765812
H	6.079601	2.817849	-0.001760
H	1.531984	-0.011878	-0.924989
H	0.567371	1.770198	-2.726765

39

Ph2H2BnSi−

C	-2.510415	4.349276	0.253451
C	-3.156578	3.482745	1.136603
C	-1.408577	3.950530	-0.511745
C	-0.988252	2.622229	-0.355051
C	-1.619610	1.740436	0.518841
C	-2.712040	2.169998	1.272869
H	-2.875102	5.372946	0.147839
H	-4.010950	3.827907	1.717498
H	-1.265076	0.715144	0.616036
H	-3.211279	1.487087	1.957157
H	-0.134816	2.269693	-0.938091
Si	-0.456993	5.214426	-1.739145
C	1.031820	5.428448	-0.556614
C	2.310896	4.969488	-0.899538
C	0.899518	6.091562	0.670239
C	1.993124	6.310497	1.504501
C	3.253345	5.839109	1.145210
C	3.406712	5.156767	-0.059676
H	2.455395	4.461558	-1.852010
H	-0.081100	6.450464	0.977083
H	1.861847	6.851338	2.439347
H	4.108257	6.001736	1.797657
H	4.385193	4.778213	-0.349298
C	0.375566	6.549339	-3.144006
H	-1.614395	6.196197	-1.613866
C	0.425691	7.913520	-2.621366
H	-0.312909	6.453590	-3.992742
H	1.361614	6.163012	-3.425081
C	1.562732	8.418466	-1.955852
C	-0.713136	8.746675	-2.641719
C	-0.708534	10.006107	-2.053912
C	0.431988	10.488587	-1.413708
C	1.566345	9.676076	-1.368898
H	2.449527	7.791604	-1.890033
H	-1.612916	8.383918	-3.133218
H	-1.607250	10.618933	-2.095191
H	0.435938	11.473542	-0.954173
H	2.464597	10.025928	-0.863882
H	-0.249967	4.109850	-2.762833

49
Ph2H2(PhBn)Si−

C	-5.212343	1.144534	-0.706155
C	-4.918341	0.105168	-1.599055
C	-4.952735	-1.229938	-1.203444
C	-5.308241	-1.561661	0.102208
C	-5.609963	-0.545699	1.005178
C	-5.547105	0.787084	0.606014
H	-4.668278	0.345248	-2.631492
H	-4.710553	-2.014969	-1.917105
H	-5.351276	-2.603113	0.412287
H	-5.899092	-0.792567	2.024260
H	-5.774638	1.567490	1.329768
C	-3.689479	4.219710	0.952892
C	-3.606732	3.474388	-0.228823
C	-2.560304	4.550629	1.703206
C	-1.298872	4.141888	1.278485
C	-1.183679	3.402146	0.101451
C	-2.322926	3.080697	-0.631948
H	-4.670910	4.555377	1.292297
H	-2.661602	5.131318	2.618833
H	-0.413362	4.397511	1.856389
H	-0.201638	3.079240	-0.240655
H	-2.214524	2.501520	-1.550953
Si	-5.225474	2.966597	-1.266915
C	-7.010138	2.620994	-2.444267
H	-6.070676	3.949670	-0.480206
H	-4.524250	3.193088	-2.590152
C	-8.130505	2.305743	-1.583625
H	-6.758020	1.829833	-3.156569
H	-7.107014	3.587040	-2.950856
C	-8.856284	3.318988	-0.914654
C	-8.475273	0.975387	-1.249482
C	-9.476592	0.683955	-0.343305
C	-10.198688	1.699563	0.310721
C	-9.851369	3.025175	0.000926
H	-8.628881	4.357719	-1.141937
H	-7.911193	0.162361	-1.701288
H	-9.677275	-0.356910	-0.098924
H	-10.404783	3.842724	0.458448
C	-11.264994	1.389395	1.269092
C	-11.531785	2.227327	2.368032
C	-12.066280	0.240773	1.130053
C	-12.546212	1.936909	3.270716
C	-13.326783	0.792414	3.117372
C	-13.073723	-0.053882	2.039286
H	-11.907438	-0.411390	0.275947
H	-14.117087	0.562991	3.826735
H	-13.678280	-0.946681	1.897052
H	-10.907379	3.101870	2.526825
H	-12.718715	2.602243	4.113528

42
Ph2H2(F3CBn)Si−

C	-2.655754	4.491957	-0.052622
C	-3.429264	3.684758	0.782769
C	-1.460981	4.041592	-0.625313
C	-1.078256	2.726704	-0.324311
C	-1.836568	1.905231	0.505588
C	-3.021122	2.384136	1.065491
H	-2.992263	5.506371	-0.273128
H	-4.353680	4.067730	1.212246
H	-1.509024	0.889034	0.719268
H	-3.619206	1.747964	1.714273
H	-0.153773	2.335913	-0.753525
Si	-0.344188	5.209810	-1.779616
C	1.025555	5.468793	-0.480025
C	2.312007	4.939781	-0.648581
C	0.794903	6.243764	0.663824
C	1.806624	6.501579	1.585434
C	3.076237	5.961332	1.400066
C	3.323933	5.169515	0.280780
H	2.530936	4.344009	-1.533576
H	-0.194894	6.663561	0.833068
H	1.604164	7.129340	2.450240
H	3.866919	6.156627	2.120637
H	4.311004	4.737749	0.127851
C	0.716423	6.493440	-3.185094
H	-1.453017	6.239615	-1.863927
C	0.753237	7.848726	-2.688517
H	0.090855	6.368731	-4.074527
H	1.701450	6.040674	-3.328014
C	1.830835	8.333405	-1.905634
C	-0.352877	8.719179	-2.838742
C	-0.376425	9.982045	-2.271371
C	0.706909	10.435811	-1.514557
C	1.812264	9.590427	-1.337605
H	2.684889	7.682491	-1.735233
H	-1.206496	8.379301	-3.419293
H	-1.241803	10.622492	-2.413329
H	2.656528	9.926361	-0.740459
H	-0.051774	4.090904	-2.754954
C	0.733726	11.796029	-0.931049
F	1.637684	12.631437	-1.544880
F	1.098036	11.804714	0.387077
F	-0.463941	12.435156	-1.003035

45
Ph2H2(MeOOCBn)Si−

C	-5.281599	1.165186	-0.559054
C	-5.084145	0.046979	-1.379831
C	-5.192836	-1.249585	-0.883382
C	-5.523933	-1.459237	0.453721
C	-5.729736	-0.362323	1.285518
C	-5.597083	0.930429	0.785252
H	-4.853039	0.192717	-2.434069
H	-5.027632	-2.099339	-1.542560
H	-5.624092	-2.469803	0.842452
H	-6.001489	-0.513650	2.327605
H	-5.755864	1.775320	1.452560
C	-3.438907	4.289965	0.731211
C	-3.486559	3.413040	-0.358524
C	-2.241936	4.607630	1.374538
C	-1.045528	4.049952	0.932567
C	-1.061400	3.174945	-0.153538
C	-2.265525	2.868679	-0.781638
H	-4.367771	4.740752	1.083311
H	-2.240565	5.293257	2.220170
H	-0.108408	4.294193	1.427910
H	-0.131041	2.733994	-0.507358
H	-2.259762	2.180743	-1.628819
Si	-5.187049	2.936156	-1.241210
C	-7.134724	2.592576	-2.317977
H	-5.946644	4.025565	-0.527089
H	-4.611443	3.023596	-2.632164
C	-8.183221	2.430177	-1.357599
H	-6.939594	1.726162	-2.953133
H	-7.169105	3.527424	-2.883001
C	-8.796507	3.551596	-0.734415
C	-8.575878	1.153167	-0.873520
C	-9.509527	1.006788	0.127478
C	-10.116923	2.132164	0.722784
C	-9.729929	3.407119	0.267751
H	-8.514521	4.547407	-1.068018

H	-8.106596	0.270219	-1.301623
H	-9.780819	0.012548	0.467509
H	-10.190396	4.278034	0.726124
C	-11.114559	2.037946	1.774983
O	-11.396076	0.733273	2.125291
C	-12.366431	0.597155	3.163594
H	-12.031192	1.086703	4.084879
H	-12.477792	-0.477877	3.325389
H	-13.325560	1.038507	2.869960
O	-11.679503	2.970412	2.329335

41

Ph2H2(O2NBn)Si−

C	-1.467478	3.726160	1.004713
C	-2.171208	2.798812	1.768521
C	-1.386152	3.609292	-0.389447
C	-2.032279	2.519799	-0.988759
C	-2.739230	1.588309	-0.232494
C	-2.810571	1.726874	1.151102
H	-0.960696	4.552434	1.499850
H	-2.217980	2.910544	2.848991
H	-3.230852	0.750722	-0.721560
H	-3.358431	1.000225	1.745874
H	-1.977488	2.393852	-2.069230
Si	-0.462252	4.899701	-1.440059
C	0.907643	5.589307	-0.369927
C	1.941026	4.752196	0.081310
C	0.900017	6.922267	0.058445
C	1.891361	7.405755	0.909103
C	2.907629	6.562939	1.346067
C	2.933588	5.232603	0.927421
H	1.966069	3.708734	-0.229901
H	0.128300	7.601935	-0.294093
H	1.875361	8.451075	1.204998
H	3.685078	6.942698	2.004226
H	3.729070	4.571031	1.262125
C	0.758058	6.867193	-3.683659
H	-1.439876	5.951062	-1.812604
C	0.733912	8.072462	-3.018601
H	-0.134638	6.473777	-4.159252
H	1.688261	6.326818	-3.828995
C	1.916810	8.654505	-2.430772
C	-0.484781	8.821026	-2.813457
C	-0.521645	9.969925	-2.073675
C	0.657675	10.485972	-1.485169
C	1.879881	9.806012	-1.697102
H	2.861775	8.131191	-2.560179
H	-1.403034	8.435429	-3.251746
H	-1.450742	10.507685	-1.918723
H	2.780174	10.214267	-1.250799
H	0.004186	4.131396	-2.624636
N	0.615537	11.652215	-0.704938
O	1.675116	12.071126	-0.175193
O	-0.480057	12.245359	-0.549089

40

Ph2H2(NCBn)Si−

C	-2.982203	0.352637	-0.829349
C	-2.960292	-0.817724	-0.042278
C	-1.948402	1.260259	-0.768211
C	-0.825506	1.070954	0.084055
C	-0.814521	-0.129392	0.847009
C	-1.843983	-1.041570	0.791799
H	-3.831681	0.532201	-1.482873
H	-1.985446	2.155708	-1.383348
H	0.037815	-0.323304	1.493672
H	-1.809849	-1.944349	1.395750
C	0.288014	1.961491	0.067561
C	-4.032281	-1.736770	-0.080778
Si	1.636877	1.212169	-1.604880
H	0.923616	1.930078	0.953489
H	0.076665	2.979651	-0.264083
C	2.775543	2.697836	-1.223117
C	3.437795	2.850783	0.001657
C	4.276188	3.935783	0.247589
C	4.452001	4.917325	-0.724680
C	3.798176	4.790377	-1.947891
C	2.986533	3.686095	-2.194698
C	2.710003	0.545873	-3.119464
C	2.138157	0.317667	-4.375953
C	4.074950	0.250019	-2.986348
C	4.830070	-0.246673	-4.044808
C	4.232085	-0.464448	-5.286191
C	2.879766	-0.179854	-5.448805
H	4.563494	0.423965	-2.026413
H	5.887932	-0.463374	-3.907616
H	4.816965	-0.851974	-6.117355
H	2.402130	-0.347098	-6.412541
H	1.078313	0.532647	-4.516647
H	2.517867	3.578848	-3.171766
H	5.096960	5.771401	-0.532623
H	3.932864	5.547667	-2.717187
H	3.298171	2.103581	0.780650
H	4.790144	4.018931	1.202801
H	1.844897	0.004863	-0.736940
H	0.412592	1.410329	-2.459557
N	-4.918940	-2.490866	-0.108725

42

Ph2H(MeOBn)Si

C	-1.717709	1.622998	2.677682
C	-2.611770	2.165542	1.757606
C	-0.798112	0.663209	2.270097
C	-0.751400	0.222526	0.938706
C	-1.658364	0.781367	0.029436
C	-2.580917	1.743900	0.432537
C	2.564676	0.808282	1.090454
C	3.867081	1.193231	1.396759
C	2.229048	-0.543608	0.938237
C	3.241516	-1.500199	1.101948
C	4.544025	-1.119263	1.406313
C	4.858598	0.229145	1.554358
H	1.796001	1.570228	0.976376
H	4.108007	2.246202	1.514888
H	3.010959	-2.556839	0.979844
H	5.314556	-1.875522	1.528689
H	5.875126	0.528063	1.795566
H	-1.648834	0.464909	-1.011119
H	-3.275360	2.165323	-0.289047
H	-1.737463	1.950394	3.713518
H	-3.330395	2.916521	2.073906
H	-0.099802	0.253915	2.998128
Si	0.505002	-1.076232	0.424235
C	0.460989	-1.371735	-1.450679
C	1.547866	-2.338608	-1.834567
H	0.595467	-0.412717	-1.965905
H	-0.526026	-1.760725	-1.731736
C	2.805596	-1.890809	-2.233791
C	1.357195	-3.722113	-1.720542
C	3.844620	-2.774726	-2.519363
C	3.630519	-4.147558	-2.399664
C	2.376534	-4.617409	-1.998897
H	0.389047	-4.104359	-1.404312
H	2.991981	-0.822180	-2.310970

H	4.805384	-2.377224	-2.825176
H	2.222331	-5.688641	-1.914183
H	0.211023	-2.376219	1.090332
O	4.571511	-5.107271	-2.649434
C	5.861518	-4.667519	-3.054799
H	5.817359	-4.109708	-4.000429
H	6.455314	-5.572158	-3.197217
H	6.332665	-4.040999	-2.284693

38

Ph2HBnSi

C	-2.001354	1.574172	2.585275
C	-2.821589	2.090618	1.584869
C	-0.992023	0.674795	2.261002
C	-0.780125	0.269498	0.934336
C	-1.615089	0.801194	-0.056501
C	-2.627060	1.703345	0.263136
C	2.450248	1.087363	1.365149
C	3.690186	1.568975	1.775527
C	2.234455	-0.285171	1.182692
C	3.301840	-1.162321	1.424080
C	4.542347	-0.684277	1.832696
C	4.738011	0.683007	2.009081
H	1.635850	1.788342	1.192777
H	3.838402	2.636292	1.915314
H	3.164906	-2.232647	1.282153
H	5.357641	-1.379281	2.013628
H	5.706001	1.057375	2.330956
H	-1.478148	0.512000	-1.096002
H	-3.263116	2.104948	-0.521076
H	-2.148867	1.874680	3.618974
H	-3.609932	2.794734	1.836192
H	-0.353263	0.285928	3.052156
Si	0.600024	-0.941396	0.538445
C	0.716609	-1.244258	-1.333832
C	1.873715	-2.154370	-1.633433
H	0.839796	-0.279804	-1.841458
H	-0.227481	-1.684536	-1.678948
C	3.135268	-1.633138	-1.936878
C	1.734187	-3.543460	-1.542287
C	4.222651	-2.473514	-2.148743
C	4.071344	-3.853940	-2.056994
C	2.820874	-4.385385	-1.753267
H	0.761740	-3.966881	-1.300451
H	3.266739	-0.555144	-1.993800
H	5.193577	-2.046188	-2.383223
H	4.920103	-4.510845	-2.222792
H	2.689374	-5.461509	-1.681837
H	0.342816	-2.257037	1.187986

21

MeOBnCO2−

C	-3.125455	0.585705	-2.540073
C	-4.227255	0.972345	-3.626192
C	-5.464543	0.139410	-3.572159
H	-3.726279	0.832340	-4.594111
H	-4.471972	2.033989	-3.522429
C	-5.428644	-1.238676	-3.848772
C	-6.693582	0.681115	-3.196819
C	-7.854569	-0.093461	-3.102409
C	-7.788739	-1.452333	-3.394380
C	-6.568637	-2.021538	-3.767039
H	-8.783701	0.379424	-2.802090
H	-6.750546	1.741736	-2.960563
H	-6.534200	-3.086856	-3.979417
H	-4.469909	-1.684827	-4.092272
O	-8.868493	-2.318732	-3.347499
C	-10.106577	-1.769249	-2.946606
H	-10.826185	-2.592471	-2.960813
H	-10.447220	-0.981026	-3.635919
H	-10.057650	-1.350356	-1.929722
O	-2.624921	1.557862	-1.935822
O	-2.868889	-0.636587	-2.460155

20

MeBnCO2−

C	-3.530889	-0.259488	-1.886885
C	-3.981520	-0.458453	-3.407913
C	-5.457261	-0.559153	-3.590940
H	-3.479517	-1.341279	-3.815035
H	-3.600523	0.429079	-3.930680
C	-6.064555	-1.754209	-3.990313
C	-6.300004	0.532298	-3.323769
C	-7.676582	0.424966	-3.462531
C	-8.279783	-0.772002	-3.866572
C	-7.447599	-1.860015	-4.126124
H	-8.304602	1.289231	-3.246762
H	-5.845492	1.452488	-2.970572
H	-7.885925	-2.806739	-4.440126
H	-5.437025	-2.619373	-4.195110
C	-9.778154	-0.882574	-3.984345
O	-4.097720	0.683431	-1.292069
O	-2.642126	-1.046118	-1.499013
H	-10.206687	-0.007699	-4.487753
H	-10.255317	-0.952704	-2.997595
H	-10.066397	-1.773382	-4.552661

17

FBnCO2−

C	-3.267940	0.223633	-1.934481
C	-3.755916	-0.317095	-3.356370
C	-5.218457	-0.594440	-3.439410
H	-3.175754	-1.207663	-3.615737
H	-3.486409	0.480670	-4.061674
C	-5.703846	-1.897193	-3.598900
C	-6.158577	0.440213	-3.305383
C	-7.525340	0.185539	-3.344078
C	-7.955260	-1.120642	-3.511137
C	-7.070839	-2.174762	-3.636391
H	-8.254687	0.983369	-3.238099
H	-5.789896	1.445124	-3.129371
H	-7.445283	-3.186776	-3.758116
H	-4.994005	-2.716433	-3.688603
F	-9.301100	-1.375660	-3.555180
O	-3.973544	1.133445	-1.445582
O	-2.215762	-0.300671	-1.514984

17

BnCO2−

C	-3.078317	0.715971	-2.490245
C	-4.254708	1.057451	-3.521696
C	-5.393882	0.100262	-3.461189
H	-3.782034	1.013118	-4.511558
H	-4.597601	2.080864	-3.343146
C	-5.240160	-1.235151	-3.868804
C	-6.640023	0.484203	-2.948169
C	-7.696469	-0.418395	-2.854126
C	-7.532315	-1.736213	-3.273358
C	-6.293511	-2.136188	-3.777348
H	-8.652331	-0.090150	-2.450778
H	-6.773676	1.510523	-2.612121
H	-6.147493	-3.166146	-4.096977
H	-4.262909	-1.550599	-4.220432
H	-8.354894	-2.443949	-3.203791

O	-2.742610	1.663481	-1.750146
O	-2.623781	-0.444658	-2.584926

27

PhBnCO2−

C	-3.663179	0.053292	-2.432707
C	-4.683661	0.717607	-3.487129
C	-5.982368	0.004048	-3.541766
H	-4.170665	0.646077	-4.453932
H	-4.824088	1.771185	-3.230762
C	-6.080018	-1.288262	-4.085855
C	-7.148264	0.560987	-2.996895
C	-8.356741	-0.121243	-3.012145
C	-8.454594	-1.405097	-3.567451
C	-7.283270	-1.973196	-4.096646
H	-9.232282	0.332501	-2.552955
H	-7.093165	1.546102	-2.538792
H	-7.329611	-2.958709	-4.555043
H	-5.178421	-1.746639	-4.479429
C	-9.734820	-2.129644	-3.593997
O	-3.254307	0.831327	-1.549445
O	-3.399274	-1.144603	-2.661869
C	-10.953135	-1.452861	-3.773130
C	-9.786290	-3.525585	-3.441684
C	-10.994914	-4.209799	-3.471837
C	-12.193716	-3.521774	-3.647033
C	-12.162164	-2.136795	-3.795102
H	-10.940775	-0.377101	-3.924777
H	-13.138915	-4.057237	-3.667175
H	-13.087542	-1.585321	-3.943138
H	-8.863131	-4.071043	-3.267770
H	-11.002311	-5.289349	-3.342454

20

F3CBnCO2−

C	-3.276543	0.532591	-2.030602
C	-3.844162	0.262169	-3.517328
C	-5.228736	-0.262054	-3.513625
H	-3.164526	-0.417646	-4.037758
H	-3.810408	1.245114	-4.002966
C	-5.504886	-1.594433	-3.855788
C	-6.306265	0.542293	-3.099940
C	-7.596687	0.042854	-3.053642
C	-7.851568	-1.285843	-3.410703
C	-6.794447	-2.104809	-3.811197
H	-8.414556	0.679811	-2.728959
H	-6.092768	1.557057	-2.781397
H	-6.982514	-3.139540	-4.082388
H	-4.682947	-2.239739	-4.156229
C	-9.246294	-1.799868	-3.435741
O	-3.996655	1.276022	-1.334719
O	-2.180698	-0.009681	-1.796272
F	-10.047022	-1.209360	-2.508810
F	-9.320607	-3.141378	-3.218279
F	-9.869683	-1.591993	-4.641314

23

MeOOCBnCO2−

C	-3.525015	-0.438845	-1.919218
C	-3.996422	-0.351661	-3.472039
C	-5.443402	-0.605261	-3.607062
H	-3.410217	-1.066014	-4.055137
H	-3.750685	0.671139	-3.778328
C	-5.926248	-1.836945	-4.083863
C	-6.391662	0.352803	-3.196082
C	-7.745905	0.100695	-3.287403
C	-8.217870	-1.125989	-3.786714
C	-7.282091	-2.098276	-4.175091
H	-8.472847	0.844359	-2.974199
H	-6.027674	1.286524	-2.779251
H	-7.627791	-3.054727	-4.552990
H	-5.208444	-2.597003	-4.383189
C	-9.663051	-1.333057	-3.881871
O	-4.034928	0.431046	-1.189851
O	-2.707825	-1.347591	-1.688230
O	-10.527213	-0.527302	-3.580411
O	-9.987569	-2.576836	-4.366604
C	-11.392362	-2.823246	-4.472107
H	-11.879202	-2.746641	-3.493906
H	-11.485005	-3.838554	-4.864216
H	-11.867271	-2.107143	-5.151582

19

O2NBnCO2−

C	-3.628022	-0.516672	-1.987215
C	-4.149283	-0.410538	-3.540208
C	-5.587179	-0.696596	-3.617961
H	-3.563323	-1.104210	-4.146301
H	-3.931702	0.623777	-3.824225
C	-6.059680	-1.945016	-4.068319
C	-6.539544	0.243281	-3.169866
C	-7.891049	-0.032234	-3.204343
C	-8.327219	-1.272517	-3.684927
C	-7.409291	-2.237743	-4.108353
H	-8.624150	0.690811	-2.866469
H	-6.180390	1.186595	-2.771343
H	-7.772434	-3.194055	-4.465941
H	-5.337714	-2.690559	-4.391237
N	-9.740924	-1.557455	-3.750429
O	-4.103749	0.358246	-1.247032
O	-2.825435	-1.443703	-1.799163
O	-10.537044	-0.683180	-3.382756
O	-10.101075	-2.664807	-4.173309

18

NCBnCO2−

C	-3.382613	0.274868	-2.261473
C	-4.322949	0.919923	-3.418458
C	-5.610252	0.210223	-3.540349
H	-3.731099	0.808478	-4.334218
H	-4.468379	1.981175	-3.203270
C	-5.667091	-1.101057	-4.053340
C	-6.808508	0.785789	-3.083328
C	-8.012686	0.107782	-3.152528
C	-8.056638	-1.191231	-3.686575
C	-6.861439	-1.789798	-4.129448
H	-8.930497	0.570864	-2.801961
H	-6.781161	1.788701	-2.664473
H	-6.891711	-2.799288	-4.529312
H	-4.738065	-1.570440	-4.359277
C	-9.289971	-1.883512	-3.787268
O	-3.016401	1.089719	-1.397221
O	-3.138865	-0.934910	-2.425045
N	-10.302879	-2.446498	-3.875748

33

TS(MeOBn--Bn--F−)

C	-2.514353	3.579010	-1.544101
C	-3.129682	2.413682	-1.974003
C	-1.606949	3.569893	-0.470530
C	-1.362456	2.344561	0.169557
C	-1.982366	1.177378	-0.260675
C	-2.868539	1.197759	-1.335063
H	-2.729482	4.524419	-2.036874

H	-3.827127	2.448857	-2.808499
H	-1.763123	0.239827	0.245463
H	-3.351719	0.283629	-1.670328
H	-0.671528	2.318483	1.007139
C	-0.978398	4.796655	-0.008830
C	0.787027	4.451743	-1.436251
H	-0.222181	4.755240	0.760386
H	-1.111399	5.709260	-0.568276
C	1.559566	3.373110	-0.918319
H	1.187561	5.455417	-1.299809
H	0.261841	4.291103	-2.375007
C	2.517956	3.552716	0.109412
C	1.334790	2.025329	-1.312961
C	2.022266	0.966196	-0.747746
C	2.966111	1.186045	0.256968
C	3.208317	2.491113	0.687706
H	2.723708	4.562464	0.459962
H	0.590826	1.825646	-2.080433
H	1.828881	-0.054353	-1.068903
H	3.931529	2.701317	1.469728
O	3.601915	0.053516	0.756948
C	4.542472	0.264456	1.785806
H	5.379992	0.899220	1.453940
H	4.928864	-0.722096	2.058000
H	4.083483	0.728403	2.673435
F	-2.263737	5.301747	1.185284

32
TS(MeBn--Bn--F−)

C	-0.748596	0.354359	0.855728
C	-0.672594	-1.029643	0.958708
C	-1.384284	0.962193	-0.237817
C	-1.956130	0.126675	-1.212021
C	-1.877282	-1.253646	-1.109568
C	-1.232359	-1.846622	-0.020359
H	-0.307376	0.983333	1.623259
H	-0.162379	-1.473425	1.810717
H	-2.328534	-1.877661	-1.878308
H	-1.170981	-2.928792	0.061576
H	-2.470158	0.582883	-2.054952
C	-1.482004	2.409336	-0.339551
C	0.484068	2.514128	-1.491851
H	-0.978991	3.035446	0.381213
H	-1.908091	2.857552	-1.223121
C	1.530133	2.249493	-0.565493
H	0.341551	3.550938	-1.793843
H	0.300847	1.778627	-2.272033
C	2.053360	3.256529	0.289832
C	2.039598	0.940901	-0.351914
C	2.990991	0.675707	0.620207
C	3.498608	1.680038	1.452411
C	3.001762	2.976009	1.259663
H	1.696635	4.278213	0.169192
H	1.660752	0.128952	-0.968432
H	3.347732	-0.347135	0.743637
H	3.374087	3.785780	1.887849
C	4.496752	1.374472	2.537830
H	5.142318	0.532600	2.260761
H	4.008613	1.107243	3.486686
H	5.142303	2.237322	2.740741
F	-3.084351	2.592582	0.544855

29
TS(FBn--Bn--F−)

C	-1.837683	2.135824	-1.227366
C	-2.262721	1.266189	-2.225125
C	-1.691775	3.508230	-1.480786
C	-2.005495	3.975005	-2.768330
C	-2.425510	3.106282	-3.763792
C	-2.558019	1.739722	-3.500940
H	-1.610591	1.757957	-0.234750
H	-2.356953	0.205479	-2.003440
H	-2.662224	3.493883	-4.752534
H	-2.889572	1.058247	-4.280095
H	-1.916902	5.039303	-2.973858
C	-1.267588	4.423212	-0.433022
C	0.919045	4.146848	-1.012688
H	-0.960202	4.036849	0.526705
H	-1.093897	5.462110	-0.664999
C	1.309428	2.839651	-0.605523
H	1.273004	4.977604	-0.403716
H	0.875230	4.348479	-2.080499
C	1.700380	2.554939	0.731180
C	1.212914	1.720048	-1.475828
C	1.505091	0.429432	-1.055296
C	1.891501	0.219125	0.258018
C	1.988959	1.266901	1.160958
H	1.776005	3.378167	1.438670
H	0.890996	1.883854	-2.500950
H	1.424199	-0.413542	-1.736045
F	2.187200	-1.056819	0.677084
H	2.289867	1.068495	2.186041
F	-2.939632	4.825155	0.208251

29
TS(Bn--Bn--F−)

C	-1.828113	2.113808	-1.262216
C	-2.273577	1.267722	-2.270836
C	-1.686344	3.491354	-1.488264
C	-2.025569	3.987695	-2.757739
C	-2.467913	3.142377	-3.763904
C	-2.595703	1.770681	-3.529115
H	-1.577497	1.713354	-0.284387
H	-2.363223	0.202010	-2.072416
H	-2.724748	3.552356	-4.738519
H	-2.943573	1.106751	-4.316400
H	-1.939647	5.056451	-2.939914
C	-1.236802	4.382656	-0.430819
C	0.933561	4.118152	-1.034277
H	-0.926299	3.978213	0.520197
H	-1.071799	5.427160	-0.642188
C	1.324550	2.820772	-0.605059
H	1.281384	4.962254	-0.440406
H	0.876132	4.300853	-2.104919
C	1.708072	2.559157	0.739576
C	1.238949	1.686751	-1.458048
C	1.525049	0.409118	-1.003930
C	1.906280	0.180612	0.320892
C	1.988978	1.278122	1.184643
H	1.777712	3.397577	1.430887
H	0.927423	1.836312	-2.489241
H	1.442533	-0.428858	-1.694178
H	2.127272	-0.823161	0.672699
H	2.279332	1.126789	2.223105
F	-2.911364	4.779590	0.242100

39
TS(PhBn--Bn--F−)

C	-2.221271	3.037971	-1.505683
C	-2.566360	1.732663	-1.823692
C	-1.400481	3.321869	-0.403721
C	-0.947885	2.248207	0.375034
C	-1.296917	0.940640	0.058377
C	-2.105676	0.670627	-1.042432

H	-2.593493	3.863734	-2.107522
H	-3.206472	1.537720	-2.681397
H	-0.926555	0.124797	0.674766
H	-2.376728	-0.352471	-1.289806
H	-0.314678	2.451370	1.233799
C	-1.038645	4.692369	-0.072803
C	0.771913	4.691486	-1.348655
H	-0.388559	4.894751	0.763789
H	-1.381862	5.507120	-0.689937
C	1.726837	3.833138	-0.747356
H	0.945919	5.762786	-1.260115
H	0.330018	4.372926	-2.289929
C	2.582637	4.273833	0.299478
C	1.807777	2.450422	-1.066313
C	2.672417	1.599384	-0.409845
C	3.529854	2.049977	0.613413
C	3.450507	3.418306	0.945232
H	2.536991	5.318415	0.602473
H	1.165319	2.060345	-1.852357
H	2.712417	0.557024	-0.718511
H	4.061062	3.805295	1.758312
C	4.452941	1.145475	1.299050
C	5.676625	1.601876	1.829082
C	4.160794	-0.223833	1.461708
C	5.040119	-1.081652	2.107944
C	6.549495	0.744151	2.484261
C	6.243555	-0.608389	2.629273
H	3.210515	-0.607494	1.101664
H	4.774927	-2.130281	2.222154
H	5.955107	2.643104	1.693454
H	7.488140	1.132453	2.873241
H	6.928551	-1.279845	3.139310
F	-2.532373	4.983291	1.037020

35

TS(MeOOCBn--Bn--F−)

C	-0.442252	0.544066	1.121927
C	-0.420136	-0.838778	1.258796
C	-1.143006	1.150669	0.071890
C	-1.823911	0.324674	-0.833619
C	-1.799921	-1.056124	-0.698650
C	-1.096123	-1.649401	0.350803
H	0.091217	1.171333	1.830344
H	0.139737	-1.286221	2.076319
H	-2.339053	-1.677201	-1.410589
H	-1.075326	-2.730877	0.456943
H	-2.385497	0.785223	-1.643013
C	-1.162023	2.599546	-0.076846
C	0.584976	2.710207	-1.373608
H	-0.645253	3.225371	0.633420
H	-1.715235	3.054113	-0.882809
C	1.719438	2.352458	-0.607154
H	0.460053	3.766368	-1.606657
H	0.268461	2.024705	-2.156351
C	2.390700	3.297687	0.220939
C	2.180654	1.007326	-0.526063
C	3.228276	0.649468	0.289228
C	3.888677	1.601643	1.092821
C	3.435152	2.937440	1.040913
H	2.059472	4.334207	0.196665
H	1.675421	0.250176	-1.121023
H	3.564531	-0.382564	0.334402
H	3.923477	3.687962	1.654345
C	4.993917	1.168163	1.927829
O	5.540333	2.200064	2.665577
O	5.440563	0.032611	2.013840
C	6.627135	1.811574	3.506063

H	6.312749	1.056902	4.235640
H	6.945974	2.722957	4.018006
H	7.453851	1.396995	2.918204
F	-2.741456	2.804747	0.977902

30

TS(NCBn--Bn--F−)

C	-1.865501	2.141464	-1.161705
C	-2.311688	1.236934	-2.117974
C	-1.663959	3.488981	-1.485912
C	-1.926576	3.899331	-2.800500
C	-2.368411	2.995764	-3.756492
C	-2.564777	1.654837	-3.421697
H	-1.668076	1.809226	-0.146441
H	-2.454843	0.194866	-1.842792
H	-2.570373	3.337792	-4.769091
H	-2.911781	0.946557	-4.169419
H	-1.789368	4.946285	-3.060681
C	-1.191932	4.436503	-0.484657
C	0.920433	4.199112	-0.941185
H	-0.991985	4.107078	0.522758
H	-1.068571	5.477190	-0.737911
C	1.306961	2.890291	-0.565789
H	1.254234	5.013194	-0.299980
H	0.897423	4.430594	-2.003475
C	1.675417	2.573723	0.773626
C	1.233414	1.795115	-1.473230
C	1.525094	0.507909	-1.086072
C	1.903477	0.220678	0.243551
C	1.964132	1.287722	1.167237
H	1.729570	3.380346	1.501697
H	0.925511	1.990320	-2.497358
H	1.459838	-0.305544	-1.803448
C	2.220202	-1.096983	0.639056
H	2.248380	1.079269	2.195155
F	-2.957997	4.891052	0.075487
N	2.483812	-2.183735	0.964457

43

TS(MeOBn--SiH2Ph2−)

C	-3.323996	-3.315055	0.347823
C	-3.983571	-4.141277	1.255687
C	-1.941158	-3.099184	0.436891
C	-1.240327	-3.734395	1.472845
C	-1.895049	-4.560133	2.383299
C	-3.269391	-4.765551	2.275592
H	-3.898402	-2.828560	-0.438753
H	-5.056629	-4.293324	1.170172
H	-1.333875	-5.040740	3.180732
H	-3.782550	-5.407218	2.987342
H	-0.167628	-3.577508	1.575732
Si	-1.048965	-2.006595	-0.818909
C	0.319378	-1.049616	0.025544
C	1.666540	-1.236547	-0.310425
C	0.004731	-0.107080	1.017989
C	1.006311	0.628068	1.651709
C	2.339853	0.426606	1.307724
C	2.670129	-0.506469	0.324435
H	1.936692	-1.949974	-1.086304
H	-1.033502	0.062580	1.299110
H	0.743476	1.363894	2.407703
H	3.121429	1.003369	1.795902
H	3.710091	-0.660926	0.048334
C	0.074477	0.572140	-3.339645
H	-2.084205	-1.110975	-1.396631
C	0.321184	1.614712	-2.431922
H	-0.940405	0.257033	-3.564730

H	0.881088	0.155007	-3.937982
C	1.633877	2.118708	-2.149673
C	-0.721718	2.245228	-1.667749
C	-0.472286	3.237878	-0.735348
C	0.829847	3.685028	-0.481652
C	1.879144	3.116746	-1.210815
H	2.481852	1.691666	-2.683843
H	-1.749892	1.920552	-1.823990
H	-1.295925	3.677259	-0.174849
H	2.905798	3.434446	-1.049683
H	-0.463997	-2.856577	-1.890738
O	0.967001	4.687728	0.472208
C	2.293830	5.012680	0.852246
H	2.834245	4.132302	1.234527
H	2.214174	5.756677	1.649864
H	2.868327	5.447396	0.020230

42

TS(MeBn--SiH2Ph2−)

C	0.789281	-0.443894	-0.523645
C	0.755373	-1.600836	-1.312904
C	0.340956	-2.819520	-0.777591
C	-0.047948	-2.898982	0.558577
C	-0.027339	-1.757677	1.355752
C	0.390267	-0.540894	0.818297
H	1.044669	-1.550360	-2.360662
H	0.316169	-3.706630	-1.405344
H	-0.380622	-3.846971	0.973719
H	-0.344547	-1.811649	2.394060
H	0.400548	0.342490	1.454634
C	3.284298	3.057520	-0.162185
C	2.978403	1.707018	-0.384729
C	4.490627	3.434573	0.424486
C	5.414679	2.463490	0.803722
C	5.127292	1.116112	0.593971
C	3.920646	0.744201	0.006381
H	2.569966	3.828723	-0.445485
H	4.707350	4.486795	0.590908
H	6.354330	2.755347	1.265810
H	5.842480	0.354032	0.893148
H	3.706907	-0.313068	-0.142229
Si	1.359139	1.199911	-1.213215
C	-2.125436	0.705924	-2.604505
H	0.382979	2.287693	-0.946595
H	1.573060	1.068653	-2.679569
C	-2.641481	0.182882	-1.415691
H	-2.008248	0.080191	-3.486068
H	-1.843117	1.752469	-2.680453
C	-2.810787	0.969263	-0.220898
C	-3.024945	-1.195868	-1.263797
C	-3.514452	-1.704422	-0.072501
C	-3.670156	-0.916600	1.080986
C	-3.302554	0.434980	0.958148
H	-2.533520	2.022827	-0.250875
H	-2.911286	-1.862931	-2.118121
H	-3.777725	-2.762484	-0.026285
H	-3.400245	1.089518	1.826142
C	-4.232557	-1.472766	2.362671
H	-4.198253	-2.568464	2.364475
H	-5.282336	-1.184999	2.526880
H	-3.671871	-1.125594	3.240579

39

TS(FBn--SiH2Ph2−)

C	-1.589192	-0.291170	-2.139937
C	-1.481295	-1.271985	-1.166958
C	-1.526076	1.048800	-1.778962
C	-1.357414	1.482174	-0.415437
C	-1.249620	0.405449	0.534276
C	-1.309078	-0.928558	0.170106
H	-1.711788	-0.578311	-3.181880
H	-1.618873	1.804235	-2.555747
H	-1.101875	0.657967	1.582534
H	-1.218768	-1.713271	0.917896
C	-1.221515	2.820580	-0.054165
F	-1.527487	-2.608728	-1.528374
Si	1.726405	1.813184	-1.572309
H	-1.125255	3.104808	0.989111
H	-1.381055	3.610642	-0.781006
C	3.136048	2.930464	-0.972233
C	4.473012	2.509814	-0.930112
C	5.478122	3.340388	-0.439414
C	5.165896	4.617153	0.019841
C	3.842658	5.055141	-0.013878
C	2.842663	4.218401	-0.501193
C	2.419231	0.649537	-2.893212
C	1.979841	-0.683053	-2.933921
C	3.315399	1.072012	-3.887099
C	3.762751	0.201327	-4.876352
C	3.315614	-1.118932	-4.897693
C	2.420242	-1.556988	-3.924425
H	3.675884	2.099770	-3.881263
H	4.460945	0.551326	-5.633554
H	3.662543	-1.801383	-5.669950
H	2.059952	-2.582783	-3.935844
H	1.266828	-1.031863	-2.188794
H	1.806788	4.555325	-0.507039
H	5.948484	5.267413	0.403599
H	3.590525	6.051014	0.343129
H	4.731891	1.517545	-1.295418
H	6.507811	2.990689	-0.415502
H	1.238111	0.944468	-0.480707
H	0.711860	2.717372	-2.150768

39

TS(Bn--SiH2Ph2−)

C	-2.982430	-2.044709	0.832016
C	-3.618783	-2.937246	1.694903
C	-1.705513	-2.311560	0.319730
C	-1.085586	-3.510667	0.706101
C	-1.714010	-4.407226	1.565866
C	-2.985269	-4.121183	2.063302
H	-3.492463	-1.124081	0.552293
H	-4.609853	-2.708298	2.079394
H	-1.214280	-5.330337	1.849705
H	-3.478568	-4.819322	2.734974
H	-0.091329	-3.750546	0.329542
Si	-0.817127	-1.086588	-0.837230
C	0.659433	-0.439148	0.118551
C	1.970567	-0.842733	-0.165593
C	0.460572	0.464411	1.174340
C	1.536083	0.956847	1.912137
C	2.831998	0.541242	1.618048
C	3.046955	-0.361174	0.576609
H	2.158581	-1.529767	-0.988803
H	-0.546064	0.797283	1.422905
H	1.360641	1.668379	2.715246
H	3.672479	0.925056	2.190714
H	4.057022	-0.685724	0.338390
C	0.219199	0.896152	-2.925335
H	-1.845865	-0.049080	-1.094209
C	0.463626	2.016743	-2.127089
H	-0.777516	0.692467	-3.305538
H	1.045051	0.336607	-3.358796

C	1.789318	2.415093	-1.731107
C	-0.590319	2.834996	-1.587919
C	-0.337983	3.904402	-0.743720
C	0.966654	4.257677	-0.368114
C	2.019965	3.484389	-0.885768
H	2.635436	1.841116	-2.107783
H	-1.619253	2.599296	-1.857108
H	-1.180077	4.484717	-0.366833
H	1.155756	5.098689	0.292394
H	3.047532	3.730359	-0.618424
H	-0.404580	-1.886534	-2.017793

49
TS(PhBn--SiH2Ph2−)

C	1.706820	-0.468244	-0.807755
C	1.933132	-1.633879	-1.551747
C	1.612226	-2.888971	-1.038702
C	1.046535	-3.003689	0.230762
C	0.812698	-1.856089	0.984376
C	1.151133	-0.604057	0.473609
H	2.351849	-1.563172	-2.554313
H	1.798022	-3.780326	-1.633354
H	0.785757	-3.981673	0.627229
H	0.364348	-1.934480	1.971936
H	0.965634	0.280341	1.081613
C	3.623739	2.651105	0.602623
C	3.685945	1.717657	-0.441153
C	4.746465	2.965760	1.369151
C	5.966295	2.348786	1.102548
C	6.054763	1.417675	0.067647
C	4.927167	1.110591	-0.690036
H	2.678362	3.146629	0.822341
H	4.670716	3.694339	2.173197
H	6.844465	2.592272	1.695488
H	7.004756	0.933694	-0.147534
H	5.016591	0.379700	-1.494344
Si	2.126265	1.242237	-1.463350
C	-0.067506	0.956544	-3.006939
H	1.203286	2.323181	-1.021555
H	2.674290	1.237738	-2.847275
C	-1.076561	0.596564	-2.101160
H	0.253232	0.233135	-3.754708
H	0.066213	2.004215	-3.266647
C	-1.673729	1.533716	-1.196670
C	-1.521012	-0.756252	-1.939141
C	-2.431950	-1.124886	-0.974371
C	-2.992680	-0.192749	-0.068448
C	-2.575810	1.148263	-0.224986
H	-1.411777	2.586337	-1.288541
H	-1.100846	-1.520699	-2.590832
H	-2.699492	-2.176595	-0.893155
H	-3.003840	1.918342	0.414006
C	-3.954688	-0.592950	0.958521
C	-4.118903	0.148078	2.149982
C	-4.764786	-1.740452	0.808517
C	-5.036889	-0.231144	3.121270
C	-5.823329	-1.371797	2.954466
C	-5.673098	-2.122345	1.787668
H	-4.697520	-2.328086	-0.102750
H	-6.537203	-1.669783	3.717264
H	-6.282627	-3.009536	1.631026
H	-3.497250	1.021412	2.326865
H	-5.129208	0.363545	4.027359

42
TS(F3CBn--SiH2Ph2−)

C	-3.000878	-2.121636	0.567499
C	-3.779454	-2.905119	1.420596
C	-1.739679	-2.545221	0.126943
C	-1.288403	-3.796179	0.577238
C	-2.056743	-4.587895	1.427370
C	-3.308147	-4.141991	1.853252
H	-3.387968	-1.158048	0.236507
H	-4.755203	-2.551421	1.746082
H	-1.682753	-5.553827	1.759308
H	-3.911568	-4.757024	2.516366
H	-0.312143	-4.162929	0.258059
Si	-0.643778	-1.438018	-1.009942
C	0.703705	-0.883744	0.181005
C	2.027767	-1.330843	0.079956
C	0.389018	0.005593	1.219465
C	1.364458	0.450611	2.109998
C	2.675097	-0.006495	1.994649
C	3.002997	-0.903648	0.978785
H	2.308615	-2.012681	-0.721027
H	-0.631942	0.368312	1.332245
H	1.099879	1.155356	2.894740
H	3.437731	0.336653	2.689152
H	4.023847	-1.266031	0.882471
C	0.551087	0.089804	-2.798757
H	-1.661830	-0.407757	-1.360396
C	0.610935	1.338322	-2.162948
H	-0.274243	-0.118880	-3.476196
H	1.481047	-0.437158	-3.006584
C	1.804961	1.813550	-1.522677
C	-0.539904	2.179321	-2.010966
C	-0.509801	3.340827	-1.265317
C	0.669673	3.757335	-0.625991
C	1.831228	2.972086	-0.784180
H	2.714474	1.224311	-1.618780
H	-1.467614	1.888358	-2.498682
H	-1.413290	3.938312	-1.175648
H	2.761146	3.286440	-0.313394
H	-0.182121	-2.437312	-2.014378
C	0.738869	4.997358	0.158665
F	1.539117	5.974099	-0.406840
F	1.281799	4.820645	1.411370
F	-0.469987	5.586563	0.350983

45
TS(MeOOCBn--SiH2Ph2−)

C	1.218548	-0.709154	-0.721057
C	1.348211	-1.860065	-1.510627
C	1.175779	-3.131927	-0.968276
C	0.853047	-3.280025	0.380127
C	0.716840	-2.148473	1.180906
C	0.909515	-0.879698	0.636510
H	1.578360	-1.765001	-2.570614
H	1.287531	-4.010063	-1.600059
H	0.708008	-4.271010	0.802901
H	0.460257	-2.253201	2.232466
H	0.809830	-0.008763	1.283124
C	3.178246	2.423170	0.494289
C	3.128382	1.499109	-0.558157
C	4.375334	2.738286	1.140051
C	5.562632	2.130158	0.739718
C	5.542263	1.207924	-0.307221
C	4.340247	0.902859	-0.942191
H	2.259922	2.913573	0.818387
H	4.382649	3.460922	1.953289
H	6.498386	2.373261	1.237278
H	6.465784	0.730189	-0.627279
H	4.347102	0.179859	-1.759241
Si	1.454886	1.025766	-1.427829

C	-0.662292	0.728695	-2.635874
H	0.640247	2.122263	-0.814526
H	1.947022	1.051938	-2.840388
C	-1.659166	0.547409	-1.655158
H	-0.438990	-0.108626	-3.296595
H	-0.582299	1.712332	-3.098191
C	-2.188941	1.645581	-0.905836
C	-2.118594	-0.748376	-1.260171
C	-3.002196	-0.929140	-0.221723
C	-3.503821	0.170339	0.513753
C	-3.070106	1.459020	0.135143
H	-1.885740	2.655009	-1.175652
H	-1.749677	-1.616566	-1.803112
H	-3.321546	-1.934814	0.035552
H	-3.452771	2.316201	0.683813
C	-4.439040	0.035065	1.618195
O	-4.765746	-1.267721	1.877648
C	-5.686803	-1.464878	2.962592
H	-5.273761	-1.086631	3.903543
H	-5.836501	-2.544596	3.029409
H	-6.641784	-0.966998	2.764716
O	-4.909253	0.956798	2.276366

40

TS(NCBn--SiH2Ph2−)

C	-2.563691	0.758528	-0.924766
C	-2.331496	-0.634374	-1.094346
C	-1.807252	1.512906	-0.069891
C	-0.722917	0.958865	0.706147
C	-0.527842	-0.461686	0.534475
C	-1.284373	-1.211114	-0.327473
H	-3.363173	1.225754	-1.495630
H	-2.006901	2.579061	0.019573
H	0.266376	-0.942920	1.101485
H	-1.088767	-2.276255	-0.431355
C	0.104461	1.735435	1.491551
C	-3.101888	-1.404106	-1.978490
Si	1.913739	1.274426	-1.360413
H	0.881421	1.280151	2.098679
H	-0.077198	2.799061	1.613011
C	2.841545	2.907821	-1.151947
C	3.099228	3.423084	0.125829
C	3.762680	4.635331	0.292970
C	4.177189	5.364983	-0.819304
C	3.926886	4.872583	-2.096958
C	3.268823	3.655335	-2.258064
C	2.585764	0.408479	-2.905900
C	1.705331	-0.323879	-3.715045
C	3.943270	0.414912	-3.256173
C	4.405356	-0.277302	-4.371885
C	3.513518	-0.999117	-5.162374
C	2.161937	-1.022597	-4.830159
H	4.650402	0.978908	-2.649843
H	5.462498	-0.254902	-4.626248
H	3.871557	-1.540874	-6.034443
H	1.459284	-1.584637	-5.440036
H	0.646195	-0.351669	-3.463489
H	3.089528	3.276489	-3.262638
H	4.691395	6.314319	-0.690374
H	4.244565	5.437269	-2.970259
H	2.749572	2.870046	0.995042
H	3.952768	5.014915	1.293985
H	2.199191	0.417923	-0.187100
H	0.471247	1.469602	-1.618339
N	-3.743342	-2.045377	-2.716414

43

TS(Ph2H(MeOBn)Si--H−)

C	-2.920846	-2.782327	-0.270706
C	-3.647663	-3.953426	-0.074594
C	-1.528983	-2.758656	-0.104622
C	-0.892704	-3.947098	0.281226
C	-1.619066	-5.115965	0.507773
C	-2.999685	-5.121390	0.328037
H	-3.444207	-1.875776	-0.563265
H	-4.725392	-3.952050	-0.218477
H	-1.105705	-6.022144	0.820127
H	-3.569590	-6.031566	0.497416
H	0.188647	-3.964208	0.412690
Si	-0.483292	-1.223162	-0.419101
C	0.640834	-0.762389	1.027720
C	1.803227	-0.020639	0.772905
C	0.401895	-1.175269	2.345991
C	1.295089	-0.866414	3.367956
C	2.428762	-0.097764	3.101400
C	2.681310	0.326190	1.800112
H	2.030844	0.297384	-0.243385
H	-0.488458	-1.755410	2.571293
H	1.097809	-1.209854	4.380671
H	3.114941	0.160694	3.904215
H	3.565193	0.920074	1.580050
C	-1.372713	0.169464	-1.365215
H	-2.380921	-0.203767	1.485913
C	-0.589698	1.449360	-1.301447
H	-2.370850	0.301476	-0.941454
H	-1.472047	-0.167131	-2.405813
C	0.359151	1.771712	-2.272205
C	-0.733215	2.329597	-0.217106
C	0.033049	3.480086	-0.117559
C	0.984018	3.783092	-1.098899
C	1.145253	2.923040	-2.186124
H	0.497890	1.107532	-3.123279
H	-1.459775	2.098183	0.557230
H	-0.088874	4.156167	0.724796
H	1.867296	3.130766	-2.968224
H	0.537605	-1.690426	-1.426176
O	1.692082	4.936568	-0.908769
C	2.686005	5.261705	-1.881509
H	3.460109	4.484887	-1.938731
H	3.139563	6.196744	-1.545705
H	2.243113	5.412105	-2.875156

39

TS(Ph2HBnSi--H−)

C	-1.571724	-2.321190	1.368671
C	-2.391995	-3.368372	1.779875
C	-1.158137	-2.212106	0.033489
C	-1.610224	-3.176745	-0.877814
C	-2.458674	-4.208994	-0.477438
C	-2.849113	-4.307361	0.855212
H	-1.235093	-1.587695	2.095930
H	-2.690327	-3.444139	2.822748
H	-2.807141	-4.938597	-1.204340
H	-3.501927	-5.115769	1.175178
H	-1.294830	-3.128282	-1.919519
Si	-0.009967	-0.848017	-0.584808
C	1.109782	-0.149046	0.768303
C	2.062590	-1.032668	1.298864
C	1.083574	1.160554	1.267983
C	1.980956	1.571758	2.250354
C	2.905499	0.672950	2.780384
C	2.942228	-0.635037	2.304989
H	2.120581	-2.054265	0.924330
H	0.351763	1.865589	0.886314

H	1.948955	2.596246	2.613207
H	3.595209	0.992311	3.557827
H	3.659312	-1.344511	2.710667
C	-0.839907	0.325447	-1.842809
H	-1.987644	0.757387	0.989199
C	-0.267812	1.711440	-1.846477
H	-1.912436	0.353212	-1.636230
H	-0.693376	-0.142822	-2.826715
C	1.050202	1.938622	-2.264891
C	-1.012519	2.802373	-1.381649
C	-0.454252	4.078047	-1.334607
C	0.861469	4.288426	-1.741138
C	1.610065	3.211489	-2.214643
H	1.646265	1.103480	-2.629247
H	-2.040179	2.646290	-1.068330
H	-1.051296	4.911636	-0.972587
H	1.297159	5.283131	-1.698289
H	2.635675	3.362643	-2.542378
H	0.977291	-1.617417	-1.419619

Geometry scan **TS(O2NBn--SiH2Ph2−) r^{2}SCAN-3c**:

41
TS(O2NBn--SiHPh2−) r^{2}SCAN-3c 2.000Å

C	-2.500095	4.415226	0.252293
C	-3.148713	3.540882	1.125412
C	-1.408973	4.015468	-0.527043
C	-1.001574	2.680721	-0.398009
C	-1.635847	1.792195	0.466732
C	-2.717141	2.221659	1.236143
H	-2.855232	5.443848	0.167600
H	-3.994856	3.885542	1.717861
H	-1.292327	0.761776	0.543753
H	-3.218748	1.533176	1.912714
H	-0.158308	2.327536	-0.994854
Si	-0.449228	5.288527	-1.732229
C	1.025098	5.499541	-0.530869
C	2.297888	5.000922	-0.838624
C	0.883459	6.192258	0.678332
C	1.965355	6.402746	1.529193
C	3.220998	5.895893	1.203350
C	3.382010	5.183350	0.017802
H	2.448691	4.461966	-1.773291
H	-0.093718	6.581013	0.958707
H	1.829337	6.965740	2.449692
H	4.066719	6.054727	1.868028
H	4.356502	4.775907	-0.243650
C	0.338441	6.523452	-3.094043
H	-1.606157	6.270121	-1.588221
C	0.454371	7.892159	-2.628716
H	-0.372530	6.424945	-3.922656
H	1.301951	6.087841	-3.379102
C	1.632423	8.375171	-2.009927
C	-0.660906	8.764581	-2.650682
C	-0.599526	10.039526	-2.127935
C	0.592293	10.488194	-1.549395
C	1.710422	9.646994	-1.484996
H	2.493413	7.715463	-1.941269
H	-1.585911	8.413909	-3.099856
H	-1.454644	10.704764	-2.159359
H	2.620074	10.009990	-1.021333
H	-0.236513	4.177809	-2.751539
N	0.671056	11.826024	-1.024154
O	1.738486	12.200554	-0.517813
O	-0.330901	12.552570	-1.099358

41
TS(O2NBn--SiHPh2−) r^{2}SCAN-3c 2.125Å

C	-2.517553	4.414135	0.252814
C	-3.171291	3.553713	1.135489
C	-1.424684	3.999161	-0.516664
C	-1.020033	2.665525	-0.366792
C	-1.661253	1.791311	0.507080
C	-2.744035	2.235009	1.265749
H	-2.869826	5.442035	0.153852
H	-4.017677	3.909438	1.720551
H	-1.321021	0.761317	0.599779
H	-3.250017	1.557704	1.950143
H	-0.174430	2.301359	-0.952973
Si	-0.461920	5.244649	-1.721277
C	1.020775	5.491554	-0.549350
C	2.282992	4.960171	-0.845161
C	0.899739	6.248357	0.622795
C	1.993329	6.486077	1.451402
C	3.238135	5.946210	1.139350
C	3.377908	5.172901	-0.010630
H	2.416825	4.374796	-1.753937
H	-0.067284	6.670512	0.889631
H	1.874755	7.099495	2.341410
H	4.093141	6.128365	1.785778
H	4.344734	4.741805	-0.262016
C	0.406322	6.548912	-3.156784
H	-1.599815	6.241562	-1.660307
C	0.494974	7.892980	-2.656806
H	-0.313025	6.426819	-3.971941
H	1.368854	6.092133	-3.400673
C	1.655407	8.376332	-1.997082
C	-0.631004	8.758100	-2.677529
C	-0.596646	10.014743	-2.114010
C	0.576491	10.462445	-1.493350
C	1.704124	9.630414	-1.432631
H	2.524320	7.726556	-1.930328
H	-1.541724	8.412061	-3.158985
H	-1.459395	10.670283	-2.144121
H	2.598796	9.989935	-0.937873
H	-0.239765	4.169910	-2.762998
N	0.625582	11.778036	-0.924753
O	1.677847	12.151949	-0.382118
O	-0.385515	12.495915	-0.996046

41
TS(O2NBn--SiHPh2−) r^{2}SCAN-3c 2.250Å

C	-2.543593	4.400978	0.251009
C	-3.209630	3.547293	1.130594
C	-1.439846	3.978470	-0.498959
C	-1.035513	2.646585	-0.331956
C	-1.689853	1.780055	0.539488
C	-2.783816	2.230085	1.277711
H	-2.894306	5.427682	0.139653
H	-4.064062	3.907817	1.700372
H	-1.350555	0.751341	0.646149
H	-3.299588	1.558470	1.960238
H	-0.180952	2.277701	-0.901276
Si	-0.470016	5.205450	-1.691678
C	1.026286	5.486263	-0.558751
C	2.274313	4.914650	-0.839061
C	0.930453	6.307002	0.571922
C	2.035974	6.562319	1.379137
C	3.266200	5.981810	1.084658
C	3.380776	5.149843	-0.026916
H	2.387364	4.281718	-1.718153
H	-0.023062	6.769000	0.820343
H	1.938218	7.224525	2.235916
H	4.130761	6.179631	1.713406

H	4.337175	4.689523	-0.265555
C	0.463230	6.582036	-3.207120
H	-1.576682	6.226643	-1.684957
C	0.525841	7.902323	-2.677740
H	-0.273613	6.429047	-3.998210
H	1.421484	6.104674	-3.417393
C	1.675189	8.390525	-1.993843
C	-0.612416	8.758175	-2.690527
C	-0.599634	9.999739	-2.097826
C	0.561274	10.451471	-1.452868
C	1.699849	9.630336	-1.401646
H	2.553124	7.751956	-1.933641
H	-1.513664	8.412845	-3.190549
H	-1.470657	10.644659	-2.122262
H	2.584559	9.990442	-0.889419
H	-0.232928	4.170952	-2.759912
N	0.586119	11.748407	-0.854202
O	1.628779	12.126130	-0.290739
O	-0.435444	12.456111	-0.916749

41

TS(O2NBn--SiHPh2−) r²SCAN-3c 2.375Å

C	-2.571914	4.370166	0.251740
C	-3.250013	3.514546	1.119704
C	-1.453457	3.949468	-0.477400
C	-1.044969	2.619909	-0.300888
C	-1.711739	1.752712	0.560050
C	-2.821075	2.199665	1.276509
H	-2.924284	5.395074	0.133639
H	-4.115948	3.872449	1.673090
H	-1.369894	0.725969	0.675029
H	-3.346541	1.527009	1.950403
H	-0.178879	2.253220	-0.853290
Si	-0.480854	5.168389	-1.653131
C	1.030549	5.486412	-0.565133
C	2.252089	4.841702	-0.801327
C	0.973776	6.402518	0.492951
C	2.091585	6.671230	1.278424
C	3.294280	6.016741	1.030943
C	3.370750	5.096870	-0.013100
H	2.334106	4.135360	-1.626170
H	0.045646	6.932897	0.695957
H	2.025488	7.405460	2.077408
H	4.168621	6.226737	1.641863
H	4.306833	4.581462	-0.216547
C	0.508376	6.623920	-3.247959
H	-1.555941	6.211926	-1.689563
C	0.549233	7.921291	-2.692252
H	-0.250369	6.437092	-4.007667
H	1.459643	6.124531	-3.427283
C	1.693719	8.415059	-1.996078
C	-0.600703	8.768419	-2.690693
C	-0.603171	9.996062	-2.073601
C	0.551365	10.452193	-1.415491
C	1.700738	9.641902	-1.381038
H	2.579971	7.786667	-1.946827
H	-1.496956	8.423274	-3.200272
H	-1.482013	10.630784	-2.086941
H	2.580005	10.003122	-0.859986
H	-0.230596	4.180712	-2.753558
N	0.557976	11.730923	-0.790279
O	1.595684	12.112170	-0.214910
O	-0.473976	12.428669	-0.837641

41

TS(O2NBn--SiHPh2−) r²SCAN-3c 2.500Å

C	-2.553086	4.343158	0.306615
C	-3.233766	3.480714	1.164799
C	-1.464825	3.911893	-0.461888
C	-1.089296	2.567285	-0.335922
C	-1.759953	1.694067	0.516130
C	-2.838342	2.150310	1.271966
H	-2.879234	5.380094	0.228460
H	-4.075822	3.845595	1.749162
H	-1.445192	0.655318	0.592039
H	-3.366898	1.472626	1.938206
H	-0.249212	2.192682	-0.921457
Si	-0.492612	5.132900	-1.613952
C	1.022955	5.497787	-0.558942
C	2.222592	4.794301	-0.734437
C	0.989976	6.494928	0.424464
C	2.109388	6.779081	1.202037
C	3.289461	6.065998	1.017013
C	3.343355	5.070710	0.042531
H	2.285449	4.023925	-1.501532
H	0.082989	7.077648	0.571134
H	2.063116	7.574052	1.941895
H	4.165413	6.290208	1.620435
H	4.263011	4.511373	-0.113775
C	0.549030	6.662385	-3.294922
H	-1.533521	6.200073	-1.687310
C	0.574108	7.940486	-2.719148
H	-0.234725	6.440295	-4.016399
H	1.491815	6.141855	-3.448808
C	1.720582	8.442766	-2.024955
C	-0.587072	8.778314	-2.695689
C	-0.598699	9.993807	-2.058714
C	0.555766	10.456286	-1.400182
C	1.716468	9.658432	-1.391660
H	2.614870	7.824465	-1.992004
H	-1.483518	8.431288	-3.204196
H	-1.485545	10.617587	-2.054046
H	2.595327	10.022403	-0.871555
H	-0.222987	4.190314	-2.742793
N	0.550165	11.718189	-0.751991
O	1.588333	12.104492	-0.175961
O	-0.492724	12.404598	-0.775960

41

TS(O2NBn--SiHPh2−) r²SCAN-3c 2.625Å

C	-1.584271	3.912104	0.926878
C	-2.265151	2.969673	1.692273
C	-1.367379	3.727331	-0.446249
C	-1.858228	2.546417	-1.016899
C	-2.541973	1.594526	-0.261496
C	-2.748281	1.804936	1.098658
H	-1.198804	4.809214	1.410050
H	-2.415678	3.139321	2.756080
H	-2.910026	0.685739	-0.732974
H	-3.277611	1.064923	1.694165
H	-1.698055	2.364200	-2.079370
Si	-0.467026	5.059413	-1.518335
C	0.939388	5.583924	-0.390815
C	2.014514	4.717451	-0.141360
C	0.932728	6.824767	0.259774
C	1.958405	7.185902	1.129922
C	3.020303	6.316720	1.358953
C	3.048510	5.080125	0.715306
H	2.039856	3.739874	-0.620662
H	0.128070	7.530095	0.066765
H	1.935328	8.161199	1.608986
H	3.825800	6.602663	2.030676
H	3.875380	4.395133	0.888452
C	0.624394	6.678958	-3.272330

H	-1.532977	6.083271	-1.682792
C	0.637187	7.981479	-2.776482
H	-0.182935	6.376430	-3.933578
H	1.561660	6.137805	-3.374177
C	1.795474	8.551262	-2.149979
C	-0.536695	8.808590	-2.781948
C	-0.553742	10.057184	-2.217646
C	0.607830	10.578459	-1.611013
C	1.784475	9.802146	-1.592089
H	2.703356	7.953894	-2.108309
H	-1.439363	8.417510	-3.245971
H	-1.450847	10.666205	-2.228107
H	2.670703	10.213761	-1.122046
H	-0.135074	4.201068	-2.692272
N	0.593483	11.869057	-1.032769
O	1.636661	12.303626	-0.496846
O	-0.462252	12.538330	-1.071734

41

TS(O2NBn--SiHPh2−) r²SCAN-3c 2.750Å

C	-1.522148	3.846055	0.954716
C	-2.212858	2.909074	1.718420
C	-1.372746	3.696288	-0.431224
C	-1.939821	2.557895	-1.018420
C	-2.633802	1.613422	-0.264224
C	-2.772887	1.787877	1.109743
H	-1.076561	4.709143	1.447289
H	-2.311014	3.049748	2.792378
H	-3.062279	0.738069	-0.747369
H	-3.310126	1.052647	1.703965
H	-1.832060	2.403278	-2.091673
Si	-0.466080	5.016250	-1.496516
C	0.935442	5.583167	-0.389229
C	2.006031	4.722204	-0.101531
C	0.924307	6.844660	0.220066
C	1.942445	7.231501	1.087844
C	2.998872	6.367209	1.356819
C	3.031292	5.110019	0.754120
H	2.034721	3.730208	-0.550021
H	0.124043	7.545555	-0.004344
H	1.917729	8.222892	1.532231
H	3.798221	6.673238	2.026989
H	3.854964	4.429627	0.957634
C	0.678130	6.709347	-3.336817
H	-1.506510	6.052020	-1.719483
C	0.672242	7.995674	-2.816311
H	-0.146689	6.376797	-3.959649
H	1.611389	6.159385	-3.418839
C	1.826770	8.573511	-2.184145
C	-0.513900	8.809973	-2.802462
C	-0.545946	10.044937	-2.212178
C	0.610368	10.571801	-1.596664
C	1.799055	9.811461	-1.601103
H	2.743961	7.989406	-2.158615
H	-1.412988	8.416082	-3.271366
H	-1.451777	10.641094	-2.206481
H	2.681135	10.226452	-1.126038
H	-0.101444	4.188552	-2.679459
N	0.578466	11.844672	-0.988436
O	1.617629	12.283658	-0.444519
O	-0.488438	12.499836	-1.006413

41

TS(O2NBn--SiHPh2−) r²SCAN-3c 2.875Å

C	-1.486706	3.799666	0.972774
C	-2.184968	2.868301	1.736642
C	-1.378549	3.670946	-0.418863
C	-1.993606	2.561165	-1.013048
C	-2.695216	1.623634	-0.258107
C	-2.793328	1.776231	1.122171
H	-1.003486	4.640399	1.467984
H	-2.251431	2.991342	2.815064
H	-3.161754	0.770591	-0.745553
H	-3.336771	1.045828	1.716597
H	-1.918024	2.423762	-2.091230
Si	-0.464824	4.978920	-1.479544
C	0.931058	5.583134	-0.388734
C	1.994663	4.727714	-0.059799
C	0.917035	6.864537	0.177085
C	1.926925	7.276741	1.042786
C	2.975410	6.417255	1.354525
C	3.010270	5.139959	0.795523
H	2.025439	3.721597	-0.475533
H	0.122721	7.560973	-0.079934
H	1.901686	8.283503	1.450826
H	3.767991	6.742930	2.023464
H	3.828685	4.463906	1.031806
C	0.721137	6.742590	-3.415671
H	-1.481526	6.024837	-1.751335
C	0.701639	8.010696	-2.863654
H	-0.122150	6.386059	-3.998475
H	1.652003	6.188694	-3.492856
C	1.857495	8.593309	-2.233406
C	-0.496433	8.809993	-2.815240
C	-0.538675	10.026924	-2.191161
C	0.617395	10.555730	-1.573849
C	1.818211	9.813757	-1.617193
H	2.784055	8.023414	-2.235674
H	-1.395979	8.415547	-3.283048
H	-1.453226	10.608787	-2.157615
H	2.700135	10.229644	-1.142456
H	-0.070720	4.174045	-2.667005
N	0.573043	11.806133	-0.927175
O	1.612271	12.245712	-0.380668
O	-0.504759	12.445780	-0.911733

41

TS(O2NBn--SiHPh2−) r²SCAN-3c 3.000Å

C	-1.472741	3.769066	0.985355
C	-2.175492	2.841596	1.749908
C	-1.384559	3.649370	-0.408327
C	-2.023520	2.554362	-1.004989
C	-2.729589	1.621657	-0.248794
C	-2.807806	1.764366	1.133921
H	-0.970995	4.598684	1.480505
H	-2.226857	2.956638	2.829943
H	-3.215160	0.779972	-0.737163
H	-3.354863	1.037290	1.729023
H	-1.963501	2.424883	-2.084985
Si	-0.464199	4.947151	-1.465684
C	0.923035	5.585218	-0.385081
C	1.977203	4.736046	-0.011670
C	0.908131	6.886344	0.133170
C	1.909602	7.324805	0.995841
C	2.947756	6.470841	1.353424
C	2.982749	5.173344	0.842776
H	2.008419	3.715787	-0.391422
H	0.121062	7.577296	-0.158572
H	1.885486	8.346580	1.364550
H	3.733477	6.816598	2.020389
H	3.794026	4.502176	1.114713
C	0.749610	6.779717	-3.507342
H	-1.461852	5.998770	-1.780107
C	0.723668	8.027095	-2.917391

H	-0.112650	6.406124	-4.050079
H	1.679440	6.226476	-3.597677
C	1.886522	8.610357	-2.296886
C	-0.484037	8.810354	-2.820292
C	-0.529072	10.005358	-2.156633
C	0.632479	10.531363	-1.544982
C	1.843308	9.809077	-1.641268
H	2.820998	8.054885	-2.337881
H	-1.388263	8.418222	-3.281149
H	-1.450627	10.572614	-2.084842
H	2.729536	10.222797	-1.172573
H	-0.041960	4.157622	-2.653001
N	0.583594	11.754849	-0.853182
O	1.627603	12.189876	-0.309542
O	-0.502823	12.379059	-0.793319

41

TS(O2NBn--SiHPh2−) r²SCAN-3c 3.125Å

C	-1.471887	3.745245	0.996233
C	-2.176813	2.819418	1.760793
C	-1.390078	3.627785	-0.397916
C	-2.037260	2.538216	-0.995874
C	-2.745457	1.607842	-0.239204
C	-2.817258	1.747529	1.144239
H	-0.964060	4.571177	1.491053
H	-2.223612	2.932023	2.841219
H	-3.237701	0.770261	-0.727767
H	-3.366049	1.021943	1.739495
H	-1.982183	2.411267	-2.076310
Si	-0.465442	4.918969	-1.451853
C	0.909985	5.589406	-0.376539
C	1.953095	4.748101	0.043265
C	0.896173	6.909510	0.090473
C	1.889560	7.375378	0.948293
C	2.915938	6.528656	1.353313
C	2.948928	5.211620	0.895045
H	1.983246	3.714090	-0.297502
H	0.117316	7.593428	-0.237459
H	1.867909	8.411366	1.275036
H	3.695213	6.895198	2.016755
H	3.751864	4.546649	1.204291
C	0.763934	6.821182	-3.604955
H	-1.450067	5.970596	-1.804609
C	0.739162	8.046337	-2.974746
H	-0.116444	6.436590	-4.109663
H	1.692684	6.271029	-3.720319
C	1.913159	8.626830	-2.369856
C	-0.474223	8.814085	-2.821506
C	-0.514738	9.984532	-2.115988
C	0.656320	10.502970	-1.514326
C	1.873439	9.800619	-1.671687
H	2.853212	8.086214	-2.456994
H	-1.386051	8.427337	-3.271954
H	-1.440282	10.538025	-2.000554
H	2.767266	10.209584	-1.213272
H	-0.015099	4.140718	-2.636084
N	0.610846	11.695986	-0.773984
O	1.663105	12.120939	-0.236070
O	-0.480516	12.306035	-0.663827

41

TS(O2NBn--SiHPh2−) r²SCAN-3c 3.250Å

C	-1.485618	3.734238	1.005583
C	-2.195176	2.810553	1.768375
C	-1.393118	3.610210	-0.387272
C	-2.034078	2.517522	-0.986312
C	-2.746764	1.589677	-0.230985
C	-2.829220	1.735275	1.151261
H	-0.983093	4.563220	1.500513
H	-2.250701	2.927773	2.847836
H	-3.234221	0.749471	-0.719698
H	-3.381619	1.011487	1.745309
H	-1.970614	2.386204	-2.065645
Si	-0.461095	4.895357	-1.436022
C	0.902726	5.590440	-0.361570
C	1.928637	4.753426	0.106599
C	0.898149	6.928042	0.051942
C	1.885621	7.416437	0.904384
C	2.894399	6.573623	1.358268
C	2.916984	5.238515	0.954939
H	1.951042	3.706477	-0.192938
H	0.132230	7.607356	-0.313639
H	1.872401	8.465114	1.188325
H	3.668796	6.957043	2.017905
H	3.706694	4.576959	1.303066
C	0.767269	6.869111	-3.707127
H	-1.433177	5.946913	-1.822208
C	0.745792	8.070257	-3.035151
H	-0.128518	6.475385	-4.176657
H	1.696475	6.328453	-3.857575
C	1.931488	8.650111	-2.450321
C	-0.472193	8.817684	-2.820317
C	-0.506022	9.961932	-2.073334
C	0.675711	10.474458	-1.486313
C	1.897420	9.796703	-1.709097
H	2.876254	8.128606	-2.588252
H	-1.392334	8.434520	-3.256796
H	-1.434565	10.498442	-1.910814
H	2.799656	10.202721	-1.264667
H	0.015175	4.124573	-2.615060
N	0.636490	11.634304	-0.696944
O	1.698245	12.049309	-0.168063
O	-0.458394	12.226782	-0.532839

41

TS(O2NBn--SiHPh2−) r²SCAN-3c 3.375Å

C	-1.522376	3.744189	1.009035
C	-2.237742	2.819787	1.765301
C	-1.389061	3.600949	-0.378683
C	-1.994575	2.489304	-0.979552
C	-2.712703	1.560710	-0.230278
C	-2.836172	1.725046	1.146700
H	-1.047954	4.588698	1.505357
H	-2.325790	2.951777	2.840836
H	-3.172360	0.705456	-0.719731
H	-3.393056	1.000766	1.735902
H	-1.898897	2.343799	-2.054542
Si	-0.449504	4.885561	-1.416326
C	0.898916	5.600936	-0.334875
C	1.894665	4.764323	0.195119
C	0.915925	6.956466	0.014134
C	1.896914	7.463763	0.863548
C	2.875119	6.621034	1.379568
C	2.874897	5.267463	1.042252
H	1.899017	3.703863	-0.053668
H	0.172835	7.634165	-0.398244
H	1.901221	8.525019	1.096952
H	3.643770	7.018366	2.037742
H	3.640862	4.606057	1.440145
C	0.735209	6.922885	-3.832188
H	-1.411832	5.935599	-1.829657
C	0.730122	8.094182	-3.111390
H	-0.179345	6.524727	-4.260182
H	1.661806	6.396352	-4.038274

C	1.935403	8.669039	-2.560212
C	-0.485537	8.818281	-2.812925
C	-0.498848	9.928204	-2.015668
C	0.701678	10.428701	-1.455913
C	1.920918	9.780332	-1.767488
H	2.879703	8.169829	-2.766854
H	-1.419724	8.442527	-3.225351
H	-1.425254	10.444877	-1.788862
H	2.837573	10.179824	-1.347235
H	0.054751	4.123404	-2.589376
N	0.683538	11.543334	-0.605249
O	1.762560	11.941599	-0.096905
O	-0.409535	12.113492	-0.364275

41

TS(O2NBn--SiHPh2−) r^{2}SCAN-3c 3.500Å

C	-1.577572	3.759714	1.005681
C	-2.292910	2.824111	1.747653
C	-1.375524	3.594610	-0.371467
C	-1.911738	2.450148	-0.976250
C	-2.629286	1.509942	-0.240786
C	-2.821894	1.696045	1.125293
H	-1.157538	4.630531	1.505607
H	-2.435316	2.973313	2.815047
H	-3.034463	0.628900	-0.732585
H	-3.378653	0.963010	1.703656
H	-1.761670	2.288284	-2.042495
Si	-0.436415	4.893320	-1.387468
C	0.888045	5.629841	-0.289360
C	1.843989	4.798023	0.316028
C	0.931751	7.001694	-0.014989
C	1.901968	7.530826	0.834016
C	2.840522	6.692550	1.424911
C	2.811939	5.322184	1.164484
H	1.825689	3.725377	0.126997
H	0.217815	7.674305	-0.483007
H	1.927674	8.603220	1.008992
H	3.600104	7.105942	2.083772
H	3.546483	4.664364	1.622987
C	0.636831	6.984153	-3.981029
H	-1.396137	5.937942	-1.819796
C	0.678983	8.119035	-3.207201
H	-0.303837	6.601155	-4.364119
H	1.546827	6.459930	-4.255505
C	1.917114	8.661950	-2.695326
C	-0.513368	8.838098	-2.813018
C	-0.475817	9.904123	-1.958908
C	0.755544	10.361465	-1.429057
C	1.952374	9.725994	-1.841862
H	2.845857	8.172866	-2.981486
H	-1.470529	8.491408	-3.197268
H	-1.384739	10.414510	-1.658732
H	2.892773	10.096356	-1.448245
H	0.102075	4.149426	-2.556918
N	0.789240	11.414093	-0.505411
O	1.894418	11.758599	-0.011505
O	-0.283475	11.983326	-0.182643

41

TS(O2NBn--SiHPh2−) r^{2}SCAN-3c 3.625Å

C	-1.603283	3.749726	1.011277
C	-2.320335	2.803972	1.738330
C	-1.363631	3.583593	-0.359840
C	-1.864076	2.427965	-0.973916
C	-2.583189	1.477633	-0.253205
C	-2.813403	1.664721	1.106890
H	-1.211544	4.629496	1.518421
H	-2.492283	2.954074	2.801208
H	-2.960227	0.587982	-0.751765
H	-3.371553	0.923820	1.673740
H	-1.684363	2.265615	-2.035398
Si	-0.421972	4.894760	-1.353988
C	0.883490	5.644211	-0.241195
C	1.808054	4.819396	0.419764
C	0.949042	7.025295	-0.022940
C	1.910943	7.570952	0.825303
C	2.818346	6.739088	1.471548
C	2.766991	5.359718	1.268387
H	1.771897	3.740334	0.274851
H	0.257512	7.691101	-0.532317
H	1.954222	8.649049	0.957850
H	3.570999	7.164665	2.130622
H	3.476981	4.707444	1.771364
C	0.559011	7.046890	-4.101101
H	-1.376455	5.938250	-1.801013
C	0.637381	8.147750	-3.283253
H	-0.398241	6.681337	-4.459525
H	1.454167	6.524572	-4.423803
C	1.896951	8.659974	-2.790692
C	-0.534554	8.863129	-2.824655
C	-0.458956	9.897510	-1.935156
C	0.793063	10.321412	-1.425496
C	1.969901	9.689397	-1.898601
H	2.811281	8.174984	-3.126109
H	-1.506933	8.540110	-3.190986
H	-1.352630	10.404974	-1.587905
H	2.925775	10.034653	-1.519728
H	0.143215	4.168833	-2.522305
N	0.865911	11.330305	-0.457792
O	1.988665	11.634281	0.025200
O	-0.189035	11.901712	-0.083382

41

TS(O2NBn--SiHPh2−) r^{2}SCAN-3c 3.750Å

C	-1.638058	3.735686	1.013553
C	-2.358826	2.778003	1.720780
C	-1.348335	3.568220	-0.347836
C	-1.802394	2.399344	-0.972610
C	-2.525004	1.437026	-0.271472
C	-2.805446	1.625463	1.078886
H	-1.282903	4.626093	1.528984
H	-2.570246	2.929242	2.776331
H	-2.865627	0.537123	-0.777723
H	-3.366598	0.875345	1.630408
H	-1.583250	2.236453	-2.026447
Si	-0.402345	4.894618	-1.314178
C	0.884289	5.650612	-0.183520
C	1.763522	4.832389	0.543889
C	0.987061	7.038474	-0.032501
C	1.942533	7.597827	0.814201
C	2.804727	6.772045	1.527032
C	2.714875	5.386293	1.392494
H	1.697296	3.748990	0.451756
H	0.327689	7.697302	-0.590924
H	2.015244	8.679572	0.895979
H	3.551421	7.207576	2.186400
H	3.389092	4.739332	1.948737
C	0.462325	7.116687	-4.208526
H	-1.351221	5.939414	-1.770379
C	0.583020	8.184791	-3.353929
H	-0.512077	6.770432	-4.538935
H	1.338652	6.594863	-4.580022
C	1.865469	8.666290	-2.888612
C	-0.563346	8.897153	-2.829817

C	-0.443974	9.902953	-1.913019
C	0.830280	10.296470	-1.434309
C	1.982158	9.665120	-1.967148
H	2.761555	8.182670	-3.271902
H	-1.551921	8.596160	-3.170730
H	-1.318700	10.408319	-1.517786
H	2.954765	9.985981	-1.610040
H	0.186965	4.189517	-2.483588
N	0.948536	11.267635	-0.434095
O	2.090617	11.534719	0.026839
O	-0.084685	11.842482	-0.007535

41

TS(O2NBn--SiHPh2−) r²SCAN-3c 3.875Å

C	-1.660391	3.716916	1.017238
C	-2.383911	2.749733	1.708438
C	-1.334093	3.550774	-0.336086
C	-1.754243	2.373973	-0.969486
C	-2.479536	1.402278	-0.284161
C	-2.796664	1.589255	1.058194
H	-1.332164	4.613804	1.539186
H	-2.624202	2.899970	2.757923
H	-2.793652	0.496268	-0.796566
H	-3.360083	0.831841	1.597264
H	-1.506338	2.212526	-2.017051
Si	-0.385248	4.889732	-1.279784
C	0.881121	5.656266	-0.133981
C	1.725877	4.850032	0.645477
C	1.008145	7.046927	-0.034551
C	1.954834	7.620962	0.812194
C	2.782744	6.806648	1.577165
C	2.667744	5.418726	1.495127
H	1.639901	3.765374	0.593668
H	0.373525	7.695484	-0.632220
H	2.047666	8.703521	0.855284
H	3.521883	7.253353	2.237578
H	3.314958	4.781297	2.092867
C	0.378512	7.186757	-4.305668
H	-1.331563	5.931506	-1.748662
C	0.538303	8.220794	-3.416905
H	-0.609892	6.862909	-4.616697
H	1.236368	6.665285	-4.718470
C	1.840060	8.669835	-2.971758
C	-0.583025	8.930600	-2.837223
C	-0.424149	9.908247	-1.896597
C	0.868411	10.271150	-1.442849
C	1.996179	9.639045	-2.024960
H	2.718394	8.187299	-3.395386
H	-1.584869	8.652050	-3.157917
H	-1.280344	10.412534	-1.461427
H	2.982657	9.936111	-1.685717
H	0.227451	4.200774	-2.447149
N	1.027167	11.207754	-0.416566
O	2.184677	11.438920	0.026814
O	0.014928	11.787454	0.052107

41

TS(O2NBn--SiHPh2−) r²SCAN-3c 4.000Å

C	-1.696671	3.702039	1.013592
C	-2.421845	2.724718	1.688373
C	-1.318934	3.531168	-0.325835
C	-1.689681	2.339389	-0.962111
C	-2.416478	1.357468	-0.293087
C	-2.784841	1.549159	1.035364
H	-1.408036	4.610986	1.538068
H	-2.702547	2.878831	2.727199
H	-2.691745	0.439948	-0.807270
H	-3.349584	0.783890	1.561777
H	-1.401458	2.174596	-1.998683
Si	-0.366642	4.881849	-1.246582
C	0.880240	5.656098	-0.084561
C	1.673934	4.863145	0.759219
C	1.049549	7.045727	-0.048730
C	1.988825	7.631456	0.798136
C	2.765638	6.829862	1.627897
C	2.607425	5.444013	1.610183
H	1.554037	3.780537	0.757285
H	0.452119	7.683627	-0.694298
H	2.114676	8.711634	0.794297
H	3.497808	7.285674	2.289853
H	3.214262	4.817150	2.259268
C	0.282848	7.265474	-4.392451
H	-1.309517	5.921373	-1.727992
C	0.485313	8.265781	-3.474685
H	-0.719367	6.964642	-4.681544
H	1.119823	6.746183	-4.848563
C	1.806763	8.682784	-3.056197
C	-0.607157	8.971089	-2.836601
C	-0.405121	9.920098	-1.875443
C	0.906147	10.254266	-1.454024
C	2.006061	9.622578	-2.088043
H	2.664633	8.201303	-3.520960
H	-1.622438	8.713481	-3.131525
H	-1.240037	10.421860	-1.398064
H	3.006788	9.896007	-1.771363
H	0.267982	4.207826	-2.411467
N	1.109638	11.158430	-0.407492
O	2.283288	11.356355	0.010368
O	0.121438	11.742192	0.105375

41

TS(O2NBn--SiHPh2−) r²SCAN-3c 4.125Å

C	-1.770261	3.687981	0.998605
C	-2.483654	2.690406	1.655649
C	-1.310990	3.508133	-0.314127
C	-1.588338	2.286812	-0.941542
C	-2.302683	1.284112	-0.289830
C	-2.752641	1.484800	1.011624
H	-1.556439	4.620637	1.517379
H	-2.828640	2.852070	2.673759
H	-2.504769	0.343830	-0.796729
H	-3.308043	0.703697	1.524503
H	-1.236533	2.115426	-1.957073
Si	-0.369960	4.882562	-1.208557
C	0.861456	5.661849	-0.033538
C	1.599430	4.882216	0.870602
C	1.082690	7.044772	-0.059732
C	2.019746	7.636234	0.785344
C	2.741316	6.847528	1.675810
C	2.530033	5.469652	1.720701
H	1.437674	3.806153	0.916877
H	0.526420	7.672687	-0.750529
H	2.186436	8.709822	0.736070
H	3.470647	7.308275	2.337473
H	3.092885	4.853592	2.418011
C	0.165674	7.354632	-4.467021
H	-1.320318	5.917162	-1.686639
C	0.424909	8.318756	-3.525352
H	-0.853180	7.088756	-4.731033
H	0.973114	6.828856	-4.966833
C	1.770272	8.689134	-3.139561
C	-0.626640	9.031568	-2.829166
C	-0.367693	9.951611	-1.853833
C	0.965445	10.244878	-1.471969

C	2.026556	9.599683	-2.156732
H	2.599306	8.198239	-3.645067
H	-1.657779	8.805490	-3.093386
H	-1.172921	10.460772	-1.335113
H	3.044220	9.839391	-1.867924
H	0.282040	4.234676	-2.378925
N	1.227034	11.120552	-0.414789
O	2.418877	11.277905	-0.031175
O	0.272438	11.719377	0.142455

41

TS(O2NBn--SiHPh2−) r²SCAN-3c 4.250Å

C	-1.791031	3.691333	0.998434
C	-2.511114	2.701638	1.659972
C	-1.306556	3.490493	-0.302166
C	-1.565730	2.256707	-0.912664
C	-2.286565	1.261752	-0.256212
C	-2.761576	1.483181	1.032866
H	-1.592185	4.634352	1.504277
H	-2.875933	2.879642	2.668380
H	-2.474305	0.311462	-0.749752
H	-3.322279	0.708298	1.549388
H	-1.194545	2.069361	-1.918382
Si	-0.354929	4.853383	-1.201655
C	0.870893	5.641970	-0.027415
C	1.581630	4.874874	0.908524
C	1.115203	7.020190	-0.087022
C	2.047506	7.619143	0.757982
C	2.741758	6.842770	1.680654
C	2.508099	5.470074	1.757896
H	1.401351	3.803219	0.980683
H	0.579504	7.638758	-0.802265
H	2.230186	8.688891	0.685989
H	3.466483	7.309758	2.342985
H	3.049451	4.864023	2.480555
C	0.155767	7.434673	-4.539114
H	-1.297179	5.886523	-1.699505
C	0.417380	8.367336	-3.566995
H	-0.863669	7.172931	-4.804898
H	0.962056	6.930696	-5.062666
C	1.763874	8.732171	-3.179403
C	-0.632259	9.051039	-2.839452
C	-0.370466	9.943682	-1.839882
C	0.964175	10.235237	-1.461676
C	2.023158	9.615458	-2.172618
H	2.591503	8.261053	-3.705630
H	-1.664318	8.825997	-3.100942
H	-1.174261	10.431896	-1.299280
H	3.041852	9.852550	-1.885229
H	0.303969	4.194268	-2.362332
N	1.229597	11.083913	-0.383930
O	2.424102	11.238362	-0.006366
O	0.276542	11.661628	0.197555

41

TS(O2NBn--SiHPh2−) r²SCAN-3c 4.375Å

C	-1.766892	3.697480	1.013180
C	-2.505798	2.727100	1.682733
C	-1.296985	3.481112	-0.290213
C	-1.589813	2.252206	-0.895416
C	-2.329718	1.276883	-0.230925
C	-2.789972	1.513406	1.060922
H	-1.541435	4.636942	1.514340
H	-2.859039	2.916653	2.693140
H	-2.543776	0.329941	-0.720162
H	-3.365492	0.753696	1.583654
H	-1.230039	2.053057	-1.903018
Si	-0.322941	4.819776	-1.201360
C	0.895191	5.621369	-0.028803
C	1.608568	4.861986	0.911568
C	1.127397	7.001717	-0.089527
C	2.048900	7.610503	0.760509
C	2.746052	6.841550	1.687091
C	2.525831	5.466587	1.764105
H	1.437504	3.788867	0.984806
H	0.590905	7.615480	-0.808642
H	2.218349	8.682615	0.691660
H	3.462076	7.316338	2.353319
H	3.069657	4.866403	2.489790
C	0.182547	7.502757	-4.619944
H	-1.249318	5.851380	-1.731286
C	0.427240	8.400165	-3.611052
H	-0.832250	7.238548	-4.900600
H	0.997725	7.028321	-5.157116
C	1.767059	8.766876	-3.202218
C	-0.634653	9.044462	-2.865678
C	-0.389555	9.910831	-1.839223
C	0.939432	10.211065	-1.447573
C	2.009908	9.623788	-2.168921
H	2.603339	8.321760	-3.737380
H	-1.662367	8.812994	-3.138485
H	-1.202206	10.371367	-1.287648
H	3.023949	9.864921	-1.868743
H	0.344148	4.135845	-2.343460
N	1.188591	11.035615	-0.347796
O	2.379851	11.198978	0.037410
O	0.225166	11.583344	0.245645

41

TS(O2NBn--SiHPh2−) r²SCAN-3c 4.500Å

C	-1.731283	3.716596	1.021473
C	-2.500105	2.778770	1.703838
C	-1.279551	3.473207	-0.283422
C	-1.621315	2.251268	-0.877157
C	-2.391345	1.308810	-0.199903
C	-2.832942	1.571771	1.093533
H	-1.467525	4.651081	1.513001
H	-2.838765	2.988786	2.715165
H	-2.643359	0.366575	-0.680076
H	-3.431898	0.837526	1.626233
H	-1.276277	2.031346	-1.885640
Si	-0.266338	4.769609	-1.213105
C	0.937860	5.588588	-0.039104
C	1.655941	4.838748	0.905403
C	1.147986	6.972648	-0.095492
C	2.051141	7.594731	0.764886
C	2.753566	6.834927	1.694901
C	2.556604	5.456129	1.766264
H	1.501397	3.762870	0.975514
H	0.608335	7.579304	-0.818238
H	2.201039	8.670243	0.703517
H	3.455190	7.320105	2.368914
H	3.104778	4.863260	2.494682
C	0.213655	7.574647	-4.698986
H	-1.164095	5.798921	-1.795502
C	0.435317	8.434311	-3.652807
H	-0.794260	7.303611	-4.997306
H	1.040722	7.137851	-5.249775
C	1.765350	8.810369	-3.221178
C	-0.643267	9.028192	-2.889741
C	-0.421179	9.865800	-1.834731
C	0.899518	10.182894	-1.427882
C	1.985502	9.638417	-2.159581
H	2.613282	8.399278	-3.765217

H	-1.664536	8.784101	-3.175484
H	-1.245754	10.290865	-1.272485
H	2.992944	9.889281	-1.845503
H	0.414714	4.041592	-2.319747
N	1.125863	10.980153	-0.303647
O	2.312074	11.160027	0.090731
O	0.148151	11.487479	0.302340

Geometry scan TS(O2NBn--SiH2Ph2−) r^2SCAN-3c(SMD=THF):

41

TS(O2NBn--SiHPh2−) r^2SCAN-3c SMD 2.000Å

C	-2.497497	4.427063	0.244917
C	-3.143903	3.552122	1.121212
C	-1.408964	4.025933	-0.540537
C	-1.002898	2.688439	-0.414149
C	-1.634036	1.800066	0.455552
C	-2.711625	2.231913	1.230491
H	-2.854952	5.455936	0.169433
H	-3.987712	3.896964	1.716213
H	-1.292220	0.769332	0.530170
H	-3.211009	1.543971	1.908768
H	-0.164706	2.327494	-1.014415
Si	-0.434493	5.298475	-1.738173
C	1.022484	5.528149	-0.522429
C	2.309422	5.057509	-0.820914
C	0.847093	6.190258	0.701973
C	1.910736	6.394996	1.579314
C	3.180031	5.913541	1.263334
C	3.374390	5.234873	0.061930
H	2.491400	4.546065	-1.765312
H	-0.139495	6.559350	0.979196
H	1.748363	6.928273	2.513268
H	4.010270	6.064362	1.948864
H	4.359547	4.849288	-0.191183
C	0.319353	6.513847	-3.136238
H	-1.585289	6.290178	-1.573271
C	0.450665	7.882889	-2.677881
H	-0.418391	6.410743	-3.941194
H	1.273795	6.073067	-3.444445
C	1.659445	8.370268	-2.123263
C	-0.671913	8.746670	-2.634358
C	-0.596077	10.009932	-2.083682
C	0.618854	10.454469	-1.550397
C	1.752538	9.630919	-1.573336
H	2.537995	7.730457	-2.135390
H	-1.612984	8.408071	-3.059005
H	-1.462339	10.661683	-2.064803
H	2.688972	9.991322	-1.163098
H	-0.212309	4.169384	-2.739882
N	0.707100	11.772743	-0.983234
O	1.793507	12.145929	-0.516220
O	-0.306008	12.487742	-0.979139

41

TS(O2NBn--SiHPh2−) r^2SCAN-3c SMD 2.125Å

C	-2.515657	4.426893	0.244706
C	-3.164057	3.566346	1.133281
C	-1.422632	4.011691	-0.527604
C	-1.012672	2.677916	-0.374811
C	-1.648610	1.804585	0.506132
C	-2.730519	2.249010	1.267345
H	-2.875234	5.452898	0.149522
H	-4.010358	3.920783	1.718649
H	-1.305950	0.775950	0.600502
H	-3.232239	1.572598	1.955319
H	-0.169619	2.307856	-0.961737
Si	-0.454735	5.253372	-1.731942
C	1.012433	5.523222	-0.547467
C	2.296829	5.046024	-0.844821
C	0.844772	6.223946	0.655985
C	1.917111	6.461945	1.514212
C	3.184718	5.975705	1.200172
C	3.369651	5.257849	0.020046
H	2.470433	4.504784	-1.773865
H	-0.140251	6.599252	0.930496
H	1.762513	7.025688	2.431318
H	4.021899	6.153610	1.870511
H	4.353800	4.868396	-0.230736
C	0.380010	6.537628	-3.204869
H	-1.591430	6.254310	-1.665388
C	0.490873	7.879155	-2.709670
H	-0.372369	6.411166	-3.989190
H	1.331102	6.070972	-3.474886
C	1.686953	8.363750	-2.113885
C	-0.640529	8.739046	-2.661077
C	-0.585730	9.981117	-2.066927
C	0.614814	10.421198	-1.491696
C	1.756310	9.603881	-1.521532
H	2.571863	7.732658	-2.129949
H	-1.570605	8.407803	-3.115417
H	-1.457787	10.625169	-2.043973
H	2.681314	9.958306	-1.080897
H	-0.232889	4.168591	-2.763647
N	0.679685	11.713325	-0.881989
O	1.754399	12.082339	-0.377313
O	-0.340471	12.423948	-0.874221

41

TS(O2NBn--SiHPh2−) r^2SCAN-3c SMD 2.250Å

C	-2.541657	4.409065	0.244508
C	-3.199856	3.553011	1.129620
C	-1.436680	3.988833	-0.508160
C	-1.022598	2.658214	-0.339086
C	-1.669984	1.790634	0.538676
C	-2.764356	2.238229	1.279521
H	-2.902839	5.432669	0.136074
H	-4.055324	3.909478	1.699836
H	-1.325848	0.764015	0.646303
H	-3.274426	1.565678	1.965019
H	-0.169419	2.286726	-0.909387
Si	-0.469927	5.212205	-1.700880
C	1.010912	5.524747	-0.557188
C	2.290427	5.033665	-0.851465
C	0.854242	6.267263	0.622189
C	1.935105	6.531252	1.461798
C	3.198542	6.032658	1.151590
C	3.371578	5.275219	-0.005556
H	2.453081	4.459855	-1.762602
H	-0.127094	6.655872	0.891411
H	1.790241	7.126077	2.360557
H	4.042636	6.232342	1.806878
H	4.352976	4.876949	-0.252845
C	0.421372	6.567531	-3.260103
H	-1.576242	6.234454	-1.696945
C	0.517041	7.882453	-2.734316
H	-0.359140	6.409477	-4.006847
H	1.363519	6.073668	-3.503395
C	1.709410	8.366499	-2.118608
C	-0.622094	8.739397	-2.671067
C	-0.577665	9.965014	-2.048057
C	0.617597	10.404048	-1.453911
C	1.765339	9.590948	-1.498649
H	2.599163	7.742254	-2.144325
H	-1.548083	8.412740	-3.137547

H	-1.454196	10.602717	-2.014652
H	2.685325	9.941262	-1.044064
H	-0.228685	4.176706	-2.764980
N	0.670037	11.675138	-0.817456
O	1.741345	12.043446	-0.296544
O	-0.356639	12.382275	-0.796658

41

TS(O2NBn--SiHPh2−) r²SCAN-3c SMD 2.375Å

C	-2.563002	4.383148	0.252376
C	-3.232262	3.527413	1.128770
C	-1.448995	3.959641	-0.485289
C	-1.034889	2.629811	-0.310459
C	-1.694832	1.763907	0.559173
C	-2.798814	2.212773	1.284386
H	-2.921836	5.406536	0.138782
H	-4.094784	3.884452	1.687509
H	-1.352493	0.737486	0.672256
H	-3.318313	1.540743	1.963166
H	-0.174513	2.257955	-0.868726
Si	-0.483761	5.172109	-1.662643
C	1.010262	5.528456	-0.561898
C	2.283334	5.016776	-0.848045
C	0.865606	6.315984	0.589402
C	1.953862	6.600702	1.412282
C	3.211313	6.082480	1.111114
C	3.371873	5.283624	-0.019831
H	2.434389	4.407630	-1.737859
H	-0.110600	6.723704	0.848904
H	1.819455	7.228451	2.289926
H	4.061321	6.299828	1.752930
H	4.349189	4.871350	-0.259841
C	0.456414	6.602369	-3.309169
H	-1.557299	6.215550	-1.711100
C	0.538455	7.891855	-2.754473
H	-0.352797	6.410678	-4.012976
H	1.388510	6.082257	-3.526949
C	1.730537	8.377850	-2.128328
C	-0.608383	8.745117	-2.674097
C	-0.570607	9.957578	-2.030390
C	0.623019	10.398389	-1.427094
C	1.776795	9.589893	-1.488622
H	2.625001	7.760517	-2.165237
H	-1.532940	8.420398	-3.145292
H	-1.451412	10.588805	-1.985013
H	2.694307	9.938433	-1.027359
H	-0.222050	4.191108	-2.762176
N	0.666791	11.651824	-0.771333
O	1.737581	12.021891	-0.242285
O	-0.366293	12.355211	-0.735805

41

TS(O2NBn--SiHPh2−) r²SCAN-3c SMD 2.500Å

C	-2.594822	4.348863	0.254389
C	-3.271663	3.490945	1.122202
C	-1.465844	3.928592	-0.462283
C	-1.042771	2.603071	-0.276290
C	-1.711434	1.736506	0.585484
C	-2.830668	2.180451	1.289689
H	-2.958800	5.368804	0.130920
H	-4.145802	3.843552	1.665112
H	-1.363417	0.713355	0.708669
H	-3.356419	1.507230	1.962339
H	-0.170537	2.235716	-0.818132
Si	-0.500819	5.134528	-1.622081
C	1.007077	5.530062	-0.563699
C	2.271080	4.991129	-0.839951
C	0.880308	6.367192	0.554158
C	1.978343	6.669728	1.357178
C	3.226852	6.124837	1.066806
C	3.369482	5.280064	-0.032895
H	2.406324	4.343235	-1.704497
H	-0.088034	6.800058	0.801924
H	1.858933	7.334295	2.209421
H	4.084670	6.357932	1.692439
H	4.340351	4.848542	-0.264727
C	0.489086	6.641177	-3.354161
H	-1.538650	6.201318	-1.717572
C	0.558711	7.907329	-2.772220
H	-0.345000	6.416539	-4.015157
H	1.411160	6.096069	-3.544322
C	1.750557	8.394347	-2.136567
C	-0.594816	8.757297	-2.677045
C	-0.562570	9.957882	-2.015488
C	0.629695	10.399628	-1.403524
C	1.788587	9.595073	-1.479504
H	2.648461	7.782124	-2.181865
H	-1.518382	8.434165	-3.151806
H	-1.447112	10.583165	-1.959163
H	2.703608	9.941213	-1.011212
H	-0.217046	4.208780	-2.755884
N	0.665732	11.636472	-0.729492
O	1.735884	12.007257	-0.192156
O	-0.373158	12.336163	-0.680349

41

TS(O2NBn--SiHPh2−) r²SCAN-3c SMD 2.625Å

C	-2.626258	4.308607	0.256617
C	-3.303243	3.447987	1.121091
C	-1.485182	3.893961	-0.444146
C	-1.048608	2.574462	-0.246954
C	-1.718551	1.706570	0.612046
C	-2.850120	2.143321	1.300506
H	-2.998525	5.323961	0.123799
H	-4.186672	3.794412	1.652496
H	-1.361267	0.688054	0.745336
H	-3.376202	1.468571	1.971270
H	-0.166362	2.213724	-0.776229
Si	-0.523336	5.095621	-1.590169
C	0.989550	5.538308	-0.564317
C	2.251179	4.987309	-0.827424
C	0.867960	6.416653	0.521979
C	1.969518	6.744175	1.309930
C	3.215863	6.187463	1.033353
C	3.353496	5.304797	-0.036928
H	2.380789	4.307847	-1.668205
H	-0.098029	6.862535	0.755214
H	1.855131	7.439383	2.137994
H	4.076744	6.441809	1.646330
H	4.323294	4.865748	-0.258589
C	0.511961	6.677985	-3.410858
H	-1.530432	6.179174	-1.737919
C	0.579077	7.920920	-2.798958
H	-0.348474	6.428192	-4.025515
H	1.422269	6.110189	-3.585296
C	1.778995	8.404169	-2.167526
C	-0.577669	8.768524	-2.672853
C	-0.541174	9.954055	-1.988268
C	0.657381	10.390841	-1.379458
C	1.819132	9.590358	-1.487994
H	2.678812	7.796550	-2.235707
H	-1.506327	8.450716	-3.141656
H	-1.427599	10.574161	-1.907441
H	2.738038	9.930590	-1.022713

H	-0.207941	4.218158	-2.748726
N	0.695537	11.607610	-0.679843
O	1.771570	11.973595	-0.144731
O	-0.347191	12.303617	-0.600852

41

TS(O2NBn--SiHPh2−) r²SCAN-3c SMD 2.750Å

C	-2.683916	4.214747	0.229563
C	-3.345995	3.340669	1.091628
C	-1.509545	3.834328	-0.434645
C	-1.022013	2.538344	-0.203866
C	-1.677484	1.658492	0.653718
C	-2.843654	2.059434	1.304967
H	-3.093390	5.211391	0.069922
H	-4.256054	3.658592	1.594880
H	-1.281573	0.658566	0.814714
H	-3.358331	1.374651	1.974331
H	-0.112001	2.206738	-0.703910
Si	-0.572875	5.045954	-1.574383
C	0.909392	5.601016	-0.562537
C	2.198560	5.105413	-0.797876
C	0.732984	6.511103	0.489985
C	1.809806	6.923329	1.271837
C	3.085010	6.421143	1.023186
C	3.276754	5.508627	-0.012843
H	2.369374	4.404431	-1.613168
H	-0.255991	6.917036	0.698349
H	1.653854	7.642423	2.072175
H	3.926958	6.743025	1.630697
H	4.269984	5.114028	-0.213087
C	0.462314	6.697124	-3.514621
H	-1.582011	6.112231	-1.789301
C	0.587844	7.905539	-2.857868
H	-0.454156	6.446489	-4.039918
H	1.344280	6.102637	-3.737094
C	1.844532	8.353220	-2.308459
C	-0.543319	8.763135	-2.603259
C	-0.435536	9.911206	-1.866539
C	0.813672	10.306735	-1.331541
C	1.953876	9.502469	-1.578278
H	2.728646	7.743460	-2.481300
H	-1.511166	8.478197	-3.009986
H	-1.304250	10.535083	-1.684143
H	2.912023	9.812243	-1.174627
H	-0.190900	4.204063	-2.736051
N	0.921936	11.479070	-0.573735
O	2.042635	11.807553	-0.105094
O	-0.103134	12.180749	-0.373137

41

TS(O2NBn--SiHPh2−) r²SCAN-3c SMD 2.875Å

C	-2.701084	4.090196	0.239918
C	-3.325528	3.188610	1.101030
C	-1.519006	3.753187	-0.434525
C	-0.984106	2.474183	-0.215765
C	-1.602735	1.567713	0.641531
C	-2.777095	1.924459	1.303087
H	-3.146297	5.072716	0.089603
H	-4.242456	3.471750	1.612315
H	-1.171348	0.581276	0.793636
H	-3.262969	1.218454	1.971866
H	-0.067521	2.177097	-0.724843
Si	-0.632385	5.001743	-1.562919
C	0.781150	5.699192	-0.544123
C	2.100101	5.256141	-0.707181
C	0.522124	6.657391	0.446984
C	1.548760	7.166732	1.238962
C	2.855036	6.716169	1.062172
C	3.128321	5.757491	0.088705
H	2.334582	4.519586	-1.473869
H	-0.492083	7.023410	0.599374
H	1.329392	7.921477	1.990067
H	3.657900	7.115544	1.676517
H	4.146407	5.404338	-0.056200
C	0.349250	6.736965	-3.634398
H	-1.678936	6.009577	-1.856154
C	0.580007	7.897923	-2.930217
H	-0.633782	6.514316	-4.036505
H	1.178735	6.117316	-3.964205
C	1.903293	8.284794	-2.497124
C	-0.489884	8.773749	-2.512795
C	-0.267978	9.872606	-1.729870
C	1.042509	10.202677	-1.304900
C	2.124426	9.383280	-1.717644
H	2.744911	7.664550	-2.798050
H	-1.503967	8.538509	-2.828868
H	-1.092404	10.507930	-1.423392
H	3.128783	9.643331	-1.400401
H	-0.152641	4.195975	-2.712091
N	1.265785	11.316307	-0.491126
O	2.439394	11.582239	-0.117371
O	0.290476	12.033784	-0.142542

41

TS(O2NBn--SiHPh2−) r²SCAN-3c SMD 3.000Å

C	-2.711090	4.052985	0.259719
C	-3.332578	3.145829	1.116714
C	-1.527466	3.721275	-0.414812
C	-0.987363	2.443662	-0.201240
C	-1.603893	1.532252	0.652232
C	-2.779774	1.882824	1.314060
H	-3.159406	5.034444	0.112821
H	-4.250739	3.423830	1.628439
H	-1.169454	0.546689	0.800740
H	-3.263791	1.172630	1.979692
H	-0.069849	2.151583	-0.711089
Si	-0.647236	4.973418	-1.535056
C	0.752393	5.718723	-0.532121
C	2.076356	5.284157	-0.677756
C	0.479733	6.703885	0.428385
C	1.498058	7.246499	1.208663
C	2.809372	6.803938	1.049580
C	3.096432	5.819760	0.106147
H	2.320772	4.527546	-1.421448
H	-0.538401	7.064821	0.565218
H	1.268792	8.021466	1.935813
H	3.605859	7.230291	1.653999
H	4.118773	5.473881	-0.025360
C	0.344525	6.781695	-3.713713
H	-1.686818	5.975227	-1.869886
C	0.591269	7.915208	-2.975796
H	-0.653156	6.553440	-4.074483
H	1.162369	6.156253	-4.061055
C	1.924870	8.287388	-2.557203
C	-0.467766	8.783852	-2.513261
C	-0.227516	9.856220	-1.700749
C	1.091962	10.168390	-1.287328
C	2.163446	9.359319	-1.747521
H	2.758779	7.674495	-2.893190
H	-1.488425	8.560906	-2.817144
H	-1.043933	10.483837	-1.358835
H	3.174209	9.605737	-1.439840
H	-0.135745	4.192512	-2.687503
N	1.332994	11.249008	-0.437956

O	2.514434	11.497661	-0.073472
O	0.366139	11.957150	-0.045721

41

TS(O2NBn--SiHPh2−) r^{2}SCAN-3c SMD 3.125Å

C	-2.727467	4.027720	0.267591
C	-3.350965	3.120279	1.122587
C	-1.535580	3.699496	-0.394000
C	-0.988651	2.426572	-0.170125
C	-1.607749	1.515424	0.681577
C	-2.791938	1.861756	1.330573
H	-3.180265	5.005649	0.112119
H	-4.275496	3.394738	1.624528
H	-1.168586	0.533355	0.838665
H	-3.277691	1.151533	1.994870
H	-0.064666	2.138171	-0.669976
Si	-0.654414	4.949497	-1.509189
C	0.741215	5.721695	-0.521511
C	2.066856	5.289991	-0.662109
C	0.466135	6.724507	0.419860
C	1.483411	7.285333	1.188292
C	2.796257	6.845323	1.034550
C	3.086126	5.845141	0.109148
H	2.312907	4.519737	-1.391031
H	-0.552980	7.084824	0.550569
H	1.252578	8.073400	1.900694
H	3.592047	7.286738	1.628981
H	4.109842	5.501986	-0.018376
C	0.352565	6.831793	-3.791420
H	-1.683069	5.952438	-1.874224
C	0.602024	7.940087	-3.019450
H	-0.651705	6.596331	-4.129048
H	1.166901	6.208296	-4.150459
C	1.938780	8.304653	-2.601098
C	-0.455168	8.795261	-2.525702
C	-0.210809	9.845099	-1.686196
C	1.111389	10.148300	-1.272875
C	2.180855	9.353908	-1.763734
H	2.771372	7.702521	-2.959434
H	-1.477752	8.578157	-2.827560
H	-1.025859	10.461126	-1.320666
H	3.193219	9.592876	-1.455409
H	-0.123110	4.185801	-2.664483
N	1.356124	11.200632	-0.391557
O	2.539886	11.439492	-0.025740
O	0.390568	11.895047	0.029456

41

TS(O2NBn--SiHPh2−) r^{2}SCAN-3c SMD 3.250Å

C	-2.736454	4.020200	0.276170
C	-3.363506	3.118358	1.134282
C	-1.540442	3.687657	-0.375807
C	-0.992669	2.417315	-0.139633
C	-1.615870	1.512114	0.715261
C	-2.804058	1.862167	1.354768
H	-3.189158	4.996446	0.110874
H	-4.291042	3.395503	1.629050
H	-1.176501	0.531842	0.882371
H	-3.292680	1.156487	2.021770
H	-0.065442	2.126510	-0.631789
Si	-0.654219	4.929404	-1.490889
C	0.738666	5.721620	-0.515215
C	2.066578	5.297203	-0.658057
C	0.461063	6.731935	0.417332
C	1.477716	7.305558	1.176833
C	2.792686	6.872422	1.021252
C	3.085468	5.866142	0.103559
H	2.314245	4.521876	-1.380958
H	-0.559603	7.087870	0.548015
H	1.245164	8.099098	1.882555
H	3.588049	7.324462	1.608215
H	4.110960	5.529093	-0.025698
C	0.369456	6.882384	-3.878452
H	-1.671535	5.933670	-1.884775
C	0.613588	7.962744	-3.067485
H	-0.636752	6.642088	-4.207629
H	1.185067	6.268079	-4.250399
C	1.949223	8.321284	-2.638608
C	-0.447429	8.798293	-2.547139
C	-0.207639	9.823232	-1.676647
C	1.113567	10.120072	-1.254313
C	2.186352	9.345398	-1.769621
H	2.784544	7.733740	-3.014573
H	-1.469227	8.585194	-2.854613
H	-1.025500	10.423538	-1.291696
H	3.197532	9.578601	-1.453090
H	-0.106902	4.173823	-2.644696
N	1.353294	11.141461	-0.337179
O	2.536006	11.371884	0.038858
O	0.384540	11.817918	0.106481

41

TS(O2NBn--SiHPh2−) r^{2}SCAN-3c SMD 3.375Å

C	-2.746851	4.010781	0.284825
C	-3.374960	3.115538	1.148879
C	-1.547144	3.674672	-0.358512
C	-0.996495	2.408315	-0.107931
C	-1.621160	1.509932	0.752905
C	-2.812940	1.863141	1.383889
H	-3.201143	4.984242	0.108360
H	-4.305156	3.395027	1.637209
H	-1.179827	0.532632	0.931600
H	-3.302345	1.162798	2.055905
H	-0.066168	2.115575	-0.592864
Si	-0.657785	4.908621	-1.476054
C	0.733816	5.716146	-0.511087
C	2.063921	5.300446	-0.660178
C	0.454589	6.729854	0.417319
C	1.471408	7.313134	1.168992
C	2.788382	6.888030	1.007862
C	3.083156	5.880079	0.092791
H	2.312562	4.523407	-1.380834
H	-0.567612	7.080155	0.551382
H	1.237724	8.108658	1.872085
H	3.583849	7.348237	1.588290
H	4.110297	5.550076	-0.041266
C	0.392120	6.937292	-3.960571
H	-1.666778	5.913258	-1.891963
C	0.628862	7.989587	-3.111963
H	-0.613728	6.697298	-4.292037
H	1.209994	6.331418	-4.341462
C	1.961516	8.338522	-2.665169
C	-0.436920	8.807617	-2.572713
C	-0.203918	9.809898	-1.674814
C	1.114780	10.099459	-1.239010
C	2.191635	9.339923	-1.768469
H	2.800104	7.761977	-3.050867
H	-1.456851	8.599565	-2.889862
H	-1.025278	10.396978	-1.277117
H	3.200445	9.565581	-1.439161
H	-0.099163	4.159062	-2.629226
N	1.347463	11.094529	-0.292346
O	2.527799	11.316180	0.097145
O	0.374937	11.757042	0.164721

41

TS(O2NBn--SiHPh2−) r^2SCAN-3c SMD 3.500Å

C	-2.751965	4.006635	0.299818
C	-3.383517	3.118090	1.168119
C	-1.552205	3.663100	-0.339543
C	-1.004814	2.396956	-0.080943
C	-1.633319	1.505457	0.784171
C	-2.825027	1.865662	1.411232
H	-3.203320	4.980217	0.117005
H	-4.313604	3.402903	1.653506
H	-1.194857	0.528095	0.969312
H	-3.317214	1.170635	2.086710
H	-0.074539	2.099050	-0.562665
Si	-0.657931	4.887900	-1.460325
C	0.733189	5.708195	-0.505428
C	2.065225	5.300890	-0.661279
C	0.452659	6.723960	0.420429
C	1.469713	7.315417	1.165107
C	2.788470	6.898107	0.997833
C	3.084871	5.889687	0.083929
H	2.314608	4.522824	-1.380495
H	-0.570915	7.068796	0.558373
H	1.235074	8.111726	1.866966
H	3.584084	7.365225	1.572497
H	4.113427	5.566354	-0.055253
C	0.418699	6.993026	-4.040890
H	-1.659340	5.892924	-1.895313
C	0.644577	8.016985	-3.155589
H	-0.584950	6.754814	-4.381013
H	1.241531	6.401638	-4.433744
C	1.972825	8.358090	-2.689333
C	-0.428334	8.814271	-2.598864
C	-0.204939	9.795148	-1.675520
C	1.110276	10.081073	-1.226435
C	2.193153	9.337992	-1.766894
H	2.816172	7.793953	-3.082978
H	-1.445673	8.608889	-2.926007
H	-1.031372	10.367463	-1.266974
H	3.198801	9.558302	-1.424543
H	-0.091733	4.138688	-2.610607
N	1.333342	11.053601	-0.254863
O	2.510750	11.271453	0.146075
O	0.355258	11.700493	0.213105

41

TS(O2NBn--SiHPh2−) r^2SCAN-3c SMD 3.625Å

C	-2.745497	3.995360	0.320782
C	-3.378726	3.101637	1.182364
C	-1.546343	3.653726	-0.320848
C	-1.001100	2.384820	-0.071817
C	-1.631591	1.488369	0.786773
C	-2.822618	1.846370	1.416214
H	-3.195013	4.971041	0.144961
H	-4.308391	3.384601	1.669594
H	-1.195200	0.508758	0.964657
H	-3.316363	1.147302	2.086348
H	-0.071650	2.088573	-0.555969
Si	-0.651702	4.884157	-1.432602
C	0.727176	5.723671	-0.476253
C	2.062261	5.320062	-0.617051
C	0.435042	6.748518	0.435985
C	1.443544	7.351920	1.182449
C	2.765420	6.938683	1.029575
C	3.073487	5.921705	0.129167
H	2.320378	4.534403	-1.324794
H	-0.590888	7.091234	0.561025
H	1.200322	8.154807	1.873828
H	3.554407	7.415902	1.605069
H	4.104529	5.601718	0.001323
C	0.397486	7.052808	-4.141252
H	-1.652386	5.883791	-1.882987
C	0.636156	8.040501	-3.218912
H	-0.610334	6.835316	-4.483515
H	1.212526	6.465620	-4.555881
C	1.969181	8.350471	-2.743977
C	-0.426458	8.831806	-2.633685
C	-0.189356	9.783258	-1.683580
C	1.130482	10.042241	-1.231299
C	2.203011	9.301366	-1.794986
H	2.804771	7.786403	-3.154002
H	-1.447033	8.646675	-2.962952
H	-1.008313	10.351454	-1.254762
H	3.211756	9.498795	-1.447841
H	-0.066753	4.143509	-2.579310
N	1.366658	10.976990	-0.227108
O	2.547790	11.170085	0.176251
O	0.396467	11.616255	0.267701

41

TS(O2NBn--SiHPh2−) r^2SCAN-3c SMD 3.750Å

C	-2.749063	3.968301	0.332057
C	-3.380908	3.073775	1.193693
C	-1.545258	3.630998	-0.303143
C	-0.993650	2.366148	-0.047712
C	-1.622889	1.469087	0.811119
C	-2.818651	1.822467	1.434087
H	-3.203287	4.940809	0.151044
H	-4.314298	3.353031	1.675868
H	-1.181774	0.492568	0.994152
H	-3.311407	1.122819	2.104329
H	-0.060562	2.073580	-0.526963
Si	-0.653505	4.862235	-1.414544
C	0.713765	5.724848	-0.462175
C	2.051162	5.322171	-0.584032
C	0.410901	6.765057	0.428999
C	1.411029	7.383259	1.174372
C	2.735037	6.969995	1.041381
C	3.053935	5.938635	0.161346
H	2.317521	4.524938	-1.275562
H	-0.616595	7.108821	0.536834
H	1.160187	8.199050	1.847671
H	3.517466	7.459161	1.615788
H	4.086852	5.619120	0.048489
C	0.383733	7.123175	-4.220750
H	-1.656034	5.852992	-1.881837
C	0.635245	8.077475	-3.267089
H	-0.627780	6.926524	-4.565221
H	1.190997	6.538813	-4.654077
C	1.972687	8.356679	-2.784959
C	-0.416757	8.864517	-2.656887
C	-0.165870	9.789111	-1.684155
C	1.158341	10.022244	-1.230281
C	2.220177	9.281601	-1.813990
H	2.800400	7.792184	-3.210119
H	-1.440411	8.698927	-2.987155
H	-0.977080	10.354892	-1.237833
H	3.231805	9.456620	-1.463243
H	-0.053842	4.124188	-2.555758
N	1.407696	10.926105	-0.201725
O	2.592434	11.098238	0.200811
O	0.445387	11.559524	0.315857

41

TS(O2NBn--SiHPh2−) r²SCAN-3c SMD 3.875Å

C	-2.917895	3.802819	-0.010110
C	-3.586062	2.936195	0.853651
C	-1.551021	3.644075	-0.276699
C	-0.871638	2.589210	0.352886
C	-1.535559	1.720960	1.214093
C	-2.896006	1.893852	1.466224
H	-3.471031	4.613304	-0.480539
H	-4.646187	3.076520	1.048865
H	-0.992415	0.909182	1.691129
H	-3.415323	1.217407	2.140228
H	0.192537	2.442859	0.172169
Si	-0.633958	4.822784	-1.423845
C	0.752637	5.684196	-0.501185
C	2.094017	5.326514	-0.696263
C	0.460302	6.680508	0.442761
C	1.474265	7.300070	1.167169
C	2.802244	6.930136	0.962463
C	3.110904	5.942477	0.030700
H	2.351560	4.564138	-1.429011
H	-0.571148	6.986368	0.610977
H	1.230849	8.081683	1.882357
H	3.594986	7.419076	1.522702
H	4.146253	5.656763	-0.137002
C	0.283913	7.259797	-4.293358
H	-1.627839	5.804255	-1.928535
C	0.577519	8.148158	-3.289131
H	-0.743254	7.071225	-4.593156
H	1.072293	6.721798	-4.812840
C	1.936683	8.414680	-2.864080
C	-0.447391	8.875322	-2.568321
C	-0.152267	9.738565	-1.552600
C	1.192903	9.963642	-1.160656
C	2.228372	9.279103	-1.850421
H	2.745279	7.891571	-3.371011
H	-1.486155	8.715532	-2.850806
H	-0.943385	10.260280	-1.024148
H	3.256139	9.446080	-1.545568
H	-0.042064	4.057659	-2.553222
N	1.489702	10.800265	-0.088773
O	2.693980	10.971819	0.250904
O	0.550399	11.373834	0.530691

41

TS(O2NBn--SiHPh2−) r²SCAN-3c SMD 4.000Å

C	-2.944688	3.333672	-0.441249
C	-3.601954	2.445418	0.406212
C	-1.602663	3.680473	-0.224216
C	-0.941292	3.112232	0.873660
C	-1.595944	2.224109	1.724271
C	-2.927538	1.888550	1.491148
H	-3.488649	3.764783	-1.280143
H	-4.642759	2.190806	0.223063
H	-1.066736	1.796244	2.571882
H	-3.440380	1.197868	2.155616
H	0.097358	3.370263	1.072401
Si	-0.712190	4.851403	-1.401454
C	0.741389	5.660526	-0.542081
C	2.060598	5.277173	-0.821648
C	0.526753	6.638847	0.440890
C	1.594444	7.215459	1.122208
C	2.899157	6.819845	0.833838
C	3.131196	5.850069	-0.138022
H	2.257731	4.526378	-1.584365
H	-0.485903	6.962912	0.675201
H	1.410649	7.984156	1.868387
H	3.733639	7.275148	1.360787
H	4.148204	5.544474	-0.370169
C	0.161053	7.433278	-4.329144
H	-1.697518	5.861556	-1.869278
C	0.520758	8.253664	-3.288847
H	-0.883482	7.277009	-4.584469
H	0.912417	6.918425	-4.921808
C	1.903897	8.473805	-2.917984
C	-0.454007	8.951684	-2.475429
C	-0.093520	9.747854	-1.426749
C	1.273227	9.929417	-1.091069
C	2.260816	9.272206	-1.871470
H	2.676948	7.970123	-3.495225
H	-1.508120	8.824676	-2.714261
H	-0.847944	10.248791	-0.828938
H	3.305159	9.405122	-1.609001
H	-0.212525	4.092186	-2.580156
N	1.637909	10.694386	0.012880
O	2.860816	10.832898	0.296134
O	0.740831	11.236796	0.717281

41

TS(O2NBn--SiHPh2−) r²SCAN-3c SMD 4.125Å

C	-2.945487	3.359856	-0.425375
C	-3.625042	2.498843	0.432662
C	-1.597096	3.680221	-0.207905
C	-0.952170	3.114795	0.901255
C	-1.629303	2.254225	1.762394
C	-2.966971	1.944050	1.528544
H	-3.476559	3.790166	-1.272806
H	-4.670477	2.264250	0.249197
H	-1.112920	1.827917	2.618636
H	-3.497249	1.274786	2.201188
H	0.091078	3.353248	1.100330
Si	-0.675126	4.810695	-1.398676
C	0.772557	5.630888	-0.539801
C	2.095575	5.281097	-0.843883
C	0.550587	6.587162	0.463364
C	1.614961	7.174757	1.140050
C	2.923820	6.812767	0.826750
C	3.163138	5.865760	-0.165359
H	2.297902	4.547450	-1.621665
H	-0.465498	6.884697	0.717511
H	1.425139	7.924347	1.903809
H	3.755563	7.276078	1.351000
H	4.183115	5.586262	-0.416485
C	0.188918	7.496328	-4.408065
H	-1.642854	5.815965	-1.912686
C	0.528465	8.282279	-3.334479
H	-0.851641	7.325315	-4.670176
H	0.952734	7.027083	-5.022226
C	1.905623	8.523302	-2.954474
C	-0.463670	8.922111	-2.494257
C	-0.123160	9.688908	-1.417560
C	1.238701	9.895553	-1.076475
C	2.242425	9.291741	-1.879028
H	2.691095	8.062098	-3.550185
H	-1.514331	8.775286	-2.736783
H	-0.890010	10.147266	-0.801697
H	3.283237	9.442708	-1.612396
H	-0.158884	4.020504	-2.549779
N	1.584138	10.637475	0.049371
O	2.803142	10.800620	0.336034
O	0.673669	11.135821	0.768813

41

TS(O2NBn--SiHPh2−) r²SCAN-3c SMD 4.250Å

C	-2.942807	3.650257	-0.329157

C	-3.713453	2.932753	0.583261
C	-1.546905	3.705797	-0.208403
C	-0.946117	3.024850	0.861047
C	-1.713191	2.307344	1.774810
C	-3.099226	2.259644	1.636503
H	-3.438207	4.177627	-1.142171
H	-4.794699	2.904129	0.475260
H	-1.230162	1.787745	2.598311
H	-3.699323	1.702854	2.351609
H	0.134715	3.059941	0.987348
Si	-0.505394	4.651933	-1.456497
C	0.921352	5.517599	-0.603504
C	2.249359	5.302355	-0.997447
C	0.678212	6.392944	0.466619
C	1.726257	7.034660	1.118657
C	3.040707	6.808727	0.713627
C	3.301106	5.942037	-0.344301
H	2.467530	4.630764	-1.825332
H	-0.342810	6.581655	0.795000
H	1.518369	7.718870	1.937115
H	3.859253	7.314305	1.219307
H	4.324714	5.767199	-0.665651
C	0.343904	7.508833	-4.486235
H	-1.403537	5.617194	-2.144443
C	0.563563	8.264156	-3.360493
H	-0.663111	7.234660	-4.788486
H	1.171511	7.168184	-5.102627
C	1.892961	8.642226	-2.926574
C	-0.516954	8.730620	-2.515307
C	-0.294500	9.457374	-1.381445
C	1.025065	9.799499	-0.987081
C	2.111427	9.374075	-1.796234
H	2.741165	8.316286	-3.525656
H	-1.537025	8.480455	-2.799756
H	-1.124321	9.781756	-0.762214
H	3.120287	9.631260	-1.490853
H	0.062058	3.726975	-2.475936
N	1.253778	10.500241	0.193634
O	2.436637	10.796772	0.521267
O	0.274949	10.828131	0.921116

41

TS(O2NBn--SiHPh2−) r²SCAN-3c SMD 4.375Å

C	-2.861152	3.825540	-0.341456
C	-3.711747	3.217984	0.580385
C	-1.469305	3.706140	-0.225745
C	-0.953413	2.963144	0.847423
C	-1.800107	2.354543	1.769213
C	-3.182022	2.481264	1.636489
H	-3.289405	4.403191	-1.158206
H	-4.788496	3.324248	0.476278
H	-1.382801	1.783360	2.594515
H	-3.844028	2.009335	2.357988
H	0.123809	2.861766	0.969967
Si	-0.317128	4.515473	-1.470627
C	1.063381	5.434717	-0.596292
C	2.403676	5.270515	-0.973805
C	0.771422	6.316873	0.455708
C	1.783537	7.018244	1.103314
C	3.110368	6.843902	0.713908
C	3.419571	5.968307	-0.323634
H	2.659624	4.592542	-1.785575
H	-0.260069	6.465385	0.771653
H	1.537691	7.709659	1.904859
H	3.900660	7.395349	1.216487
H	4.453228	5.831509	-0.631026
C	0.246594	7.573847	-4.547832
H	-1.138142	5.427999	-2.310397
C	0.463142	8.275772	-3.387622
H	-0.755064	7.270440	-4.839650
H	1.071556	7.304933	-5.201969
C	1.785970	8.690451	-2.966552
C	-0.613420	8.645509	-2.490909
C	-0.392868	9.322376	-1.326097
C	0.920214	9.704219	-0.947171
C	2.002151	9.370980	-1.804057
H	2.631306	8.435646	-3.602893
H	-1.628630	8.362803	-2.762204
H	-1.219317	9.574970	-0.669982
H	3.006448	9.657941	-1.510252
H	0.305694	3.494252	-2.357387
N	1.149737	10.351228	0.263217
O	2.327046	10.681265	0.577643
O	0.177428	10.597064	1.030982

41

TS(O2NBn--SiHPh2−) r²SCAN-3c SMD 4.500Å

C	-2.435925	4.712086	-0.581113
C	-3.503652	4.648862	0.311898
C	-1.211364	4.097298	-0.285716
C	-1.091091	3.410465	0.932931
C	-2.155302	3.346192	1.827624
C	-3.363846	3.968065	1.518443
H	-2.559372	5.249291	-1.518525
H	-4.444536	5.134469	0.066158
H	-2.042109	2.811923	2.767381
H	-4.194804	3.921194	2.217578
H	-0.153564	2.920204	1.191221
Si	0.250904	4.215464	-1.454063
C	1.615151	5.295456	-0.740947
C	2.819259	5.457744	-1.444764
C	1.480513	5.932898	0.499274
C	2.514707	6.705490	1.024367
C	3.701709	6.854955	0.313182
C	3.852553	6.230925	-0.924561
H	2.953370	4.978182	-2.413500
H	0.555152	5.833523	1.062538
H	2.385460	7.202428	1.981779
H	4.506952	7.462476	0.718463
H	4.775380	6.350643	-1.486458
C	0.315878	7.481431	-4.549097
H	-0.197441	4.731041	-2.773774
C	0.238996	7.957265	-3.263392
H	-0.498243	6.907504	-4.983376
H	1.192454	7.667589	-5.163727
C	1.307333	8.720649	-2.651416
C	-0.910786	7.718931	-2.415513
C	-0.972661	8.162188	-1.125941
C	0.100724	8.898088	-0.561685
C	1.239035	9.170116	-1.365330
H	2.196080	8.927941	-3.244445
H	-1.747195	7.161074	-2.831222
H	-1.842628	7.954750	-0.511317
H	2.061089	9.730043	-0.931927
H	0.812726	2.851018	-1.672969
N	0.062806	9.299871	0.770169
O	1.027913	9.958695	1.247160
O	-0.934488	8.996254	1.483299

Geometry scan **TS(MeOBn--CO2−) r²SCAN-3c**:

21

TS(MeOBn—CO2−) r²SCAN-3c 1.40Å

C	-3.154432	0.610494	-2.687905

C	-4.201129	0.965699	-3.547123
C	-5.448796	0.110791	-3.496335
H	-3.812427	0.909425	-4.578524
H	-4.498921	2.011684	-3.399728
C	-5.411897	-1.266629	-3.766236
C	-6.680043	0.666498	-3.161623
C	-7.851130	-0.097913	-3.094272
C	-7.786060	-1.458689	-3.374022
C	-6.560640	-2.039663	-3.710927
H	-8.783734	0.383379	-2.819705
H	-6.734898	1.729169	-2.933995
H	-6.530062	-3.105959	-3.919097
H	-4.444413	-1.710476	-3.978274
O	-8.871465	-2.316944	-3.347666
C	-10.118182	-1.755200	-2.990795
H	-10.842112	-2.574131	-3.018798
H	-10.431519	-0.972426	-3.698710
H	-10.097549	-1.326198	-1.977348
O	-2.617775	1.544855	-2.029034
O	-2.810782	-0.609952	-2.685144

21

TS(MeOBn—CO2−) r²SCAN-3c 1.55Å

C	-3.131496	0.591596	-2.574103
C	-4.222025	0.971433	-3.608014
C	-5.461793	0.133794	-3.555041
H	-3.744558	0.849710	-4.591276
H	-4.478209	2.030101	-3.495958
C	-5.425297	-1.243488	-3.832882
C	-6.690911	0.677776	-3.186640
C	-7.854049	-0.095003	-3.098401
C	-7.788107	-1.453681	-3.390270
C	-6.567040	-2.024637	-3.757430
H	-8.783847	0.378939	-2.802041
H	-6.747423	1.738410	-2.950312
H	-6.533466	-3.089741	-3.970946
H	-4.464952	-1.688793	-4.071252
O	-8.868827	-2.318626	-3.348506
C	-10.108883	-1.767488	-2.955512
H	-10.829242	-2.589927	-2.973955
H	-10.444118	-0.979182	-3.647277
H	-10.065748	-1.348383	-1.938505
O	-2.618128	1.556805	-1.963079
O	-2.859847	-0.631800	-2.503862

21

TS(MeOBn—CO2−) r²SCAN-3c 1.70Å

C	-3.106627	0.574413	-2.466216
C	-4.242611	0.978123	-3.664782
C	-5.472682	0.153821	-3.608869
H	-3.692771	0.804609	-4.597701
H	-4.460895	2.044989	-3.570526
C	-5.438401	-1.224587	-3.891419
C	-6.701111	0.687271	-3.212061
C	-7.856416	-0.092719	-3.105476
C	-7.790718	-1.450212	-3.405742
C	-6.573727	-2.012418	-3.797633
H	-8.783372	0.375399	-2.790925
H	-6.759877	1.746421	-2.969676
H	-6.537252	-3.076859	-4.014150
H	-4.484035	-1.670525	-4.152333
O	-8.867586	-2.321272	-3.349051
C	-10.100088	-1.778709	-2.923998
H	-10.817513	-2.604031	-2.931480
H	-10.455202	-0.986919	-3.602046
H	-10.035700	-1.366045	-1.905285
O	-2.602631	1.563688	-1.909481
O	-2.908749	-0.646626	-2.346411

21

TS(MeOBn—CO2−) r²SCAN-3c 1.85Å

C	-3.092797	0.567566	-2.350498
C	-4.258330	0.979755	-3.726774
C	-5.478107	0.167091	-3.663931
H	-3.640500	0.750111	-4.599918
H	-4.443387	2.052877	-3.653838
C	-5.452003	-1.212364	-3.954839
C	-6.704735	0.692848	-3.237696
C	-7.853590	-0.091432	-3.112756
C	-7.793138	-1.447738	-3.422528
C	-6.582838	-2.003548	-3.841990
H	-8.775435	0.373126	-2.777808
H	-6.762443	1.750215	-2.987447
H	-6.546894	-3.066801	-4.064880
H	-4.505643	-1.660113	-4.242294
O	-8.868508	-2.322358	-3.349646
C	-10.089699	-1.785403	-2.888987
H	-10.806333	-2.611630	-2.883795
H	-10.464173	-0.988900	-3.551339
H	-9.999803	-1.379641	-1.869185
O	-2.629531	1.580163	-1.823835
O	-2.940080	-0.646009	-2.211276

21

TS(MeOBn—CO2−) r²SCAN-3c 2.00Å

C	-3.065408	0.566120	-2.245307
C	-4.276134	0.982782	-3.781711
C	-5.483452	0.177076	-3.706929
H	-3.604973	0.712627	-4.599431
H	-4.433242	2.059302	-3.714984
C	-5.465957	-1.203811	-4.007337
C	-6.709592	0.695292	-3.256076
C	-7.852731	-0.092756	-3.117242
C	-7.797353	-1.447777	-3.436162
C	-6.592933	-1.997348	-3.879660
H	-8.769752	0.368671	-2.764444
H	-6.767050	1.750868	-2.998103
H	-6.558009	-3.059146	-4.110288
H	-4.526645	-1.651756	-4.318625
O	-8.871400	-2.326174	-3.349835
C	-10.083281	-1.792826	-2.863969
H	-10.800057	-2.619063	-2.849890
H	-10.470790	-0.992308	-3.514363
H	-9.975162	-1.392130	-1.843625
O	-2.600134	1.599059	-1.786234
O	-2.983911	-0.638888	-2.071046

21

TS(MeOBn—CO2−) r²SCAN-3c 2.15Å

C	-3.029511	0.569328	-2.146422
C	-4.294273	0.985526	-3.834514
C	-5.488148	0.183245	-3.742869
H	-3.579658	0.684336	-4.600475
H	-4.428154	2.063641	-3.764685
C	-5.479744	-1.199281	-4.052397
C	-6.714165	0.695345	-3.270742
C	-7.852509	-0.095432	-3.120444
C	-7.802735	-1.449432	-3.447274
C	-6.603919	-1.993872	-3.911858
H	-8.764768	0.363775	-2.752001
H	-6.770812	1.749314	-3.005910
H	-6.570742	-3.054147	-4.150506
H	-4.547599	-1.647641	-4.385655
O	-8.876313	-2.330730	-3.349288

C	-10.079902	-1.798834	-2.844446
H	-10.797804	-2.624191	-2.823195
H	-10.476384	-0.994871	-3.485645
H	-9.957475	-1.401657	-1.824010
O	-2.561828	1.618492	-1.754472
O	-3.011523	-0.625104	-1.948453

21

TS(MeOBn—CO2−) r²SCAN-3c 2.30Å

C	-2.986809	0.573899	-2.052081
C	-4.312449	0.988596	-3.885303
C	-5.493327	0.187967	-3.774890
H	-3.561331	0.663474	-4.603014
H	-4.425670	2.067207	-3.804128
C	-5.493352	-1.196789	-4.091488
C	-6.719846	0.694962	-3.285313
C	-7.853848	-0.098229	-3.124873
C	-7.809036	-1.451699	-3.457003
C	-6.615104	-1.991823	-3.939389
H	-8.761886	0.359261	-2.743378
H	-6.776086	1.747772	-3.015570
H	-6.583711	-3.050883	-4.184546
H	-4.567759	-1.645426	-4.443589
O	-8.882074	-2.335990	-3.347625
C	-10.078362	-1.804546	-2.827939
H	-10.797404	-2.628957	-2.799657
H	-10.482015	-0.998325	-3.462335
H	-9.944131	-1.408932	-1.808028
O	-2.515150	1.635035	-1.726833
O	-3.028616	-0.608762	-1.838279

21

TS(MeOBn—CO2−) r²SCAN-3c 2.45Å

C	-2.940289	0.571348	-1.962416
C	-4.330602	0.994532	-3.934838
C	-5.499669	0.193851	-3.805401
H	-3.550600	0.651788	-4.610706
H	-4.427257	2.072669	-3.838878
C	-5.505381	-1.194199	-4.123461
C	-6.727642	0.696872	-3.303848
C	-7.857328	-0.099013	-3.133288
C	-7.815176	-1.453121	-3.465179
C	-6.624406	-1.989940	-3.959958
H	-8.762359	0.357644	-2.743092
H	-6.784912	1.749550	-3.033460
H	-6.593258	-3.048745	-4.207152
H	-4.584504	-1.643056	-4.488528
O	-8.886871	-2.340896	-3.343931
C	-10.076763	-1.809860	-2.811956
H	-10.795801	-2.634157	-2.774293
H	-10.488354	-1.004038	-3.442291
H	-9.931829	-1.412225	-1.793947
O	-2.481850	1.642412	-1.679719
O	-3.023117	-0.603601	-1.758918

21

TS(MeOBn—CO2−) r²SCAN-3c 2.60Å

C	-2.886429	0.567972	-1.875349
C	-4.348565	1.000262	-3.981361
C	-5.507307	0.198910	-3.833378
H	-3.544852	0.642982	-4.619823
H	-4.432132	2.077634	-3.871250
C	-5.517154	-1.192721	-4.150228
C	-6.736742	0.699153	-3.322575
C	-7.862458	-0.099238	-3.142327
C	-7.821907	-1.454537	-3.471444
C	-6.633618	-1.989052	-3.975654
H	-8.765213	0.356936	-2.745745
H	-6.795434	1.752231	-3.053763
H	-6.602512	-3.048056	-4.222885
H	-4.600357	-1.641986	-4.525746
O	-8.892253	-2.345578	-3.338776
C	-10.077315	-1.813951	-2.799012
H	-10.796175	-2.638198	-2.752609
H	-10.494835	-1.009763	-3.428035
H	-9.924613	-1.412478	-1.783307
O	-2.461875	1.651823	-1.616472
O	-2.986219	-0.604532	-1.705522

21

TS(MeOBn—CO2−) r²SCAN-3c 2.75Å

C	-2.830691	0.562354	-1.789198
C	-4.366354	1.006802	-4.026764
C	-5.515420	0.204282	-3.860672
H	-3.543360	0.638262	-4.632749
H	-4.439080	2.083088	-3.902486
C	-5.528442	-1.191184	-4.174391
C	-6.746266	0.701771	-3.341416
C	-7.867938	-0.099284	-3.151169
C	-7.828267	-1.455937	-3.476274
C	-6.642080	-1.988331	-3.988693
H	-8.768693	0.356570	-2.749124
H	-6.806974	1.755520	-3.075333
H	-6.610506	-3.047776	-4.234845
H	-4.615344	-1.640822	-4.559124
O	-8.896886	-2.350456	-3.332091
C	-10.078180	-1.817834	-2.787394
H	-10.796709	-2.642094	-2.732540
H	-10.500651	-1.016132	-3.416793
H	-9.919752	-1.411247	-1.774261
O	-2.442481	1.658228	-1.551877
O	-2.943893	-0.607968	-1.658068

21

TS(MeOBn—CO2−) r²SCAN-3c 2.90Å

C	-2.781416	0.548619	-1.702056
C	-4.383642	1.016167	-4.073611
C	-5.523557	0.211683	-3.889718
H	-3.545053	0.638867	-4.651830
H	-4.448763	2.091489	-3.938417
C	-5.537935	-1.187914	-4.197311
C	-6.755768	0.706379	-3.363021
C	-7.872781	-0.097891	-3.161324
C	-7.832459	-1.456400	-3.479813
C	-6.647917	-1.986651	-3.999149
H	-8.771983	0.357530	-2.754790
H	-6.819388	1.761235	-3.101633
H	-6.614833	-3.047032	-4.241897
H	-4.627891	-1.637770	-4.589627
O	-8.898378	-2.355010	-3.322724
C	-10.076477	-1.821652	-2.774011
H	-10.793685	-2.646579	-2.709241
H	-10.504766	-1.024046	-3.405164
H	-9.912729	-1.408023	-1.764249
O	-2.430037	1.654090	-1.478759
O	-2.908509	-0.619277	-1.616917

21

TS(MeOBn—CO2−) r²SCAN-3c 3.05Å

C	-3.111650	0.431522	-1.523451
C	-4.336642	1.001821	-4.257798
C	-5.472064	0.213229	-4.013037
H	-3.493269	0.591288	-4.804588
H	-4.379489	2.078956	-4.127877

C	-5.519798	-1.192778	-4.305586
C	-6.678685	0.725801	-3.436612
C	-7.789754	-0.066892	-3.168648
C	-7.777850	-1.430942	-3.466382
C	-6.625179	-1.977561	-4.039212
H	-8.663686	0.403089	-2.724662
H	-6.720597	1.783749	-3.183770
H	-6.614769	-3.040538	-4.273917
H	-4.637972	-1.656769	-4.744242
O	-8.844761	-2.319273	-3.244906
C	-9.987658	-1.765309	-2.647110
H	-10.709848	-2.580887	-2.533270
H	-10.439253	-0.972107	-3.268147
H	-9.770040	-1.337239	-1.653215
O	-3.592478	1.340913	-0.959914
O	-2.522523	-0.532260	-1.838917

21

TS(MeOBn—CO2−) r^{2}SCAN-3c SMD 1.40Å

C	-3.372320	0.709950	-2.495888
C	-4.120643	0.877108	-3.667243
C	-5.405935	0.073253	-3.618770
H	-3.549571	0.543996	-4.544694
H	-4.387757	1.928838	-3.828018
C	-5.424133	-1.284449	-3.963850
C	-6.596080	0.644681	-3.172346
C	-7.775710	-0.096676	-3.063834
C	-7.769541	-1.448180	-3.412566
C	-6.584498	-2.038283	-3.867729
H	-8.678247	0.392223	-2.713707
H	-6.612213	1.697125	-2.898136
H	-6.591437	-3.089599	-4.143495
H	-4.507723	-1.754688	-4.310021
O	-8.859556	-2.270747	-3.353001
C	-10.082271	-1.705232	-2.878522
H	-10.814604	-2.515318	-2.897828
H	-10.427943	-0.890781	-3.528966
H	-9.980884	-1.334851	-1.849714
O	-3.468414	1.607596	-1.604880
O	-2.678485	-0.348151	-2.402052

21

TS(MeOBn—CO2−) r^{2}SCAN-3c SMD 1.55Å

C	-3.336819	0.701284	-2.403302
C	-4.141834	0.879278	-3.715845
C	-5.414331	0.082115	-3.659620
H	-3.527440	0.535004	-4.556047
H	-4.377152	1.939096	-3.859127
C	-5.436550	-1.278526	-4.000483
C	-6.604735	0.648531	-3.200760
C	-7.779854	-0.096418	-3.075370
C	-7.774319	-1.449415	-3.418802
C	-6.592698	-2.036345	-3.885860
H	-8.679911	0.391196	-2.716925
H	-6.622508	1.702033	-2.929906
H	-6.598749	-3.088844	-4.157344
H	-4.524834	-1.749531	-4.359037
O	-8.861733	-2.275648	-3.343272
C	-10.080484	-1.710551	-2.859247
H	-10.811243	-2.522318	-2.866228
H	-10.435143	-0.900476	-3.510489
H	-9.969593	-1.333873	-1.833628
O	-3.460545	1.614551	-1.547713
O	-2.657489	-0.353330	-2.316256

21

TS(MeOBn—CO2−) r^{2}SCAN-3c SMD 1.70Å

C	-3.302032	0.691738	-2.312417
C	-4.162808	0.883070	-3.765846
C	-5.422427	0.091417	-3.699075
H	-3.511609	0.527807	-4.570562
H	-4.369702	1.949737	-3.888907
C	-5.447599	-1.273033	-4.033654
C	-6.613445	0.653184	-3.228468
C	-7.783504	-0.095850	-3.086271
C	-7.778193	-1.450539	-3.423642
C	-6.599452	-2.034363	-3.901111
H	-8.681184	0.390466	-2.719849
H	-6.632959	1.707730	-2.961139
H	-6.604179	-3.088181	-4.167867
H	-4.539897	-1.744160	-4.403016
O	-8.862941	-2.280758	-3.332618
C	-10.078259	-1.715588	-2.841727
H	-10.807845	-2.528520	-2.838112
H	-10.440189	-0.909516	-3.494175
H	-9.960089	-1.332818	-1.819049
O	-3.451060	1.617126	-1.490017
O	-2.638594	-0.361137	-2.237738

21

TS(MeOBn—CO2−) r^{2}SCAN-3c SMD 1.85Å

C	-3.259783	0.673778	-2.226957
C	-4.186492	0.893357	-3.812989
C	-5.431672	0.104562	-3.733682
H	-3.506320	0.534379	-4.589519
H	-4.369158	1.966154	-3.905421
C	-5.456204	-1.265560	-4.057003
C	-6.626788	0.661272	-3.258409
C	-7.790567	-0.093707	-3.100801
C	-7.781674	-1.451256	-3.426734
C	-6.602879	-2.031346	-3.908485
H	-8.688076	0.391074	-2.731523
H	-6.652101	1.718007	-2.999586
H	-6.603348	-3.087389	-4.166938
H	-4.550065	-1.736584	-4.430899
O	-8.862461	-2.287474	-3.320542
C	-10.076390	-1.722832	-2.827685
H	-10.803496	-2.537973	-2.812854
H	-10.445422	-0.923262	-3.484444
H	-9.954285	-1.330659	-1.808852
O	-3.407438	1.613899	-1.437338
O	-2.633348	-0.390626	-2.174599

21

TS(MeOBn—CO2−) r^{2}SCAN-3c SMD 2.00Å

C	-3.227274	0.662094	-2.134608
C	-4.205164	0.898254	-3.863179
C	-5.437437	0.114170	-3.770494
H	-3.494505	0.528828	-4.604782
H	-4.363599	1.975460	-3.933917
C	-5.465747	-1.260775	-4.087807
C	-6.635512	0.667292	-3.287774
C	-7.794109	-0.091578	-3.115369
C	-7.785226	-1.451150	-3.433489
C	-6.608697	-2.028426	-3.924076
H	-8.689713	0.392622	-2.740232
H	-6.663796	1.725245	-3.033688
H	-6.607716	-3.085749	-4.177969
H	-4.563092	-1.732840	-4.469476
O	-8.863375	-2.291823	-3.311526
C	-10.072194	-1.727589	-2.808278
H	-10.797924	-2.543783	-2.780983
H	-10.450881	-0.931708	-3.464372
H	-9.940058	-1.329615	-1.792717

O	-3.408175	1.608074	-1.374981
O	-2.613773	-0.399188	-2.105545

21

TS(MeOBn—CO2−) r^{2}SCAN-3c SMD 2.15Å

C	-3.195178	0.706671	-2.028028
C	-4.195916	0.833330	-3.926707
C	-5.431304	0.079755	-3.815102
H	-3.454409	0.431106	-4.617014
H	-4.316710	1.914746	-4.010623
C	-5.493308	-1.297076	-4.135635
C	-6.614282	0.650391	-3.308563
C	-7.781282	-0.091302	-3.116815
C	-7.803346	-1.449337	-3.438844
C	-6.645624	-2.044734	-3.954072
H	-8.661562	0.404723	-2.720766
H	-6.619823	1.706985	-3.050116
H	-6.666918	-3.101299	-4.210791
H	-4.605424	-1.785948	-4.533073
O	-8.893082	-2.274766	-3.295050
C	-10.086036	-1.687641	-2.782151
H	-10.827053	-2.489849	-2.747935
H	-10.454580	-0.884054	-3.434804
H	-9.938369	-1.292173	-1.767488
O	-3.919808	1.138972	-1.152140
O	-2.083951	0.229315	-2.169544

21

TS(MeOBn—CO2−) r^{2}SCAN-3c SMD 2.30Å

C	-3.052209	0.764693	-1.959836
C	-4.206249	0.773897	-3.949335
C	-5.438089	0.038654	-3.823033
H	-3.435964	0.341993	-4.587169
H	-4.288873	1.857456	-4.031323
C	-5.521912	-1.345381	-4.118524
C	-6.621014	0.629337	-3.329109
C	-7.801841	-0.089455	-3.143864
C	-7.844603	-1.451128	-3.451189
C	-6.688871	-2.070913	-3.940715
H	-8.676309	0.427021	-2.760996
H	-6.613254	1.688234	-3.076263
H	-6.722361	-3.131237	-4.180743
H	-4.638264	-1.856694	-4.497046
O	-8.951670	-2.256279	-3.312011
C	-10.130746	-1.647046	-2.794898
H	-10.885814	-2.435741	-2.752155
H	-10.491296	-0.840579	-3.448898
H	-9.970094	-1.248180	-1.783411
O	-3.743339	0.307032	-1.081510
O	-1.965196	1.242129	-2.193234

21

TS(MeOBn—CO2−) r^{2}SCAN-3c SMD 2.45Å

C	-2.996156	0.753847	-1.879380
C	-4.226983	0.784648	-3.997543
C	-5.445987	0.049297	-3.851343
H	-3.438489	0.349060	-4.608639
H	-4.291136	1.870842	-4.038126
C	-5.529542	-1.342068	-4.132346
C	-6.635758	0.637189	-3.357875
C	-7.811036	-0.086915	-3.160283
C	-7.849991	-1.452204	-3.452381
C	-6.692340	-2.069850	-3.940820
H	-8.686410	0.429792	-2.779137
H	-6.634580	1.698861	-3.116178
H	-6.720860	-3.133073	-4.169424
H	-4.646060	-1.854863	-4.509620
O	-8.953929	-2.263497	-3.298566
C	-10.131957	-1.652935	-2.783906
H	-10.884753	-2.443207	-2.728183
H	-10.498991	-0.854979	-3.445196
H	-9.969132	-1.240909	-1.777721
O	-3.710371	0.270170	-1.046129
O	-1.933505	1.248608	-2.142466

21

TS(MeOBn—CO2−) r^{2}SCAN-3c SMD 2.60Å

C	-2.948548	0.748479	-1.792362
C	-4.244144	0.793353	-4.046119
C	-5.452367	0.058816	-3.881081
H	-3.438329	0.350823	-4.627793
H	-4.290166	1.880580	-4.052279
C	-5.537440	-1.338600	-4.152327
C	-6.647436	0.643500	-3.384190
C	-7.817539	-0.085477	-3.174542
C	-7.854431	-1.453289	-3.455720
C	-6.696405	-2.068158	-3.947561
H	-8.692541	0.431112	-2.791656
H	-6.651459	1.706967	-3.149839
H	-6.721164	-3.133270	-4.168850
H	-4.655433	-1.852376	-4.532154
O	-8.955646	-2.270311	-3.287774
C	-10.130511	-1.659610	-2.769720
H	-10.881511	-2.450893	-2.701943
H	-10.505439	-0.867237	-3.433843
H	-9.962347	-1.238544	-1.767786
O	-3.706631	0.273516	-1.006408
O	-1.898480	1.228433	-2.091315

21

TS(MeOBn—CO2−) r^{2}SCAN-3c SMD 2.75Å

C	-2.914913	0.736853	-1.698853
C	-4.258575	0.803536	-4.097316
C	-5.456664	0.069798	-3.914485
H	-3.434997	0.353530	-4.646793
H	-4.286982	1.890790	-4.069557
C	-5.543294	-1.333773	-4.175572
C	-6.656915	0.651315	-3.413475
C	-7.821391	-0.082757	-3.190299
C	-7.856049	-1.453170	-3.459722
C	-6.698170	-2.065154	-3.956119
H	-8.695894	0.433498	-2.805020
H	-6.666415	1.716529	-3.186592
H	-6.719398	-3.132188	-4.169720
H	-4.663251	-1.848786	-4.558747
O	-8.954391	-2.276201	-3.276028
C	-10.124459	-1.665856	-2.750563
H	-10.873082	-2.458330	-2.668720
H	-10.509633	-0.878851	-3.415807
H	-9.947788	-1.236171	-1.753337
O	-3.730975	0.280457	-0.975704
O	-1.874729	1.192744	-2.032831

21

TS(MeOBn—CO2−) r^{2}SCAN-3c SMD 2.90Å

C	-2.869981	0.727989	-1.615614
C	-4.277095	0.814742	-4.149880
C	-5.462458	0.079453	-3.943606
H	-3.426285	0.355211	-4.646167
H	-4.280318	1.899036	-4.066634
C	-5.549827	-1.330687	-4.192557
C	-6.668232	0.657443	-3.438300
C	-7.827181	-0.081369	-3.203099
C	-7.859594	-1.454341	-3.461025

C	-6.700923	-2.063575	-3.959601
H	-8.701351	0.434817	-2.816128
H	-6.683465	1.724459	-3.219370
H	-6.717907	-3.132688	-4.164516
H	-4.671271	-1.847116	-4.577798
O	-8.955694	-2.283038	-3.263481
C	-10.123329	-1.670943	-2.738678
H	-10.871024	-2.463384	-2.645929
H	-10.513903	-0.889873	-3.408423
H	-9.944064	-1.231180	-1.745852
O	-3.725883	0.267676	-0.957488
O	-1.858182	1.185185	-2.001114

21

TS(MeOBn—CO2−) r^2SCAN-3c SMD 3.05Å

C	-2.768054	0.692288	-1.579602
C	-4.314366	0.841375	-4.204329
C	-5.482873	0.096877	-3.966922
H	-3.404771	0.356991	-4.549193
H	-4.282422	1.910411	-4.010788
C	-5.557102	-1.323006	-4.179540
C	-6.701664	0.673036	-3.481785
C	-7.854748	-0.072084	-3.238876
C	-7.873012	-1.451512	-3.463021
C	-6.703600	-2.060123	-3.936821
H	-8.736275	0.444972	-2.869620
H	-6.730757	1.744980	-3.289271
H	-6.707007	-3.134769	-4.112427
H	-4.671218	-1.841838	-4.544523
O	-8.964178	-2.287462	-3.252184
C	-10.135048	-1.675047	-2.737713
H	-10.876243	-2.471320	-2.625933
H	-10.534872	-0.911252	-3.422215
H	-9.957650	-1.212206	-1.754755
O	-3.566549	0.073813	-0.993032
O	-1.865555	1.303688	-2.002712

Geometry scan **TS(MeBn--CO2−) r^2SCAN-3c**:

20

TS(MeBn—CO2−) r^2SCAN-3c 1.40Å

C	-3.524497	-0.203414	-2.026613
C	-3.972777	-0.488605	-3.321878
C	-5.470959	-0.574973	-3.512175
H	-3.528125	-1.416819	-3.702510
H	-3.610544	0.316353	-3.983668
C	-6.064254	-1.750714	-3.974201
C	-6.313683	0.508099	-3.224083
C	-7.687106	0.407125	-3.400924
C	-8.280718	-0.773176	-3.864359
C	-7.444991	-1.850911	-4.147828
H	-8.320106	1.263834	-3.170083
H	-5.854977	1.406906	-2.823506
H	-7.876088	-2.785086	-4.505786
H	-5.432079	-2.608152	-4.196462
C	-9.776245	-0.876441	-4.020563
O	-4.006136	0.832552	-1.476640
O	-2.656081	-0.980254	-1.539581
H	-10.188793	0.009193	-4.518279
H	-10.275929	-0.961219	-3.046523
H	-10.053592	-1.755993	-4.611107

20

TS(MeBn—CO2−) r^2SCAN-3c 1.55Å

C	-3.527642	-0.245889	-1.921480
C	-3.979863	-0.466493	-3.387539
C	-5.461056	-0.564105	-3.572027
H	-3.490662	-1.358859	-3.791277
H	-3.603071	0.403833	-3.943612
C	-6.065351	-1.754533	-3.985670
C	-6.302907	0.525984	-3.299711
C	-7.678875	0.420693	-3.447214
C	-8.280376	-0.772538	-3.865019
C	-7.448243	-1.858702	-4.129968
H	-8.307739	1.283404	-3.227760
H	-5.847174	1.440866	-2.934597
H	-7.885499	-2.802514	-4.453851
H	-5.437109	-2.618250	-4.194327
C	-9.778171	-0.879835	-3.993458
O	-4.083275	0.710495	-1.328912
O	-2.633252	-1.023017	-1.516642
H	-10.199164	-0.012739	-4.516428
H	-10.263383	-0.929654	-3.009539
H	-10.064866	-1.779841	-4.547737

20

TS(MeBn—CO2−) r^2SCAN-3c 1.70Å

C	-3.525238	-0.284504	-1.820788
C	-3.987058	-0.448193	-3.448648
C	-5.452325	-0.552798	-3.625116
H	-3.461949	-1.314920	-3.857692
H	-3.597324	0.466570	-3.910391
C	-6.066854	-1.755472	-3.997596
C	-6.296372	0.541751	-3.366244
C	-7.673904	0.432300	-3.487860
C	-8.281636	-0.770736	-3.866439
C	-7.450425	-1.862600	-4.116337
H	-8.299477	1.299854	-3.277913
H	-5.843251	1.471014	-3.035432
H	-7.891652	-2.814299	-4.411089
H	-5.442086	-2.624277	-4.195547
C	-9.780910	-0.882747	-3.969194
O	-4.146598	0.599070	-1.207688
O	-2.601503	-1.045569	-1.490580
H	-10.212292	-0.028637	-4.505273
H	-10.252710	-0.912193	-2.977672
H	-10.074114	-1.795310	-4.499268

20

TS(MeBn—CO2−) r^2SCAN-3c 1.85Å

C	-3.522492	-0.328314	-1.723828
C	-3.993002	-0.425037	-3.510379
C	-5.443622	-0.536413	-3.673383
H	-3.438154	-1.270693	-3.919661
H	-3.593912	0.524171	-3.878875
C	-6.065619	-1.751551	-4.007831
C	-6.293670	0.560619	-3.429623
C	-7.671952	0.444212	-3.527413
C	-8.282725	-0.768687	-3.867954
C	-7.448819	-1.863522	-4.102829
H	-8.296326	1.315421	-3.328533
H	-5.844279	1.503416	-3.131983
H	-7.890954	-2.823515	-4.368890
H	-5.442267	-2.623907	-4.194462
C	-9.782714	-0.889206	-3.944886
O	-4.174832	0.517531	-1.113814
O	-2.604221	-1.101985	-1.451656
H	-10.227964	-0.045496	-4.486502
H	-10.240947	-0.908089	-2.946372
H	-10.079207	-1.810650	-4.457896

20

TS(MeBn—CO2−) r^2SCAN-3c 2.00Å

C	-3.509919	-0.367496	-1.629214

C	-3.999525	-0.404696	-3.568003
C	-5.437282	-0.521710	-3.712836
H	-3.421911	-1.237481	-3.968158
H	-3.592076	0.566155	-3.855852
C	-6.065902	-1.747093	-4.016183
C	-6.294734	0.577482	-3.484294
C	-7.672947	0.454454	-3.563011
C	-8.285842	-0.766525	-3.870659
C	-7.448237	-1.862976	-4.092163
H	-8.296900	1.328824	-3.375762
H	-5.848948	1.531606	-3.216739
H	-7.889989	-2.829906	-4.333814
H	-5.443587	-2.622334	-4.192958
C	-9.785971	-0.894612	-3.925167
O	-4.194398	0.443260	-1.028480
O	-2.588014	-1.140303	-1.417388
H	-10.243863	-0.057567	-4.467087
H	-10.232879	-0.909589	-2.920990
H	-10.084755	-1.821189	-4.428010

20

TS(MeBn—CO2−) r²SCAN-3c 2.15Å

C	-3.489710	-0.396586	-1.535952
C	-4.006189	-0.389496	-3.622983
C	-5.433082	-0.510884	-3.746262
H	-3.411173	-1.217252	-4.003982
H	-3.589716	0.593042	-3.843857
C	-6.068252	-1.743985	-4.025669
C	-6.297455	0.590322	-3.529890
C	-7.675254	0.462186	-3.593152
C	-8.290517	-0.764545	-3.874481
C	-7.449626	-1.862013	-4.086232
H	-8.298643	1.339273	-3.415476
H	-5.854602	1.553082	-3.287515
H	-7.890999	-2.834034	-4.308769
H	-5.447231	-2.621598	-4.195757
C	-9.790468	-0.898373	-3.909111
O	-4.208849	0.381235	-0.951610
O	-2.557183	-1.156207	-1.381540
H	-10.259538	-0.062264	-4.443368
H	-10.227214	-0.918764	-2.900027
H	-10.091978	-1.824835	-4.411135

20

TS(MeBn—CO2−) r²SCAN-3c 2.30Å

C	-3.468695	-0.405032	-1.440783
C	-4.011346	-0.383213	-3.675745
C	-5.429554	-0.505205	-3.776717
H	-3.405198	-1.216723	-4.022192
H	-3.582823	0.601862	-3.851073
C	-6.073067	-1.747254	-4.021382
C	-6.298313	0.601801	-3.587793
C	-7.676361	0.470959	-3.639018
C	-8.295448	-0.761807	-3.882216
C	-7.452830	-1.864823	-4.070328
H	-8.298023	1.353675	-3.483531
H	-5.856885	1.573953	-3.380988
H	-7.895316	-2.842662	-4.265447
H	-5.454249	-2.629715	-4.174587
C	-9.794740	-0.903388	-3.891700
O	-4.214071	0.359184	-0.889861
O	-2.535635	-1.158642	-1.329061
H	-10.280277	0.009198	-4.258795
H	-10.202135	-1.102200	-2.888865
H	-10.112713	-1.731665	-4.536685

20

TS(MeBn—CO2−) r²SCAN-3c 2.45Å

C	-3.443351	-0.418661	-1.347502
C	-4.016467	-0.374926	-3.729124
C	-5.427410	-0.499610	-3.806529
H	-3.400073	-1.210590	-4.048808
H	-3.579260	0.612847	-3.854736
C	-6.076946	-1.745675	-4.038200
C	-6.301851	0.608166	-3.619395
C	-7.679198	0.474081	-3.657797
C	-8.300988	-0.761073	-3.886347
C	-7.456012	-1.863699	-4.073965
H	-8.300156	1.357951	-3.504081
H	-5.862136	1.584445	-3.426933
H	-7.898306	-2.843994	-4.258552
H	-5.459768	-2.629307	-4.192275
C	-9.799666	-0.905524	-3.878656
O	-4.219313	0.324251	-0.826646
O	-2.502834	-1.156898	-1.283142
H	-10.291209	0.005353	-4.242834
H	-10.199194	-1.103560	-2.871921
H	-10.123541	-1.735272	-4.519324

20

TS(MeBn—CO2−) r²SCAN-3c 2.60Å

C	-3.415979	-0.430102	-1.251894
C	-4.021135	-0.365794	-3.779670
C	-5.426361	-0.493684	-3.835276
H	-3.397441	-1.203550	-4.077589
H	-3.577941	0.623184	-3.862584
C	-6.080272	-1.743478	-4.056365
C	-6.306390	0.614139	-3.647907
C	-7.682999	0.476238	-3.674265
C	-8.306312	-0.761242	-3.890738
C	-7.458668	-1.862770	-4.079813
H	-8.303984	1.360515	-3.521084
H	-5.868845	1.594128	-3.468008
H	-7.900258	-2.845235	-4.255962
H	-5.463974	-2.627470	-4.212818
C	-9.804238	-0.909209	-3.866955
O	-4.199409	0.321273	-0.770974
O	-2.492406	-1.181291	-1.226516
H	-10.301820	0.000746	-4.225972
H	-10.195892	-1.109133	-2.856931
H	-10.133353	-1.738960	-4.505445

20

TS(MeBn—CO2−) r²SCAN-3c 2.75Å

C	-3.430791	-0.445699	-1.157635
C	-4.017804	-0.344881	-3.842361
C	-5.418010	-0.480894	-3.871299
H	-3.386853	-1.181861	-4.126075
H	-3.574091	0.646012	-3.884354
C	-6.072368	-1.736099	-4.082384
C	-6.306587	0.623320	-3.676922
C	-7.681891	0.476417	-3.684375
C	-8.303087	-0.765171	-3.888638
C	-7.449754	-1.861813	-4.086638
H	-8.305516	1.358396	-3.526719
H	-5.873952	1.607952	-3.509341
H	-7.887634	-2.847624	-4.254898
H	-5.454636	-2.617844	-4.246521
C	-9.799379	-0.922004	-3.842140
O	-4.117654	0.428163	-0.757165
O	-2.638487	-1.325528	-1.131500
H	-10.307474	-0.012064	-4.186913
H	-10.177239	-1.132162	-2.828362
H	-10.134471	-1.748311	-4.482528

20

TS(MeBn—CO2−) r^2SCAN-3c 2.90Å

C	-3.432943	-0.457983	-1.059785
C	-4.016711	-0.335716	-3.897789
C	-5.412585	-0.474547	-3.905951
H	-3.382377	-1.175370	-4.165515
H	-3.568928	0.654011	-3.910749
C	-6.070762	-1.732686	-4.107209
C	-6.305556	0.628772	-3.704818
C	-7.679923	0.478142	-3.696064
C	-8.302499	-0.765370	-3.889433
C	-7.447497	-1.860162	-4.094833
H	-8.303258	1.359867	-3.534162
H	-5.874836	1.616254	-3.547616
H	-7.884880	-2.847687	-4.255803
H	-5.454266	-2.614178	-4.278189
C	-9.797432	-0.925607	-3.822078
O	-4.123519	0.427886	-0.711589
O	-2.663405	-1.350943	-1.062646
H	-10.312805	-0.019160	-4.166146
H	-10.164444	-1.130868	-2.802766
H	-10.139054	-1.756351	-4.453628

20

TS(MeBn—CO2−) r^2SCAN-3c 3.05Å

C	-3.831572	-0.603367	-1.027707
C	-3.955321	-0.222542	-4.051308
C	-5.341665	-0.399661	-3.993167
H	-3.301831	-1.056730	-4.288614
H	-3.525680	0.774736	-4.044357
C	-5.974805	-1.684810	-4.115892
C	-6.264144	0.686000	-3.799311
C	-7.630816	0.494968	-3.721027
C	-8.225201	-0.773106	-3.833389
C	-7.344603	-1.849473	-4.034110
H	-8.272975	1.365513	-3.569833
H	-5.859802	1.693278	-3.708767
H	-7.757404	-2.855851	-4.133218
H	-5.341185	-2.556053	-4.276450
C	-9.709504	-0.973868	-3.688276
O	-3.951596	0.530061	-0.760242
O	-3.700466	-1.766126	-1.060971
H	-10.267588	-0.094004	-4.035706
H	-10.025179	-1.157020	-2.647080
H	-10.056341	-1.833639	-4.277344

20

TS(MeBn—CO2−) r^2SCAN-3c SMD 1.40Å

C	-3.602588	-0.520815	-2.022646
C	-3.959030	-0.287799	-3.356307
C	-5.453222	-0.448307	-3.559643
H	-3.450471	-0.985035	-4.033880
H	-3.688510	0.731420	-3.662042
C	-6.006553	-1.694154	-3.863810
C	-6.324828	0.634826	-3.401323
C	-7.699447	0.475270	-3.543265
C	-8.255577	-0.773659	-3.843536
C	-7.384549	-1.853863	-4.002167
H	-8.354882	1.336350	-3.422386
H	-5.915504	1.614016	-3.165298
H	-7.788452	-2.835454	-4.243017
H	-5.350188	-2.550939	-3.996694
C	-9.744804	-0.940480	-3.981204
O	-3.736833	0.445771	-1.212669
O	-3.205748	-1.686143	-1.717805
H	-10.170730	-0.186262	-4.652741
H	-10.247030	-0.826871	-3.012235
H	-9.998732	-1.929567	-4.374101

20

TS(MeBn—CO2−) r^2SCAN-3c SMD 1.55Å

C	-3.592030	-0.544794	-1.928758
C	-3.968749	-0.274583	-3.407801
C	-5.448970	-0.436543	-3.602535
H	-3.431683	-0.979252	-4.051467
H	-3.672728	0.747621	-3.668719
C	-6.007104	-1.685359	-3.896312
C	-6.326743	0.643501	-3.437718
C	-7.702426	0.478371	-3.558473
C	-8.259568	-0.773145	-3.846166
C	-7.385789	-1.850098	-4.014047
H	-8.358730	1.337992	-3.431157
H	-5.919737	1.626782	-3.213618
H	-7.789303	-2.834084	-4.246146
H	-5.350945	-2.541489	-4.036377
C	-9.750070	-0.945997	-3.961707
O	-3.731085	0.425353	-1.141896
O	-3.207773	-1.711111	-1.659626
H	-10.187872	-0.201929	-4.637110
H	-10.240562	-0.822271	-2.987912
H	-10.005811	-1.940662	-4.339222

20

TS(MeBn—CO2−) r^2SCAN-3c SMD 1.70Å

C	-3.582955	-0.566241	-1.835680
C	-3.977351	-0.259743	-3.460645
C	-5.444214	-0.425617	-3.643590
H	-3.414923	-0.973257	-4.069111
H	-3.661519	0.764233	-3.680332
C	-6.006521	-1.677521	-3.929338
C	-6.328649	0.650928	-3.469443
C	-7.704783	0.479841	-3.569718
C	-8.262603	-0.773705	-3.848040
C	-7.385618	-1.846792	-4.027101
H	-8.361922	1.337760	-3.434216
H	-5.924570	1.637889	-3.255100
H	-7.788221	-2.832819	-4.252782
H	-5.350501	-2.532555	-4.077942
C	-9.754000	-0.952053	-3.943363
O	-3.701173	0.416428	-1.078962
O	-3.239858	-1.740213	-1.596547
H	-10.202542	-0.219464	-4.624544
H	-10.234665	-0.816131	-2.966206
H	-10.011089	-1.952661	-4.304111

20

TS(MeBn—CO2−) r^2SCAN-3c SMD 1.85Å

C	-3.577362	-0.586302	-1.742354
C	-3.984360	-0.245088	-3.514478
C	-5.438175	-0.415441	-3.683546
H	-3.399379	-0.969009	-4.085737
H	-3.651557	0.779254	-3.696595
C	-6.005172	-1.670185	-3.963693
C	-6.329771	0.657828	-3.498850
C	-7.705729	0.480836	-3.579211
C	-8.264609	-0.774483	-3.849784
C	-7.384454	-1.843322	-4.041711
H	-8.363535	1.336925	-3.434129
H	-5.928489	1.647558	-3.290735
H	-7.786123	-2.831094	-4.262358
H	-5.349659	-2.524109	-4.121871
C	-9.756713	-0.957874	-3.924722
O	-3.672824	0.411092	-1.019615

O	-3.280644	-1.767368	-1.532647
H	-10.215920	-0.238897	-4.613659
H	-10.228166	-0.806904	-2.945154
H	-10.015035	-1.965113	-4.265919

20

TS(MeBn—CO2−) r²SCAN-3c SMD 2.00Å

C	-3.573809	-0.598317	-1.648507
C	-3.989125	-0.238873	-3.571607
C	-5.431038	-0.409873	-3.721546
H	-3.387645	-0.978228	-4.102136
H	-3.638103	0.783003	-3.723943
C	-6.005672	-1.666107	-3.998712
C	-6.327939	0.661702	-3.523850
C	-7.703550	0.481282	-3.585208
C	-8.266222	-0.774123	-3.851091
C	-7.385015	-1.840134	-4.057987
H	-8.360402	1.336356	-3.428459
H	-5.927023	1.652380	-3.318374
H	-7.787652	-2.828347	-4.276078
H	-5.352040	-2.519812	-4.167632
C	-9.759100	-0.959607	-3.905822
O	-3.708552	0.407353	-0.962171
O	-3.265298	-1.770604	-1.469863
H	-10.229205	-0.246033	-4.593435
H	-10.220264	-0.804552	-2.921786
H	-10.020023	-1.969163	-4.238561

20

TS(MeBn—CO2−) r²SCAN-3c SMD 2.15Å

C	-3.567748	-0.605142	-1.554572
C	-3.993172	-0.235052	-3.629312
C	-5.424287	-0.407323	-3.756045
H	-3.377819	-0.989036	-4.119495
H	-3.626503	0.784085	-3.751449
C	-6.007661	-1.664250	-4.033250
C	-6.325904	0.663090	-3.543525
C	-7.700936	0.480462	-3.588058
C	-8.268413	-0.774112	-3.852409
C	-7.386955	-1.837599	-4.075245
H	-8.355937	1.334888	-3.418658
H	-5.924439	1.653603	-3.337170
H	-7.791057	-2.825579	-4.293049
H	-5.357005	-2.517989	-4.214679
C	-9.761894	-0.960469	-3.889513
O	-3.739256	0.412339	-0.912540
O	-3.247187	-1.768085	-1.405052
H	-10.241650	-0.250984	-4.575165
H	-10.214488	-0.802930	-2.901631
H	-10.025366	-1.971612	-4.215951

20

TS(MeBn—CO2−) r²SCAN-3c SMD 2.30Å

C	-3.558624	-0.610098	-1.459026
C	-3.996112	-0.233515	-3.685411
C	-5.417770	-0.406045	-3.788642
H	-3.369671	-1.000255	-4.138424
H	-3.615000	0.782744	-3.772802
C	-6.011016	-1.663066	-4.068182
C	-6.323951	0.663578	-3.561676
C	-7.698289	0.479267	-3.590382
C	-8.271328	-0.774023	-3.853952
C	-7.390069	-1.834954	-4.093842
H	-8.350922	1.333249	-3.408186
H	-5.922217	1.654054	-3.354502
H	-7.795978	-2.822255	-4.312856
H	-5.364269	-2.517099	-4.263248
C	-9.765274	-0.960690	-3.873914
O	-3.771652	0.418520	-0.866815
O	-3.220044	-1.761801	-1.340750
H	-10.254198	-0.257863	-4.560503
H	-10.210072	-0.796896	-2.883224
H	-10.031224	-1.974546	-4.190431

20

TS(MeBn—CO2−) r²SCAN-3c SMD 2.45Å

C	-3.564934	-0.626765	-1.363708
C	-3.995520	-0.223373	-3.741600
C	-5.408428	-0.399511	-3.822025
H	-3.358221	-0.997367	-4.164820
H	-3.604936	0.791029	-3.791872
C	-6.009512	-1.658802	-4.098906
C	-6.321319	0.668254	-3.583831
C	-7.694398	0.479634	-3.595636
C	-8.270978	-0.774465	-3.853452
C	-7.387865	-1.831835	-4.107003
H	-8.345855	1.332789	-3.403646
H	-5.921234	1.660341	-3.379701
H	-7.793699	-2.820139	-4.323315
H	-5.365266	-2.512571	-4.304448
C	-9.764768	-0.964203	-3.855283
O	-3.782584	0.419746	-0.826050
O	-3.250145	-1.778850	-1.285856
H	-10.264413	-0.274324	-4.547928
H	-10.202584	-0.787781	-2.863330
H	-10.031019	-1.983501	-4.154358

20

TS(MeBn—CO2−) r²SCAN-3c SMD 2.60Å

C	-3.565871	-0.646990	-1.271545
C	-3.995904	-0.210240	-3.798266
C	-5.400222	-0.392104	-3.850482
H	-3.348014	-0.991064	-4.190454
H	-3.596901	0.801478	-3.806764
C	-6.008507	-1.655049	-4.121268
C	-6.320533	0.673109	-3.599801
C	-7.692135	0.479797	-3.597324
C	-8.271638	-0.775160	-3.850810
C	-7.385996	-1.829292	-4.114227
H	-8.342883	1.332013	-3.396864
H	-5.922745	1.667007	-3.398453
H	-7.791217	-2.819054	-4.326913
H	-5.366403	-2.508773	-4.334792
C	-9.765320	-0.966869	-3.842941
O	-3.786401	0.415554	-0.790835
O	-3.272704	-1.797230	-1.251901
H	-10.270429	-0.300290	-4.555037
H	-10.203855	-0.763339	-2.856212
H	-10.030000	-1.995200	-4.111879

20

TS(MeBn—CO2−) r²SCAN-3c SMD 2.75Å

C	-3.610457	-0.680028	-1.190596
C	-3.989765	-0.188278	-3.869552
C	-5.384382	-0.378707	-3.885233
H	-3.328455	-0.980323	-4.211797
H	-3.580475	0.817311	-3.816766
C	-5.998969	-1.647644	-4.143828
C	-6.313265	0.682177	-3.617075
C	-7.682836	0.481451	-3.594366
C	-8.264156	-0.775637	-3.840117
C	-7.375106	-1.825193	-4.115923
H	-8.333207	1.331766	-3.382720
H	-5.919178	1.678713	-3.419958

H	-7.778555	-2.817740	-4.321114
H	-5.358801	-2.500981	-4.365729
C	-9.757444	-0.971252	-3.820993
O	-3.832298	0.397967	-0.773284
O	-3.344133	-1.825313	-1.248004
H	-10.268674	-0.350689	-4.570531
H	-10.199496	-0.714740	-2.848051
H	-10.018029	-2.014555	-4.031130

20

TS(MeBn—CO2−) r^2SCAN-3c SMD 2.90Å

C	-3.962428	-0.750943	-1.167630
C	-3.934429	-0.139493	-4.002299
C	-5.320154	-0.343636	-3.962801
H	-3.259419	-0.949275	-4.266024
H	-3.514098	0.854558	-3.875367
C	-5.934786	-1.623851	-4.186095
C	-6.256490	0.709618	-3.671536
C	-7.620992	0.491795	-3.592408
C	-8.198477	-0.775175	-3.798825
C	-7.305960	-1.815849	-4.100990
H	-8.272303	1.336910	-3.361965
H	-5.868608	1.712808	-3.496351
H	-7.704096	-2.816592	-4.276693
H	-5.294620	-2.474470	-4.418312
C	-9.686836	-0.990315	-3.711135
O	-4.131516	0.350668	-0.813977
O	-3.797880	-1.900772	-1.301252
H	-10.241996	-0.396809	-4.451870
H	-10.092429	-0.718548	-2.726094
H	-9.940162	-2.042323	-3.885144

20

TS(MeBn—CO2−) r^2SCAN-3c SMD 3.05Å

C	-4.050436	-0.763096	-1.109060
C	-3.926975	-0.117431	-4.087377
C	-5.307932	-0.326594	-4.001369
H	-3.256073	-0.929215	-4.356795
H	-3.492126	0.854116	-3.868820
C	-5.927274	-1.598694	-4.268272
C	-6.238726	0.703046	-3.619801
C	-7.600903	0.473484	-3.511226
C	-8.180837	-0.781404	-3.768523
C	-7.292548	-1.801483	-4.152502
H	-8.246465	1.300801	-3.210991
H	-5.849383	1.695571	-3.394516
H	-7.691667	-2.794978	-4.364922
H	-5.290051	-2.434737	-4.555762
C	-9.668746	-1.004784	-3.697963
O	-4.067790	0.360840	-0.798135
O	-4.028787	-1.917686	-1.270813
H	-10.177739	-0.803341	-4.653586
H	-10.137506	-0.354899	-2.948742
H	-9.905714	-2.041212	-3.427591

Geometry scan **TS(FBn--CO2−) r^2SCAN-3c**:

17

TS(FBn—CO2−) r^2SCAN-3c 1.40Å

C	-3.277863	0.238341	-2.064752
C	-3.742114	-0.325900	-3.258948
C	-5.227215	-0.593552	-3.362901
H	-3.209079	-1.256247	-3.491825
H	-3.483118	0.374265	-4.071589
C	-5.700250	-1.887762	-3.588431
C	-6.168068	0.436369	-3.221457
C	-7.532822	0.180816	-3.311569
C	-7.954249	-1.119441	-3.538381
C	-7.065961	-2.167339	-3.677396
H	-8.267424	0.973446	-3.202597
H	-5.794138	1.429713	-2.995557
H	-7.434297	-3.174998	-3.844356
H	-4.985662	-2.701925	-3.687191
F	-9.297028	-1.373944	-3.628522
O	-3.926775	1.229038	-1.612226
O	-2.226553	-0.260221	-1.575798

17

TS(FBn—CO2−) r^2SCAN-3c 1.55Å

C	-3.268919	0.231208	-1.967287
C	-3.752734	-0.322531	-3.331764
C	-5.220836	-0.596457	-3.420110
H	-3.183716	-1.223677	-3.581900
H	-3.483504	0.450091	-4.066364
C	-5.704802	-1.896665	-3.595100
C	-6.159582	0.438531	-3.285413
C	-7.526325	0.185193	-3.337026
C	-7.955879	-1.119380	-3.517856
C	-7.072059	-2.173322	-3.645511
H	-8.256014	0.982702	-3.231096
H	-5.788794	1.440521	-3.097746
H	-7.446396	-3.183989	-3.777879
H	-4.994791	-2.715621	-3.686276
F	-9.301358	-1.372560	-3.573562
O	-3.968230	1.152795	-1.481147
O	-2.208676	-0.276181	-1.537460

17

TS(FBn—CO2−) r^2SCAN-3c 1.70Å

C	-3.259780	0.213345	-1.870365
C	-3.763163	-0.312687	-3.406560
C	-5.214853	-0.592644	-3.476201
H	-3.163259	-1.186005	-3.674021
H	-3.489785	0.526289	-4.057110
C	-5.705430	-1.899942	-3.600057
C	-6.156661	0.443395	-3.349113
C	-7.523559	0.188332	-3.362311
C	-7.956133	-1.120569	-3.496449
C	-7.071956	-2.176822	-3.611700
H	-8.250941	0.988560	-3.260759
H	-5.791394	1.454160	-3.200865
H	-7.447839	-3.191151	-3.708004
H	-4.997295	-2.721357	-3.682800
F	-9.303049	-1.376876	-3.517495
O	-3.986363	1.091068	-1.375490
O	-2.211156	-0.326438	-1.484198

17

TS(FBn—CO2−) r^2SCAN-3c 1.85Å

C	-3.243771	0.191765	-1.775246
C	-3.774854	-0.300978	-3.477496
C	-5.211013	-0.585054	-3.524660
H	-3.149484	-1.150814	-3.755086
H	-3.497715	0.591185	-4.045894
C	-5.705342	-1.899231	-3.602716
C	-6.160133	0.449622	-3.408205
C	-7.525603	0.190303	-3.387737
C	-7.957605	-1.122380	-3.478098
C	-7.069659	-2.178012	-3.580587
H	-8.252807	0.991724	-3.293327
H	-5.801740	1.468156	-3.298121
H	-7.443436	-3.195953	-3.643442
H	-4.997341	-2.721622	-3.676527
F	-9.305068	-1.383921	-3.468100

O	-3.989033	1.036613	-1.280728
O	-2.208013	-0.380745	-1.437528

17
TS(FBn—CO2−) r^{2}SCAN-3c 2.00Å

C	-3.215880	0.174159	-1.681888
C	-3.788014	-0.293006	-3.540495
C	-5.210203	-0.578374	-3.563298
H	-3.142250	-1.125523	-3.818006
H	-3.504697	0.637647	-4.035210
C	-5.708301	-1.897948	-3.603616
C	-6.167720	0.454912	-3.459966
C	-7.531287	0.192186	-3.413554
C	-7.962366	-1.122926	-3.466061
C	-7.070286	-2.177407	-3.555811
H	-8.258773	0.994640	-3.328703
H	-5.815813	1.479396	-3.384661
H	-7.441310	-3.197944	-3.590207
H	-5.000608	-2.721466	-3.668085
F	-9.310349	-1.388420	-3.430971
O	-3.993470	0.968576	-1.180679
O	-2.171287	-0.397841	-1.412285

17
TS(FBn—CO2−) r^{2}SCAN-3c 2.15Å

C	-3.185962	0.163863	-1.591543
C	-3.800265	-0.287265	-3.601920
C	-5.210192	-0.572845	-3.596913
H	-3.138093	-1.108308	-3.869550
H	-3.509884	0.670308	-4.033138
C	-5.711488	-1.896905	-3.607955
C	-6.176040	0.458841	-3.502357
C	-7.537410	0.192631	-3.433285
C	-7.967313	-1.123989	-3.455515
C	-7.071095	-2.176705	-3.537844
H	-8.265360	0.995607	-3.355122
H	-5.830339	1.487597	-3.455220
H	-7.439083	-3.199095	-3.549753
H	-5.003914	-2.721020	-3.666981
F	-9.315695	-1.392999	-3.397622
O	-3.989113	0.923985	-1.100813
O	-2.141370	-0.413039	-1.377967

17
TS(FBn—CO2−) r^{2}SCAN-3c 2.30Å

C	-3.150772	0.161422	-1.502626
C	-3.812569	-0.285685	-3.659504
C	-5.211782	-0.569732	-3.626639
H	-3.136658	-1.100130	-3.908132
H	-3.512392	0.690263	-4.035980
C	-5.717143	-1.897225	-3.615276
C	-6.184919	0.461155	-3.537292
C	-7.544158	0.192695	-3.449518
C	-7.973906	-1.124565	-3.448159
C	-7.074681	-2.176060	-3.526530
H	-8.272148	0.996336	-3.375739
H	-5.844047	1.492729	-3.512307
H	-7.440537	-3.199519	-3.521032
H	-5.010395	-2.722354	-3.671694
F	-9.322887	-1.395482	-3.370292
O	-3.985119	0.880912	-1.023635
O	-2.098503	-0.404102	-1.349139

17
TS(FBn—CO2−) r^{2}SCAN-3c 2.45Å

C	-3.114435	0.168168	-1.414362
C	-3.823724	-0.285912	-3.715061
C	-5.214330	-0.568855	-3.654596
H	-3.138113	-1.096148	-3.947157
H	-3.516272	0.703051	-4.046172
C	-5.722789	-1.899104	-3.626962
C	-6.193250	0.461564	-3.564600
C	-7.550365	0.191119	-3.459654
C	-7.979997	-1.126611	-3.441533
C	-7.078546	-2.176987	-3.521139
H	-8.278202	0.995192	-3.386115
H	-5.855999	1.494916	-3.555461
H	-7.442372	-3.201248	-3.503129
H	-5.017058	-2.725117	-3.684639
F	-9.329365	-1.398826	-3.345158
O	-3.966208	0.874440	-0.965582
O	-2.071591	-0.408984	-1.302176

17
TS(FBn—CO2−) r^{2}SCAN-3c 2.60Å

C	-3.076597	0.176387	-1.324925
C	-3.834850	-0.286908	-3.768368
C	-5.217445	-0.568713	-3.681887
H	-3.139958	-1.093994	-3.980367
H	-3.519366	0.712068	-4.056888
C	-5.729399	-1.901311	-3.640039
C	-6.202019	0.461268	-3.590115
C	-7.556909	0.189310	-3.469218
C	-7.986767	-1.128668	-3.436357
C	-7.083334	-2.177991	-3.518338
H	-8.284448	0.993911	-3.395351
H	-5.868126	1.496073	-3.595371
H	-7.445416	-3.202860	-3.489612
H	-5.024797	-2.728285	-3.700119
F	-9.336383	-1.401765	-3.322342
O	-3.943931	0.872513	-0.908859
O	-2.042868	-0.410376	-1.255341

17
TS(FBn—CO2−) r^{2}SCAN-3c 2.75Å

C	-3.045427	0.188926	-1.234756
C	-3.843690	-0.289733	-3.822450
C	-5.219433	-0.569654	-3.710476
H	-3.142919	-1.095376	-4.018645
H	-3.521433	0.716216	-4.075911
C	-5.734774	-1.904122	-3.656375
C	-6.208136	0.460400	-3.612346
C	-7.560725	0.187534	-3.473948
C	-7.991125	-1.130470	-3.428870
C	-7.086890	-2.179220	-3.517199
H	-8.287422	0.992822	-3.396393
H	-5.877061	1.496258	-3.629529
H	-7.447667	-3.204478	-3.479520
H	-5.032006	-2.732397	-3.721851
F	-9.340795	-1.403682	-3.295730
O	-3.927059	0.881212	-0.861613
O	-2.026053	-0.413580	-1.197885

17
TS(FBn—CO2−) r^{2}SCAN-3c 2.90Å

C	-3.161750	0.091563	-1.146429
C	-3.839541	-0.205337	-3.950435
C	-5.196610	-0.519538	-3.772525
H	-3.118479	-0.993298	-4.142941
H	-3.543857	0.813819	-4.179468
C	-5.673164	-1.868695	-3.659310
C	-6.217743	0.483583	-3.669867
C	-7.553354	0.174356	-3.463585
C	-7.942175	-1.153679	-3.355620

C	-7.009130	-2.176793	-3.452670
H	-8.300982	0.960555	-3.385116
H	-5.922582	1.528271	-3.742047
H	-7.335457	-3.211017	-3.368107
H	-4.949841	-2.678786	-3.728062
F	-9.278042	-1.461863	-3.154315
O	-3.642607	1.151467	-0.978916
O	-2.607301	-0.933950	-0.984083

17

TS(FBn—CO2−) r^2SCAN-3c 3.05Å

C	-3.360392	0.007793	-1.075716
C	-3.819237	-0.167207	-4.085921
C	-5.158482	-0.491968	-3.836803
H	-3.086739	-0.948834	-4.262055
H	-3.532138	0.857057	-4.302589
C	-5.615627	-1.845288	-3.666017
C	-6.192215	0.500936	-3.712099
C	-7.511819	0.180133	-3.435102
C	-7.878677	-1.149374	-3.273610
C	-6.936226	-2.162395	-3.388795
H	-8.265381	0.959486	-3.343123
H	-5.917490	1.547639	-3.827382
H	-7.243588	-3.198253	-3.261332
H	-4.886308	-2.649083	-3.745629
F	-9.201386	-1.468603	-3.001164
O	-3.637755	1.141059	-0.978571
O	-3.049155	-1.112439	-0.937587

17

TS(FBn—CO2−) r^2SCAN-3c SMD 1.40Å

C	-3.276383	0.066071	-2.016605
C	-3.751400	-0.252269	-3.294501
C	-5.231282	-0.544168	-3.396239
H	-3.199665	-1.097175	-3.723144
H	-3.532200	0.606422	-3.950382
C	-5.681917	-1.861499	-3.507413
C	-6.183037	0.481094	-3.349750
C	-7.545689	0.205315	-3.413231
C	-7.943395	-1.117791	-3.515489
C	-7.043294	-2.164736	-3.564909
H	-8.288741	0.997030	-3.383823
H	-5.847995	1.509104	-3.255310
H	-7.396040	-3.188314	-3.649168
H	-4.958197	-2.672636	-3.546551
F	-9.279141	-1.396205	-3.576993
O	-3.984418	0.839985	-1.305884
O	-2.149823	-0.409569	-1.684104

17

TS(FBn—CO2−) r^2SCAN-3c SMD 1.55Å

C	-3.239662	0.056713	-1.921657
C	-3.766916	-0.264743	-3.343335
C	-5.233266	-0.553843	-3.437954
H	-3.185687	-1.101916	-3.744629
H	-3.519285	0.612373	-3.957376
C	-5.703565	-1.869039	-3.501452
C	-6.175697	0.481598	-3.414235
C	-7.542373	0.222131	-3.454433
C	-7.956614	-1.097927	-3.512480
C	-7.068019	-2.156415	-3.537349
H	-8.275171	1.023732	-3.439569
H	-5.832441	1.511162	-3.359063
H	-7.434056	-3.177725	-3.586227
H	-4.990924	-2.690745	-3.518843
F	-9.296343	-1.363258	-3.551856
O	-4.078915	0.416765	-1.060778
O	-1.993681	-0.048205	-1.792261

17

TS(FBn—CO2−) r^2SCAN-3c SMD 1.70Å

C	-3.221809	0.074489	-1.837891
C	-3.776651	-0.271378	-3.407135
C	-5.230819	-0.558751	-3.484010
H	-3.173730	-1.107739	-3.771402
H	-3.512623	0.624523	-3.981188
C	-5.708854	-1.874920	-3.528632
C	-6.174572	0.477871	-3.442466
C	-7.542122	0.220244	-3.452036
C	-7.960751	-1.098586	-3.497113
C	-7.073506	-2.158652	-3.535279
H	-8.272501	1.023800	-3.423966
H	-5.829697	1.507573	-3.398679
H	-7.442087	-3.179646	-3.572202
H	-4.999122	-2.698883	-3.556269
F	-9.302157	-1.362266	-3.508664
O	-4.085555	0.425677	-1.014404
O	-1.986060	-0.042698	-1.722161

17

TS(FBn—CO2−) r^2SCAN-3c SMD 1.85Å

C	-3.196163	0.056727	-1.748215
C	-3.789237	-0.263206	-3.471121
C	-5.230371	-0.551593	-3.529786
H	-3.165807	-1.087098	-3.824933
H	-3.515295	0.664519	-3.982073
C	-5.711508	-1.871346	-3.536921
C	-6.180685	0.482987	-3.493157
C	-7.547428	0.221978	-3.471944
C	-7.965111	-1.097586	-3.482631
C	-7.074502	-2.156084	-3.513976
H	-8.278282	1.025338	-3.446536
H	-5.839703	1.515193	-3.476931
H	-7.441079	-3.178575	-3.522771
H	-5.001604	-2.695420	-3.558313
F	-9.306920	-1.364858	-3.464864
O	-4.083605	0.331201	-0.936770
O	-1.965316	-0.031519	-1.672554

17

TS(FBn—CO2−) r^2SCAN-3c SMD 2.00Å

C	-3.163023	0.051875	-1.661051
C	-3.802519	-0.260532	-3.530127
C	-5.231021	-0.548061	-3.565577
H	-3.162170	-1.077559	-3.865004
H	-3.518585	0.689732	-3.987439
C	-5.716408	-1.870744	-3.547787
C	-6.188548	0.485124	-3.529448
C	-7.553867	0.221508	-3.485488
C	-7.971419	-1.098228	-3.473627
C	-7.077804	-2.155185	-3.502871
H	-8.284882	1.024927	-3.460374
H	-5.850836	1.518843	-3.531486
H	-7.442313	-3.178585	-3.493292
H	-5.006936	-2.695334	-3.567558
F	-9.313923	-1.367684	-3.434215
O	-4.068504	0.287410	-0.872657
O	-1.939859	-0.026848	-1.625494

17

TS(FBn—CO2−) r^2SCAN-3c SMD 2.15Å

C	-3.132963	0.048751	-1.574831
C	-3.813697	-0.259987	-3.590713
C	-5.230399	-0.545658	-3.599532

H	-3.158491	-1.072721	-3.902482
H	-3.519828	0.707944	-3.998131
C	-5.720867	-1.870851	-3.559662
C	-6.194605	0.487059	-3.561964
C	-7.558217	0.221804	-3.495916
C	-7.976631	-1.097669	-3.463610
C	-7.080740	-2.153601	-3.492965
H	-8.288797	1.025838	-3.469914
H	-5.860037	1.521876	-3.580751
H	-7.443677	-3.177479	-3.466551
H	-5.012538	-2.696535	-3.579303
F	-9.320149	-1.367983	-3.402509
O	-4.063259	0.236336	-0.817763
O	-1.917719	-0.006467	-1.576898

17

TS(FBn—CO2−) r²SCAN-3c SMD 2.30Å

C	-3.098170	0.046288	-1.487762
C	-3.824268	-0.262399	-3.648200
C	-5.230550	-0.544749	-3.630688
H	-3.156450	-1.074936	-3.928995
H	-3.518943	0.716926	-4.014232
C	-5.728014	-1.871371	-3.568256
C	-6.200432	0.489064	-3.595995
C	-7.562347	0.224530	-3.510053
C	-7.983535	-1.093984	-3.455623
C	-7.086879	-2.150047	-3.482025
H	-8.291440	1.030241	-3.485616
H	-5.867561	1.524236	-3.631959
H	-7.449557	-3.173647	-3.437964
H	-5.022138	-2.699329	-3.585451
F	-9.328534	-1.362548	-3.373780
O	-4.051019	0.116740	-0.753459
O	-1.892779	0.085643	-1.543438

17

TS(FBn—CO2−) r²SCAN-3c SMD 2.45Å

C	-3.062958	0.057244	-1.400633
C	-3.834150	-0.268159	-3.703213
C	-5.230683	-0.546332	-3.659859
H	-3.154099	-1.080798	-3.949147
H	-3.515906	0.721477	-4.024950
C	-5.735459	-1.875007	-3.585801
C	-6.206601	0.488503	-3.619342
C	-7.566506	0.223881	-3.514968
C	-7.990489	-1.093907	-3.448342
C	-7.093053	-2.149959	-3.481327
H	-8.294043	1.031151	-3.485594
H	-5.875734	1.524124	-3.664815
H	-7.455157	-3.173580	-3.427961
H	-5.032320	-2.705346	-3.608140
F	-9.336973	-1.361180	-3.346647
O	-4.037672	0.090463	-0.708453
O	-1.870812	0.118081	-1.504303

17

TS(FBn—CO2−) r²SCAN-3c SMD 2.60Å

C	-3.000309	0.067145	-1.310365
C	-3.849394	-0.269576	-3.744635
C	-5.236704	-0.545930	-3.681716
H	-3.155978	-1.080898	-3.951883
H	-3.519462	0.729570	-4.019569
C	-5.747095	-1.877528	-3.597364
C	-6.220971	0.488353	-3.642396
C	-7.579043	0.221420	-3.528896
C	-8.004050	-1.096251	-3.451801
C	-7.103659	-2.150763	-3.483960
H	-8.306477	1.029185	-3.500902
H	-5.893804	1.524904	-3.697356
H	-7.463679	-3.174962	-3.422664
H	-5.045304	-2.709226	-3.619347
F	-9.352363	-1.364442	-3.340078
O	-3.986947	0.070446	-0.650765
O	-1.827373	0.139210	-1.489798

17

TS(FBn—CO2−) r²SCAN-3c SMD 2.75Å

C	-2.891876	0.096162	-1.221021
C	-3.872536	-0.275688	-3.763172
C	-5.251633	-0.550143	-3.691953
H	-3.163959	-1.085184	-3.917073
H	-3.528331	0.733081	-3.976877
C	-5.769187	-1.885006	-3.608599
C	-6.243857	0.484479	-3.656222
C	-7.601484	0.215354	-3.549572
C	-8.029184	-1.102368	-3.474608
C	-7.126279	-2.155868	-3.502253
H	-8.328466	1.024030	-3.525460
H	-5.919220	1.522021	-3.711471
H	-7.485720	-3.180673	-3.442624
H	-5.069303	-2.718561	-3.628317
F	-9.381032	-1.371269	-3.369630
O	-3.878121	0.089171	-0.577452
O	-1.752428	0.161120	-1.517192

17

TS(FBn—CO2−) r²SCAN-3c SMD 2.90Å

C	-2.785478	0.102653	-1.141817
C	-3.895930	-0.271416	-3.794545
C	-5.267298	-0.547199	-3.707327
H	-3.170355	-1.076575	-3.869050
H	-3.540270	0.746860	-3.927074
C	-5.786680	-1.885505	-3.611799
C	-6.269019	0.485116	-3.680150
C	-7.625429	0.210868	-3.577067
C	-8.051492	-1.107591	-3.494375
C	-7.143330	-2.157701	-3.509352
H	-8.354464	1.018223	-3.561231
H	-5.949523	1.523952	-3.743670
H	-7.499151	-3.183495	-3.441122
H	-5.085769	-2.718562	-3.621816
F	-9.405508	-1.380318	-3.392444
O	-3.776838	0.065387	-0.520727
O	-1.686079	0.175960	-1.539928

17

TS(FBn—CO2−) r²SCAN-3c SMD 3.05Å

C	-2.731638	0.113879	-1.063026
C	-3.908006	-0.273550	-3.850239
C	-5.275249	-0.548735	-3.735896
H	-3.175653	-1.075792	-3.873319
H	-3.547678	0.749047	-3.923992
C	-5.795753	-1.887907	-3.629082
C	-6.279739	0.483456	-3.701052
C	-7.634687	0.208514	-3.584313
C	-8.060535	-1.109809	-3.493331
C	-7.151319	-2.159271	-3.512844
H	-8.363475	1.016128	-3.563517
H	-5.962089	1.522484	-3.771635
H	-7.505671	-3.185070	-3.435674
H	-5.095364	-2.721459	-3.641914
F	-9.414510	-1.382648	-3.376159
O	-3.741712	0.071714	-0.480186
O	-1.649535	0.179677	-1.497317

Geometry scan **TS(Bn--CO2−) r^{2}SCAN-3c**:

17

TS(Bn—CO2−) r^{2}SCAN-3c 1.40Å

C	-3.106354	0.720992	-2.639780
C	-4.220843	1.053218	-3.419229
C	-5.374718	0.075195	-3.386248
H	-3.889074	1.119009	-4.469046
H	-4.610180	2.045160	-3.157723
C	-5.210318	-1.264356	-3.766567
C	-6.640756	0.485859	-2.958330
C	-7.715326	-0.401487	-2.914227
C	-7.541638	-1.726601	-3.300954
C	-6.280925	-2.150011	-3.724730
H	-8.688365	-0.055547	-2.571724
H	-6.780433	1.518895	-2.646169
H	-6.131327	-3.186168	-4.021849
H	-4.211760	-1.583243	-4.050000
H	-8.375661	-2.423924	-3.268123
O	-2.719621	1.581051	-1.800083
O	-2.569413	-0.403616	-2.874029

17

TS(Bn—CO2−) r^{2}SCAN-3c 1.55Å

C	-3.084982	0.718043	-2.526954
C	-4.246936	1.056556	-3.495341
C	-5.389847	0.094279	-3.442216
H	-3.806090	1.038075	-4.501897
H	-4.601830	2.073611	-3.300305
C	-5.233618	-1.240504	-3.848342
C	-6.639952	0.482905	-2.945645
C	-7.700837	-0.416589	-2.864111
C	-7.534461	-1.734566	-3.280457
C	-6.290996	-2.138437	-3.769611
H	-8.660483	-0.085585	-2.472304
H	-6.774680	1.509791	-2.611759
H	-6.144593	-3.168793	-4.087691
H	-4.251834	-1.556205	-4.186609
H	-8.359628	-2.440284	-3.220474
O	-2.727112	1.653234	-1.775523
O	-2.618836	-0.441106	-2.639571

17

TS(Bn—CO2−) r^{2}SCAN-3c 1.70Å

C	-3.052287	0.711855	-2.432415
C	-4.275483	1.063107	-3.559541
C	-5.404142	0.110935	-3.488743
H	-3.756562	0.991011	-4.522142
H	-4.596905	2.093709	-3.390729
C	-5.254285	-1.223554	-3.906368
C	-6.643840	0.481996	-2.944988
C	-7.691058	-0.427684	-2.833530
C	-7.529581	-1.743093	-3.263964
C	-6.299107	-2.131696	-3.796708
H	-8.640136	-0.107086	-2.408231
H	-6.777505	1.505456	-2.600119
H	-6.152860	-3.159100	-4.124581
H	-4.285777	-1.535548	-4.284652
H	-8.346092	-2.456172	-3.180718
O	-2.713832	1.690992	-1.748518
O	-2.647263	-0.460704	-2.482863

17

TS(Bn—CO2−) r^{2}SCAN-3c 1.85Å

C	-3.026627	0.709587	-2.336660
C	-4.298016	1.065812	-3.632490
C	-5.411956	0.122703	-3.536419
H	-3.710577	0.940722	-4.545903
H	-4.590532	2.106495	-3.485110
C	-5.275678	-1.214205	-3.962118
C	-6.639706	0.483040	-2.948371
C	-7.675384	-0.432312	-2.803963
C	-7.525125	-1.747968	-3.242290
C	-6.310577	-2.127467	-3.817461
H	-8.611198	-0.116471	-2.346308
H	-6.767924	1.504787	-2.596340
H	-6.169204	-3.153381	-4.152720
H	-4.322601	-1.525383	-4.379819
H	-8.334842	-2.464837	-3.132381
O	-2.775187	1.703393	-1.655690
O	-2.621579	-0.450091	-2.394764

17

TS(Bn—CO2−) r^{2}SCAN-3c 2.00Å

C	-2.982928	0.710924	-2.251261
C	-4.325806	1.070612	-3.689073
C	-5.421704	0.131108	-3.570539
H	-3.685200	0.913720	-4.558357
H	-4.592113	2.116746	-3.541667
C	-5.295230	-1.208494	-4.003423
C	-6.642965	0.480822	-2.951927
C	-7.668392	-0.439863	-2.786433
C	-7.525216	-1.755772	-3.230828
C	-6.321585	-2.126009	-3.835840
H	-8.594739	-0.128318	-2.306454
H	-6.769817	1.500865	-2.594162
H	-6.182711	-3.150186	-4.178159
H	-4.354088	-1.517504	-4.450097
H	-8.328594	-2.476530	-3.103177
O	-2.756449	1.734009	-1.624918
O	-2.619178	-0.451704	-2.292494

17

TS(Bn—CO2−) r^{2}SCAN-3c 2.15Å

C	-2.939152	0.711210	-2.168663
C	-4.351758	1.075022	-3.748120
C	-5.429830	0.137700	-3.603406
H	-3.665673	0.891364	-4.574190
H	-4.593584	2.124548	-3.592826
C	-5.313764	-1.206095	-4.039775
C	-6.645198	0.480581	-2.958911
C	-7.661427	-0.443808	-2.771960
C	-7.525760	-1.761636	-3.217475
C	-6.332835	-2.125734	-3.848848
H	-8.578468	-0.134052	-2.272683
H	-6.769941	1.500175	-2.598832
H	-6.197279	-3.149229	-4.195391
H	-4.384519	-1.514954	-4.511922
H	-8.323477	-2.484943	-3.071538
O	-2.769419	1.747794	-1.564446
O	-2.584630	-0.443519	-2.229822

17

TS(Bn—CO2−) r^{2}SCAN-3c 2.30Å

C	-2.888415	0.706495	-2.091558
C	-4.380678	1.084458	-3.800445
C	-5.440303	0.146104	-3.631751
H	-3.657893	0.884782	-4.589094
H	-4.601035	2.134891	-3.626964
C	-5.329245	-1.202481	-4.068332
C	-6.653116	0.480746	-2.968512
C	-7.658994	-0.449123	-2.763787
C	-7.525451	-1.769362	-3.206894

C	-6.339689	-2.125832	-3.858009
H	-8.569741	-0.142289	-2.250713
H	-6.780157	1.500498	-2.609175
H	-6.203687	-3.149134	-4.205871
H	-4.408586	-1.509463	-4.559121
H	-8.316217	-2.496740	-3.045447
O	-2.734997	1.762706	-1.534935
O	-2.578512	-0.451833	-2.158201

17

TS(Bn—CO2−) r^2SCAN-3c 2.45Å

C	-2.829433	0.702149	-2.017867
C	-4.409679	1.093915	-3.848671
C	-5.452027	0.153268	-3.656826
H	-3.656663	0.882105	-4.603909
H	-4.612081	2.143949	-3.656778
C	-5.344036	-1.200441	-4.091042
C	-6.663317	0.480886	-2.978786
C	-7.660091	-0.453731	-2.759347
C	-7.527623	-1.776582	-3.198825
C	-6.346939	-2.126781	-3.864729
H	-8.565830	-0.148741	-2.235789
H	-6.793286	1.501071	-2.621190
H	-6.209542	-3.150201	-4.212602
H	-4.430034	-1.506098	-4.595782
H	-8.312265	-2.507406	-3.024620
O	-2.698308	1.773525	-1.502037
O	-2.555560	-0.456465	-2.100011

17

TS(Bn—CO2−) r^2SCAN-3c 2.60Å

C	-2.789821	0.686074	-1.939654
C	-4.432475	1.102452	-3.911535
C	-5.459106	0.160971	-3.688388
H	-3.654081	0.874240	-4.634718
H	-4.618790	2.152942	-3.708127
C	-5.356506	-1.199769	-4.113857
C	-6.665469	0.487576	-2.993567
C	-7.653372	-0.449489	-2.750862
C	-7.524382	-1.777188	-3.180106
C	-6.352096	-2.126611	-3.863238
H	-8.551881	-0.142825	-2.215416
H	-6.795625	1.510143	-2.642552
H	-6.216424	-3.152104	-4.206519
H	-4.451666	-1.507445	-4.634346
H	-8.303251	-2.509366	-2.987261
O	-2.773376	1.737854	-1.384489
O	-2.468393	-0.443032	-2.114175

17

TS(Bn—CO2−) r^2SCAN-3c 2.75Å

C	-2.736929	0.673824	-1.862240
C	-4.458287	1.112389	-3.961546
C	-5.470162	0.168808	-3.715679
H	-3.659569	0.875193	-4.658762
H	-4.631646	2.161622	-3.741930
C	-5.369191	-1.197666	-4.134543
C	-6.673772	0.490829	-3.007238
C	-7.652376	-0.450353	-2.747199
C	-7.523252	-1.781523	-3.168712
C	-6.356695	-2.127009	-3.865589
H	-8.545836	-0.144158	-2.202566
H	-6.807257	1.514899	-2.661348
H	-6.219564	-3.153599	-4.205859
H	-4.471393	-1.505271	-4.667793
H	-8.295524	-2.516568	-2.961291
O	-2.768297	1.726836	-1.323670
O	-2.426965	-0.443829	-2.082845

17

TS(Bn—CO2−) r^2SCAN-3c 2.90Å

C	-2.850327	0.600620	-1.746900
C	-4.453121	1.117428	-4.107824
C	-5.447015	0.183055	-3.789708
H	-3.649821	0.851595	-4.788571
H	-4.599063	2.170104	-3.884411
C	-5.377303	-1.194667	-4.191320
C	-6.613670	0.518574	-3.020843
C	-7.576436	-0.415937	-2.690492
C	-7.475838	-1.756245	-3.093831
C	-6.350267	-2.114959	-3.851120
H	-8.436524	-0.096994	-2.100772
H	-6.725581	1.547813	-2.682687
H	-6.235495	-3.147090	-4.183930
H	-4.515022	-1.514899	-4.774468
H	-8.236879	-2.485049	-2.830866
O	-3.325329	1.414066	-1.043973
O	-2.199021	-0.272990	-2.187095

17

TS(Bn—CO2−) r^2SCAN-3c 3.05Å

C	-2.994555	0.455169	-1.661104
C	-4.478859	1.170841	-4.227650
C	-5.435926	0.226037	-3.849958
H	-3.671020	0.900896	-4.901333
H	-4.614429	2.221757	-3.989864
C	-5.354645	-1.162398	-4.222695
C	-6.581680	0.552101	-3.040965
C	-7.508134	-0.395256	-2.650967
C	-7.394960	-1.743683	-3.026012
C	-6.292445	-2.093933	-3.821895
H	-8.350499	-0.080284	-2.033703
H	-6.704483	1.586204	-2.721373
H	-6.167482	-3.130921	-4.136313
H	-4.510780	-1.479523	-4.834169
H	-8.128063	-2.482268	-2.715613
O	-3.524799	1.259418	-0.997178
O	-2.353957	-0.399734	-2.138017

17

TS(Bn—CO2−) r^2SCAN-3c SMD 1.40Å

C	-3.304546	0.832219	-2.453521
C	-4.120649	0.962512	-3.583565
C	-5.322654	0.043799	-3.492666
H	-3.572554	0.708831	-4.499828
H	-4.491438	1.989847	-3.689354
C	-5.260066	-1.267195	-3.976758
C	-6.503837	0.471017	-2.876810
C	-7.593840	-0.387162	-2.749306
C	-7.520977	-1.691179	-3.236128
C	-6.348600	-2.127519	-3.850950
H	-8.504828	-0.034914	-2.271330
H	-6.564665	1.487257	-2.495259
H	-6.282310	-3.141499	-4.238115
H	-4.347659	-1.612044	-4.456876
H	-8.372172	-2.360399	-3.141342
O	-3.559066	1.583131	-1.464033
O	-2.396853	-0.052279	-2.492970

17

TS(Bn—CO2−) r^2SCAN-3c SMD 1.55Å

C	-3.252583	0.814378	-2.379879
C	-4.153637	0.972341	-3.631138
C	-5.338532	0.057289	-3.527947

H	-3.571800	0.724515	-4.525352
H	-4.494717	2.010786	-3.699727
C	-5.273597	-1.261694	-3.996981
C	-6.520873	0.477741	-2.904146
C	-7.599810	-0.389460	-2.751959
C	-7.521052	-1.699556	-3.222121
C	-6.351156	-2.130707	-3.846047
H	-8.508459	-0.039375	-2.267732
H	-6.590665	1.498617	-2.536050
H	-6.278880	-3.148762	-4.221599
H	-4.365274	-1.605598	-4.485959
H	-8.364181	-2.375873	-3.108268
O	-3.526941	1.561029	-1.407102
O	-2.354556	-0.061247	-2.456803

17

TS(Bn—CO2−) r²SCAN-3c SMD 1.70Å

C	-3.206016	0.799678	-2.303518
C	-4.185170	0.981790	-3.681231
C	-5.352843	0.070060	-3.563811
H	-3.568974	0.732487	-4.549507
H	-4.498279	2.028752	-3.719539
C	-5.287228	-1.255851	-4.021786
C	-6.534656	0.483136	-2.927651
C	-7.601914	-0.392608	-2.750753
C	-7.519305	-1.707550	-3.208084
C	-6.353745	-2.132479	-3.845499
H	-8.506573	-0.045316	-2.256795
H	-6.611211	1.507110	-2.568975
H	-6.276949	-3.153481	-4.212501
H	-4.384086	-1.598026	-4.521996
H	-8.354073	-2.390494	-3.074654
O	-3.497591	1.555318	-1.357221
O	-2.328102	-0.078101	-2.405290

17

TS(Bn—CO2−) r²SCAN-3c SMD 1.85Å

C	-3.163374	0.787851	-2.225130
C	-4.215599	0.990801	-3.733153
C	-5.364713	0.081690	-3.598403
H	-3.565612	0.736400	-4.572981
H	-4.502635	2.044286	-3.744386
C	-5.300447	-1.250603	-4.048680
C	-6.545399	0.487189	-2.947390
C	-7.600967	-0.396116	-2.747242
C	-7.516454	-1.715138	-3.194880
C	-6.356366	-2.133432	-3.848083
H	-8.500574	-0.051623	-2.241783
H	-6.626734	1.513135	-2.594649
H	-6.276355	-3.156459	-4.209314
H	-4.403526	-1.590851	-4.561757
H	-8.343076	-2.403879	-3.042671
O	-3.469452	1.562998	-1.314106
O	-2.315431	-0.101825	-2.344202

17

TS(Bn—CO2−) r²SCAN-3c SMD 2.00Å

C	-3.120208	0.774777	-2.148682
C	-4.245775	1.000282	-3.786439
C	-5.374859	0.092771	-3.629954
H	-3.565387	0.741214	-4.598623
H	-4.508391	2.058725	-3.768680
C	-5.312045	-1.246991	-4.071175
C	-6.555580	0.491810	-2.965953
C	-7.600096	-0.397804	-2.744839
C	-7.514109	-1.721309	-3.182125
C	-6.358304	-2.134284	-3.848305
H	-8.495267	-0.054864	-2.230005
H	-6.641217	1.519723	-2.619237
H	-6.275095	-3.159510	-4.203267
H	-4.420548	-1.586517	-4.594661
H	-8.333382	-2.414699	-3.013068
O	-3.454865	1.555263	-1.266955
O	-2.291586	-0.114162	-2.296843

17

TS(Bn—CO2−) r²SCAN-3c SMD 2.15Å

C	-3.079273	0.756322	-2.073722
C	-4.276181	1.012822	-3.841240
C	-5.384264	0.105411	-3.660401
H	-3.567647	0.749490	-4.625865
H	-4.515444	2.074565	-3.791458
C	-5.321468	-1.242770	-4.090704
C	-6.566253	0.498003	-2.985052
C	-7.599040	-0.398100	-2.743552
C	-7.510252	-1.726669	-3.168703
C	-6.357496	-2.134378	-3.845950
H	-8.490408	-0.056523	-2.220679
H	-6.656771	1.527947	-2.644865
H	-6.269597	-3.162239	-4.192949
H	-4.433774	-1.581906	-4.621431
H	-8.321657	-2.424838	-2.983010
O	-3.445410	1.538466	-1.220336
O	-2.271779	-0.131176	-2.258893

17

TS(Bn—CO2−) r²SCAN-3c SMD 2.30Å

C	-3.033859	0.739677	-1.998695
C	-4.307642	1.027031	-3.892079
C	-5.394249	0.117783	-3.687422
H	-3.570873	0.759933	-4.647470
H	-4.523865	2.090380	-3.806850
C	-5.330308	-1.238327	-4.109074
C	-6.579295	0.502331	-3.002211
C	-7.599893	-0.401028	-2.743485
C	-7.506645	-1.734141	-3.157868
C	-6.355920	-2.134772	-3.845483
H	-8.488505	-0.061763	-2.213826
H	-6.677601	1.533932	-2.668400
H	-6.262272	-3.164449	-4.186521
H	-4.446494	-1.576196	-4.647471
H	-8.309808	-2.437654	-2.957781
O	-3.403153	1.553265	-1.191028
O	-2.276332	-0.171579	-2.213147

17

TS(Bn—CO2−) r²SCAN-3c SMD 2.45Å

C	-2.996992	0.720687	-1.918253
C	-4.334239	1.039262	-3.946252
C	-5.401152	0.129421	-3.716416
H	-3.571947	0.767282	-4.673037
H	-4.527522	2.103288	-3.829098
C	-5.339371	-1.234864	-4.129874
C	-6.586939	0.507161	-3.017737
C	-7.595949	-0.401692	-2.740093
C	-7.501699	-1.739335	-3.144799
C	-6.355286	-2.134238	-3.845927
H	-8.479720	-0.063608	-2.200956
H	-6.690290	1.540133	-2.688853
H	-6.258396	-3.165624	-4.181962
H	-4.461290	-1.572875	-4.678049
H	-8.297091	-2.446778	-2.928901
O	-3.398674	1.548847	-1.156649
O	-2.270159	-0.192645	-2.171955

17

TS(Bn—CO2−) r^2SCAN-3c SMD 2.60Å

C	-3.005936	0.674220	-1.832820
C	-4.358243	1.061469	-4.019439
C	-5.401821	0.149494	-3.756095
H	-3.568638	0.782577	-4.712853
H	-4.523654	2.124142	-3.860832
C	-5.341519	-1.224526	-4.157268
C	-6.583835	0.518941	-3.035060
C	-7.575159	-0.397670	-2.726799
C	-7.478821	-1.741159	-3.117512
C	-6.341748	-2.128815	-3.840976
H	-8.450054	-0.061340	-2.171523
H	-6.692772	1.553713	-2.712850
H	-6.240333	-3.162675	-4.169289
H	-4.473084	-1.562323	-4.721292
H	-8.261306	-2.454391	-2.875739
O	-3.448245	1.518642	-1.129826
O	-2.321549	-0.245872	-2.128634

17

TS(Bn—CO2−) r^2SCAN-3c SMD 2.75Å

C	-3.024575	0.616887	-1.755189
C	-4.384491	1.085361	-4.099045
C	-5.400800	0.169996	-3.793135
H	-3.560325	0.795663	-4.745443
H	-4.514323	2.143513	-3.888583
C	-5.339078	-1.215599	-4.174299
C	-6.578190	0.533480	-3.050562
C	-7.554162	-0.388262	-2.714673
C	-7.456319	-1.737824	-3.089829
C	-6.325790	-2.122218	-3.828592
H	-8.421135	-0.051468	-2.146636
H	-6.692076	1.570533	-2.736765
H	-6.219190	-3.159622	-4.145150
H	-4.478124	-1.555913	-4.748783
H	-8.227964	-2.454572	-2.825215
O	-3.548895	1.436568	-1.097070
O	-2.341277	-0.262100	-2.129841

17

TS(Bn—CO2−) r^2SCAN-3c SMD 2.90Å

C	-3.027401	0.568297	-1.684033
C	-4.416441	1.109848	-4.171459
C	-5.406133	0.187426	-3.825729
H	-3.563271	0.812165	-4.774999
H	-4.517340	2.160095	-3.911756
C	-5.336517	-1.206660	-4.186979
C	-6.577916	0.544414	-3.064997
C	-7.540563	-0.382762	-2.708287
C	-7.437747	-1.736234	-3.071388
C	-6.310772	-2.117198	-3.819256
H	-8.401958	-0.047293	-2.130648
H	-6.698812	1.582992	-2.758377
H	-6.196314	-3.157489	-4.124110
H	-4.479283	-1.547469	-4.767014
H	-8.199172	-2.457027	-2.788920
O	-3.578919	1.396671	-1.071037
O	-2.378153	-0.305351	-2.109821

17

TS(Bn—CO2−) r^2SCAN-3c SMD 3.05Å

C	-3.072003	0.454058	-1.624216
C	-4.453738	1.156762	-4.250910
C	-5.413233	0.221155	-3.863313
H	-3.582113	0.858987	-4.827694
H	-4.552893	2.204467	-3.979328
C	-5.317677	-1.181116	-4.189538
C	-6.583782	0.569783	-3.094577
C	-7.521168	-0.369084	-2.703548
C	-7.393853	-1.728908	-3.035004
C	-6.267093	-2.102898	-3.787673
H	-8.381820	-0.038279	-2.121937
H	-6.722941	1.612530	-2.810235
H	-6.132408	-3.148006	-4.067052
H	-4.458686	-1.518385	-4.769008
H	-8.134934	-2.459151	-2.724157
O	-3.600557	1.305464	-1.027784
O	-2.477817	-0.432956	-2.092836

<u>Geometry scan **TS(PhBn--CO2−) r^2SCAN-3c**:</u>

27

TS(PhBn—CO2−) r^2SCAN-3c 1.40Å

C	-3.645266	0.084065	-2.661607
C	-4.668454	0.715941	-3.378418
C	-5.979524	-0.029253	-3.443625
H	-4.321141	0.842826	-4.417314
H	-4.867773	1.723720	-2.993498
C	-6.063983	-1.322631	-3.979628
C	-7.153738	0.546367	-2.948246
C	-8.369387	-0.126413	-2.994136
C	-8.455608	-1.414765	-3.535135
C	-7.273930	-1.997621	-4.022098
H	-9.255714	0.337542	-2.566966
H	-7.106240	1.536859	-2.500924
H	-7.315628	-2.987266	-4.472275
H	-5.143191	-1.779769	-4.329095
C	-9.740150	-2.134222	-3.587199
O	-3.128490	0.735828	-1.712993
O	-3.317644	-1.075546	-3.054296
C	-10.944088	-1.457062	-3.839032
C	-9.804444	-3.522563	-3.387501
C	-11.015728	-4.201354	-3.439886
C	-12.201447	-3.513312	-3.687093
C	-12.156040	-2.135093	-3.884619
H	-10.917245	-0.387339	-4.027610
H	-13.148585	-4.044384	-3.725318
H	-13.071458	-1.585183	-4.089034
H	-8.890990	-4.065033	-3.160545
H	-11.035735	-5.275472	-3.272826

27

TS(PhBn—CO2−) r^2SCAN-3c 1.55Å

C	-3.644395	0.061552	-2.504421
C	-4.683418	0.718921	-3.448241
C	-5.985119	-0.004209	-3.507279
H	-4.216950	0.710180	-4.442954
H	-4.840441	1.761281	-3.154199
C	-6.077669	-1.294817	-4.053758
C	-7.153432	0.555347	-2.974638
C	-8.363980	-0.124929	-3.000465
C	-8.457119	-1.407869	-3.556442
C	-7.282640	-1.977581	-4.076329
H	-9.243124	0.329471	-2.548905
H	-7.101334	1.540871	-2.516885
H	-7.327338	-2.962577	-4.536057
H	-5.170887	-1.751968	-4.436076
C	-9.738283	-2.131923	-3.592580
O	-3.139182	0.830122	-1.656953
O	-3.413645	-1.148987	-2.731786
C	-10.953005	-1.455230	-3.792255
C	-9.792099	-3.526096	-3.429021

C	-11.001075	-4.209559	-3.467391
C	-12.196755	-3.521763	-3.662904
C	-12.162476	-2.138245	-3.822922
H	-10.937157	-0.380754	-3.952667
H	-13.142170	-4.056584	-3.689694
H	-13.085557	-1.587464	-3.986836
H	-8.871120	-4.070380	-3.240281
H	-11.011253	-5.287941	-3.328979

27

TS(PhBn—CO2−) r^{2}SCAN-3c 1.70Å

C	-3.653754	0.036383	-2.361744
C	-4.693590	0.722823	-3.518266
C	-5.984311	0.016636	-3.571261
H	-4.135527	0.609036	-4.453612
H	-4.810913	1.781065	-3.274738
C	-6.086755	-1.276319	-4.117711
C	-7.150355	0.567448	-3.014091
C	-8.355699	-0.117740	-3.021649
C	-8.456820	-1.401644	-3.579120
C	-7.287354	-1.964512	-4.118946
H	-9.228406	0.333473	-2.554518
H	-7.094647	1.551410	-2.553430
H	-7.333755	-2.949221	-4.579102
H	-5.190672	-1.734248	-4.524577
C	-9.734562	-2.128534	-3.597025
O	-3.252360	0.850504	-1.517711
O	-3.429181	-1.167453	-2.556362
C	-10.958062	-1.453393	-3.749956
C	-9.781485	-3.527063	-3.462197
C	-10.988554	-4.214004	-3.484540
C	-12.191880	-3.527383	-3.633542
C	-12.165369	-2.140329	-3.763567
H	-10.951411	-0.375801	-3.888263
H	-13.135930	-4.065067	-3.647241
H	-13.094055	-1.589211	-3.891340
H	-8.855092	-4.072779	-3.307484
H	-10.991123	-5.295206	-3.368924

27

TS(PhBn—CO2−) r^{2}SCAN-3c 1.85Å

C	-3.664790	0.028584	-2.222588
C	-4.700916	0.723192	-3.588773
C	-5.977684	0.026700	-3.629414
H	-4.067767	0.521129	-4.455476
H	-4.783786	1.790206	-3.380317
C	-6.092422	-1.270233	-4.173691
C	-7.143247	0.573691	-3.054707
C	-8.344322	-0.113700	-3.045832
C	-8.455439	-1.400528	-3.600375
C	-7.290165	-1.960227	-4.155237
H	-9.210148	0.338091	-2.566409
H	-7.083528	1.557287	-2.593753
H	-7.339259	-2.944974	-4.615128
H	-5.206181	-1.729887	-4.601271
C	-9.730699	-2.126955	-3.601136
O	-3.385823	0.885237	-1.388052
O	-3.416954	-1.159567	-2.405734
C	-10.960538	-1.451098	-3.704424
C	-9.775968	-3.529697	-3.499233
C	-10.982656	-4.216913	-3.506646
C	-12.190792	-3.529113	-3.605968
C	-12.167048	-2.138910	-3.702000
H	-10.960261	-0.370630	-3.817672
H	-13.134644	-4.067262	-3.607447
H	-13.098816	-1.585151	-3.791266
H	-8.846782	-4.079675	-3.381105
H	-10.980985	-5.300730	-3.417264

27

TS(PhBn—CO2−) r^{2}SCAN-3c 2.00Å

C	-3.702250	0.044252	-2.070963
C	-4.696823	0.710608	-3.673085
C	-5.960186	0.021860	-3.692193
H	-3.998436	0.425222	-4.459177
H	-4.749051	1.783577	-3.495783
C	-6.094480	-1.277866	-4.237646
C	-7.122549	0.569692	-3.098067
C	-8.321556	-0.115421	-3.071160
C	-8.449521	-1.404397	-3.623439
C	-7.291799	-1.964289	-4.197776
H	-9.176968	0.339627	-2.576144
H	-7.053271	1.551265	-2.634410
H	-7.349357	-2.947118	-4.660971
H	-5.221589	-1.739680	-4.691544
C	-9.724134	-2.125511	-3.604373
O	-3.697920	0.900921	-1.206898
O	-3.289792	-1.078621	-2.289894
C	-10.956997	-1.445331	-3.650790
C	-9.774441	-3.532024	-3.539961
C	-10.983050	-4.214948	-3.529830
C	-12.192313	-3.522397	-3.572345
C	-12.164794	-2.129677	-3.629887
H	-10.958679	-0.362320	-3.735355
H	-13.137716	-4.057585	-3.559612
H	-13.096564	-1.570278	-3.675028
H	-8.845481	-4.089708	-3.464154
H	-10.981905	-5.300985	-3.470433

27

TS(PhBn—CO2−) r^{2}SCAN-3c 2.15Å

C	-3.686902	0.024676	-1.967012
C	-4.709135	0.719901	-3.726046
C	-5.957052	0.031367	-3.727032
H	-3.969098	0.398306	-4.455790
H	-4.734136	1.789342	-3.530926
C	-6.097103	-1.277370	-4.261793
C	-7.125715	0.581456	-3.135263
C	-8.321063	-0.105403	-3.099632
C	-8.452315	-1.402500	-3.637673
C	-7.291965	-1.963619	-4.210327
H	-9.174675	0.355080	-2.606358
H	-7.059128	1.567861	-2.681210
H	-7.347646	-2.950062	-4.666176
H	-5.227881	-1.742670	-4.719992
C	-9.722971	-2.124081	-3.607654
O	-3.731047	0.909383	-1.147530
O	-3.285708	-1.087922	-2.201574
C	-10.960212	-1.446680	-3.620244
C	-9.773689	-3.533260	-3.566288
C	-10.980960	-4.217430	-3.546556
C	-12.192780	-3.527116	-3.555147
C	-12.166088	-2.133106	-3.589021
H	-10.967160	-0.362555	-3.687577
H	-13.137208	-4.063712	-3.534649
H	-13.098866	-1.573564	-3.608050
H	-8.844294	-4.092964	-3.515629
H	-10.976821	-5.304491	-3.505770

27

TS(PhBn—CO2−) r^{2}SCAN-3c 2.30Å

C	-3.723687	0.031089	-1.834321
C	-4.703899	0.709796	-3.801182
C	-5.940966	0.025911	-3.778749

H	-3.924798	0.343007	-4.463734
H	-4.700862	1.776817	-3.596646
C	-6.095916	-1.288721	-4.307821
C	-7.109048	0.581230	-3.179041
C	-8.303171	-0.102802	-3.129075
C	-8.447360	-1.404794	-3.658005
C	-7.291031	-1.969511	-4.240487
H	-9.149072	0.363924	-2.628322
H	-7.036202	1.568662	-2.728257
H	-7.352699	-2.956666	-4.694177
H	-5.236052	-1.758369	-4.780326
C	-9.716481	-2.121654	-3.610675
O	-4.023623	0.866671	-1.031555
O	-3.151302	-0.984121	-2.105922
C	-10.954400	-1.442441	-3.572286
C	-9.774325	-3.533111	-3.602556
C	-10.982802	-4.213515	-3.566723
C	-12.193271	-3.521001	-3.524563
C	-12.160703	-2.126143	-3.524499
H	-10.961995	-0.357092	-3.613243
H	-13.138651	-4.055184	-3.491255
H	-13.091369	-1.562814	-3.503891
H	-8.847340	-4.098847	-3.590603
H	-10.980598	-5.301454	-3.553005

27

TS(PhBn—CO_2^-) r^{2}SCAN-3c 2.45Å

C	-3.728066	-0.008995	-1.732292
C	-4.712439	0.724810	-3.852442
C	-5.937567	0.039535	-3.812894
H	-3.905947	0.335713	-4.466844
H	-4.688124	1.784671	-3.617355
C	-6.094745	-1.285581	-4.326839
C	-7.112941	0.598361	-3.220265
C	-8.303472	-0.087944	-3.162156
C	-8.447941	-1.399396	-3.672864
C	-7.287288	-1.966466	-4.249480
H	-9.149505	0.385132	-2.667529
H	-7.044996	1.592570	-2.783237
H	-7.345214	-2.959142	-4.691587
H	-5.235985	-1.759627	-4.797575
C	-9.712190	-2.118081	-3.614006
O	-4.057613	0.850412	-0.980627
O	-3.181647	-1.019202	-2.036265
C	-10.953697	-1.443903	-3.545428
C	-9.770025	-3.531408	-3.623802
C	-10.976147	-4.214398	-3.576691
C	-12.188408	-3.526441	-3.504553
C	-12.156971	-2.131138	-3.486335
H	-10.966874	-0.358231	-3.572049
H	-13.131978	-4.063134	-3.462428
H	-13.088035	-1.569460	-3.442562
H	-8.843117	-4.097197	-3.635055
H	-10.970689	-5.302594	-3.577759

27

TS(PhBn—CO_2^-) r^{2}SCAN-3c 2.60Å

C	-3.788637	-0.069429	-1.624011
C	-4.713181	0.742626	-3.914379
C	-5.927771	0.055693	-3.856155
H	-3.881479	0.330834	-4.477698
H	-4.669238	1.794355	-3.648710
C	-6.087871	-1.279567	-4.354146
C	-7.108236	0.617523	-3.266677
C	-8.295027	-0.071232	-3.196930
C	-8.440974	-1.391470	-3.689715
C	-7.277738	-1.960634	-4.263954
H	-9.139508	0.407810	-2.705344
H	-7.043596	1.617647	-2.842305
H	-7.332627	-2.958732	-4.694169
H	-5.231237	-1.758046	-4.824938
C	-9.700230	-2.111913	-3.615739
O	-4.192176	0.791695	-0.925064
O	-3.234333	-1.053389	-1.966369
C	-10.944196	-1.442773	-3.512643
C	-9.758945	-3.526820	-3.643646
C	-10.962597	-4.212133	-3.581399
C	-12.175573	-3.528757	-3.475165
C	-12.144302	-2.133281	-3.438629
H	-10.962679	-0.356984	-3.523982
H	-13.117198	-4.067753	-3.421312
H	-13.074837	-1.573083	-3.368392
H	-8.833013	-4.092944	-3.681739
H	-10.954423	-5.300375	-3.597707

27

TS(PhBn—CO_2^-) r^{2}SCAN-3c 2.75Å

C	-3.897429	-0.158385	-1.519182
C	-4.709377	0.766697	-3.978342
C	-5.915072	0.077717	-3.902485
H	-3.860134	0.337087	-4.500680
H	-4.650479	1.810491	-3.685587
C	-6.076725	-1.266893	-4.384640
C	-7.099316	0.640784	-3.313320
C	-8.281834	-0.051486	-3.230367
C	-8.428167	-1.379751	-3.706133
C	-7.263114	-1.949614	-4.280303
H	-9.124714	0.431881	-2.740207
H	-7.038005	1.646405	-2.901093
H	-7.314745	-2.952936	-4.698683
H	-5.221878	-1.748595	-4.855954
C	-9.681740	-2.103308	-3.615408
O	-4.349048	0.714598	-0.878338
O	-3.352419	-1.125746	-1.898507
C	-10.927883	-1.440251	-3.481026
C	-9.739856	-3.519591	-3.656439
C	-10.940092	-4.208403	-3.577493
C	-12.153747	-3.530663	-3.440586
C	-12.123939	-2.135084	-3.390737
H	-10.951808	-0.354567	-3.480623
H	-13.092688	-4.072839	-3.373894
H	-13.054163	-1.577557	-3.296759
H	-8.814598	-4.084573	-3.718898
H	-10.928652	-5.296550	-3.605234

27

TS(PhBn—CO_2^-) r^{2}SCAN-3c 2.90Å

C	-4.054602	-0.277593	-1.428609
C	-4.702111	0.798115	-4.042721
C	-5.900648	0.105862	-3.950383
H	-3.841250	0.355694	-4.534657
H	-4.633203	1.835399	-3.729376
C	-6.062067	-1.246425	-4.418110
C	-7.086953	0.668637	-3.359228
C	-8.264590	-0.028398	-3.261262
C	-8.409539	-1.363593	-3.721328
C	-7.244022	-1.932639	-4.298341
H	-9.105862	0.457100	-2.770407
H	-7.028580	1.679016	-2.957972
H	-7.292587	-2.940646	-4.705713
H	-5.208663	-1.729510	-4.890974
C	-9.656932	-2.092013	-3.612401
O	-4.531496	0.608332	-0.833721
O	-3.529799	-1.237548	-1.840128

C	-10.905169	-1.435768	-3.454018
C	-9.711522	-3.509462	-3.657921
C	-10.907231	-4.203328	-3.561099
C	-12.122005	-3.532022	-3.400775
C	-12.096215	-2.136207	-3.346183
H	-10.935150	-0.350287	-3.448628
H	-13.057376	-4.078439	-3.320286
H	-13.026891	-1.582563	-3.234235
H	-8.785907	-4.071444	-3.738915
H	-10.891252	-5.291403	-3.593525

27

TS(PhBn—CO2−) r²SCAN-3c 3.05Å

C	-4.210440	-0.399562	-1.353779
C	-4.696426	0.830792	-4.101969
C	-5.888141	0.133462	-3.992271
H	-3.831146	0.382599	-4.580643
H	-4.624306	1.865236	-3.780264
C	-6.048066	-1.224722	-4.447513
C	-7.074722	0.694007	-3.396205
C	-8.248023	-0.007480	-3.285100
C	-8.390940	-1.347556	-3.733698
C	-7.225582	-1.915115	-4.314333
H	-9.087695	0.477845	-2.791314
H	-7.018850	1.707303	-3.001698
H	-7.271467	-2.926925	-4.712479
H	-5.195431	-1.708670	-4.921158
C	-9.632821	-2.080868	-3.608927
O	-4.696967	0.496306	-0.787123
O	-3.701636	-1.354279	-1.789811
C	-10.882892	-1.430382	-3.436014
C	-9.682629	-3.499235	-3.651895
C	-10.873923	-4.198008	-3.539284
C	-12.090017	-3.532200	-3.364809
C	-12.069196	-2.135999	-3.312604
H	-10.918236	-0.345063	-3.431638
H	-13.021895	-4.082600	-3.272051
H	-13.000826	-1.586101	-3.190037
H	-8.755979	-4.057811	-3.743800
H	-10.853368	-5.286108	-3.570501

27

TS(PhBn—CO2−) r²SCAN-3c SMD 1.40Å

C	-3.899992	0.273470	-2.351114
C	-4.576991	0.600807	-3.532013
C	-5.923216	-0.089693	-3.587991
H	-4.006155	0.298987	-4.418779
H	-4.751026	1.682803	-3.597477
C	-6.070418	-1.332659	-4.210938
C	-7.044093	0.469771	-2.965691
C	-8.268508	-0.186833	-2.968269
C	-8.416885	-1.435253	-3.590382
C	-7.292560	-1.995664	-4.212094
H	-9.116793	0.265080	-2.460018
H	-6.946217	1.428092	-2.462464
H	-7.380985	-2.948909	-4.727462
H	-5.216053	-1.782932	-4.710346
C	-9.719439	-2.135003	-3.595391
O	-4.172981	0.960251	-1.320956
O	-3.096052	-0.705790	-2.393091
C	-10.921442	-1.420806	-3.712508
C	-9.787272	-3.531890	-3.482435
C	-11.012682	-4.189722	-3.487789
C	-12.198851	-3.467945	-3.603750
C	-12.146122	-2.080159	-3.715914
H	-10.894204	-0.340187	-3.824725
H	-13.155853	-3.982584	-3.606891
H	-13.064123	-1.506864	-3.816237
H	-8.871601	-4.105548	-3.365604
H	-11.041109	-5.271953	-3.390588

27

TS(PhBn—CO2−) r²SCAN-3c SMD 1.55Å

C	-3.864624	0.227743	-2.260433
C	-4.597194	0.613976	-3.570648
C	-5.929809	-0.069108	-3.625240
H	-3.989407	0.311406	-4.429581
H	-4.736017	1.700525	-3.590649
C	-6.079423	-1.320107	-4.237209
C	-7.055068	0.489035	-3.004136
C	-8.275910	-0.172901	-2.995057
C	-8.423151	-1.427966	-3.604682
C	-7.297851	-1.988063	-4.226137
H	-9.123575	0.280737	-2.487141
H	-6.962536	1.453489	-2.511133
H	-7.384006	-2.945900	-4.733501
H	-5.226877	-1.771729	-4.739035
C	-9.722271	-2.132774	-3.598557
O	-4.159341	0.911744	-1.248714
O	-3.071564	-0.743470	-2.338066
C	-10.928770	-1.424417	-3.708524
C	-9.784134	-3.530026	-3.482418
C	-11.006669	-4.193109	-3.478388
C	-12.196922	-3.476858	-3.587136
C	-12.150575	-2.088980	-3.701841
H	-10.907466	-0.343959	-3.823496
H	-13.151711	-3.995573	-3.582733
H	-13.071645	-1.519640	-3.796791
H	-8.865489	-4.099893	-3.370546
H	-11.029617	-5.275312	-3.379126

27

TS(PhBn—CO2−) r²SCAN-3c SMD 1.70Å

C	-3.836563	0.180070	-2.168627
C	-4.616103	0.628406	-3.611302
C	-5.934818	-0.048276	-3.663046
H	-3.973039	0.320793	-4.440033
H	-4.722841	1.716595	-3.587583
C	-6.085889	-1.309117	-4.261531
C	-7.065577	0.510198	-3.045661
C	-8.282484	-0.157023	-3.025364
C	-8.428259	-1.420206	-3.619856
C	-7.300643	-1.981181	-4.238364
H	-9.129869	0.299616	-2.519476
H	-6.978468	1.481159	-2.564038
H	-7.383593	-2.944591	-4.735847
H	-5.233785	-1.763573	-4.762079
C	-9.723570	-2.129905	-3.602137
O	-4.155621	0.865337	-1.179415
O	-3.058837	-0.785923	-2.278419
C	-10.935123	-1.427304	-3.699616
C	-9.779749	-3.528054	-3.487243
C	-10.999307	-4.196302	-3.473209
C	-12.193905	-3.485339	-3.569169
C	-12.153922	-2.096988	-3.681776
H	-10.920419	-0.346693	-3.813998
H	-13.146422	-4.008064	-3.556498
H	-13.078133	-1.531105	-3.766943
H	-8.858218	-4.094858	-3.384132
H	-11.016464	-5.278804	-3.375554

27

TS(PhBn—CO2−) r²SCAN-3c SMD 1.85Å

C	-3.816278	0.124812	-2.074652

C	-4.633888	0.645504	-3.650372
C	-5.938024	-0.025388	-3.700484
H	-3.955735	0.331075	-4.445939
H	-4.709879	1.732560	-3.580584
C	-6.089878	-1.297599	-4.283896
C	-7.077493	0.535688	-3.093194
C	-8.290230	-0.136460	-3.063609
C	-8.433254	-1.409938	-3.638820
C	-7.301101	-1.972731	-4.250294
H	-9.138402	0.325267	-2.563476
H	-6.997392	1.514389	-2.625556
H	-7.379090	-2.942793	-4.735793
H	-5.236827	-1.756801	-4.778816
C	-9.723574	-2.125520	-3.607989
O	-4.137702	0.829649	-1.114389
O	-3.082021	-0.856773	-2.214878
C	-10.941600	-1.430249	-3.686938
C	-9.772706	-3.525104	-3.497364
C	-10.988287	-4.199903	-3.470516
C	-12.188340	-3.495604	-3.547301
C	-12.156335	-2.106484	-3.654704
H	-10.935740	-0.349277	-3.798301
H	-13.137833	-4.023399	-3.523738
H	-13.084400	-1.544751	-3.725051
H	-8.847697	-4.088379	-3.407327
H	-10.997914	-5.282923	-3.376936

27

TS(PhBn—CO2−) r²SCAN-3c SMD 2.00Å

C	-3.787133	0.067530	-1.987945
C	-4.654607	0.669034	-3.686675
C	-5.942024	-0.001225	-3.729385
H	-3.945575	0.348264	-4.450475
H	-4.704215	1.751936	-3.569483
C	-6.093072	-1.285645	-4.297403
C	-7.093108	0.561678	-3.135206
C	-8.300940	-0.115953	-3.098982
C	-8.439218	-1.400570	-3.653624
C	-7.300136	-1.964469	-4.254674
H	-9.151518	0.351119	-2.607706
H	-7.022262	1.548452	-2.682368
H	-7.371176	-2.941523	-4.727414
H	-5.238461	-1.748920	-4.785854
C	-9.723865	-2.122138	-3.612912
O	-4.045914	0.829612	-1.067229
O	-3.154168	-0.964568	-2.159141
C	-10.948396	-1.434658	-3.676436
C	-9.766436	-3.523363	-3.507042
C	-10.977848	-4.204831	-3.470824
C	-12.183160	-3.507586	-3.531499
C	-12.158786	-2.117679	-3.633328
H	-10.951404	-0.353401	-3.784794
H	-13.129465	-4.040613	-3.499813
H	-13.090380	-1.560233	-3.691493
H	-8.838314	-4.083042	-3.427677
H	-10.980039	-5.288339	-3.381536

27

TS(PhBn—CO2−) r²SCAN-3c SMD 2.15Å

C	-3.784250	0.022135	-1.892924
C	-4.668179	0.685830	-3.737017
C	-5.939546	0.016509	-3.764878
H	-3.925437	0.343954	-4.456376
H	-4.690898	1.763937	-3.584792
C	-6.094050	-1.279288	-4.318826
C	-7.098651	0.581234	-3.175858
C	-8.302273	-0.099742	-3.130404
C	-8.440955	-1.393816	-3.667712
C	-7.297677	-1.959543	-4.263620
H	-9.152375	0.372162	-2.642557
H	-7.030847	1.573059	-2.733161
H	-7.365064	-2.942361	-4.725198
H	-5.239672	-1.746994	-4.804015
C	-9.720861	-2.118216	-3.615495
O	-4.083579	0.796207	-1.009612
O	-3.168953	-1.001816	-2.097725
C	-10.950704	-1.435626	-3.655565
C	-9.760577	-3.521792	-3.521680
C	-10.969200	-4.207069	-3.475597
C	-12.178195	-3.513958	-3.512338
C	-12.158002	-2.122798	-3.601190
H	-10.960416	-0.353623	-3.755487
H	-13.122371	-4.050132	-3.472296
H	-13.091750	-1.567133	-3.641121
H	-8.830800	-4.080772	-3.459061
H	-10.966339	-5.291476	-3.396413

27

TS(PhBn—CO2−) r²SCAN-3c SMD 2.30Å

C	-3.784488	-0.017012	-1.799738
C	-4.681119	0.703970	-3.791280
C	-5.935315	0.031917	-3.796999
H	-3.905995	0.338310	-4.461450
H	-4.678765	1.775403	-3.602764
C	-6.092657	-1.276994	-4.333879
C	-7.103183	0.598523	-3.213138
C	-8.302239	-0.085908	-3.158381
C	-8.441256	-1.390089	-3.676940
C	-7.292764	-1.958300	-4.265815
H	-9.152176	0.391539	-2.675303
H	-7.039906	1.596250	-2.782656
H	-7.355848	-2.947301	-4.715006
H	-5.239114	-1.749563	-4.816273
C	-9.716752	-2.115727	-3.615693
O	-4.106893	0.787091	-0.967609
O	-3.203601	-1.040600	-2.039876
C	-10.951034	-1.436500	-3.629526
C	-9.756452	-3.522160	-3.540053
C	-10.963282	-4.209522	-3.487622
C	-12.174753	-3.519040	-3.497853
C	-12.156074	-2.126369	-3.567422
H	-10.966178	-0.353460	-3.716257
H	-13.117530	-4.057174	-3.452407
H	-13.090750	-1.570904	-3.587108
H	-8.826448	-4.082464	-3.496444
H	-10.957049	-5.295048	-3.423429

27

TS(PhBn—CO2−) r²SCAN-3c SMD 2.45Å

C	-3.804553	-0.053000	-1.701816
C	-4.689841	0.721302	-3.851052
C	-5.927123	0.045177	-3.830789
H	-3.882350	0.328925	-4.464046
H	-4.663559	1.784840	-3.627969
C	-6.086446	-1.279330	-4.344336
C	-7.104315	0.614912	-3.253952
C	-8.298743	-0.072408	-3.189156
C	-8.438542	-1.388176	-3.685348
C	-7.283265	-1.960685	-4.263296
H	-9.148546	0.412443	-2.712997
H	-7.045882	1.619599	-2.838624
H	-7.340672	-2.957398	-4.696213
H	-5.232821	-1.758839	-4.820232
C	-9.709629	-2.113485	-3.615106

O	-4.160503	0.785494	-0.935963
O	-3.254646	-1.070660	-1.981130
C	-10.947584	-1.436502	-3.591914
C	-9.752511	-3.523333	-3.568401
C	-10.958357	-4.211061	-3.509762
C	-12.171043	-3.522073	-3.482994
C	-12.150946	-2.127723	-3.522289
H	-10.967285	-0.352053	-3.656394
H	-13.113019	-4.061038	-3.432305
H	-13.085192	-1.570923	-3.513039
H	-8.823901	-4.087238	-3.552545
H	-10.950347	-5.297897	-3.469250

27

TS(PhBn—CO2−) r²SCAN-3c SMD 2.60Å

C	-3.874176	-0.088121	-1.595663
C	-4.690289	0.735946	-3.922652
C	-5.910106	0.055490	-3.871546
H	-3.847540	0.309383	-4.459425
H	-4.640470	1.789829	-3.663472
C	-6.070266	-1.289463	-4.348421
C	-7.098035	0.632337	-3.307891
C	-8.287904	-0.056667	-3.230286
C	-8.429127	-1.387518	-3.694516
C	-7.264519	-1.968725	-4.253197
H	-9.137787	0.440728	-2.767384
H	-7.045411	1.646999	-2.916113
H	-7.313573	-2.976841	-4.659933
H	-5.215040	-1.780928	-4.809651
C	-9.695869	-2.110432	-3.612996
O	-4.304941	0.775554	-0.919573
O	-3.323370	-1.077599	-1.921326
C	-10.935536	-1.435041	-3.528807
C	-9.747605	-3.523756	-3.616849
C	-10.953521	-4.209389	-3.549774
C	-12.164648	-3.521168	-3.462853
C	-12.137896	-2.125760	-3.449816
H	-10.958158	-0.349179	-3.551720
H	-13.106579	-4.059364	-3.405317
H	-13.068926	-1.566068	-3.392155
H	-8.823630	-4.094165	-3.649121
H	-10.946699	-5.297214	-3.550461

27

TS(PhBn—CO2−) r²SCAN-3c SMD 2.75Å

C	-3.987013	-0.165952	-1.491571
C	-4.684911	0.755359	-3.986892
C	-5.894486	0.074343	-3.917398
H	-3.817751	0.301037	-4.457519
H	-4.615729	1.798084	-3.690026
C	-6.054854	-1.284037	-4.368594
C	-7.090155	0.656760	-3.364890
C	-8.276403	-0.034160	-3.276117
C	-8.417199	-1.376244	-3.714701
C	-7.246782	-1.962530	-4.261971
H	-9.126131	0.472057	-2.822557
H	-7.041959	1.678274	-2.990409
H	-7.289019	-2.978846	-4.648447
H	-5.198073	-1.783982	-4.818052
C	-9.677923	-2.101488	-3.615307
O	-4.476916	0.710712	-0.890574
O	-3.432603	-1.127394	-1.863442
C	-10.919365	-1.432438	-3.483096
C	-9.731867	-3.516626	-3.646971
C	-10.934600	-4.204954	-3.561422
C	-12.145276	-3.522369	-3.427315
C	-12.117695	-2.127015	-3.385662
H	-10.947356	-0.346637	-3.481026
H	-13.084541	-4.063311	-3.354909
H	-13.046975	-1.569319	-3.290072
H	-8.810614	-4.087795	-3.716841
H	-10.925425	-5.292663	-3.585137

27

TS(PhBn—CO2−) r²SCAN-3c SMD 2.90Å

C	-4.045949	-0.265069	-1.418713
C	-4.692812	0.788808	-4.041863
C	-5.892486	0.098693	-3.949614
H	-3.812083	0.323323	-4.475169
H	-4.620184	1.826963	-3.729278
C	-6.046782	-1.269677	-4.377679
C	-7.092383	0.680145	-3.400573
C	-8.274286	-0.015506	-3.302068
C	-8.410019	-1.364797	-3.723008
C	-7.234763	-1.952247	-4.261023
H	-9.125305	0.492615	-2.852987
H	-7.049179	1.706228	-3.037778
H	-7.269818	-2.975016	-4.630862
H	-5.187106	-1.772763	-4.818218
C	-9.666196	-2.093934	-3.613808
O	-4.562569	0.605444	-0.838449
O	-3.487644	-1.196668	-1.845529
C	-10.910266	-1.429600	-3.473325
C	-9.716970	-3.510093	-3.643715
C	-10.916755	-4.202026	-3.548748
C	-12.129252	-3.523845	-3.406472
C	-12.105316	-2.128152	-3.366566
H	-10.942856	-0.343967	-3.472005
H	-13.066145	-4.067825	-3.326718
H	-13.035601	-1.573110	-3.264794
H	-8.795062	-4.079277	-3.720271
H	-10.903833	-5.289778	-3.571686

27

TS(PhBn—CO2−) r²SCAN-3c SMD 3.05Å

C	-4.060540	-0.403517	-1.371478
C	-4.710340	0.834186	-4.082263
C	-5.900864	0.132834	-3.971921
H	-3.819827	0.365619	-4.491953
H	-4.641618	1.871565	-3.765273
C	-6.043345	-1.245047	-4.375692
C	-7.107077	0.712374	-3.432784
C	-8.283814	0.009512	-3.327536
C	-8.408396	-1.347614	-3.727265
C	-7.226094	-1.934808	-4.250867
H	-9.140148	0.517757	-2.888648
H	-7.073146	1.744250	-3.085451
H	-7.251691	-2.964297	-4.602559
H	-5.178699	-1.748888	-4.805500
C	-9.659154	-2.083683	-3.611281
O	-4.606308	0.436167	-0.776142
O	-3.485352	-1.290915	-1.860750
C	-10.909132	-1.426128	-3.489590
C	-9.699876	-3.500779	-3.615362
C	-10.895102	-4.199490	-3.513823
C	-12.113374	-3.527807	-3.390350
C	-12.099580	-2.131360	-3.376559
H	-10.949342	-0.340880	-3.508664
H	-13.046744	-4.077046	-3.305606
H	-13.034439	-1.581333	-3.290326
H	-8.773396	-4.064417	-3.676691
H	-10.874221	-5.287386	-3.516584

Geometry scan **TS(F3CBn--CO2−) r²SCAN-3c:**

20

TS(F3CBn—CO2−) r^{2}SCAN-3c 1.40Å

C	-3.306051	0.614139	-2.176016
C	-3.809214	0.221778	-3.422162
C	-5.226288	-0.291790	-3.428068
H	-3.167998	-0.531466	-3.895897
H	-3.785729	1.105419	-4.081446
C	-5.506371	-1.598560	-3.840885
C	-6.295489	0.508021	-2.996105
C	-7.592654	0.019329	-2.992307
C	-7.853779	-1.288931	-3.412511
C	-6.803149	-2.099490	-3.838471
H	-8.409656	0.649639	-2.652793
H	-6.060858	1.501170	-2.626988
H	-6.997110	-3.119341	-4.157126
H	-4.686596	-2.237576	-4.160111
C	-9.254986	-1.790334	-3.470078
O	-3.996446	1.457952	-1.531220
O	-2.199636	0.117303	-1.831646
F	-10.053574	-1.240920	-2.517684
F	-9.340040	-3.139802	-3.320335
F	-9.866350	-1.514972	-4.665808

20

TS(F3CBn—CO2−) r^{2}SCAN-3c 1.55Å

C	-3.282173	0.559290	-2.073585
C	-3.833666	0.246116	-3.487897
C	-5.228291	-0.273776	-3.488064
H	-3.165099	-0.454240	-3.997350
H	-3.801200	1.203137	-4.026295
C	-5.506552	-1.599296	-3.847505
C	-6.302210	0.532090	-3.072449
C	-7.595667	0.037580	-3.039328
C	-7.853349	-1.286226	-3.411244
C	-6.799097	-2.105750	-3.815234
H	-8.412938	0.674611	-2.713408
H	-6.082928	1.541390	-2.741202
H	-6.990078	-3.136878	-4.097613
H	-4.685581	-2.244555	-4.150744
C	-9.250824	-1.795093	-3.446416
O	-4.012083	1.297495	-1.372284
O	-2.160365	0.067182	-1.826356
F	-10.051876	-1.211483	-2.515900
F	-9.330372	-3.138270	-3.243060
F	-9.867623	-1.571758	-4.651724

20

TS(F3CBn—CO2−) r^{2}SCAN-3c 1.70Å

C	-3.267083	0.502765	-1.977255
C	-3.856940	0.278691	-3.555818
C	-5.229337	-0.248566	-3.545331
H	-3.163356	-0.380606	-4.081737
H	-3.817334	1.287375	-3.979866
C	-5.503201	-1.591003	-3.860036
C	-6.312498	0.556264	-3.140849
C	-7.599207	0.051438	-3.077932
C	-7.850413	-1.285064	-3.409120
C	-6.789006	-2.105392	-3.799385
H	-8.418199	0.690544	-2.760174
H	-6.107605	1.579513	-2.843833
H	-6.973067	-3.145827	-4.051201
H	-4.679772	-2.238206	-4.152210
C	-9.241424	-1.805097	-3.420058
O	-3.980267	1.247877	-1.289482
O	-2.198231	-0.086314	-1.766813
F	-10.042079	-1.202673	-2.499782
F	-9.309679	-3.143523	-3.180442
F	-9.873272	-1.620630	-4.626336

20

TS(F3CBn—CO2−) r^{2}SCAN-3c 1.85Å

C	-3.248467	0.436732	-1.885520
C	-3.881071	0.315905	-3.619796
C	-5.232165	-0.217638	-3.594318
H	-3.165135	-0.309943	-4.152736
H	-3.830220	1.360713	-3.932910
C	-5.498960	-1.575889	-3.869538
C	-6.328850	0.583475	-3.205036
C	-7.607385	0.066065	-3.117105
C	-7.848361	-1.282772	-3.407908
C	-6.776600	-2.099929	-3.784068
H	-8.430742	0.705566	-2.811126
H	-6.141217	1.619925	-2.942267
H	-6.950070	-3.149167	-4.006061
H	-4.670980	-2.222867	-4.149058
C	-9.230435	-1.817418	-3.395237
O	-3.924315	1.212726	-1.217065
O	-2.251428	-0.259422	-1.721032
F	-10.034488	-1.197061	-2.488031
F	-9.283485	-3.149884	-3.116670
F	-9.877599	-1.677550	-4.602177

20

TS(F3CBn—CO2−) r^{2}SCAN-3c 2.00Å

C	-3.224982	0.377402	-1.797639
C	-3.904300	0.350369	-3.678542
C	-5.235424	-0.189403	-3.633516
H	-3.166574	-0.253996	-4.203246
H	-3.839976	1.415596	-3.898826
C	-5.495871	-1.560725	-3.877687
C	-6.346750	0.607211	-3.259329
C	-7.617107	0.078179	-3.151871
C	-7.848041	-1.281079	-3.407350
C	-6.765786	-2.093298	-3.772197
H	-8.445600	0.717266	-2.858424
H	-6.175197	1.654517	-3.028200
H	-6.928436	-3.149491	-3.969142
H	-4.663547	-2.206771	-4.146450
C	-9.220881	-1.828542	-3.374254
O	-3.861180	1.186879	-1.151482
O	-2.299961	-0.404850	-1.687894
F	-10.028600	-1.195308	-2.477024
F	-9.259939	-3.155860	-3.064844
F	-9.883820	-1.726527	-4.579740

20

TS(F3CBn—CO2−) r^{2}SCAN-3c 2.15Å

C	-3.165600	0.211670	-1.737670
C	-3.961996	0.474575	-3.717351
C	-5.261292	-0.103735	-3.655053
H	-3.199523	-0.083342	-4.256306
H	-3.903869	1.555881	-3.814229
C	-5.471280	-1.500082	-3.827467
C	-6.417043	0.667226	-3.347386
C	-7.666553	0.096752	-3.230063
C	-7.843442	-1.284725	-3.407305
C	-6.720739	-2.071081	-3.709582
H	-8.524726	0.720762	-2.993550
H	-6.298269	1.736634	-3.190246
H	-6.838196	-3.142998	-3.844592
H	-4.609691	-2.128579	-4.036417
C	-9.193412	-1.876067	-3.361970
O	-3.232929	1.276905	-1.175915

O	-2.818998	-0.938991	-1.642926
F	-10.022801	-1.254649	-2.472013
F	-9.190393	-3.199135	-3.031973
F	-9.871222	-1.815455	-4.565643

20

TS(F3CBn—CO2−) r^2SCAN-3c 2.30Å

C	-3.150276	0.195475	-1.646729
C	-3.975626	0.485010	-3.773926
C	-5.260825	-0.093909	-3.687906
H	-3.188563	-0.081280	-4.264771
H	-3.898285	1.567004	-3.830739
C	-5.474783	-1.494996	-3.857899
C	-6.422473	0.673192	-3.369453
C	-7.666489	0.098523	-3.236766
C	-7.844255	-1.285805	-3.406818
C	-6.719985	-2.067481	-3.724009
H	-8.523657	0.721243	-2.992378
H	-6.307747	1.744052	-3.217673
H	-6.835452	-3.140037	-3.857618
H	-4.615842	-2.123209	-4.079681
C	-9.188255	-1.880098	-3.345574
O	-3.203759	1.278513	-1.139166
O	-2.857360	-0.962559	-1.583642
F	-10.015002	-1.254852	-2.453588
F	-9.179336	-3.202272	-3.005908
F	-9.884002	-1.834946	-4.543412

20

TS(F3CBn—CO2−) r^2SCAN-3c 2.45Å

C	-3.145736	0.173824	-1.553835
C	-3.984888	0.494729	-3.833165
C	-5.258059	-0.084897	-3.722381
H	-3.178290	-0.079151	-4.280123
H	-3.892773	1.576283	-3.856421
C	-5.476335	-1.490627	-3.886809
C	-6.423968	0.679716	-3.393237
C	-7.662950	0.102130	-3.244002
C	-7.842530	-1.285064	-3.404869
C	-6.717577	-2.063597	-3.735864
H	-8.518419	0.724596	-2.992186
H	-6.312028	1.751999	-3.248026
H	-6.831813	-3.136948	-3.866087
H	-4.620365	-2.119304	-4.120063
C	-9.181009	-1.880840	-3.326697
O	-3.235217	1.261635	-1.082492
O	-2.863634	-0.979889	-1.543467
F	-10.003789	-1.250854	-2.432235
F	-9.167286	-3.202006	-2.977631
F	-9.895306	-1.850166	-4.518066

20

TS(F3CBn—CO2−) r^2SCAN-3c 2.60Å

C	-3.212754	0.158409	-1.452001
C	-3.974722	0.485903	-3.916175
C	-5.239363	-0.086807	-3.771320
H	-3.155426	-0.101029	-4.319825
H	-3.862726	1.565668	-3.906780
C	-5.473814	-1.494467	-3.937387
C	-6.399267	0.681461	-3.411825
C	-7.635315	0.108592	-3.235722
C	-7.828515	-1.278787	-3.395721
C	-6.713165	-2.060150	-3.759478
H	-8.481024	0.734815	-2.960618
H	-6.279856	1.752632	-3.263807
H	-6.835898	-3.132338	-3.893677
H	-4.628776	-2.127864	-4.198208
C	-9.164386	-1.867635	-3.290745
O	-3.534238	1.198184	-0.994338
O	-2.763613	-0.931110	-1.519604
F	-9.972267	-1.228299	-2.387651
F	-9.151151	-3.188284	-2.932852
F	-9.905698	-1.847326	-4.469924

20

TS(F3CBn—CO2−) r^2SCAN-3c 2.75Å

C	-3.299787	0.100470	-1.359586
C	-3.971587	0.504962	-3.995411
C	-5.224456	-0.070309	-3.818469
H	-3.139991	-0.087057	-4.365003
H	-3.848952	1.582827	-3.953969
C	-5.460516	-1.483273	-3.970081
C	-6.385739	0.695169	-3.442474
C	-7.614467	0.118277	-3.239405
C	-7.807055	-1.272600	-3.383610
C	-6.693399	-2.051031	-3.764856
H	-8.457420	0.743721	-2.953534
H	-6.269817	1.768383	-3.305339
H	-6.814034	-3.124760	-3.889884
H	-4.619190	-2.116573	-4.243846
C	-9.136225	-1.864044	-3.253000
O	-3.661663	1.137957	-0.946427
O	-2.856047	-0.979039	-1.483500
F	-9.934168	-1.221323	-2.341662
F	-9.114649	-3.183624	-2.887390
F	-9.902811	-1.856567	-4.420210

20

TS(F3CBn—CO2−) r^2SCAN-3c 2.90Å

C	-3.420574	0.040602	-1.270627
C	-3.962627	0.520218	-4.078856
C	-5.205578	-0.056204	-3.868140
H	-3.125996	-0.075838	-4.430329
H	-3.831533	1.596170	-4.015981
C	-5.444608	-1.472729	-4.007569
C	-6.363645	0.707424	-3.468916
C	-7.585135	0.128386	-3.236366
C	-7.779310	-1.264793	-3.368609
C	-6.670867	-2.041414	-3.772722
H	-8.422795	0.753627	-2.934424
H	-6.248133	1.781651	-3.338512
H	-6.791382	-3.116068	-3.891057
H	-4.608940	-2.106230	-4.298487
C	-9.102578	-1.856989	-3.210548
O	-3.849774	1.063657	-0.901665
O	-2.943552	-1.014355	-1.433963
F	-9.886494	-1.211405	-2.287708
F	-9.073281	-3.176019	-2.839868
F	-9.895171	-1.858123	-4.363311

20

TS(F3CBn—CO2−) r^2SCAN-3c 3.05Å

C	-3.546905	-0.033504	-1.192712
C	-3.956539	0.543874	-4.159417
C	-5.188953	-0.037070	-3.914461
H	-3.118195	-0.053297	-4.505094
H	-3.824822	1.619511	-4.091727
C	-5.424557	-1.457612	-4.034735
C	-6.345035	0.723249	-3.497560
C	-7.558415	0.140096	-3.237308
C	-7.748693	-1.255834	-3.353588
C	-6.642958	-2.029988	-3.772216
H	-8.393512	0.763503	-2.924423
H	-6.233016	1.799092	-3.376915

H	-6.760056	-3.106345	-3.878417
H	-4.591140	-2.090254	-4.334236
C	-9.065502	-1.851902	-3.169637
O	-3.985044	0.992897	-0.852518
O	-3.085784	-1.088015	-1.384220
F	-9.837512	-1.204021	-2.237497
F	-9.025514	-3.169192	-2.792155
F	-9.879819	-1.863623	-4.308822

20

TS(F3CBn—CO2−) r²SCAN-3c SMD 1.40Å

C	-3.261552	0.358893	-2.165877
C	-3.844932	0.336329	-3.438339
C	-5.252595	-0.208571	-3.452759
H	-3.243094	-0.242780	-4.148122
H	-3.882126	1.366202	-3.825162
C	-5.478068	-1.558404	-3.738671
C	-6.351148	0.600936	-3.142293
C	-7.638264	0.082155	-3.125241
C	-7.842797	-1.270615	-3.407138
C	-6.761353	-2.093659	-3.716647
H	-8.481651	0.725953	-2.892452
H	-6.186436	1.648308	-2.910028
H	-6.916506	-3.144772	-3.940560
H	-4.634507	-2.201098	-3.979024
C	-9.233541	-1.814425	-3.445747
O	-3.899689	0.967163	-1.255778
O	-2.139467	-0.209967	-2.025471
F	-10.053287	-1.220796	-2.541274
F	-9.282786	-3.147517	-3.203051
F	-9.828173	-1.631769	-4.664022

20

TS(F3CBn—CO2−) r²SCAN-3c SMD 1.55Å

C	-3.251816	0.375399	-2.068527
C	-3.857575	0.332488	-3.494611
C	-5.249907	-0.209598	-3.503389
H	-3.214959	-0.266447	-4.146150
H	-3.861709	1.365729	-3.865275
C	-5.486005	-1.560160	-3.789609
C	-6.346367	0.597444	-3.168072
C	-7.631973	0.078299	-3.125678
C	-7.844523	-1.273074	-3.409787
C	-6.768088	-2.093980	-3.745798
H	-8.469745	0.721513	-2.871604
H	-6.179820	1.644651	-2.935791
H	-6.927683	-3.143947	-3.972528
H	-4.648503	-2.203768	-4.048304
C	-9.234413	-1.816070	-3.423199
O	-3.922344	0.998402	-1.210044
O	-2.144805	-0.198576	-1.925003
F	-10.039334	-1.220219	-2.506256
F	-9.280150	-3.149128	-3.176584
F	-9.852255	-1.637390	-4.631450

20

TS(F3CBn—CO2−) r²SCAN-3c SMD 1.70Å

C	-3.269438	0.325018	-1.974024
C	-3.876800	0.378986	-3.560907
C	-5.248114	-0.175201	-3.564654
H	-3.202993	-0.203684	-4.192878
H	-3.866454	1.433109	-3.853212
C	-5.476701	-1.535147	-3.832713
C	-6.354922	0.620183	-3.220922
C	-7.631023	0.083778	-3.145171
C	-7.832690	-1.273728	-3.408996
C	-6.749482	-2.082586	-3.759067
H	-8.471564	0.719258	-2.880690
H	-6.201569	1.673729	-3.005934
H	-6.899609	-3.136775	-3.973323
H	-4.636745	-2.171428	-4.100826
C	-9.212413	-1.836597	-3.386816
O	-3.784290	1.155421	-1.204823
O	-2.394417	-0.538904	-1.785440
F	-10.012466	-1.230789	-2.471384
F	-9.234332	-3.164556	-3.107416
F	-9.855951	-1.698520	-4.588460

20

TS(F3CBn—CO2−) r²SCAN-3c SMD 1.85Å

C	-3.253225	0.278151	-1.885232
C	-3.898758	0.407721	-3.614104
C	-5.249979	-0.152272	-3.607043
H	-3.195793	-0.157101	-4.227831
H	-3.873865	1.477244	-3.831810
C	-5.475461	-1.521849	-3.853237
C	-6.368117	0.637707	-3.270006
C	-7.636952	0.091020	-3.172732
C	-7.831520	-1.273499	-3.409986
C	-6.741490	-2.076768	-3.758711
H	-8.480392	0.724577	-2.912189
H	-6.226588	1.697943	-3.078569
H	-6.883904	-3.135613	-3.955622
H	-4.633465	-2.156451	-4.119079
C	-9.203336	-1.846831	-3.364459
O	-3.705901	1.153101	-1.145347
O	-2.474386	-0.665364	-1.736451
F	-10.001250	-1.232071	-2.451625
F	-9.212200	-3.170805	-3.062322
F	-9.865390	-1.737273	-4.561303

20

TS(F3CBn—CO2−) r²SCAN-3c SMD 2.00Å

C	-3.244053	0.257823	-1.793037
C	-3.913429	0.416913	-3.670969
C	-5.247676	-0.140949	-3.646605
H	-3.183223	-0.151505	-4.246017
H	-3.865098	1.492436	-3.842812
C	-5.478617	-1.516585	-3.884398
C	-6.372202	0.646074	-3.302380
C	-7.635525	0.094961	-3.185521
C	-7.831521	-1.273352	-3.409772
C	-6.740093	-2.073081	-3.769960
H	-8.477612	0.728085	-2.918700
H	-6.234377	1.708660	-3.119866
H	-6.880958	-3.133591	-3.960198
H	-4.639026	-2.151367	-4.158110
C	-9.197386	-1.849688	-3.344007
O	-3.720288	1.134458	-1.089871
O	-2.481472	-0.689641	-1.683308
F	-9.989363	-1.230301	-2.427243
F	-9.199813	-3.172505	-3.031389
F	-9.880240	-1.755278	-4.533494

20

TS(F3CBn—CO2−) r²SCAN-3c SMD 2.15Å

C	-3.237993	0.236736	-1.703696
C	-3.927045	0.425926	-3.731482
C	-5.244298	-0.130609	-3.683084
H	-3.171424	-0.147644	-4.264561
H	-3.855852	1.505528	-3.853632
C	-5.481304	-1.512617	-3.912937
C	-6.374787	0.654092	-3.329562
C	-7.632607	0.099431	-3.194581

C	-7.831139	-1.272528	-3.407612
C	-6.738449	-2.069346	-3.779515
H	-8.472799	0.732627	-2.921088
H	-6.239559	1.718372	-3.153557
H	-6.878416	-3.131302	-3.963895
H	-4.644590	-2.148110	-4.194460
C	-9.190827	-1.850876	-3.325087
O	-3.753911	1.105748	-1.039282
O	-2.476930	-0.702152	-1.641601
F	-9.981425	-1.222683	-2.411197
F	-9.188637	-3.171310	-2.996323
F	-9.889979	-1.777715	-4.510505

20

TS(F3CBn—CO2−) r^{2}SCAN-3c SMD 2.30Å

C	-3.241810	0.206997	-1.614385
C	-3.938288	0.438636	-3.794124
C	-5.238865	-0.117683	-3.719210
H	-3.159270	-0.140098	-4.284104
H	-3.845476	1.520336	-3.861147
C	-5.482245	-1.506611	-3.941304
C	-6.376161	0.664041	-3.355530
C	-7.627740	0.105509	-3.201669
C	-7.828616	-1.270544	-3.402381
C	-6.734334	-2.063730	-3.787852
H	-8.465939	0.738767	-2.921321
H	-6.244244	1.730164	-3.186469
H	-6.873120	-3.127214	-3.966303
H	-4.648677	-2.142717	-4.231536
C	-9.180765	-1.851717	-3.304411
O	-3.797492	1.070533	-0.996792
O	-2.487481	-0.725106	-1.608624
F	-9.975526	-1.209019	-2.402264
F	-9.173639	-3.167458	-2.949311
F	-9.892283	-1.811520	-4.488921

20

TS(F3CBn—CO2−) r^{2}SCAN-3c SMD 2.45Å

C	-3.292108	0.150889	-1.526499
C	-3.947783	0.472371	-3.865139
C	-5.229030	-0.091565	-3.760044
H	-3.143507	-0.107513	-4.309770
H	-3.838157	1.553548	-3.868122
C	-5.470013	-1.489785	-3.968833
C	-6.377752	0.681796	-3.386951
C	-7.618721	0.112214	-3.210435
C	-7.813033	-1.270705	-3.392266
C	-6.712967	-2.054552	-3.790548
H	-8.458765	0.741057	-2.924494
H	-6.255536	1.751177	-3.228785
H	-6.843578	-3.121195	-3.958080
H	-4.636147	-2.122236	-4.266365
C	-9.153904	-1.860830	-3.275094
O	-3.721074	1.125024	-1.000526
O	-2.727773	-0.893622	-1.552845
F	-9.948619	-1.217863	-2.370231
F	-9.134607	-3.175837	-2.910820
F	-9.888897	-1.840807	-4.451812

20

TS(F3CBn—CO2−) r^{2}SCAN-3c SMD 2.60Å

C	-3.344506	0.095859	-1.442986
C	-3.954885	0.497739	-3.938168
C	-5.218125	-0.071581	-3.796785
H	-3.127759	-0.089591	-4.326786
H	-3.828880	1.575574	-3.887810
C	-5.458290	-1.479486	-3.984886
C	-6.375167	0.696445	-3.413291
C	-7.607429	0.120025	-3.216158
C	-7.798652	-1.268834	-3.379416
C	-6.694072	-2.047836	-3.785245
H	-8.447743	0.746930	-2.925503
H	-6.259389	1.768396	-3.265734
H	-6.818857	-3.117458	-3.939613
H	-4.624500	-2.111311	-4.284708
C	-9.131426	-1.861389	-3.250341
O	-3.753542	1.101106	-0.988175
O	-2.848283	-0.966434	-1.543496
F	-9.924750	-1.221255	-2.339202
F	-9.108157	-3.178533	-2.888581
F	-9.887559	-1.846799	-4.420773

20

TS(F3CBn—CO2−) r^{2}SCAN-3c SMD 2.75Å

C	-3.389230	0.056912	-1.358146
C	-3.960138	0.514473	-4.009033
C	-5.209623	-0.058954	-3.831823
H	-3.118649	-0.079422	-4.354383
H	-3.821278	1.587799	-3.915866
C	-5.450688	-1.472916	-4.004965
C	-6.368999	0.705292	-3.432089
C	-7.594953	0.125271	-3.216761
C	-7.786228	-1.266946	-3.369828
C	-6.681016	-2.042941	-3.786787
H	-8.433022	0.751115	-2.916666
H	-6.256097	1.778407	-3.289374
H	-6.802980	-3.114255	-3.932874
H	-4.618881	-2.104820	-4.310744
C	-9.113409	-1.860207	-3.229817
O	-3.802920	1.078454	-0.965938
O	-2.926669	-1.005932	-1.515885
F	-9.903768	-1.220838	-2.313468
F	-9.087436	-3.178722	-2.868728
F	-9.885988	-1.850204	-4.394479

20

TS(F3CBn—CO2−) r^{2}SCAN-3c SMD 2.90Å

C	-3.423706	0.016059	-1.270648
C	-3.965611	0.530529	-4.072729
C	-5.205632	-0.047178	-3.866113
H	-3.117904	-0.065513	-4.398952
H	-3.823133	1.602064	-3.963936
C	-5.444733	-1.464962	-4.024671
C	-6.364833	0.714277	-3.454575
C	-7.585615	0.131104	-3.222171
C	-7.774804	-1.263451	-3.364462
C	-6.670086	-2.037578	-3.789200
H	-8.422106	0.755927	-2.915287
H	-6.255380	1.788707	-3.318449
H	-6.789063	-3.110497	-3.926263
H	-4.614585	-2.097078	-4.334827
C	-9.097623	-1.859007	-3.210278
O	-3.846049	1.043018	-0.912940
O	-2.978263	-1.043895	-1.468016
F	-9.882818	-1.218602	-2.289518
F	-9.066333	-3.176852	-2.846044
F	-9.883694	-1.855507	-4.368577

20

TS(F3CBn—CO2−) r^{2}SCAN-3c SMD 3.05Å

C	-3.500963	-0.050662	-1.188551
C	-3.966420	0.552936	-4.141773
C	-5.198288	-0.029830	-3.907184
H	-3.114865	-0.044208	-4.456496

H	-3.823834	1.624432	-4.030271
C	-5.432545	-1.451086	-4.047899
C	-6.357214	0.729120	-3.487481
C	-7.570656	0.141441	-3.230993
C	-7.754069	-1.255980	-3.354934
C	-6.650705	-2.027944	-3.788126
H	-8.406135	0.765051	-2.918713
H	-6.252022	1.805582	-3.364276
H	-6.765002	-3.102995	-3.912130
H	-4.603140	-2.081776	-4.363029
C	-9.071392	-1.856004	-3.178691
O	-3.936391	0.974540	-0.846794
O	-3.056709	-1.103352	-1.416210
F	-9.847240	-1.213076	-2.251151
F	-9.029936	-3.171607	-2.806949
F	-9.874447	-1.863014	-4.326005

Geometry scan **TS(MeOOCBn--CO2−) r²SCAN-3c**:

23
TS(MeOOCBn—CO2−) r²SCAN-3c 1.40Å

C	-3.503062	-0.283886	-2.075172
C	-3.974411	-0.425580	-3.385803
C	-5.454481	-0.662690	-3.516384
H	-3.449275	-1.226664	-3.919742
H	-3.749346	0.510101	-3.923201
C	-5.936180	-1.862104	-4.056999
C	-6.391014	0.289405	-3.079751
C	-7.748265	0.053992	-3.202313
C	-8.222694	-1.146619	-3.756397
C	-7.295697	-2.109252	-4.177519
H	-8.472654	0.792205	-2.870996
H	-6.009518	1.192798	-2.615149
H	-7.645004	-3.045994	-4.598604
H	-5.221381	-2.614770	-4.381303
C	-9.672938	-1.340086	-3.871901
O	-4.033839	0.634136	-1.385188
O	-2.578841	-1.065675	-1.721575
O	-10.530552	-0.539267	-3.545727
O	-10.000787	-2.559122	-4.407252
C	-11.407180	-2.792173	-4.534491
H	-11.900945	-2.749195	-3.557923
H	-11.502318	-3.791005	-4.965771
H	-11.870315	-2.047082	-5.190183

23
TS(MeOOCBn—CO2−) r²SCAN-3c 1.55Å

C	-3.520079	-0.387732	-1.967391
C	-3.988877	-0.376279	-3.444752
C	-5.446388	-0.624890	-3.580861
H	-3.420814	-1.115046	-4.017466
H	-3.748997	0.625889	-3.822525
C	-5.929188	-1.847033	-4.075494
C	-6.389724	0.332859	-3.163317
C	-7.745262	0.086917	-3.264684
C	-8.218547	-1.132352	-3.779314
C	-7.286621	-2.103455	-4.175782
H	-8.470961	0.829824	-2.946958
H	-6.020113	1.257301	-2.731545
H	-7.634235	-3.054276	-4.565753
H	-5.212685	-2.605772	-4.381240
C	-9.665814	-1.334299	-3.880882
O	-4.047669	0.487767	-1.245735
O	-2.660672	-1.253038	-1.694849
O	-10.527014	-0.527907	-3.575063
O	-9.992245	-2.572130	-4.376454
C	-11.397796	-2.814049	-4.487857
H	-11.886823	-2.742716	-3.510437
H	-11.491852	-3.826300	-4.887232
H	-11.868323	-2.091806	-5.163754

23
TS(MeOOCBn—CO2−) r²SCAN-3c 1.70Å

C	-3.539462	-0.504317	-1.873977
C	-4.003568	-0.318332	-3.498790
C	-5.439846	-0.577373	-3.634508
H	-3.399589	-1.007814	-4.091710
H	-3.751804	0.721173	-3.726714
C	-5.920515	-1.822155	-4.086651
C	-6.395512	0.382942	-3.240180
C	-7.747854	0.122520	-3.322697
C	-8.216092	-1.115721	-3.797929
C	-7.274018	-2.090415	-4.169414
H	-8.477469	0.868637	-3.021554
H	-6.039185	1.329356	-2.844916
H	-7.615656	-3.055786	-4.528043
H	-5.199634	-2.584809	-4.371396
C	-9.658437	-1.331147	-3.886003
O	-3.982510	0.390367	-1.142090
O	-2.821842	-1.494424	-1.682472
O	-10.527498	-0.525399	-3.596028
O	-9.978358	-2.585469	-4.349595
C	-11.381760	-2.840183	-4.446959
H	-11.866579	-2.751319	-3.468682
H	-11.470784	-3.861863	-4.823312
H	-11.862727	-2.136993	-5.135722

23
TS(MeOOCBn—CO2−) r²SCAN-3c 1.85Å

C	-3.498909	-0.796515	-1.845778
C	-4.051779	-0.160997	-3.492882
C	-5.462039	-0.454716	-3.642218
H	-3.407578	-0.729715	-4.165501
H	-3.814037	0.902472	-3.480992
C	-5.909214	-1.748236	-4.000977
C	-6.456223	0.510224	-3.356449
C	-7.799463	0.212951	-3.446165
C	-8.229926	-1.072437	-3.823357
C	-7.251656	-2.050511	-4.089455
H	-8.551911	0.966796	-3.232478
H	-6.141058	1.507074	-3.057185
H	-7.562588	-3.052463	-4.367042
H	-5.163395	-2.516236	-4.184848
C	-9.660684	-1.328604	-3.922790
O	-3.222489	0.123862	-1.084663
O	-3.490466	-2.021825	-1.826225
O	-10.557396	-0.526921	-3.708243
O	-9.942836	-2.622146	-4.301850
C	-11.336864	-2.917168	-4.405316
H	-11.843057	-2.773751	-3.444249
H	-11.395183	-3.964355	-4.711576
H	-11.821949	-2.275309	-5.149106

23
TS(MeOOCBn—CO2−) r²SCAN-3c 2.00Å

C	-3.519061	-0.782234	-1.734677
C	-4.050014	-0.170214	-3.563207
C	-5.447010	-0.460105	-3.687294
H	-3.383006	-0.790337	-4.160179
H	-3.781971	0.884226	-3.538927
C	-5.908674	-1.748307	-4.066178
C	-6.443080	0.500150	-3.366184
C	-7.784771	0.202927	-3.436604
C	-8.227155	-1.076257	-3.830331

C	-7.250880	-2.047773	-4.134740
H	-8.532632	0.952788	-3.193887
H	-6.123420	1.491291	-3.053007
H	-7.566649	-3.043660	-4.428890
H	-5.169824	-2.513674	-4.288803
C	-9.655130	-1.329611	-3.907468
O	-3.409758	0.183982	-1.007041
O	-3.400201	-1.990385	-1.740577
O	-10.551309	-0.533976	-3.662092
O	-9.946189	-2.619110	-4.306013
C	-11.341270	-2.909635	-4.389514
H	-11.831030	-2.783946	-3.417246
H	-11.407757	-3.950995	-4.714412
H	-11.839907	-2.253670	-5.112074

23

TS(MeOOCBn—CO_2^-) r^2SCAN-3c 2.15Å

C	-3.517192	-0.808214	-1.641318
C	-4.057002	-0.155693	-3.617507
C	-5.440790	-0.449564	-3.718972
H	-3.368762	-0.799790	-4.159926
H	-3.769403	0.890436	-3.551926
C	-5.909303	-1.739933	-4.101016
C	-6.443525	0.507510	-3.385893
C	-7.782463	0.205774	-3.444511
C	-8.229288	-1.074489	-3.838334
C	-7.249637	-2.041069	-4.157155
H	-8.529699	0.953284	-3.191997
H	-6.125423	1.499782	-3.073928
H	-7.565153	-3.036507	-4.453768
H	-5.172589	-2.503298	-4.338743
C	-9.652871	-1.330610	-3.901590
O	-3.420291	0.180244	-0.960227
O	-3.419073	-2.006931	-1.683898
O	-10.552718	-0.540716	-3.643602
O	-9.945938	-2.622191	-4.304558
C	-11.340221	-2.913681	-4.375932
H	-11.822113	-2.794230	-3.398673
H	-11.409229	-3.953599	-4.705870
H	-11.848017	-2.255041	-5.090001

23

TS(MeOOCBn—CO_2^-) r^2SCAN-3c 2.30Å

C	-3.553342	-0.809954	-1.538215
C	-4.050499	-0.160483	-3.687872
C	-5.424490	-0.453406	-3.761394
H	-3.347319	-0.832224	-4.171893
H	-3.742388	0.876020	-3.584809
C	-5.906800	-1.740975	-4.154816
C	-6.427343	0.501314	-3.401573
C	-7.764929	0.200234	-3.443168
C	-8.223526	-1.075708	-3.845921
C	-7.246633	-2.038382	-4.193748
H	-8.506934	0.945325	-3.168344
H	-6.104372	1.489331	-3.081071
H	-7.567722	-3.029240	-4.500260
H	-5.176585	-2.502374	-4.419816
C	-9.643980	-1.328671	-3.888201
O	-3.687581	0.182082	-0.886900
O	-3.276477	-1.967740	-1.634602
O	-10.542707	-0.543089	-3.606961
O	-9.946693	-2.619010	-4.301671
C	-11.341503	-2.905528	-4.353771
H	-11.808980	-2.795741	-3.368104
H	-11.419653	-3.941865	-4.693850
H	-11.860243	-2.238437	-5.052386

23

TS(MeOOCBn—CO_2^-) r^2SCAN-3c 2.45Å

C	-3.583235	-0.852178	-1.445128
C	-4.050024	-0.140935	-3.742680
C	-5.414659	-0.438864	-3.795494
H	-3.333395	-0.826810	-4.184293
H	-3.729905	0.885796	-3.593464
C	-5.902380	-1.729784	-4.187727
C	-6.423277	0.513145	-3.426674
C	-7.758310	0.207332	-3.456497
C	-8.220275	-1.071035	-3.855366
C	-7.240271	-2.029534	-4.214388
H	-8.499841	0.950391	-3.174310
H	-6.102056	1.503333	-3.110142
H	-7.560906	-3.021115	-4.519696
H	-5.173716	-2.489426	-4.463076
C	-9.636352	-1.328206	-3.882087
O	-3.736297	0.155782	-0.839672
O	-3.322728	-1.999311	-1.593886
O	-10.538404	-0.547884	-3.590006
O	-9.940580	-2.622011	-4.294738
C	-11.334287	-2.911156	-4.331996
H	-11.792341	-2.804250	-3.341340
H	-11.414316	-3.947343	-4.672977
H	-11.863145	-2.244462	-5.023706

23

TS(MeOOCBn—CO_2^-) r^2SCAN-3c 2.60Å

C	-3.655134	-0.907656	-1.354901
C	-4.042664	-0.117407	-3.801394
C	-5.399305	-0.420365	-3.834469
H	-3.315677	-0.816398	-4.203467
H	-3.713136	0.898888	-3.608374
C	-5.891758	-1.714912	-4.224098
C	-6.412930	0.528308	-3.454783
C	-7.745411	0.216888	-3.470440
C	-8.210155	-1.064251	-3.864226
C	-7.227605	-2.018144	-4.236033
H	-8.486297	0.957439	-3.179476
H	-6.093522	1.520749	-3.142573
H	-7.547669	-3.010812	-4.538984
H	-5.164653	-2.472436	-4.510305
C	-9.621909	-1.326624	-3.872118
O	-3.831146	0.114217	-0.796050
O	-3.410163	-2.040928	-1.562361
O	-10.526995	-0.552317	-3.567726
O	-9.927270	-2.624621	-4.282505
C	-11.319346	-2.917777	-4.301653
H	-11.766105	-2.813056	-3.305341
H	-11.401286	-3.954307	-4.642070
H	-11.860562	-2.253004	-4.985996

23

TS(MeOOCBn—CO_2^-) r^2SCAN-3c 2.75Å

C	-3.783257	-0.971312	-1.273790
C	-4.026593	-0.091454	-3.867848
C	-5.376952	-0.400383	-3.878210
H	-3.292603	-0.801026	-4.237566
H	-3.689856	0.914822	-3.637753
C	-5.873740	-1.698101	-4.263932
C	-6.393461	0.543677	-3.482590
C	-7.723522	0.226502	-3.480586
C	-8.191080	-1.056714	-3.870047
C	-7.207577	-2.005498	-4.257997
H	-8.463017	0.963566	-3.177040
H	-6.074738	1.537330	-3.172797
H	-7.527645	-2.999120	-4.558292

H	-5.148579	-2.452965	-4.562738
C	-9.598904	-1.324860	-3.856021
O	-3.997298	0.060066	-0.760439
O	-3.533375	-2.085953	-1.535073
O	-10.505347	-0.556625	-3.535766
O	-9.905996	-2.625655	-4.265062
C	-11.296420	-2.923347	-4.263299
H	-11.728906	-2.822749	-3.260082
H	-11.380496	-3.959537	-4.604952
H	-11.851339	-2.259187	-4.937465

23
TS(MeOOCBn—CO2−) r²SCAN-3c 2.90Å

C	-3.943170	-1.042713	-1.202812
C	-4.006097	-0.063734	-3.931849
C	-5.352235	-0.378018	-3.923005
H	-3.269140	-0.778695	-4.285429
H	-3.665340	0.935335	-3.676858
C	-5.851725	-1.678039	-4.305450
C	-6.370110	0.561009	-3.511468
C	-7.697820	0.237737	-3.491090
C	-8.166883	-1.047532	-3.875722
C	-7.183412	-1.990724	-4.280712
H	-8.435953	0.970932	-3.174834
H	-6.051675	1.555622	-3.203983
H	-7.503248	-2.985339	-4.578276
H	-5.127975	-2.429456	-4.616641
C	-9.571159	-1.322653	-3.838337
O	-4.180318	-0.004061	-0.723949
O	-3.696733	-2.143965	-1.504545
O	-10.477807	-0.560419	-3.501698
O	-9.879044	-2.626056	-4.244079
C	-11.267354	-2.930204	-4.218574
H	-11.683424	-2.832314	-3.208052
H	-11.352582	-3.966716	-4.559414
H	-11.837496	-2.268522	-4.882567

23
TS(MeOOCBn—CO2−) r²SCAN-3c 3.05Å

C	-4.095303	-1.111273	-1.141405
C	-3.987721	-0.035731	-3.993445
C	-5.330262	-0.357124	-3.962088
H	-3.250652	-0.752079	-4.344163
H	-3.646145	0.960697	-3.729201
C	-5.830754	-1.660165	-4.337619
C	-6.348508	0.576678	-3.535375
C	-7.674142	0.247915	-3.498791
C	-8.143576	-1.039318	-3.878704
C	-7.160405	-1.978157	-4.296490
H	-8.411375	0.977369	-3.171839
H	-6.030992	1.572195	-3.229547
H	-7.479897	-2.974199	-4.589760
H	-5.108270	-2.409463	-4.657066
C	-9.545014	-1.320505	-3.822227
O	-4.338755	-0.066910	-0.683459
O	-3.856719	-2.203819	-1.472086
O	-10.451418	-0.562972	-3.472955
O	-9.852974	-2.626073	-4.224498
C	-11.239215	-2.935965	-4.179787
H	-11.642049	-2.839538	-3.163704
H	-11.324940	-3.973012	-4.519144
H	-11.821613	-2.277076	-4.835989

23
TS(MeOOCBn—CO2−) r²SCAN-3c SMD 1.40Å

C	-3.576306	-0.598404	-2.075573
C	-3.978960	-0.276614	-3.377234
C	-5.454260	-0.546981	-3.557231
H	-3.433016	-0.866871	-4.122908
H	-3.803221	0.785069	-3.592148
C	-5.902405	-1.797352	-3.999138
C	-6.407336	0.426028	-3.230063
C	-7.763101	0.160138	-3.344785
C	-8.204718	-1.093247	-3.787248
C	-7.258163	-2.072870	-4.113616
H	-8.497189	0.920689	-3.095700
H	-6.072640	1.399277	-2.882299
H	-7.582261	-3.048287	-4.460547
H	-5.174176	-2.561738	-4.257038
C	-9.662229	-1.325467	-3.897305
O	-3.758739	0.283137	-1.184055
O	-3.096208	-1.755369	-1.886040
O	-10.516605	-0.497891	-3.635899
O	-9.955519	-2.573049	-4.324161
C	-11.362203	-2.870401	-4.451667
H	-11.866669	-2.772071	-3.485830
H	-11.411584	-3.903277	-4.799401
H	-11.833191	-2.202974	-5.179457

23
TS(MeOOCBn—CO2−) r²SCAN-3c SMD 1.55Å

C	-3.571590	-0.643750	-1.979012
C	-3.991938	-0.259157	-3.420503
C	-5.450714	-0.526699	-3.599635
H	-3.412962	-0.852388	-4.135029
H	-3.786135	0.804802	-3.577104
C	-5.903534	-1.781223	-4.034858
C	-6.410068	0.443245	-3.269559
C	-7.764791	0.170999	-3.369717
C	-8.207561	-1.085952	-3.803068
C	-7.257925	-2.062172	-4.135090
H	-8.499321	0.929921	-3.116539
H	-6.078817	1.421107	-2.930592
H	-7.580821	-3.040235	-4.475937
H	-5.176007	-2.544923	-4.297690
C	-9.662877	-1.324845	-3.897361
O	-3.764746	0.235434	-1.104504
O	-3.104193	-1.799752	-1.835349
O	-10.520184	-0.501315	-3.630148
O	-9.955227	-2.576342	-4.317299
C	-11.361301	-2.880581	-4.428196
H	-11.855806	-2.782857	-3.457072
H	-11.409841	-3.914521	-4.773131
H	-11.844343	-2.217320	-5.151950

23
TS(MeOOCBn—CO2−) r²SCAN-3c SMD 1.70Å

C	-3.570354	-0.678037	-1.883884
C	-4.003172	-0.243294	-3.469339
C	-5.445663	-0.510595	-3.640260
H	-3.394857	-0.849298	-4.144871
H	-3.770933	0.819141	-3.578611
C	-5.904088	-1.768901	-4.070818
C	-6.411169	0.455580	-3.301859
C	-7.764306	0.177122	-3.386033
C	-8.209608	-1.082235	-3.813724
C	-7.256994	-2.054234	-4.155471
H	-8.498669	0.933902	-3.125487
H	-6.082274	1.436606	-2.968912
H	-7.579363	-3.033853	-4.492776
H	-5.177622	-2.531029	-4.341920
C	-9.661891	-1.326206	-3.892682
O	-3.767871	0.212984	-1.039759
O	-3.125248	-1.833867	-1.774353

O	-10.521952	-0.508104	-3.614006
O	-9.954875	-2.578992	-4.314041
C	-11.360394	-2.887134	-4.410710
H	-11.845286	-2.795599	-3.434043
H	-11.409770	-3.919798	-4.759679
H	-11.854339	-2.222683	-5.126107

23

TS(MeOOCBn—CO2−) r^2SCAN-3c SMD 1.85Å

C	-3.575511	-0.712887	-1.790677
C	-4.013324	-0.224819	-3.520593
C	-5.438983	-0.494083	-3.679221
H	-3.377387	-0.844938	-4.153938
H	-3.757530	0.834303	-3.579677
C	-5.903210	-1.756771	-4.106028
C	-6.411989	0.468353	-3.332535
C	-7.762639	0.183032	-3.401081
C	-8.210718	-1.079221	-3.823223
C	-7.254111	-2.046356	-4.175239
H	-8.497016	0.937457	-3.133131
H	-6.085876	1.452522	-3.005233
H	-7.575551	-3.027520	-4.509535
H	-5.177540	-2.516894	-4.385469
C	-9.658746	-1.328386	-3.886691
O	-3.752084	0.200861	-0.984408
O	-3.182605	-1.877569	-1.714616
O	-10.522314	-0.516669	-3.595289
O	-9.952754	-2.582503	-4.311180
C	-11.357429	-2.894032	-4.393436
H	-11.832405	-2.810328	-3.411055
H	-11.408296	-3.924817	-4.748174
H	-11.862682	-2.227261	-5.098914

23

TS(MeOOCBn—CO2−) r^2SCAN-3c SMD 2.00Å

C	-3.580161	-0.749632	-1.700400
C	-4.023409	-0.202283	-3.572282
C	-5.432303	-0.477041	-3.713202
H	-3.361860	-0.835922	-4.162279
H	-3.748368	0.851891	-3.578835
C	-5.901700	-1.745494	-4.135372
C	-6.414531	0.482128	-3.360830
C	-7.761836	0.189195	-3.415369
C	-8.212033	-1.077116	-3.830869
C	-7.250145	-2.039370	-4.190805
H	-8.496847	0.941338	-3.141995
H	-6.092331	1.470071	-3.039957
H	-7.569824	-3.022592	-4.521466
H	-5.176365	-2.503736	-4.421205
C	-9.654808	-1.331616	-3.880648
O	-3.694013	0.197078	-0.939949
O	-3.280368	-1.931173	-1.660407
O	-10.522821	-0.526756	-3.577258
O	-9.949695	-2.587278	-4.309516
C	-11.353214	-2.901620	-4.379310
H	-11.819246	-2.827733	-3.391673
H	-11.405765	-3.929758	-4.741969
H	-11.869055	-2.231108	-5.073746

23

TS(MeOOCBn—CO2−) r^2SCAN-3c SMD 2.15Å

C	-3.598632	-0.786557	-1.610584
C	-4.028662	-0.184231	-3.629192
C	-5.421885	-0.463272	-3.747770
H	-3.344618	-0.835433	-4.169754
H	-3.734681	0.862444	-3.582117
C	-5.899198	-1.736581	-4.167339
C	-6.412073	0.493630	-3.386984
C	-7.756055	0.195338	-3.428120
C	-8.211369	-1.073885	-3.838907
C	-7.245169	-2.032297	-4.208878
H	-8.490338	0.945955	-3.147695
H	-6.091192	1.483431	-3.069710
H	-7.564697	-3.016684	-4.537067
H	-5.175449	-2.493886	-4.460784
C	-9.648193	-1.331460	-3.874302
O	-3.755228	0.170334	-0.890272
O	-3.293724	-1.955279	-1.624816
O	-10.520270	-0.531852	-3.561221
O	-9.946105	-2.590304	-4.303777
C	-11.348583	-2.905442	-4.359977
H	-11.806264	-2.835356	-3.367932
H	-11.404547	-3.932810	-4.725019
H	-11.873768	-2.234329	-5.047127

23

TS(MeOOCBn—CO2−) r^2SCAN-3c SMD 2.30Å

C	-3.633710	-0.829347	-1.522246
C	-4.030483	-0.163286	-3.687641
C	-5.409044	-0.447461	-3.781946
H	-3.326138	-0.832956	-4.174789
H	-3.718778	0.872570	-3.579828
C	-5.894366	-1.725903	-4.199725
C	-6.407983	0.506792	-3.411743
C	-7.748298	0.202850	-3.440422
C	-8.208671	-1.069496	-3.847215
C	-7.237548	-2.023621	-4.227734
H	-8.482047	0.951481	-3.152316
H	-6.088802	1.498189	-3.096715
H	-7.556578	-3.009257	-4.553609
H	-5.172118	-2.482075	-4.500715
C	-9.638794	-1.330867	-3.867796
O	-3.808569	0.147612	-0.854607
O	-3.349402	-1.988759	-1.597474
O	-10.515884	-0.536872	-3.545793
O	-9.939268	-2.594140	-4.296208
C	-11.340230	-2.911076	-4.337747
H	-11.789329	-2.843285	-3.341357
H	-11.399102	-3.938456	-4.703127
H	-11.875556	-2.241161	-5.018592

23

TS(MeOOCBn—CO2−) r^2SCAN-3c SMD 2.45Å

C	-3.683182	-0.875481	-1.436000
C	-4.028690	-0.141634	-3.747837
C	-5.394562	-0.431558	-3.815315
H	-3.308100	-0.829484	-4.181612
H	-3.700924	0.879999	-3.576862
C	-5.887580	-1.715546	-4.229754
C	-6.401416	0.519637	-3.433525
C	-7.738253	0.210531	-3.450620
C	-8.203735	-1.064609	-3.854459
C	-7.228009	-2.015057	-4.244912
H	-8.471270	0.957008	-3.154121
H	-6.083687	1.512205	-3.119697
H	-7.546717	-3.002166	-4.567583
H	-5.166645	-2.470842	-4.537104
C	-9.627272	-1.329370	-3.860783
O	-3.882673	0.123435	-0.831035
O	-3.412355	-2.019702	-1.580915
O	-10.509285	-0.540792	-3.530216
O	-9.930292	-2.597426	-4.287679
C	-11.329546	-2.916108	-4.314906
H	-11.770631	-2.849334	-3.314586

H	-11.391272	-3.943964	-4.679354
H	-11.874602	-2.248264	-4.990469

23

TS(MeOOCBn—CO2−) r²SCAN-3c SMD 2.60Å

C	-3.786425	-0.936585	-1.351539
C	-4.017866	-0.117578	-3.808297
C	-5.373310	-0.411381	-3.855215
H	-3.285242	-0.823804	-4.188576
H	-3.676784	0.885829	-3.570141
C	-5.873495	-1.699191	-4.269583
C	-6.386684	0.536182	-3.459843
C	-7.720365	0.222146	-3.465019
C	-8.190577	-1.055258	-3.866961
C	-7.211364	-2.001019	-4.271007
H	-8.452256	0.965652	-3.157528
H	-6.070037	1.528948	-3.144732
H	-7.529961	-2.989054	-4.591820
H	-5.154495	-2.452824	-4.586302
C	-9.607716	-1.325507	-3.852722
O	-4.013022	0.083245	-0.814457
O	-3.525275	-2.060398	-1.572133
O	-10.493724	-0.543281	-3.510212
O	-9.912806	-2.599459	-4.274381
C	-11.309435	-2.923828	-4.276920
H	-11.735472	-2.856741	-3.269830
H	-11.373642	-3.953263	-4.637311
H	-11.870746	-2.261357	-4.944814

23

TS(MeOOCBn—CO2−) r²SCAN-3c SMD 2.75Å

C	-3.902771	-0.996905	-1.270258
C	-4.003621	-0.094558	-3.866043
C	-5.353076	-0.392701	-3.894974
H	-3.266608	-0.811102	-4.217795
H	-3.656576	0.896619	-3.588048
C	-5.857905	-1.682571	-4.308390
C	-6.369168	0.550653	-3.484957
C	-7.700676	0.232583	-3.477700
C	-8.174268	-1.046066	-3.878311
C	-7.193896	-1.987373	-4.296108
H	-8.430958	0.972898	-3.158347
H	-6.052441	1.542756	-3.167375
H	-7.512586	-2.976158	-4.615032
H	-5.140587	-2.434403	-4.633701
C	-9.586822	-1.322024	-3.843233
O	-4.152437	0.036268	-0.784184
O	-3.645959	-2.102788	-1.547917
O	-10.474285	-0.545495	-3.487579
O	-9.893417	-2.599728	-4.261354
C	-11.287386	-2.930540	-4.238942
H	-11.696706	-2.865573	-3.224615
H	-11.353532	-3.960584	-4.597772
H	-11.865016	-2.271734	-4.896711

23

TS(MeOOCBn—CO2−) r²SCAN-3c SMD 2.90Å

C	-3.981062	-1.048324	-1.193768
C	-3.995237	-0.070909	-3.924053
C	-5.341192	-0.377024	-3.927989
H	-3.256257	-0.793309	-4.259960
H	-3.645694	0.913051	-3.623628
C	-5.847541	-1.670216	-4.334556
C	-6.358241	0.561940	-3.505160
C	-7.688087	0.239559	-3.485818
C	-8.162496	-1.040418	-3.883604
C	-7.181952	-1.978865	-4.309793
H	-8.417697	0.976777	-3.157676
H	-6.041480	1.554234	-3.187839
H	-7.500525	-2.969225	-4.624131
H	-5.130733	-2.420454	-4.664953
C	-9.572706	-1.320674	-3.835282
O	-4.236135	-0.004994	-0.739657
O	-3.732082	-2.143951	-1.505993
O	-10.460291	-0.547332	-3.471092
O	-9.879713	-2.599956	-4.250876
C	-11.272239	-2.934710	-4.215291
H	-11.672354	-2.870829	-3.197186
H	-11.338807	-3.965054	-4.573347
H	-11.858177	-2.277841	-4.867694

23

TS(MeOOCBn—CO2−) r²SCAN-3c SMD 3.05Å

C	-4.052894	-1.094162	-1.116257
C	-3.988263	-0.048993	-3.980859
C	-5.331231	-0.363553	-3.959768
H	-3.249128	-0.771473	-4.316907
H	-3.637968	0.933560	-3.676227
C	-5.838167	-1.659764	-4.358239
C	-6.347630	0.570488	-3.522407
C	-7.676018	0.244071	-3.490357
C	-8.151123	-1.036790	-3.885807
C	-7.171142	-1.972422	-4.320718
H	-8.404310	0.978501	-3.153087
H	-6.030983	1.563019	-3.205485
H	-7.489368	-2.964189	-4.631018
H	-5.122565	-2.408546	-4.694720
C	-9.559603	-1.320371	-3.826186
O	-4.306229	-0.042331	-0.684773
O	-3.812666	-2.184571	-1.448211
O	-10.446650	-0.549910	-3.453804
O	-9.867271	-2.600090	-4.241794
C	-11.258722	-2.937328	-4.196810
H	-11.651877	-2.876184	-3.175798
H	-11.326193	-3.967120	-4.556396
H	-11.850697	-2.280369	-4.843715

Geometry scan **TS(O2NBn--CO2−) r²SCAN-3c**:

19

TS(O2NBn—CO2−) r²SCAN-3c 1.40Å

C	-3.613233	-0.371073	-2.124891
C	-4.121837	-0.513552	-3.421434
C	-5.598760	-0.762113	-3.518902
H	-3.597318	-1.298993	-3.977723
H	-3.913862	0.430209	-3.951701
C	-6.079607	-1.961912	-4.063200
C	-6.533687	0.175598	-3.045697
C	-7.892531	-0.065755	-3.136743
C	-8.338565	-1.266707	-3.696308
C	-7.436997	-2.224529	-4.156804
H	-8.618712	0.656863	-2.783582
H	-6.151844	1.077476	-2.579983
H	-7.810073	-3.149846	-4.579436
H	-5.365942	-2.702819	-4.414542
N	-9.763888	-1.520639	-3.809180
O	-4.199985	0.456268	-1.373283
O	-2.592648	-1.059479	-1.853402
O	-10.547158	-0.656013	-3.403200
O	-10.132561	-2.589559	-4.309386

19

TS(O2NBn—CO2−) r²SCAN-3c 1.55Å

C	-3.627959	-0.459217	-2.036808

C	-4.137919	-0.446754	-3.500463
C	-5.589875	-0.721561	-3.586448
H	-3.573228	-1.170304	-4.094229
H	-3.925645	0.563675	-3.870965
C	-6.065997	-1.953571	-4.067958
C	-6.534124	0.218201	-3.127601
C	-7.888800	-0.044973	-3.180185
C	-8.329542	-1.271574	-3.688485
C	-7.419073	-2.235634	-4.125159
H	-8.618608	0.677854	-2.834917
H	-6.165511	1.146608	-2.704251
H	-7.787029	-3.181450	-4.504850
H	-5.347595	-2.697982	-4.401327
N	-9.748396	-1.545049	-3.769252
O	-4.142753	0.408199	-1.300127
O	-2.755863	-1.320360	-1.800559
O	-10.538670	-0.672041	-3.390556
O	-10.112619	-2.640643	-4.215256

19

TS(O2NBn—CO2−) r²SCAN-3c 1.70Å

C	-3.630970	-0.558988	-1.958642
C	-4.156961	-0.380101	-3.565295
C	-5.585478	-0.675750	-3.637147
H	-3.557328	-1.054634	-4.178148
H	-3.937346	0.666132	-3.792070
C	-6.052182	-1.937902	-4.061414
C	-6.546205	0.264532	-3.203388
C	-7.894859	-0.021969	-3.226696
C	-8.324480	-1.274621	-3.682044
C	-7.398588	-2.240506	-4.090170
H	-8.632076	0.701910	-2.899635
H	-6.196166	1.220576	-2.826914
H	-7.755624	-3.206453	-4.427578
H	-5.325562	-2.684312	-4.371449
N	-9.733750	-1.570673	-3.737076
O	-4.048455	0.339410	-1.220139
O	-2.908768	-1.548880	-1.800635
O	-10.536317	-0.695813	-3.381767
O	-10.088093	-2.688533	-4.139190

19

TS(O2NBn—CO2−) r²SCAN-3c 1.85Å

C	-3.620762	-0.698355	-1.899421
C	-4.183499	-0.285571	-3.612732
C	-5.587298	-0.610557	-3.670409
H	-3.548733	-0.909309	-4.240990
H	-3.961517	0.778019	-3.693260
C	-6.030383	-1.907294	-4.031244
C	-6.578069	0.326117	-3.284278
C	-7.917342	0.006193	-3.288009
C	-8.320599	-1.278884	-3.677288
C	-7.366556	-2.240102	-4.039479
H	-8.671538	0.728909	-2.998909
H	-6.259084	1.315951	-2.969844
H	-7.700303	-3.231119	-4.324445
H	-5.285963	-2.652112	-4.299028
N	-9.715932	-1.609510	-3.712028
O	-3.770763	0.259993	-1.154912
O	-3.201990	-1.846205	-1.848896
O	-10.541454	-0.739319	-3.390023
O	-10.047423	-2.753420	-4.064201

19

TS(O2NBn—CO2−) r²SCAN-3c 2.00Å

C	-3.611044	-0.725725	-1.813536
C	-4.197117	-0.269734	-3.670574
C	-5.584106	-0.598308	-3.704111
H	-3.535738	-0.908290	-4.251878
H	-3.950734	0.789498	-3.696835
C	-6.033121	-1.900963	-4.057647
C	-6.581726	0.336549	-3.311556
C	-7.916918	0.009991	-3.297813
C	-8.323760	-1.279941	-3.675573
C	-7.365936	-2.237268	-4.047247
H	-8.670674	0.731190	-3.003330
H	-6.265882	1.330988	-3.007873
H	-7.699147	-3.230600	-4.325226
H	-5.290847	-2.644614	-4.335397
N	-9.711645	-1.615681	-3.689428
O	-3.783944	0.243846	-1.108914
O	-3.201113	-1.865356	-1.810717
O	-10.540213	-0.747502	-3.359892
O	-10.045538	-2.764655	-4.031851

19

TS(O2NBn—CO2−) r²SCAN-3c 2.15Å

C	-3.607229	-0.758046	-1.728568
C	-4.208725	-0.249276	-3.729032
C	-5.580208	-0.584105	-3.735997
H	-3.523712	-0.901662	-4.263364
H	-3.941631	0.803446	-3.699740
C	-6.033141	-1.893428	-4.081172
C	-6.585357	0.348427	-3.338046
C	-7.916161	0.014326	-3.308138
C	-8.324813	-1.281161	-3.673709
C	-7.362277	-2.234210	-4.053309
H	-8.670408	0.733522	-3.009527
H	-6.273597	1.347876	-3.045785
H	-7.693839	-3.230403	-4.323480
H	-5.291987	-2.635447	-4.367041
N	-9.704809	-1.623193	-3.667477
O	-3.780477	0.229924	-1.069357
O	-3.233700	-1.897848	-1.774711
O	-10.537420	-0.757491	-3.331375
O	-10.039714	-2.777826	-3.999571

19

TS(O2NBn—CO2−) r²SCAN-3c 2.30Å

C	-3.613859	-0.793018	-1.642942
C	-4.216744	-0.227507	-3.789271
C	-5.574656	-0.569450	-3.768652
H	-3.511851	-0.893705	-4.277051
H	-3.932951	0.817402	-3.709995
C	-6.031123	-1.885389	-4.104644
C	-6.586710	0.360442	-3.364106
C	-7.913092	0.018888	-3.317240
C	-8.323281	-1.282140	-3.670331
C	-7.356474	-2.230870	-4.058621
H	-8.667636	0.735915	-3.013695
H	-6.278851	1.364657	-3.083100
H	-7.686335	-3.229970	-4.320610
H	-5.291178	-2.625669	-4.399033
N	-9.695309	-1.630707	-3.642692
O	-3.791408	0.213257	-1.032067
O	-3.275010	-1.929893	-1.742553
O	-10.531683	-0.767709	-3.298925
O	-10.031057	-2.791111	-3.963868

19

TS(O2NBn—CO2−) r²SCAN-3c 2.45Å

C	-3.637670	-0.828790	-1.554460
C	-4.219792	-0.207418	-3.851747
C	-5.566501	-0.555722	-3.804225

H	-3.499765	-0.886369	-4.297406
H	-3.922631	0.829429	-3.730296
C	-6.026716	-1.877346	-4.131064
C	-6.583050	0.371627	-3.389868
C	-7.905273	0.023519	-3.323870
C	-8.317502	-1.282263	-3.665224
C	-7.348494	-2.227049	-4.065067
H	-8.658992	0.738275	-3.012596
H	-6.277632	1.379546	-3.118566
H	-7.677613	-3.228554	-4.319221
H	-5.288845	-2.615910	-4.435780
N	-9.682231	-1.636883	-3.613612
O	-3.840179	0.191804	-0.993984
O	-3.316228	-1.955581	-1.708690
O	-10.520661	-0.776525	-3.259566
O	-10.019430	-2.802364	-3.924156

19

TS(O2NBn—CO2−) r^2SCAN-3c 2.60Å

C	-3.692331	-0.869826	-1.465939
C	-4.216720	-0.186162	-3.919023
C	-5.553879	-0.540543	-3.844262
H	-3.486170	-0.876142	-4.329163
H	-3.909281	0.842879	-3.761832
C	-6.017499	-1.867604	-4.160305
C	-6.573170	0.383616	-3.416869
C	-7.890862	0.028617	-3.327721
C	-8.304990	-1.281826	-3.656242
C	-7.335223	-2.222323	-4.070537
H	-8.642887	0.740755	-3.006129
H	-6.269785	1.395024	-3.155452
H	-7.663733	-3.226370	-4.315820
H	-5.282346	-2.604174	-4.477098
N	-9.662259	-1.643179	-3.576543
O	-3.928017	0.162516	-0.957749
O	-3.377905	-1.982249	-1.674014
O	-10.501417	-0.785949	-3.209354
O	-10.000734	-2.813637	-3.875346

19

TS(O2NBn—CO2−) r^2SCAN-3c 2.75Å

C	-3.783297	-0.921564	-1.384502
C	-4.208815	-0.158698	-3.992081
C	-5.537581	-0.521246	-3.887652
H	-3.471222	-0.856026	-4.376856
H	-3.895177	0.864376	-3.809178
C	-6.001355	-1.854528	-4.188623
C	-6.558457	0.398552	-3.446140
C	-7.870858	0.034858	-3.330041
C	-8.283863	-1.281074	-3.642670
C	-7.314033	-2.216817	-4.071347
H	-8.621444	0.743574	-2.997493
H	-6.258045	1.413951	-3.196262
H	-7.640383	-3.224576	-4.304318
H	-5.268067	-2.588596	-4.515942
N	-9.633788	-1.651472	-3.531723
O	-4.029845	0.123190	-0.919965
O	-3.488756	-2.024893	-1.637416
O	-10.473177	-0.798154	-3.151314
O	-9.971044	-2.827430	-3.815872

19

TS(O2NBn—CO2−) r^2SCAN-3c 2.90Å

C	-3.886603	-0.974480	-1.308346
C	-4.199416	-0.130936	-4.065261
C	-5.520960	-0.501980	-3.930821
H	-3.458891	-0.831701	-4.438428
H	-3.883498	0.889738	-3.872285
C	-5.983251	-1.840871	-4.215856
C	-6.541611	0.413305	-3.474735
C	-7.848324	0.041054	-3.330538
C	-8.259121	-1.280060	-3.627047
C	-7.290337	-2.211189	-4.070297
H	-8.597086	0.746371	-2.986719
H	-6.243517	1.432272	-3.236370
H	-7.614114	-3.222621	-4.290819
H	-5.251463	-2.571901	-4.553507
N	-9.601953	-1.659464	-3.485102
O	-4.142236	0.077643	-0.873172
O	-3.609144	-2.071426	-1.593453
O	-10.440613	-0.809983	-3.091817
O	-9.937068	-2.840346	-3.754824

19

TS(O2NBn—CO2−) r^2SCAN-3c 3.05Å

C	-3.995673	-1.027512	-1.237565
C	-4.190201	-0.103588	-4.137741
C	-5.504520	-0.483504	-3.971593
H	-3.449903	-0.804788	-4.510746
H	-3.874947	0.917573	-3.945911
C	-5.964152	-1.827739	-4.239696
C	-6.523659	0.427110	-3.500220
C	-7.824622	0.047012	-3.329336
C	-8.232756	-1.278662	-3.610928
C	-7.265506	-2.205710	-4.067372
H	-8.571578	0.748956	-2.974765
H	-6.228537	1.449287	-3.271832
H	-7.586752	-3.220552	-4.275742
H	-5.234323	-2.556673	-4.586183
N	-9.568926	-1.666232	-3.440590
O	-4.258272	0.029257	-0.822201
O	-3.727072	-2.119251	-1.544321
O	-10.406348	-0.820169	-3.035747
O	-9.901460	-2.851390	-3.696907

19

TS(O2NBn—CO2−) r^2SCAN-3c SMD 1.40Å

C	-3.677745	-0.592899	-2.141417
C	-4.121806	-0.357093	-3.448018
C	-5.591607	-0.664264	-3.564914
H	-3.585100	-0.977420	-4.174493
H	-3.974240	0.694941	-3.723515
C	-6.024163	-1.923086	-4.003207
C	-6.552146	0.281347	-3.177237
C	-7.904689	-0.010605	-3.232881
C	-8.301745	-1.271469	-3.677856
C	-7.373153	-2.236059	-4.063211
H	-8.647507	0.722735	-2.941031
H	-6.224071	1.255532	-2.828386
H	-7.706569	-3.208462	-4.406410
H	-5.289805	-2.664628	-4.304742
N	-9.724334	-1.584993	-3.750489
O	-3.899097	0.318108	-1.290927
O	-3.124724	-1.707705	-1.911732
O	-10.530711	-0.716468	-3.413790
O	-10.055996	-2.704086	-4.145141

19

TS(O2NBn—CO2−) r^2SCAN-3c SMD 1.55Å

C	-3.658774	-0.655618	-2.062377
C	-4.142254	-0.323200	-3.497035
C	-5.592787	-0.636865	-3.604104
H	-3.573574	-0.919286	-4.216259
H	-3.973436	0.741899	-3.683808

C	-6.024025	-1.911603	-4.008755
C	-6.563587	0.308126	-3.230131
C	-7.912665	0.002799	-3.264613
C	-8.305653	-1.271686	-3.676038
C	-7.368797	-2.236541	-4.047561
H	-8.658797	0.736700	-2.982578
H	-6.245191	1.296079	-2.910962
H	-7.696839	-3.219484	-4.365219
H	-5.286705	-2.654286	-4.300409
N	-9.721433	-1.597385	-3.728793
O	-3.866712	0.235441	-1.205995
O	-3.134402	-1.784400	-1.915172
O	-10.534736	-0.727827	-3.405229
O	-10.048837	-2.729439	-4.094359

19

TS(O2NBn—CO2−) r^2SCAN-3c SMD 1.70Å

C	-3.648860	-0.687883	-1.975751
C	-4.159223	-0.303448	-3.551105
C	-5.590919	-0.620418	-3.643953
H	-3.558308	-0.907568	-4.233525
H	-3.964153	0.762875	-3.684444
C	-6.026255	-1.902697	-4.035633
C	-6.569029	0.321022	-3.263940
C	-7.914275	0.006766	-3.277206
C	-8.309273	-1.273625	-3.674927
C	-7.367489	-2.234235	-4.053455
H	-8.660730	0.738308	-2.989578
H	-6.254585	1.314524	-2.957245
H	-7.693895	-3.220891	-4.361525
H	-5.289390	-2.643651	-4.333473
N	-9.717178	-1.606325	-3.706154
O	-3.843880	0.218132	-1.150930
O	-3.161494	-1.824067	-1.868966
O	-10.534551	-0.738946	-3.377610
O	-10.045719	-2.744447	-4.059977

19

TS(O2NBn—CO2−) r^2SCAN-3c SMD 1.85Å

C	-3.641370	-0.724634	-1.892065
C	-4.176557	-0.277760	-3.605651
C	-5.588332	-0.601150	-3.680113
H	-3.544556	-0.890190	-4.248906
H	-3.958420	0.787662	-3.680908
C	-6.025839	-1.892496	-4.059046
C	-6.576653	0.336018	-3.296171
C	-7.916737	0.010785	-3.290033
C	-8.311733	-1.276852	-3.673350
C	-7.362443	-2.231884	-4.057531
H	-8.665020	0.739056	-2.998445
H	-6.268459	1.335697	-3.002158
H	-7.685348	-3.222863	-4.355757
H	-5.287913	-2.630665	-4.361463
N	-9.709652	-1.618222	-3.684768
O	-3.775126	0.213312	-1.110852
O	-3.243669	-1.884481	-1.824129
O	-10.533509	-0.754790	-3.350090
O	-10.037871	-2.763118	-4.027960

19

TS(O2NBn—CO2−) r^2SCAN-3c SMD 2.00Å

C	-3.647869	-0.756802	-1.807299
C	-4.189214	-0.260386	-3.667546
C	-5.581736	-0.586520	-3.718207
H	-3.528026	-0.889753	-4.260309
H	-3.944096	0.800043	-3.686430
C	-6.025942	-1.885248	-4.087552
C	-6.577418	0.348595	-3.325413
C	-7.912416	0.015992	-3.299023
C	-8.312303	-1.277470	-3.669813
C	-7.358254	-2.228526	-4.064482
H	-8.660124	0.742236	-3.000417
H	-6.271119	1.351853	-3.040951
H	-7.680825	-3.222374	-4.353988
H	-5.289705	-2.622149	-4.398017
N	-9.699491	-1.623708	-3.657922
O	-3.821072	0.189747	-1.065490
O	-3.250377	-1.905006	-1.791477
O	-10.527656	-0.762374	-3.314144
O	-10.031564	-2.774724	-3.990918

19

TS(O2NBn—CO2−) r^2SCAN-3c SMD 2.15Å

C	-3.665090	-0.792261	-1.724164
C	-4.199469	-0.237890	-3.731554
C	-5.573067	-0.570006	-3.754185
H	-3.511113	-0.885026	-4.269308
H	-3.930702	0.814696	-3.690243
C	-6.022501	-1.877012	-4.114135
C	-6.577620	0.362201	-3.352452
C	-7.906708	0.020869	-3.306689
C	-8.310066	-1.279198	-3.665038
C	-7.349816	-2.225223	-4.070274
H	-8.654733	0.744135	-3.001116
H	-6.274526	1.369302	-3.077173
H	-7.670861	-3.222253	-4.351109
H	-5.287099	-2.611750	-4.432360
N	-9.685239	-1.631678	-3.630152
O	-3.838665	0.178608	-1.036751
O	-3.312628	-1.941017	-1.763071
O	-10.519180	-0.773631	-3.276065
O	-10.020123	-2.789442	-3.953557

19

TS(O2NBn—CO2−) r^2SCAN-3c SMD 2.30Å

C	-3.688446	-0.826808	-1.640196
C	-4.206686	-0.216918	-3.796457
C	-5.563152	-0.555648	-3.787954
H	-3.494987	-0.883005	-4.275783
H	-3.917001	0.825057	-3.694917
C	-6.018376	-1.870611	-4.138865
C	-6.575431	0.374038	-3.375407
C	-7.898948	0.025448	-3.311753
C	-8.306339	-1.280287	-3.660005
C	-7.341066	-2.222284	-4.075917
H	-8.646696	0.745850	-2.998249
H	-6.274595	1.383990	-3.107118
H	-7.661504	-3.221914	-4.348799
H	-5.284454	-2.603863	-4.464713
N	-9.670285	-1.637464	-3.604139
O	-3.877767	0.168466	-1.016308
O	-3.365598	-1.967284	-1.743730
O	-10.509060	-0.782342	-3.238540
O	-10.008816	-2.800996	-3.920547

19

TS(O2NBn—CO2−) r^2SCAN-3c SMD 2.45Å

C	-3.724861	-0.864065	-1.554058
C	-4.209284	-0.196874	-3.861154
C	-5.551123	-0.542099	-3.822406
H	-3.480097	-0.879632	-4.286847
H	-3.903220	0.833204	-3.704879
C	-6.012259	-1.864556	-4.162739
C	-6.569406	0.385043	-3.397351

C	-7.887736	0.030438	-3.315281
C	-8.299625	-1.280409	-3.653684
C	-7.330586	-2.219156	-4.080474
H	-8.634216	0.748395	-2.992726
H	-6.269889	1.397291	-3.135218
H	-7.650822	-3.221430	-4.344467
H	-5.280386	-2.597081	-4.495717
N	-9.653047	-1.641657	-3.575984
O	-3.951595	0.151176	-1.000375
O	-3.410102	-1.985541	-1.732904
O	-10.495294	-0.789130	-3.197729
O	-9.995660	-2.810491	-3.885404

19
TS(O2NBn—CO2−) r²SCAN-3c SMD 2.60Å

C	-3.748109	-0.898433	-1.465673
C	-4.212284	-0.179528	-3.920814
C	-5.543858	-0.530870	-3.853761
H	-3.473942	-0.873605	-4.310918
H	-3.895760	0.841024	-3.727033
C	-6.008616	-1.859194	-4.183124
C	-6.564987	0.393516	-3.416134
C	-7.879469	0.034478	-3.318511
C	-8.294519	-1.279857	-3.649695
C	-7.323556	-2.216516	-4.084793
H	-8.624348	0.750346	-2.987365
H	-6.266067	1.407191	-3.158010
H	-7.643442	-3.221184	-4.340474
H	-5.278279	-2.591394	-4.520934
N	-9.640641	-1.644234	-3.554819
O	-4.012475	0.130135	-0.972797
O	-3.428242	-1.998087	-1.709246
O	-10.484587	-0.793539	-3.166736
O	-9.986027	-2.816823	-3.858561

19
TS(O2NBn—CO2−) r²SCAN-3c SMD 2.75Å

C	-3.785451	-0.942026	-1.382425
C	-4.212905	-0.155964	-3.982788
C	-5.537086	-0.515693	-3.888296
H	-3.468911	-0.856774	-4.350025
H	-3.892561	0.859330	-3.767572
C	-6.001248	-1.849929	-4.203242
C	-6.559770	0.405239	-3.440368
C	-7.870222	0.039845	-3.323920
C	-8.284228	-1.278911	-3.643410
C	-7.312334	-2.212949	-4.085717
H	-8.614280	0.753225	-2.985505
H	-6.262843	1.421559	-3.189853
H	-7.630257	-3.221079	-4.330217
H	-5.271441	-2.580954	-4.545076
N	-9.624769	-1.649373	-3.528710
O	-4.064673	0.095513	-0.926787
O	-3.477335	-2.030351	-1.670091
O	-10.469662	-0.801008	-3.133219
O	-9.969229	-2.826274	-3.822177

Geometry scan **TS(NCBn--CO2−) r²SCAN-3c**:

18
TS(NCBn—CO2−) r²SCAN-3c 1.40Å

C	-3.372074	0.317903	-2.423638
C	-4.306478	0.898158	-3.289775
C	-5.610632	0.166074	-3.448619
H	-3.837257	0.942517	-4.286848
H	-4.521611	1.936755	-3.011506
C	-5.656275	-1.147159	-3.946079
C	-6.815462	0.774029	-3.074390
C	-8.030244	0.116014	-3.196396
C	-8.064075	-1.189484	-3.706883
C	-6.859860	-1.815809	-4.078610
H	-8.956779	0.599505	-2.901237
H	-6.792783	1.784193	-2.672779
H	-6.888345	-2.829262	-4.468264
H	-4.713362	-1.627145	-4.185194
C	-9.303823	-1.866639	-3.853675
O	-2.812242	1.095855	-1.605717
O	-3.150504	-0.918417	-2.576302
N	-10.319398	-2.416299	-3.977380

18
TS(NCBn—CO2−) r²SCAN-3c 1.55Å

C	-3.380054	0.285791	-2.317245
C	-4.317533	0.914693	-3.379373
C	-5.611260	0.198859	-3.511667
H	-3.763345	0.849316	-4.324988
H	-4.484401	1.970358	-3.147085
C	-5.662785	-1.112867	-4.019740
C	-6.811615	0.783276	-3.078595
C	-8.018995	0.110275	-3.163641
C	-8.058296	-1.190348	-3.691271
C	-6.859697	-1.796689	-4.112742
H	-8.940210	0.578613	-2.829602
H	-6.786260	1.788235	-2.664552
H	-6.888641	-2.807469	-4.509323
H	-4.728389	-1.584599	-4.304642
C	-9.293404	-1.878960	-3.806636
O	-2.963899	1.081813	-1.449803
O	-3.136037	-0.930084	-2.486953
N	-10.306379	-2.439425	-3.905435

18
TS(NCBn—CO2−) r²SCAN-3c 1.70Å

C	-3.391842	0.266340	-2.205703
C	-4.325757	0.924376	-3.464588
C	-5.606459	0.220154	-3.572324
H	-3.699051	0.766920	-4.347527
H	-4.450751	1.990072	-3.264397
C	-5.672689	-1.088467	-4.095864
C	-6.799910	0.785663	-3.083147
C	-8.001033	0.103496	-3.133407
C	-8.053777	-1.191690	-3.678990
C	-6.864973	-1.780966	-4.152097
H	-8.912804	0.560141	-2.759131
H	-6.767334	1.784338	-2.654742
H	-6.899359	-2.787182	-4.559968
H	-4.751161	-1.554268	-4.429693
C	-9.285717	-1.886854	-3.760896
O	-3.121190	1.088540	-1.321917
O	-3.109462	-0.925561	-2.386419
N	-10.297937	-2.454262	-3.832481

18
TS(NCBn—CO2−) r²SCAN-3c 1.85Å

C	-3.404107	0.254911	-2.095701
C	-4.331576	0.928822	-3.547647
C	-5.598138	0.232263	-3.626572
H	-3.646630	0.696038	-4.364366
H	-4.418428	1.998977	-3.362053
C	-5.684804	-1.072963	-4.166649
C	-6.783457	0.784653	-3.088417
C	-7.979615	0.097876	-3.107194
C	-8.050808	-1.192429	-3.668276
C	-6.874403	-1.768597	-4.189796

H	-8.879347	0.546388	-2.695225
H	-6.740234	1.776933	-2.646392
H	-6.918231	-2.769223	-4.610754
H	-4.778112	-1.534043	-4.546494
C	-9.280604	-1.890119	-3.717726
O	-3.301464	1.092771	-1.207351
O	-3.048263	-0.899729	-2.299790
N	-10.292982	-2.461742	-3.762890

18

TS(NCBn—CO2−) r^{2}SCAN-3c 2.00Å

C	-3.399186	0.240531	-1.996551
C	-4.341626	0.935520	-3.617907
C	-5.592666	0.241166	-3.667894
H	-3.613813	0.655230	-4.377747
H	-4.397149	2.004295	-3.421721
C	-5.697025	-1.063504	-4.219315
C	-6.773664	0.784777	-3.097165
C	-7.965139	0.094585	-3.092576
C	-8.051258	-1.193993	-3.661250
C	-6.883952	-1.760321	-4.217476
H	-8.855163	0.538482	-2.654722
H	-6.723830	1.773656	-2.648133
H	-6.935459	-2.756959	-4.647507
H	-4.802649	-1.520425	-4.633350
C	-9.277931	-1.894008	-3.684620
O	-3.423409	1.088323	-1.127655
O	-2.987265	-0.877167	-2.229421
N	-10.290020	-2.469400	-3.708282

18

TS(NCBn—CO2−) r^{2}SCAN-3c 2.15Å

C	-3.416798	0.239381	-1.886700
C	-4.338984	0.928003	-3.702705
C	-5.578891	0.240132	-3.716094
H	-3.577147	0.599827	-4.405170
H	-4.360255	1.994279	-3.493199
C	-5.712493	-1.060852	-4.283227
C	-6.747191	0.779772	-3.103182
C	-7.937299	0.092585	-3.069932
C	-8.050169	-1.191251	-3.650387
C	-6.900393	-1.751689	-4.252105
H	-8.810778	0.534171	-2.597221
H	-6.680220	1.761671	-2.641423
H	-6.969200	-2.741416	-4.696093
H	-4.837475	-1.515616	-4.740449
C	-9.276736	-1.887210	-3.641208
O	-3.703577	1.032590	-1.028009
O	-2.823149	-0.771280	-2.159802
N	-10.290449	-2.462310	-3.636388

18

TS(NCBn—CO2−) r^{2}SCAN-3c 2.30Å

C	-3.398588	0.196359	-1.808365
C	-4.358201	0.954409	-3.756314
C	-5.580901	0.258913	-3.744427
H	-3.569153	0.609426	-4.418178
H	-4.356716	2.010632	-3.503809
C	-5.716667	-1.050084	-4.305274
C	-6.754288	0.792558	-3.124183
C	-7.936628	0.096559	-3.075567
C	-8.048925	-1.194238	-3.645665
C	-6.898222	-1.747244	-4.257173
H	-8.808616	0.535702	-2.597292
H	-6.693284	1.779240	-2.671259
H	-6.963800	-2.739893	-4.695683
H	-4.845070	-1.502074	-4.772295
C	-9.267438	-1.899511	-3.616711
O	-3.699184	1.013271	-0.990534
O	-2.838567	-0.809608	-2.125144
N	-10.276954	-2.483629	-3.595418

18

TS(NCBn—CO2−) r^{2}SCAN-3c 2.45Å

C	-3.389934	0.148339	-1.727899
C	-4.373479	0.981288	-3.811486
C	-5.581213	0.277953	-3.774161
H	-3.562897	0.621203	-4.437553
H	-4.353439	2.027876	-3.523946
C	-5.719073	-1.038941	-4.327324
C	-6.757706	0.805841	-3.144715
C	-7.932676	0.101761	-3.079765
C	-8.045042	-1.195675	-3.639518
C	-6.894439	-1.742089	-4.261529
H	-8.802368	0.538669	-2.594764
H	-6.701263	1.796770	-2.699926
H	-6.957335	-2.737793	-4.694052
H	-4.851149	-1.488510	-4.804096
C	-9.255540	-1.909711	-3.589725
O	-3.723545	0.978393	-0.949344
O	-2.849579	-0.841979	-2.092730
N	-10.260526	-2.502608	-3.550759

18

TS(NCBn—CO2−) r^{2}SCAN-3c 2.60Å

C	-3.427128	0.083084	-1.647347
C	-4.381212	1.013999	-3.879637
C	-5.573974	0.301782	-3.811788
H	-3.551941	0.637673	-4.470326
H	-4.343421	2.049757	-3.557317
C	-5.714872	-1.023389	-4.355272
C	-6.749671	0.822977	-3.165949
C	-7.915889	0.109470	-3.077144
C	-8.029405	-1.194686	-3.625368
C	-6.882680	-1.733913	-4.264537
H	-8.780520	0.543086	-2.579818
H	-6.696202	1.817822	-2.729085
H	-6.944177	-2.732949	-4.690055
H	-4.853185	-1.470025	-4.846543
C	-9.230365	-1.918964	-3.546796
O	-3.824247	0.912949	-0.911420
O	-2.882986	-0.876194	-2.061492
N	-10.229329	-2.521692	-3.483399

18

TS(NCBn—CO2−) r^{2}SCAN-3c 2.75Å

C	-3.489692	0.004594	-1.569207
C	-4.385684	1.051003	-3.949276
C	-5.564340	0.328798	-3.850679
H	-3.545367	0.664369	-4.517429
H	-4.335524	2.078262	-3.602006
C	-5.706012	-1.004759	-4.382135
C	-6.737479	0.842223	-3.186871
C	-7.893735	0.118204	-3.072001
C	-8.006763	-1.192862	-3.607498
C	-6.864693	-1.724376	-4.264434
H	-8.753036	0.547800	-2.561701
H	-6.687275	1.841230	-2.758708
H	-6.923782	-2.727315	-4.681508
H	-4.850276	-1.447716	-4.887482
C	-9.196800	-1.928508	-3.498466
O	-3.935183	0.839612	-0.877858
O	-2.947158	-0.928235	-2.026614
N	-10.188404	-2.541537	-3.409420

18

TS(NCBn—CO2−) r²SCAN-3c 2.90Å

C	-3.577124	-0.087584	-1.499731
C	-4.388856	1.094270	-4.020508
C	-5.553498	0.360027	-3.890751
H	-3.542408	0.701927	-4.575771
H	-4.332426	2.116492	-3.659173
C	-5.691914	-0.982222	-4.406903
C	-6.723028	0.865418	-3.209868
C	-7.867360	0.128942	-3.065946
C	-7.975974	-1.189959	-3.585428
C	-6.839132	-1.713521	-4.259600
H	-8.721726	0.553781	-2.543279
H	-6.677336	1.869444	-2.792731
H	-6.893731	-2.721420	-4.665478
H	-4.841080	-1.420586	-4.924702
C	-9.153040	-1.939136	-3.443494
O	-4.047871	0.755841	-0.842550
O	-3.049660	-1.007108	-1.990771
N	-10.135037	-2.563819	-3.326608

18

TS(NCBn—CO2−) r²SCAN-3c 3.05Å

C	-3.674298	-0.182180	-1.437234
C	-4.392295	1.138337	-4.091139
C	-5.542311	0.390625	-3.928357
H	-3.545555	0.745274	-4.645566
H	-4.336048	2.160073	-3.728243
C	-5.675119	-0.960112	-4.427216
C	-6.706591	0.887757	-3.229791
C	-7.839310	0.139885	-3.058876
C	-7.942772	-1.185769	-3.563768
C	-6.810900	-1.702514	-4.252704
H	-8.688940	0.559552	-2.524303
H	-6.665427	1.895724	-2.821545
H	-6.860637	-2.715449	-4.646677
H	-4.828275	-1.395243	-4.954385
C	-9.106910	-1.947578	-3.391131
O	-4.155618	0.670322	-0.803541
O	-3.161464	-1.094783	-1.951158
N	-10.078732	-2.583132	-3.247657

18

TS(NCBn—CO2−) r²SCAN-3c SMD 1.40Å

C	-3.464757	0.419925	-2.283958
C	-4.283943	0.863654	-3.328965
C	-5.601195	0.150776	-3.484422
H	-3.720396	0.745107	-4.268318
H	-4.465826	1.942739	-3.243305
C	-5.667800	-1.100803	-4.110745
C	-6.782177	0.713942	-2.990163
C	-7.995616	0.049289	-3.094427
C	-8.042935	-1.205049	-3.719148
C	-6.867272	-1.778206	-4.235168
H	-8.906534	0.492505	-2.703828
H	-6.748591	1.688553	-2.509189
H	-6.910685	-2.746302	-4.724797
H	-4.758168	-1.545783	-4.504352
C	-9.278766	-1.893891	-3.836740
O	-2.475084	1.156374	-2.002780
O	-3.752309	-0.677880	-1.721479
N	-10.289149	-2.454162	-3.941509

18

TS(NCBn—CO2−) r²SCAN-3c SMD 1.55Å

C	-3.434321	0.386733	-2.205325
C	-4.305787	0.883583	-3.386929
C	-5.609743	0.176191	-3.534562
H	-3.697039	0.740633	-4.289464
H	-4.461156	1.961113	-3.269785
C	-5.676745	-1.084166	-4.146854
C	-6.789224	0.725494	-3.014553
C	-7.996502	0.049784	-3.095109
C	-8.044237	-1.209170	-3.713466
C	-6.871451	-1.774407	-4.243727
H	-8.904153	0.485320	-2.688469
H	-6.755680	1.700469	-2.534215
H	-6.913002	-2.747487	-4.723762
H	-4.770050	-1.525722	-4.551174
C	-9.276129	-1.906469	-3.811868
O	-2.476683	1.144029	-1.916031
O	-3.745446	-0.709691	-1.681993
N	-10.283858	-2.475451	-3.896008

18

TS(NCBn—CO2−) r²SCAN-3c SMD 1.70Å

C	-3.404283	0.350360	-2.127090
C	-4.328639	0.903915	-3.442059
C	-5.617414	0.201909	-3.578767
H	-3.677110	0.729454	-4.305055
H	-4.453603	1.979571	-3.295913
C	-5.685692	-1.066808	-4.180703
C	-6.797931	0.739170	-3.038658
C	-7.997789	0.051885	-3.096582
C	-8.045858	-1.212280	-3.708222
C	-6.874518	-1.768392	-4.252733
H	-8.902665	0.481120	-2.676927
H	-6.766694	1.716279	-2.562364
H	-6.913565	-2.745301	-4.725445
H	-4.781813	-1.503732	-4.596608
C	-9.271287	-1.920685	-3.783629
O	-2.482468	1.131858	-1.833595
O	-3.734741	-0.747403	-1.650554
N	-10.275135	-2.500133	-3.848387

18

TS(NCBn—CO2−) r²SCAN-3c SMD 1.85Å

C	-3.378086	0.319568	-2.045114
C	-4.350060	0.922530	-3.499144
C	-5.621754	0.223553	-3.620525
H	-3.655480	0.713454	-4.316745
H	-4.445452	1.995226	-3.325603
C	-5.695236	-1.051776	-4.218159
C	-6.803621	0.749579	-3.059459
C	-7.995991	0.052626	-3.095638
C	-8.047538	-1.215831	-3.703294
C	-6.878373	-1.762108	-4.265709
H	-8.896808	0.476322	-2.661376
H	-6.773335	1.727887	-2.585321
H	-6.916525	-2.741209	-4.734378
H	-4.795763	-1.484380	-4.648478
C	-9.266296	-1.933338	-3.755322
O	-2.491932	1.125698	-1.757157
O	-3.732103	-0.774962	-1.611365
N	-10.266851	-2.522051	-3.800506

18

TS(NCBn—CO2−) r²SCAN-3c SMD 2.00Å

C	-3.379524	0.253670	-1.981895
C	-4.356734	0.942450	-3.585218
C	-5.611716	0.244713	-3.666952
H	-3.625827	0.682462	-4.352392
H	-4.421865	2.011453	-3.390092

C	-5.698385	-1.051331	-4.233120
C	-6.796217	0.783942	-3.105493
C	-7.983862	0.084057	-3.105074
C	-8.045427	-1.205410	-3.673874
C	-6.879521	-1.762341	-4.238758
H	-8.879519	0.520843	-2.672439
H	-6.763256	1.776921	-2.662793
H	-6.922247	-2.753810	-4.680988
H	-4.806707	-1.493545	-4.668701
C	-9.260814	-1.924030	-3.689026
O	-2.944745	1.191778	-1.334781
O	-3.373576	-0.963793	-1.959860
N	-10.261262	-2.517240	-3.701835

18

TS(NCBn—CO2−) r^{2}SCAN-3c SMD 2.15Å

C	-3.360212	0.219737	-1.901514
C	-4.375953	0.963404	-3.644423
C	-5.612035	0.263173	-3.699987
H	-3.608007	0.672800	-4.360274
H	-4.413364	2.025364	-3.414003
C	-5.705433	-1.039878	-4.264830
C	-6.800320	0.793500	-3.121777
C	-7.979701	0.085347	-3.102927
C	-8.045554	-1.209058	-3.667788
C	-6.880267	-1.756413	-4.249976
H	-8.871532	0.517918	-2.657588
H	-6.769307	1.787815	-2.681556
H	-6.922052	-2.748988	-4.690517
H	-4.818832	-1.478431	-4.715152
C	-9.253241	-1.934353	-3.663094
O	-2.965407	1.180270	-1.284130
O	-3.379238	-0.985949	-1.923946
N	-10.250748	-2.535469	-3.659809

18

TS(NCBn—CO2−) r^{2}SCAN-3c SMD 2.30Å

C	-3.377073	0.175373	-1.817180
C	-4.382622	0.982802	-3.721630
C	-5.600885	0.279488	-3.741478
H	-3.579484	0.658376	-4.379694
H	-4.392958	2.035245	-3.452788
C	-5.708669	-1.032280	-4.301948
C	-6.789201	0.806920	-3.142941
C	-7.961447	0.093696	-3.096261
C	-8.038477	-1.208129	-3.651419
C	-6.878599	-1.750376	-4.257185
H	-8.845368	0.526459	-2.634778
H	-6.755241	1.804431	-2.709793
H	-6.924870	-2.745102	-4.693251
H	-4.832062	-1.470805	-4.772209
C	-9.239359	-1.936732	-3.616711
O	-3.175264	1.150553	-1.154769
O	-3.294131	-1.006198	-1.970026
N	-10.235493	-2.542931	-3.589231

18

TS(NCBn—CO2−) r^{2}SCAN-3c SMD 2.45Å

C	-3.474356	0.121204	-1.722217
C	-4.360510	0.984932	-3.836739
C	-5.567158	0.287850	-3.796449
H	-3.540380	0.613108	-4.444201
H	-4.327852	2.028068	-3.535784
C	-5.709610	-1.032163	-4.351059
C	-6.740944	0.821951	-3.158606
C	-7.911148	0.114083	-3.069243
C	-8.019798	-1.192777	-3.615759
C	-6.879500	-1.740483	-4.262101
H	-8.775900	0.554256	-2.578455
H	-6.689541	1.821506	-2.732248
H	-6.945778	-2.736021	-4.694564
H	-4.854259	-1.477970	-4.854109
C	-9.219611	-1.912496	-3.537099
O	-3.868988	0.930923	-0.955130
O	-2.906839	-0.850949	-2.086478
N	-10.219033	-2.514233	-3.473052

18

TS(NCBn—CO2−) r^{2}SCAN-3c SMD 2.60Å

C	-3.488646	0.063161	-1.646297
C	-4.374211	1.015260	-3.897803
C	-5.562845	0.308429	-3.825679
H	-3.532152	0.625888	-4.462496
H	-4.320386	2.043066	-3.550987
C	-5.706406	-1.022587	-4.367857
C	-6.738921	0.835800	-3.174253
C	-7.901417	0.120898	-3.069036
C	-8.010992	-1.192462	-3.606912
C	-6.869820	-1.735722	-4.261630
H	-8.763000	0.558552	-2.569896
H	-6.690631	1.837891	-2.752978
H	-6.932895	-2.735499	-4.685348
H	-4.853572	-1.468115	-4.875935
C	-9.202794	-1.918232	-3.510284
O	-3.931696	0.887713	-0.938000
O	-2.932037	-0.876497	-2.076716
N	-10.198783	-2.526756	-3.431185

18

TS(NCBn—CO2−) r^{2}SCAN-3c SMD 2.75Å

C	-3.520512	-0.006691	-1.572727
C	-4.385331	1.050818	-3.959411
C	-5.559304	0.332392	-3.857679
H	-3.529857	0.650014	-4.495412
H	-4.319910	2.068416	-3.585218
C	-5.700208	-1.007844	-4.385449
C	-6.735239	0.852734	-3.193927
C	-7.889072	0.129008	-3.069579
C	-7.996347	-1.191099	-3.595910
C	-6.855503	-1.728863	-4.259471
H	-8.747588	0.563157	-2.561773
H	-6.690348	1.857862	-2.779115
H	-6.913760	-2.733542	-4.672598
H	-4.849262	-1.451623	-4.898549
C	-9.178377	-1.926267	-3.476915
O	-3.986688	0.834557	-0.909091
O	-2.985490	-0.928802	-2.051244
N	-10.168408	-2.543438	-3.379224

18

TS(NCBn—CO2−) r^{2}SCAN-3c SMD 2.90Å

C	-3.572177	-0.079179	-1.498299
C	-4.393064	1.086615	-4.023585
C	-5.554614	0.356364	-3.892737
H	-3.534430	0.682059	-4.552229
H	-4.324322	2.101087	-3.641050
C	-5.691878	-0.990804	-4.407306
C	-6.725928	0.869216	-3.212191
C	-7.871007	0.136416	-3.066391
C	-7.975517	-1.188374	-3.583314
C	-6.838622	-1.720523	-4.259490
H	-8.724798	0.565771	-2.546532
H	-6.683218	1.876341	-2.801875
H	-6.893114	-2.729298	-4.663220

H	-4.843535	-1.431806	-4.927217
C	-9.147640	-1.933718	-3.439338
O	-4.050337	0.776106	-0.866340
O	-3.056736	-0.995839	-2.001950
N	-10.130267	-2.559647	-3.320229

18

TS(NCBn—CO2−) r²SCAN-3c SMD 3.05Å

C	-3.622424	-0.146684	-1.423520
C	-4.400845	1.118841	-4.087165
C	-5.550724	0.377342	-3.926370
H	-3.543215	0.714263	-4.617879
H	-4.329997	2.132936	-3.703398
C	-5.684755	-0.975488	-4.428489
C	-6.715667	0.883681	-3.228974
C	-7.852547	0.143026	-3.062222
C	-7.955434	-1.185399	-3.570979
C	-6.823632	-1.712760	-4.260018
H	-8.701078	0.568576	-2.530658
H	-6.674353	1.892588	-2.822834
H	-6.875415	-2.724660	-4.656294
H	-4.840605	-1.414323	-4.957078
C	-9.119018	-1.938758	-3.404543
O	-4.120154	0.709537	-0.810558
O	-3.106373	-1.050494	-1.946420
N	-10.094968	-2.571438	-3.265894

13. References

[1] N. Hirone, H. Sanjiki, R. Tanaka, T. Hata, H. Urabe, *Angew. Chem. Int. Ed.* **2010**, *49*, 7762–7764.

[2] Y. Gao, L. Wang, L. Deng, *ACS Catal.* **2018**, *8*, 9637–9646.

[3] I. Hossain, J. A. R. Schmidt, *Organometallics* **2020**, *39*, 3441–3451.

[4] J. Robertson, M. J. Hall, P. M. Stafford, S. P. Green, *Org. Biomol. Chem.* **2003**, *1*, 3758–3767.

[5] Z. Li, C. Yang, H. Zheng, H. Qiu, G. Lai, *J. Organomet. Chem.* **2008**, *693*, 3771–3779.

[6] S.-S. Yan, S.-H. Liu, L. Chen, Z.-Y. Bo, K. Jing, T.-Y. Gao, B. Yu, Y. Lan, S.-P. Luo, D.-G. Yu, *Chem* **2021**, *7*, 3099–3113.

[7] Q.-Y. Meng, S. Wang, G. S. Huff, B. König, *J. Am. Chem. Soc.* **2018**, *140*, 3198–3201.

[8] D. Bai, F. Wu, L. Chang, M. Wang, H. Wu, J. Chang, *Angew. Chem. Int. Ed.* **2022**, *61*, e202114918.

[9] Z. Cheng, J. Guo, Y. Sun, Y. Zheng, Z. Zhou, Z. Lu, *Angew. Chem. Int. Ed.* **2021**, *60*, 22454–22460.

[10] P. He, F. Zhang, X. Si, W. Jiang, Q. Shen, Z. Li, Z. Zhu, S. Tang, Q.-W. Gui, *Synthesis* **2023**, *55*, 765–772.

[11] X. Wang, C. Wang, Y. Liu, J. Xiao, *Green Chem.* **2016**, *18*, 4605–4610.

[12] L. Nielsen, T. Skrydstrup, *J. Am. Chem. Soc.* **2008**, *130*, 13145–13151.

[13] M. E. Avanthay, O. H. Goodrich, D. Tiemessen, C. M. Alder, M. W. George, A. J. J. Lennox, *JACS Au* **2024**, *4*, 2220–2227.

[14] Y. Hua, H. H. Nguyen, G. Trog, A. S. Berlin, J. Jeon, *Eur. J. Org. Chem.* **2014**, *2014*, 5890–5895.

[15] A. Weickgenannt, M. Oestreich, *Chem. Asian J.* **2009**, *4*, 406–410.

[16] J. J. Kennedy-Smith, K. A. Nolin, H. P. Gunterman, F. D. Toste, *J. Am. Chem. Soc.* **2003**, *125*, 4056–4057.

[17] T. Ito, A. Hayashi, A. Kondo, T. Uchida, K. Tanabe, H. Yamada, S. Nishimoto, *Org. Lett.* **2009**, *11*, 927–930.

[18] Q. Yan, Y. C. Fang, Y. X. Jia, X. H. Duan, *New J. Chem.* **2017**, *41*, 2372–2377.

[19] R. Baati, A. Valleix, C. Mioskowski, D. K. Barma, J. R. Falck, *Org. Lett.* **2000**, *2*, 485–487.

[20] H. M. S. Kumar, B. V. S. Reddy, E. J. Reddy, J. S. Yadav, *Chem. Lett.* **1999**, *28*, 857–858.

[21] B. Das, M. Krishnaiah, V. S. Reddy, K. Laxminarayana, *Helv. Chim. Acta* **2007**, *90*, 2163–2166.

[22] X. Si, L. Zhang, Z. Wu, M. Rudolph, A. M. Asiri, A. S. K. Hashmi, *Org. Lett.* **2020**, *22*, 5844–5849.

[23] J. A. Gurak, K. M. Engle, *ACS Catal.* **2018**, *8*, 8987–8992.

[24] F. Neese, *WIREs Comput. Mol. Sci.* **2012**, *2*, 73–78.

[25] F. Neese, *WIREs Comput. Mol. Sci.* **2018**, *8*, e1327.

[26] F. Neese, *WIREs Comput. Mol. Sci.* **2022**, *12*, e1606.

[27] H. Kruse, S. Grimme, *J. Chem. Phys.* **2012**, *136*, 154101.

[28] E. Caldeweyher, C. Bannwarth, S. Grimme, *J. Chem. Phys.* **2017**, *147*, 034112.

[29] E. Caldeweyher, S. Ehlert, A. Hansen, H. Neugebauer, S. Spicher, C. Bannwarth, S. Grimme, *J. Chem. Phys.* **2019**, *150*, 154122.

[30] J. W. Furness, A. D. Kaplan, J. Ning, J. P. Perdew, J. Sun, *J. Phys. Chem. Lett.* **2020**, *11*, 8208–8215.

[31] S. Grimme, A. Hansen, S. Ehlert, J.-M. Mewes, *J. Chem. Phys.* **2021**, *154*, 064103.

[32] J.-D. Chai, M. Head-Gordon, *J. Chem. Phys.* **2008**, *128*, 084106.

[33] F. Weigend, R. Ahlrichs, *Phys. Chem. Chem. Phys.* **2005**, *7*, 3297–3305.

[34] C. van Wüllen, *J. Chem. Phys.* **1998**, *109*, 392–399.
[35] F. Weigend, *Phys. Chem. Chem. Phys.* **2006**, *8*, 1057–1065.
[36] D. A. Pantazis, X.-Y. Chen, C. R. Landis, F. Neese, *J. Chem. Theory Comput.* **2008**, *4*, 908–919.
[37] D. A. Pantazis, F. Neese, *J. Chem. Theory Comput.* **2009**, *5*, 2229–2238.
[38] D. A. Pantazis, F. Neese, *J. Chem. Theory Comput.* **2011**, *7*, 677–684.
[39] D. A. Pantazis, F. Neese, *Theor. Chem. Acc.* **2012**, *131*, 1292.
[40] A. V. Marenich, C. J. Cramer, D. G. Truhlar, *J. Phys. Chem. B* **2009**, *113*, 6378–6396.
[41] J. L. McDonagh, N. Nath, L. De Ferrari, T. van Mourik, J. B. O. Mitchell, *J. Chem. Inf. Model.* **2014**, *54*, 844–856.
[42] G. Knizia, J. E. M. N. Klein, *Angew. Chem. Int. Ed.* **2015**, *54*, 5518–5522.
[43] M. Namazian, C. Y. Lin, M. L. Coote, *J. Chem. Theory Comput.* **2010**, *6*, 2721–2725.
[44] A. Streitwieser, P. H. Owens, G. Sonnichsen, W. K. Smith, G. R. Ziegler, H. M. Niemeyer, T. L. Kruger, *J. Am. Chem. Soc.* **1973**, *95*, 4254–4257.
[45] V. Ásgeirsson, B. O. Birgisson, R. Bjornsson, U. Becker, F. Neese, C. Riplinger, H. Jónsson, *J. Chem. Theory Comput.* **2021**, *17*, 4929–4945.
[46] C. L. McMullin, J. Jover, J. N. Harvey, N. Fey, *Dalton Trans.* **2010**, *39*, 10833–10836.
[47] H. Eyring, *J. Chem. Phys.* **1935**, *3*, 107–115.
[48] K. J. Laidler, M. C. King, *J. Phys. Chem.* **1983**, *87*, 2657–2664.
[49] J. R. Rumble, *CRC Handbook of Chemistry and Physics*, 104th Edition, CRC Press, Taylor & Francis Group, an Informa Group company, **2023**.

14. Quantitative ^{1}H-NMR analysis of reaction mixtures for 2a-2s

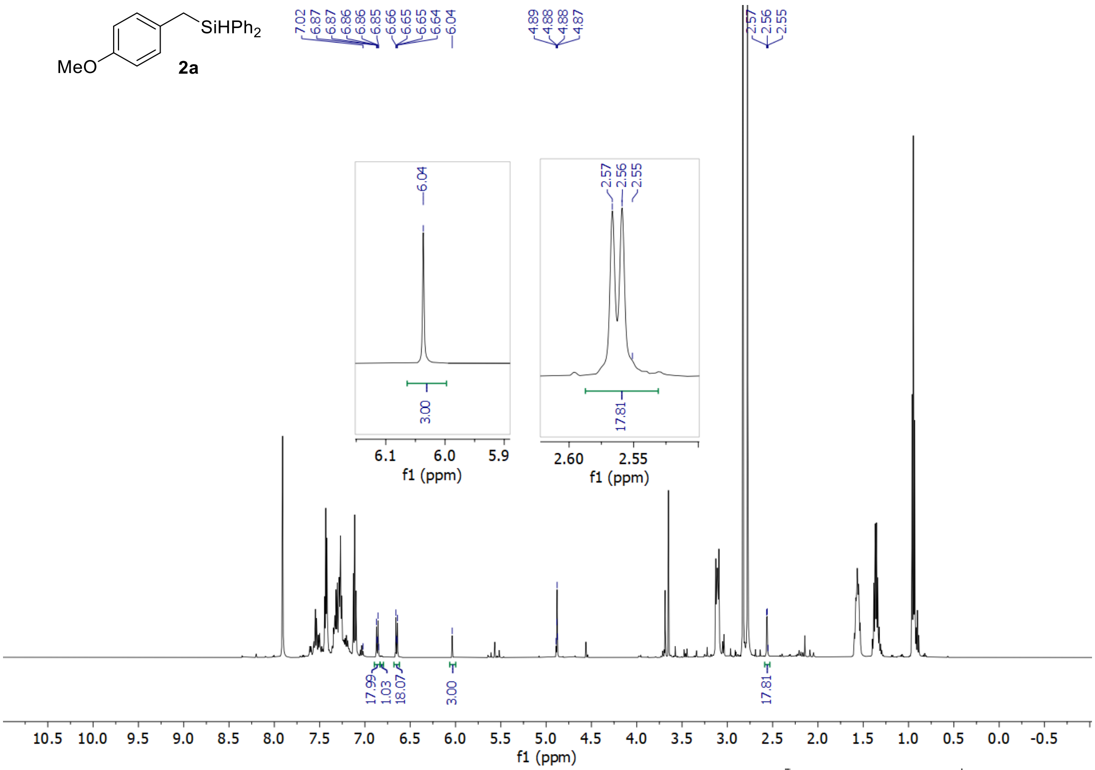

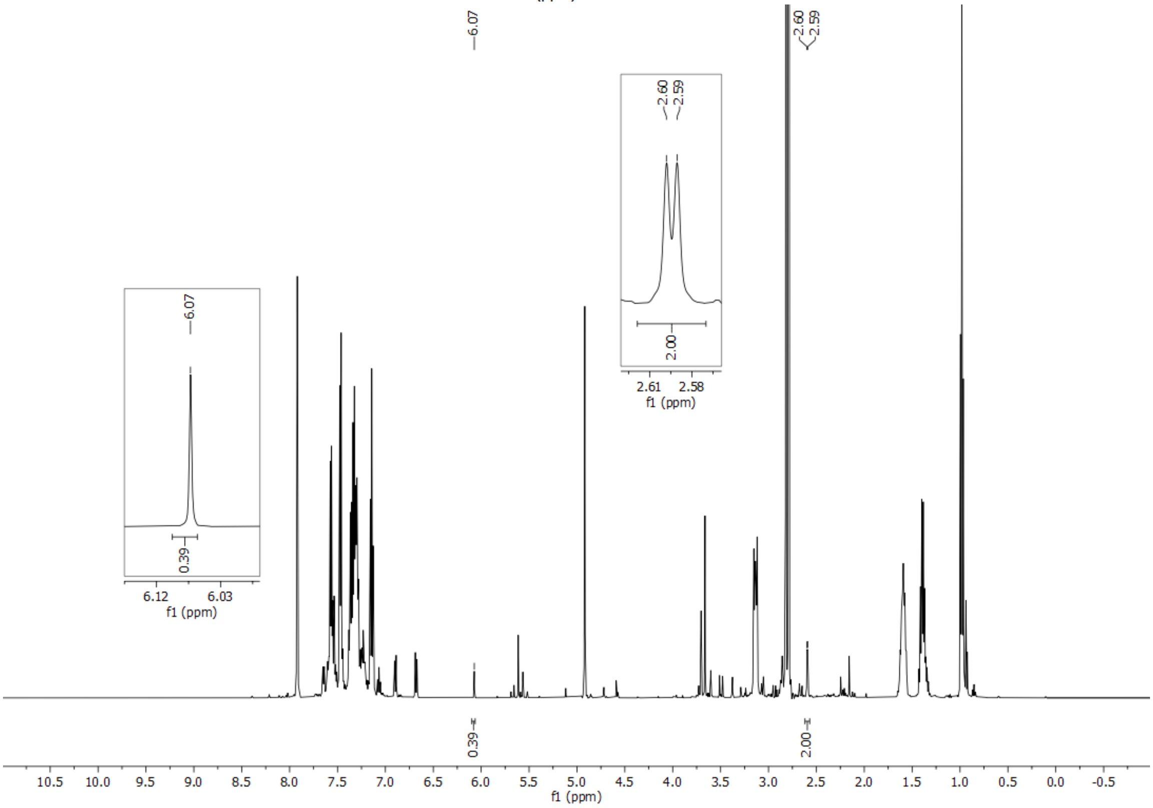

Figure S-30. *Top:* ^{1}H-NMR (500 MHz, $CDCl_3$) spectrum of reaction crude mixture after evaporation of solvent containing **2a** and internal standard 1,3,5-trimethoxybenzene (5.3 mg). Quantitative NMR analysis resulted in 92% yield for **2a** (Table S-1). See sections 2.2 and 5 for details. *Bottom:* from aldehyde ^{1}H-NMR(500 MHz, $CDCl_3$) spectrum of reaction crude mixture

after evaporation of solvent containing **2a** and internal standard 1,3,5-trimethoxybenzene (5.8 mg). Quantitative NMR analysis resulted in 88% yield for **2a** (Table S-1). See sections 2.2 and 5 for details.

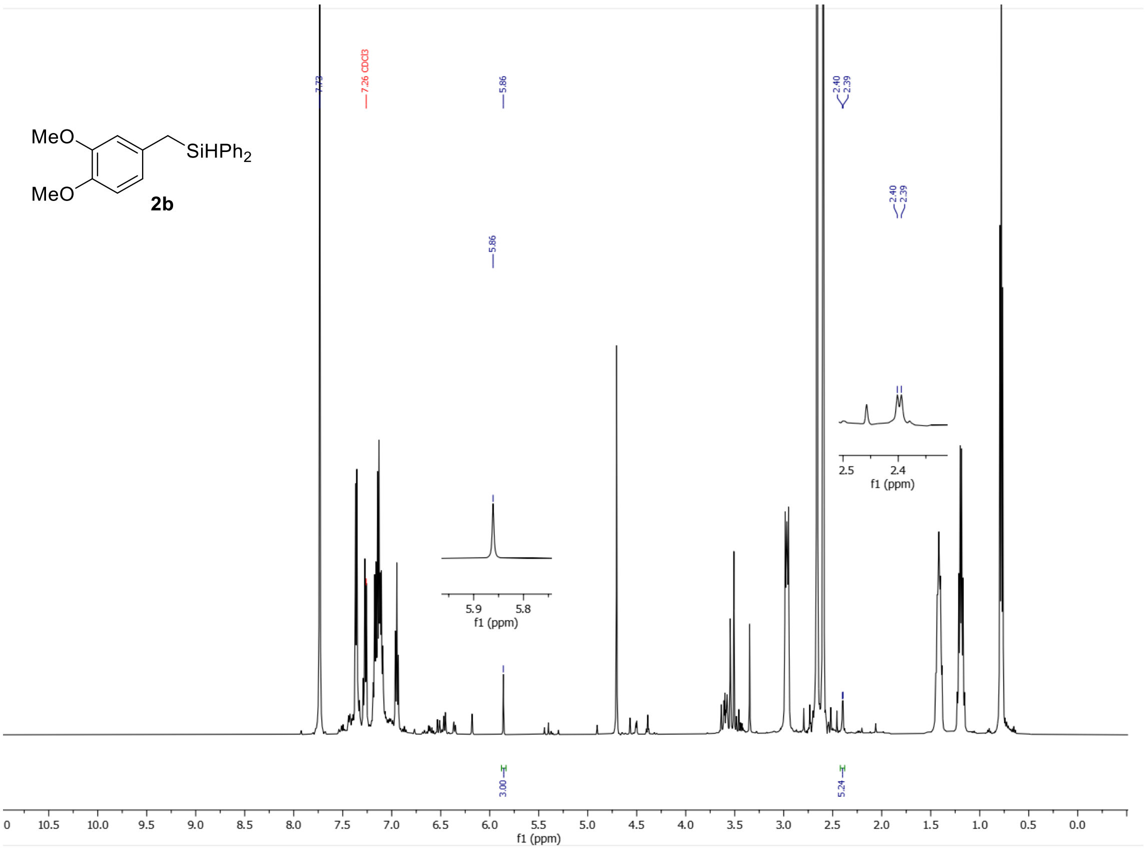

Figure S-31. ^{1}H-NMR(400 MHz, $CDCl_3$) spectrum of reaction crude mixture after evaporation of solvent containing **2b** and internal standard 1,3,5-trimethoxybenzene (9.0 mg). Quantitative NMR analysis resulted in 45% yield for **2b**. See sections 2.2 and 5 for details.

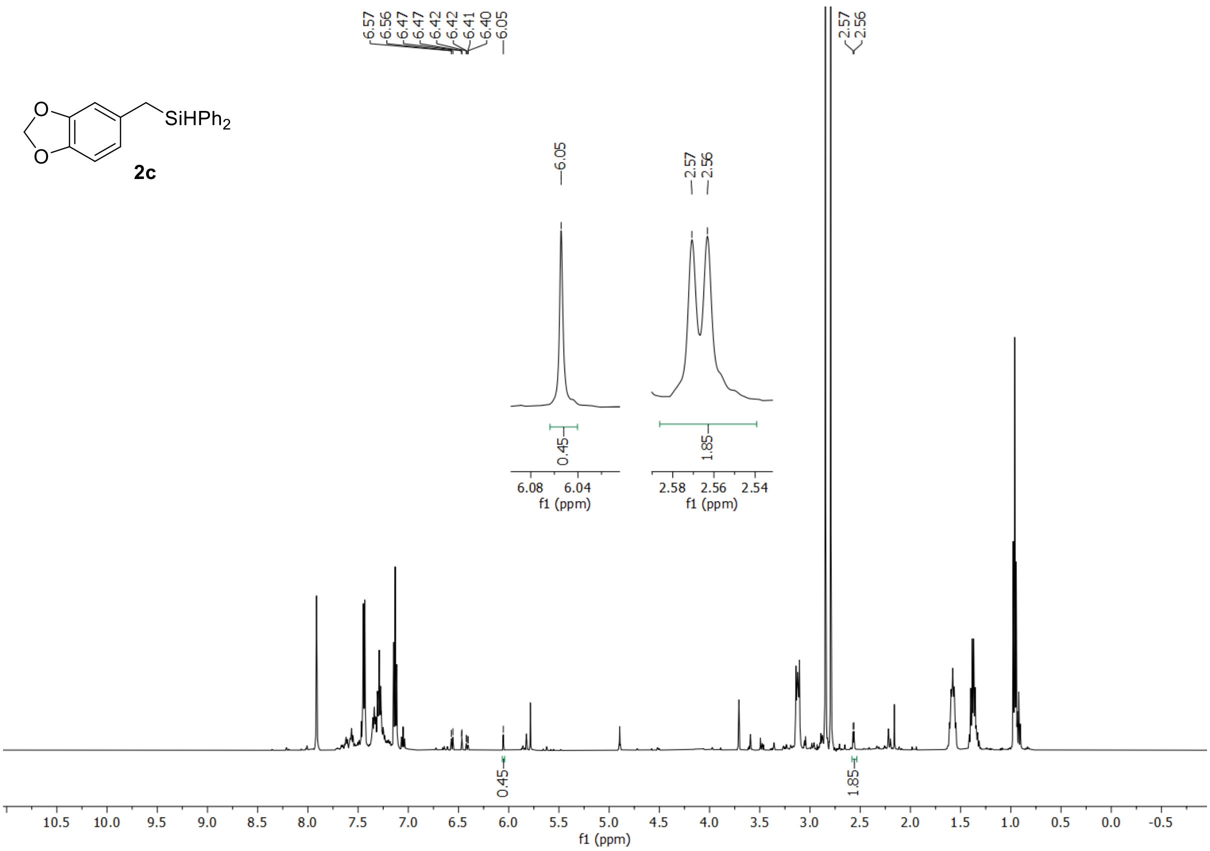

Figure S-32. ^{1}H-NMR(400 MHz, $CDCl_3$) spectrum of reaction crude mixture after evaporation of solvent containing **2c** and internal standard 1,3,5-trimethoxybenzene (5.2 mg). Quantitative NMR analysis resulted in 56% yield for **2c**. See sections 2.2 and 5 for details.

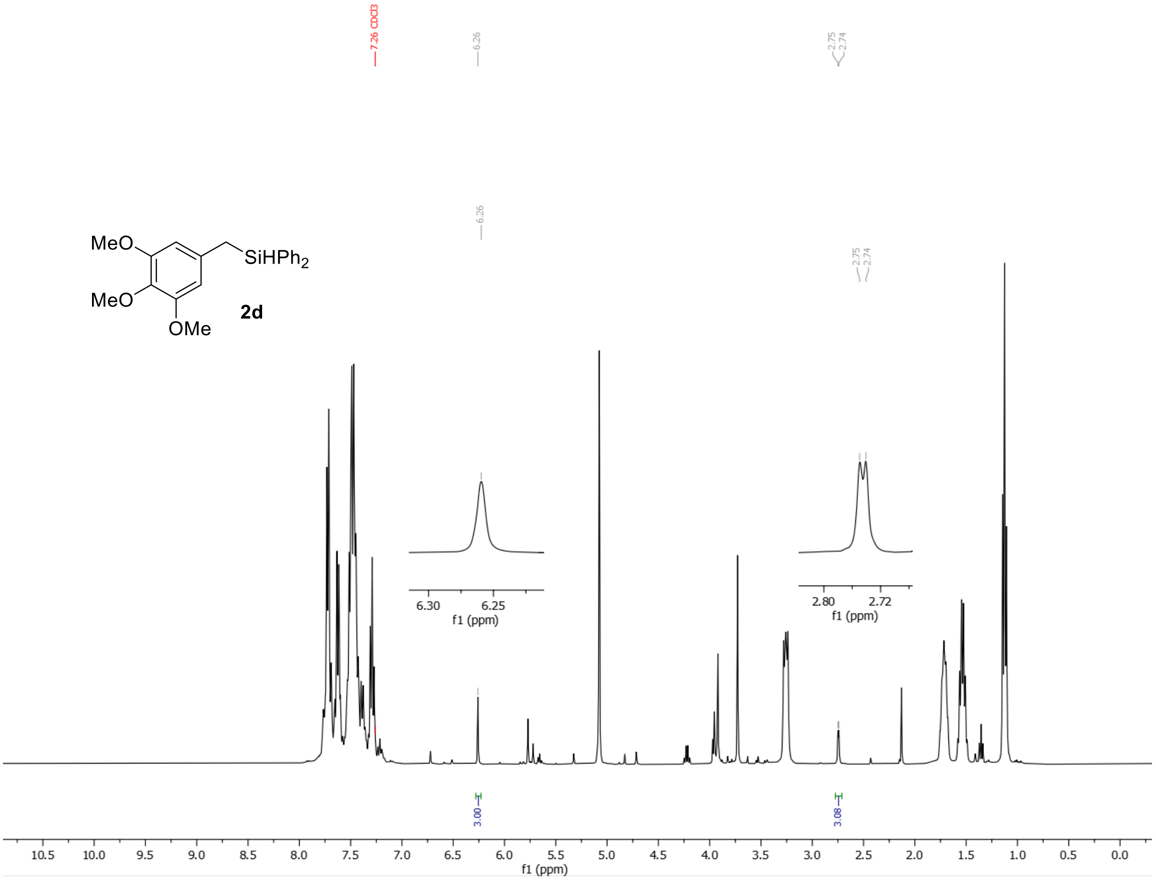

Figure S-33. ^{1}H-NMR(400 MHz, $CDCl_3$) spectrum of reaction crude mixture after evaporation of solvent containing **2d** and internal standard 1,3,5-trimethoxybenzene (7.7 mg). Quantitative NMR analysis resulted in 25% yield for **2d**. See sections 2.2 and 5 for details.

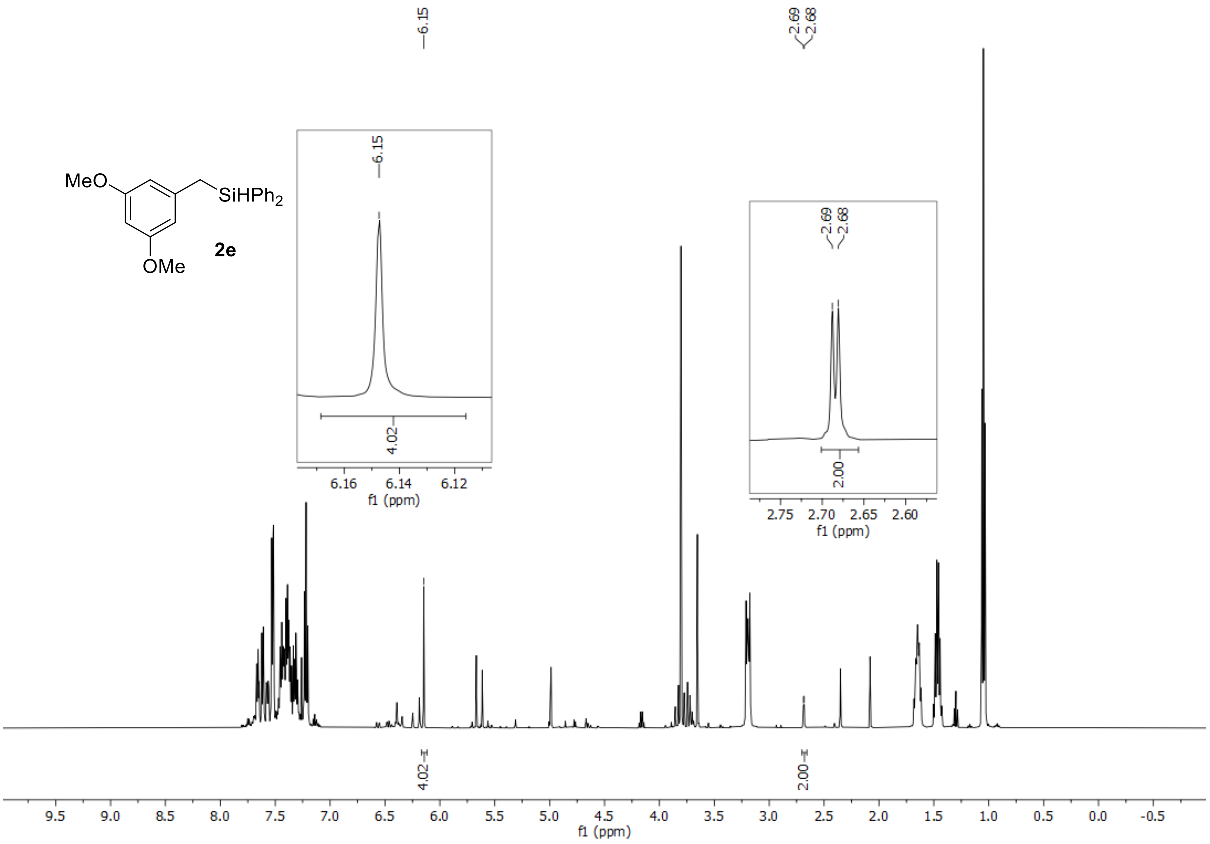

Figure S-34. ^{1}H-NMR(400 MHz, $CDCl_3$) spectrum of reaction crude mixture after evaporation of solvent containing **2e** and internal standard 1,3,5-trimethoxybenzene (18.4 mg). Quantitative NMR analysis resulted in 26% yield for **2e**. See sections 2.2 and 5 for details.

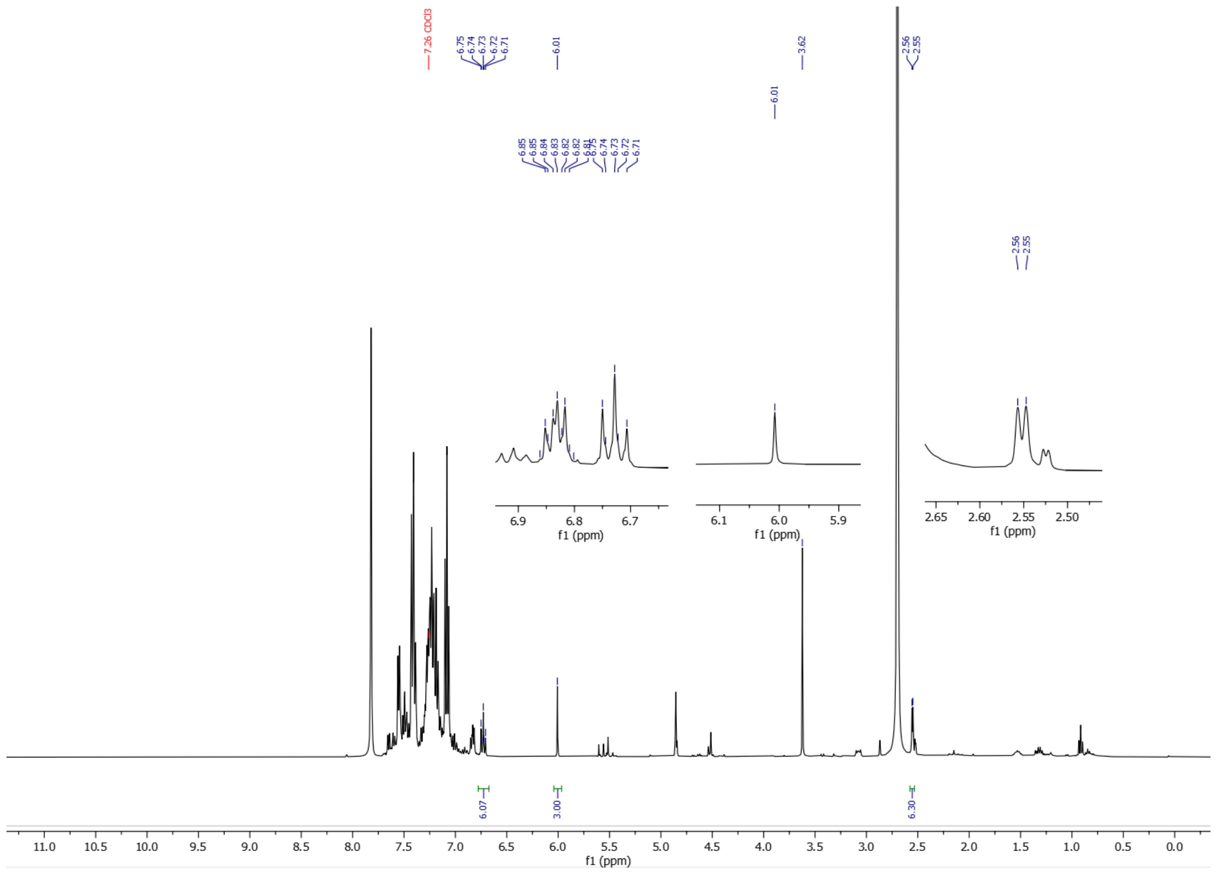

Figure S-35. ^{1}H-NMR(400 MHz, $CDCl_3$) spectrum of reaction crude mixture after evaporation of solvent and filtration through celite plug with Et_2O containing **2f** and internal standard 1,3,5-trimethoxybenzene (7.3 mg). Quantitative NMR analysis resulted in 44% yield for **2f**. See sections 2.2 and 5 for details.

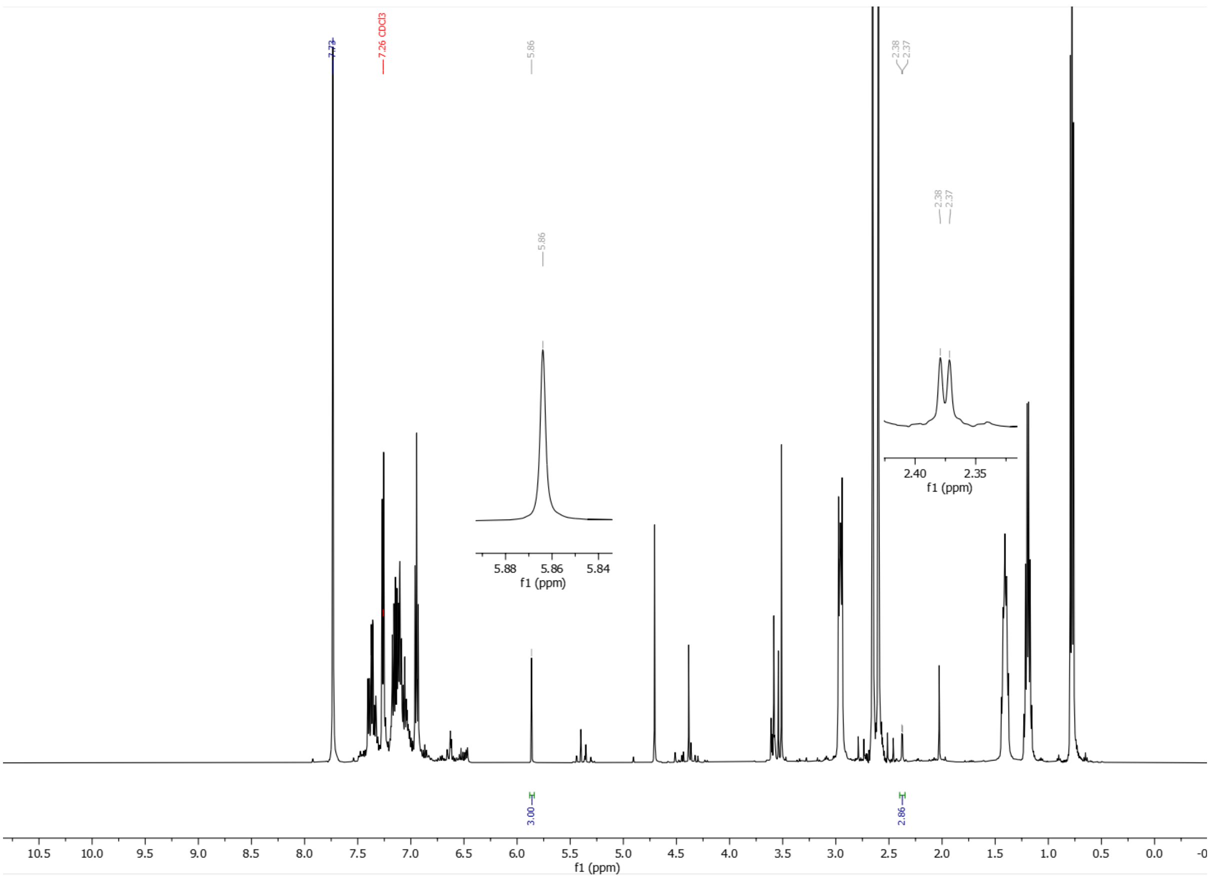

Figure S-36. ^{1}H-NMR(400 MHz, $CDCl_3$) spectrum of reaction crude mixture after evaporation of solvent and filtration through celite plug with $E_{t2}O$ containing **2g** and internal standard 1,3,5-trimethoxybenzene (10.7 mg). Quantitative NMR analysis resulted in 31% yield for **2g**. See sections 2.2 and 5 for details.

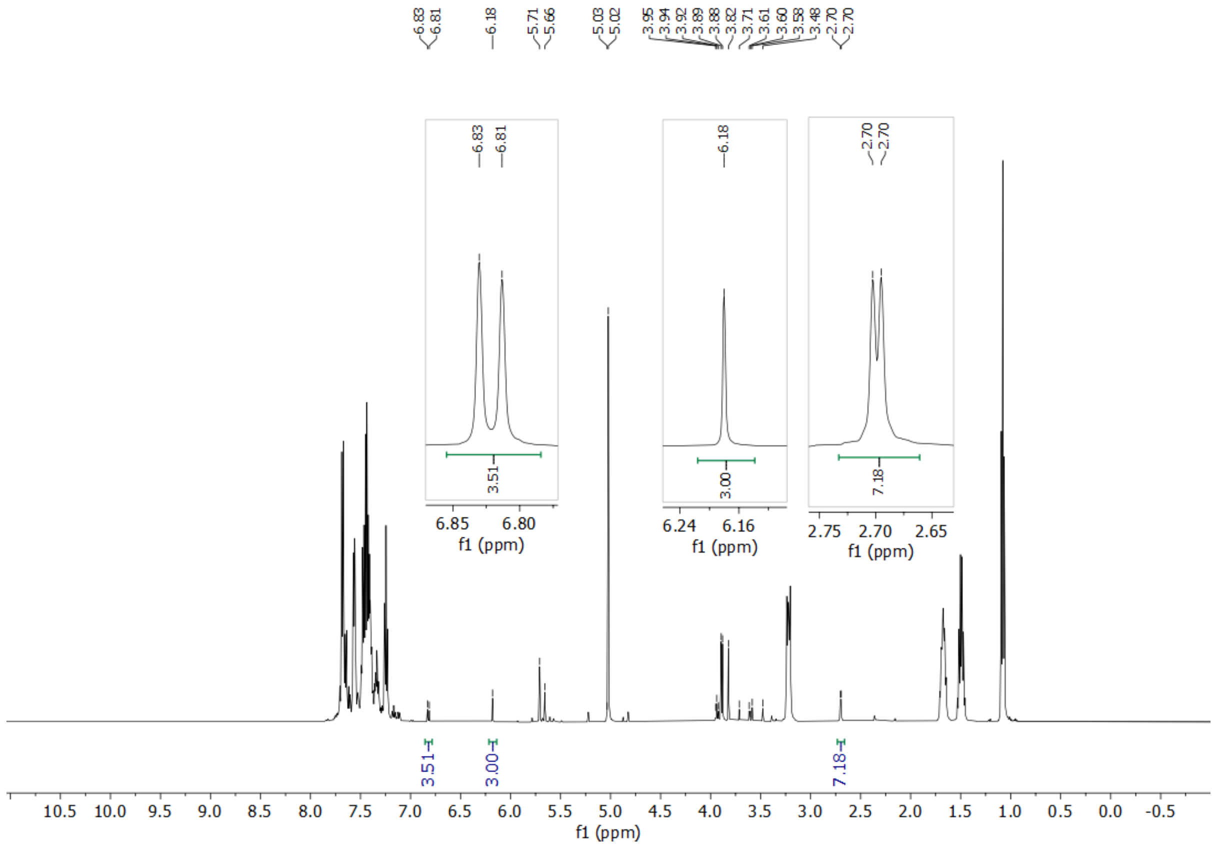

Figure S-37. ^{1}H-NMR(400 MHz, $CDCl_3$) spectrum of reaction mixture after extraction containing **2h** and internal standard 1,3,5-trimethoxybenzene (6.9 mg). Quantitative NMR analysis resulted in 46% yield for **2h**. See sections 2.2 and 5 for details.

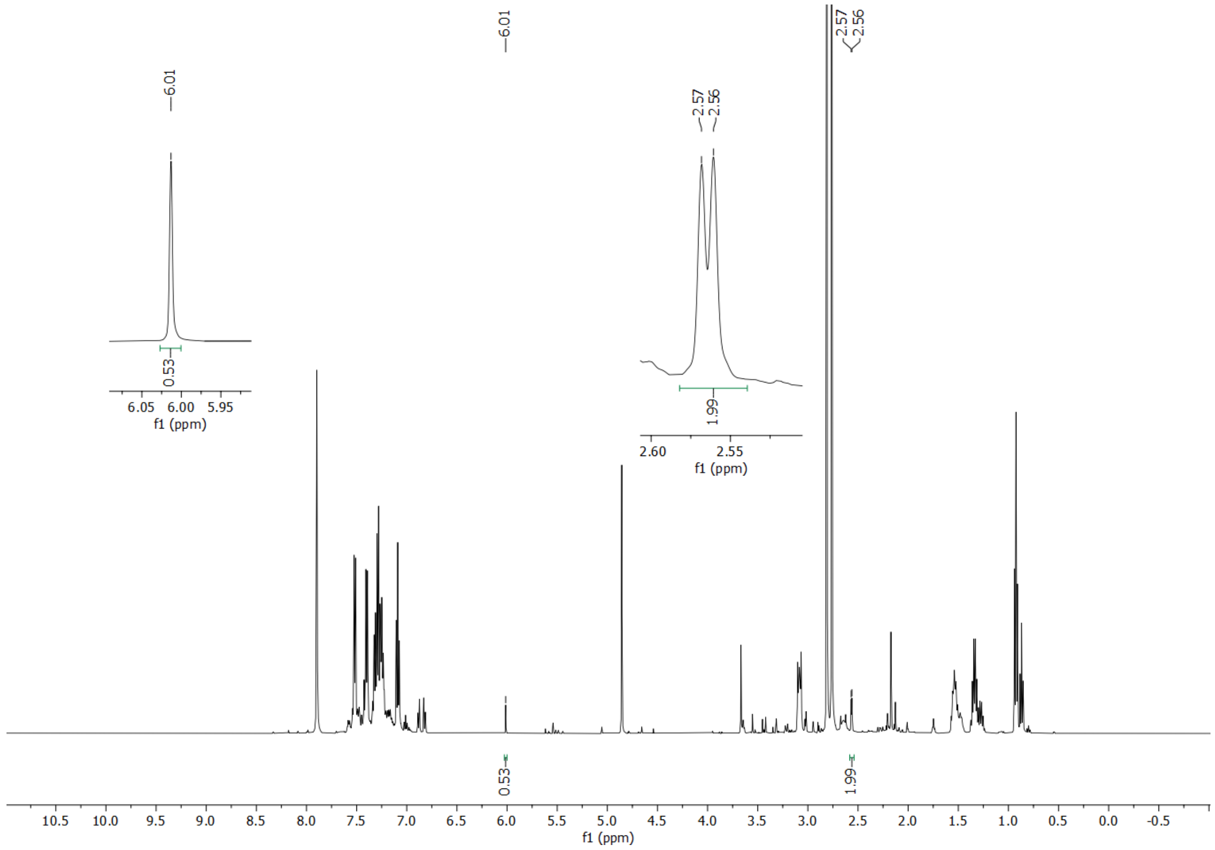

Figure S-38. ^{1}H-NMR(400 MHz, CDCl3) spectrum of reaction mixture after extraction containing **2i** and internal standard 1,3,5-trimethoxybenzene (7.2 mg). Quantitative NMR analysis resulted in 99% yield for **2i**. See sections 2.2 and 5 for details.

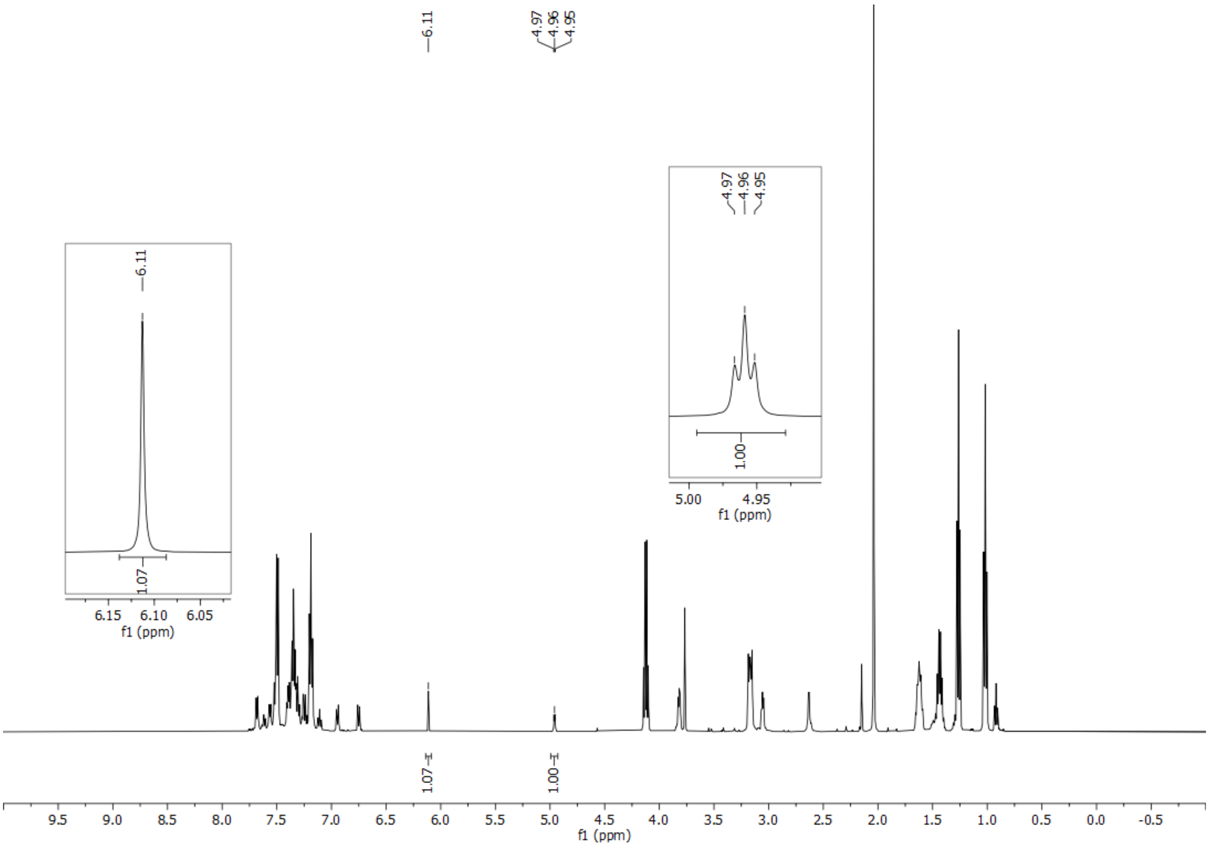

Figure S-39. ^{1}H-NMR(400 MHz, CDCl3) spectrum of reaction mixture after extraction containing **2j** and internal standard 1,3,5-trimethoxybenzene (10.0 mg). Quantitative NMR analysis resulted in 54% yield for **2j**. See sections 2.2 and 5 for details.

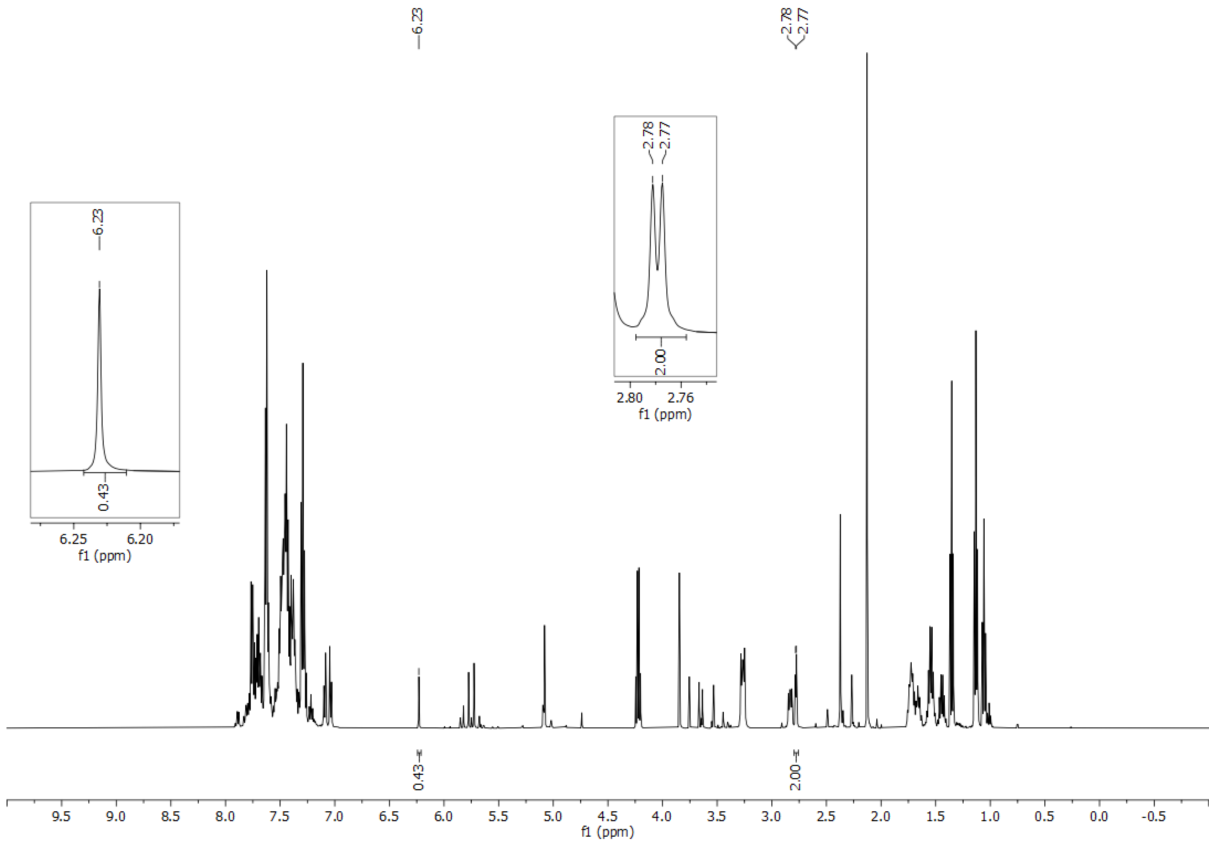

Figure S-40. ^{1}H-NMR(400 MHz, $CDCl_3$) spectrum of reaction mixture after extraction containing **2l** and internal standard 1,3,5-trimethoxybenzene (3.5 mg). Quantitative NMR analysis resulted in 85% yield for **2l**. See sections 2.2 and 5 for details.

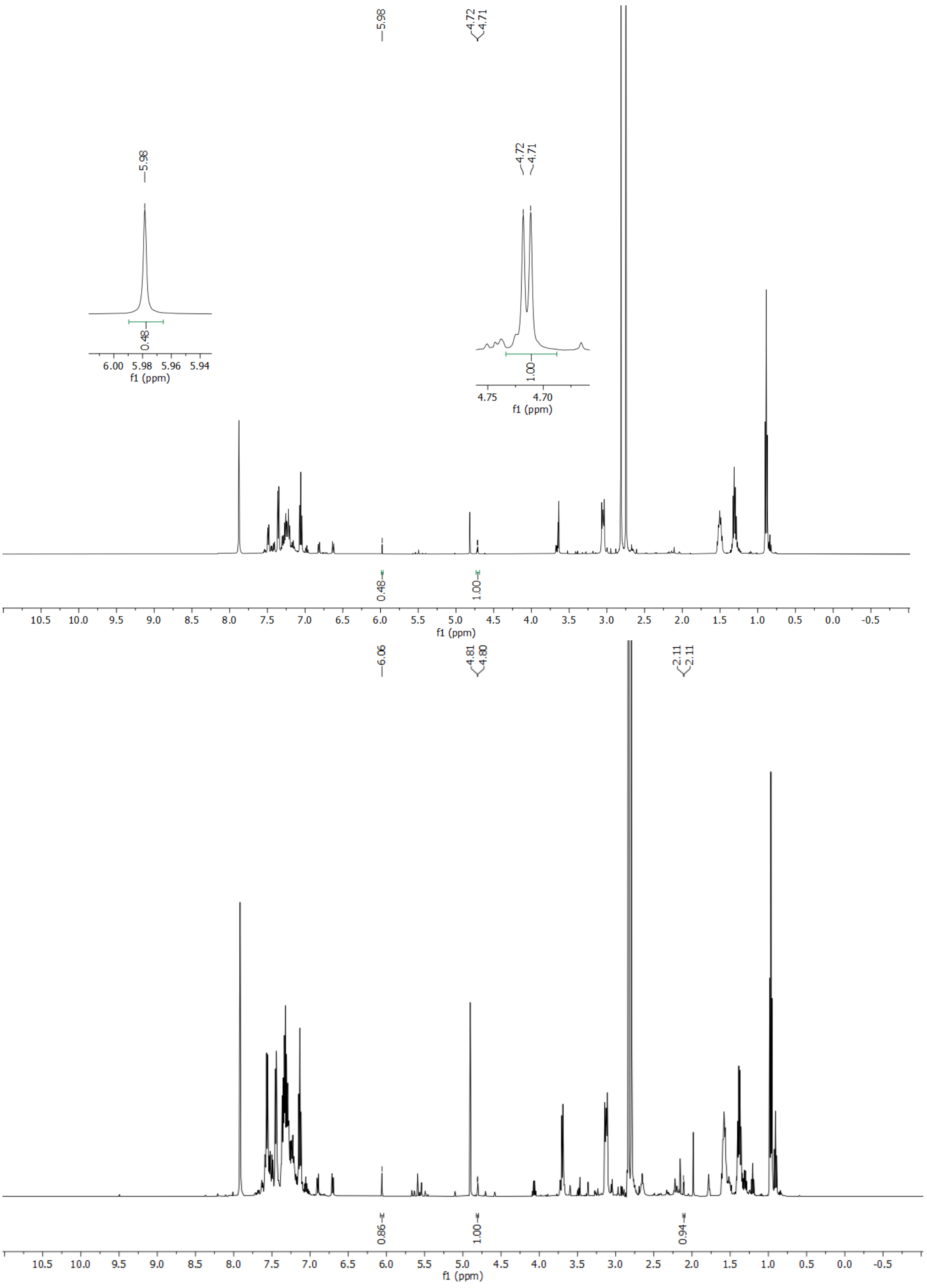

Figure S-41. *Top (from alcohol):* ^{1}H-NMR(400 MHz, $CDCl_3$) spectrum of reaction mixture after extraction containing **2m** and internal standard 1,3,5-trimethoxybenzene (5.9 mg). Quantitative NMR analysis resulted in 72% yield for **2m**. See sections 2.2 and 5 for details. *Bottom (from ketone):* ^{1}H-NMR(500 MHz, $CDCl_3$) spectrum of reaction mixture after extraction containing **2m** and internal standard 1,3,5-trimethoxybenzene (8.5 mg). Quantitative NMR analysis resulted in 58% yield for **2m**. See sections 2.2 and 5 for details.

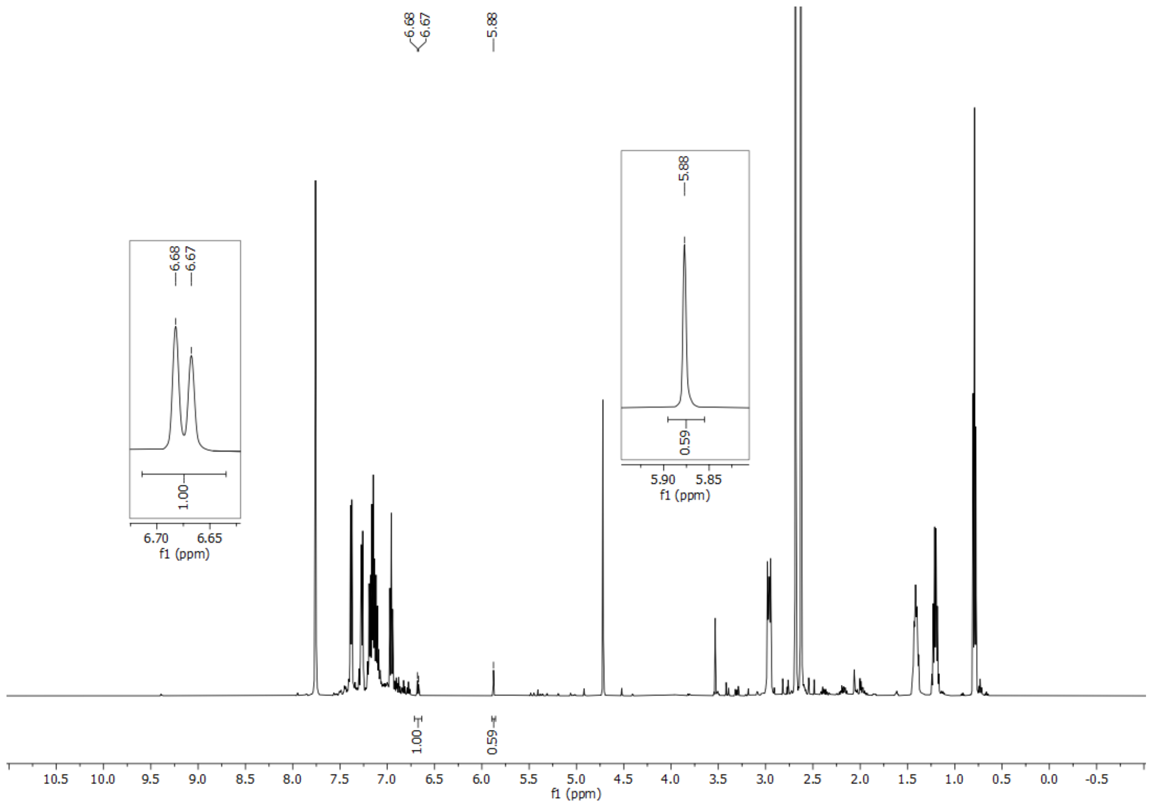

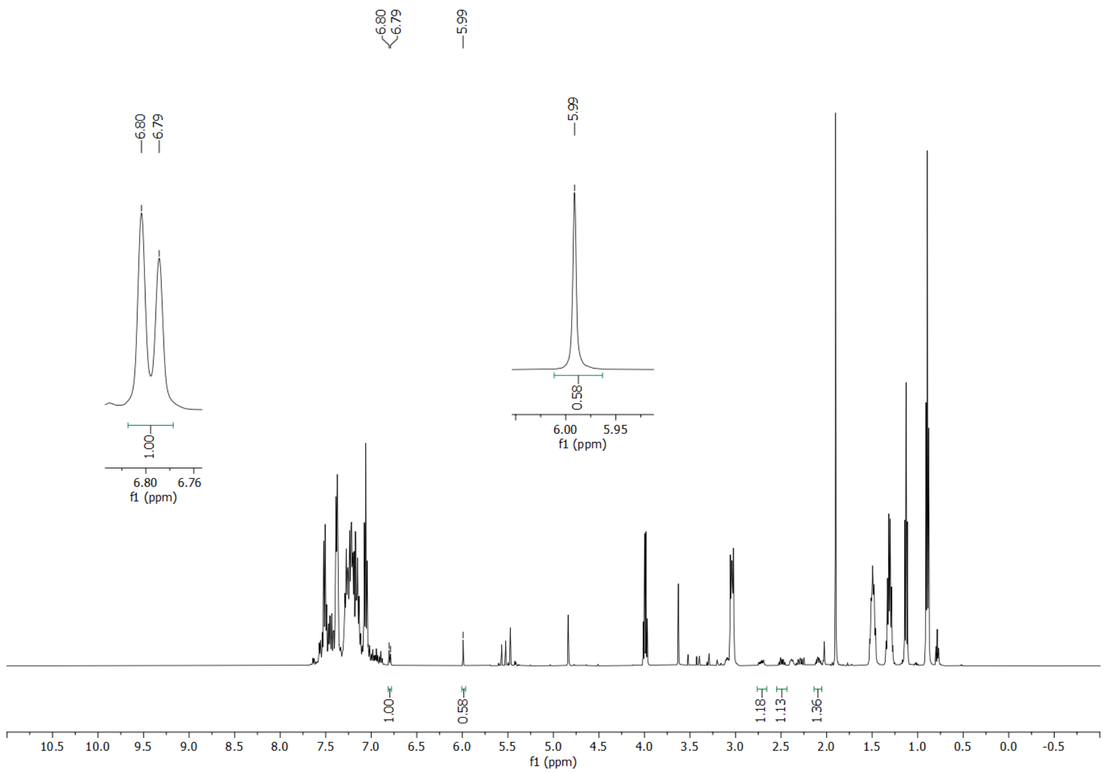

Figure S-42. *Top (from alcohol):* ^{1}H-NMR(500 MHz, $CDCl_3$) spectrum of reaction mixture after extraction containing **2n** and internal standard 1,3,5-trimethoxybenzene (7.6 mg). Quantitative NMR analysis resulted in 77% yield for **2n**. See sections 2.2 and 5 for details. *Bottom (from ketone):* ^{1}H-NMR(400 MHz, $CDCl_3$) spectrum of reaction mixture after extraction containing **2n** and internal standard 1,3,5-trimethoxybenzene (8.9 mg). Quantitative NMR analysis resulted in 91% yield for **2n**. See sections 2.2 and 5 for details.

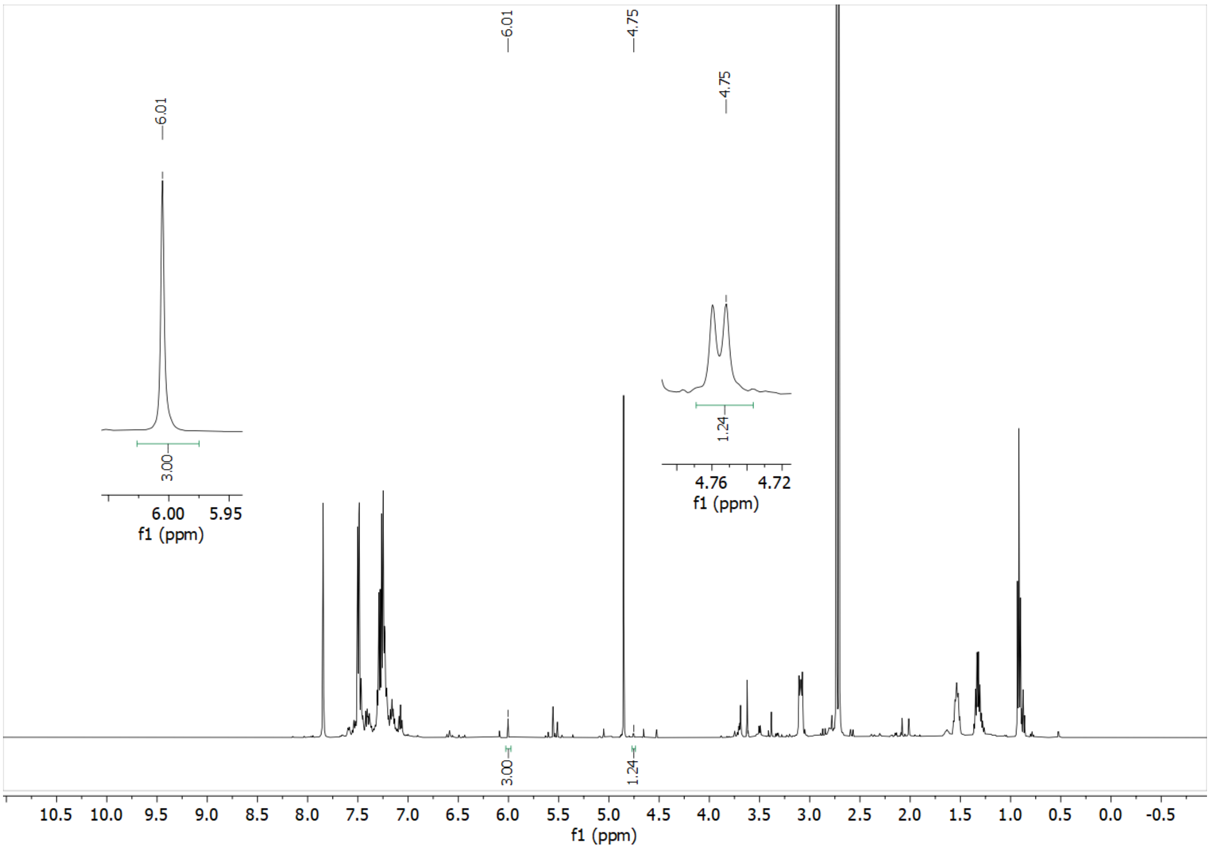

Figure S-43. ^{1}H-NMR(400 MHz, $CDCl_3$) spectrum of reaction mixture after extraction containing **2o** and internal standard 1,3,5-trimethoxybenzene (7.4 mg). Quantitative NMR analysis resulted in 16% yield for **2o**. See sections 2.2 and 5 for details.

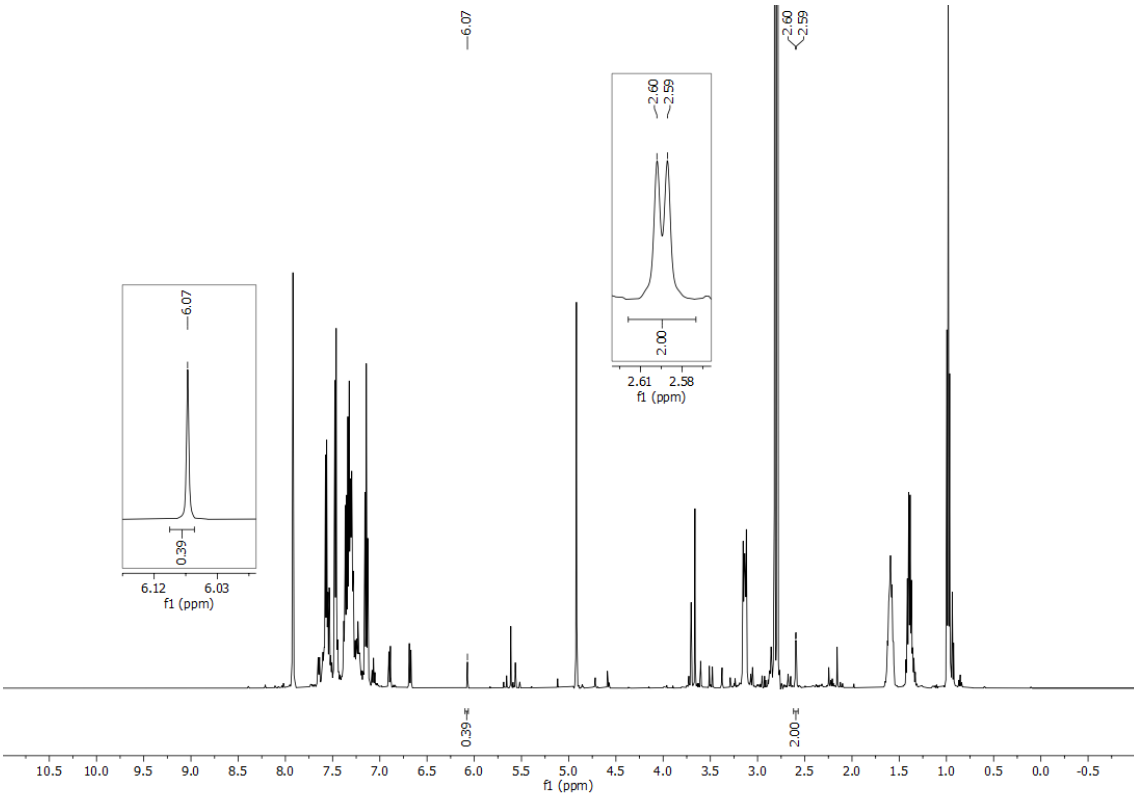

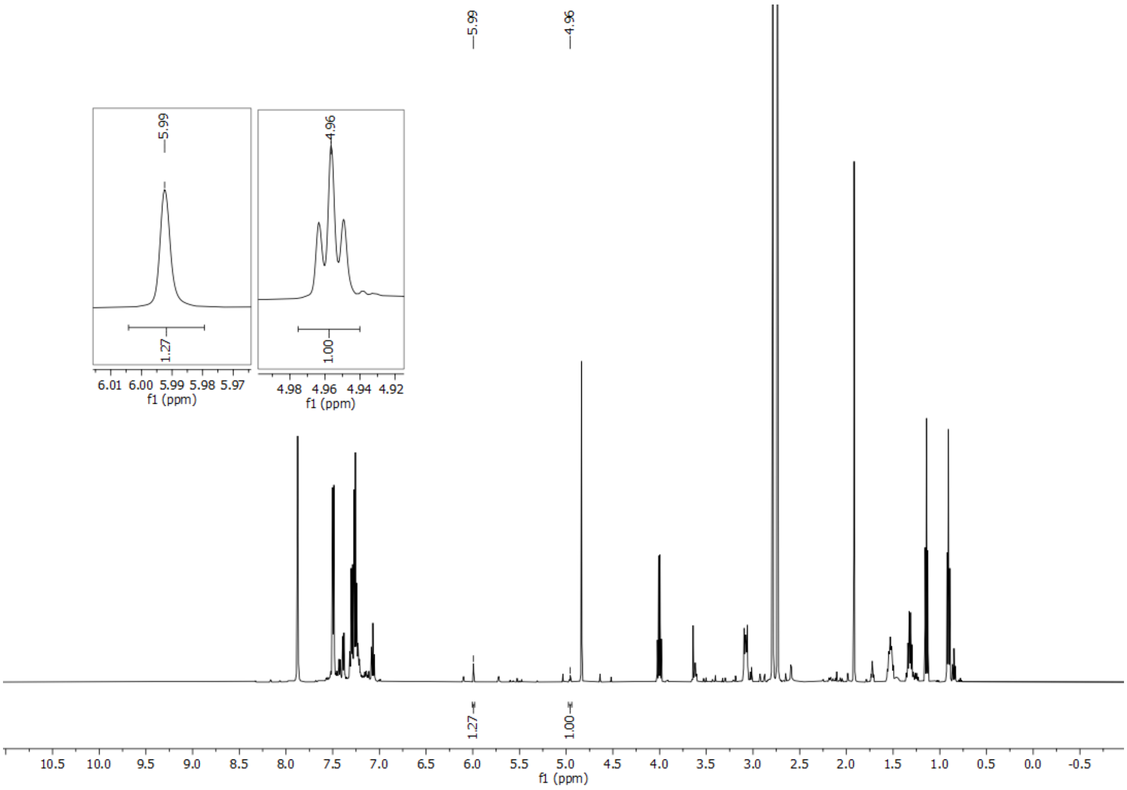

Figure S-44. *Top (from alcohol):* ^{1}H-NMR(500 MHz, $CDCl_3$) spectrum of reaction mixture after extraction containing **2p** and internal standard 1,3,5-trimethoxybenzene (7.6 mg). Quantitative NMR analysis resulted in 37% yield for **2p**. See sections 2.2 and 5 for details. *Bottom: from respective ketone* ^{1}H-NMR(500 MHz, $CDCl_3$) spectrum of reaction mixture after extraction containing **2p** and internal standard 1,3,5-trimethoxybenzene (8.2 mg). Quantitative NMR analysis resulted in 58% yield for **2p**. See sections 2.2 and 5 for details.

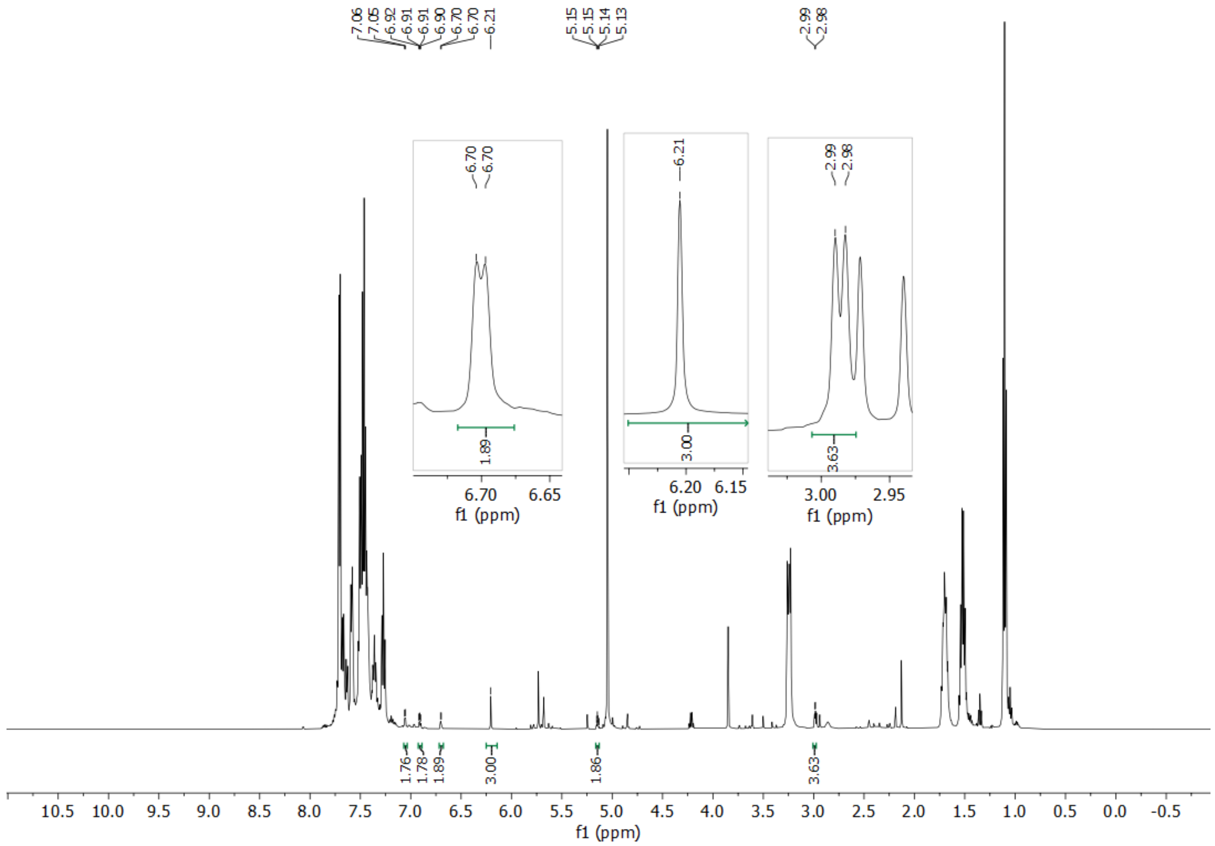

Figure S-45. ^{1}H-NMR(400 MHz, $CDCl_3$) spectrum of reaction mixture after extraction containing **2q** and internal standard 1,3,5-trimethoxybenzene (8.2 mg). Quantitative NMR analysis resulted in 38% yield for **2q**. See sections 2.2 and 5 for details.

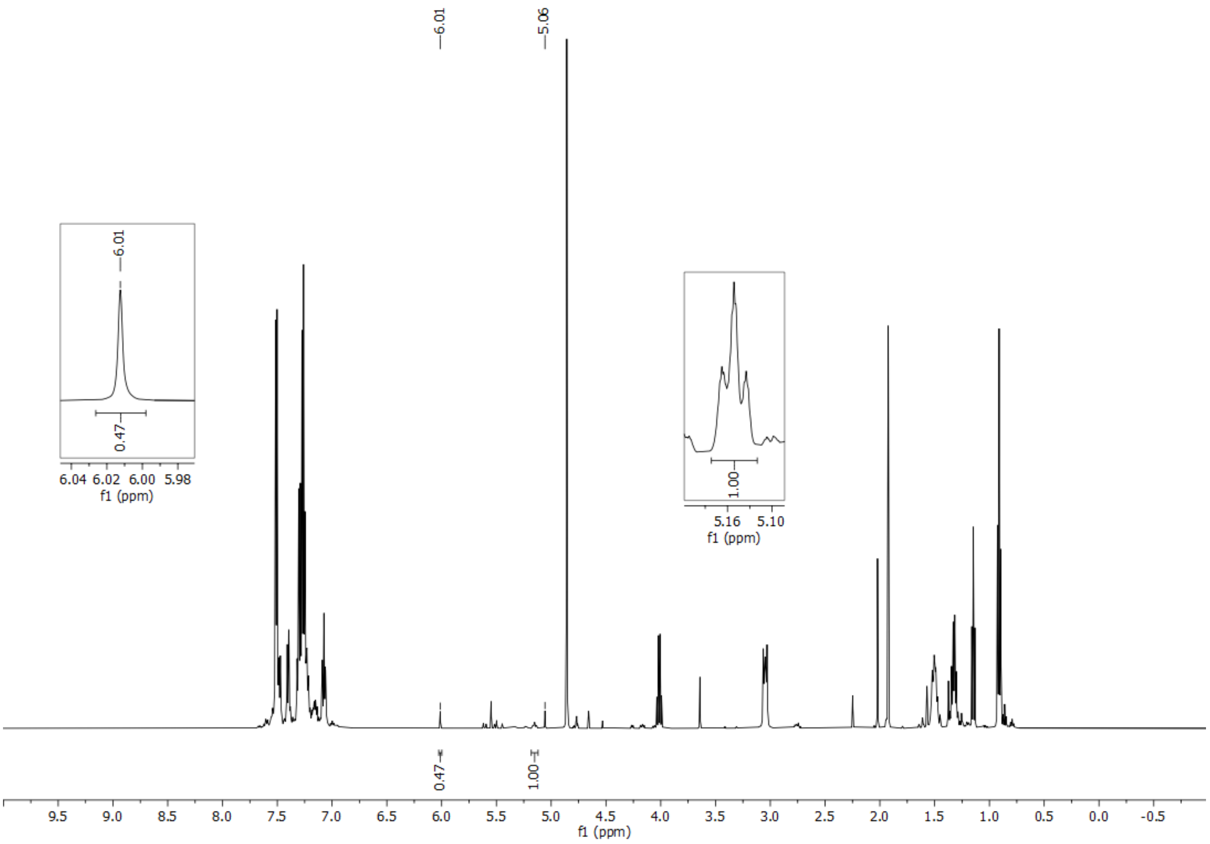

Figure S-46. ^{1}H-NMR(400 MHz, $CDCl_3$) spectrum of reaction mixture after extraction containing **2r** and internal standard 1,3,5-trimethoxybenzene (4.2 mg). Quantitative NMR analysis resulted in 53% yield for **2r**. See sections 2.2 and 5 for details.

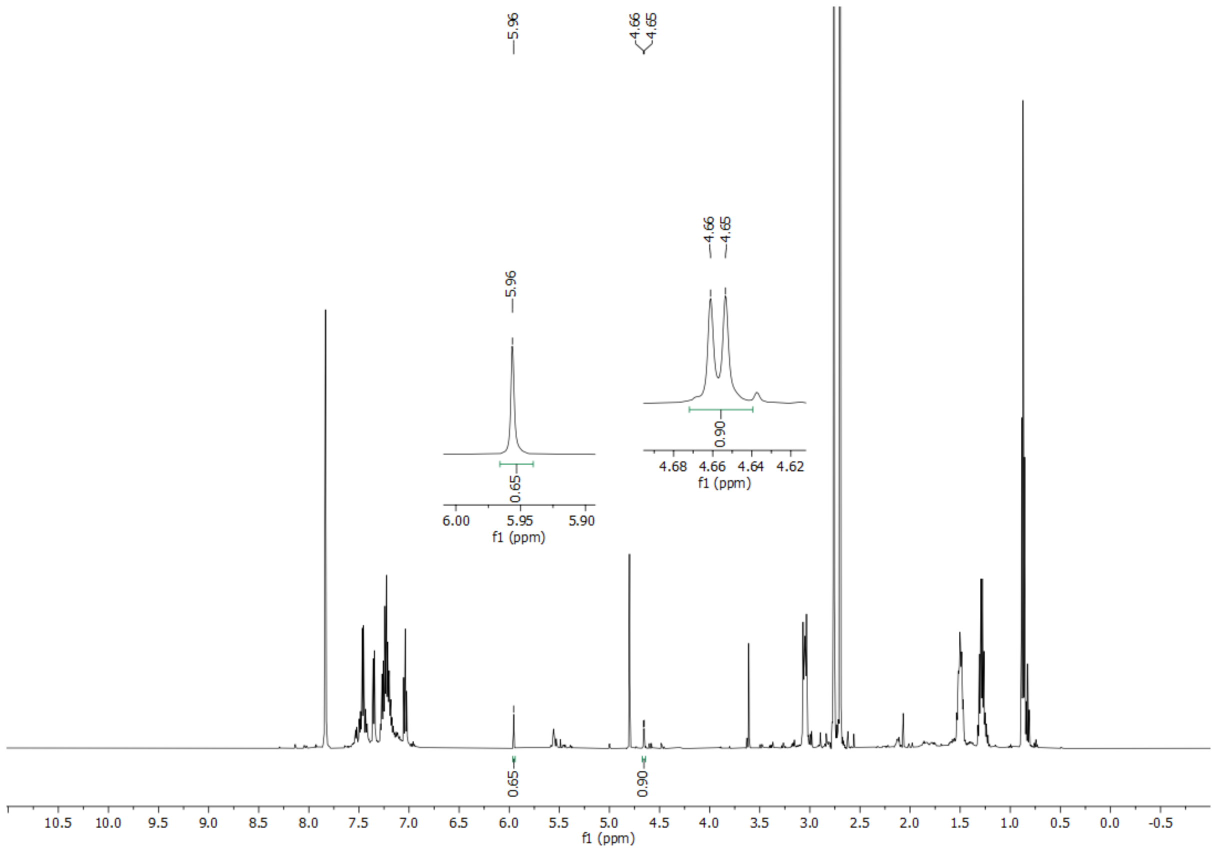

Figure S-47. ^{1}H-NMR(400 MHz, $CDCl_3$) spectrum of reaction mixture after extraction containing **2s** and internal standard 1,3,5-trimethoxybenzene (7.0 mg). Quantitative NMR analysis resulted in 56% yield for **2s**. See sections 2.2 and 5 for details.

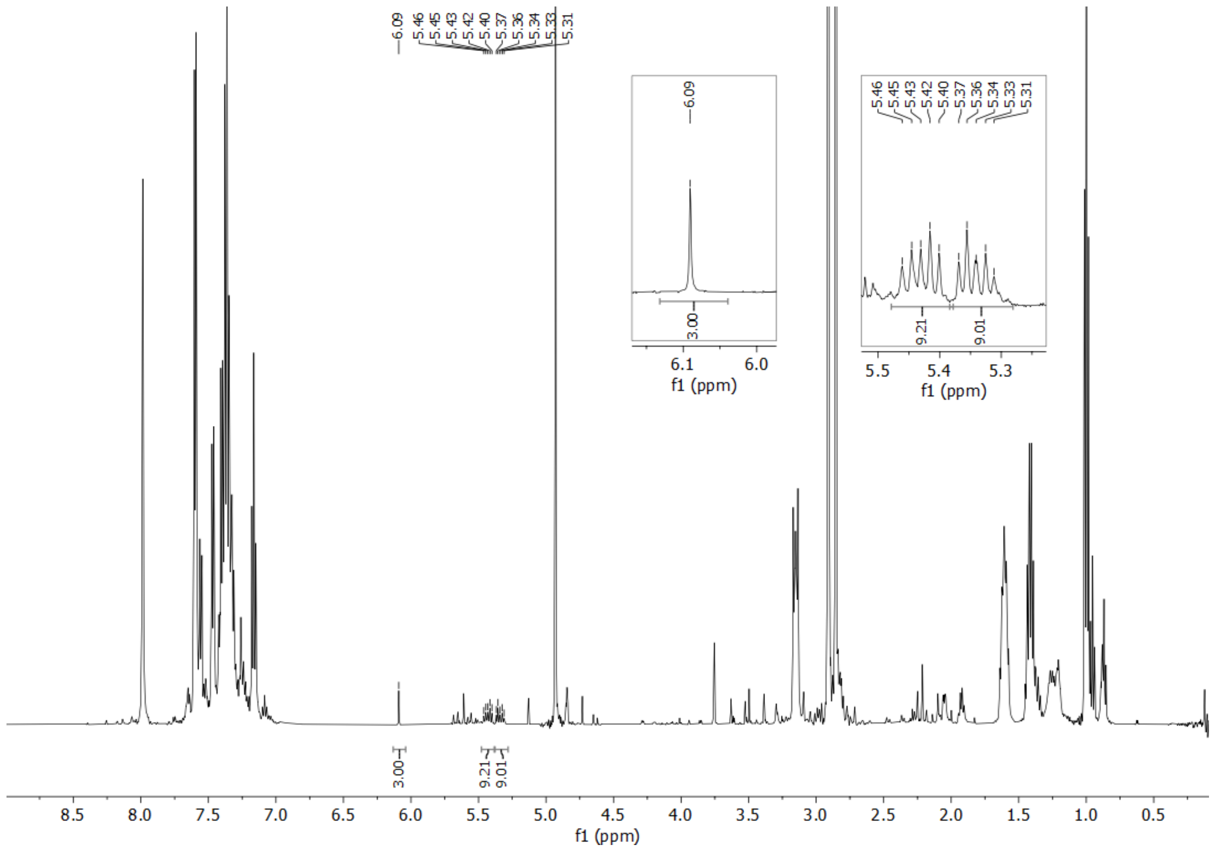

Figure S-48. ^{1}H-NMR(500 MHz, $CDCl_3$) spectrum of reaction mixture after extraction containing **2u** and internal standard 1,3,5-trimethoxybenzene (3.8 mg). Quantitative NMR analysis resulted in 67% yield for **2u**. See sections 2.2 and 5 for details.

15. ^{1}H-NMR and ^{13}C-NMR spectra for compounds 2a-2s and silanols

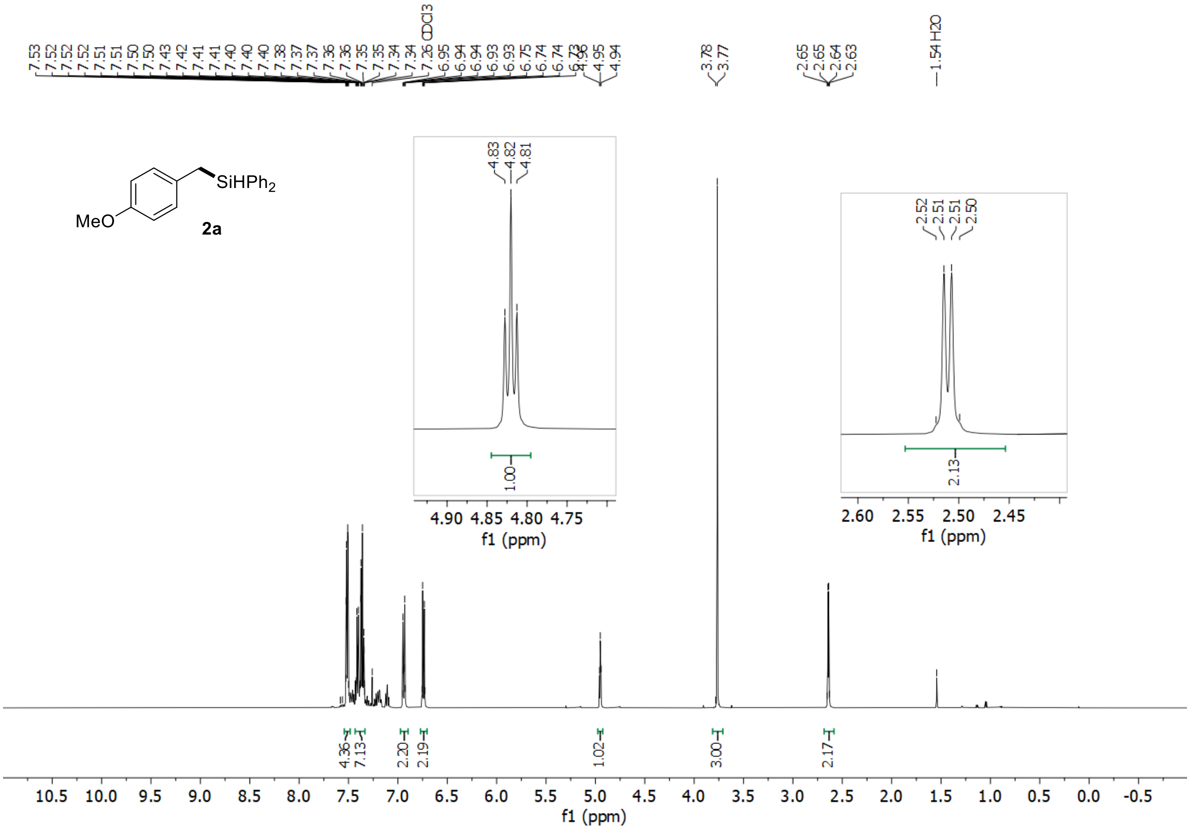

Figure S-49. ^{1}H-NMR (500 MHz, $CDCl_3$) of **2a**.

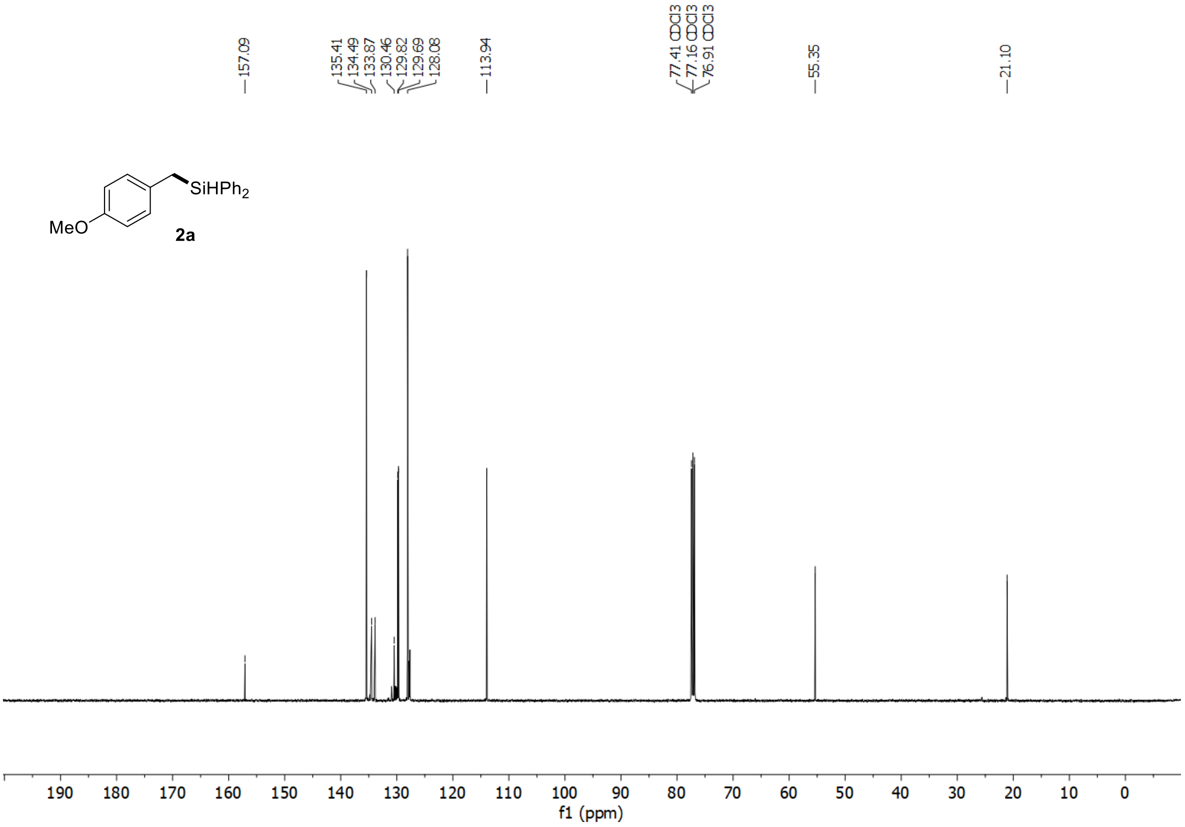

Figure S-50. ^{13}C-NMR (126 MHz, $CDCl_3$) of **2a**.

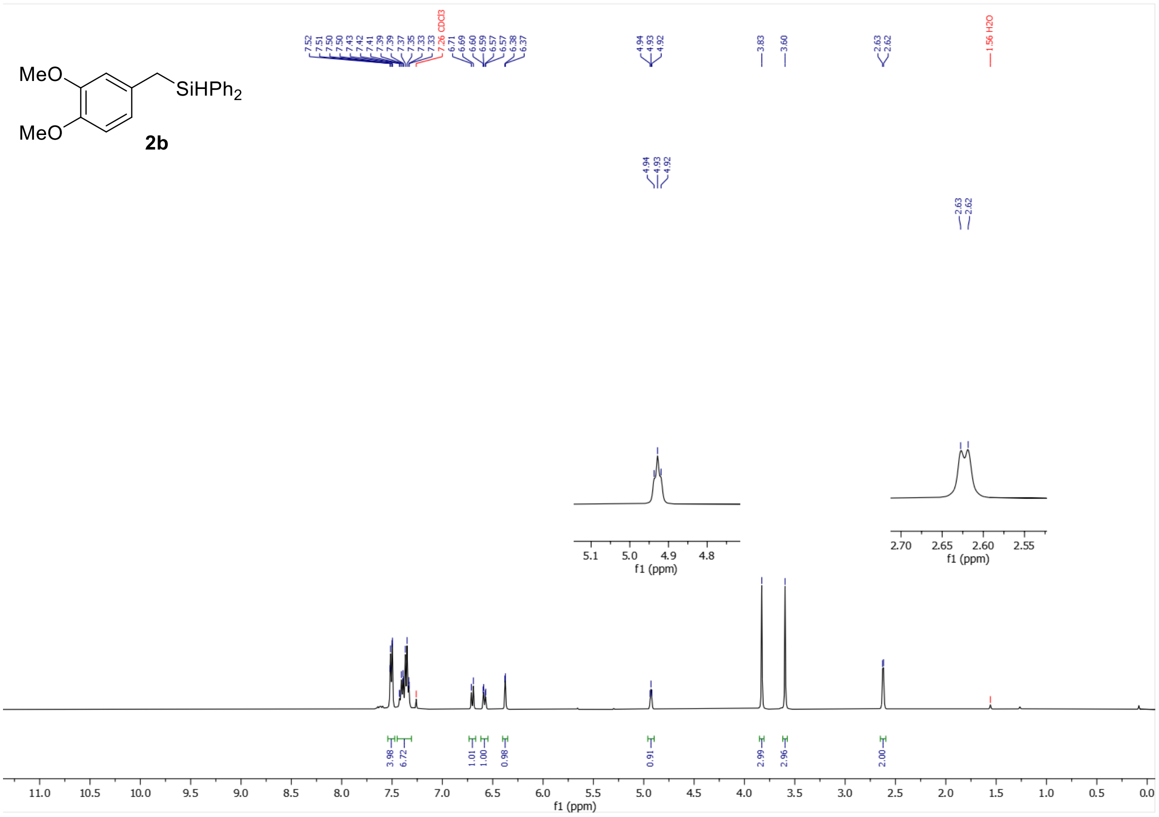

Figure S-51. ^{1}H-NMR (500 MHz, $CDCl_3$) of **2b**.

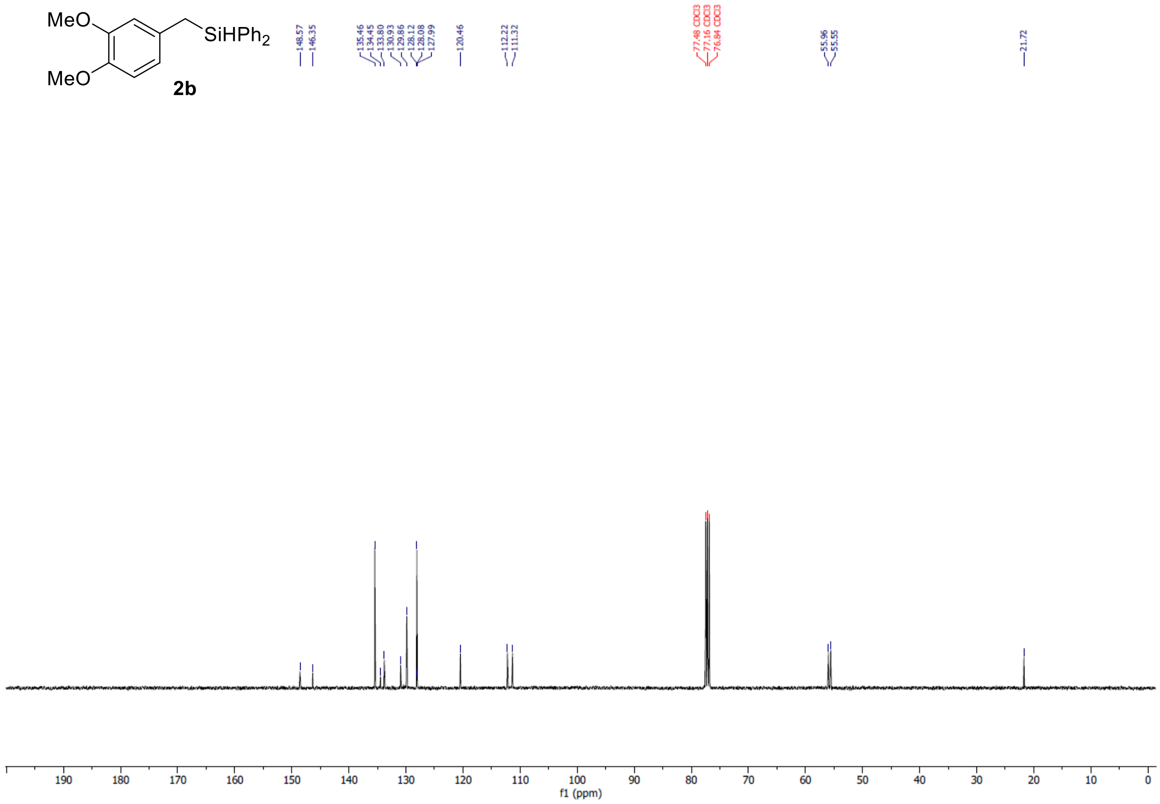

Figure S-52. ^{13}C-NMR (126 MHz, $CDCl_3$) of **2b**.

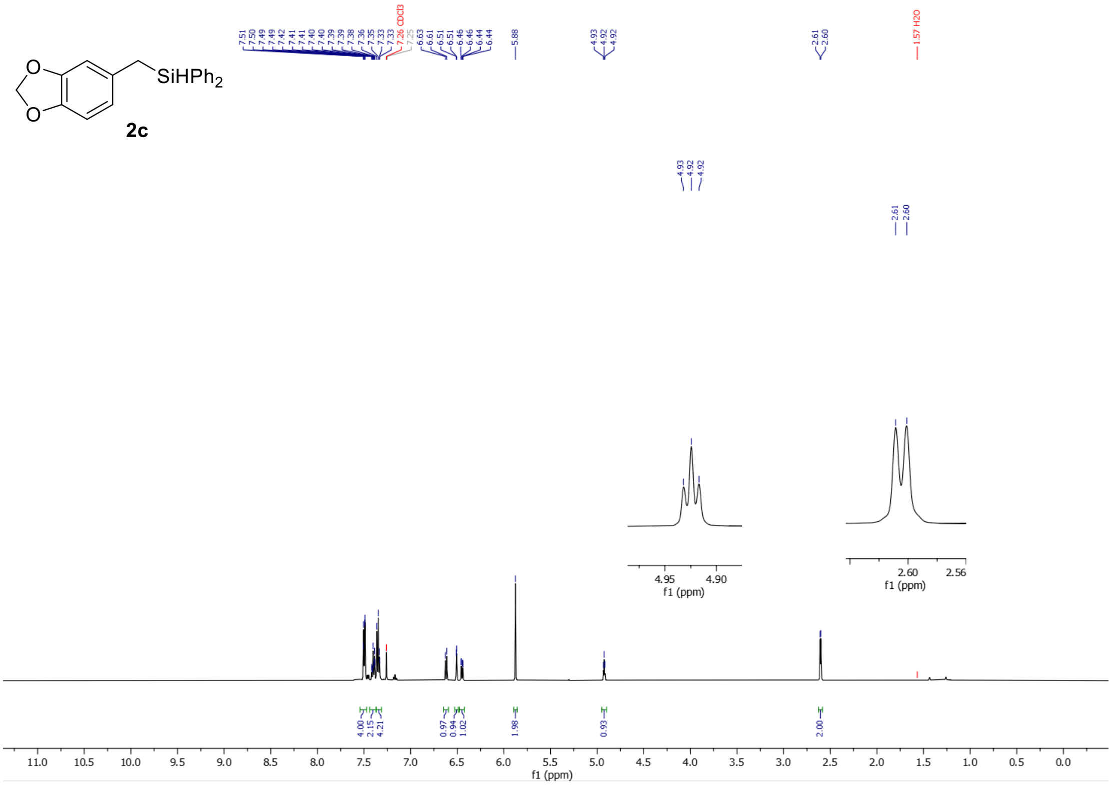

Figure S-53. ^{1}H-NMR (500 MHz, $CDCl_3$) of **2c**.

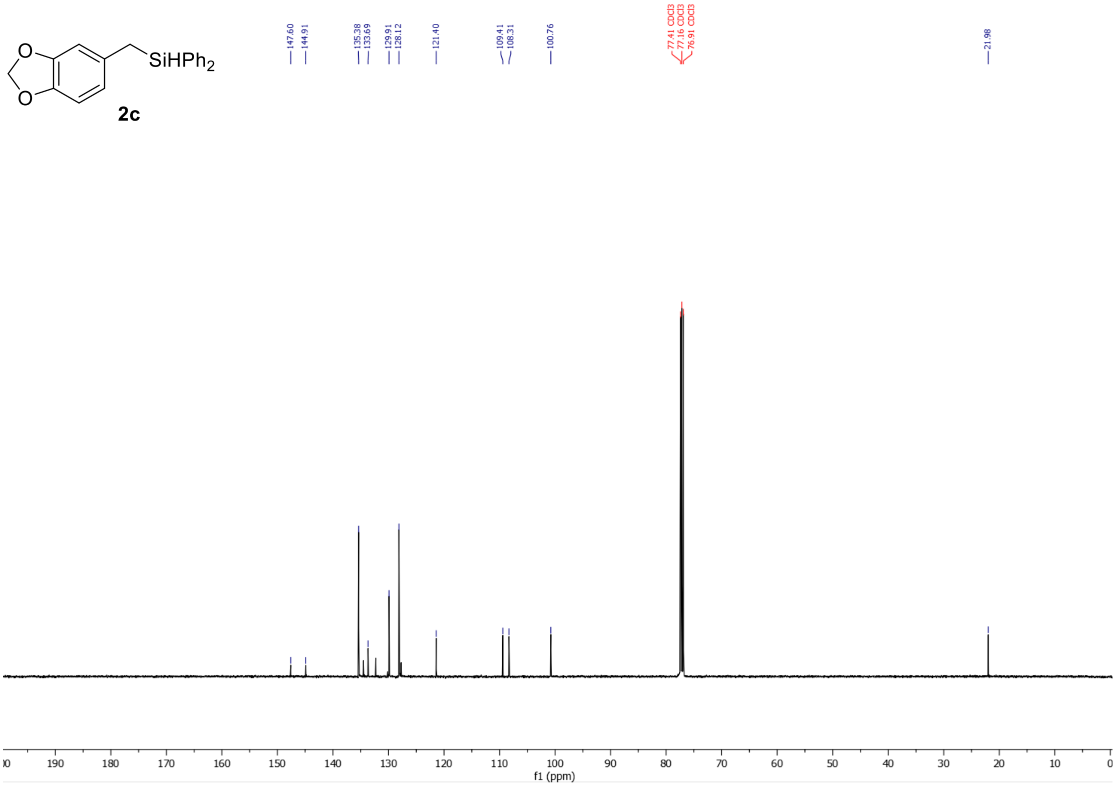

Figure S-54. ^{13}C-NMR (126 MHz, $CDCl_3$) of **2c**.

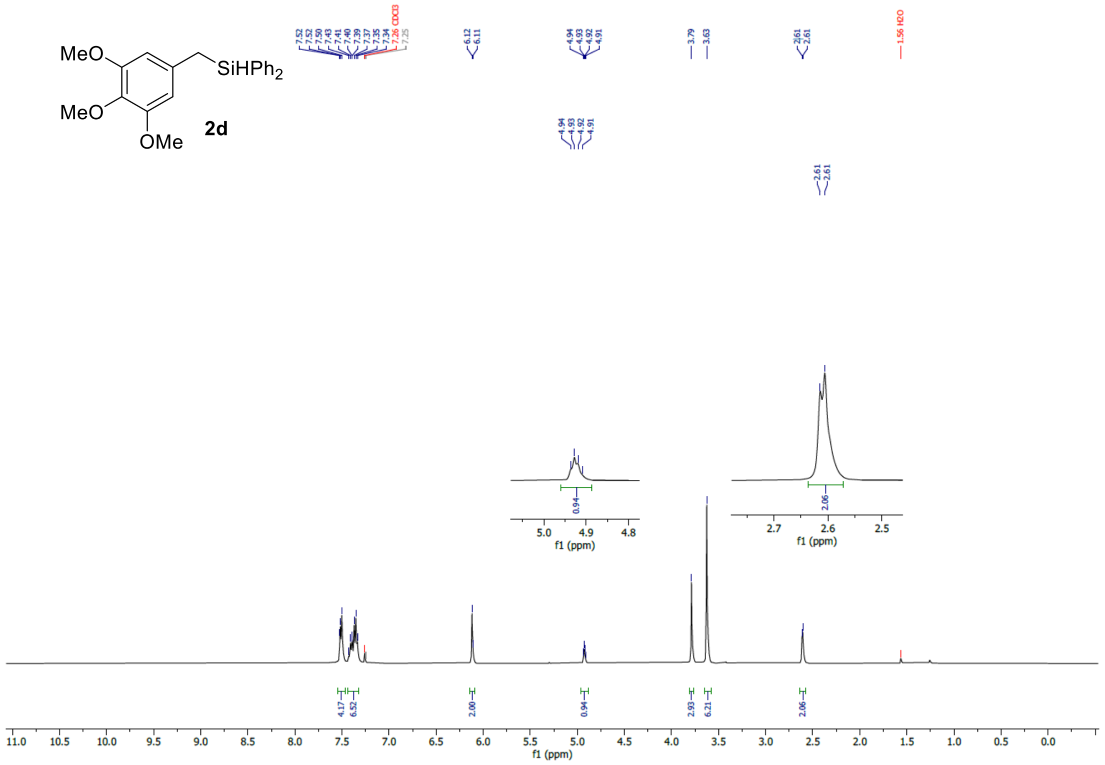

Figure S-55. ^{1}H-NMR (400 MHz, $CDCl_3$) of **2d**.

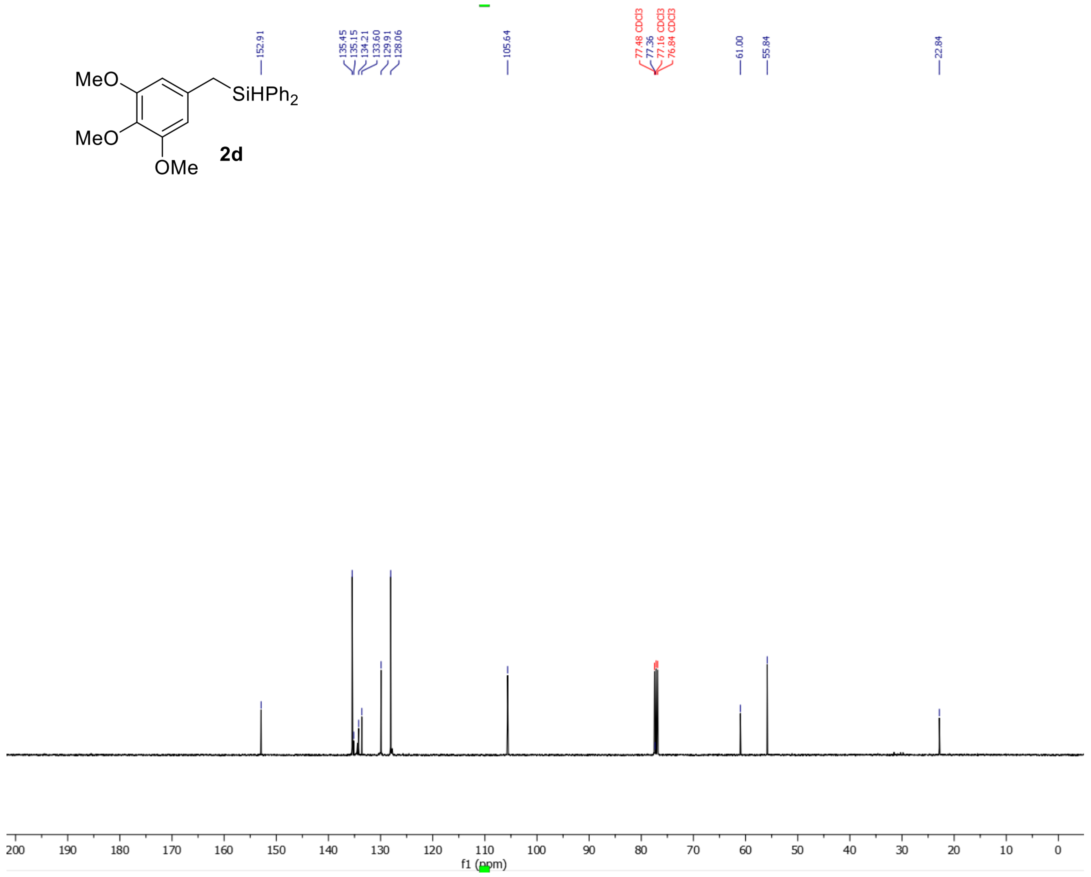

Figure S-56. ^{13}C-NMR (101 MHz, $CDCl_3$) of **2d**.

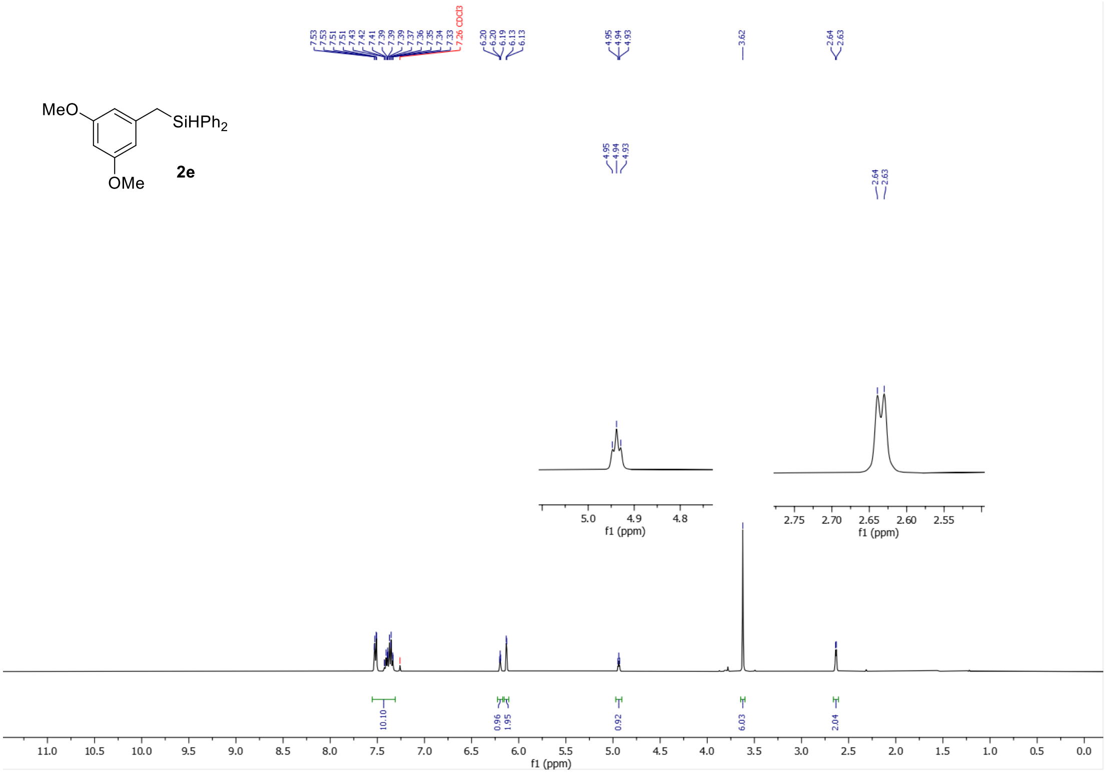

Figure S-57. ^{1}H-NMR (400 MHz, $CDCl_3$) of **2e**.

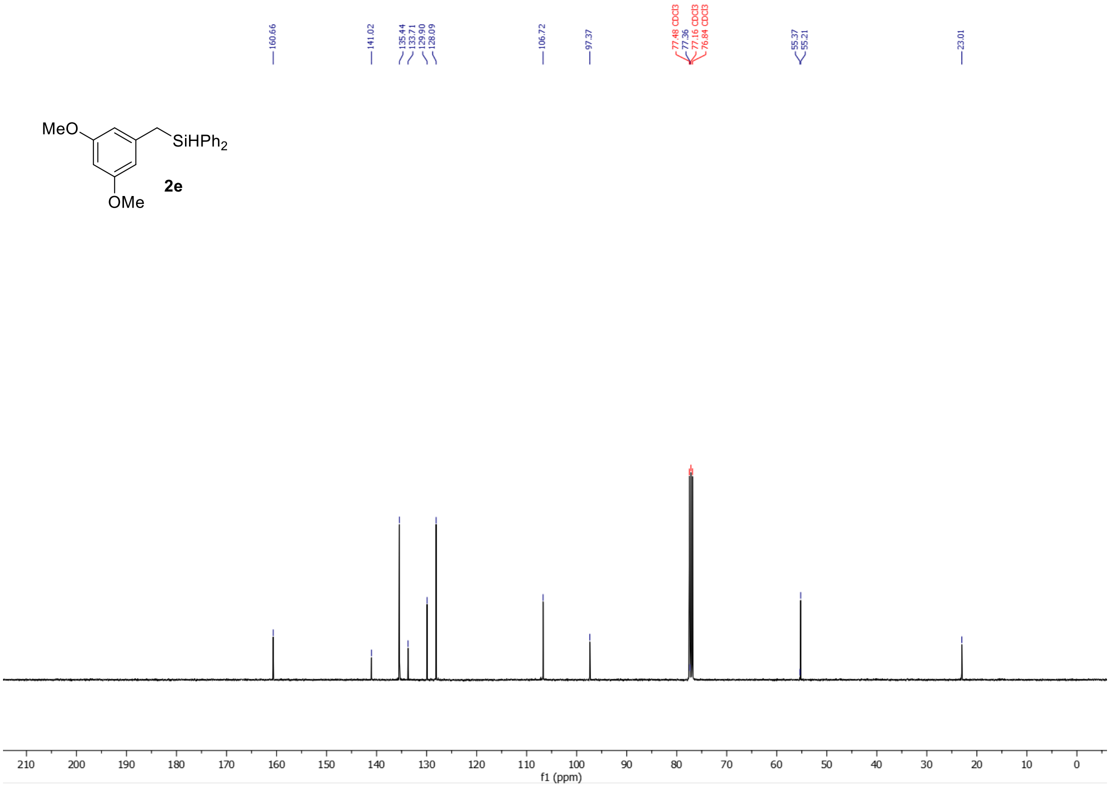

Figure S-58. ^{13}C-NMR (101 MHz, $CDCl_3$) of **2d**.

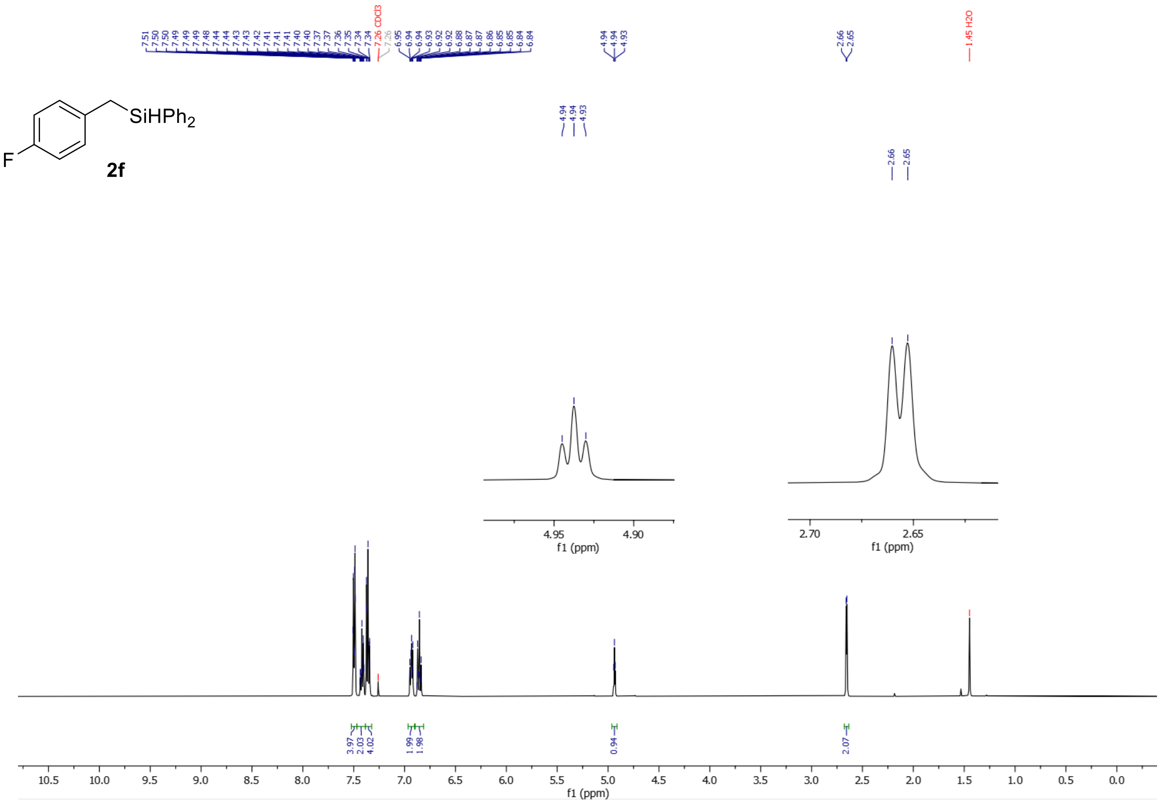

Figure S-59. ^{1}H-NMR (500 MHz, $CDCl_3$) of **2f**.

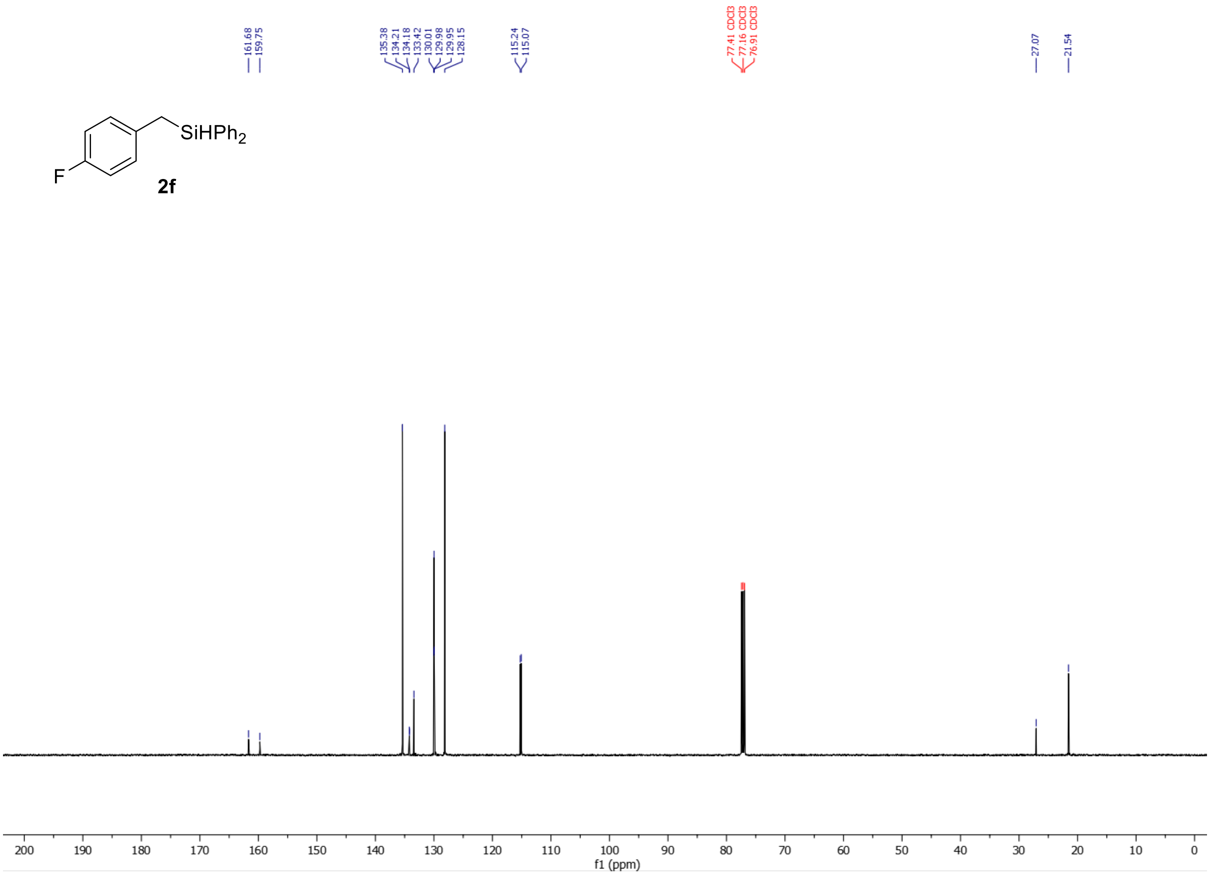

Figure S-60. ^{13}C-NMR (101 MHz, $CDCl_3$) of **2f**.

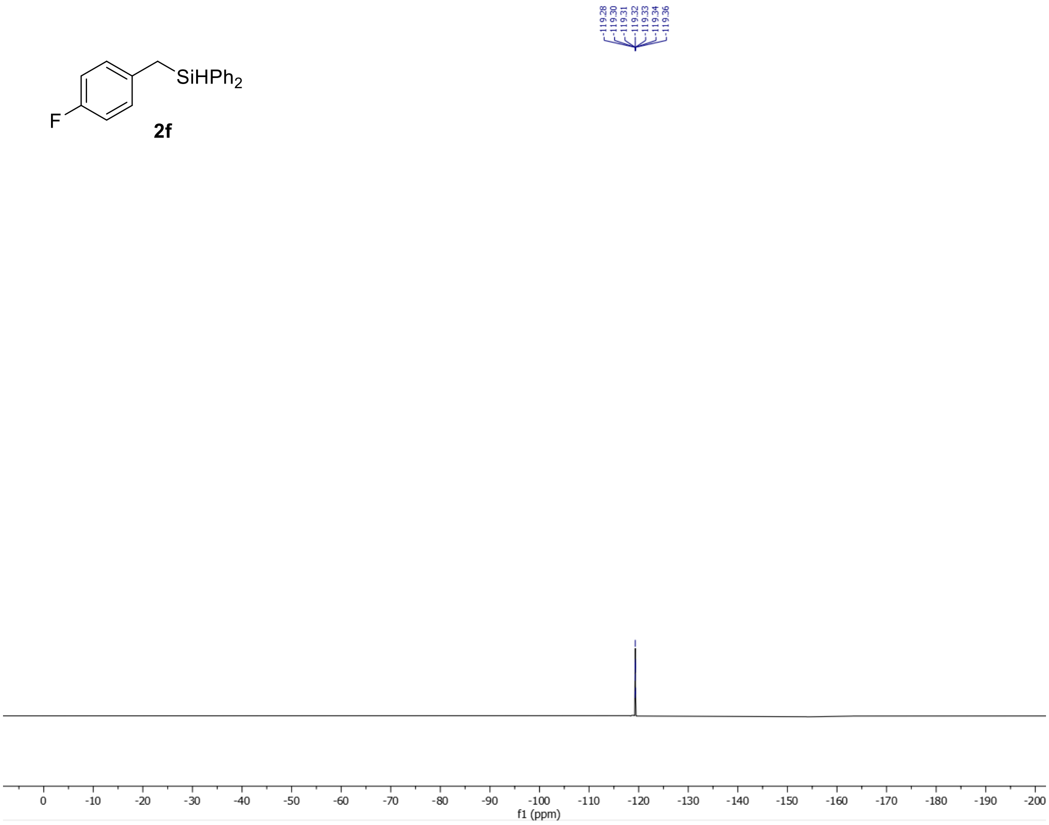

Figure S-61. ^{19}F-NMR (377 MHz, $CDCl_3$) of **2f**.

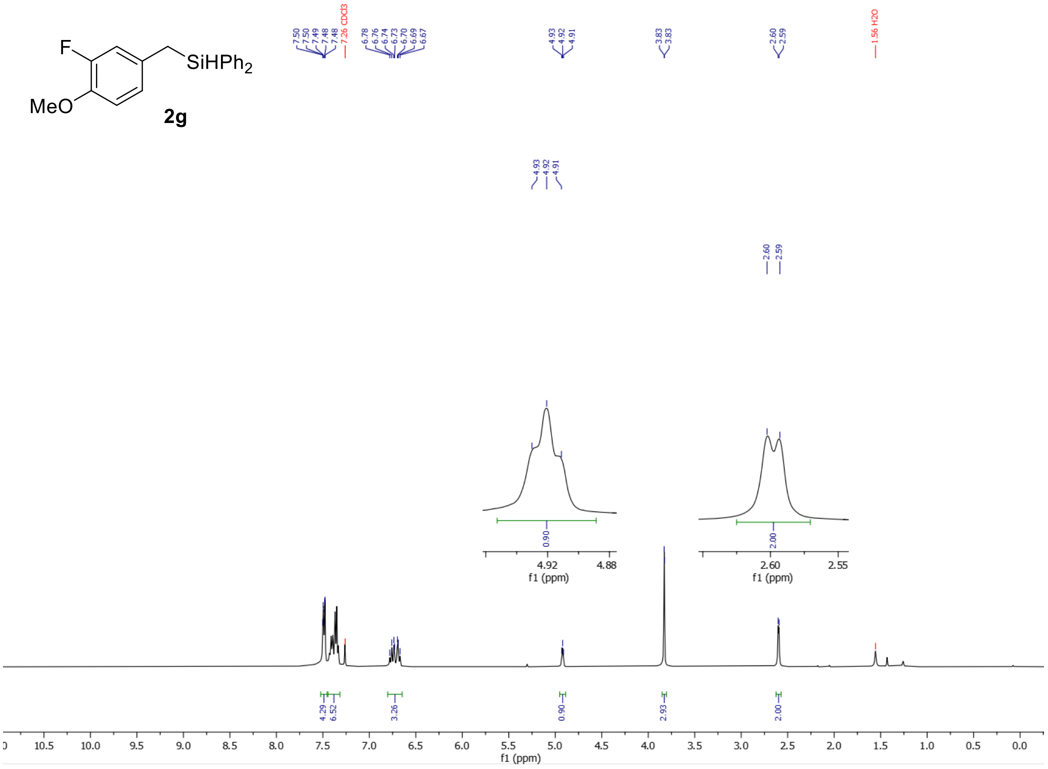

Figure S-62. ^{1}H-NMR (400 MHz, $CDCl_3$) of **2g**.

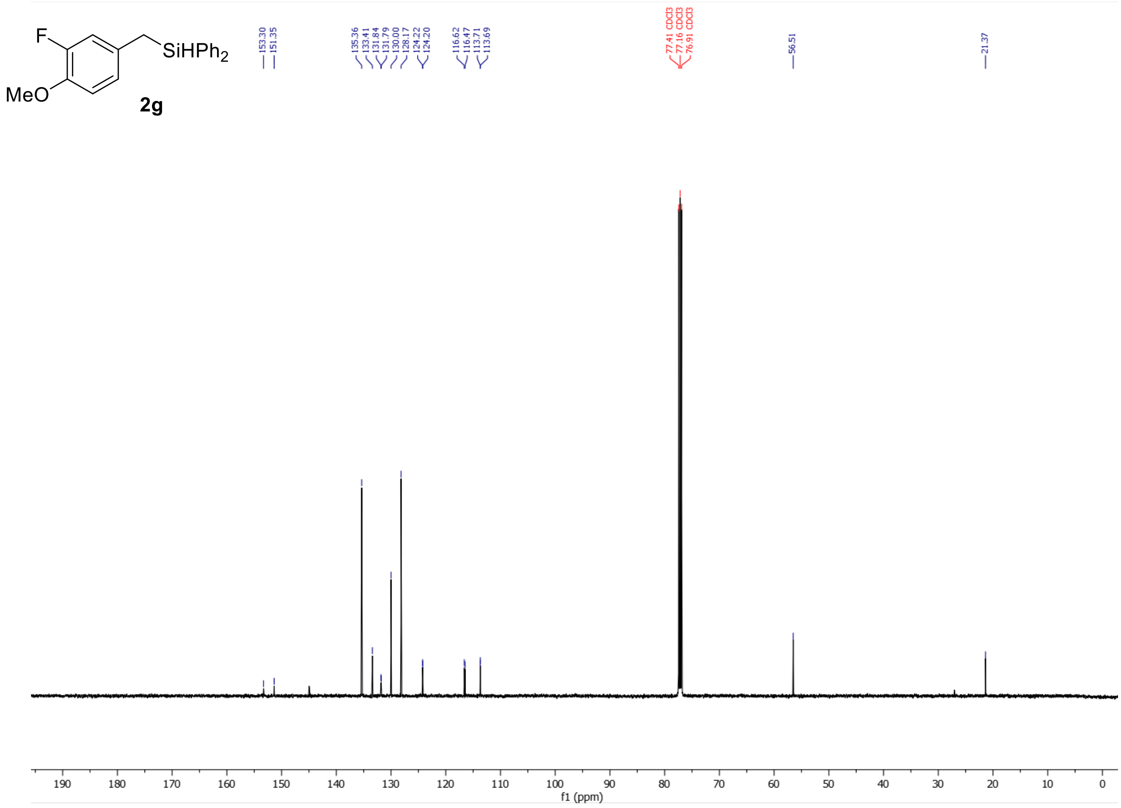

Figure S-63. ^{13}C-NMR (101 MHz, CDCl$_3$) of **2g**.

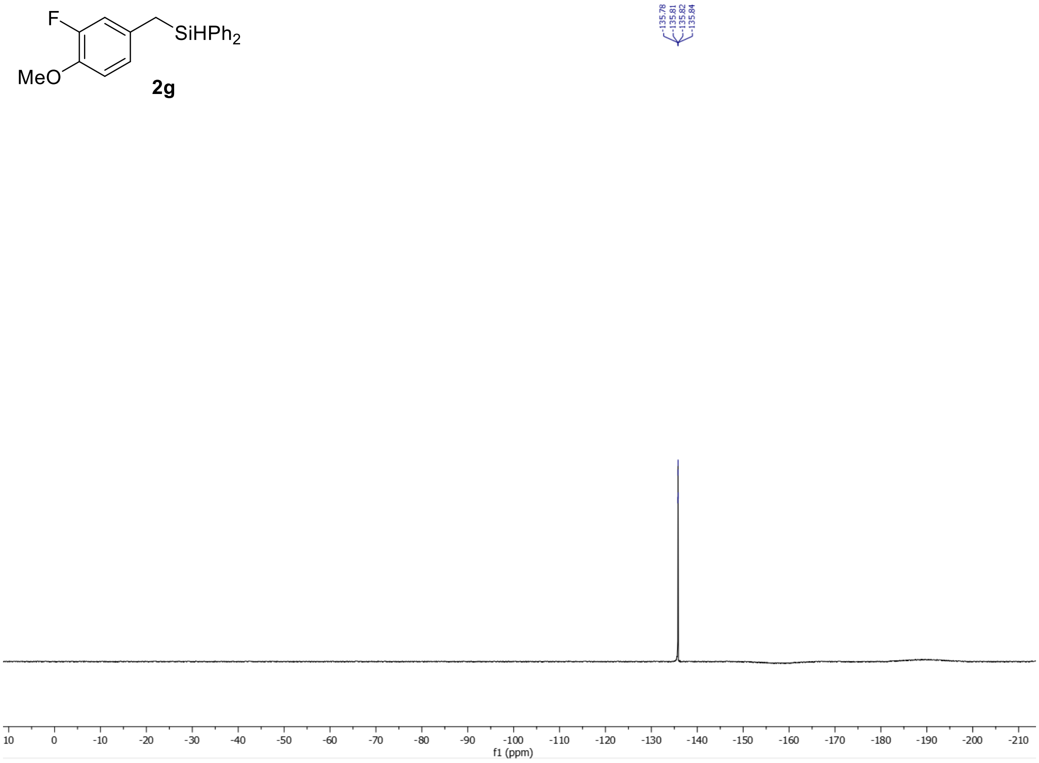

Figure S-64. ^{19}F-NMR (377 MHz, CDCl$_3$) of **2g**.

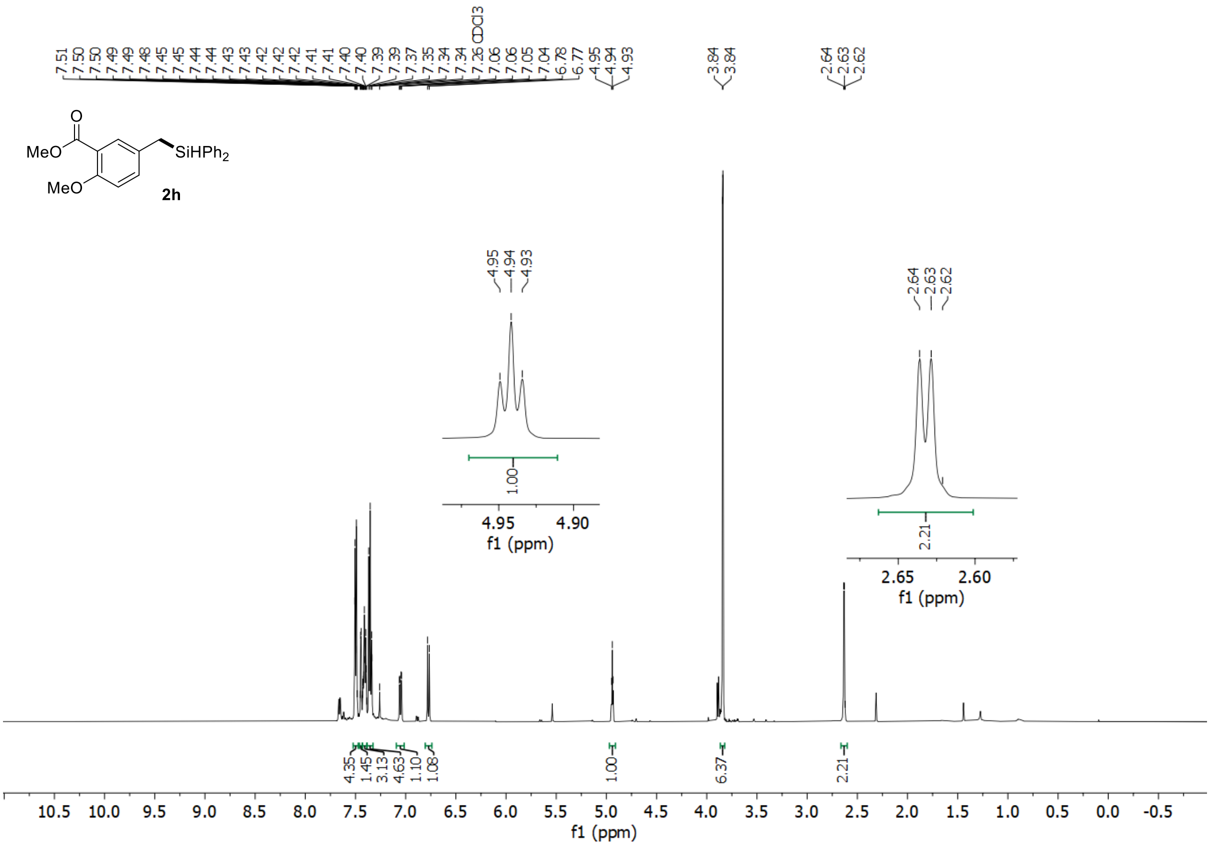

Figure S-65. ^{1}H-NMR (500 MHz, $CDCl_3$) of **2h**.

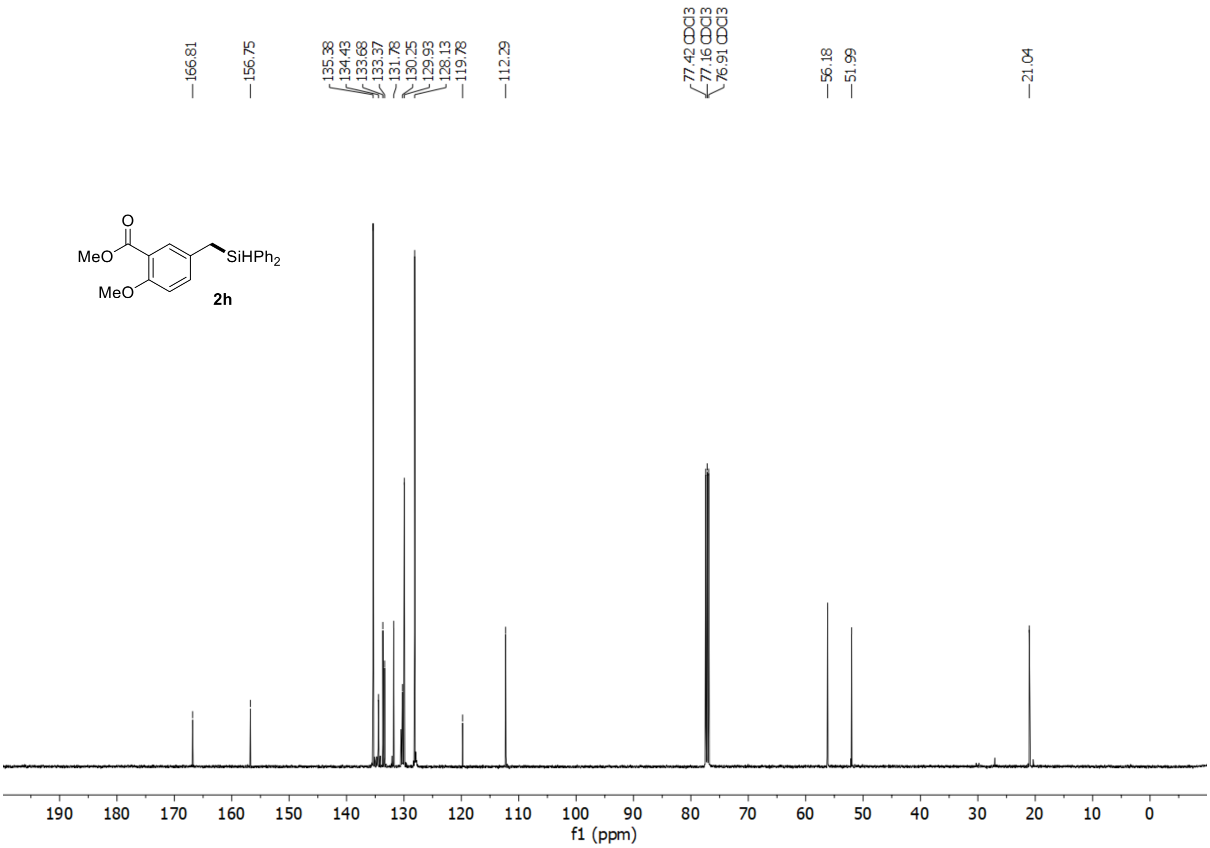

Figure S-66. ^{13}C-NMR (126 MHz, $CDCl_3$) of **2h**.

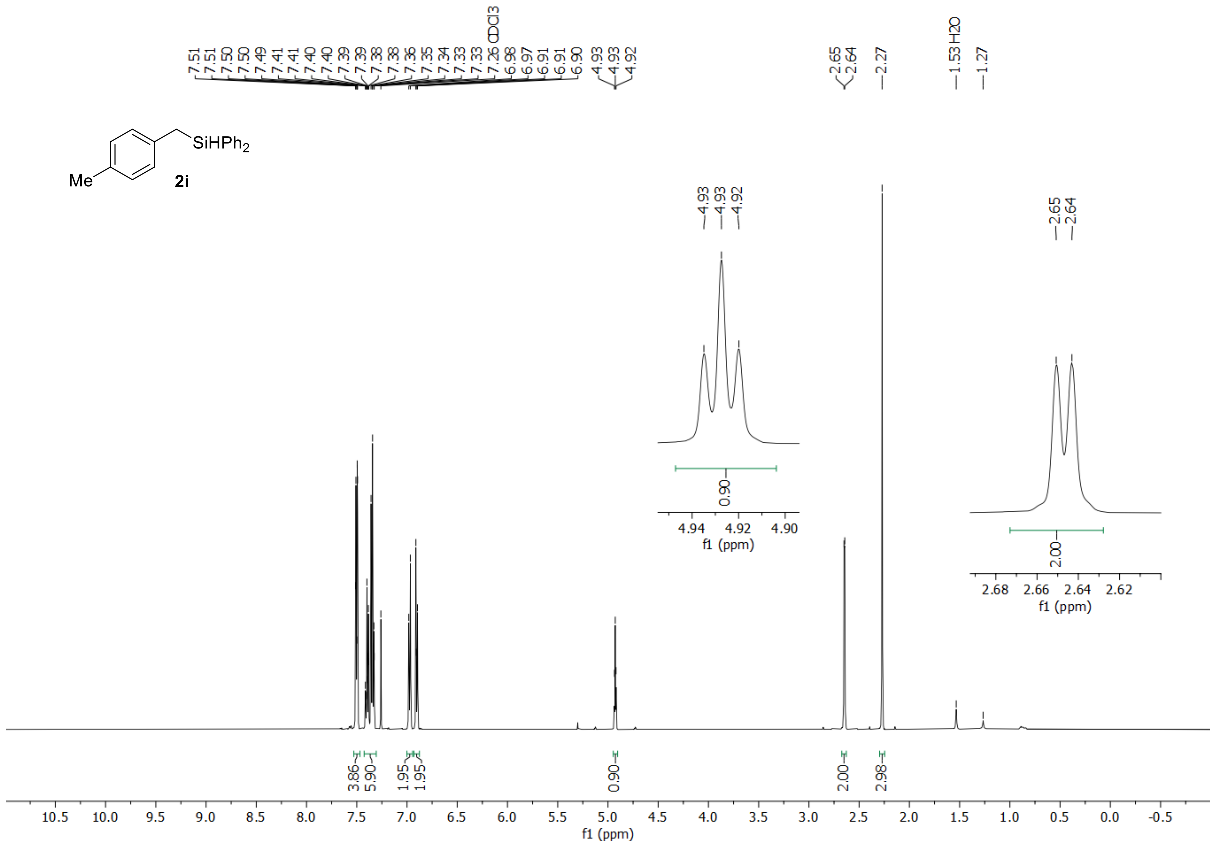

Figure S-67. ^{1}H-NMR (500 MHz, $CDCl_3$) of **2i**.

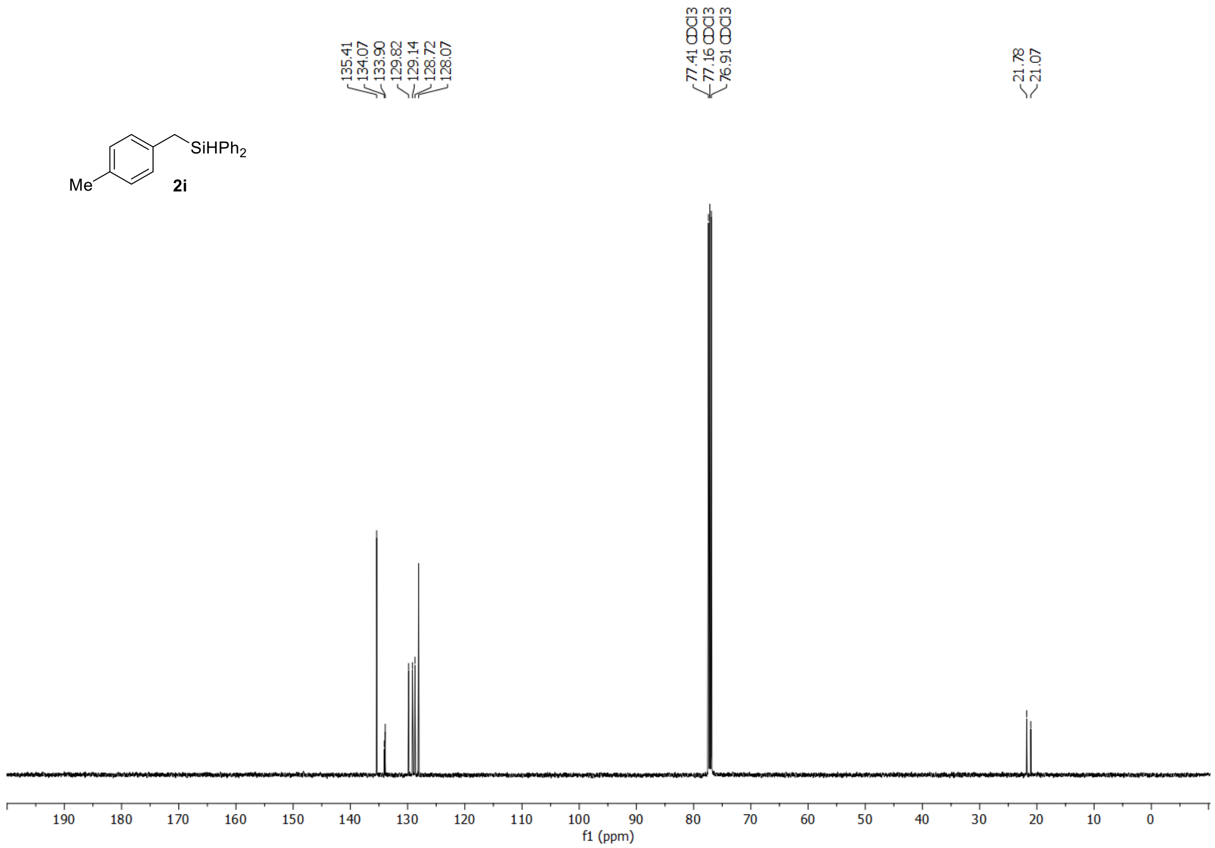

Figure S-68. ^{13}C-NMR (126 MHz, $CDCl_3$) of **2i**.

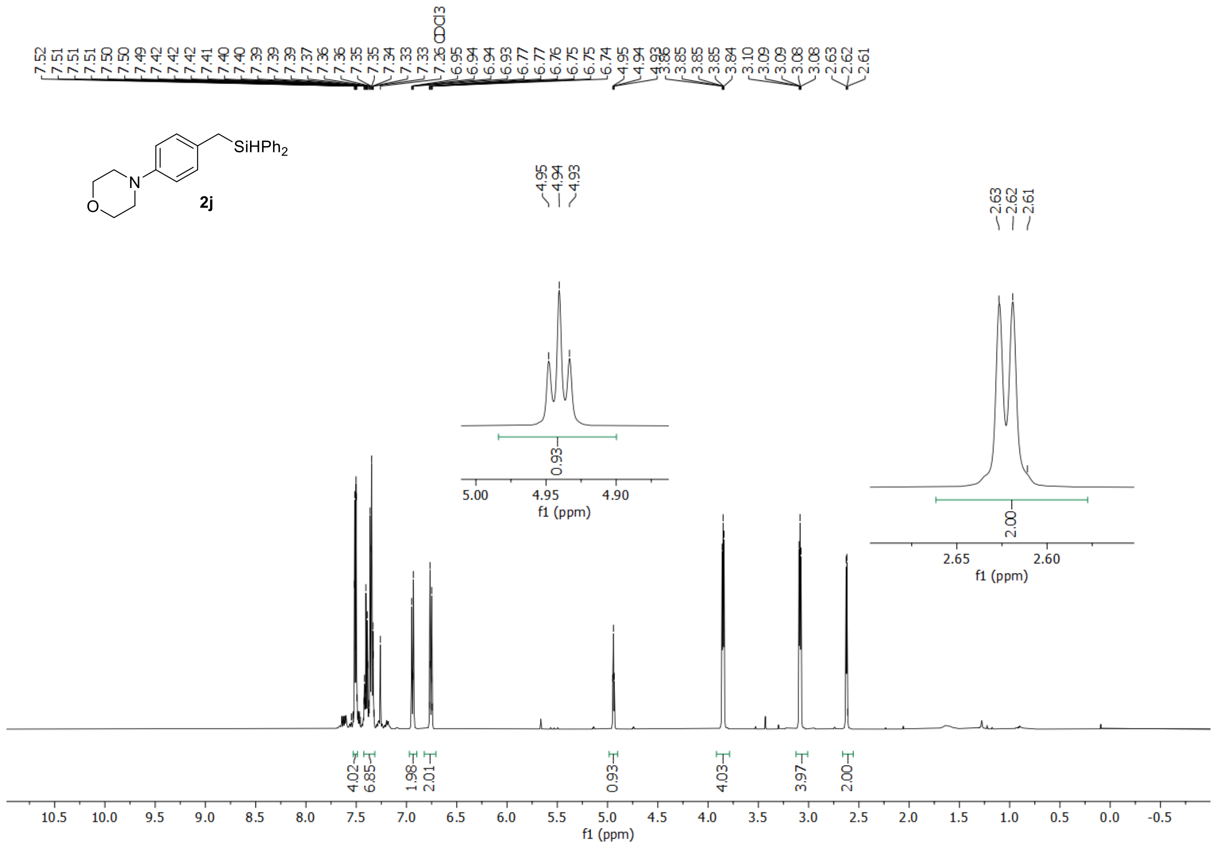

Figure S-69. ^{1}H-NMR (500 MHz, $CDCl_3$) of **2j**.

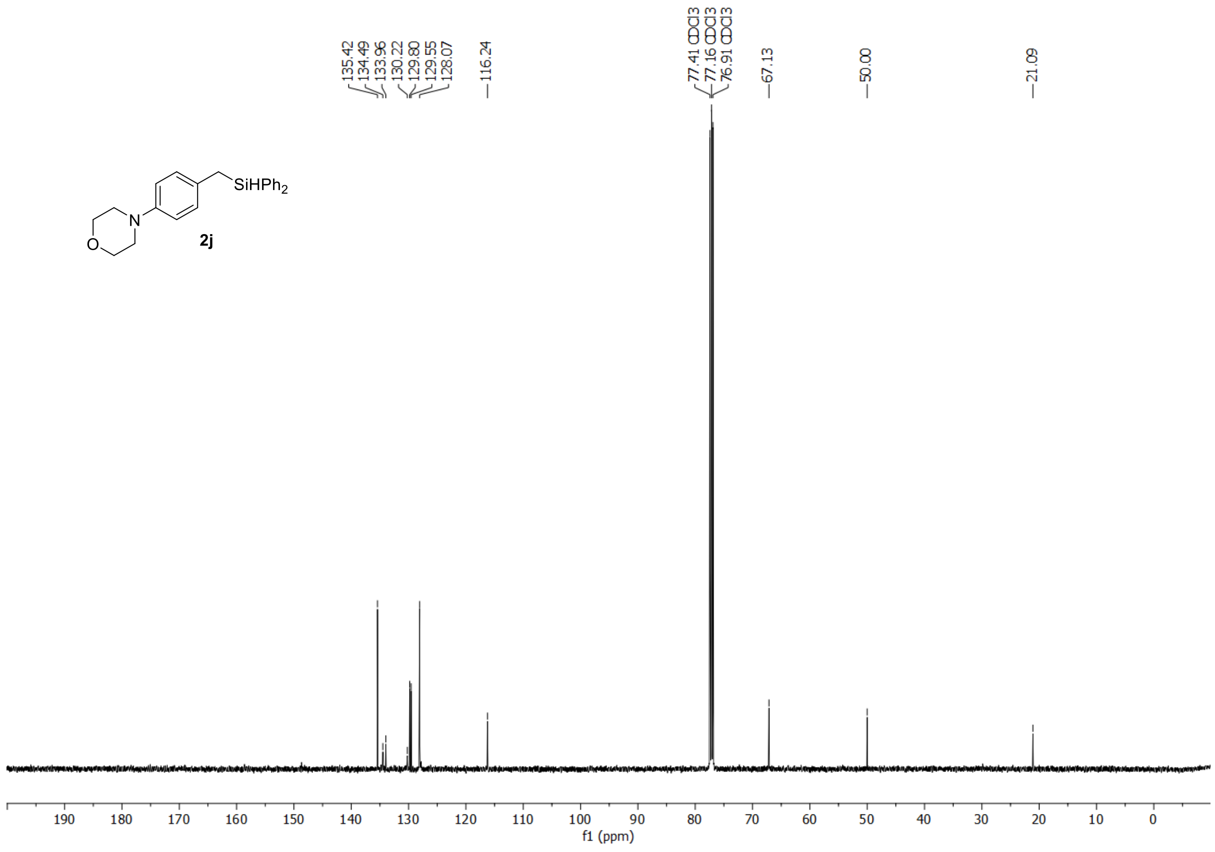

Figure S-70. ^{13}C-NMR (126 MHz, $CDCl_3$) of **2j**.

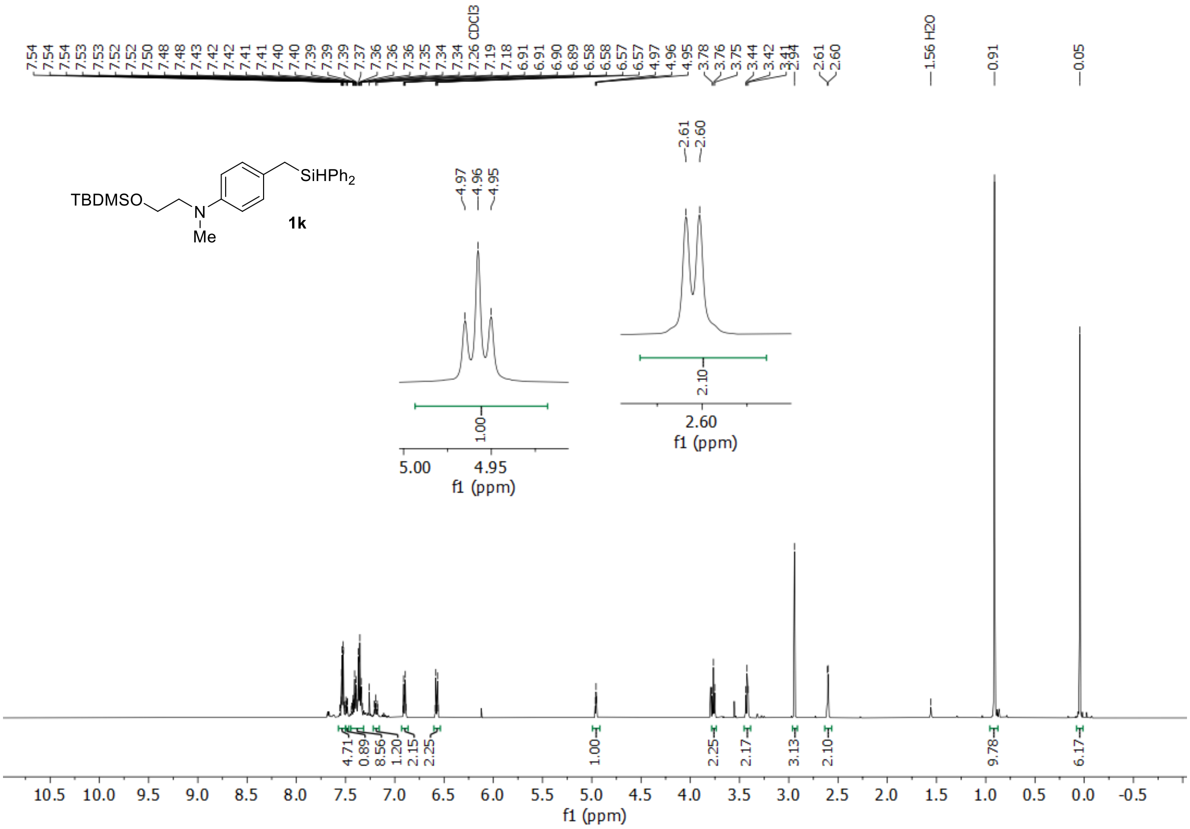

Figure S-71. ^{1}H-NMR (500 MHz, $CDCl_3$) of **2k**.

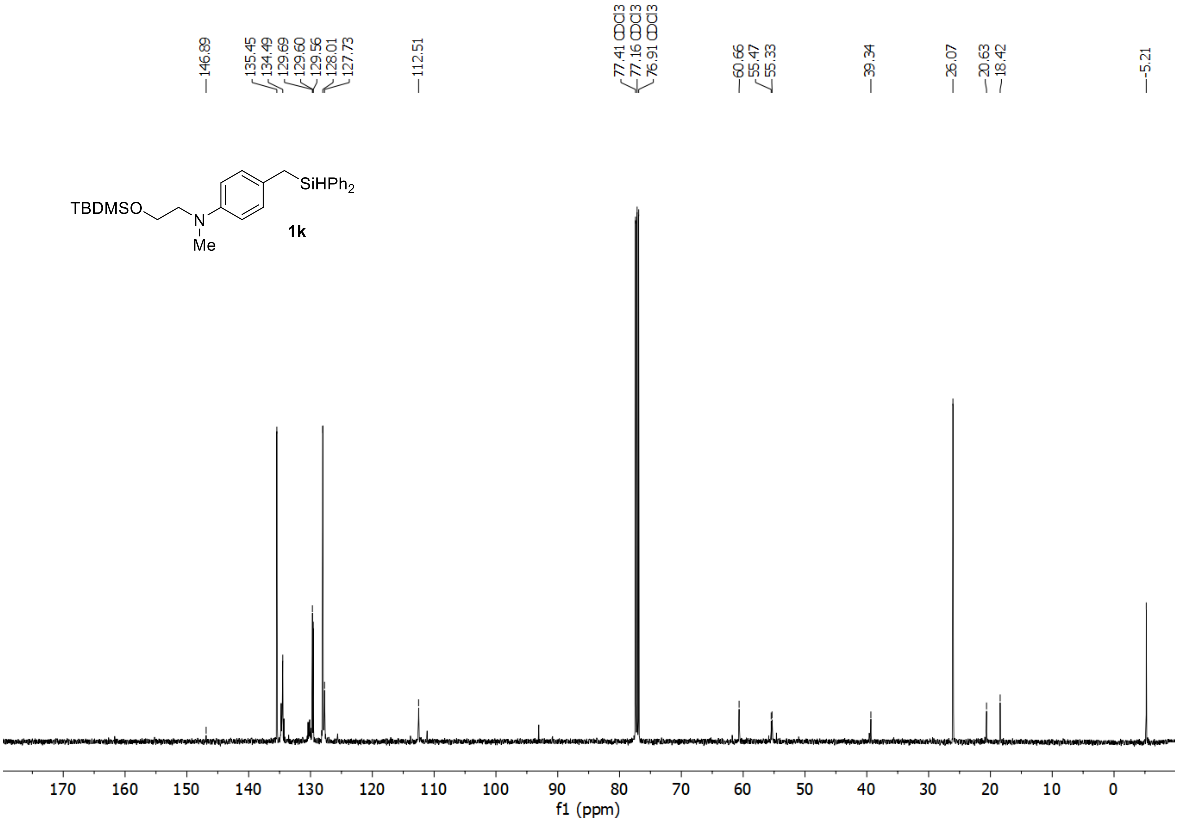

Figure S-72. ^{13}C-NMR (126 MHz, $CDCl_3$) of **2k**.

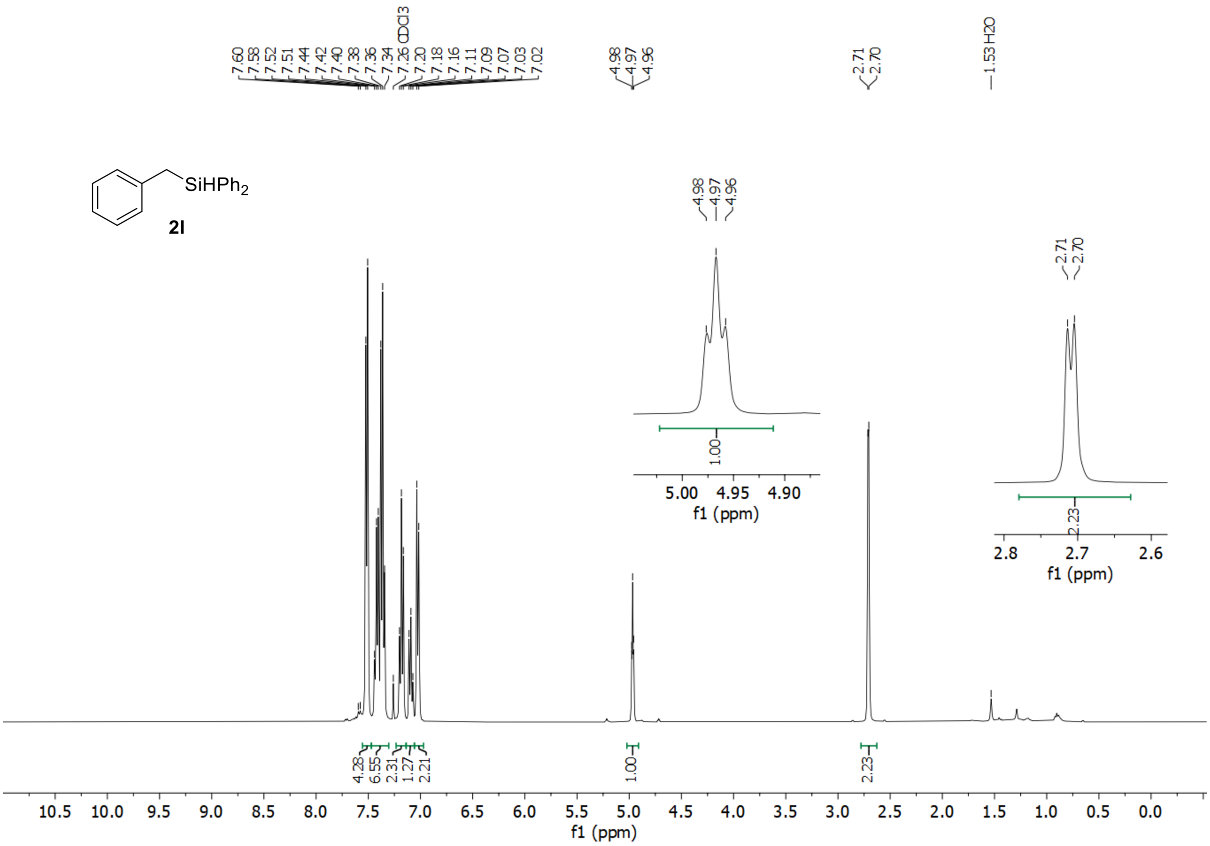

Figure S-73. ^{1}H-NMR (500 MHz, $CDCl_3$) of **2l**.

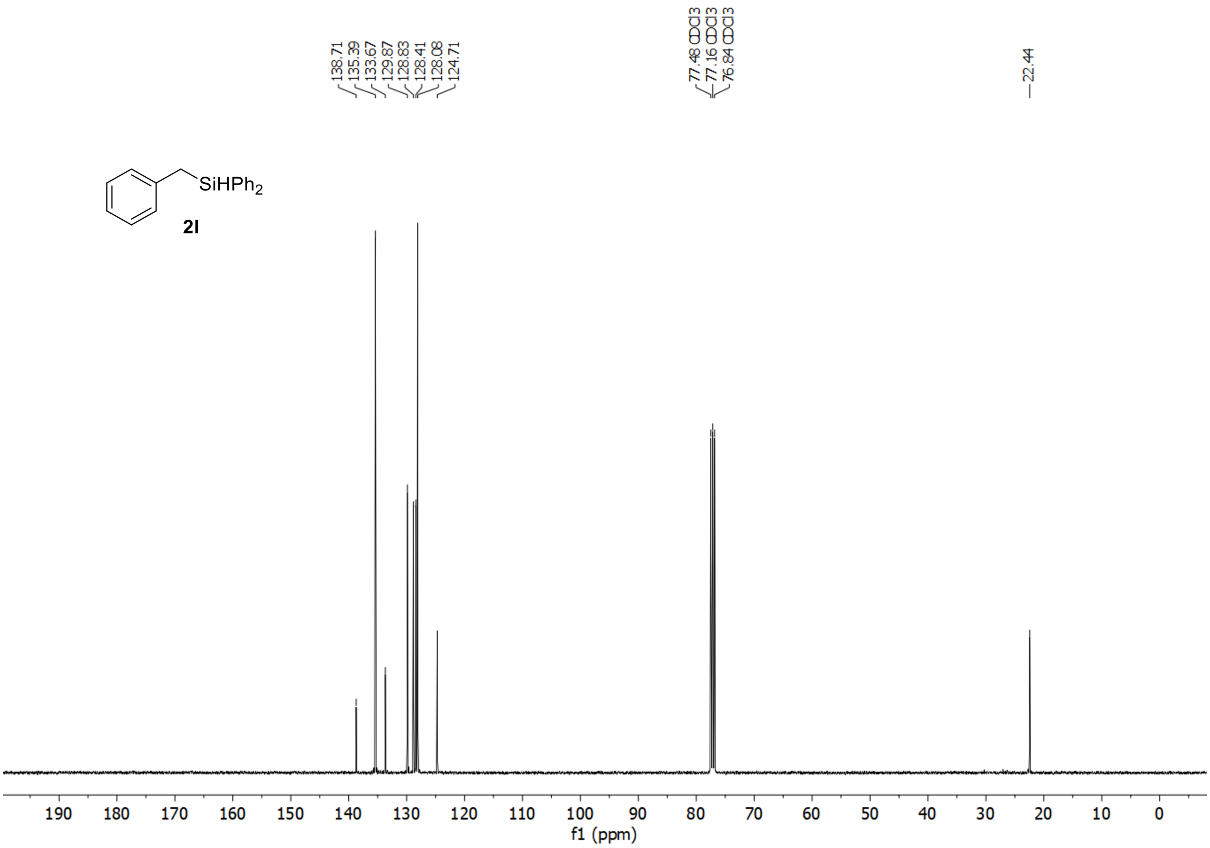

Figure S-74. ^{13}C-NMR (126 MHz, $CDCl_3$) of **2l**.

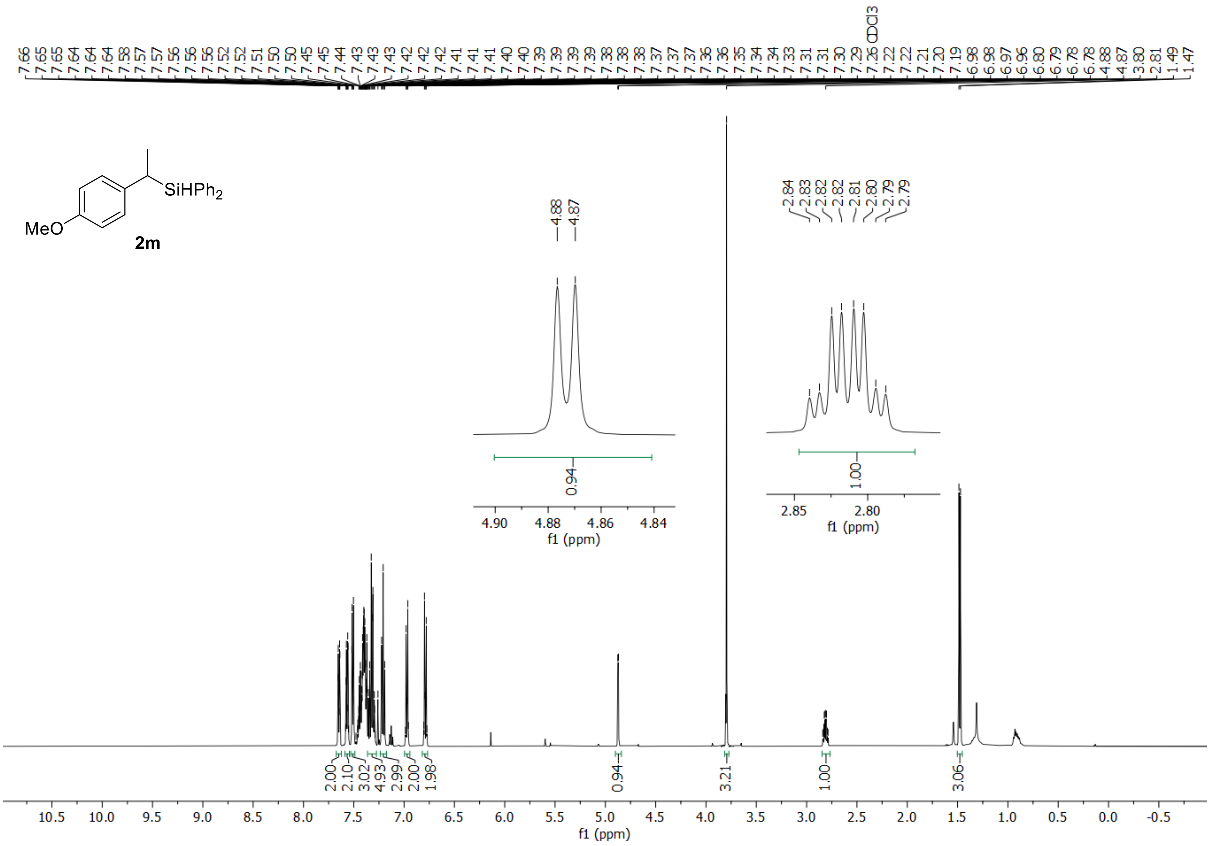

Figure S-75. ^{1}H-NMR (500 MHz, $CDCl_3$) of **2m**.

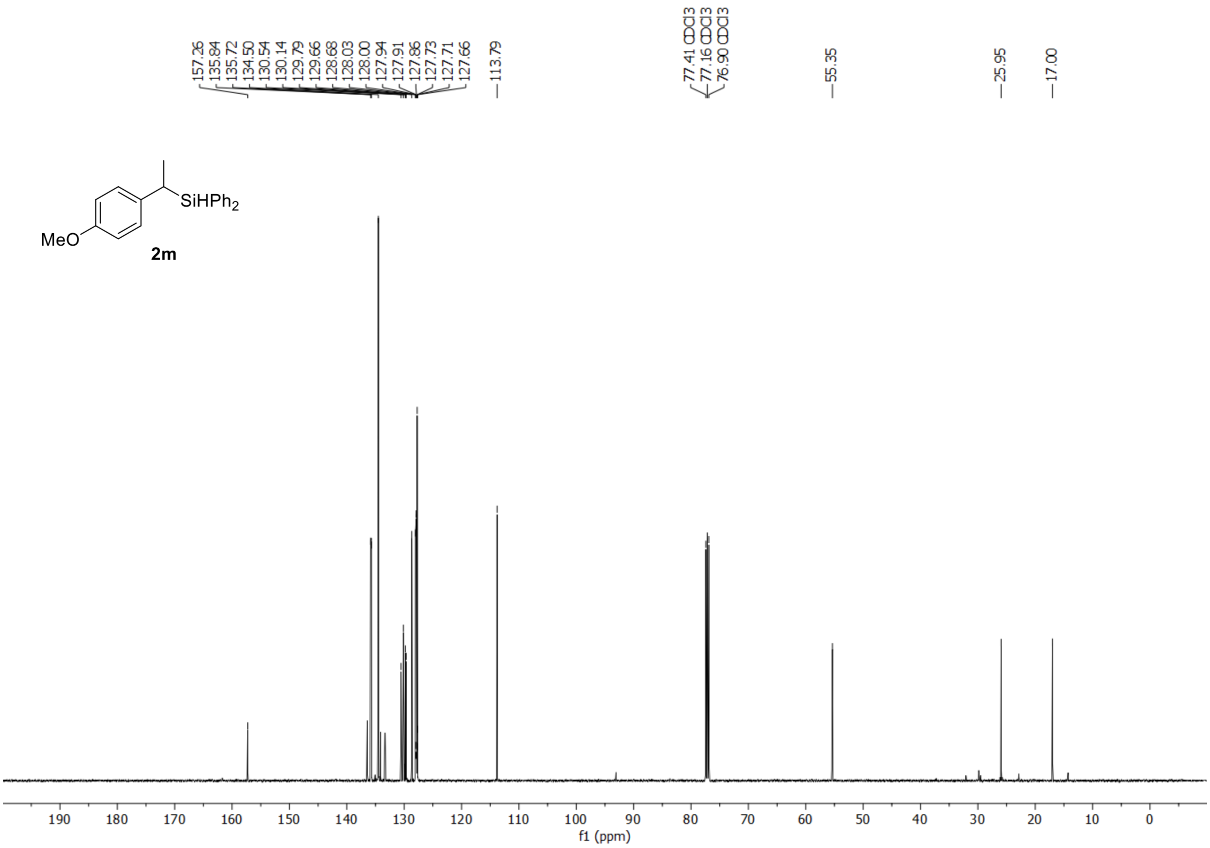

Figure S-76. ^{13}C-NMR (126 MHz, $CDCl_3$) of **2m.**

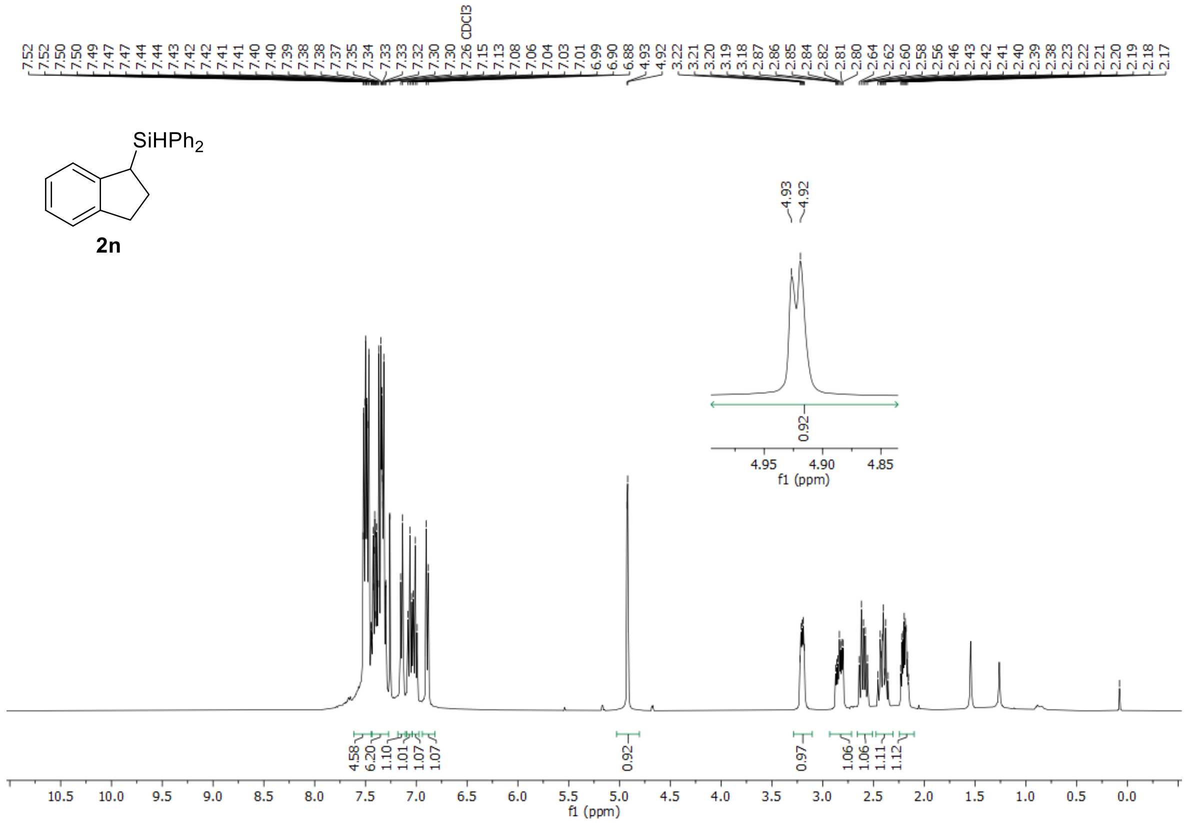

Figure S-77. ^{1}H-NMR (400 MHz, $CDCl_3$) of **2n**.

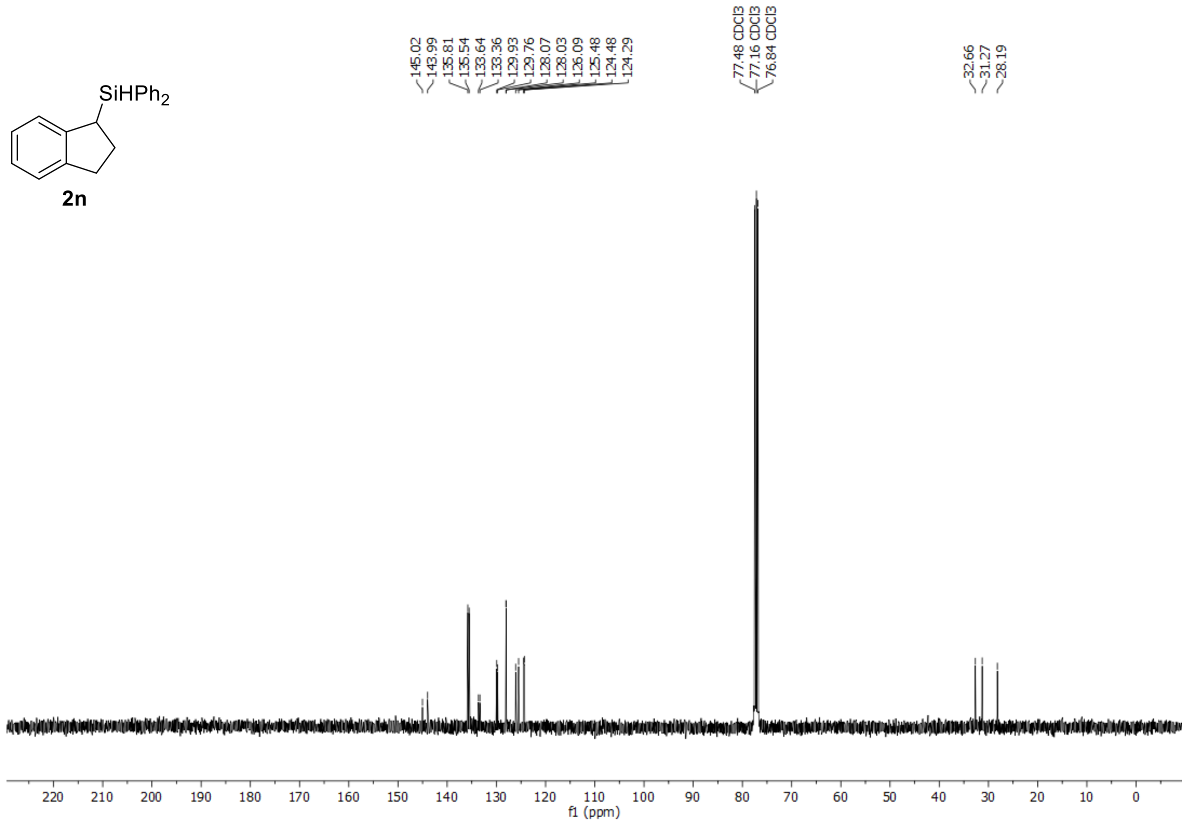

Figure S-78. ^{13}C-NMR (101 MHz, $CDCl_3$) of **2n**.

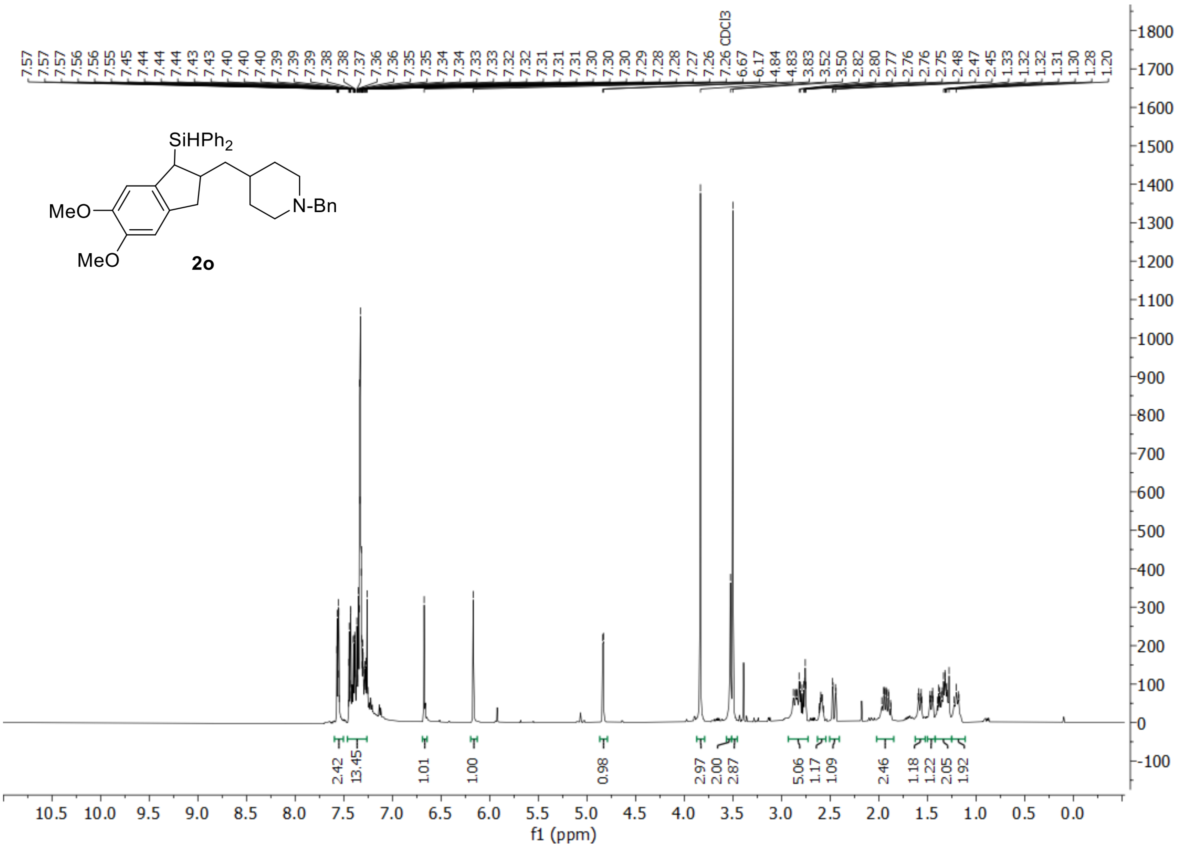

Figure S-79. ^{1}H-NMR (500 MHz, $CDCl_3$) of **2o**.

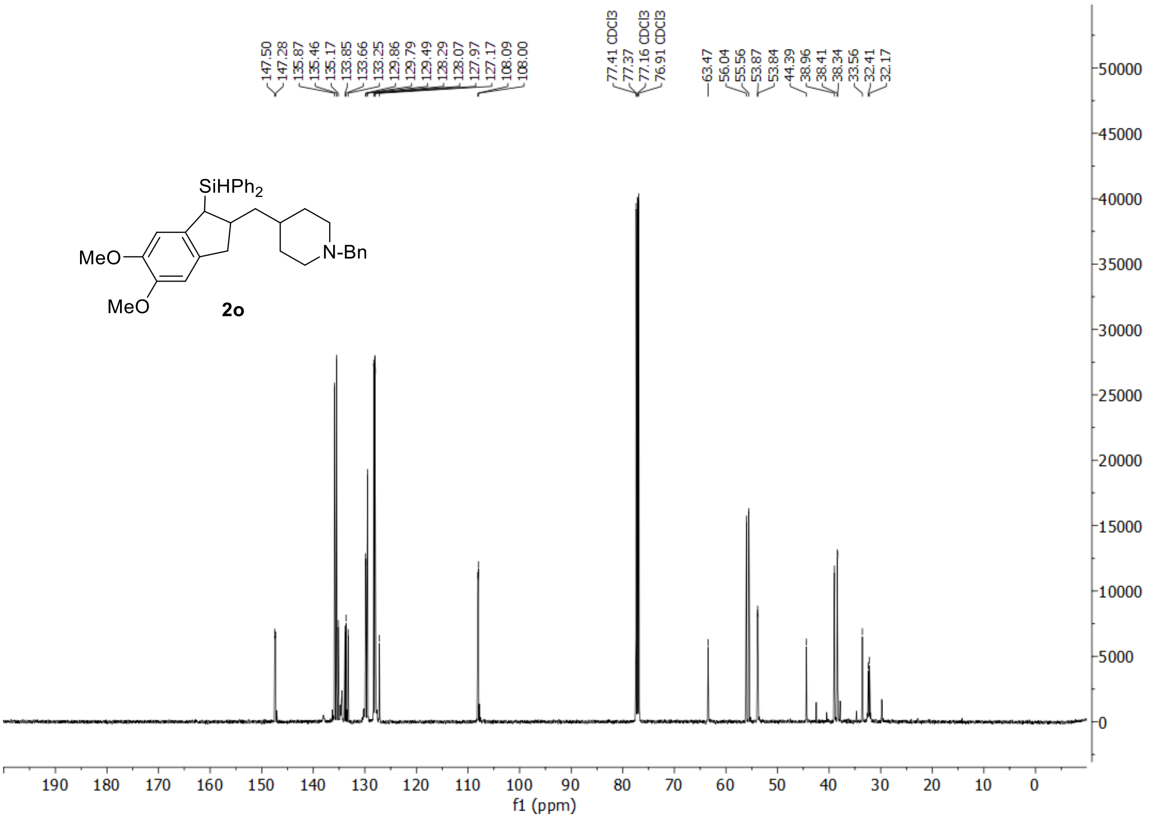

Figure S-80. ^{13}C-NMR (126 MHz, $CDCl_3$) of **2o**.

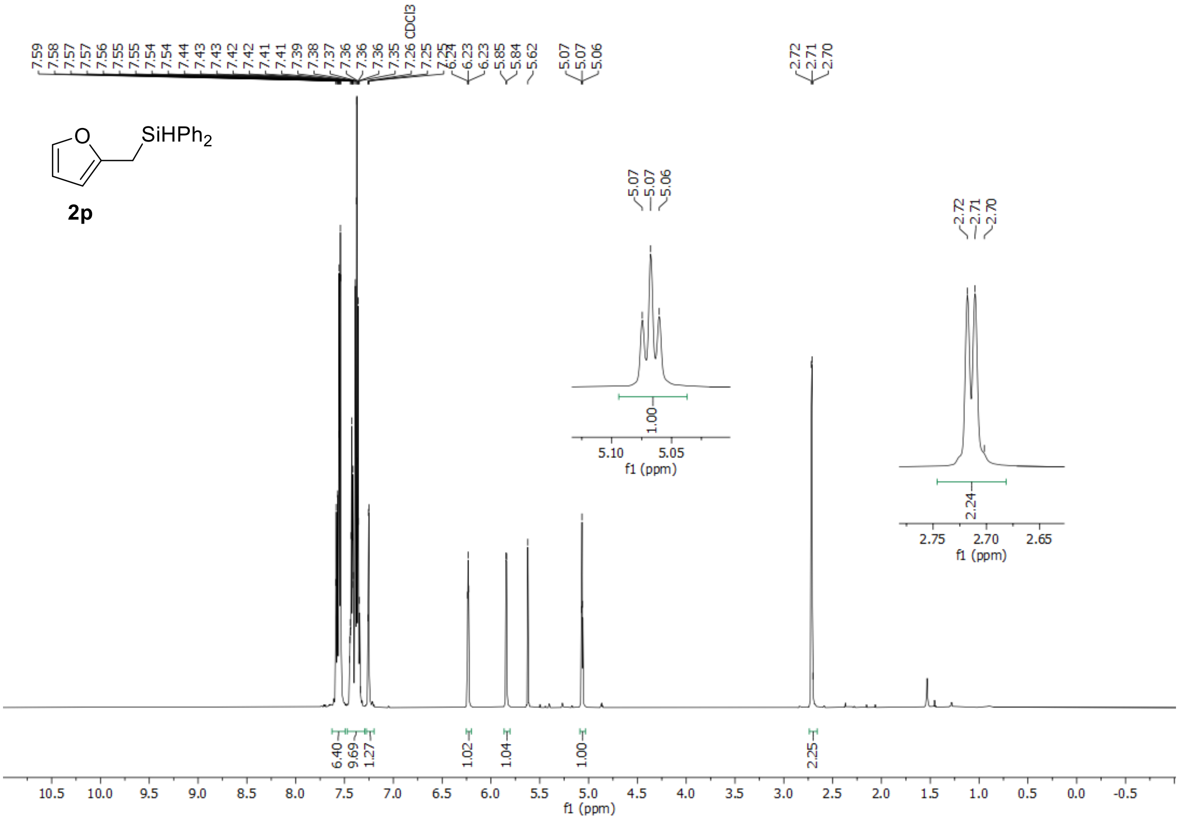

Figure S-81. ^{1}H-NMR (500 MHz, $CDCl_3$) of **2p**.

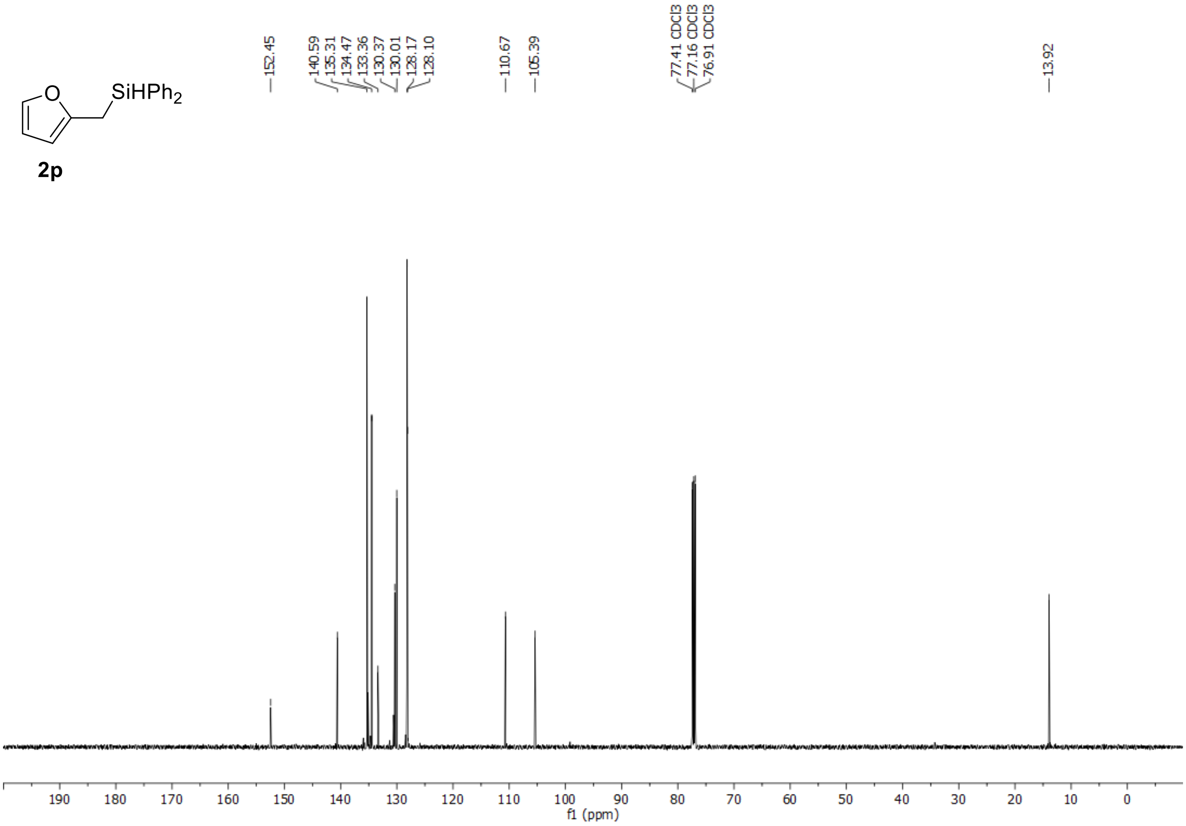

Figure S-82. ^{13}C-NMR (126 MHz, $CDCl_3$) of **2p**.

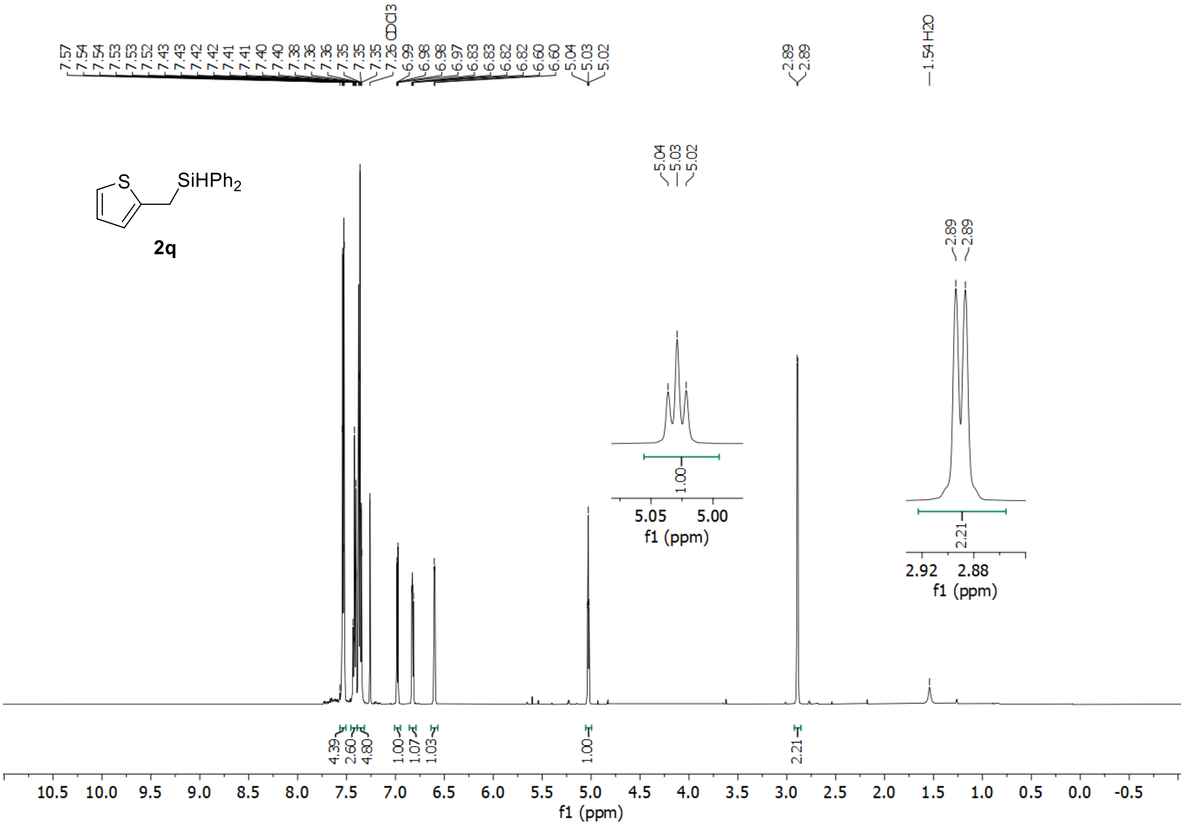

Figure S-83. ^{1}H-NMR (500 MHz, $CDCl_3$) of **2q**.

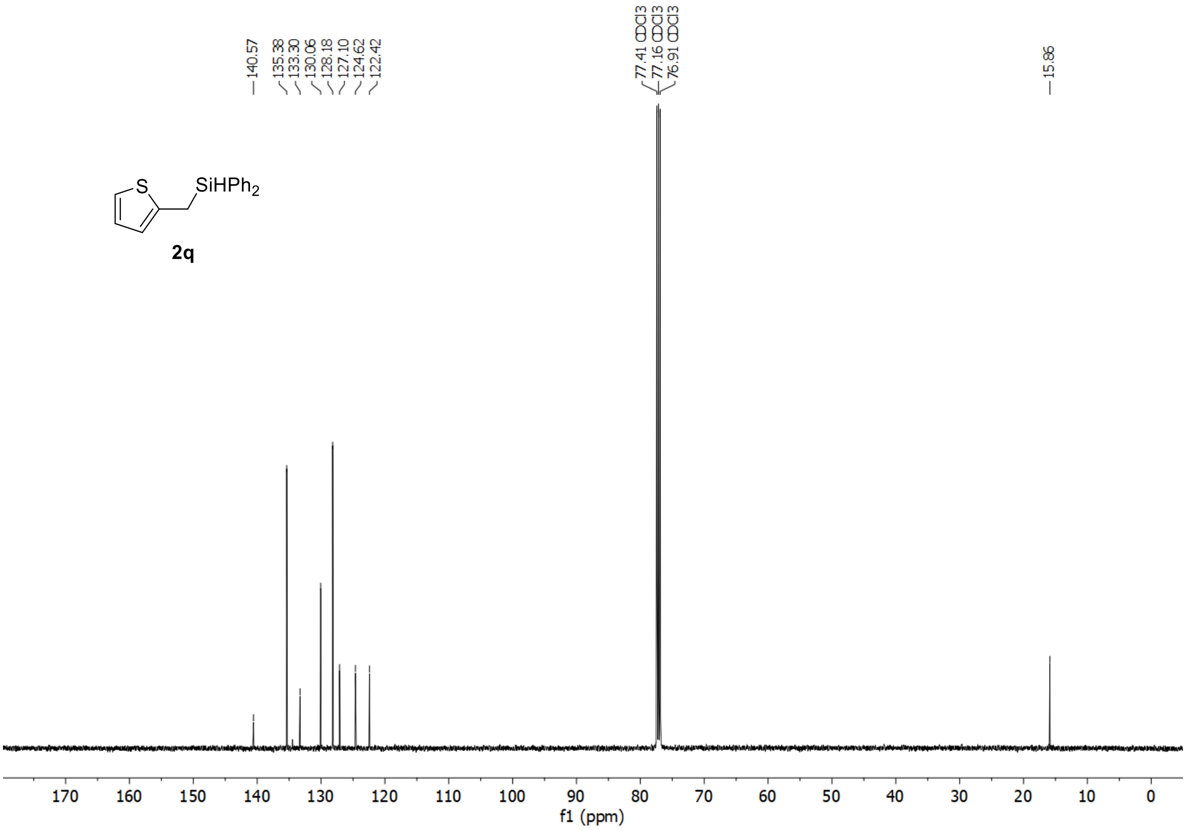

Figure S-84. ^{13}C-NMR (126 MHz, $CDCl_3$) of **2q**.

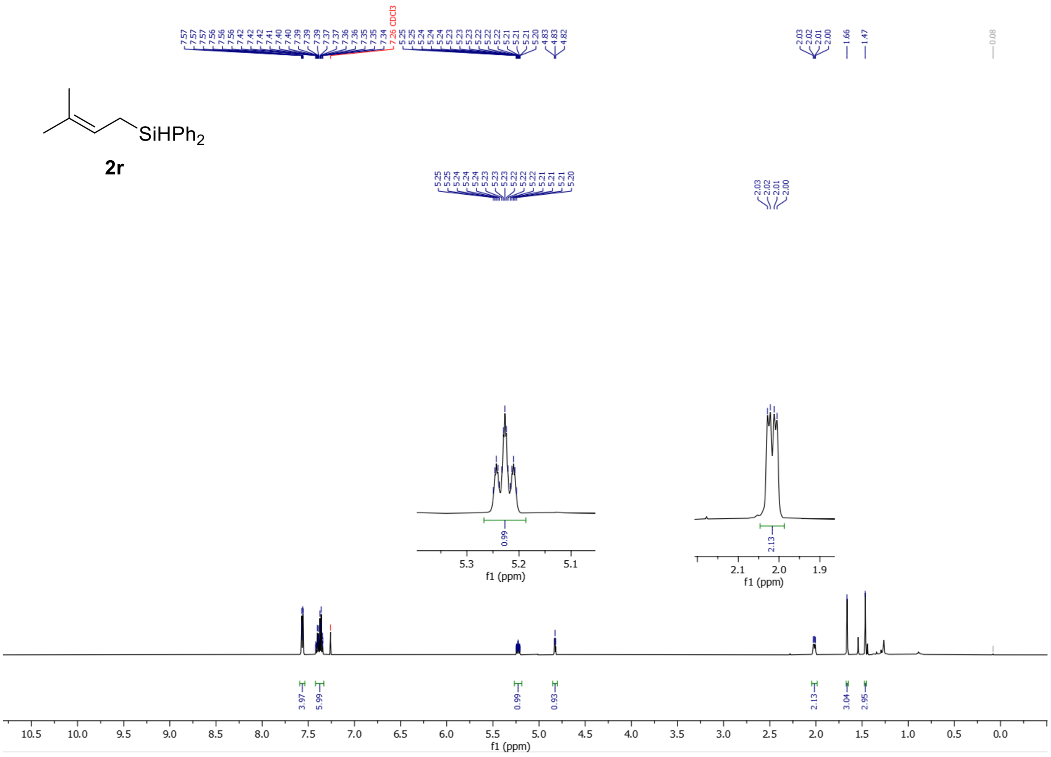

Figure S-85. ^{1}H-NMR (500 MHz, $CDCl_3$) of **2r**.

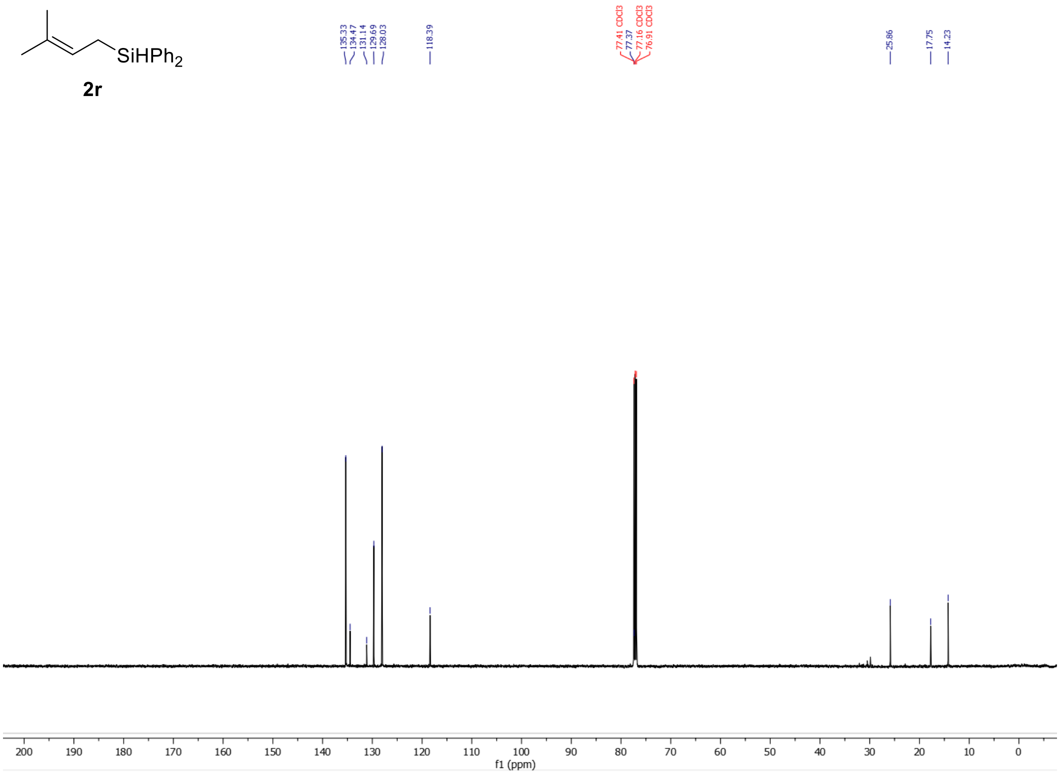

Figure S-86. ^{13}C-NMR (126 MHz, $CDCl_3$) of **2r**.

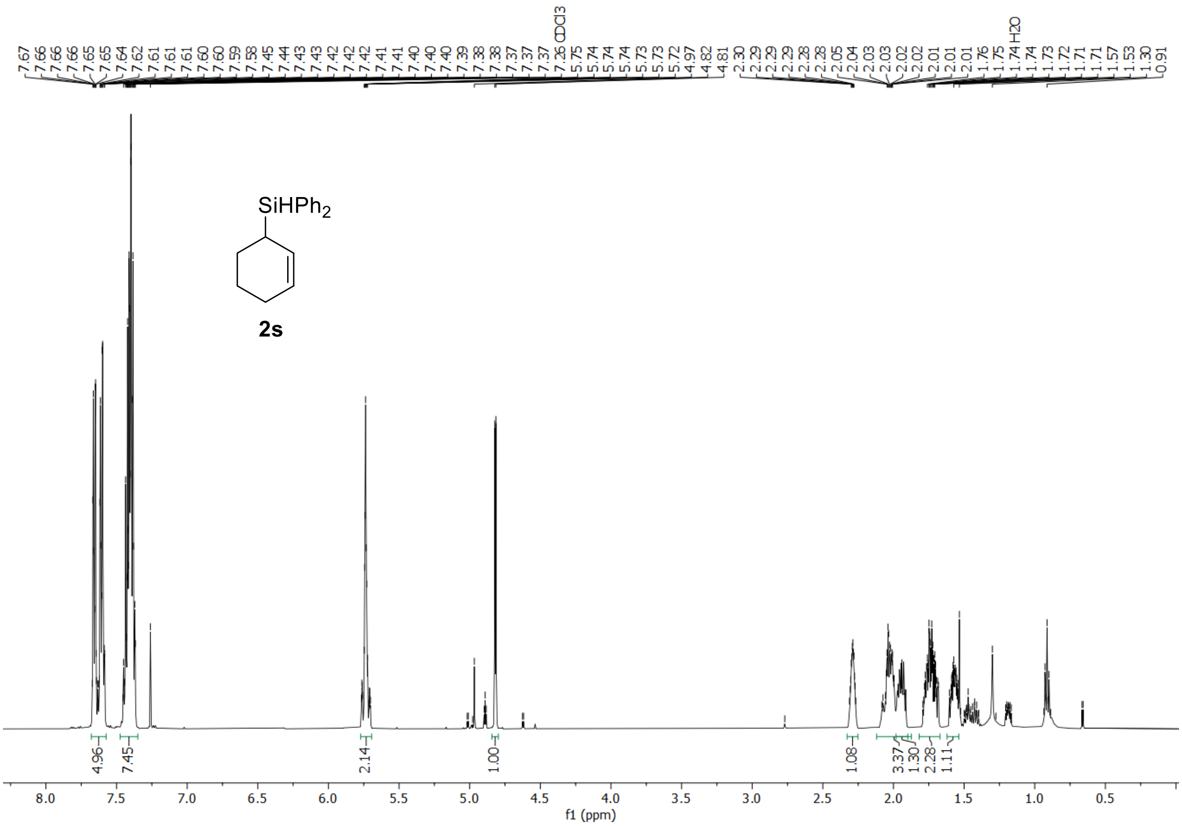

Figure S-87. ^{1}H-NMR (500 MHz, $CDCl_3$) of **2s**.

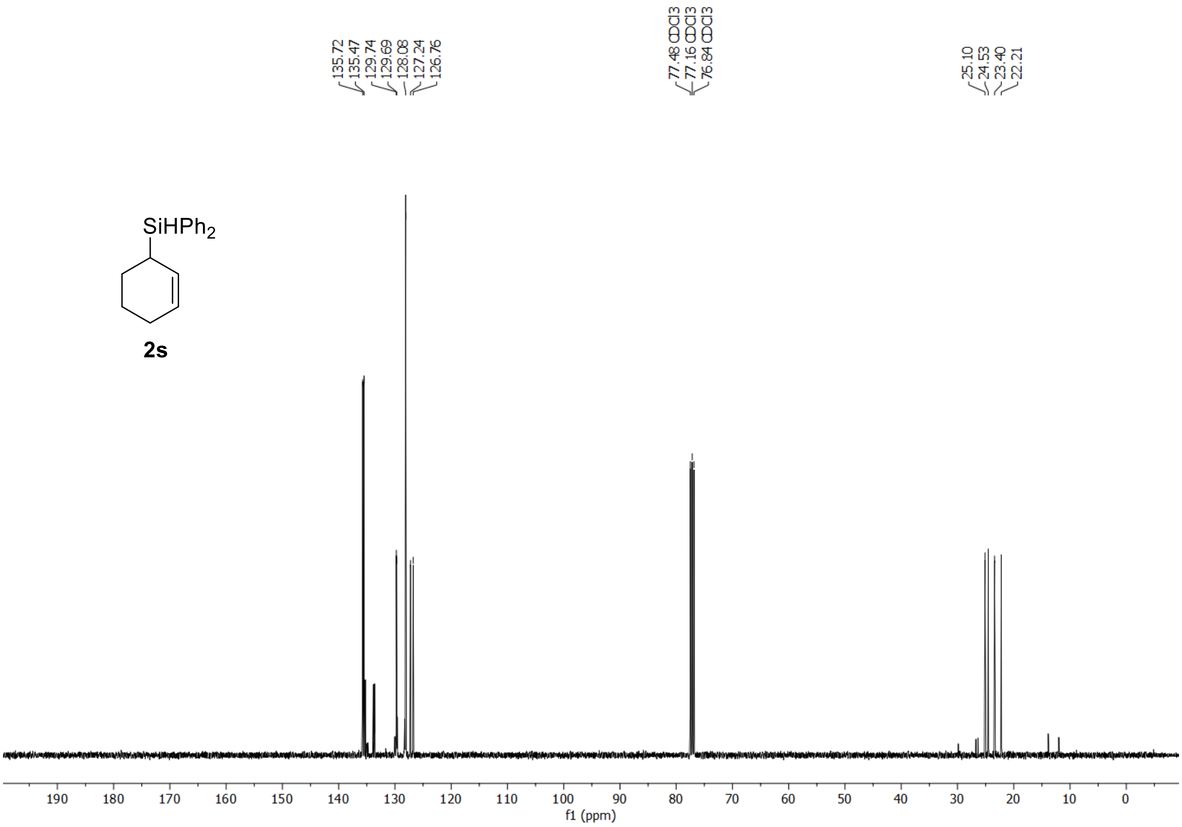

Figure S-88. ^{13}C-NMR (101 MHz, $CDCl_3$) of **2s**.

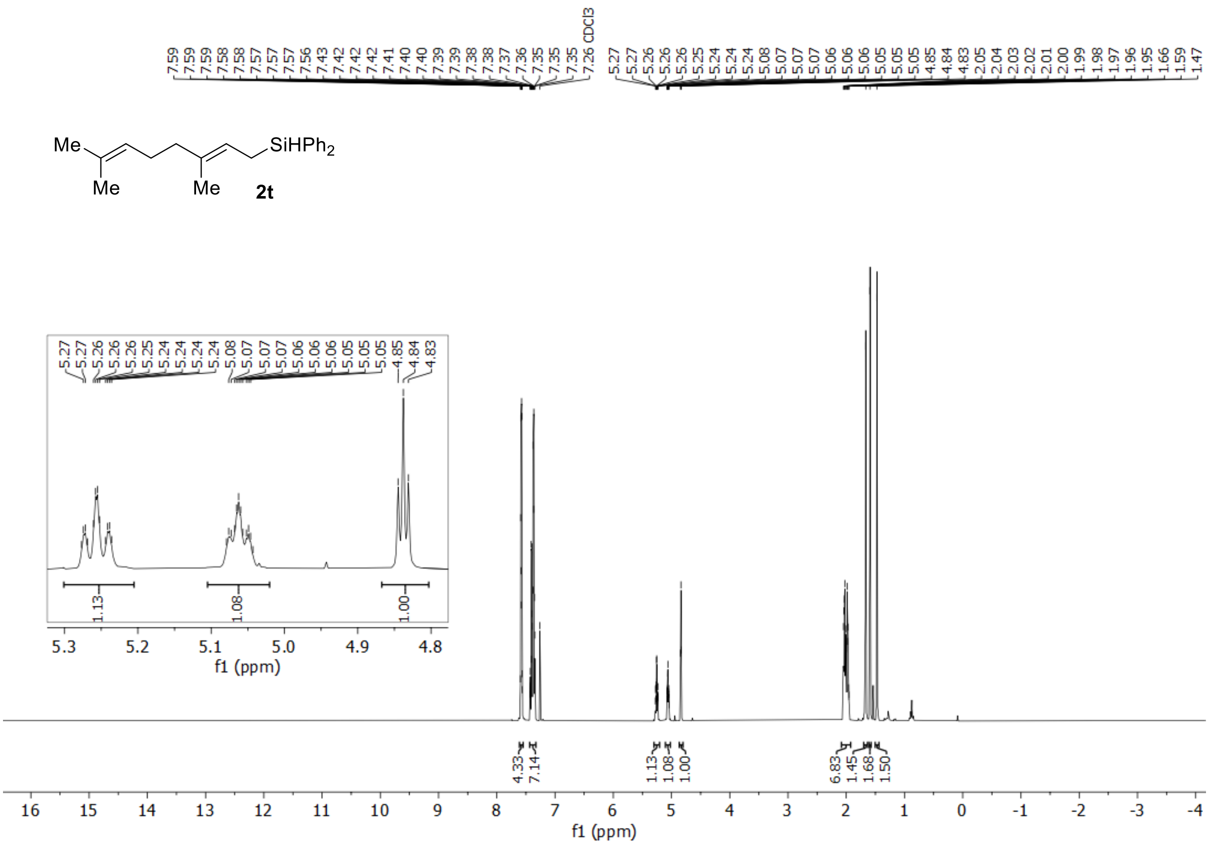

Figure S-89. ^{1}H-NMR (500 MHz, $CDCl_3$) of **2t**.

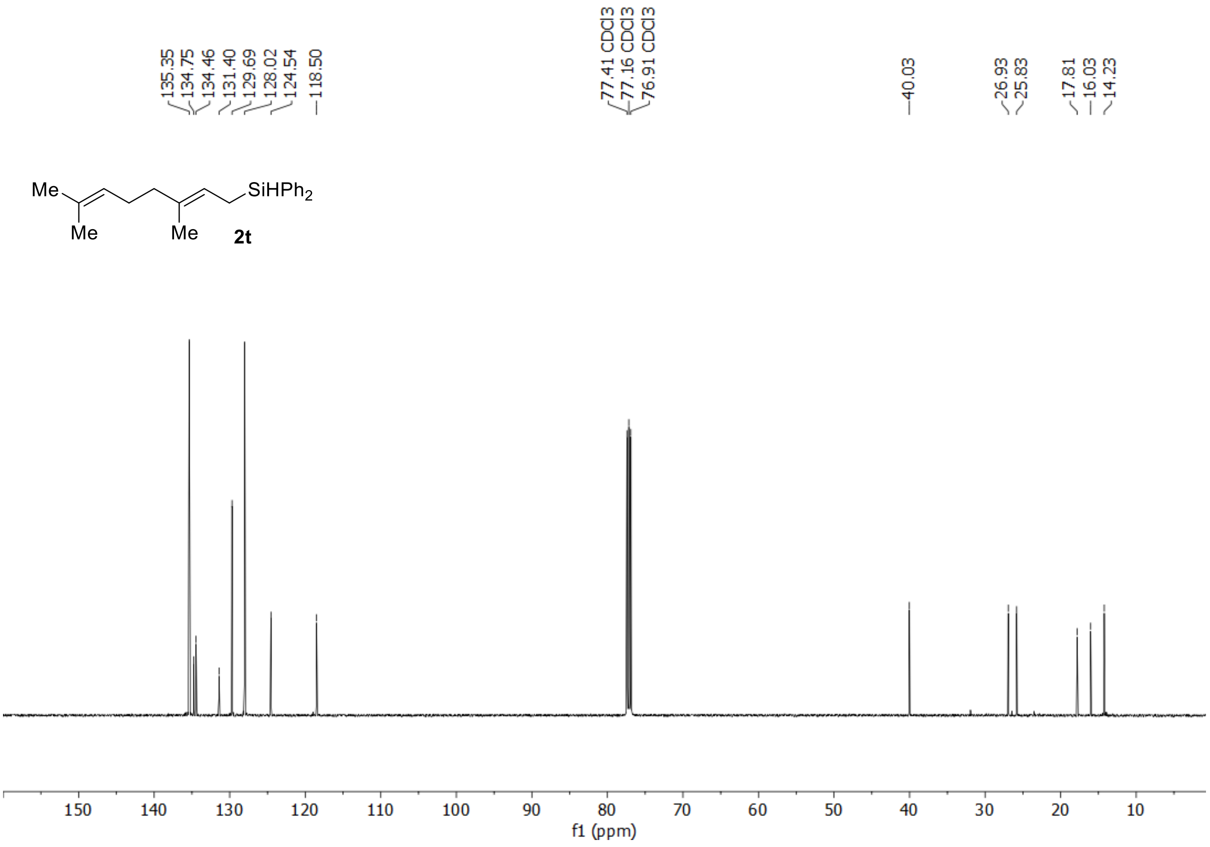

Figure S-90. ^{13}C-NMR (101 MHz, $CDCl_3$) of **2t**.

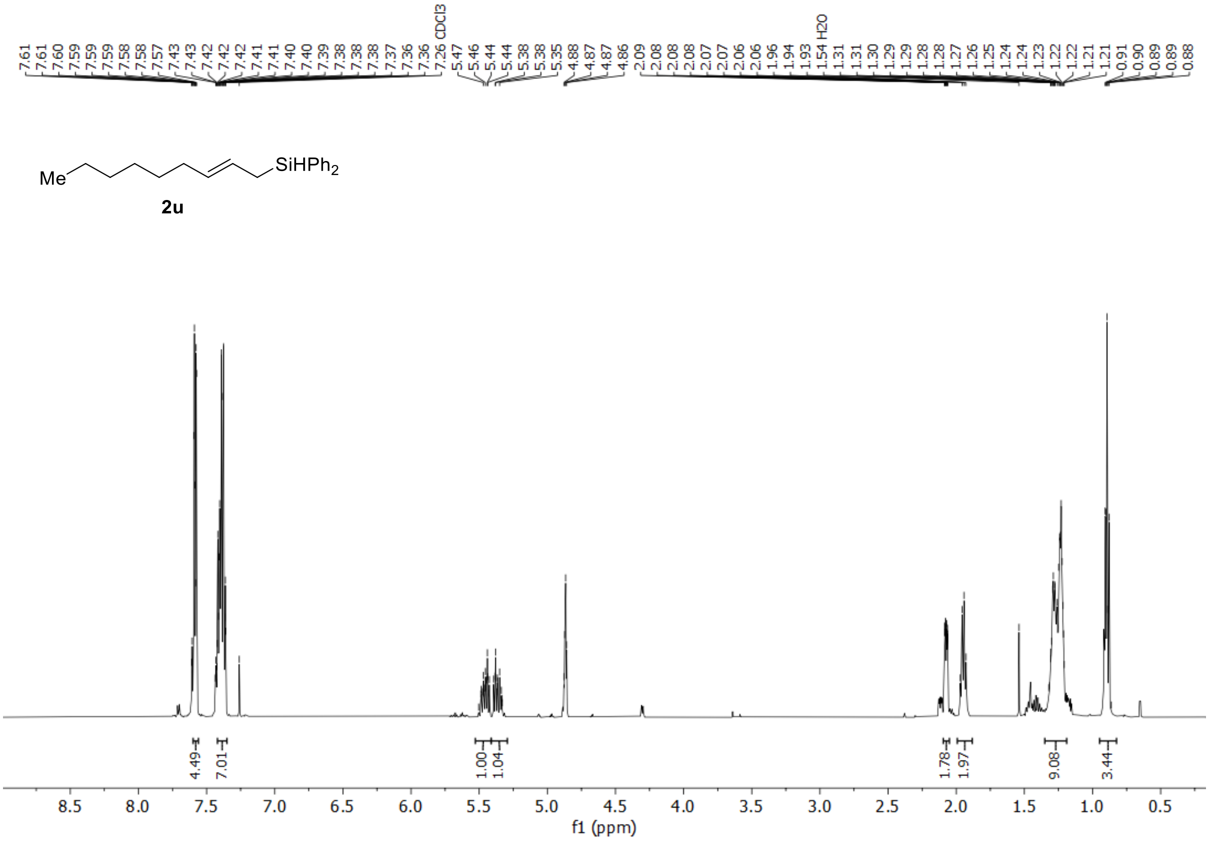

Figure S-91. ^{1}H-NMR (500 MHz, $CDCl_3$) of **2u**.

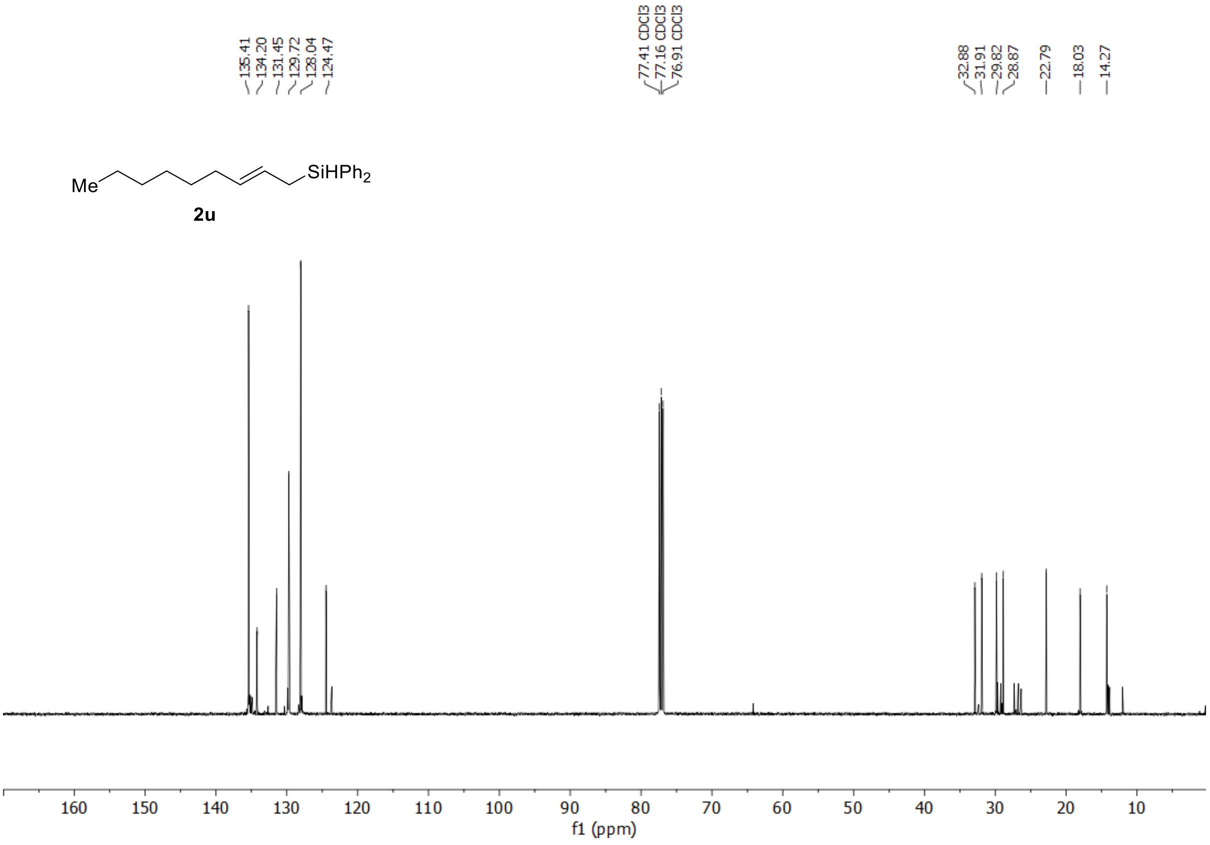

Figure S-92. ^{13}C-NMR (101 MHz, $CDCl_3$) of **2u**.

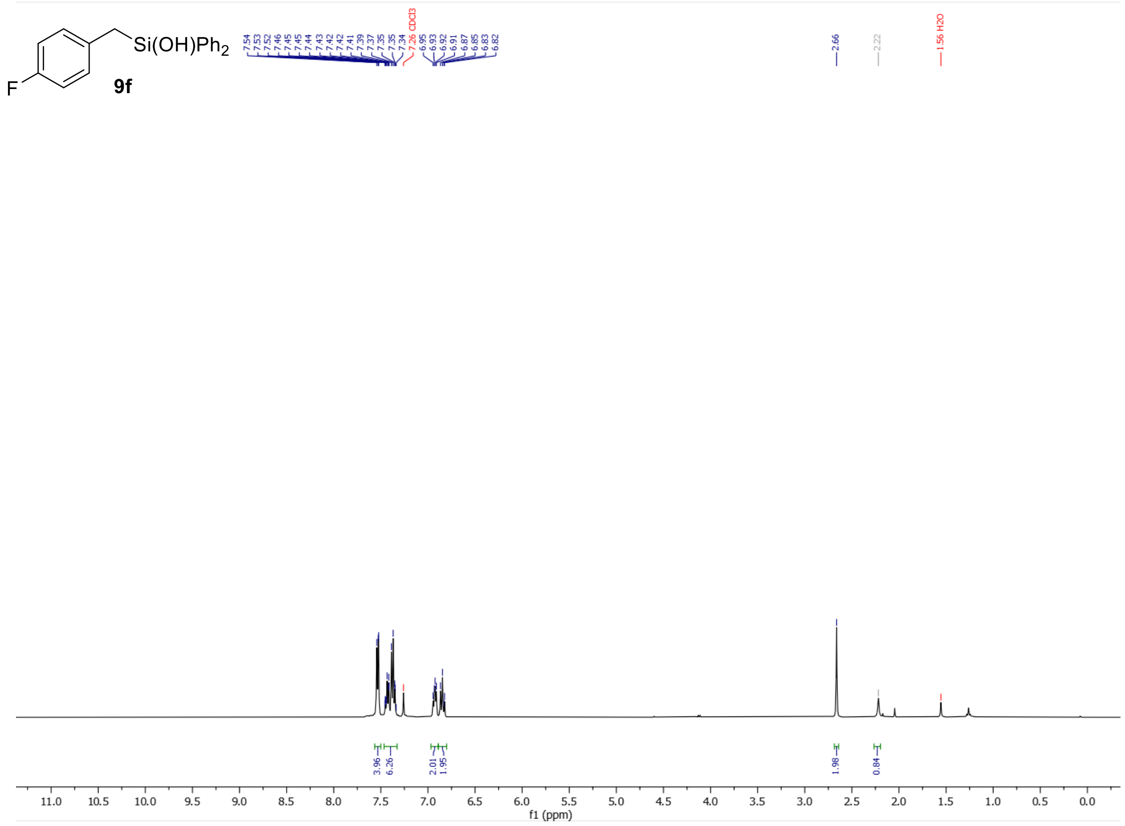

Figure S-93. ^{1}H-NMR (400 MHz, $CDCl_3$) of **9f**.

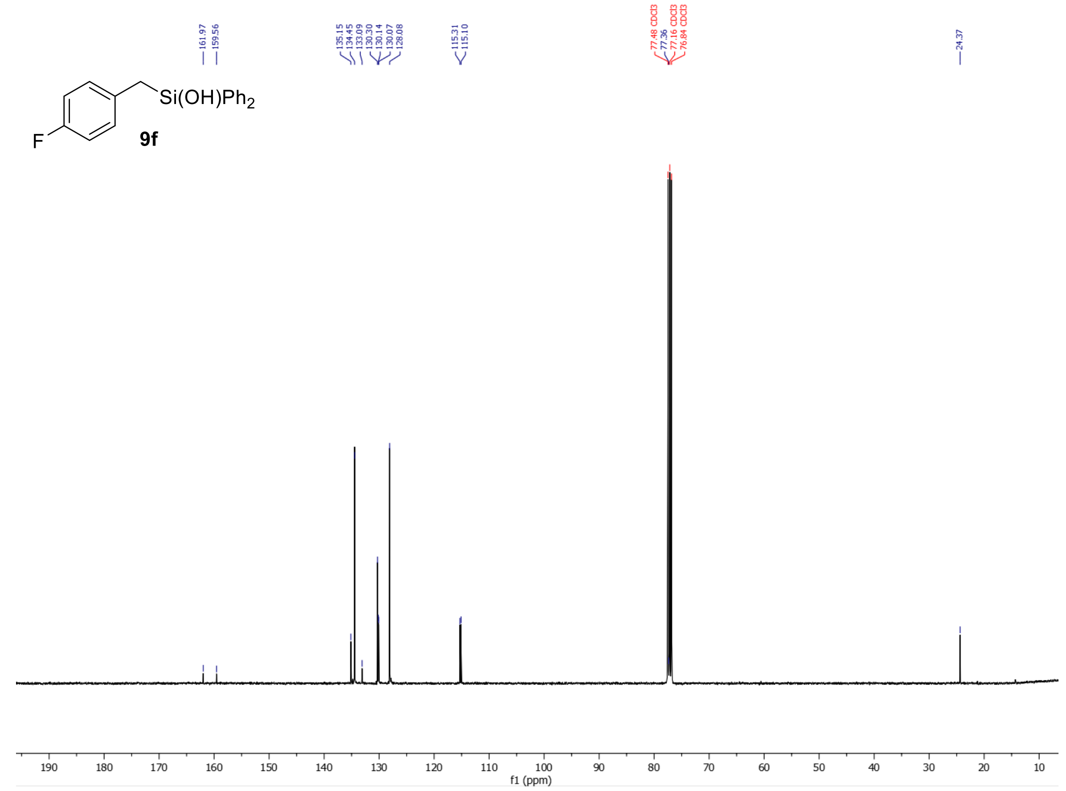

Figure S-94. ^{13}C-NMR (126 MHz, $CDCl_3$) of **9f**.

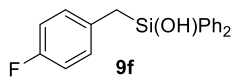

9f

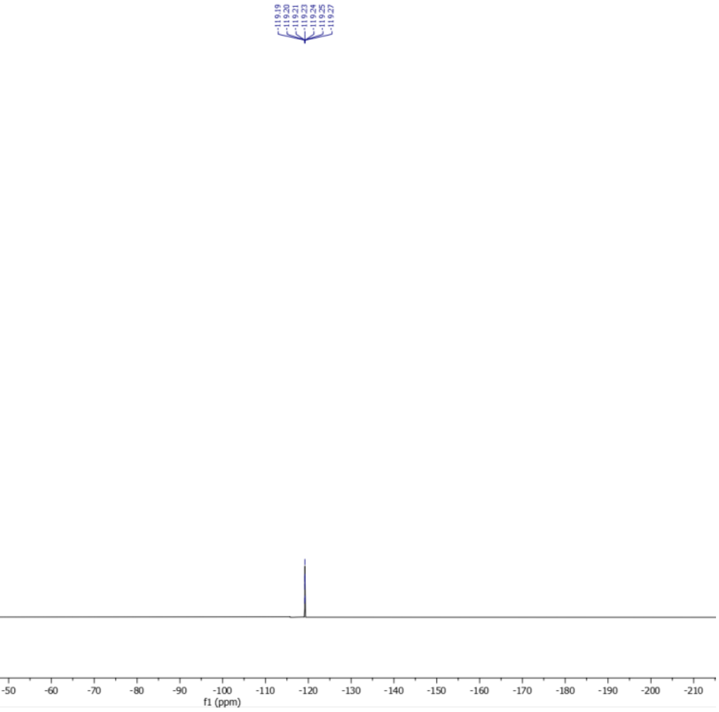

Figure S-95. ^{19}F-NMR (377 MHz, $CDCl_3$) of **9f**.

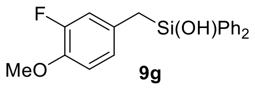

9g

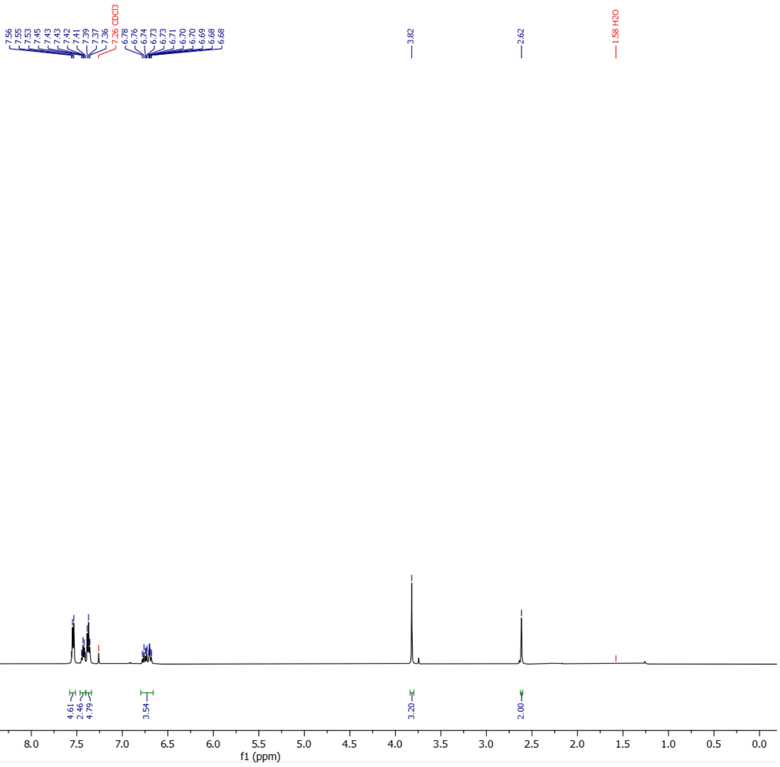

Figure S-96. ^{1}H-NMR (400 MHz, $CDCl_3$) of **9g**.

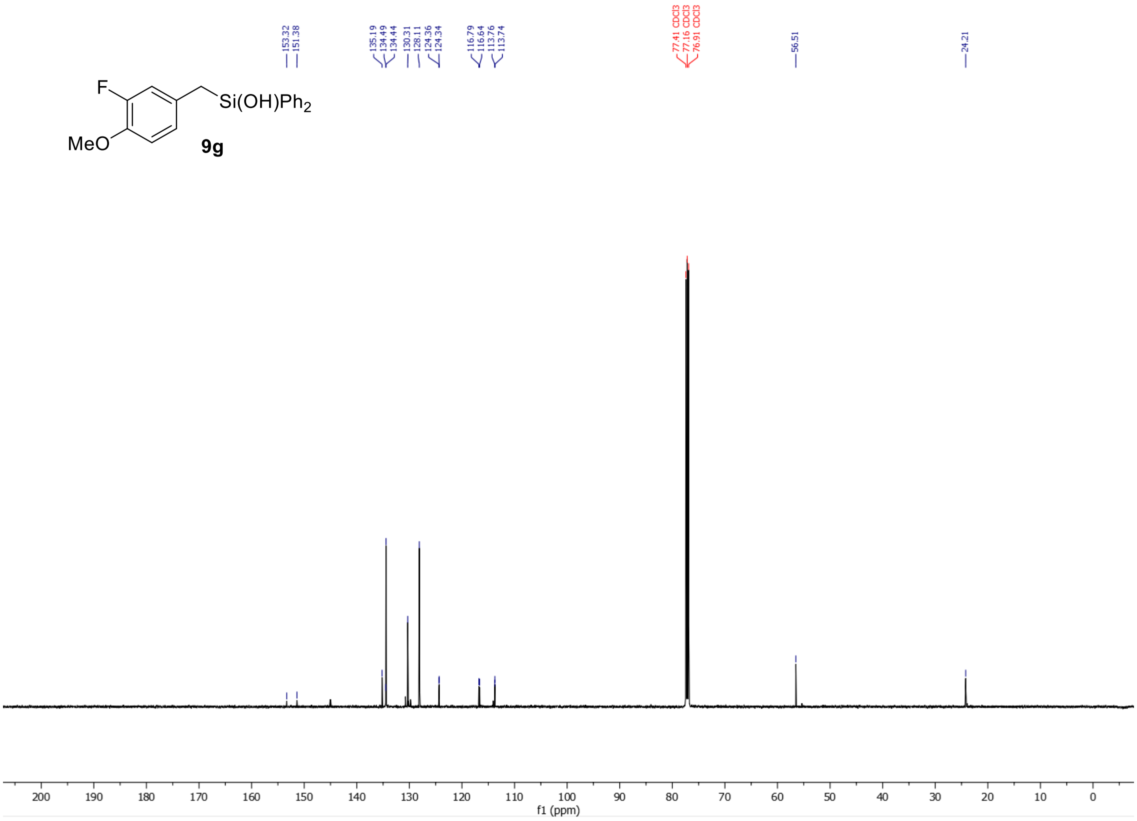

Figure S-97. ^{13}C-NMR (126 MHz, $CDCl_3$) of **9g**.

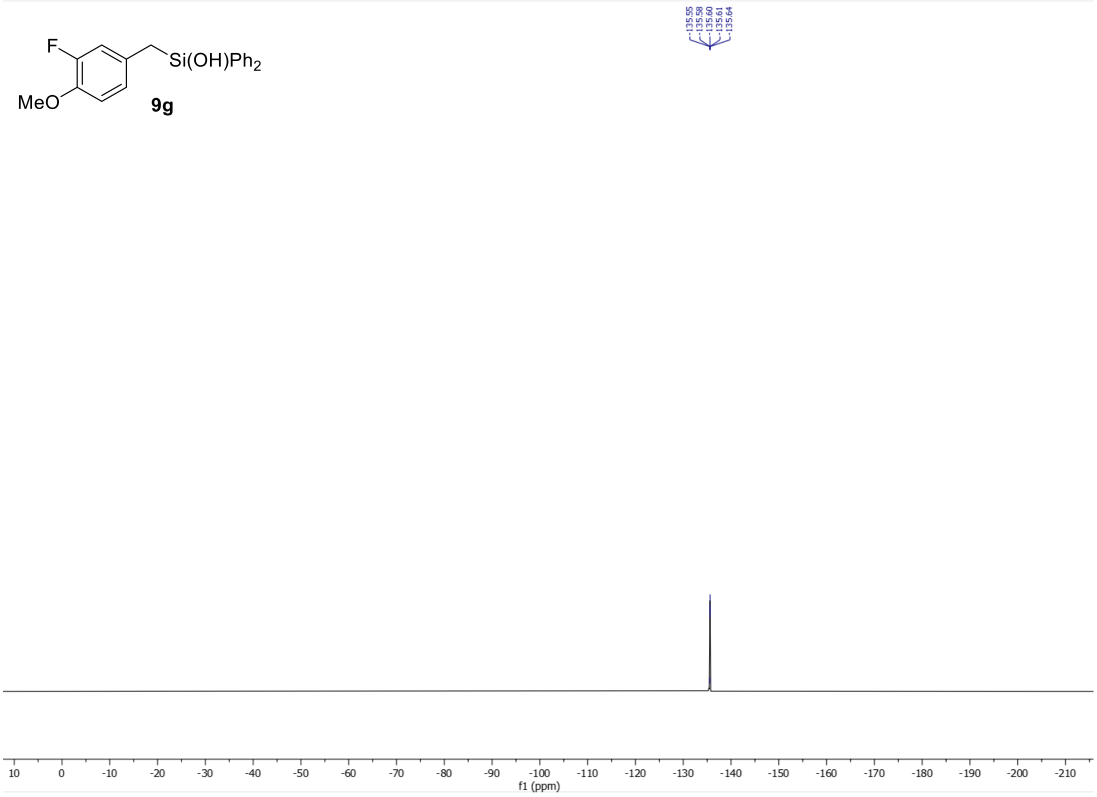

Figure S-98. ^{19}F-NMR (377 MHz, $CDCl_3$) of **9g**.

16. HPLC chromatograms

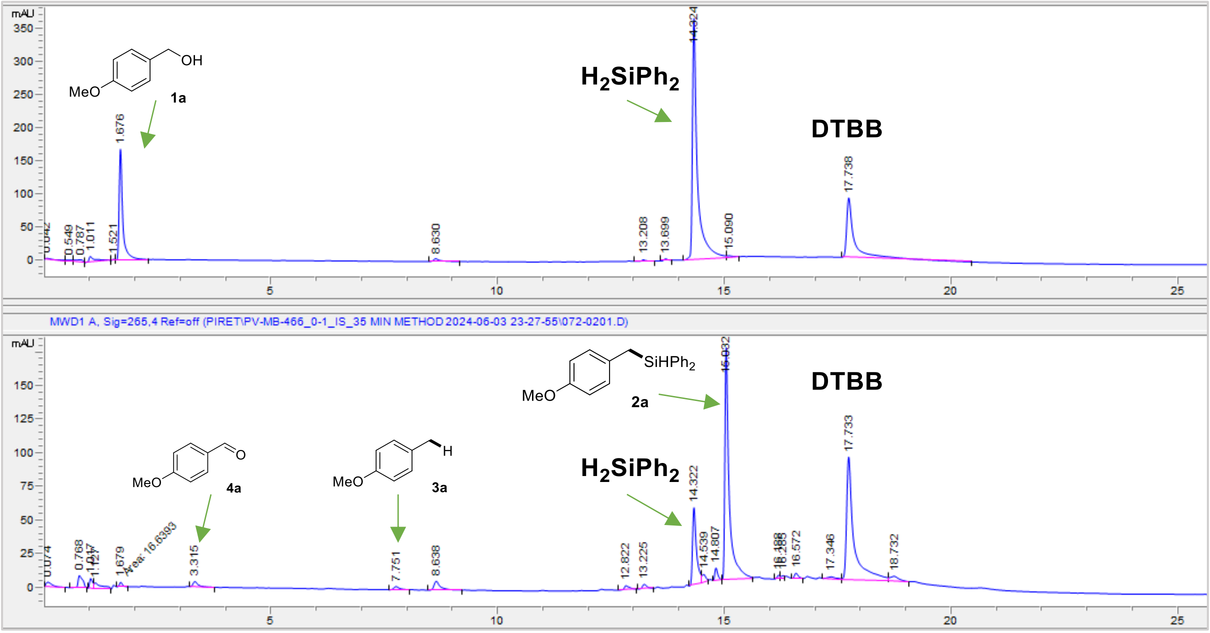

Figure S-99. HPLC analysis of electroreductive silylation of **1a** (0.1 M) with 5 equiv. diphenylsilane (10 mA) at 0-time (top) and after 3 h (bottom) with 4,4'-di-*tert*-butylbiphenyl (DTBB) as HPLC standard. Retention times for **1a** (R_t = 1.6 min), **4a** (R_t = 3.3 min), **3a** (R_t = 7.7 min), H_2SiPh_2 (R_t = 14.3 min), **2a** (R_t = 15.0 min), DTBB (R_t = 17.7 min). For reaction details see Figure S-1.

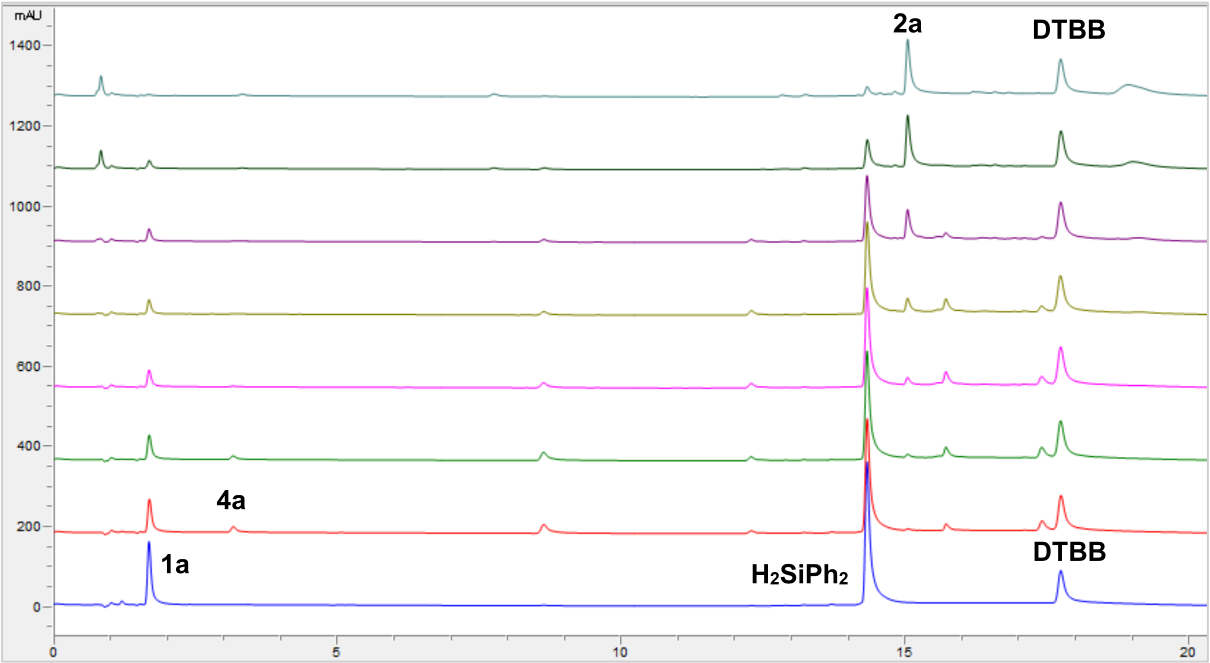

Figure S-100. HPLC analysis over time of electroreductive silylation of **1a** (0.1 M) with 5 equiv. diphenylsilane at 10 mA from 0-time (bottom) to 3 h (top) with 4,4'-di-*tert*-butylbiphenyl (DTBB) as HPLC standard. Retention times for **1a** (R_t = 1.6 min), **4a** (R_t = 3.3 min), **3a** (R_t = 7.7 min), H_2SiPh_2 (R_t = 14.3 min), **2a** (R_t = 15.0 min), DTBB (R_t = 17.7 min). For reaction details see Figure S-1.

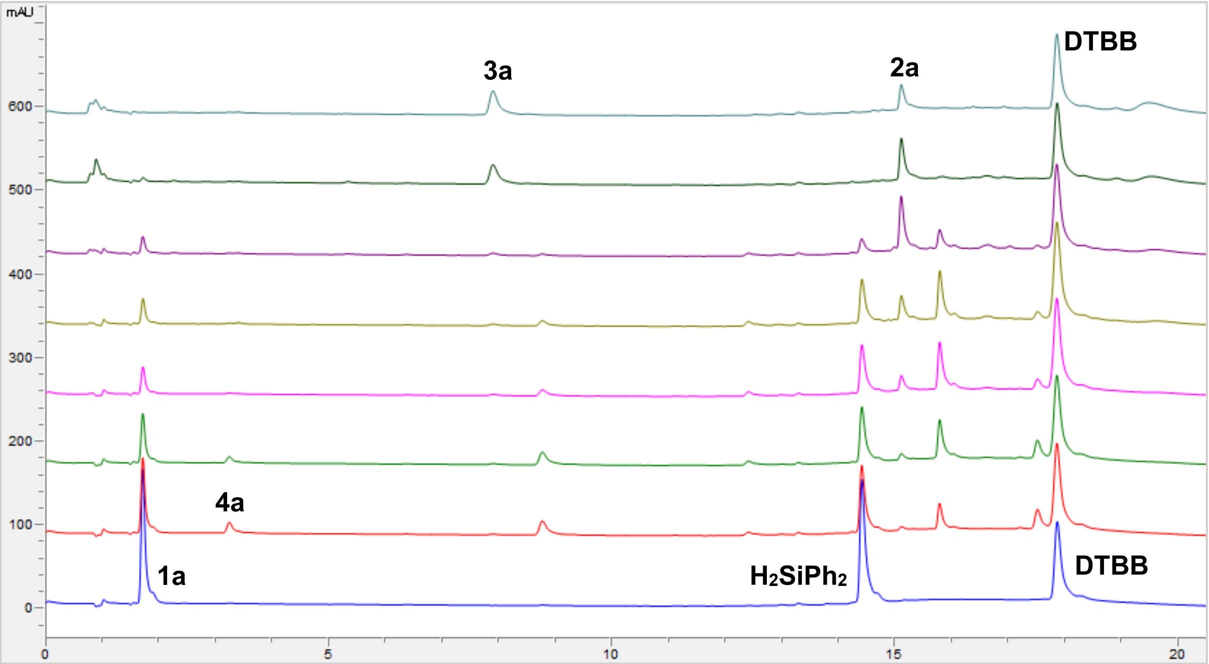

Figure S-101. HPLC analysis over time of electroreductive silylation of **1a** (0.1 M) 2 equiv. diphenylsilane at 10 mA from 0-time (bottom) to 3 h (top) with 4,4'-di-*tert*-butylbiphenyl (DTBB) as HPLC standard. Retention times for **1a** (R_t = 1.6 min), **4a** (R_t = 3.3 min), **3a** (R_t = 7.7 min), H_2SiPh_2 (R_t = 14.3 min), **2a** (R_t = 15.0 min), DTBB (R_t = 17.7 min). For reaction details see Figure S-2.

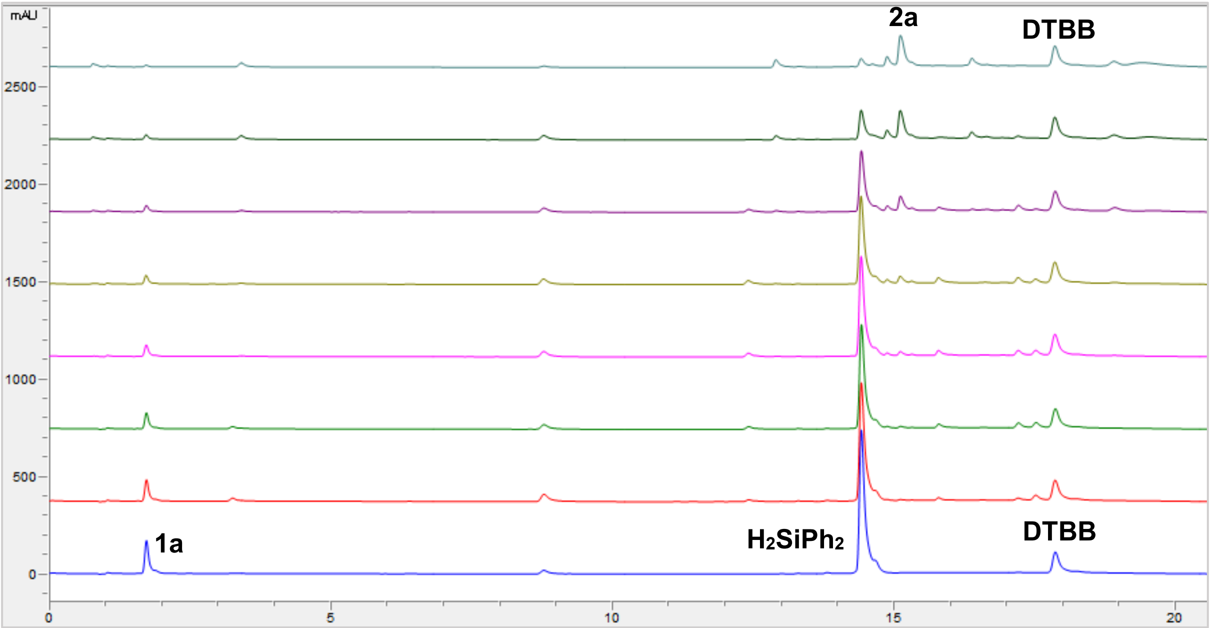

Figure S-102. HPLC analysis over time of electroreductive silylation of **1a** (0.1 M) 10 equiv. diphenylsilane at 10 mA from 0-time (bottom) to 3 h (top) with 4,4'-di-*tert*-butylbiphenyl (DTBB) as HPLC standard. Retentions times for **1a** (R_t = 1.6 min), **4a** (R_t = 3.3 min), **3a** (R_t = 7.7 min), H_2SiPh_2 (R_t = 14.3 min), **2a** (R_t = 15.0 min), DTBB (R_t = 17.7 min). For reaction details see Figure S-2.

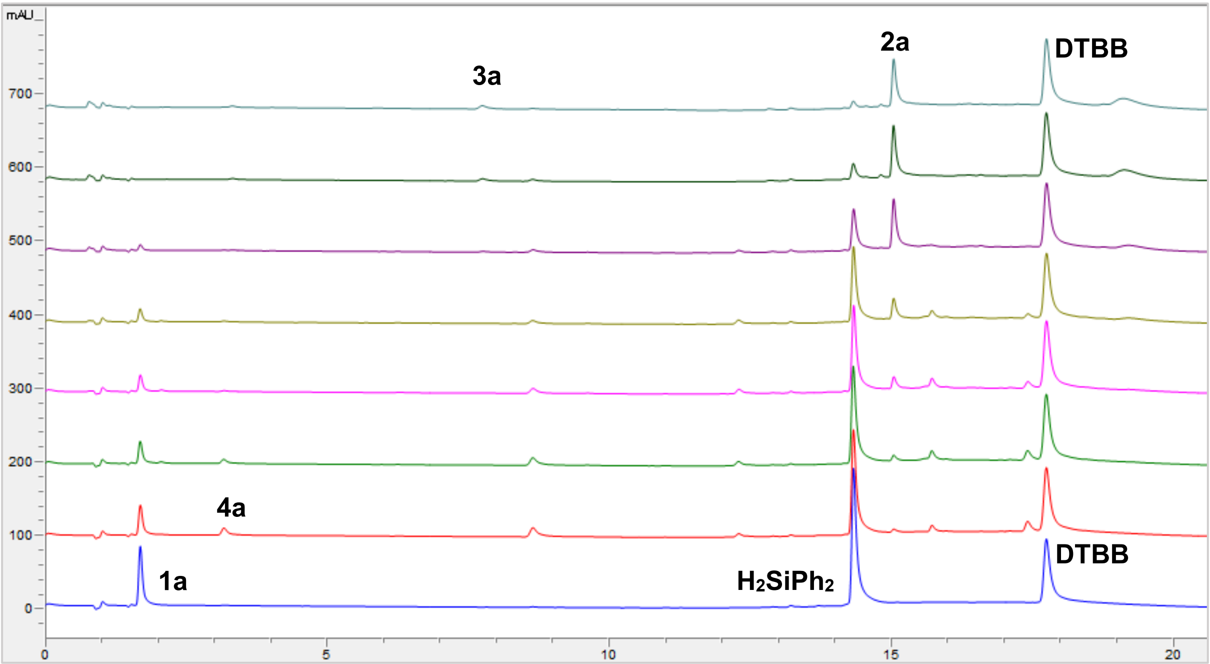

Figure S-103. HPLC analysis over time of electroreductive silylation of **1a** (0.05 M) 5 equiv. diphenylsilane at 10 mA from 0-time (bottom) to 3 h (top) with 4,4'-di-*tert*-butylbiphenyl (DTBB) as HPLC standard. Retention times for **1a** (R_t = 1.6 min), **4a** (R_t = 3.3 min), **3a** (R_t = 7.7 min), H_2SiPh_2 (R_t = 14.3 min), **2a** (R_t = 15.0 min), DTBB (R_t = 17.7 min). For reaction details see Figure S-3.

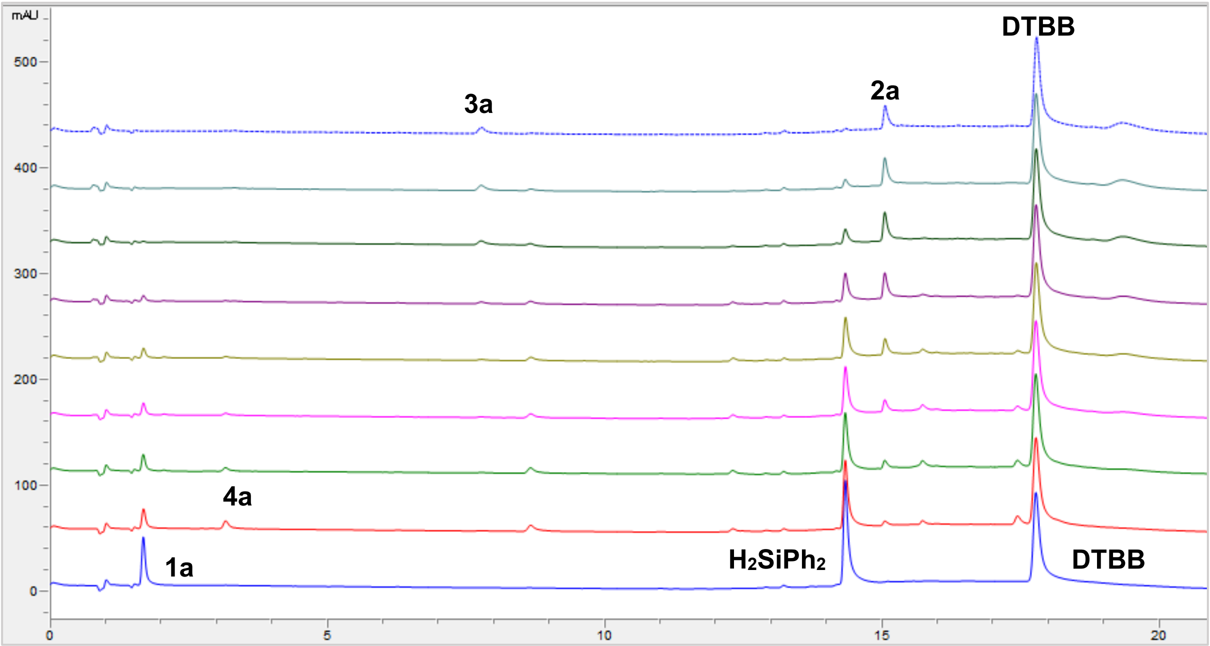

Figure S-104. HPLC analysis over time of electroreductive silylation of **1a** (0.02 M) 5 equiv. diphenylsilane at 10 mA from 0-time (bottom) to 3 h (top) with 4,4'-di-*tert*-butylbiphenyl (DTBB) as HPLC standard. Retention times for **1a** (R_t = 1.6 min), **4a** (R_t = 3.3 min), **3a** (R_t = 7.7 min), H_2SiPh_2 (R_t = 14.3 min), **2a** (R_t = 15.0 min), DTBB (R_t = 17.7 min). For reaction details see Figure S-3.

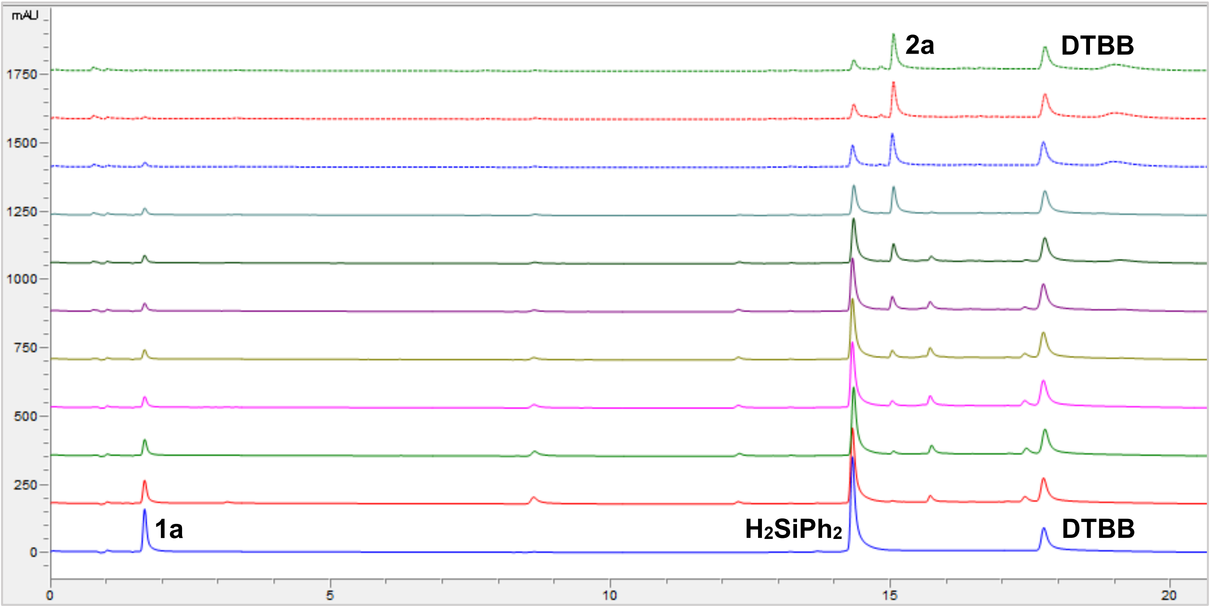

Figure S-105. HPLC analysis over time of electroreductive silylation of **1a** (0.1 M) 5 equiv. diphenylsilane at 5 mA from 0-time (bottom) to 3 h (top) with 4,4'-di-*tert*-butylbiphenyl (DTBB) as HPLC standard. Retention times for **1a** (R_t = 1.6 min), **4a** (R_t = 3.3 min), **3a** (R_t = 7.7 min), H_2SiPh_2 (R_t = 14.3 min), **2a** (R_t = 15.0 min), DTBB (R_t = 17.7 min). For reaction details see Figure S-4.
